# Functional group interaction profiles: a general treatment of solvent effects on non-covalent interactions†

**DOI:** 10.1039/d0sc01288b

**Published:** 2020-04-21

**Authors:** Mark D. Driver, Mark J. Williamson, Joanne L. Cook, Christopher A. Hunter

**Affiliations:** Department of Chemistry, University of Cambridge Lensfield Road Cambridge CB2 1EW UK herchelsmith.orgchem@ch.cam.ac.uk +44 (0)1223 336710; Department of Chemistry, University of Sheffield Sheffield S3 7HF UK

## Abstract

Solvation has profound effects on the behaviour of supramolecular systems, but the effects can be difficult to predict even at a qualitative level. Functional group interaction profiles (FGIPs) provide a simple visual method for understanding how solvent affects the free energy contribution due to a single point interaction, such as a hydrogen bond, between two solute functional groups. A generalised theoretical approach has been developed, which allows calculation of FGIPs for any solvent or solvent mixture, and FGIPs for 300 different solvents have been produced, providing a comprehensive description of solvent effects on non-covalent chemistry. The free energy calculations have been validated using experimental measurements of association constants for hydrogen bonded complexes in multiple solvent mixtures. The calculated FGIPs provide good descriptions of the solvation of polar solutes, solvophobic interactions between non-polar solutes in polar solvents like water, and preferential solvation in solvent mixtures. Applications are explored of the use of FGIPs in drug design, for optimising receptor-ligand interactions, and in enantioselective catalysis for solvent selection to optimise selectivity.

## Abstract 2

Functional group interaction profiles are a quantitative tool for predicting the effect of solvent on the free energy changes associated with non-covalent interactions.

## Graphical abstract

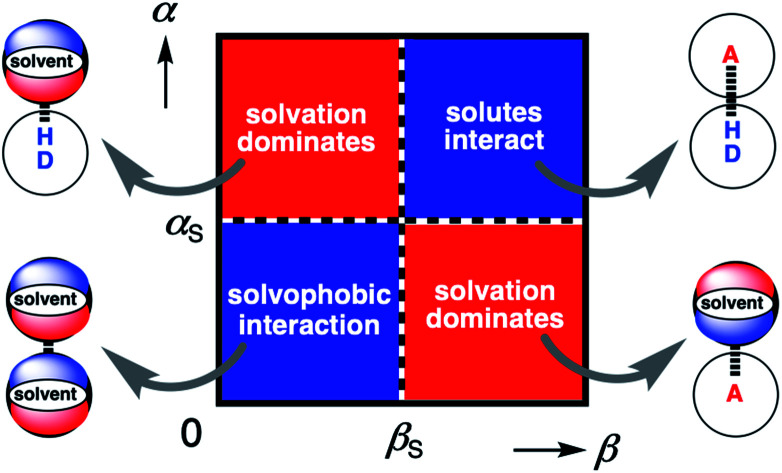

## 1 Introduction

Solvation plays an essential role in a wide range of different condensed phase phenomena. One of the major determinants of solvent effects is the non-covalent interactions made between solvent and solute. These interactions govern physical properties such as solubility, miscibility and vapour pressure,^1–4^ as well as chemical properties such as molecular recognition, supramolecular self-assembly and the rates of chemical reactions.^5–9^ The complexity of the network of coupled equilibria involved in solvation of molecular mixtures in different solvent environments has been a long standing challenge for theoretical prediction. Empirical solvent descriptors have proved valuable in extrapolating experimental data,^5,10–13^ and computational methods have been developed for including solvent effects in *ab initio* simulations of molecular properties.^14–18^ We have developed an approach to understanding solvation, which is based on experimental studies of pairwise interactions between hydrogen bonded solutes.^19^ This approach has provided simple rules of thumb for predicting how solvent will affect non-covalent interactions at a quantitative level. In this paper, the approach is generalised to provide a comprehensive description for any solvent environment.

**Fig. 1:**
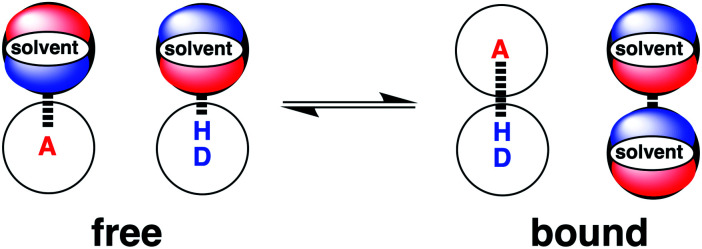
The solvent competition model for the formation of a hydrogen bonded complex between two solutes. The position of equilibrium is determined by the energies of the solute–solvent interactions in the free state, and the solute–solute and solvent–solvent interactions in the bound state. D a hydrogen bond donor solute and A represents a hydrogen bond acceptor solute.^19^

The association of two solutes in solution can be described by the equilibrium shown in Fig. 1. The equilibrium constant depends on stability of the four complexes shown, which can be estimated using eqn (1).^19^

1 Δ*G*°/kJ mol^−1^ = −(*α* − *α*_s_)(*β* − *β*_s_) + *c*

where Δ*G*° is the free energy change for formation of a 1 : 1 complex between two solutes that make a single hydrogen bond, *R* is the gas constant, *T* is the temperature, *α*, *β* are the solute hydrogen bond donor and acceptor parameters, *α*_s_, *β*_s_ are the solvent hydrogen bond parameters, and *c* is a constant which was experimentally determined to be 6 kJ mol^−1^ in carbon tetrachloride.^20^

The H-bond parameters used in eqn (1) have been experimentally determined for a wide range of different functional groups^13^ and can also be calculated from the values of the maxima and minima in *ab initio* molecular electrostatic potential surfaces.^21^ The validity of eqn (1) has been experimentally demonstrated for formation of 1 : 1 complexes between a wide variety of different solutes in a range of different solvents.^20,22–35^ The constant *c* in eqn (1) is related to the fact that organic solvents typically have a concentration of about 10 M, whereas the standard state for solutes is 1 M.^36^ The origin of the value *c* will be discussed in more detail below, but the focus of this paper is the development of a computational method for calculation of the first term in eqn (1) for any solvent environment. The first term in eqn (1) represents the free energy change associated with the exchange of polar interactions between solutes and solvent. Expressing this energy as ΔΔ*G*_FGI_ in eqn (2) provides a useful tool for predicting the free energy contribution that a specific functional group interaction makes to the stability of a supramolecular system where there are multiple non-covalent interactions.

2 ΔΔ*G*_FGI_/kJ mol^−1^ = −(*α* − *α*_s_)(*β* − *β*_s_)

**Fig. 2:**
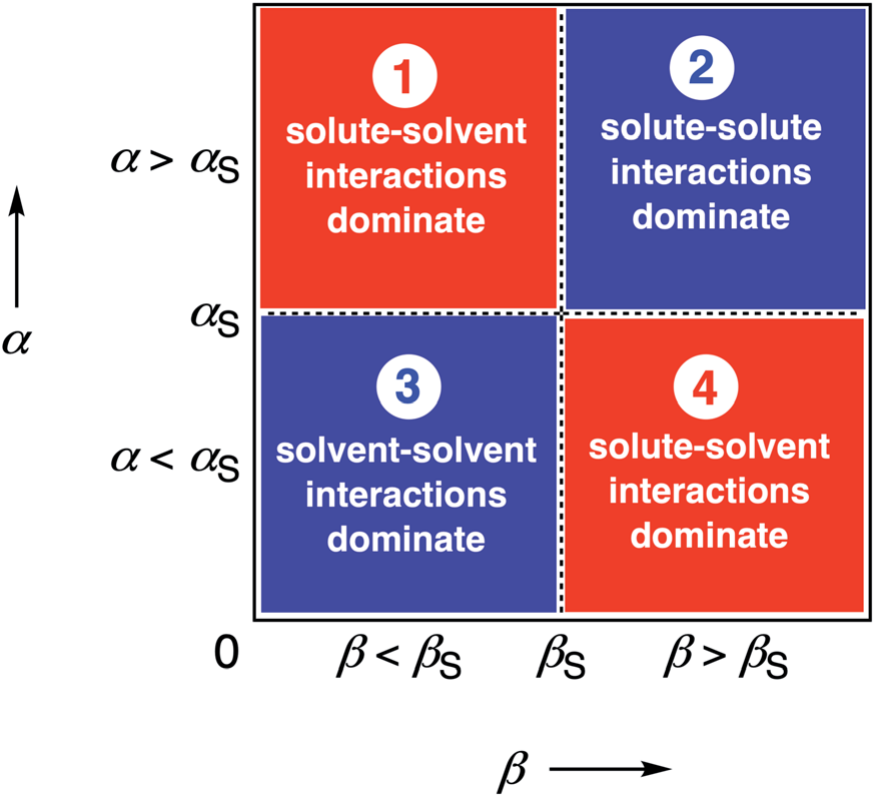
Generic Functional Group Interaction Profile (FGIP) for the free energy contribution due to the interaction of a hydrogen bond donor (*α*) with a hydrogen bond acceptor (*β*) in solvent S (ΔΔ*G*_FGI_). In the two red quadrants, ΔΔ*G*_FGI_ is positive, and the functional group interactions are unfavorable. In the two blue quadrants, ΔΔ*G*_FGI_ is negative, and the functional group interactions are favorable. The solvent parameters *α*_S_ and *β*_S_ set the boundaries between these quadrants.^19^

We call a two-dimensional plot of ΔΔ*G*_FGI_ calculated as a function of the two solute H-bond parameters, *α* and *β*, a Functional Group Interaction Profile (FGIP).^19^ A FGIP shows the free energy contribution for all possible solute–solute interactions in a given solvent and provides a simple visual method for understanding how that solvent affects non-covalent chemistry. Fig. 2 illustrates the general result. The FGIP is divided into four different regions, which are characterised by which of the four complexes shown in Fig. 1 is the most stable.

When the solute–solute interaction is the most stable of the four complexes, the equilibrium in Fig. 1 lies to the right, *i.e.* when *α* > *α*_S_ and *β* > *β*_S_, ΔΔ*G*_FGI_ will be negative. This regime is represented by quadrant 2 of Fig. 2.

When either of the two solute–solvent interactions are the most stable of the four species in Fig. 1, then the equilibrium lies to the left, *i.e.* when *α* < *α*_S_ and *β* > *β*_S_, or when *β* < *β*_S_ and *α* > *α*_S_, ΔΔ*G*_FGI_ will be positive. These two regimes are represented by quadrants 1 and 4 in Fig. 2.

If the solvent–solvent interaction is the most stable of the four species in Fig. 1, the equilibrium will lie to the right, *i.e.* when *α* < *α*_S_ and *β* < *β*_S_, ΔΔ*G*_FGI_ will be negative. This regime describes solvophobic interactions and is represented by quadrant 3 in Fig. 2.

The boundaries between the four regimes in the FGIP are defined by the lines *α* = *α*_S_ and *β* = *β*_S_ where like for like interactions between solvent and solute are exchanged.

In practice, eqn (2) overestimates the magnitude of solvophobic interactions, and a more complicated formulation was developed to accurately describe the hydrophobic effect in water.^19^ However, the major limitation of eqn (2) is that the solvent is described by a single type of hydrogen bond donor and a single type of hydrogen bond acceptor. Thus eqn (2) cannot be used to construct the FGIP for solvents like alcohols that have both OH and CH donors. Another limitation is that the stability of H-bonded complexes is known to depend on the concentrations of the solvating functional groups as well as their polarity, and eqn (2) does not capture any information about solvent concentration.^25^ Solvent mixtures are similarly beyond the scope of eqn (2). In this paper, we develop a generalised treatment that allows calculation of FGIPs for any solvent composition and illustrate the power of the approach by providing FGIPs for about 300 different solvents and solvent mixtures.

## 2 Approach

We have previously shown that it is possible to describe the non-covalent interactions of a molecule with its environment by representing the molecular surface as a discrete set of surface site interaction points (SSIP). The positions and values (*ε*_*i*_) of each SSIP can be calculated from the gas phase *ab initio* molecular electrostatic potential surface using a footprinting algorithm.^21^ The calculated SSIP interaction parameter, *ε*_*i*_, is equivalent to the experimentally determined hydrogen bond donor parameter (*α*) for positive sites or the acceptor parameter (−*β*) for negative sites.^19^ We have also previously described the surface site interaction model for liquids at equilibrium (SSIMPLE) algorithm for calculating solvation free energies using this SSIP description of molecular surfaces.^36^ In this paper, solvation energies will be calculated using values of *ε* to describe the interaction properties of the solutes in the SSIMPLE algorithm, but the resulting FGIPs will be plotted as a function of the corresponding values of *α* and *β* to make explicit the connection with the experimentally determined solute hydrogen bond parameters.

Briefly in SSIMPLE to describe a liquid, SSIP interactions are treated in a pairwise manner, such that the association constant for interaction between the ith and jth SSIP, *K*_*ij*_, is given by eqn (3).3
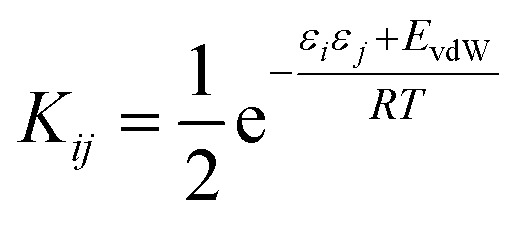
where *E*_vdW_ = −5.6 kJ mol^−1^.^37^

The interaction energy is made up of a polar term and a non-polar term, *E*_vdW_, which is the energy of the van der Waals interaction between two SSIPs. For repulsive interactions (*i.e. ε*_*i*_ and *ε*_*j*_ have the same sign), it is assumed that a state can be found where the polar sites are misaligned such that only non-directional van der Waals interactions are made, and the polar interaction term, *ε*_*i*_*ε*_*j*_, is set to zero. The standard state used to ensure *K*_*ij*_ is dimensionless is the maximum theoretical density of SSIPs, *c*_max_ = 300 M.^36^ The speciation of all SSIP contacts in the liquid phase can then be calculated.

The free energy of solvation of the SSIP that represents solute 1, Δ*G*_S_(1), can be calculated by considering the concentration of this SSIP that is not bonded to a solvent SSIP ([1_nb_]). Δ*G*_S_ in eqn (4) is the free energy of transfer of solute 1 from a reference state, which corresponds to a dilute gas where there are no SSIP interactions.4
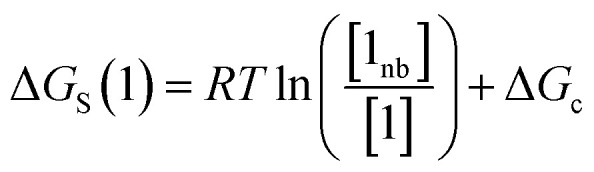
where [1] is the total concentration of SSIP 1 in the phase, and Δ*G*_c_ is the confinement energy.

The first term in eqn (4) describes the interactions made by the solute SSIP with the solvent SSIPs. The second term in eqn (4), Δ*G*_c_, corrects for the increased probability of interaction between SSIPs when they are confined to a condensed phase.^36^ This confinement energy affects the free energy of transfer between two phases of different SSIP density, but for processes that take place within the same phase, such as solute complexation, Δ*G*_c_ will be the same in the free and bound states and cancels out.

In order to use the solvation energies calculated with SSIMPLE to describe the free state in Fig. 1, the free energy of the bound state must be defined relative to the same non-bonded reference state. Therefore we require the probability that the solute SSIPs do not interact with one another in a phase that describes the bound state. We consider the bound state to be a phase where only the two solute SSIPs are present and the total SSIP concentration is the same as the bulk liquid. The total concentrations of each SSIP in the bound state, [1] and [2], are given by eqn (5) and (6).

5 [1] = [1_nb_] + 2*K*_12_[1_nb_][2_nb_] + 2*K*_11_[1_nb_]^2^

6 [2] = [2_nb_] + 2*K*_12_[1_nb_][2_nb_] + 2*K*_22_[2_nb_]^2^

where [1_nb_] and [2_nb_] are the non-bonded concentrations of the two solute SSIPs in the bound state, *K*_12_, *K*_11_ and *K*_22_ are the association constants for the interactions between solute SSIPs, and the factor of 2 is a statistical factor that accounts for the fact that complexes 1·2 and 2·1 are equivalent.

The total concentrations of the two SSIPs in the bound state are the same, and because the self-interactions are both repulsive, *K*_11_ and *K*_22_ are both equal to *K*_vdW_. The non-bonded concentrations, [1_nb_] and [2_nb_], are therefore equal.

Rearrangement of eqn (5) and (6) gives eqn (7).7
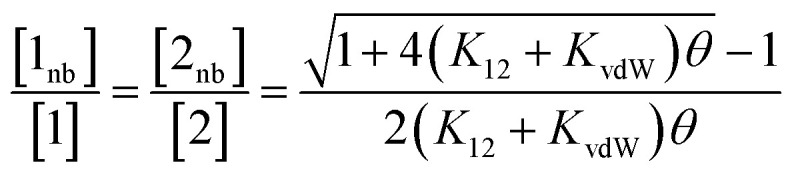


Thus the free energy for transfer of a solute SSIP from the reference state to the bound state is given by Δ*G*_B_ (eqn (8)). As for the solvation energy, the first term describes the SSIP interactions made in the bound state, and the second term corrects for the probability of confinement in a condensed phase.8
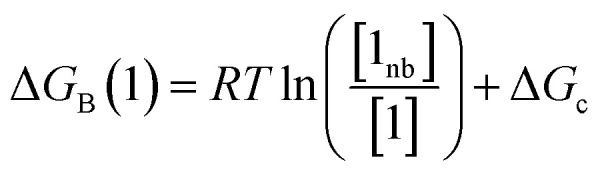


Thus eqn (4) can be used to calculate the free energy of a solute in the free state, and eqn (8) can be used to calculate the free energy of a solute in the bound state, both relative to the same reference state. These equations can be combined to give a calculated value of ΔΔ*G*_FGI_ (eqn (9)).

9 ΔΔ*G*_FGI_ = Δ*G*_B_(1) + Δ*G*_B_(2) − Δ*G*_S_(1) − Δ*G*_S_(2)

**Fig. 3:**
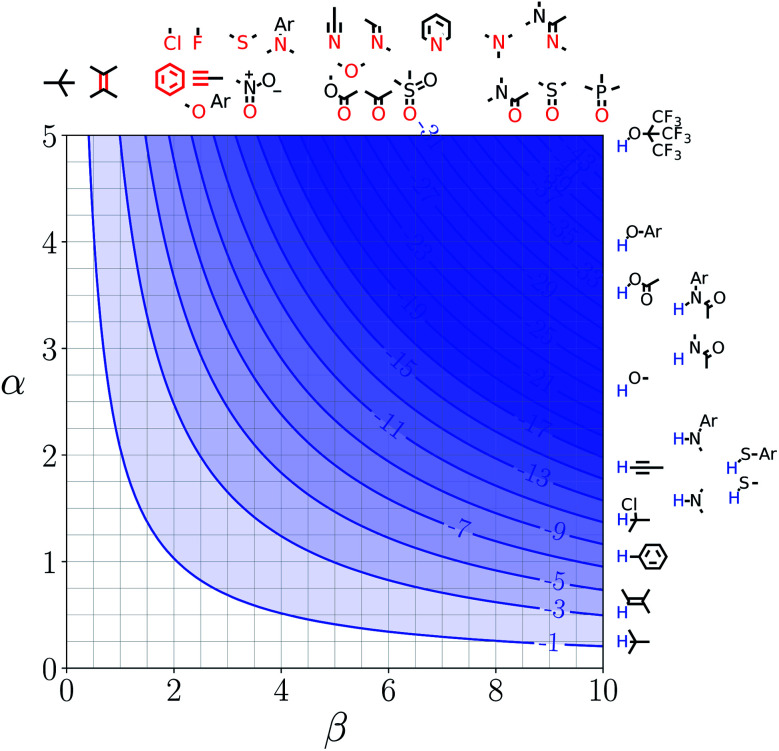
FGIP for the interaction of two solutes in a hypothetical room temperature liquid state of a noble gas where the concentration of solvent SSIPs is 160 M (ΔΔ*G*_FGI_ in kJ mol^−1^).

**Fig. 4:**
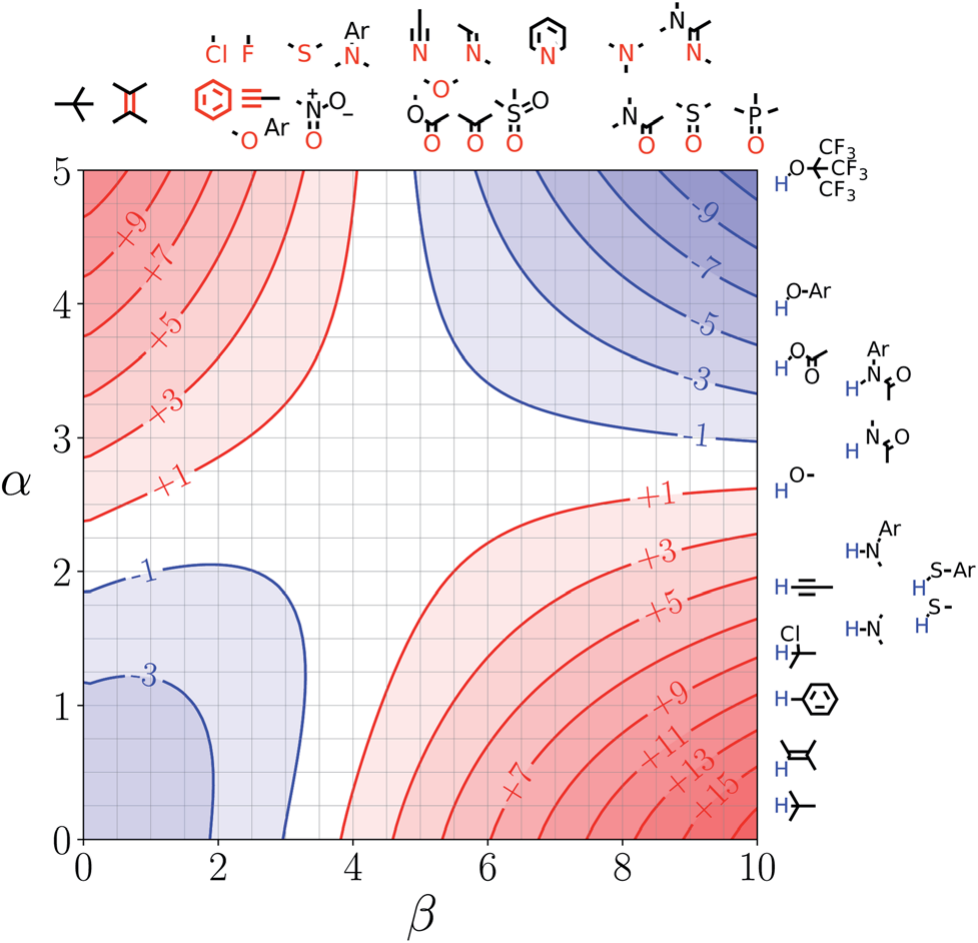
FGIP for the interaction of two solutes in water at 298 K (ΔΔ*G*_FGI_ in kJ mol^−1^). The solute–solute interactions are favourable in the blue region, and unfavourable in the red region.

Note that the second term in eqn (4) and (8), which is associated with the confinement energy, cancels out in this analysis, so the value ΔΔ*G*_FGI_ depends only on the relative probability of SSIP interactions in the free and bound states. The key feature of this treatment is that the value of ΔΔ*G*_FGI_ is zero when *α* = *α*_S_ and *β* = *β*_S_, as demonstrated in Fig. 3 and 4. Fig. 3 shows the FGIP for a hypothetical room temperature liquid state of a nobel gas, where there are no polar interactions (*ε*_*i*_ is zero for all solvent SSIPs). The value of ΔΔ*G*_FGI_ at the origin is zero as required. Fig. 4 shows the FGIP for water, where polar interactions dominate. The value of ΔΔ*G*_FGI_ is zero at the centre of the FGIP (*α* = *α*_S_ = 2.8, *β* = *β*_S_ = 4.5), where the solutes and solvent have the same polarity.

Representative hydrogen bond acceptor functional groups are illustrated along the top of Fig. 3 and 4. The hydrogen bond acceptor interaction sites are colored red and are aligned with the corresponding *β* values. Representative hydrogen bond donor functional groups are illustrated down the right side of Fig. 3 and 4. The hydrogen bond interaction sites are colored blue and are aligned with the corresponding *α* values. The interaction between any pair of functional groups can simply be read from these plots. For example, the interaction between a phosphine oxide (*β* ≈ 10) and a phenol (*α* ≈ 4) is about −37 kJ mol^−1^ in the hypothetical liquid state of a noble gas and about −7 kJ mol^−1^ in water. This interaction is in the blue region for both FGIPS, which corresponds to a favourable interaction. The interaction of an aryl CH donor (*α* ≈ 1) with a phosphine oxide is quite different. In water, this interaction falls in the red region, which corresponds to an unfavourable interaction, and is worth +11 kJ mol^−1^. In the hypothetical liquid state of a noble gas, this CH–O H-bond is in the blue region and corresponds to a favourable contribution to the free energy of binding of −7 kJ mol^−1^.

## 3 Results

The SSIP representation of 261 different solvents was calculated as described previously (see ESI† for details).^21^ Experimental measurements of solvent effects on the stabilities of hydrogen bonded complexes have shown that hydrogen bond parameters determined for isolated molecules generally provide a good description of the corresponding solvent parameters, *i.e.* the hydrogen bond parameters for a specific molecule are independent of whether the molecule is the acting as the solvent or solute.^36^ The exception is alcohols, which are more polar solvents than would be expected based on their solute properties.^22,34^ Self-association of alcohols leads to polarisation of the hydroxyl groups at the ends of oligomeric hydrogen bonded chains, and experimental hydrogen bond parameters have been measured for these sites.^34^ The self-assembly of alcohols is concentration dependent and involves the formation of both cyclic and linear species, but at the concentrations that correspond to the bulk solvent, linear chains dominate, and so solvation is determined by the properties of the chain end hydroxyl groups. The experimentally measured chain end hydrogen bond parameters were therefore used to represent alcohol hydroxyl group SSIPs in all calculations below.

The SSIMPLE algorithm was used to calculate values of Δ*G*_S_ for all values of solute SSIP between −10 and 5. Eqn (9) was then used to produce the FGIP for each of the 261 solvents. The complete set of FGIPs for all solvents is provided in the ESI,† but we will highlight some of the key features with selected examples.

### Mixed polarity solvents

**Fig. 5:**
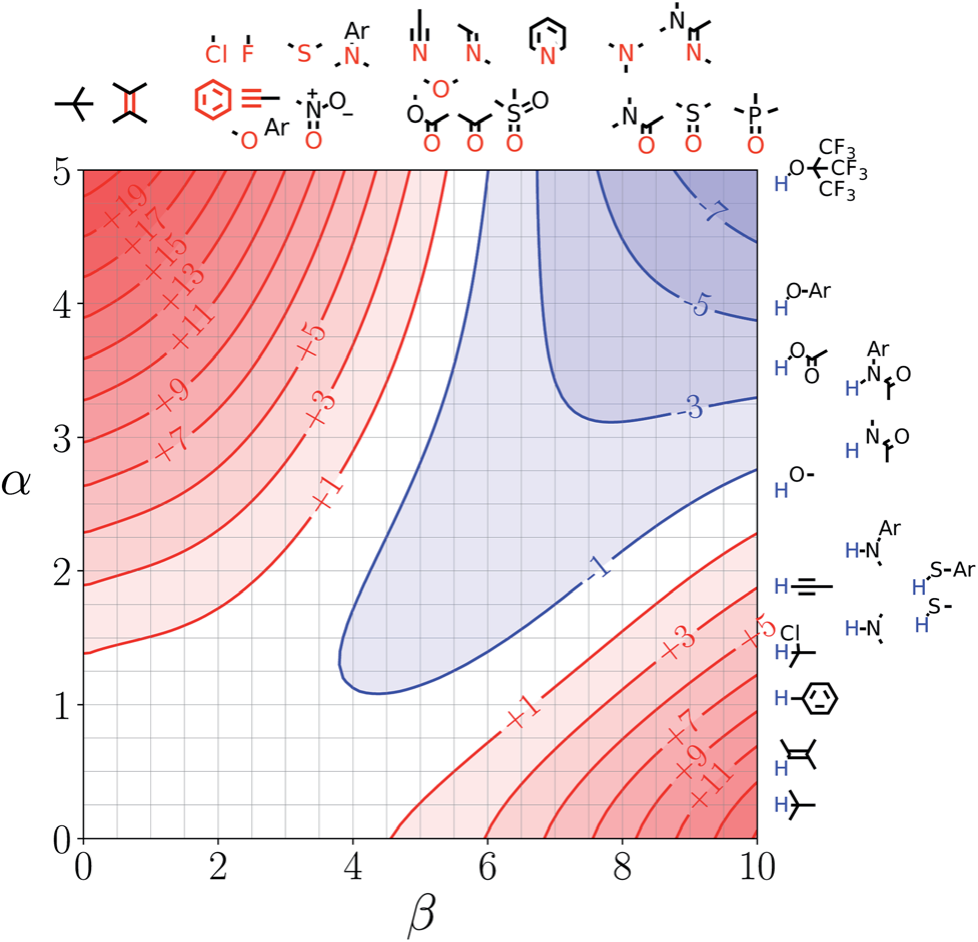
FGIP for the interaction of two solutes in ethanol at 298 K (ΔΔ*G*_FGI_ in kJ mol^−1^). The solute–solute interactions are favourable in the blue region, and unfavourable in the red region.

Fig. 5 shows the FGIP for ethanol, a solvent which contains both polar and non-polar SSIPs. Comparison with the FGIP for water in Fig. 4 shows that the effect of the ethyl group in ethanol is to eliminate the hydrophobic quadrant observed for water (quadrant 3 in Fig. 2). The behaviour of ethanol as a solvent is a consequence of preferential solvation. In water, non-polar solutes can only interact with the polar solvent SSIPs associated with the hydrogen bond donor and acceptor sites, which leads to poor solvation and favourable interactions between two non-polar solutes, *i.e.* the hydrophobic effect. In ethanol, non-polar solutes can choose between interaction with the polar solvent SSIPs associated with the hydroxyl group or the non-polar SSIPs associated with the ethyl group. The most favourable solvent–solute interactions are more highly populated, and so non-polar solutes interact preferentially with the non-polar solvent SSIPs in ethanol, which leads to good solvation and negligible solute–solute interactions in the bottom left region of the FGIP. These preferential solvation effects are also important in solvent mixtures and will be discussed further below.

### Solvent functional group polarity

**Fig. 6:**
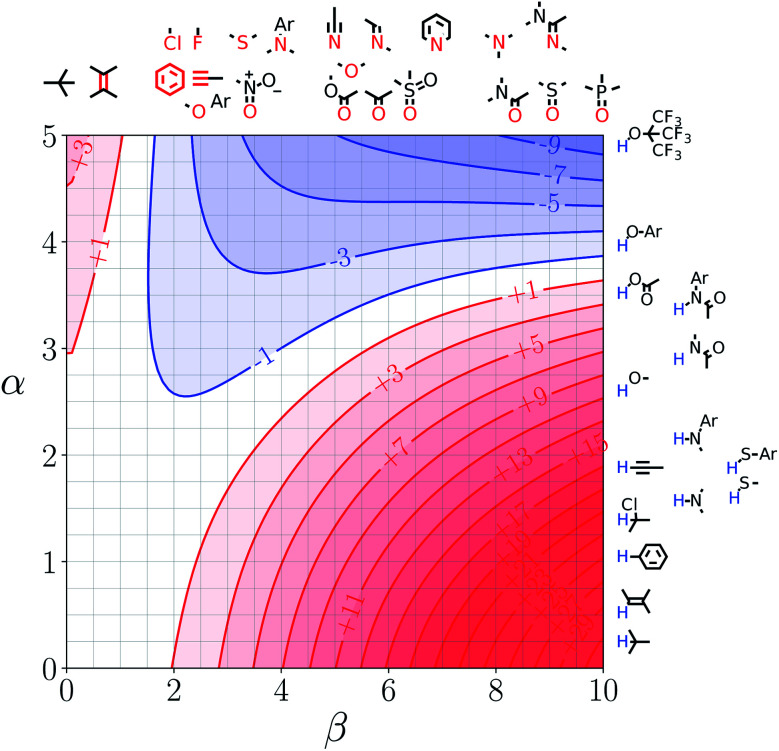
FGIP for the interaction of two solutes in 1,1,1,3,3,3-hexafluoro-2-propanol at 298 K (ΔΔ*G*_FGI_ in kJ mol^−1^). The solute–solute interactions in the blue region, and unfavourable in the red region.

Fig. 6 shows the FGIP for 1,1,1,3,3,3-hexafluoro-2-propanol (HFIP). The trifluoromethyl groups are associated with non-polar SSIPs, so the solvophobic quadrant is eliminated in this FGIP, as for ethanol. The effect of the trifluoromethyl groups on the polar SSIPs is to make the hydroxyl hydrogen bond donor SSIP more polar than ethanol (+4.3 compared to +3.5) and to make the hydroxyl hydrogen bond acceptor SSIP less polar (−2.7 compared with −6.9). The result is that quadrant 2 of the FGIP is compressed towards the top of the plot, because only very polar hydrogen bond donors can compete with the HFIP hydroxyl group and quadrant 1 is almost absent, because HFIP is a very poor hydrogen bond acceptor.

### Solvent functional group concentration

**Fig. 7:**
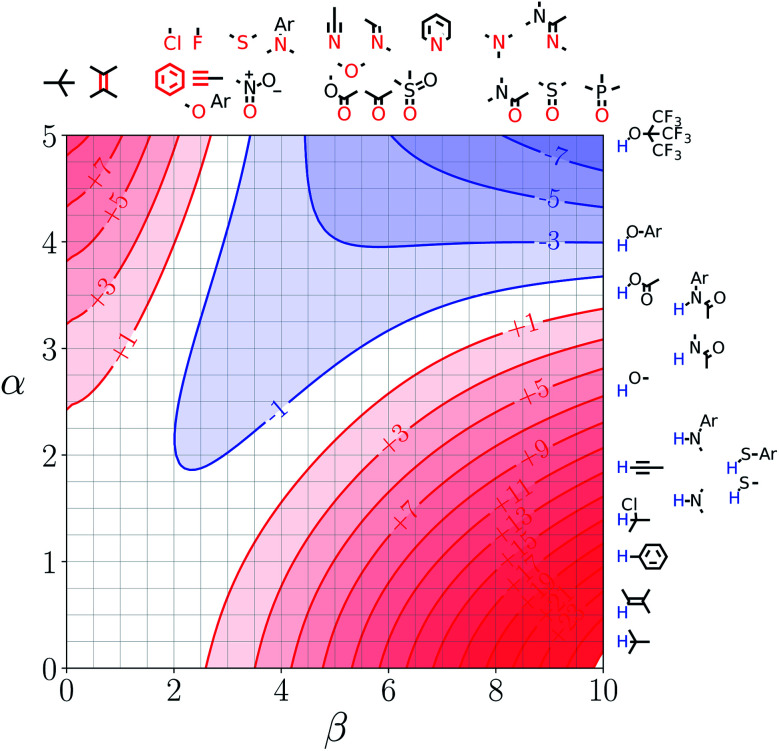
FGIP for the interaction of two solutes in formic acid at 298 K (ΔΔ*G*_FGI_ in kJ mol^−1^). The solute–solute interactions in the blue region, and unfavourable in the red region.

**Fig. 8:**
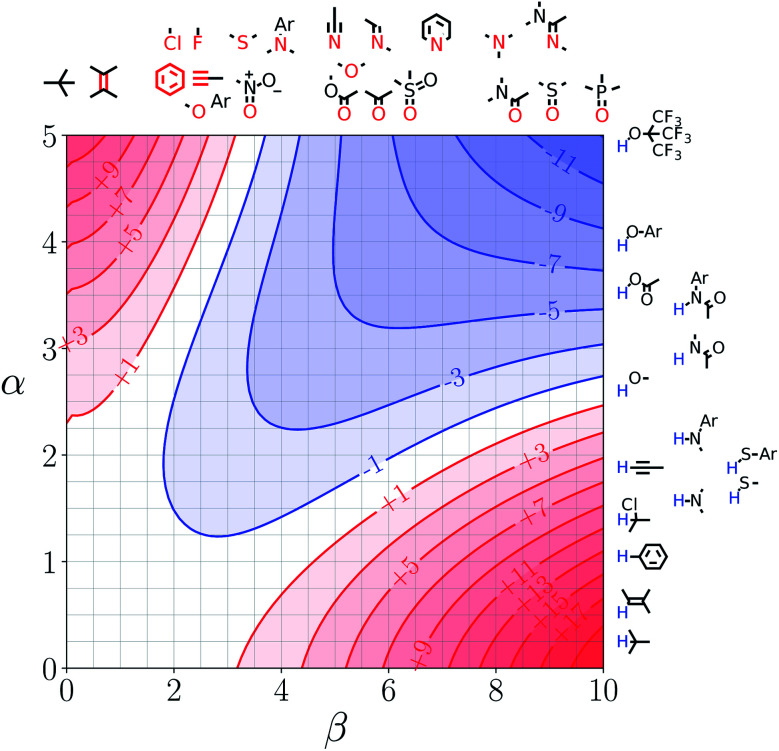
FGIP for the interaction of two solutes in heptanoic acid at 298 K (ΔΔ*G*_FGI_ in kJ mol^−1^). The solute–solute interactions in the blue region, and unfavourable in the red region.

Fig. 7 and 8 show the FGIPs for formic acid and heptanoic acid. The polarities of the SSIPs in these two solvents are very similar. The major difference between the two solvents is the relative concentration of the polar and non-polar SSIPs. The polar SSIPs associated with the carboxylic acid group are about 4 times less concentrated in heptanoic acid, and the result is that interactions between polar solutes are significantly more favourable in this solvent compared with formic acid. Thus quadrant 2 covers a much larger area of the heptanoic acid FGIP, and interactions between the most polar solutes in the top right corner of the FGIP are more favourable: −11 kJ mol^−1^*versus* −7 kJ mol^−1^ in formic acid.

### Solvent mixtures

**Fig. 9:**
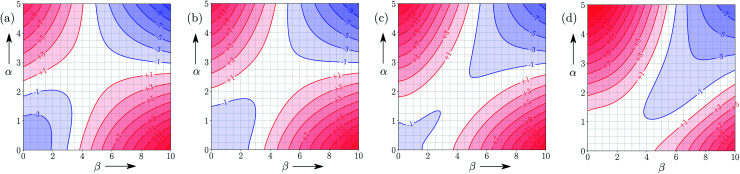
FGIPs for the interaction of two solutes in (a) 100% water 0% ethanol (b) 70% water 30% ethanol (c) 30% water 70% ethanol (d) 0% water 100% ethanol mixtures by volume at 298 K (ΔΔ*G*_FGI_ in kJ mol^−1^). The solute–solute interactions are favourable in the blue region, and unfavourable in the red region.

Fig. 9 shows FGIPs for mixtures of water and ethanol in different proportions (see ESI† for more compositions). The size of the hydrophobic region decreases with the amount of ethanol, because there is a corresponding increase in the concentration of non-polar SSIPs.

**Fig. 10:**
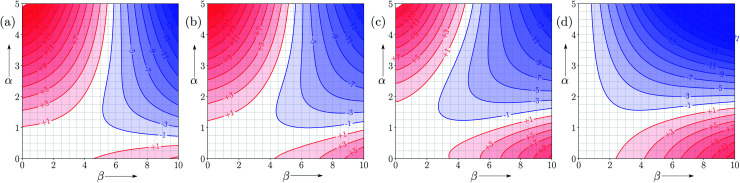
FGIPs for the interaction of two solutes in (a) 100% THF 0% chloroform (b) 70% THF 30% chloroform (c) 30% THF 70% chloroform (d) 0% THF 100% chloroform mixtures by volume at 298 K (ΔΔ*G*_FGI_ in kJ mol^−1^). The solute–solute interactions are favourable in the blue region, and unfavourable in the red region.

Fig. 10 shows FGIPs for mixtures of tetrahydrofuran (THF) and chloroform (see ESI† for more compositions). The FGIPs for the pure solvents show only two of the quadrants from Fig. 2. In THF, only quadrants 1 and 2 appear. The reason is that the most positive SSIP in THF is +0.6, so the effective value of *α*_S_ is close to zero, and quadrants 3 and 4 disappear. The most negative SSIP in THF has a value of −6.3, so *β*_S_ falls in the middle of the *β* scale, splitting the FGIP into two regions of similar area. In chloroform, only quadrants 2 and 4 appear. The reason is that the most negative SSIP in chloroform is −0.5, so the effective value of *β*_S_ is close to zero, and quadrants 1 and 3 disappear. The most positive SSIP in chloroform has a value of +2.2, so *α*_S_ falls in the middle of the *α* scale, splitting the FGIP into two regions of similar area. Thus THF is a moderately good acceptor and solvates hydrogen bond donor solutes well, and chloroform is a moderately good donor and solvates hydrogen bond acceptors well. In mixtures of THF and chloroform, preferential solvation leads to good solvation of hydrogen bond donor solutes by THF and good solvation of hydrogen bond acceptor solutes by chloroform. As a result, interactions between polar solutes are less favourable in mixtures than in either of the two pure solvents.^23^ The FGIPs for THF–chloroform mixtures in Fig. 10(b) and (c) are effectively a combination of the red quadrants from the two pure solvent FGIPs.

### Experimental validation

**Fig. 11:**
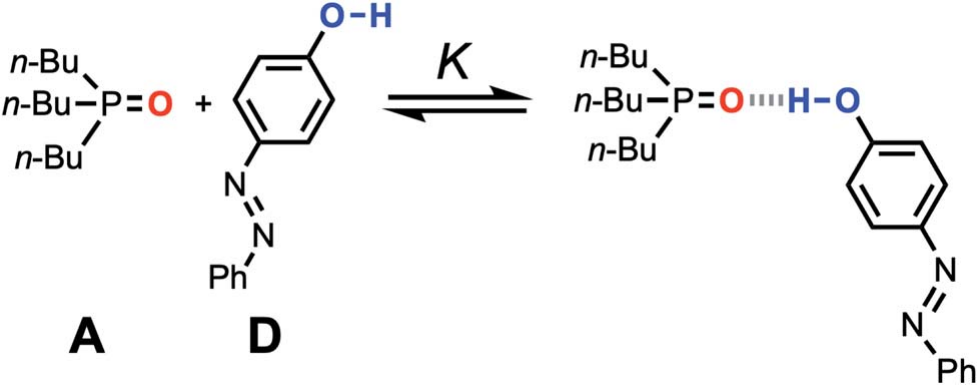
Association equilibrium for 1 : 1 complex between tri-*n*-butylphosphine oxide (hydrogen acceptor A) and 4-phenyl azophenol (hydrogen bond donor D).

The accuracy of the solvation energies used to calculate the FGIPs for solvent mixtures can be validated by experimental measurement of association constants for hydrogen bonded complexes. High throughput titration experiments using a UV-vis plate reader were used to measure association constants for formation of the 1 : 1 complex shown in Fig. 11 across the entire composition range for THF–chloroform mixtures (see ESI† for details).^27,29,30,32–35^ These experimental measurements can be compared with the solvation energies Δ*G*_S_ calculated using the SSIMPLE approach as follows.

The experimentally measured free energy change for formation of a 1 : 1 complex between a hydrogen acceptor A and a hydrogen bond donor D, Δ*G*°, is defined by eqn (10).10
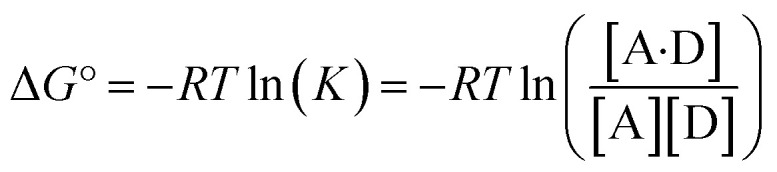


The concentration of the A·D complex is related to the concentration of solute–solute interactions in an SSIMPLE calculation by eqn (11).11
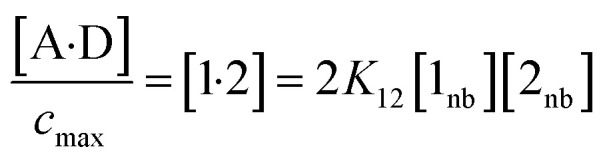
where [A·D] is the concentration of the 1 : 1 complex relative to the conventional 1 M standard state, [1·2] is the concentration of interacting solute SSIPs relative to *c*_max_, *K*_12_ is calculated from the solute SSIP values *ε*_1_ and *ε*_2_ using eqn (3), and [1_nb_] and [2_nb_] are the non-bonded concentrations of the solute SSIPs that do not interact with each other or the solvent, defined relative to *c*_max_.

The concentration of free hydrogen bond donor [D] is given by the concentration of solute 1 SSIP that is not bound to solute 2 SSIP in the SSIMPLE calculation (eqn (12)).12
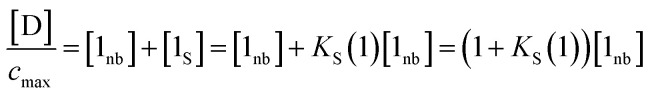
where [D] is the concentration of the free hydrogen bond donor relative to the conventional 1 M standard state, [1_S_] is the concentration of the solute 1 SSIP bound to solvent (all SSIP concentrations defined relative to *c*_max_), and *K*_S_(1) is the equilibrium constant for solvation of solute 1 SSIP as defined in eqn (13).13
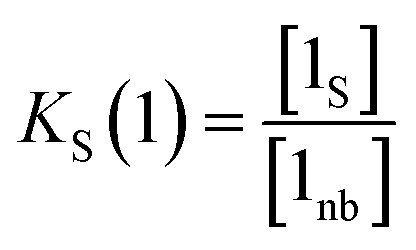


A similar expression can be written for [A] in terms of the concentration of solute 2 SSIP. Substitution for [A·D], [A] and [D] in eqn (10) gives eqn (14).14
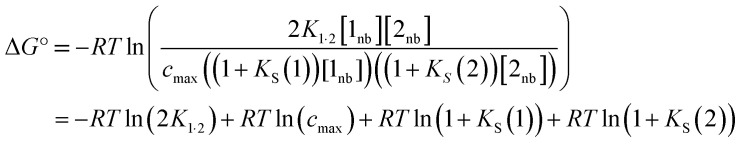


The solvation energy defined in eqn (4) can also be expressed in terms of *K*_S_ as follows (eqn (15)).15
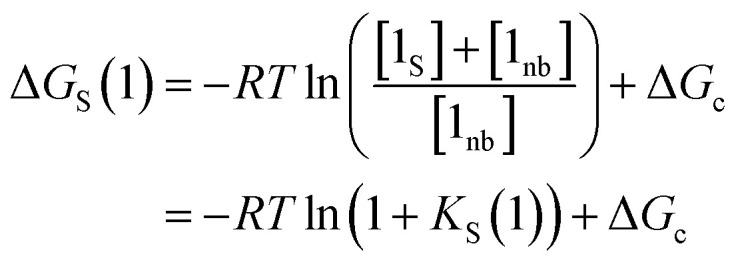


Substituting into in eqn (14) yields eqn (16), which defines the free energy for the formation of a 1 : 1 complex in terms of the SSIP values of the two solutes (*i.e. α* and *β*), the solvation energies of the two solutes, and some constants.

16 Δ*G*° = *ε*_1_*ε*_2_ + *E*_vdW_ + *RT* ln(*c*_max_) − Δ*G*_S_(1) − Δ*G*_S_(2) + 2Δ*G*_c_

**Fig. 12:**
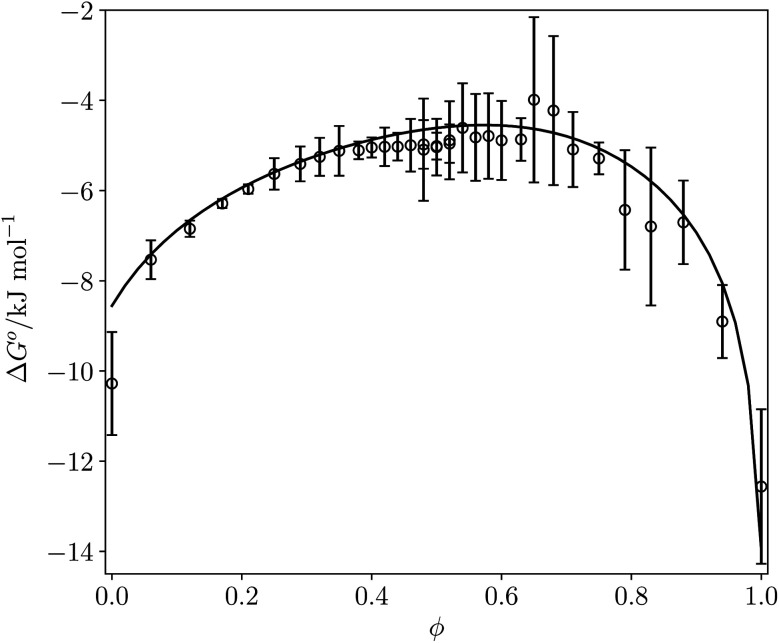
Δ*G*° (kJ mol^−1^) for formation of a 1 : 1 complex between tri-*n*-butylphosphine oxide (*β* = 10.7) and 4-phenyl azophenol (*α* = 4.1) in THF–chloroform mixtures. Experimental measurements (black circles) and calculated values using eqn (16) (black line) as function of chloroform volume fraction, *ϕ*. The experimental values are the average of five experiments with error bars at the 95% confidence limit.

Fig. 12 compares the values of Δ*G*° calculated using eqn (16) with the corresponding experimentally measured values for the azophenol·phosphine oxide complex as a function of solvent composition for THF–chloroform mixtures. The agreement is both qualitatively and quantitatively good. The calculated line closely follows the experimental data, and the largest difference is within 2 kJ mol^−1^ for pure THF. This agreement with experiment suggests that SSIMPLE provides an accurate description of such complex solvent environments and that the calculated FGIPs provide a realistic description of the solvation properties of liquids.

## 4 Origin of the constant *c*

Eqn (16) provides insight into the origin of the constant *c* in eqn (1), because this expression for Δ*G*° can be compared with the expression derived above for Δ*G*_FGI_ in eqn (9). The difference between these two expressions is equal to the constant *c* (eqn (17)).17
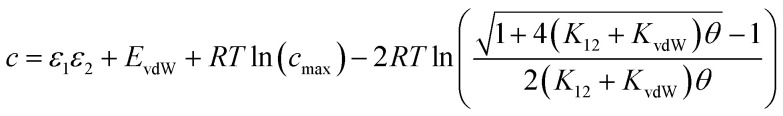


Eqn (17) suggests that *c* is not a constant but depends on the solute SSIP values, because *ε*_1_ and *ε*_2_ appear in the equation. However in the limit of tight binding for two polar solutes, *i.e.* when *K*_12_ is large, eqn (17) simplifies to give a constant value for *c* (eqn (18)).18
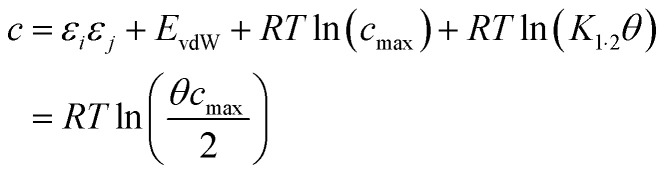


The concentration inside the bracket in eqn (18) is half the total concentration of solvent SSIPs, *i.e.* the concentration of solvent–solvent SSIP interactions if they were fully bound. This suggests that the origin of the constant in the eqn (1) is related to the concentration of solvent–solvent interactions. Experimentally determined equilibrium constants are conventionally defined relative to the 1 M standard state, and the concentration of solvent is conventionally ignored. However as Fig. 1 illustrates, complexation in solution is a competition between solvent–solute, solute–solute and solvent–solvent interactions, so the concentration of the solvent–solvent complex shown in Fig. 1 should be considered in the expression for the equilibrium constant (eqn (19)).19
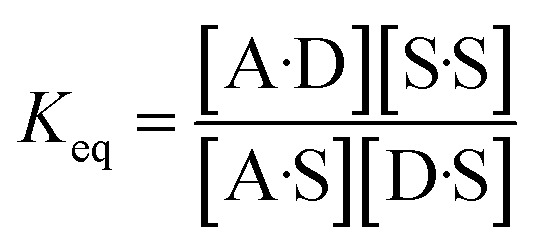
where [S·S] is the concentration of solvent–solvent interactions.

**Fig. 13:**
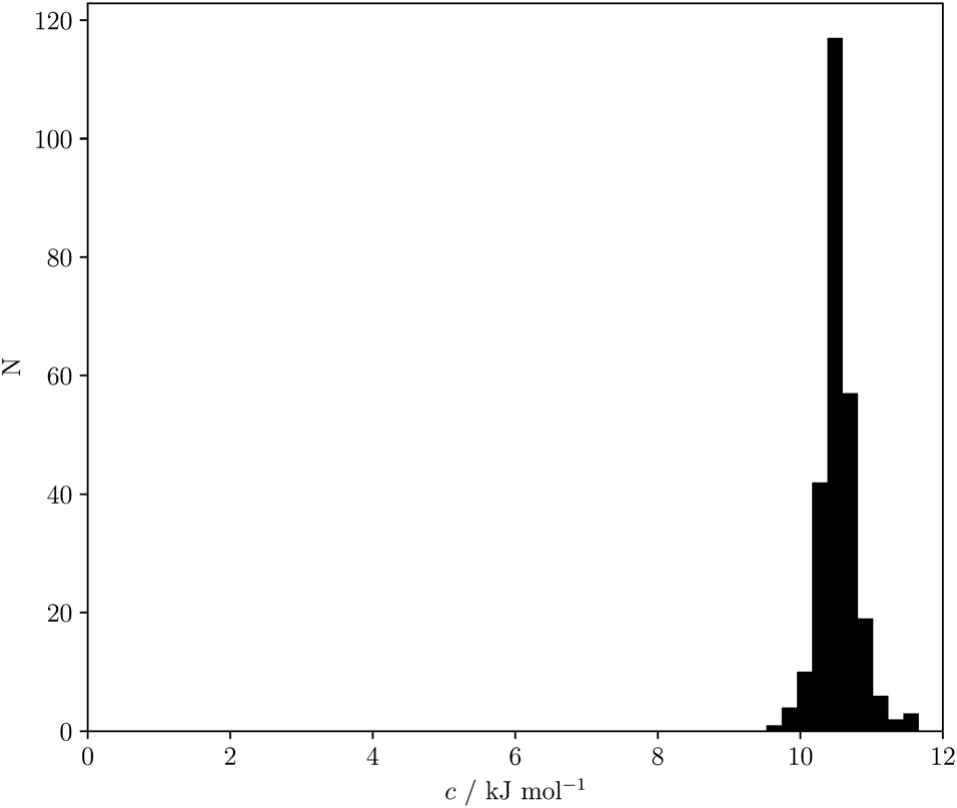
Frequency distribution for values of *c* calculated using eqn (18) for 261 solvents.

Eqn (18) and (19) both indicate that the constant *c* in eqn (1) accounts for the absence of the solvent concentration in the conventional definition of equilibrium constants. Eqn (18) provides an expression for *c* that is independent of the solute SSIP values, so it is possible to calculate values of *c* for different solvents. Fig. 13 shows the distribution of *c* values for the 261 pure solvents studied here. The value is more or less constant for all solvents at 10.5 ± 0.6 kJ mol^−1^.

**Fig. 14:**
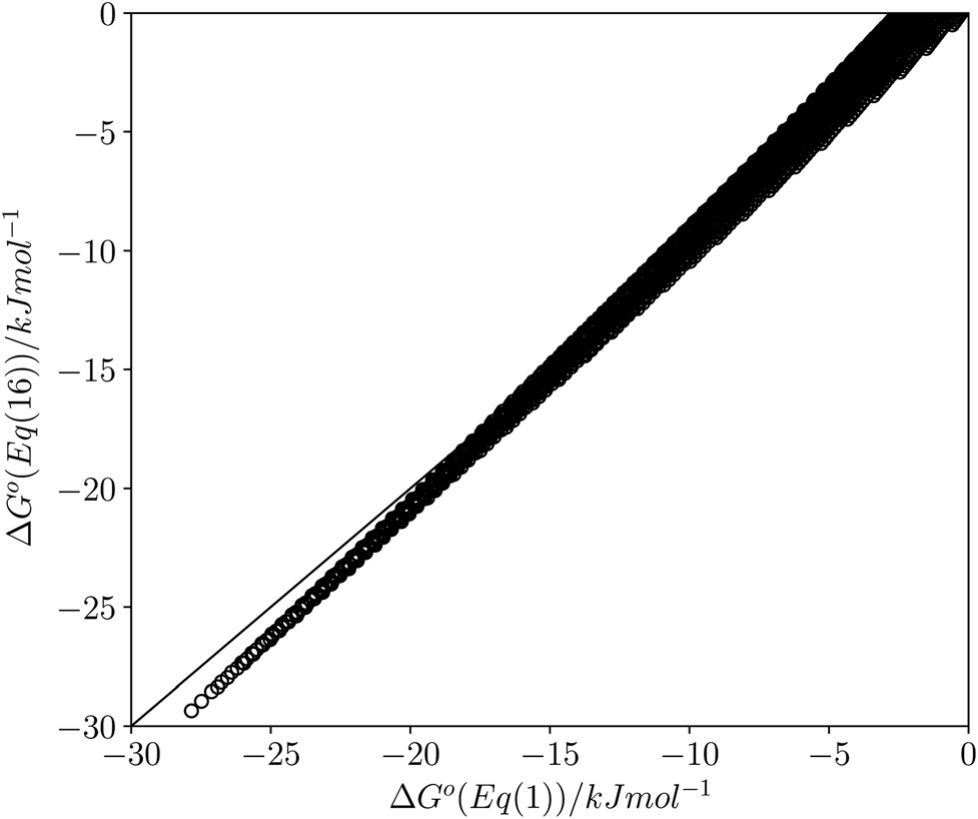
Comparison of values of Δ*G*° in carbon tetrachloride calculated using eqn (1) with values calculated using eqn (16) for all values of *ε*_1_ between 0 and +5 and all values of *ε*_2_ between 0 and −10 (0.1 increments). Black line is *y* = *x*.

This calculated value of *c* is some 4 kJ mol^−1^ higher than the experimentally determined value of 6 kJ mol^−1^ for carbon tetrachloride. There are some important differences between the experimentally derived eqn (1), which considers only the exchange of polar interaction sites, and the SSIMPLE calculation, which also takes into account non-polar van der Waals interactions and the concentrations of different interaction sites. This is presumably the origin of the difference between the two constants. A better comparison is therefore to look at values of Δ*G*° calculated using eqn (1) and (16). Fig. 14 shows a plot of Δ*G*° in carbon tetrachloride calculated using both methods for all solute combinations with *α* values between 0 and 5 and *β* values between 0 and 10 (in increments of 0.1). The agreement is much better than the difference in the values of *c* would suggest. There is generally good agreement between the absolute values of Δ*G*°. The largest deviations occur for the most polar and least polar interactions, where the SSIMPLE values are respectively 2 kJ mol^−1^ lower and higher than eqn (1).

## 5 How to use an FGIP

Next we will look at how FGIPs can be used in two different types of supramolecular design scenarios. The first example deals with choice of solute functional groups to optimise non-covalent interactions in drug design. The second example deals with choice of solvent to optimise non-covalent interactions that control selectivity in enantioselective catalysis.

**Fig. 15:**
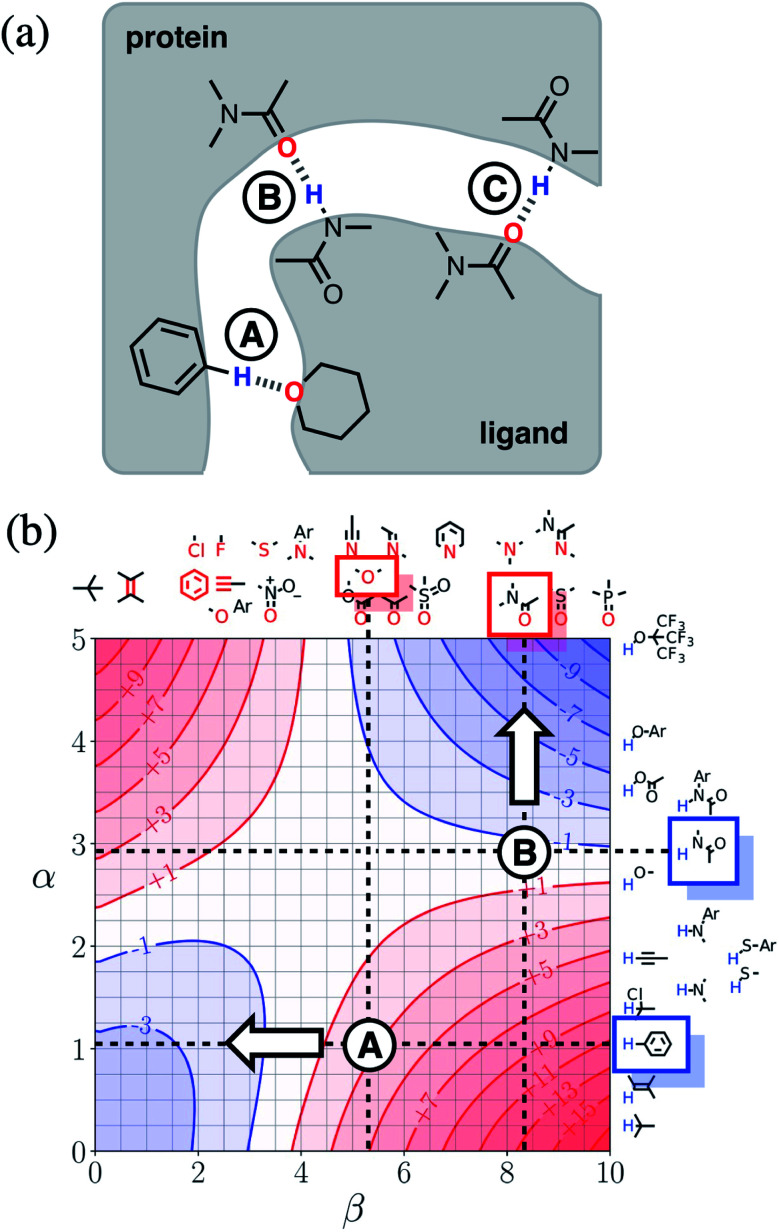
(a) Cartoon of the binding interface in a receptor–ligand complex, highlighting three different functional group interactions, labelled A, B and C. (b) The FGIP for water (ΔΔ*G*_FGI_ in kJ mol^−1^), highlighting the key functional groups involved in binding. Dotted lines are drawn horizontally at the hydrogen bond donor parameters of these functional groups and vertically at the hydrogen bond acceptor parameters. The points of intersection of the dotted lines that correspond to Interactions A and B are marked. Interaction C is not labelled, because it falls at the same point on the FGIP as Interaction B. The arrows indicate changes to the ligand hydrogen bond parameters that would make interactions A and B more favourable.

Fig. 15(a) shows a cartoon of an idealised binding interface between a ligand and a protein. Sites A, B and C indicate a CH–O interaction and two different amide–amide hydrogen bonding interactions. The FGIP for water shown in Fig. 15(b) can be used to evaluate the free energy contributions that each of these interactions makes to the overall stability of the complex. Interaction A falls in region 4 of the FGIP (*cf.*Fig. 2), so the CH–O interaction reduces the stability of the complex, due to the free energy penalty for desolvation of the ether oxygen. Interaction B falls on the borderline between regions 2 and 4, because the hydrogen bond donor parameter for the amide NH is approximately the same as the hydrogen bond donor parameter for water, *i.e. α* ≈ *α*_S_ ≈ 3. Thus the amide–amide hydrogen bond makes no contribution to the stability of the complex, because the free energy penalty for desolvation of the polar groups is exactly matched by the new hydrogen bond made in the complex. Interaction C is not shown in Fig. 15(b), because it is the same as Interaction B and falls at the same point on the FGIP.

Fig. 15(b) also illustrates how the FGIP can be used to develop strategies for optimising these interactions in order to increase the overall binding affinity. The arrow next to Interaction A in Fig. 15(b) indicates that decreasing the hydrogen bond acceptor strength would lead to a more favourable interaction. A less polar acceptor would reduce the desolvation penalty and move Interaction A into the solvophobic zone of the FGIP (region 3 in Fig. 2). For example, replacing the alkyl ether in the ligand with an aryl ether would give rise to a favourable hydrophobic interaction, making ΔΔ*G*_FGI_ more negative by about 5 kJ mol^−1^ (reading from the contours in Fig. 15(b)), and lead to an increase in binding affinity of an order of magnitude. The arrow next to Interaction B in Fig. 15(b) indicates that increasing the hydrogen bond donor strength would lead to a more favourable interaction. For example, replacing the amide group in the ligand with a phenol would make ΔΔ*G*_FGI_ more favourable by about 5 kJ mol^−1^, leading to an increase in binding affinity of an order of magnitude. The situation is quite different for Interaction C. Interaction C falls at the same point on the FGIP as Interaction B, but in this case, it is not possible to improve the binding affinity by changing the functional group on the ligand, the amide acceptor. A horizontal dotted line is drawn at *α* ≈ 3, which corresponds to the hydrogen bond donor parameter for the protein amide NH. This line falls almost exactly on the borderline between regions 2 and 4 of the FGIP, showing that ΔΔ*G*_FGI_ would be approximately zero for all hydrogen bond acceptor partners, regardless of polarity. Of course there are many other factors that govern overall binding affinity in addition to the properties of single point receptor–ligand interactions, but Fig. 15 shows that an FGIP can suggest useful strategies for guiding drug design, because it integrates interaction strength and desolvation on a free energy scale.

**Fig. 16:**
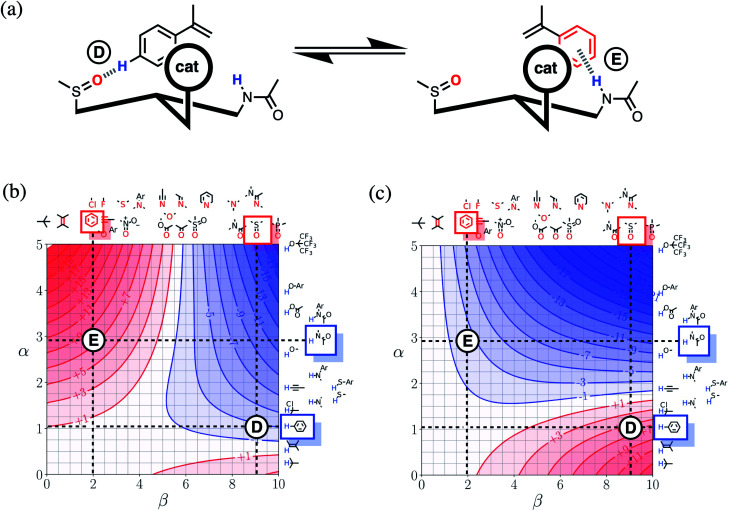
(a) Cartoon of the interaction of a substrate with an enantioselective catalyst, highlighting two different functional group interactions, labelled D and E. The catalytic site is labelled cat. (b) The FGIP for THF (ΔΔ*G*_FGI_ in kJ mol^−1^). (c) The FGIP for chloroform (ΔΔ*G*_FGI_ in kJ mol^−1^). The dotted lines on the FGIPs are drawn horizontally at the hydrogen bond donor parameters of the interacting functional groups and vertically at the hydrogen bond acceptor parameters. The points of intersection of the dotted lines that correspond to Interactions D and E are marked.

Fig. 16(a) shows a cartoon of an idealised interaction between a substrate and an enantioselective catalyst. The two modes of interaction present different faces of the prochiral substrate to the catalytic site (labelled cat) and would therefore lead to different stereochemical outcomes in the reaction. In one mode, there is a CH–O interaction between the substrate and the catalyst (labelled D), and in the other, there is a π-facial hydrogen bond between the substrate and the catalyst (labelled E). The FGIPs for THF and chloroform shown in Fig. 16(b) and (c) can be used to evaluate solvent effects on the free energy contributions that each of these interactions makes to the position of equilibrium in Fig. 16(a). Interaction D is favourable in THF (ΔΔ*G*_FGI_ ≈ −3 kJ mol^−1^ reading from the contours) and unfavourable in chloroform (+5 kJ mol^−1^). In contrast, Interaction E is unfavourable in THF (+7 kJ mol^−1^) and favourable in chloroform (−3 kJ mol^−1^). These differences in free energy of interaction can be used to select a solvent to favour a particular stereochemical outcome of the reaction. Thus we would predict that chloroform would favour one enantiomer and that THF would favour the other. These two solvents have been chosen to illustrate the approach, but the full set of FGIPs provided in the ESI† can be scrutinised to find the optimum solvent combination to favour/disfavour particular sets of non-covalent interactions for specific applications. Alternatively, the solvent-dependence of the enantiomeric excess of a reaction could be used to infer what kind of interactions are likely to be important in determining stereoselectivity for a particular catalyst.

## 6 Conclusions

Functional group interaction profiles (FGIP) provide a straightforward tool for visualising the effects of solvent on non-covalent interactions at a quantitative level. The original formulation of the FGIP was limited to simple solvents, because solvation was described as interaction with a single type of solvent hydrogen donor and a single type of solvent hydrogen bond acceptor.^19^ Most solvents and all solvent mixtures are composed of a more complicated collection of different types of interaction site, so a more sophisticated treatment is required. The theoretical basis for that treatment is described in this paper, providing the means to calculate FGIPs for any solvent environment.

The non-covalent interaction properties of 261 different solvent molecules have been characterised as a set of surface site interaction points (SSIPs), which were obtained by footprinting *ab initio* molecular electrostatic potential surfaces calculated for the isolated molecules in the gas phase. These solvent descriptions were then used to calculate solvation free energies for all possible solute SSIP values in pure solvents and in solvent mixtures. The results allow construction of FGIPs for the pairwise interaction of any two solutes in any solvent environment. The ESI† provides a point of reference with 300 such plots for both pure solvents and solvent mixtures. The examples illustrated in the main text show that the approach provides a good description of solvophobic effects, such as the hydrophobic effect, as well as polar solvent–solute interactions and selective solvation phenomena in solvent mixtures.

The validity of the approach has been demonstrated by experimentally measuring the equilibrium constant for formation of a hydrogen bonded complex across the full composition range of mixtures of chloroform and tetrahydrofuran. The calculated and experimental values of Δ*G*° are within 2 kJ mol^−1^ for all measurements. The theoretical analysis also provides insight into the factors that govern the free energy of complexation in the liquid state. There are three contributions: the exchange of polar interactions between solvent and solutes, which can be described by the hydrogen bond parameters, *α* and *β*; the exchange of non-polar van der Waals contacts, which usually cancel out; and a constant term associated with the fact that the concentration of solvent is significantly higher than the conventional standard state of 1 M for solutes. Thus the FGIPs described here not only provide a quantitative guide to solvent effects on the free energy contributions that can be expected for specific non-covalent interactions between different functional groups, but also provide a qualitative understanding of the relative magnitudes the different factors that influence the strengths of these interactions.

## Conflicts of interest

There are no conflicts to declare.

## Supplementary Material

SC-011-D0SC01288B-s001
