# Supplementary material for: Functional group interaction profiles: a general treatment of solvent effects on non-covalent interactions
Source: Chem Sci. 2020 Apr 21;11(17):4456–66. doi: 10.1039/d0sc01288b (PMC8159447; doi:10.1039/d0sc01288b)
Supplement: SC-011-D0SC01288B-s001 [file SC-011-D0SC01288B-s001.pdf]

Electronic Supplementary Material (ESI) for Chemical Science.
This journal is © The Royal Society of Chemistry 2020

Functional Group Interaction Profiles: A General Treatment of Solvent Effects on Non-Covalent Interactions.

Supplementary Information

Mark D. Driver,† Joanne L. Cook,‡ Mark J. Williamson,† and Christopher A. Hunter*,†

†*Department of Chemistry, University of Cambridge, Lensfield Road, Cambridge, UK*

‡*Department of Chemistry, University of Sheffield, Sheffield, S3 7HF , UK*

E-mail: herchelsmith.orgchem@ch.cam.ac.uk

Phone: +44 (0)1223 336710

Contents

Automated UV-Vis titrations **S13**

Pure Solvent information **S17**

Solvent SSIP Descriptions **S39**

FGIP plots for solvents **S63**

List of FGIPs

S3 FGIP for tetramethylsilane at 298K. S63

S4 FGIP for n-pentane at 298K. S64

S5 FGIP for 2-methylbutane at 298K. S65

S6 FGIP for n-hexane at 298K. S66

S7 FGIP for cyclohexane at 298K. S67

S8 FGIP for n-heptane at 298K. S68

S9 FGIP for n-octane at 298K. S69

S10 FGIP for 2,2,4-trimethylpentane at 298K. S70

S11 FGIP for n-decane at 298K. S71

S12 FGIP for n-dodecane at 298K. S72

S13 FGIP for n-hexadecane at 298K. S73

S14 FGIP for benzene at 298K. S74

S15 FGIP for toluene at 298K. S75

S16 FGIP for ortho-xylene at 298K. S76

S17 FGIP for meta-xylene at 298K. S77

S18 FGIP for para-xylene at 298K. S78

S19 FGIP for ethylbenzene at 298K. S79

S20 FGIP for isopropylbenzene at 298K. S80

S21 FGIP for 1,3,5-trimethylbenzene at 298K. S81

S22 FGIP for styrene at 298K. S82

S23 FGIP for 1,2,3,4-tetrahydronaphthalene at 298K. S83

S24 FGIP for cis-decalin at 298K. S84

S25 FGIP for water at 298K. S85

S26 FGIP for methanol at 298K. S86

S27 FGIP for ethanol at 298K. S87

S28 FGIP for 1-propanol at 298K. S88

S29 FGIP for 2-propanol at 298K. S89
S30 FGIP for 1-butanol at 298K. S90
S31 FGIP for 2-methyl-1-propanol at 298K. S91
S32 FGIP for 2-butanol at 298K. S92
S33 FGIP for 2-methyl-2-propanol at 298K. S93
S34 FGIP for 1-pentanol at 298K. S94
S35 FGIP for 3-methyl-1-butanol at 298K. S95
S36 FGIP for 2-methyl-2-butanol at 298K. S96
S37 FGIP for 1-hexanol at 298K. S97
S38 FGIP for cyclohexanol at 298K. S98
S39 FGIP for 1-octanol at 298K. S99
S40 FGIP for 1-decanol at 298K. S100
S41 FGIP for 1-dodecanol at 298K. S101
S42 FGIP for benzyl alcohol at 298K. S102
S43 FGIP for 2-phenylethanol at 298K. S103
S44 FGIP for allyl alcohol at 298K. S104
S45 FGIP for 2-chloroethanol at 298K. S105
S46 FGIP for 2-cyanoethanol at 298K. S106
S47 FGIP for 2,2,2-trifluoroethanol at 298K. S107
S48 FGIP for 1,1,1,3,3,3-hexafluoro-2-propanol at 298K. S108
S49 FGIP for 2-methoxyethanol at 298K. S109
S50 FGIP for 2-ethoxyethanol at 298K. S110
S51 FGIP for ethylene glycol at 298K. S111
S52 FGIP for 1,2-propanediol at 298K. S112
S53 FGIP for 1,3-propanediol at 298K. S113
S54 FGIP for 1,2-butanediol at 298K. S114
S55 FGIP for (2R,3S)-2,3-butanediol at 298K. S115

S56 FGIP for 1,4-butanediol at 298K. S116
S57 FGIP for 1,5-pentanediol at 298K. S117
S58 FGIP for diethylene glycol at 298K. S118
S59 FGIP for triethylene glycol at 298K. S119
S60 FGIP for glycerol at 298K. S120
S61 FGIP for phenol at 298K. S121
S62 FGIP for ortho-cresol at 298K. S122
S63 FGIP for meta-cresol at 298K. S123
S64 FGIP for para-cresol at 298K. S124
S65 FGIP for 2-methoxyphenol at 298K. S125
S66 FGIP for 2,4-dimethylphenol at 298K. S126
S67 FGIP for 3-chlorophenol at 298K. S127
S68 FGIP for diethyl ether at 298K. S128
S69 FGIP for di-n-propyl ether at 298K. S129
S70 FGIP for diisopropyl ether at 298K. S130
S71 FGIP for dibutyl ether at 298K. S131
S72 FGIP for bis(2-chloroethyl) ether at 298K. S132
S73 FGIP for 1,2-dimethyoxyethane at 298K. S133
S74 FGIP for diethylene glycol dimethyl ether at 298K. S134
S75 FGIP for furan at 298K. S135
S76 FGIP for tetrahydrofuran at 298K. S136
S77 FGIP for 2-methyltetrahydrofuran at 298K. S137
S78 FGIP for tetrahydropyran at 298K. S138
S79 FGIP for 1,3-dioxane at 298K. S139
S80 FGIP for 1,3-dioxolan at 298K. S140
S81 FGIP for 1,8-cineole at 298K. S141
S82 FGIP for anisole at 298K. S142

S83 FGIP for ethyl phenyl ether at 298K. S143
S84 FGIP for diphenyl ether at 298K. S144
S85 FGIP for dibenzyl ether at 298K. S145
S86 FGIP for 1,2-dimethoxybenzene at 298K. S146
S87 FGIP for methyl orthoformate at 298K. S147
S88 FGIP for methyl orthoacetate at 298K. S148
S89 FGIP for propionaldehyde at 298K. S149
S90 FGIP for butyraldehyde at 298K. S150
S91 FGIP for benzaldehyde at 298K. S151
S92 FGIP for p-methoxybenzaldehyde at 298K. S152
S93 FGIP for cinnamaldehyde at 298K. S153
S94 FGIP for acetone at 298K. S154
S95 FGIP for 2-butanone at 298K. S155
S96 FGIP for 2-pentanone at 298K. S156
S97 FGIP for 3-methyl-2-butanone at 298K. S157
S98 FGIP for 3-pentanone at 298K. S158
S99 FGIP for cyclopentanone at 298K. S159
S100 FGIP for 4-methyl-2-pentanone at 298K. S160
S101 FGIP for 3,3-dimethyl-2-butanone at 298K. S161
S102 FGIP for perfluorooctane at 298K. S162
S103 FGIP for cyclohexanone at 298K. S163
S104 FGIP for 2-heptanone at 298K. S164
S105 FGIP for 3-heptanone at 298K. S165
S106 FGIP for 2,2,4,4-tetramethyl-3-pentanone at 298K. S166
S107 FGIP for acetophenone at 298K. S167
S108 FGIP for ethyl phenyl ketone at 298K. S168
S109 FGIP for benzyl methyl ketone at 298K. S169

S110 FGIP for 2,4,5-trimethylacetophenone at 298K. S170
S111 FGIP for p-chloroacetophenone at 298K. S171
S112 FGIP for diphenyl ketone at 298K. S172
S113 FGIP for 2,4-pentanedione at 298K. S173
S114 FGIP for 2,3-butanedione at 298K. S174
S115 FGIP for formic acid at 298K. S175
S116 FGIP for acetic acid at 298K. S176
S117 FGIP for propanoic acid at 298K. S177
S118 FGIP for butanoic acid at 298K. S178
S119 FGIP for pentanoic acid at 298K. S179
S120 FGIP for hexanoic acid at 298K. S180
S121 FGIP for heptanoic acid at 298K. S181
S122 FGIP for dichloroacetic acid at 298K. S182
S123 FGIP for trifluoroacetic acid at 298K. S183
S124 FGIP for acetic anhydride at 298K. S184
S125 FGIP for benzoyl chloride at 298K. S185
S126 FGIP for benzoyl bromide at 298K. S186
S127 FGIP for methyl formate at 298K. S187
S128 FGIP for ethyl formate at 298K. S188
S129 FGIP for methyl acetate at 298K. S189
S130 FGIP for ethyl acetate at 298K. S190
S131 FGIP for n-propyl acetate at 298K. S191
S132 FGIP for butyl acetate at 298K. S192
S133 FGIP for isopentyl acetate at 298K. S193
S134 FGIP for methyl propionate at 298K. S194
S135 FGIP for ethyl propionate at 298K. S195
S136 FGIP for dimethyl carbonate at 298K. S196

S137 FGIP for diethyl carbonate at 298K. S197
S138 FGIP for ethylene carbonate at 298K. S198
S139 FGIP for 4-methyl-1,3-dioxolan-2-one at 298K. S199
S140 FGIP for diethyl malonate at 298K. S200
S141 FGIP for methyl benzoate at 298K. S201
S142 FGIP for ethyl benzoate at 298K. S202
S143 FGIP for dimethylphthalate at 298K. S203
S144 FGIP for di-n-butylorthophthalate at 298K. S204
S145 FGIP for ethyl chloroacetate at 298K. S205
S146 FGIP for ethyl trichloroacetate at 298K. S206
S147 FGIP for ethyl acetoacetate at 298K. S207
S148 FGIP for gamma-butyrolactone at 298K. S208
S149 FGIP for n-perfluorohexane at 298K. S209
S150 FGIP for perfluoromethylcyclohexane at 298K. S210
S151 FGIP for perfluoroheptane at 298K. S211
S152 FGIP for cis-perfluorodecalin at 298K. S212
S153 FGIP for fluorobenzene at 298K. S213
S154 FGIP for hexafluorobenzene at 298K. S214
S155 FGIP for 1,4-dichlorobutane at 298K. S215
S156 FGIP for chlorobenzene at 298K. S216
S157 FGIP for dichloromethane at 298K. S217
S158 FGIP for 1,1-dichloroethane at 298K. S218
S159 FGIP for 1,2-dichloroethane at 298K. S219
S160 FGIP for trans-1,2-dichloroethylene at 298K. S220
S161 FGIP for ortho-dichlorobenzene at 298K. S221
S162 FGIP for meta-dichlorobenzene at 298K. S222
S163 FGIP for chloroform at 298K. S223

S164 FGIP for 1,1,1-trichloroethane at 298K. S224
S165 FGIP for 1,1,2-trichloroethane at 298K. S225
S166 FGIP for trichloroethylene at 298K. S226
S167 FGIP for 1,2,4-trichlorobenzene at 298K. S227
S168 FGIP for carbon tetrachloride at 298K. S228
S169 FGIP for tetrachloroethylene at 298K. S229
S170 FGIP for 1,1,2,2-tetrachloroethane at 298K. S230
S171 FGIP for pentachloroethane at 298K. S231
S172 FGIP for 1-bromobutane at 298K. S232
S173 FGIP for bromobenzene at 298K. S233
S174 FGIP for dibromomethane at 298K. S234
S175 FGIP for 1,2-dibromoethane at 298K. S235
S176 FGIP for bromoform at 298K. S236
S177 FGIP for n-butyl iodide at 298K. S237
S178 FGIP for iodobenzene at 298K. S238
S179 FGIP for methylene iodide at 298K. S239
S180 FGIP for n-butylamine at 298K. S240
S181 FGIP for benzylamine at 298K. S241
S182 FGIP for ethylenediamine at 298K. S242
S183 FGIP for diethylamine at 298K. S243
S184 FGIP for di-n-butylamine at 298K. S244
S185 FGIP for pyrrole at 298K. S245
S186 FGIP for pyrrolidine at 298K. S246
S187 FGIP for piperidine at 298K. S247
S188 FGIP for morpholine at 298K. S248
S189 FGIP for triethylamine at 298K. S249
S190 FGIP for tri-(n-butyl)amine at 298K. S250

S191 FGIP for aniline at 298K. S251
S192 FGIP for o-chloroaniline at 298K. S252
S193 FGIP for methylphenylamine at 298K. S253
S194 FGIP for N,N-dimethylaniline at 298K. S254
S195 FGIP for aminoethanol at 298K. S255
S196 FGIP for diethanolamine at 298K. S256
S197 FGIP for triethanolamine at 298K. S257
S198 FGIP for pyridine at 298K. S258
S199 FGIP for 2-methylpyridine at 298K. S259
S200 FGIP for 3-methylpyridine at 298K. S260
S201 FGIP for 4-methylpyridine at 298K. S261
S202 FGIP for 2,4-dimethylpyridine at 298K. S262
S203 FGIP for 2,6-dimethylpyridine at 298K. S263
S204 FGIP for 2,4,6-trimethylpyridine at 298K. S264
S205 FGIP for 2-bromopyridine at 298K. S265
S206 FGIP for 3-bromopyridine at 298K. S266
S207 FGIP for 2-cyanopyridine at 298K. S267
S208 FGIP for pyrimidine at 298K. S268
S209 FGIP for quinoline at 298K. S269
S210 FGIP for acetonitrile at 298K. S270
S211 FGIP for propionitrile at 298K. S271
S212 FGIP for n-butyronitrile at 298K. S272
S213 FGIP for 3-methylbutanenitrile at 298K. S273
S214 FGIP for acrylonitrile at 298K. S274
S215 FGIP for phenylacetonitrile at 298K. S275
S216 FGIP for benzonitrile at 298K. S276
S217 FGIP for nitromethane at 298K. S277

S218 FGIP for nitroethane at 298K. S278

S219 FGIP for 1-nitropropane at 298K. S279

S220 FGIP for 2-nitropropane at 298K. S280

S221 FGIP for nitrobenzene at 298K. S281

S222 FGIP for formamide at 298K. S282

S223 FGIP for N-methylformamide at 298K. S283

S224 FGIP for N,N-dimethylformamide at 298K. S284

S225 FGIP for N,N-dimethylthioformamide at 298K. S285

S226 FGIP for N,N-diethylformamide at 298K. S286

S227 FGIP for N-methylacetamide at 298K. S287

S228 FGIP for N,N-dimethylacetamide at 298K. S288

S229 FGIP for N,N-diethylacetamide at 298K. S289

S230 FGIP for 2-pyrrolidinone at 298K. S290

S231 FGIP for N-methyl pyrrolidinone at 298K. S291

S232 FGIP for 1-methyl-2-pyrrolidinethione at 298K. S292

S233 FGIP for tetramethylurea at 298K. S293

S234 FGIP for tetraethylurea at 298K. S294

S235 FGIP for dimethylcyanamide at 298K. S295

S236 FGIP for carbon disulfide at 298K. S296

S237 FGIP for dimethyl sulfide at 298K. S297

S238 FGIP for diethyl sulfide at 298K. S298

S239 FGIP for diisopropyl sulfide at 298K. S299

S240 FGIP for dibutyl sulfide at 298K. S300

S241 FGIP for tetrahydrothiophene at 298K. S301

S242 FGIP for thiane at 298K. S302

S243 FGIP for dimethylsulfoxide at 298K. S303

S244 FGIP for dibutyl sulfoxide at 298K. S304

S245 FGIP for sulfolane at 298K. S305
S246 FGIP for thiobis(2-ethanol) at 298K. S306
S247 FGIP for diethyl sulfite at 298K. S307
S248 FGIP for dimethyl sulfate at 298K. S308
S249 FGIP for diethyl sulfate at 298K. S309
S250 FGIP for methanesulfonic acid at 298K. S310
S251 FGIP for trimethylphosphate at 298K. S311
S252 FGIP for triethylphosphate at 298K. S312
S253 FGIP for tri-n-butylphosphate at 298K. S313
S254 FGIP for hexamethylphosphoric triamide at 298K. S314
S255 FGIP for hydrogen peroxide at 298K. S315
S256 FGIP for hydrogen fluoride at 298K. S316
S257 FGIP for sulfuric acid at 298K. S317
S258 FGIP for ammonia at 298K. S318
S259 FGIP for hydrazine at 298K. S319
S260 FGIP for sulfur dioxide at 298K. S320
S261 FGIP for thionyl chloride at 298K. S321
S262 FGIP for phosphorus oxychloride at 298K. S322
S263 FGIP for dihydrolevoglucosenone at 298K. S323
S264 FGIP for 0.0% ethanol 100.0% water at 298K. S324
S265 FGIP for 5.0% ethanol 95.0% water at 298K. S325
S266 FGIP for 10.0% ethanol 90.0% water at 298K. S326
S267 FGIP for 15.0% ethanol 85.0% water at 298K. S327
S268 FGIP for 20.0% ethanol 80.0% water at 298K. S328
S269 FGIP for 25.0% ethanol 75.0% water at 298K. S329
S270 FGIP for 30.0% ethanol 70.0% water at 298K. S330
S271 FGIP for 35.0% ethanol 65.0% water at 298K. S331

S272 FGIP for 40.0% ethanol 60.0% water at 298K. S332
S273 FGIP for 45.0% ethanol 55.0% water at 298K. S333
S274 FGIP for 50.0% ethanol 50.0% water at 298K. S334
S275 FGIP for 55.0% ethanol 45.0% water at 298K. S335
S276 FGIP for 60.0% ethanol 40.0% water at 298K. S336
S277 FGIP for 65.0% ethanol 35.0% water at 298K. S337
S278 FGIP for 70.0% ethanol 30.0% water at 298K. S338
S279 FGIP for 75.0% ethanol 25.0% water at 298K. S339
S280 FGIP for 80.0% ethanol 20.0% water at 298K. S340
S281 FGIP for 85.0% ethanol 15.0% water at 298K. S341
S282 FGIP for 90.0% ethanol 10.0% water at 298K. S342
S283 FGIP for 95.0% ethanol 5.0% water at 298K. S343
S284 FGIP for 100.0% ethanol 0.0% water at 298K. S344
S285 FGIP for 0.0% chloroform 100.0% tetrahydrofuran at 298K. S345
S286 FGIP for 5.0% chloroform 95.0% tetrahydrofuran at 298K. S346
S287 FGIP for 10.0% chloroform 90.0% tetrahydrofuran at 298K. S347
S288 FGIP for 15.0% chloroform 85.0% tetrahydrofuran at 298K. S348
S289 FGIP for 20.0% chloroform 80.0% tetrahydrofuran at 298K. S349
S290 FGIP for 25.0% chloroform 75.0% tetrahydrofuran at 298K. S350
S291 FGIP for 30.0% chloroform 70.0% tetrahydrofuran at 298K. S351
S292 FGIP for 35.0% chloroform 65.0% tetrahydrofuran at 298K. S352
S293 FGIP for 40.0% chloroform 60.0% tetrahydrofuran at 298K. S353
S294 FGIP for 45.0% chloroform 55.0% tetrahydrofuran at 298K. S354
S295 FGIP for 50.0% chloroform 50.0% tetrahydrofuran at 298K. S355
S296 FGIP for 55.0% chloroform 45.0% tetrahydrofuran at 298K. S356
S297 FGIP for 60.0% chloroform 40.0% tetrahydrofuran at 298K. S357
S298 FGIP for 65.0% chloroform 35.0% tetrahydrofuran at 298K. S358

S299 FGIP for 70.0% chloroform 30.0% tetrahydrofuran at 298K. S359

S300 FGIP for 75.0% chloroform 25.0% tetrahydrofuran at 298K. S360

S301 FGIP for 80.0% chloroform 20.0% tetrahydrofuran at 298K. S361

S302 FGIP for 85.0% chloroform 15.0% tetrahydrofuran at 298K. S362

S303 FGIP for 90.0% chloroform 10.0% tetrahydrofuran at 298K. S363

S304 FGIP for 95.0% chloroform 5.0% tetrahydrofuran at 298K. S364

S305 FGIP for 100.0% chloroform 0.0% tetrahydrofuran at 298K. S365

Automated UV-Vis Titrations

All solvents were HPLC grade and were used without further purification. 4-phenyl azophenol and tri-n-butylphosphine oxide were purchased from Aldrich and used without further purification. Tri-n-butylphosphine oxide was dried in a vacuum desiccator over phosphorous pentaoxide before use. Association constants were determined using a BMG Labtech Fluorostar Optima plate reader with a Hellma quartz 96-well microplate. In a typical experiment, the micro-plate contained two titrations; the first in S1 in wells 1-48 (titration 1), the second in S2 in wells 49-96 (titration 2). Host stock solutions were prepared from accurately weighed samples of 4-phenyl azophenol (5 mg) dissolved in chloroform in 25 mL volumetric flasks, to give a concentration of 1 mM. Five guest stock solutions were prepared by dissolving an accurately weighed sample of tri-n-butylphosphine oxide (4.36 g) in chloroform in a 10 mL volumetric flask to give a 2 M stock solution of guest (stock solution 1). A serial dilution was carried out, whereby 313 μL of stock solution 1 was transferred to a 10 mL volumetric flask, which was then filled with chloroform to give stock solution 2 (6x10^{-2} M). Each new stock solution was diluted in the same way to give a further three stock solutions, with concentrations of 2x10^{-3} M, 6x10^{-5} M and 2x10^{-6} M. These solutions were loaded onto the 96-well quartz micro-plate using purpose written protocols with the UV-Vis plate reader. The solvent was allowed to evaporate, and then the micro-plate was

dried in a vacuum desiccator over phosphorous pentaoxide to remove residual chloroform. The micro-plate now contained dried samples of host and guest in each well. The filling procedure was designed such that the total volume in each well did not exceed 90 μL, to facilitate dissolution on addition of pure S1 or S2 (150 μL) in the solvent titration.

Preparation of the micro-plate was followed by automated solvent addition. The dry plate was loaded into the plate reader. Then 150 μL of S1 was added to well 1 of titration 1. After addition of S1 the plate was agitated (20 s, double orbital shaking at 600 rpm) to ensure dissolution, and then the instrument recorded the absorbance in the well. The instrument read the absorbance at 6 fixed wavelengths; 260 nm, 280 nm, 340 nm, 390 nm, 420 nm and 600 nm. This was followed by the addition of a 10 μL aliquot of S2 into the same well, followed by agitation (5 s, double orbital shaking at 600 rpm) and an absorbance measurement. The addition of 10 μL aliquots of S2 into well 1 of titration 1 was repeated until the total volume in the well reached 320 μL, which gave a total of 17 additions. This titration of S1 and S2 was carried out, well-by-well, for each well of titration 1, and the procedure takes approximately six minutes for each well. After this, the solvent addition was carried out for all the wells in titration 2. 150 μL of S2 was added to well 49, which is the first well of titration 2. After addition of S2 the plate was agitated to ensure dissolution, and then the instrument recorded the absorbance in the well. This was followed by the addition of a 10 μL aliquot of S1 into the same well, followed by agitation and an absorbance measurement. The addition of 10 μL aliquots of S1 into well 49 was repeated until the total volume in the well reached 320 μL. This titration of S1 and S2 was carried out, well-by-well, for each well of titration 2. The automated solvent addition was completed in around 12 hours.

Collation of the data gave one binding isotherm for each solvent mixture. The binding isotherms fit well to a 1:1 binding isotherm, and an example of a typical binding isotherm is shown in Figure S1. The observed changes in absorbance were analysed using purpose-written software on an Apple Macintosh microcomputer. The software generates a binding

Figure S1: A typical binding isotherm obtained from an automated UV-Vis absorption titration of tri-n-butylphosphine oxide into 4-phenyl azophenol in 6% chloroform 94% tetrahydrofuran. The line shows the best fit of the experimental data to Equation (S2).

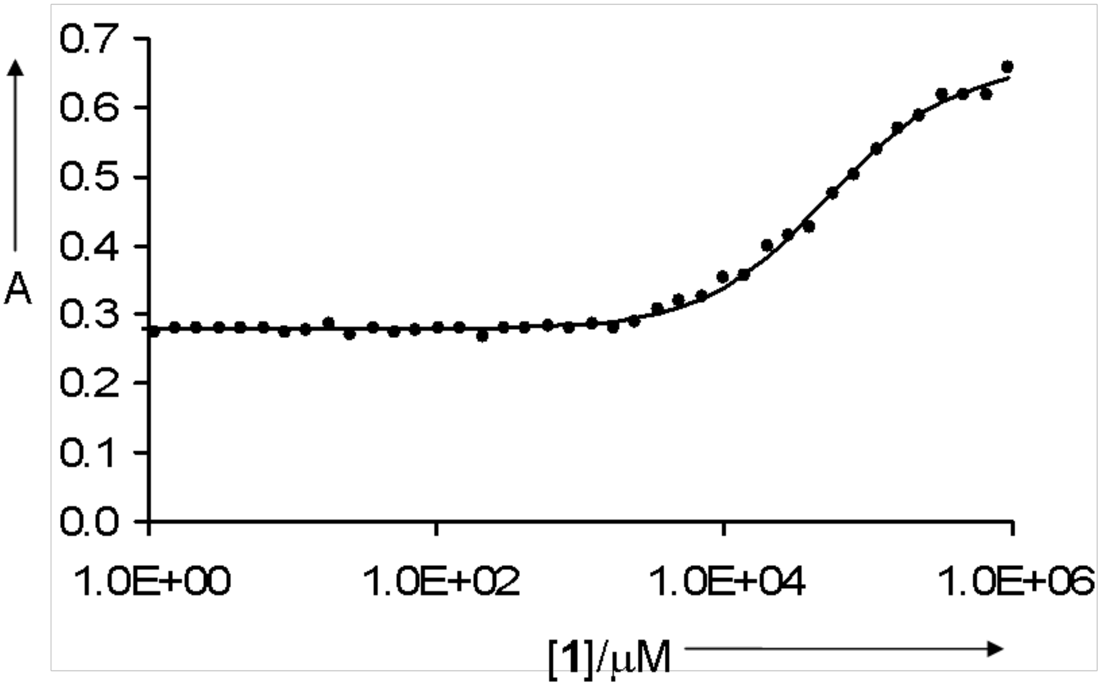

constant for each solvent composition by an iterative procedure which changes the binding constant (K), the absorbance of the free host (A_{free}), the absorbance of the bound host (A_{bound}) and the absorbance of the free guest (A_G) to give a calculated absorbance (A_{calc}) that matches the experimental absorbance (A_{expt}).

$$A_{calc} = A_G[G] + A_{free}[H_{free}] + A_{bound}[H_{bound}] \tag{S1}$$

The first term in equation (S1) is a linear correction to account for the absorbance of guest at high concentrations. The concentration of host bound to guest ($[H_{bound}]$) is given by equation (S2).

$$[H_{bound}] = \frac{1 + K([G]+[H]) + \sqrt{(1 + K([G]+[H]))^2 - 4K^2[G][H]}}{2K} \tag{S2}$$

Dilution of the solutes during the solvent titration is taken into account in Equation (S2).

A_{expt} is not affected by this dilution, because there is a corresponding increase in the path length as the well is filled. The data is fitted by an iterative procedure which minimises the sum of the residuals between A_{calc} and the experimental absorbance (A_{expt}) for every solvent composition in every well of the plate. This procedure includes all of the experimental data. To eliminate outliers, a user defined error threshold is implemented. For the first fitting, the error threshold is set to be large (10), so all data points are included in the fitting. After the first fitting, the error threshold is reduced, and any point for which the absolute difference between A_{expt} and A_{calc} is greater than the error threshold is replaced by A_{calc}, then the fitting procedure is repeated. This process is carried out until the error threshold is 0.1.

Pure Solvent Information

Solvent	ID	[liquid]/ M	InChIKey	N_{SSIP}	ΔG_c/ kJ mol^{-1}
tetramethylsilane	1	7.33	CZDYPVPMEAXLPK-UHFFFAOYSA-N	16	1.03
n-pentane	2	8.61	OFBQJSOFQDEBGM-UHFFFAOYSA-N	14	1.05
2-methylbutane	3	8.51	QWTDNUCVQCZILF-UHFFFAOYSA-N	14	1.04
n-hexane	4	7.60	VLKZOEOYAKHREP-UHFFFAOYSA-N	16	1.05
cyclohexane	5	9.20	XDTMQSROBMDMFD-UHFFFAOYSA-N	14	1.09
n-heptane	6	6.78	IMNFDUFMRHMDMM-UHFFFAOYSA-N	18	1.06
n-octane	7	6.12	TVMXDCGIABBOFY-UHFFFAOYSA-N	21	1.09
2,2,4-trimethylpentane	8	6.24	NHTMVDHEPJAVLT-UHFFFAOYSA-N	19	1.04
n-decane	9	5.11	DIOQZVSQGTUSAI-UHFFFAOYSA-N	25	1.08
n-dodecane	10	4.43	SNRUBQQJIBEYMU-UHFFFAOYSA-N	29	1.09
n-hexadecane	11	3.40	DCAYPVUWAIABOU-UHFFFAOYSA-N	37	1.08

Continued on next page

Solvent	ID	[liquid]/ M	InChIKey	N_{SSIP}	ΔG_c/ kJ mol^{-1}
benzene	12	11.12	UHOVQNZJYSORNB-UHFFFAOYSA-N	12	1.11
toluene	13	9.35	YXFVVABEGXRONW-UHFFFAOYSA-N	14	1.10
ortho-xylene	14	8.26	CTQNGGLPUBDAKN-UHFFFAOYSA-N	16	1.11
meta-xylene	15	8.11	IVSZLXZYQVIEFR-UHFFFAOYSA-N	16	1.10
para-xylene	16	8.08	URLKBWYHVLBVBO-UHFFFAOYSA-N	16	1.09
ethylbenzene	17	8.13	YNQLUTRBYVCPMQ-UHFFFAOYSA-N	16	1.10
isopropylbenzene	18	7.14	RWGFKTVRMDUZSP-UHFFFAOYSA-N	18	1.09
1,3,5-trimethylbenzene	19	7.17	AUHZEENZYGFFBQ-UHFFFAOYSA-N	18	1.09
styrene	20	8.66	PPBRXRYQALVLMV-UHFFFAOYSA-N	15	1.10
1,2,3,4-tetrahydronaphthalene	21	7.30	CXWXQJXEFPUFDZ-UHFFFAOYSA-N	18	1.10
cis-decalin	22	6.46	NNBZCPXTIHJBJL-AOOOYVTPSA-N	19	1.06
water	23	55.35	XLYOFNOQVPJJNP-UHFFFAOYSA-N	4	1.48

Continued on next page

Solvent	ID	[liquid]/ M	InChIKey	N_{SSIP}	ΔG_c/ kJ mol^{-1}
methanol	24	24.57	OKKJLVBELUTLKV-UHFFFAOYSA-N	7	1.29
ethanol	25	17.03	LFQSCWFLJHTTHZ-UHFFFAOYSA-N	9	1.21
1-propanol	26	13.32	BDERNNFJNOPAEC-UHFFFAOYSA-N	11	1.18
2-propanol	27	13.00	KFZMGEQAYNKOFK-UHFFFAOYSA-N	11	1.16
1-butanol	28	10.87	LRHPLDYGYMQRHN-UHFFFAOYSA-N	13	1.15
2-methyl-1-propanol	29	10.76	ZXEKIIBDNHEJCQ-UHFFFAOYSA-N	13	1.15
2-butanol	30	10.83	BTANRVKWQNVYAZ-BYPYZUCNSA-N	13	1.15
2-methyl-2-propanol	31	10.54	DKGAVHZHDRPRBM-UHFFFAOYSA-N	13	1.13
1-pentanol	32	9.22	AMQJEAYHLZJPGS-UHFFFAOYSA-N	15	1.14
3-methyl-1-butanol	33	9.16	PHTQWCKDNZKARW-UHFFFAOYSA-N	15	1.13
2-methyl-2-butanol	34	9.13	MSXVEPNJUHWQHW-UHFFFAOYSA-N	14	1.09
1-hexanol	35	7.99	ZSIAUFGUXNUGDI-UHFFFAOYSA-N	17	1.13

Continued on next page

Solvent	ID	[liquid]/ M	InChIKey	N_{SSIP}	ΔG_c/ kJ mol^{-1}
cyclohexanol	36	9.68	HPXRVTGHNJAIIH-UHFFFAOYSA-N	15	1.17
1-octanol	37	6.31	KBPLFHHGFOOTCA-UHFFFAOYSA-N	21	1.11
1-decanol	38	5.24	MWKFXSUHUHTGQN-UHFFFAOYSA-N	26	1.13
1-dodecanol	39	4.46	LQZZUXJYWNFBMV-UHFFFAOYSA-N	30	1.12
benzyl alcohol	40	9.63	WVDDGKGOMKODPV-UHFFFAOYSA-N	15	1.17
2-phenylethanol	41	8.38	WRMNZCZEMHIOCP-UHFFFAOYSA-N	17	1.16
allyl alcohol	42	14.58	XXROGKLTLUQVRX-UHFFFAOYSA-N	10	1.17
2-chloroethanol	43	15.48	SZIFAVKTNFCBPC-UHFFFAOYSA-N	11	1.28
2-cyanoethanol	44	14.64	WSGYTJNNHPZFKR-UHFFFAOYSA-N	11	1.24
2,2,2-trifluoroethanol	45	13.82	RHQDFWAXVIIEBN-UHFFFAOYSA-N	10	1.14
1,1,1,3,3,3-hexafluoro-2-propanol	46	9.56	BYEAHWXPCBROCE-UHFFFAOYSA-N	13	1.07
2-methoxyethanol	47	12.61	XNWFRZJHXBZDAG-UHFFFAOYSA-N	12	1.20

Continued on next page

Solvent	ID	[liquid]/ M	InChIKey	N_{SSIP}	ΔG_c/ kJ mol^{-1}
2-ethoxyethanol	48	10.27	ZNQVEEAIQZEUHB-UHFFFAOYSA-N	14	1.16
ethylene glycol	49	17.89	LYCAIKOWRPUZTN-UHFFFAOYSA-N	10	1.32
1,2-propanediol	50	13.57	DNIAPMSPPWPWGF-GSVOUGTGSA-N	12	1.25
1,3-propanediol	51	13.79	YPFDHNVEDLHUCE-UHFFFAOYSA-N	12	1.26
1,2-butanediol	52	11.09	BMRWNKZVCUKKSR-SCSAIBSYSA-N	14	1.22
(2R,3S)-2,3-butanediol	53	10.95	OWBTYPJTUOEWEK-ZXZARUISSA-N	14	1.21
1,4-butanediol	54	11.24	WERYXYBDKMZEQL-UHFFFAOYSA-N	14	1.23
1,5-pentanediol	55	9.47	ALQSHHUCVQOPAS-UHFFFAOYSA-N	16	1.20
diethylene glycol	56	10.52	MTHSVFCYNBDYFN-UHFFFAOYSA-N	15	1.23
triethylene glycol	57	7.46	ZIBGPFATKBEMQZ-UHFFFAOYSA-N	21	1.22
glycerol	58	13.66	PEDCQBHIVMGVHV-UHFFFAOYSA-N	13	1.31
phenol	59	11.39	ISWSIDIOOBJBQZ-UHFFFAOYSA-N	13	1.18

Continued on next page

Solvent	ID	[liquid]/ M	InChIKey	N_{SSIP}	ΔG_c/ kJ mol^{-1}
ortho-cresol	60	9.62	QWVGKYWNOKOFNN-UHFFFAOYSA-N	15	1.17
meta-cresol	61	9.53	RLSSMJSEOOYNOY-UHFFFAOYSA-N	15	1.16
para-cresol	62	9.44	IWDCLRJOBJJRNH-UHFFFAOYSA-N	15	1.15
2-methoxyphenol	63	9.09	LHGVFZTZFXWLCP-UHFFFAOYSA-N	16	1.17
2,4-dimethylphenol	64	8.32	KUFFULVDNCHOFZ-UHFFFAOYSA-N	17	1.15
3-chlorophenol	65	9.87	HORNXRXVQWOLPJ-UHFFFAOYSA-N	15	1.18
diethyl ether	66	9.55	RTZKZFJDLAIYFH-UHFFFAOYSA-N	13	1.07
di-n-propyl ether	67	7.27	POLCUAVZOMRGSN-UHFFFAOYSA-N	18	1.10
diisopropyl ether	68	7.03	ZAFNJMIOTHYJRJ-UHFFFAOYSA-N	17	1.04
dibutyl ether	69	5.87	DURPTKYDGMDSBL-UHFFFAOYSA-N	22	1.09
bis(2-chloroethyl) ether	70	8.48	ZNSMNVMLTJELDZ-UHFFFAOYSA-N	17	1.17
1,2-dimethyoxyethane	71	9.57	XTHFKEDIFFGKHM-UHFFFAOYSA-N	14	1.12

Continued on next page

Solvent	ID	[liquid]/ M	InChIKey	N_{SSIP}	ΔG_c/ kJ mol^{-1}
diethylene glycol dimethyl ether	72	7.00	SBZXBUIDTXKZTM-UHFFFAOYSA-N	20	1.15
furan	73	13.68	YLQBMQCUIZJEEH-UHFFFAOYSA-N	10	1.13
tetrahydrofuran	74	12.25	WYURNTSHIVDZCO-UHFFFAOYSA-N	11	1.12
2-methyltetrahydrofuran	75	9.91	JWUJQDFVADABEY-RXMQYKEDSA-N	13	1.09
tetrahydropyran	76	10.18	DHXVGJBLRPWPCS-UHFFFAOYSA-N	13	1.11
1,3-dioxane	77	11.67	VDFVNEFVBPFDSB-UHFFFAOYSA-N	12	1.15
1,3-dioxolan	78	14.37	WNXJIVFYUVYPPR-UHFFFAOYSA-N	11	1.23
1,8-cineole	79	5.96	WEEGYLXZBRQIMU-WAAGHKOSSA-N	20	1.04
anisole	80	9.15	RDOXTESZEPMUJZ-UHFFFAOYSA-N	15	1.13
ethyl phenyl ether	81	7.87	DLRJIFUOBPOJNS-UHFFFAOYSA-N	17	1.12
diphenyl ether	82	6.29	USIUVYZYUHIAEV-UHFFFAOYSA-N	21	1.11
dibenzyl ether	83	5.19	MHDVGSVTJDSBDK-UHFFFAOYSA-N	26	1.12

Continued on next page

Solvent	ID	[liquid]/ M	InChIKey	N_{SSIP}	ΔG_c/ kJ mol^{-1}
1,2-dimethoxybenzene	84	7.83	ABDKAPXRBAPSQN-UHFFFAOYSA-N	18	1.15
methyl orthoformate	85	8.89	PYOKUURKVVELLB-UHFFFAOYSA-N	15	1.11
methyl orthoacetate	86	7.37	HDPNBNXLBDFELL-UHFFFAOYSA-N	16	1.03
propionaldehyde	87	13.62	NBBJYMSMWIIQGU-UHFFFAOYSA-N	10	1.13
butyraldehyde	88	11.05	ZTQSAGDEMFDKMZ-UHFFFAOYSA-N	12	1.11
benzaldehyde	89	9.84	HUMNYLRZRPPJDN-UHFFFAOYSA-N	14	1.13
p-methoxybenzaldehyde	90	8.23	ZRSNZINYAWTAHE-UHFFFAOYSA-N	17	1.14
cinnamaldehyde	91	7.95	KJPRLNWUNMBNBZ-QPJJXVBHSA-N	18	1.16
acetone	92	13.51	CSCPPACGZOOCGX-UHFFFAOYSA-N	10	1.12
2-butanone	93	10.93	ZWEHNKRNPOVVGH-UHFFFAOYSA-N	12	1.10
2-pentanone	94	9.30	XNLICIUVMPYHGG-UHFFFAOYSA-N	14	1.10
3-methyl-2-butanone	95	9.35	SYBYTAAJFKOIEJ-UHFFFAOYSA-N	14	1.10

Continued on next page

Solvent	ID	[liquid]/ M	InChIKey	N_{SSIP}	ΔG_c/ kJ mol^{-1}
3-pentanone	96	9.40	FDPIMTJIUBPUKL-UHFFFAOYSA-N	14	1.10
cyclopentanone	97	11.24	BGTOWKSIORTVQH-UHFFFAOYSA-N	13	1.17
4-methyl-2-pentanone	98	7.96	NTIZESTWPVYFNL-UHFFFAOYSA-N	16	1.08
3,3-dimethyl-2-butanone	99	8.00	PJGSXYOJTGTZAV-UHFFFAOYSA-N	15	1.05
perfluorooctane	100	4.03	YVBBRRALBYAZBM-UHFFFAOYSA-N	27	0.98
cyclohexanone	101	9.60	JHIVVAPYMSGYDF-UHFFFAOYSA-N	14	1.12
2-heptanone	102	7.11	CATSNJVOTSVZJV-UHFFFAOYSA-N	19	1.12
3-heptanone	103	7.11	NGAZZOYFWWSOGK-UHFFFAOYSA-N	19	1.12
2,2,4,4-tetramethyl-3-pentanone	104	5.77	UIQGEWJEWJMQSL-UHFFFAOYSA-N	20	1.02
acetophenone	105	8.52	KWOLFJPFCHCOCG-UHFFFAOYSA-N	16	1.13
ethyl phenyl ketone	106	7.53	KRIOVPPHQSLHCZ-UHFFFAOYSA-N	18	1.12
benzyl methyl ketone	107	7.57	QCCDLTOVEPVEJK-UHFFFAOYSA-N	18	1.13

Continued on next page

Solvent	ID	[liquid]/ M	InChIKey	N_{SSIP}	ΔG_c/ kJ mol^{-1}
2,4,5-trimethylacetophenone	108	7.50	GENBEGZNCBFHSU-UHFFFAOYSA-N	22	1.26
p-chloroacetophenone	109	7.71	BUZYGTVTZYSBCU-UHFFFAOYSA-N	18	1.14
diphenyl ketone	110	6.08	RWCCWEUUXYIKHB-UHFFFAOYSA-N	22	1.12
2,4-pentanedione	111	9.71	YRKCREAYFQTBPV-UHFFFAOYSA-N	14	1.13
2,3-butanedione	112	11.39	QSJXEFYPDANLFS-UHFFFAOYSA-N	13	1.18
formic acid	113	26.38	BDAGIHXWWSANSR-UHFFFAOYSA-N	7	1.34
acetic acid	114	17.39	QTBSBXVTEAMEQO-UHFFFAOYSA-N	9	1.22
propanoic acid	115	13.33	XBDQKXXYIPTUBI-UHFFFAOYSA-N	11	1.18
butanoic acid	116	10.82	FERIUCNNQQJTOY-UHFFFAOYSA-N	13	1.15
pentanoic acid	117	9.15	NQPDZGIKBAWPEJ-UHFFFAOYSA-N	15	1.13
hexanoic acid	118	7.95	FUZZWVXGSFPDMH-UHFFFAOYSA-N	17	1.12
heptanoic acid	119	7.06	MNWFXJYAOYHMED-UHFFFAOYSA-N	20	1.15

Continued on next page

Solvent	ID	[liquid]/ M	InChIKey	N_{SSIP}	ΔG_c/ kJ mol^{-1}
dichloroacetic acid	120	12.12	JXTHNDFMNIQAHM-UHFFFAOYSA-N	13	1.23
trifluoroacetic acid	121	12.97	DTQVDTLACAAQTR-UHFFFAOYSA-N	11	1.16
acetic anhydride	122	10.54	WFDIJRYMOXRFFG-UHFFFAOYSA-N	14	1.18
benzoyl chloride	123	8.62	PASDCCFISLVPSO-UHFFFAOYSA-N	16	1.14
benzoyl bromide	124	8.48	AQIHMSVIAGNIDM-UHFFFAOYSA-N	16	1.12
methyl formate	125	16.10	TZIHFWKZFHZASV-UHFFFAOYSA-N	9	1.17
ethyl formate	126	12.36	WBJINCZRORDGAQ-UHFFFAOYSA-N	11	1.13
methyl acetate	127	12.53	KXKVLQRXCPHEJC-UHFFFAOYSA-N	11	1.14
ethyl acetate	128	10.15	XEKOWRVHYACXOJ-UHFFFAOYSA-N	13	1.11
n-propyl acetate	129	8.65	YKYONYBAUNKHLG-UHFFFAOYSA-N	16	1.14
butyl acetate	130	7.55	DKPFZGUDAPQIHT-UHFFFAOYSA-N	18	1.13
isopentyl acetate	131	6.66	MLFHJEHSLIIPHL-UHFFFAOYSA-N	19	1.08

Continued on next page

Solvent	ID	[liquid]/ M	InChIKey	N_{SSIP}	ΔG_c/ kJ mol^{-1}
methyl propionate	132	10.31	RJUFJBKOKNCXHH-UHFFFAOYSA-N	13	1.12
ethyl propionate	133	8.66	FKRCODPIKNYEAC-UHFFFAOYSA-N	16	1.14
dimethyl carbonate	134	11.88	IEJIGPNLZYLLBP-UHFFFAOYSA-N	12	1.16
diethyl carbonate	135	8.21	OIFBSDVPJOWBCH-UHFFFAOYSA-N	17	1.14
ethylene carbonate	136	15.12	KMTRUDSVKNLOMY-UHFFFAOYSA-N	11	1.26
4-methyl-1,3-dioxolan-2-one	137	11.75	RUOJZAUFBMNUDX-GSVOUGTGSA-N	13	1.20
diethyl malonate	138	6.56	IYXGSMUGOJNHAZ-UHFFFAOYSA-N	21	1.13
methyl benzoate	139	7.96	QPJVMBTYPHYUOC-UHFFFAOYSA-N	17	1.12
ethyl benzoate	140	6.94	MTZQAGJQAFMTAQ-UHFFFAOYSA-N	19	1.11
dimethylphthalate	141	6.13	NIQCNGHVCWTJSM-UHFFFAOYSA-N	22	1.12
di-n-butylorthophthalate	142	3.75	DOIRQSBPFJWKBE-UHFFFAOYSA-N	36	1.12
ethyl chloroacetate	143	10.26	VEUUMBGHMNQHGO-UHFFFAOYSA-N	15	1.21

Continued on next page

Solvent	ID	[liquid]/ M	InChIKey	N_{SSIP}	ΔG_c/ kJ mol^{-1}
ethyl trichloroacetate	144	7.23	SJMLNDPIJZBEKY-UHFFFAOYSA-N	19	1.13
ethyl acetoacetate	145	7.85	XYIBRDXRRQCHLP-UHFFFAOYSA-N	18	1.15
gamma-butyrolactone	146	13.07	YEJRWHAVMIAJKC-UHFFFAOYSA-N	12	1.22
n-perfluorohexane	147	4.97	ZJIJAJXFLBMLCK-UHFFFAOYSA-N	22	0.99
perfluoromethylcyclohexane	148	4.45	QIROQPWSJUXOJC-UHFFFAOYSA-N	21	0.89
perfluoroheptane	149	4.51	LGUZHRODIJCVOC-UHFFFAOYSA-N	24	0.98
cis-perfluorodecalin	150	4.21	UWEYRJFJVCLAGH-XIXRPRMCSA-N	25	0.96
fluorobenzene	151	10.60	PYLWMHQQBFSUBP-UHFFFAOYSA-N	12	1.08
hexafluorobenzene	152	8.67	ZQBFAOFFOQMSGJ-UHFFFAOYSA-N	15	1.10
1,4-dichlorobutane	153	9.51	KJDRSWPQXHESDQ-UHFFFAOYSA-N	16	1.20
chlorobenzene	154	9.79	MVPPADPHJFYWMZ-UHFFFAOYSA-N	14	1.13
dichloromethane	155	15.50	YMWUJEATGCHHMB-UHFFFAOYSA-N	10	1.22

Continued on next page

Solvent	ID	[liquid]/ M	InChIKey	N_{SSIP}	ΔG_c/ kJ mol^{-1}
1,1-dichloroethane	156	11.81	SCYULBFZEHDVBN-UHFFFAOYSA-N	12	1.15
1,2-dichloroethane	157	12.59	WSLDOOZREJYCGB-UHFFFAOYSA-N	12	1.20
trans-1,2-dichloroethylene	158	12.85	KFUSEUYYWQURPO-OWOJBTEDSA-N	11	1.15
ortho-dichlorobenzene	159	8.84	RFFLAFLAYFXFSW-UHFFFAOYSA-N	15	1.11
meta-dichlorobenzene	160	8.73	ZPQOPVIELGIULI-UHFFFAOYSA-N	16	1.14
chloroform	161	12.40	HEDRZPFGACZZDS-UHFFFAOYSA-N	11	1.13
1,1,1-trichloroethane	162	9.97	UOCLXMDMGBRAIB-UHFFFAOYSA-N	13	1.09
1,1,2-trichloroethane	163	10.73	UBOXGVDOUJQMTN-UHFFFAOYSA-N	13	1.14
trichloroethylene	164	11.12	XSTXAVWGXDQKEL-UHFFFAOYSA-N	13	1.17
1,2,4-trichlorobenzene	165	8.02	PBKONEOXTCPAFI-UHFFFAOYSA-N	17	1.13
carbon tetrachloride	166	10.30	VZGDMQKNWNREIO-UHFFFAOYSA-N	13	1.12
tetrachloroethylene	167	9.74	CYTYCFOTNPOANT-UHFFFAOYSA-N	14	1.13

Continued on next page

Solvent	ID	[liquid]/ M	InChIKey	N_{SSIP}	ΔG_c/ kJ mol^{-1}
1,1,2,2-tetrachloroethane	168	9.45	QPFMBZIOSGYJDE-UHFFFAOYSA-N	15	1.15
pentachloroethane	169	8.27	BNIXVQGCZULYKV-UHFFFAOYSA-N	16	1.11
1-bromobutane	170	9.26	MPPPKRYCTPRNTB-UHFFFAOYSA-N	15	1.14
bromobenzene	171	9.48	QARVLSVVCXYDNA-UHFFFAOYSA-N	14	1.11
dibromomethane	172	14.33	FJBFPHVGVWTDIP-UHFFFAOYSA-N	11	1.23
1,2-dibromoethane	173	11.55	PAAZPARNPHGIKF-UHFFFAOYSA-N	13	1.19
bromoform	174	11.39	DIKBFYAXUHHXCS-UHFFFAOYSA-N	13	1.18
n-butyl iodide	175	8.73	KMGBZBJJOKUPIA-UHFFFAOYSA-N	16	1.14
iodobenzene	176	8.94	SNHMUERNLJLMHN-UHFFFAOYSA-N	15	1.12
methylene iodide	177	12.41	NZZFYRREKKOMAT-UHFFFAOYSA-N	13	1.24
n-butylamine	178	10.07	HQABUPZFAYXKJW-UHFFFAOYSA-N	14	1.15
benzylamine	179	9.16	WGQKYBSKWIADBV-UHFFFAOYSA-N	15	1.13

Continued on next page

Solvent	ID	[liquid]/ M	InChIKey	N_{SSIP}	ΔG_c/ kJ mol^{-1}
ethylenediamine	180	14.79	PIICEJLVQHRZGT-UHFFFAOYSA-N	11	1.25
diethylamine	181	9.60	HPNMFZURTQLUMO-UHFFFAOYSA-N	14	1.12
di-n-butylamine	182	5.86	JQVDAXLFBXTEQA-UHFFFAOYSA-N	22	1.09
pyrrole	183	14.39	KAESVJOAVNADME-UHFFFAOYSA-N	10	1.16
pyrrolidine	184	12.01	RWRDLPDLKQPQOW-UHFFFAOYSA-N	12	1.16
piperidine	185	10.06	NQRYJNQNLNOLGT-UHFFFAOYSA-N	13	1.10
morpholine	186	11.43	YNAVUWVOSKDBBP-UHFFFAOYSA-N	13	1.19
triethylamine	187	7.15	ZMANZCXQSJIPKH-UHFFFAOYSA-N	17	1.05
tri-(n-butyl)amine	188	4.18	IMFACGCPASFAPR-UHFFFAOYSA-N	29	1.05
aniline	189	10.93	PAYRUJLWNCNPSJ-UHFFFAOYSA-N	13	1.16
o-chloroaniline	190	9.47	AKCRQHGQIJBRMN-UHFFFAOYSA-N	15	1.16
methylphenylamine	191	9.17	AFBPFSWMIHJQDM-UHFFFAOYSA-N	15	1.13

Continued on next page

Solvent	ID	[liquid]/ M	InChIKey	N_{SSIP}	ΔG_c/ kJ mol^{-1}
N,N-dimethylaniline	192	7.87	JLTDJTHDQAWBAV-UHFFFAOYSA-N	17	1.12
aminoethanol	193	16.58	HZAXFHJVJLSVMW-UHFFFAOYSA-N	10	1.26
diethanolamine	194	10.42	ZBCBWPMODOFKDW-UHFFFAOYSA-N	15	1.22
triethanolamine	195	7.51	GSEJCLTVZPLZKY-UHFFFAOYSA-N	20	1.19
pyridine	196	12.36	JUJWROOIHBZHMG-UHFFFAOYSA-N	12	1.18
2-methylpyridine	197	10.09	BSKHPKMHTQYZBB-UHFFFAOYSA-N	14	1.15
3-methylpyridine	198	10.24	ITQTTZVARXURQS-UHFFFAOYSA-N	14	1.16
4-methylpyridine	199	10.20	FKNQCJSGGFJEIZ-UHFFFAOYSA-N	14	1.16
2,4-dimethylpyridine	200	8.66	JYYNAJVZFGKDEQ-UHFFFAOYSA-N	16	1.14
2,6-dimethylpyridine	201	8.57	OISVCGZHLKNMSJ-UHFFFAOYSA-N	16	1.13
2,4,6-trimethylpyridine	202	7.52	BWZVCCNYKMEVEX-UHFFFAOYSA-N	18	1.12
2-bromopyridine	203	10.48	IMRWILPUOVGIMU-UHFFFAOYSA-N	14	1.18

Continued on next page

Solvent	ID	[liquid]/ M	InChIKey	N_{SSIP}	ΔG_c/ kJ mol^{-1}
3-bromopyridine	204	10.38	NYPYPOZNGOXYSU-UHFFFAOYSA-N	14	1.17
2-cyanopyridine	205	10.39	FFNVQNRYTPFDDP-UHFFFAOYSA-N	14	1.17
pyrimidine	206	12.69	CZPWVGJYEJSRLH-UHFFFAOYSA-N	11	1.14
quinoline	207	8.44	SMWDFEZZVXVKRB-UHFFFAOYSA-N	16	1.12
acetonitrile	208	18.90	WEVYAHXRMPXWCK-UHFFFAOYSA-N	8	1.20
propionitrile	209	14.10	FVSKHRXBFJPNKK-UHFFFAOYSA-N	10	1.15
n-butyronitrile	210	11.38	KVNRLNFWIYMESJ-UHFFFAOYSA-N	12	1.13
3-methylbutanenitrile	211	9.56	QHDRKFYEGYYIIK-UHFFFAOYSA-N	14	1.12
acrylonitrile	212	15.10	NLHHRLWOUZZQLW-UHFFFAOYSA-N	9	1.13
phenylacetonitrile	213	8.65	SUSQOBVLVYHIEX-UHFFFAOYSA-N	16	1.14
benzonitrile	214	9.70	JFDZBHWFFUWGJE-UHFFFAOYSA-N	14	1.13
nitromethane	215	18.52	LYGJENNIWJXYER-UHFFFAOYSA-N	9	1.27

Continued on next page

Solvent	ID	[liquid]/ M	InChIKey	N_{SSIP}	ΔG_c/ kJ mol^{-1}
nitroethane	216	13.91	MCSAJNNLRCFZED-UHFFFAOYSA-N	11	1.21
1-nitropropane	217	11.17	JSZOAYXJRCEYSX-UHFFFAOYSA-N	13	1.17
2-nitropropane	218	11.04	FGLBSLMDCBOPQK-UHFFFAOYSA-N	13	1.16
nitrobenzene	219	9.74	LQNUZADURLCDLV-UHFFFAOYSA-N	15	1.17
formamide	220	25.06	ZHNUHDYFZUAESO-UHFFFAOYSA-N	7	1.30
N-methylformamide	221	16.92	ATHHXGZTWNVVOU-UHFFFAOYSA-N	10	1.28
N,N-dimethylformamide	222	12.90	ZMXDDKWLCZADIW-UHFFFAOYSA-N	12	1.21
N,N-dimethylthioformamide	223	11.74	SKECXRFZFFAANN-UHFFFAOYSA-N	13	1.20
N,N-diethylformamide	224	8.98	SUAKHGWARZSWIH-UHFFFAOYSA-N	15	1.12
N-methylacetamide	225	12.99	OHLUUHNLEMFGTQ-UHFFFAOYSA-N	12	1.22
N,N-dimethylacetamide	226	10.75	FXHOOIRPVKKKFG-UHFFFAOYSA-N	13	1.14
N,N-diethylacetamide	227	7.86	AJFDBNQQDYLMJN-UHFFFAOYSA-N	17	1.11

Continued on next page

Solvent	ID	[liquid]/ M	InChIKey	N_{SSIP}	ΔG_c/ kJ mol^{-1}
2-pyrrolidinone	228	13.01	HNJBEVLQSNELDL-UHFFFAOYSA-N	12	1.22
N-methyl pyrrolidinone	229	10.37	SECXISVLQFMRJM-UHFFFAOYSA-N	14	1.17
1-methyl-2-pyrrolidinethione	230	11.66	OQILOJRSIWGQSM-UHFFFAOYSA-N	15	1.30
tetramethylurea	231	8.31	AVQQQNCBBIEMEU-UHFFFAOYSA-N	17	1.15
tetraethylurea	232	5.26	UWHSPZZUAYSGTB-UHFFFAOYSA-N	24	1.08
dimethylcyanamide	233	12.38	OAGOUCJGXNLJNL-UHFFFAOYSA-N	12	1.19
carbon disulfide	234	16.50	QGJOPFRUJISHPQ-UHFFFAOYSA-N	9	1.19
dimethyl sulfide	235	13.55	QMMFVYPAHWMCMS-UHFFFAOYSA-N	10	1.12
diethyl sulfide	236	9.22	LJSQFQKUNVCTIA-UHFFFAOYSA-N	15	1.14
diisopropyl sulfide	237	6.88	XYWDPYKBIRQXQS-UHFFFAOYSA-N	18	1.07
dibutyl sulfide	238	5.73	HTIRHQRTDBPHNZ-UHFFFAOYSA-N	23	1.11
tetrahydrothiophene	239	11.27	RAOIDOHSFRTOEL-UHFFFAOYSA-N	13	1.18

Continued on next page

Solvent	ID	[liquid]/ M	InChIKey	N_{SSIP}	ΔG_c/ kJ mol^{-1}
thiane	240	9.63	YPWFISCTZQNZAU-UHFFFAOYSA-N	14	1.12
dimethylsulfoxide	241	14.03	IAZDPXIOMUYVGZ-UHFFFAOYSA-N	11	1.21
dibutyl sulfoxide	242	5.13	LOWMYOWHQMKBTM-UHFFFAOYSA-N	23	1.03
sulfolane	243	10.50	HXJUTPCZVOIRIF-UHFFFAOYSA-N	14	1.18
thiobis(2-ethanol)	244	9.67	YODZTKMDCQEPHD-UHFFFAOYSA-N	16	1.21
diethyl sulfite	245	7.84	NVJBFARDFTXOTO-UHFFFAOYSA-N	18	1.15
dimethyl sulfate	246	10.57	VAYGXNSJCAHWJZ-UHFFFAOYSA-N	14	1.18
diethyl sulfate	247	7.64	DENRZWYUOJLTMF-UHFFFAOYSA-N	18	1.13
methanesulfonic acid	248	15.37	AFVFQIVMOAPDHO-UHFFFAOYSA-N	11	1.28
trimethylphosphate	249	8.67	WVLBCYQITXONBZ-UHFFFAOYSA-N	17	1.18
triethylphosphate	250	5.87	DQWPFSLDHJDLRL-UHFFFAOYSA-N	23	1.12
tri-n-butylphosphate	251	3.65	STCOOQWBFONSKY-UHFFFAOYSA-N	36	1.10

Continued on next page

Solvent	ID	[liquid]/ M	InChIKey	N_{SSIP}	ΔG_c/ kJ mol^{-1}
hexamethylphosphoric triamide	252	5.69	GNOIPBMMFNIUFM-UHFFFAOYSA-N	22	1.07
hydrogen peroxide	253	42.37	MHAJPDPJQMAIIY-UHFFFAOYSA-N	5	1.44
hydrogen fluoride	254	47.62	KRHYYFGTRYWZRS-UHFFFAOYSA-N	3	1.16
sulfuric acid	255	18.69	QAOWNCQODCNURD-UHFFFAOYSA-N	10	1.35
ammonia	256	40.00	QGZKDVFQNNGYKY-UHFFFAOYSA-N	5	1.40
hydrazine	257	31.35	OAKJQQAXSVQMHS-UHFFFAOYSA-N	7	1.47
sulfur dioxide	258	22.83	RAHZWNYVWXNFOC-UHFFFAOYSA-N	7	1.24
thionyl chloride	259	13.71	FYSNRJHAOHDILO-UHFFFAOYSA-N	11	1.20
phosphorus oxychloride	260	10.87	XHXFXVLFKHQFAL-UHFFFAOYSA-N	13	1.15
dihydrolevoglucosenone	261	9.76	WHIRALQRTSITMI-UJURSFKZSA-N	15	1.18

Table S1: Solvent information.[1,2]

Solvent SSIP Descriptions

All solvent molecules were footprinted as described previously.[3] The SSIP description of some molecules is dependent on the conformation used in the calculation.[4] By using an extended conformation in these cases, it is possible to capture all of the possible interaction sites on the surface of the molecule. For example, the two hydroxyl groups of ethylene glycol are represented by four hydrogen bond acceptor SSIPs associated with the four oxygen lone pairs and two hydrogen bond donor SSIPs associated with the two hydroxyl protons. Ethylene glycol can adopt a folded conformation with an intramolecular hydrogen bond, and when the molecule is footprinted in this geometry, the SSIPs associated with one of the oxygen lone pairs and one of the hydroxyl protons are missing. However, when ethylene glycol is the solvent, the molecules are always in close contact, so we assume that the probability of intermolecular and intramolecular hydroxyl-hydroxyl interactions are similar. Thus the footprint of the extended conformation provides a good description of the solvent properties of this molecule, because the SSIMPLE approach does not differentiate between intermolecular and intramolecular SSIP interactions, i.e. the probability of intramolecular hydrogen bonding in a folded conformation is built into the SSIMPLE calculation.

Self-association of alcohols leads to an increase in the hydroxyl group SSIPs for the solvent compared with the monomeric molecule. The positive hydroxyl SSIP was adjusted to the experimental solvent value (+3.5) and one negative hydroxyl SSIP was adjusted to the experimental solvent value (-6.9) for alcohols that self-associate: methanol, ethanol, 1-propanol, 2-propanol, 1-butanol, 2-methyl-1-propanol, 2-butanol, 2-methyl-2-propanol, 1-pentanol, 3-methyl-1-butanol, 2-methyl-2-butanol, 1-hexanol, cyclohexanol, 1-octanol, 1-decanol, 1-dodecanol, benzyl alcohol, 2-phenylethanol, allyl alcohol, 2-methoxyethanol, 2-ethoxyethanol, ethylene glycol, 1,2-propanediol, 1,3-propanediol, 1,2-butanediol, 2,3-butanediol, 1,4-butanediol, 1,5-pentanediol, diethylene glycol, triethylene glycol, glycerol.

The solvent SSIP ϵ_i values are plotted below for each molecule. Experimental solute values of α and/or β,[3] are plotted as red crosses. Mean experimental functional group values of α and/or β[5] and the experimental solvent parameters for self-associated alcohols are shown as horizontal black lines. If the largest SSIPs showed significant deviation from the experimental value or the mean

experimental functional group value, the calculated SSIP values were manually adjusted. Changes were required for five solvents as detailed in table S2.

Table S2: Solvent SSIP modifications

Solvent	Calculated ϵ_i	Corrected ϵ_i
water	2.9	2.8
	2.9	2.8
	-6.2	-4.5
	-6.3	-4.5
ammonia	-9.5	-6.8
1,2-dimethoxybenzene	-7.0	-3.6
	-7.9	-3.6
glycerol	0.0	-5.3
	0.0	-6.9
	-4.9	-5.3
	-5.1	-6.9
	-8.4	-5.3
	-8.4	-6.9
bis(chloroethyl)ether	-1.7	-5.3

Figure S2: SSIP description of solvent molecules. Each blue cross represents the value of ϵ_i for a single SSIP, which can be read off the y axis. The calculated SSIP values are spaced in the x direction in an arbitrary manner. Where experimentally determined values of α and/or β are available for the solvent molecule, these are plotted as red crosses, and values can be read off the y axis. The black line represents the mean experimentally determined value of α and/or β for the most polar functional group present in the solvent. Continued on next page.

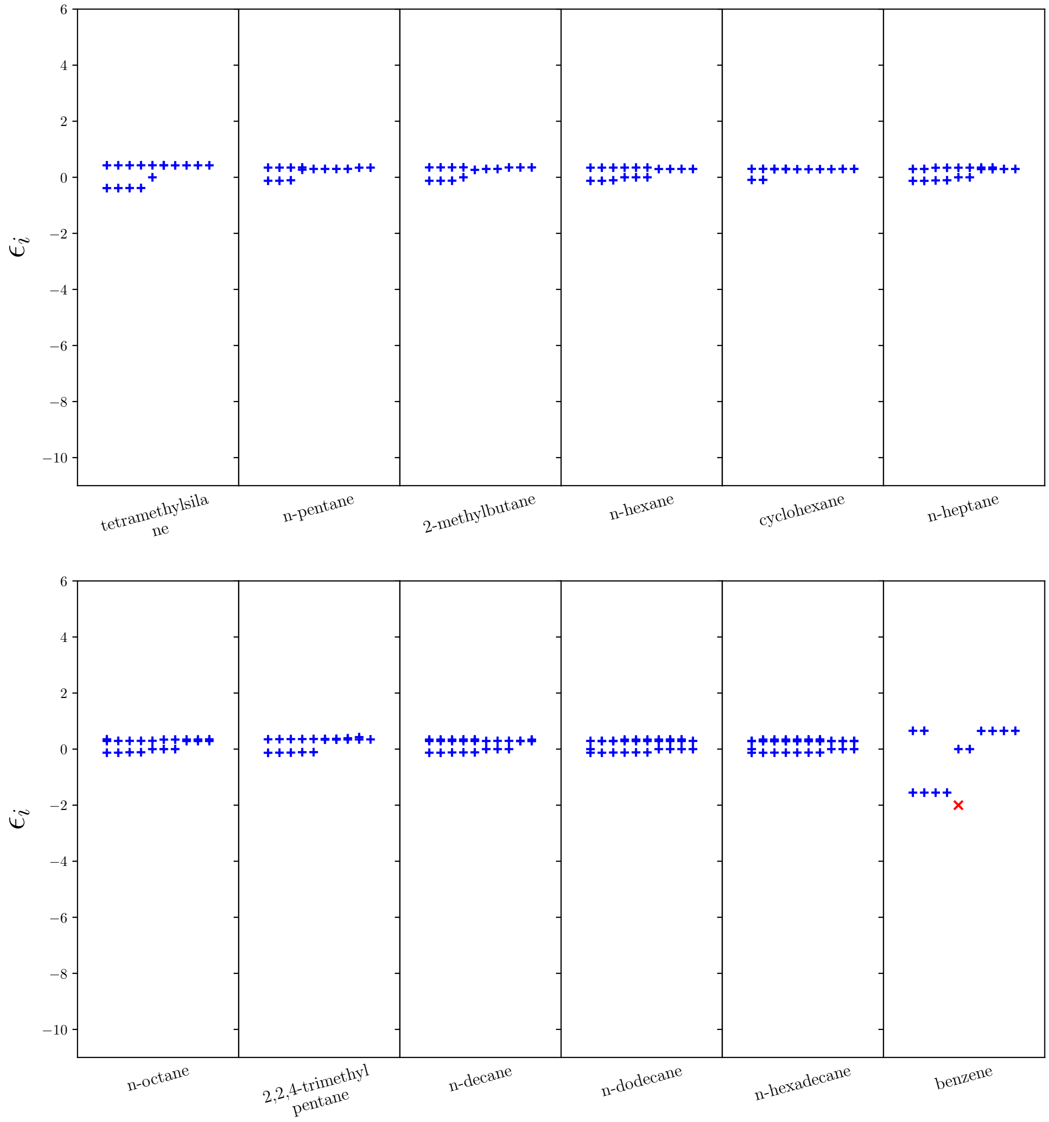

Figure S2: Continued on next page.

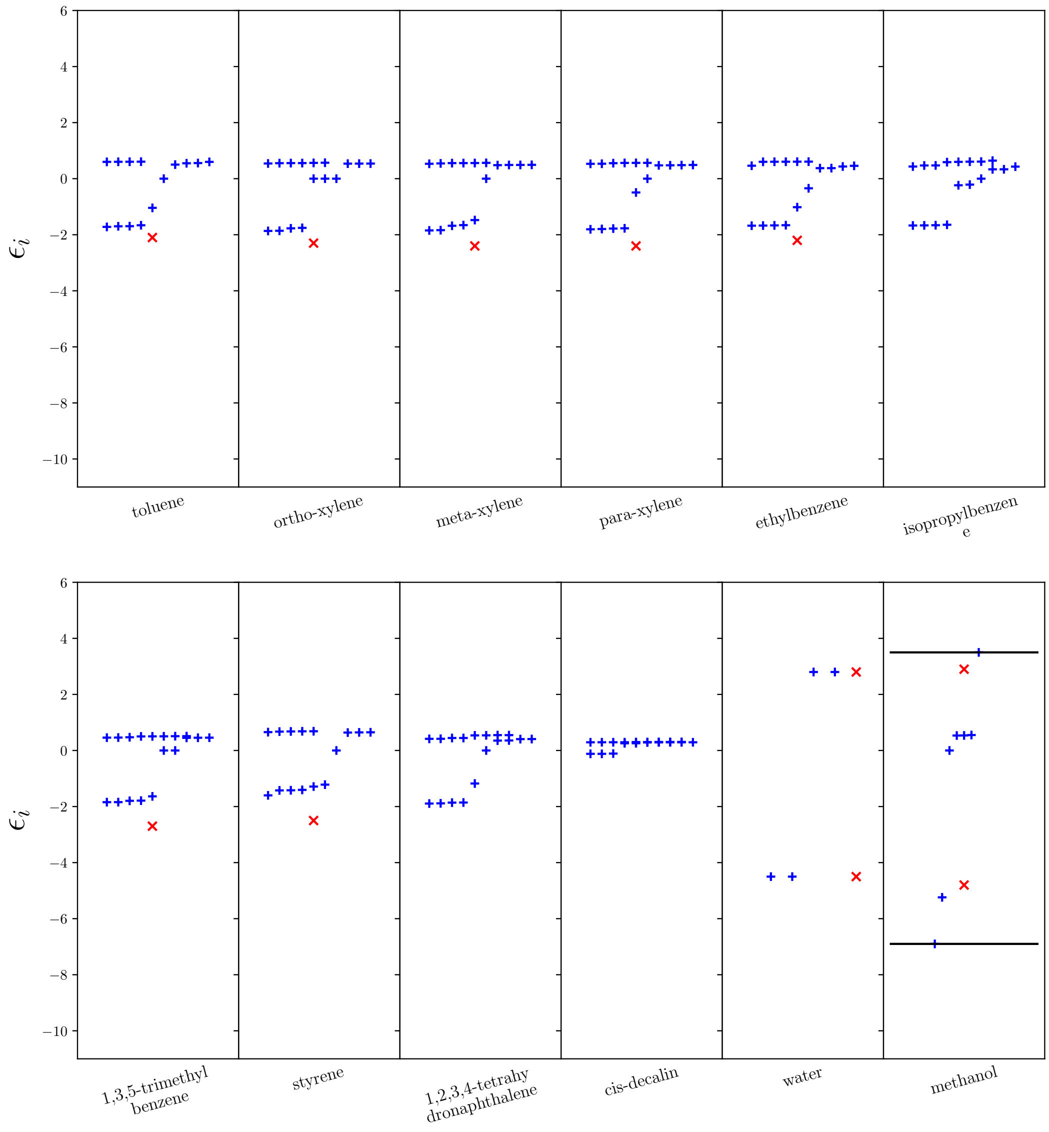

Figure S2: Continued on next page.

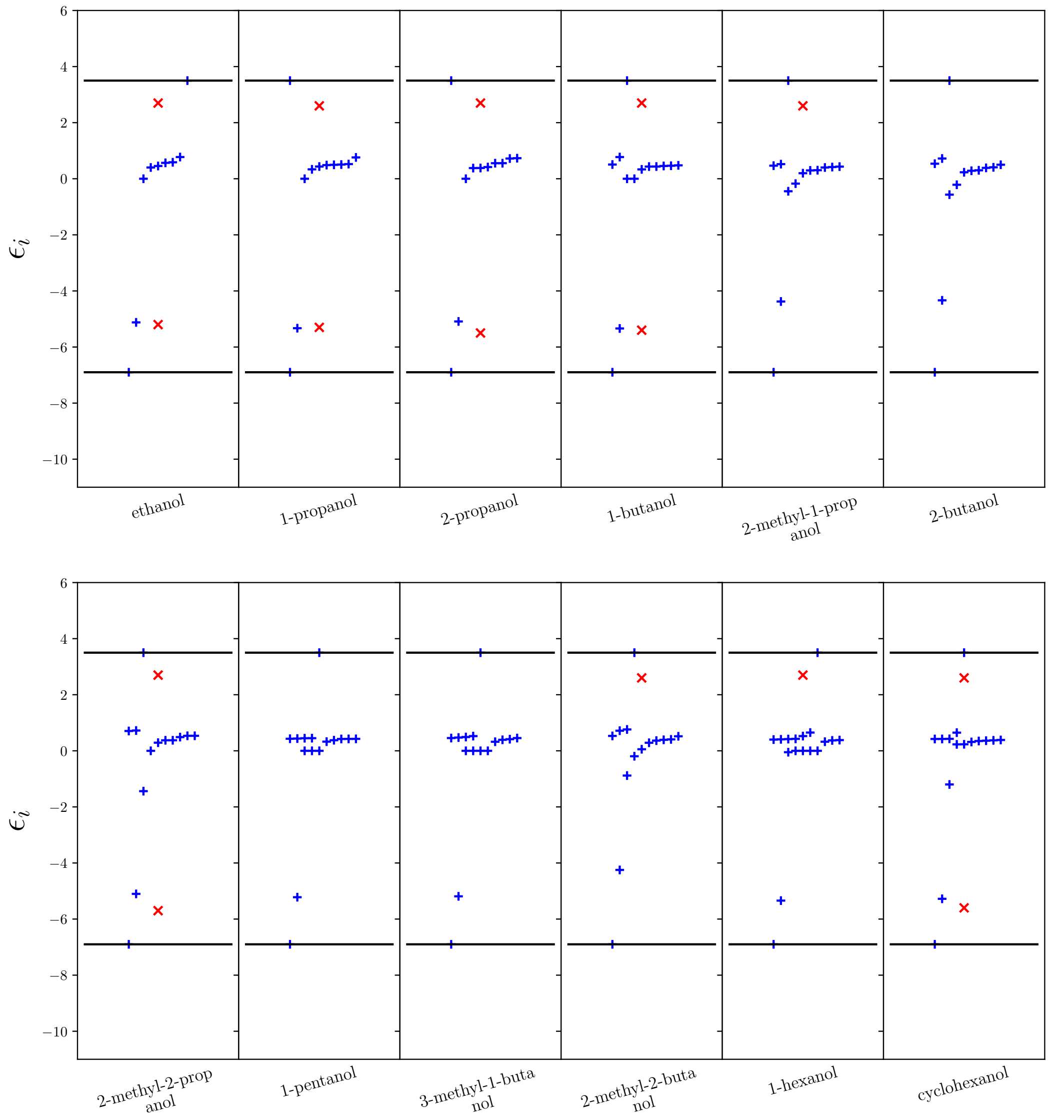

Figure S2: Continued on next page.

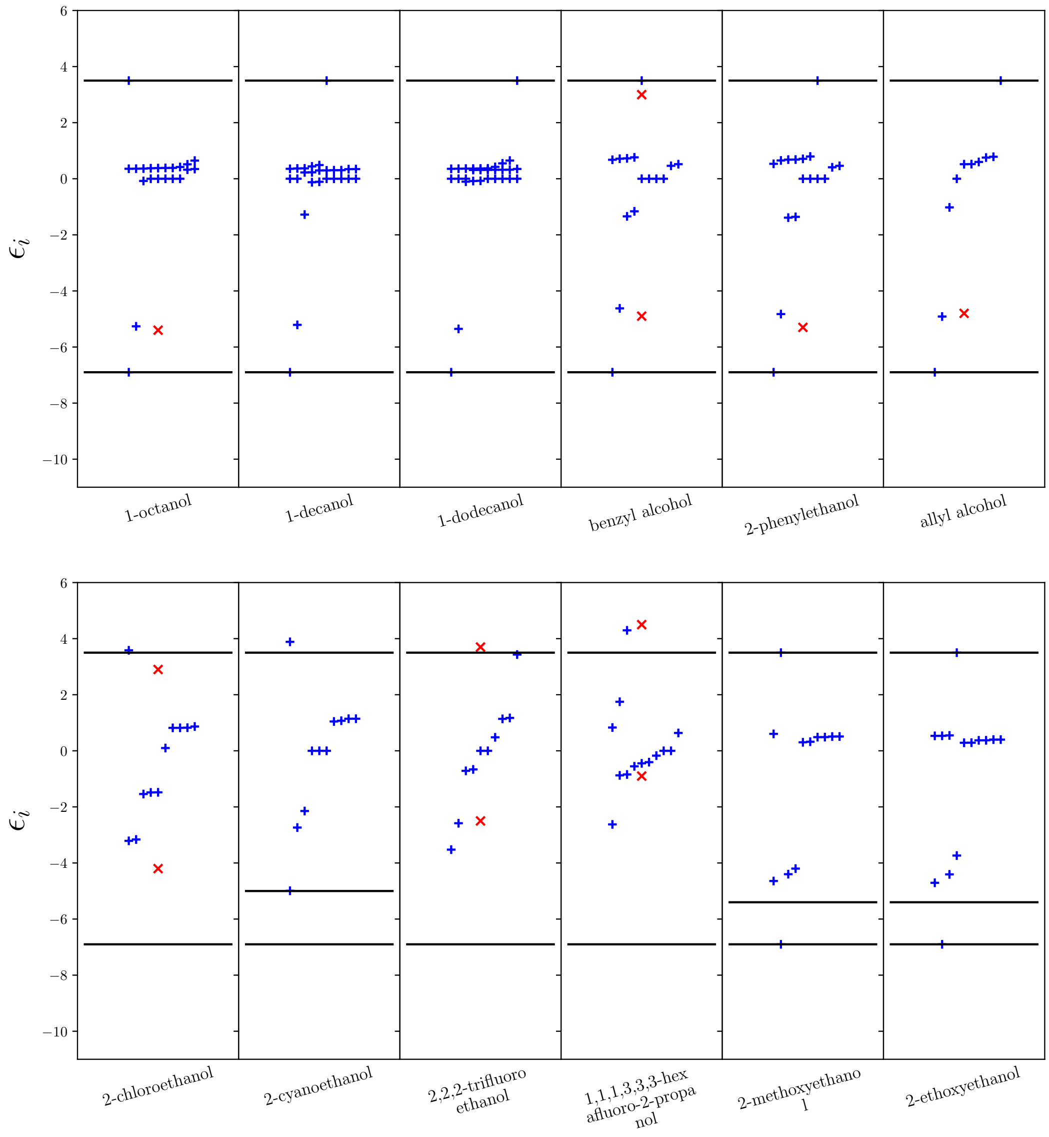

Figure S2: Continued on next page.

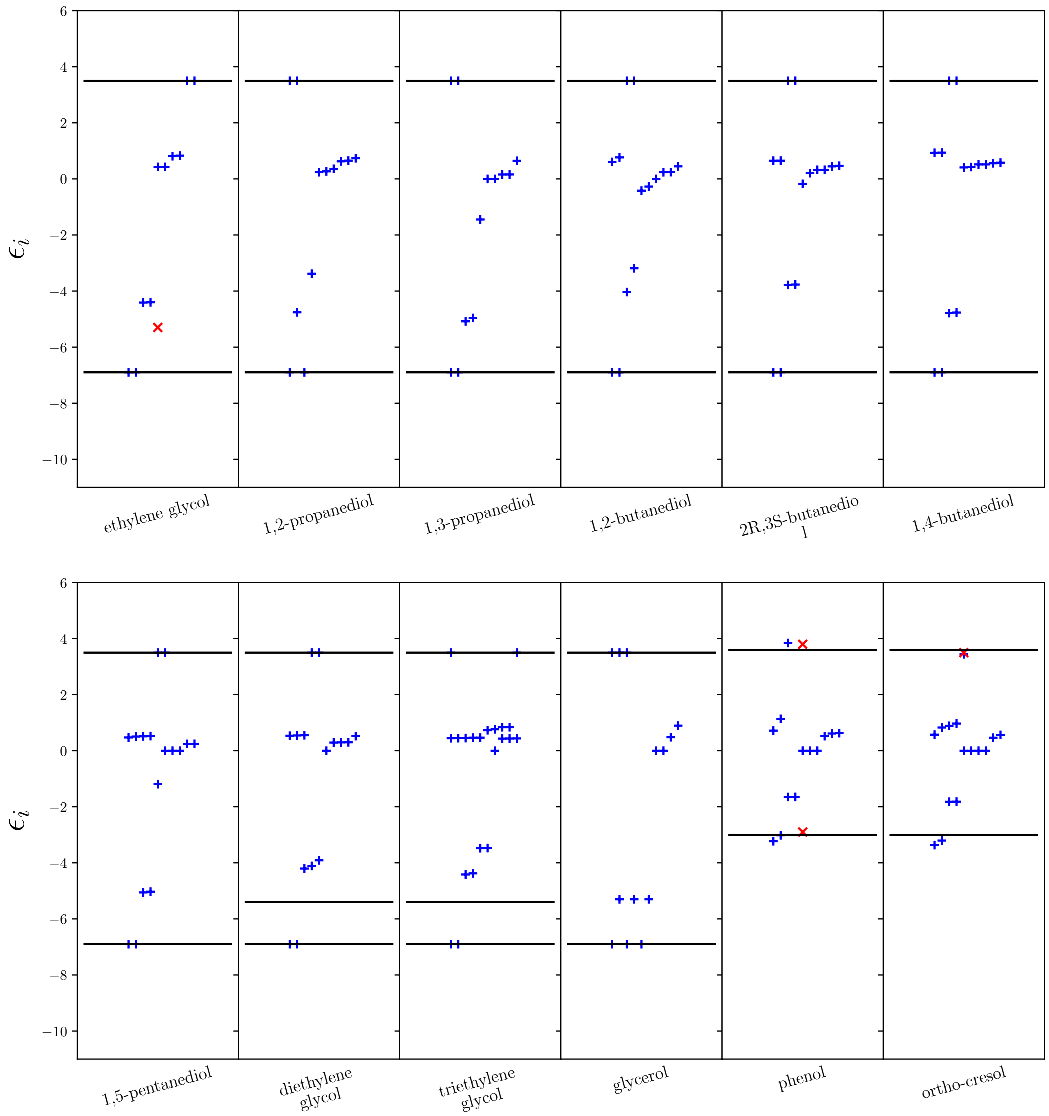

Figure S2: Continued on next page.

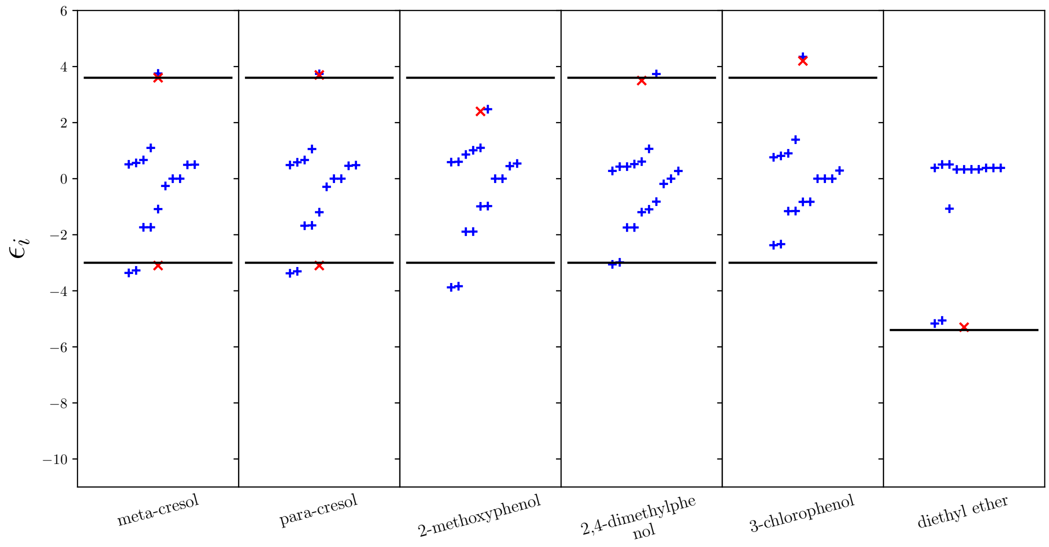

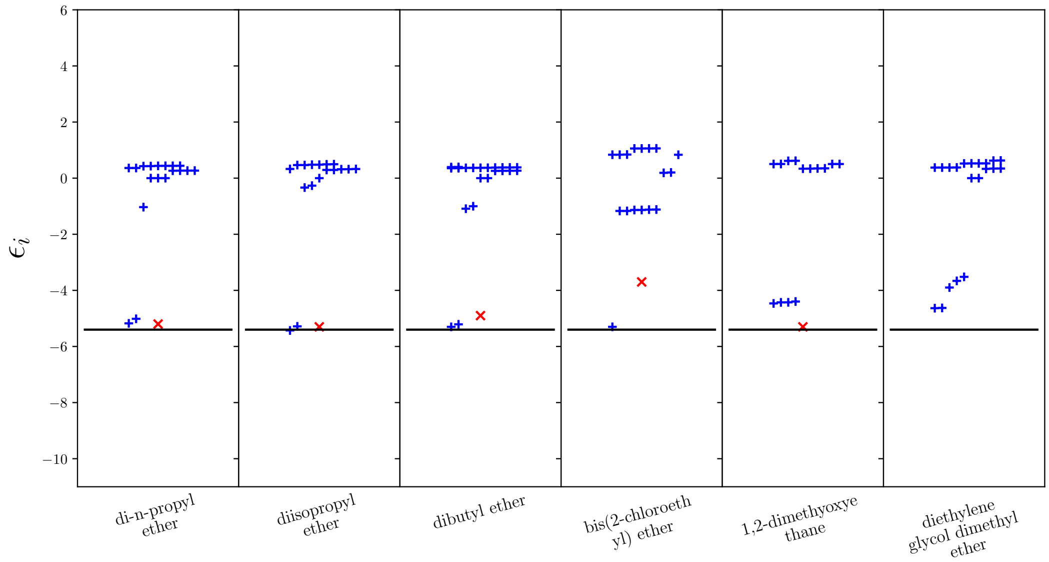

Figure S2: Continued on next page.

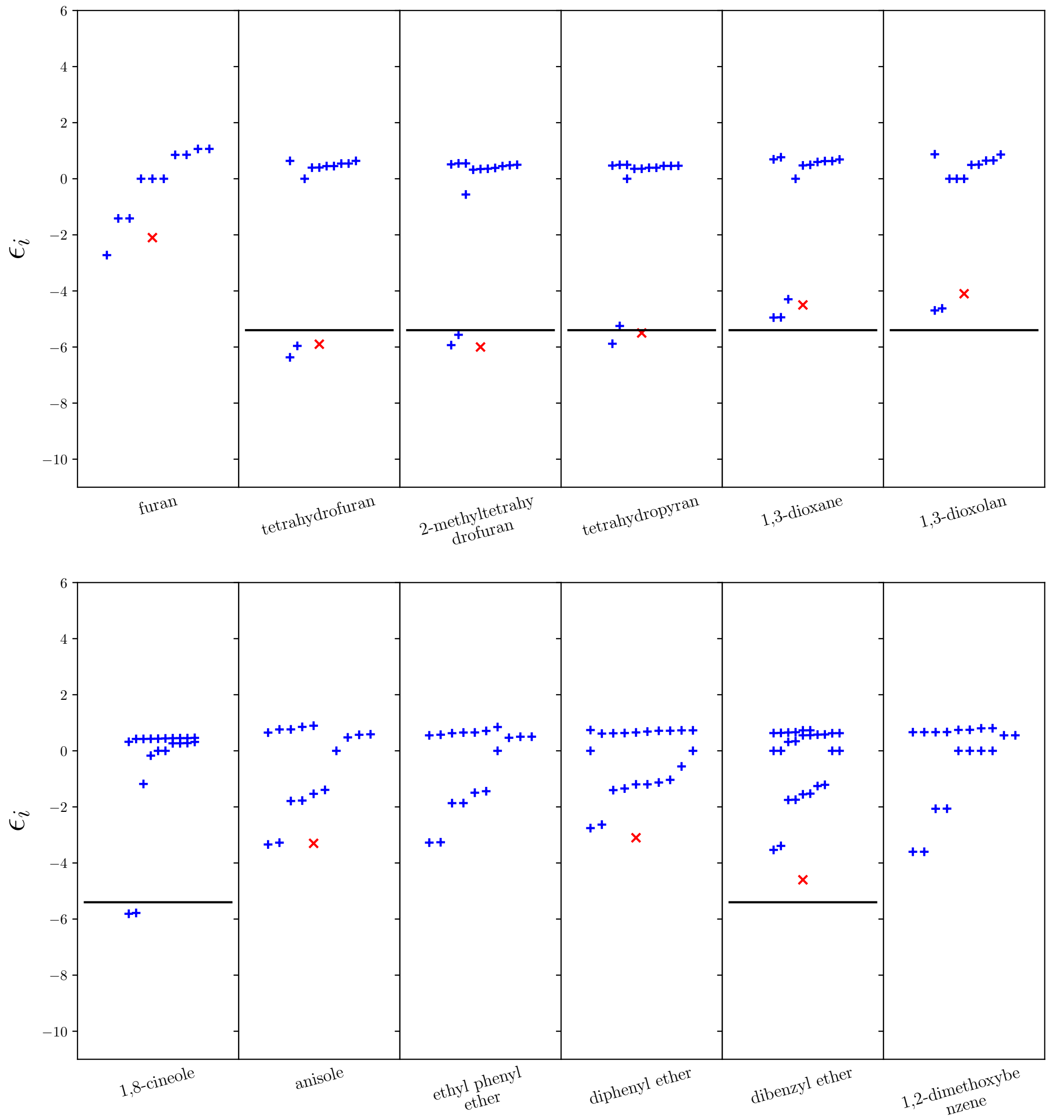

Figure S2: Continued on next page.

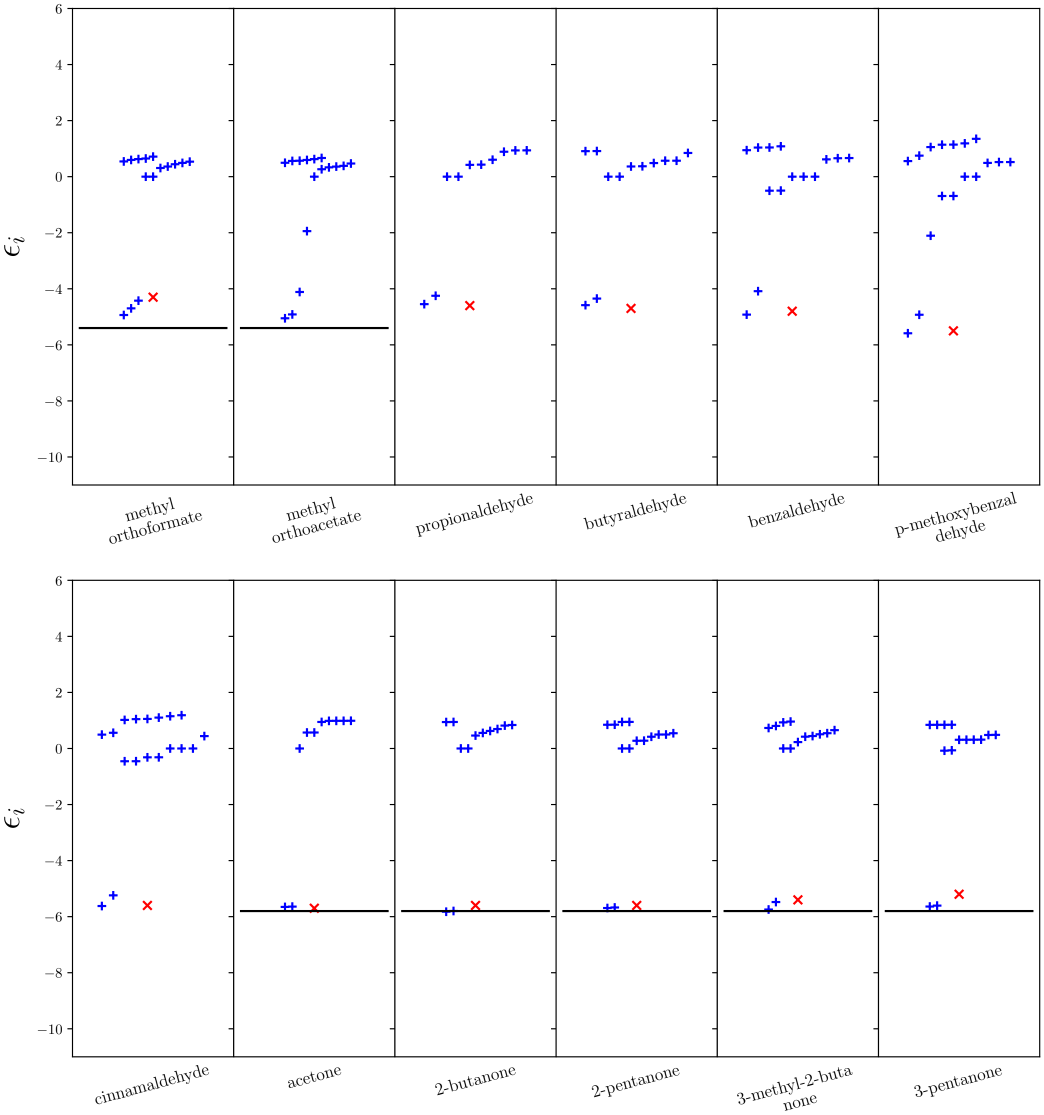

Figure S2: Continued on next page.

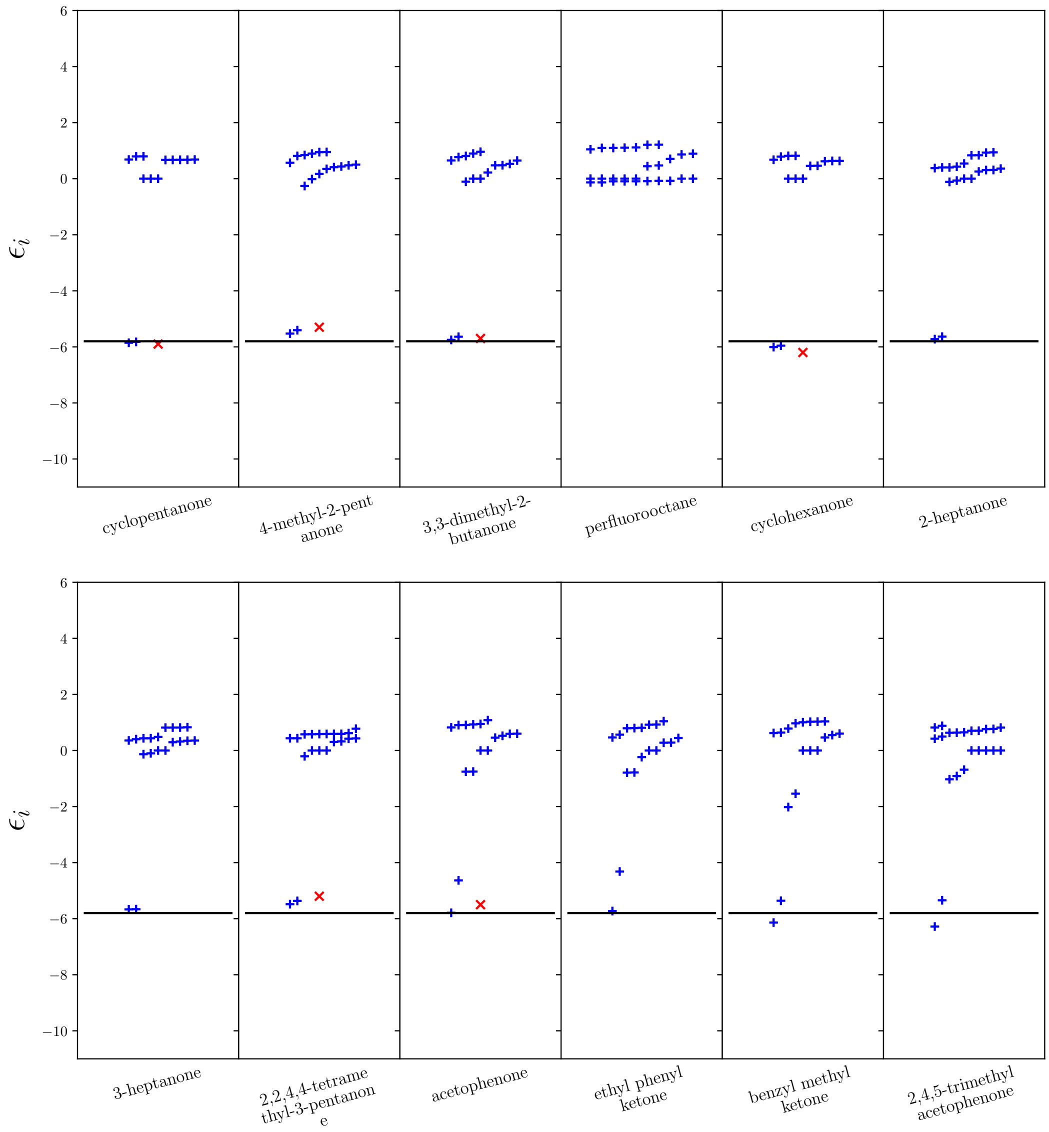

Figure S2: Continued on next page.

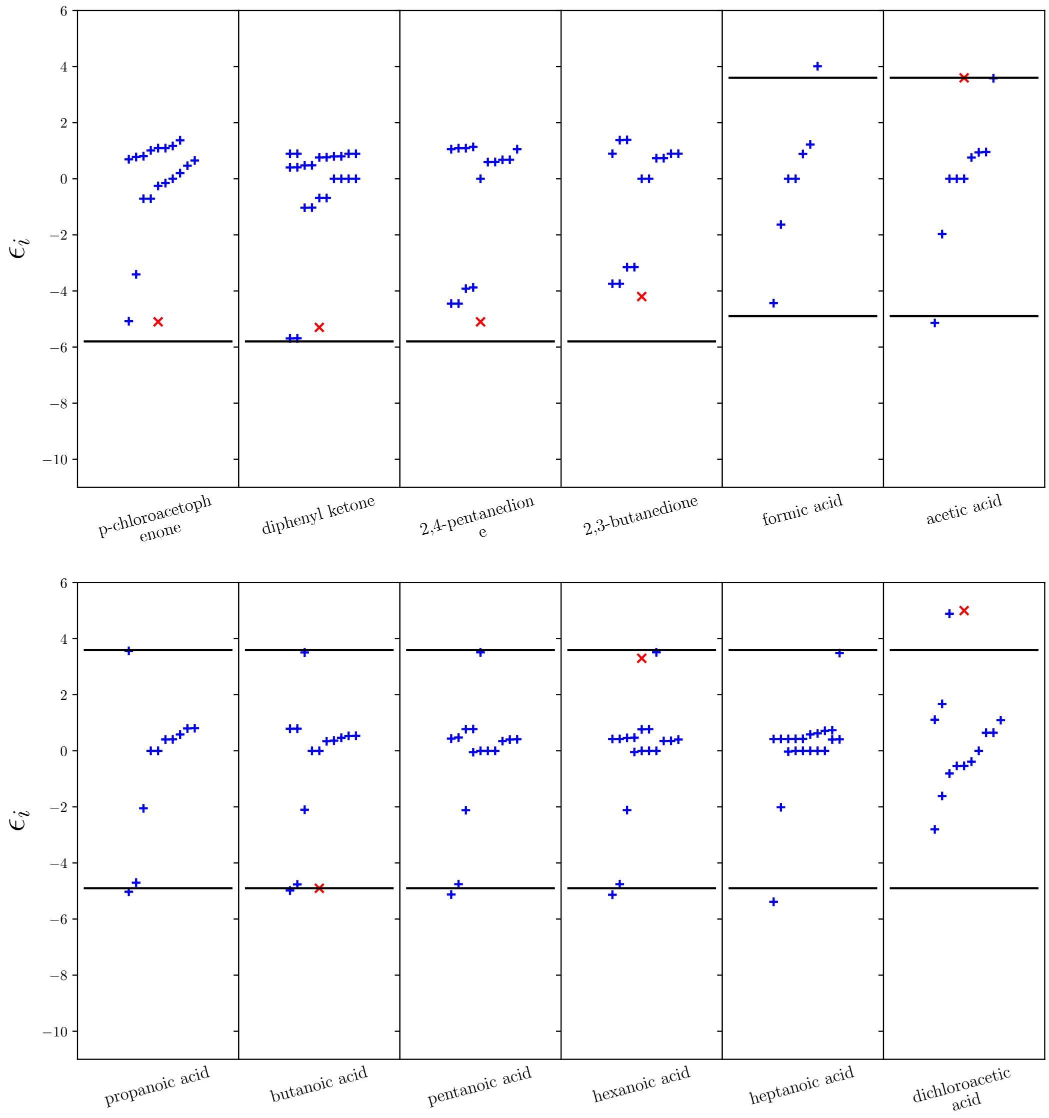

Figure S2: Continued on next page.

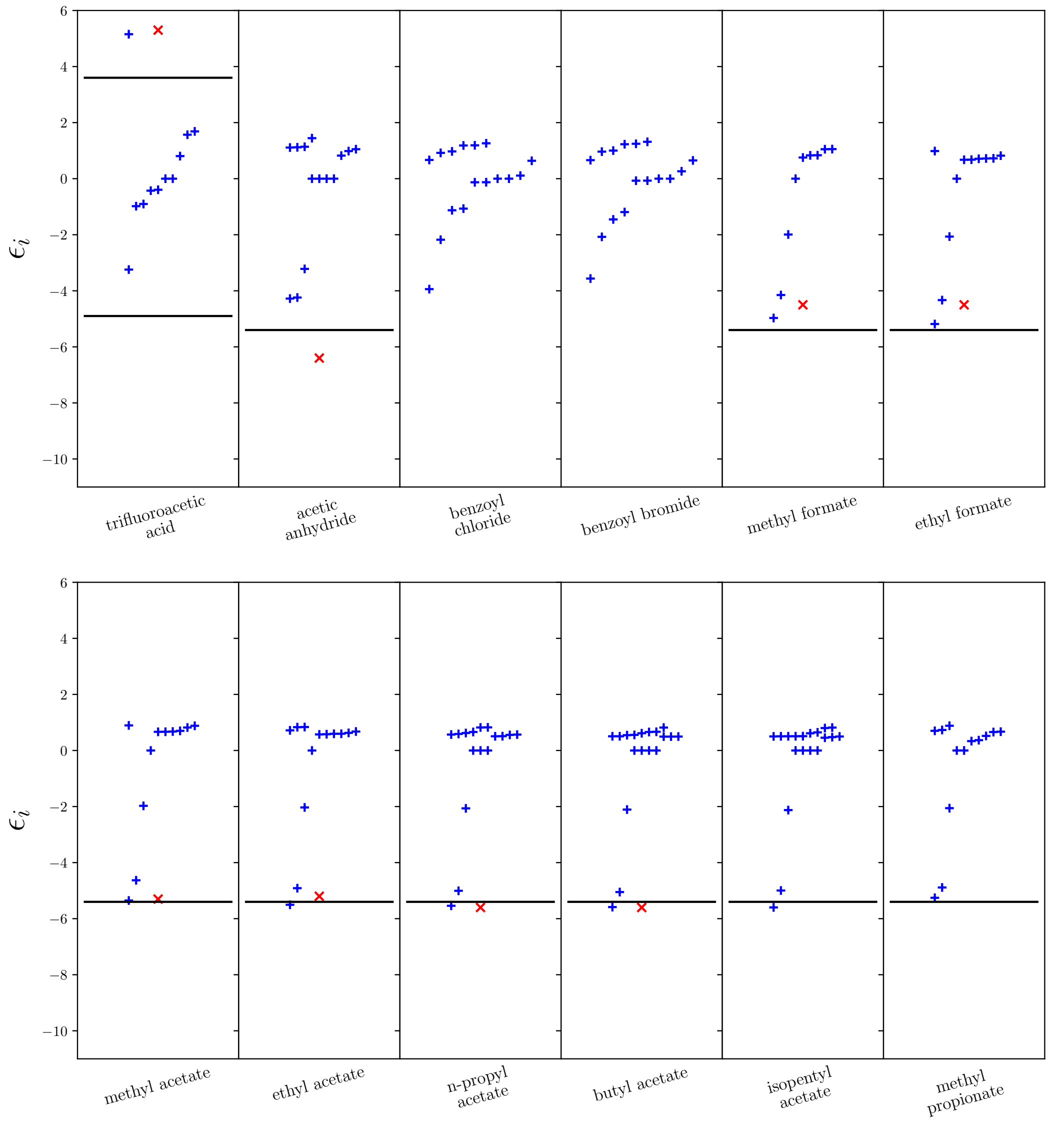

Figure S2: Continued on next page.

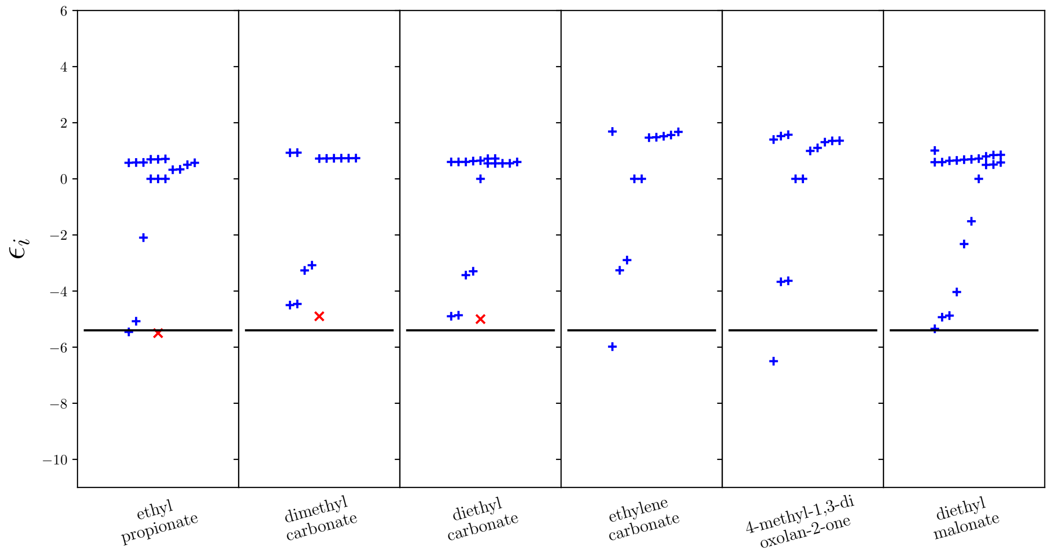

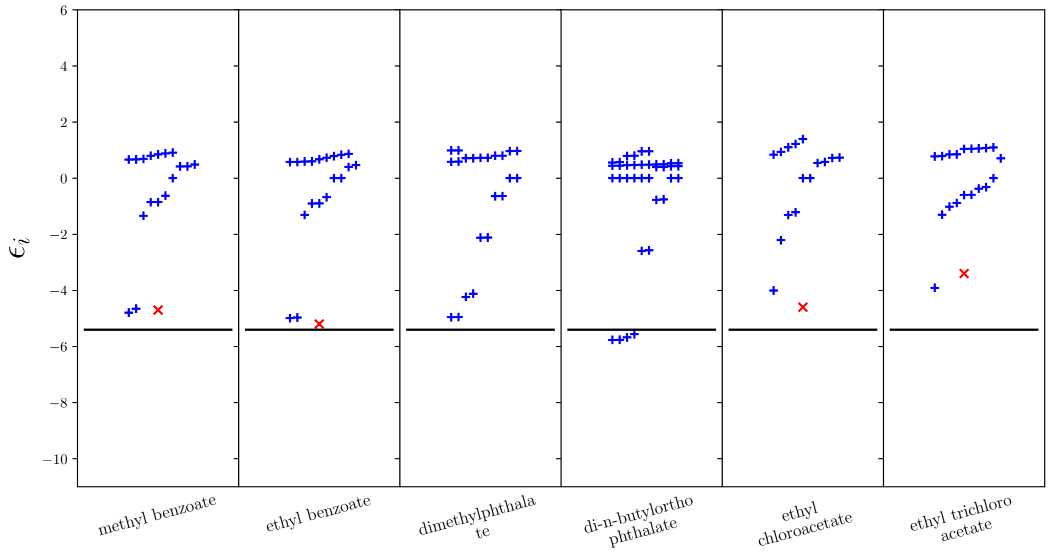

Figure S2: Continued on next page.

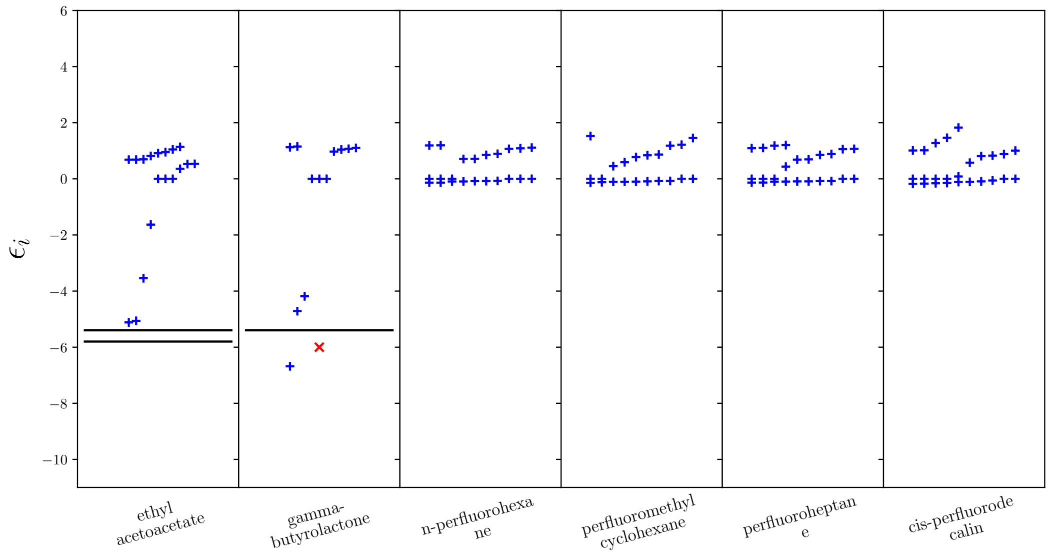

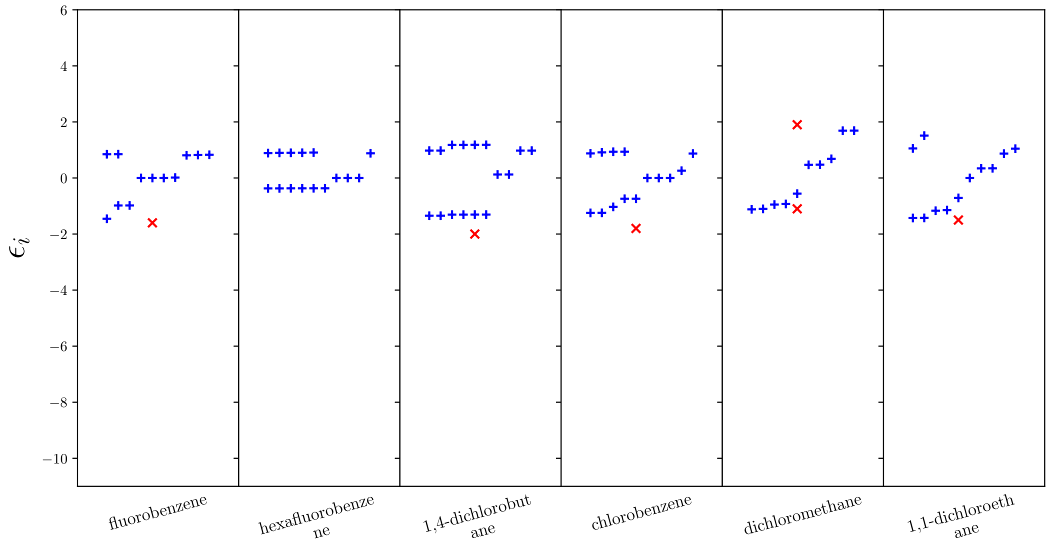

Figure S2: Continued on next page.

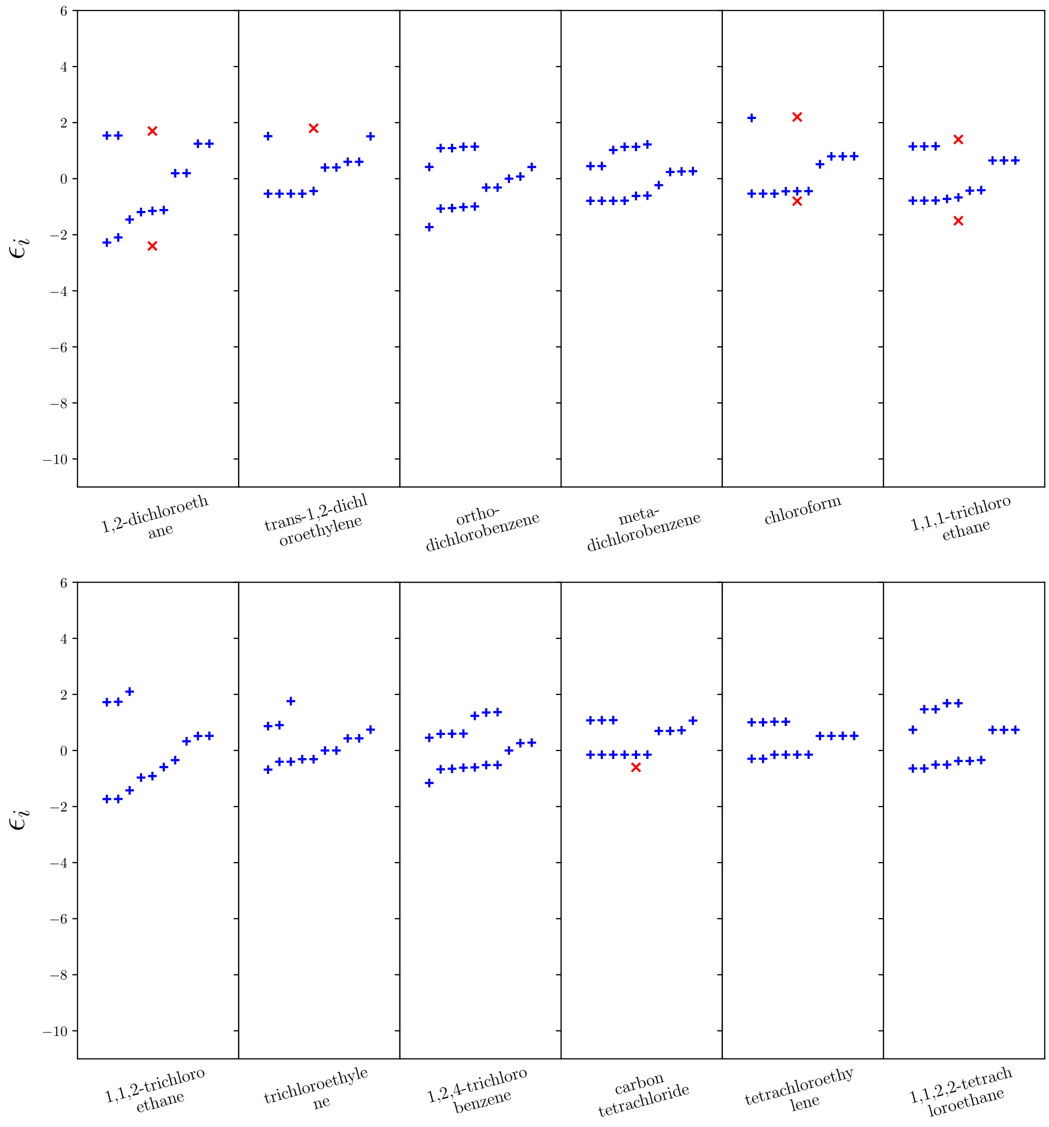

Figure S2: Continued on next page.

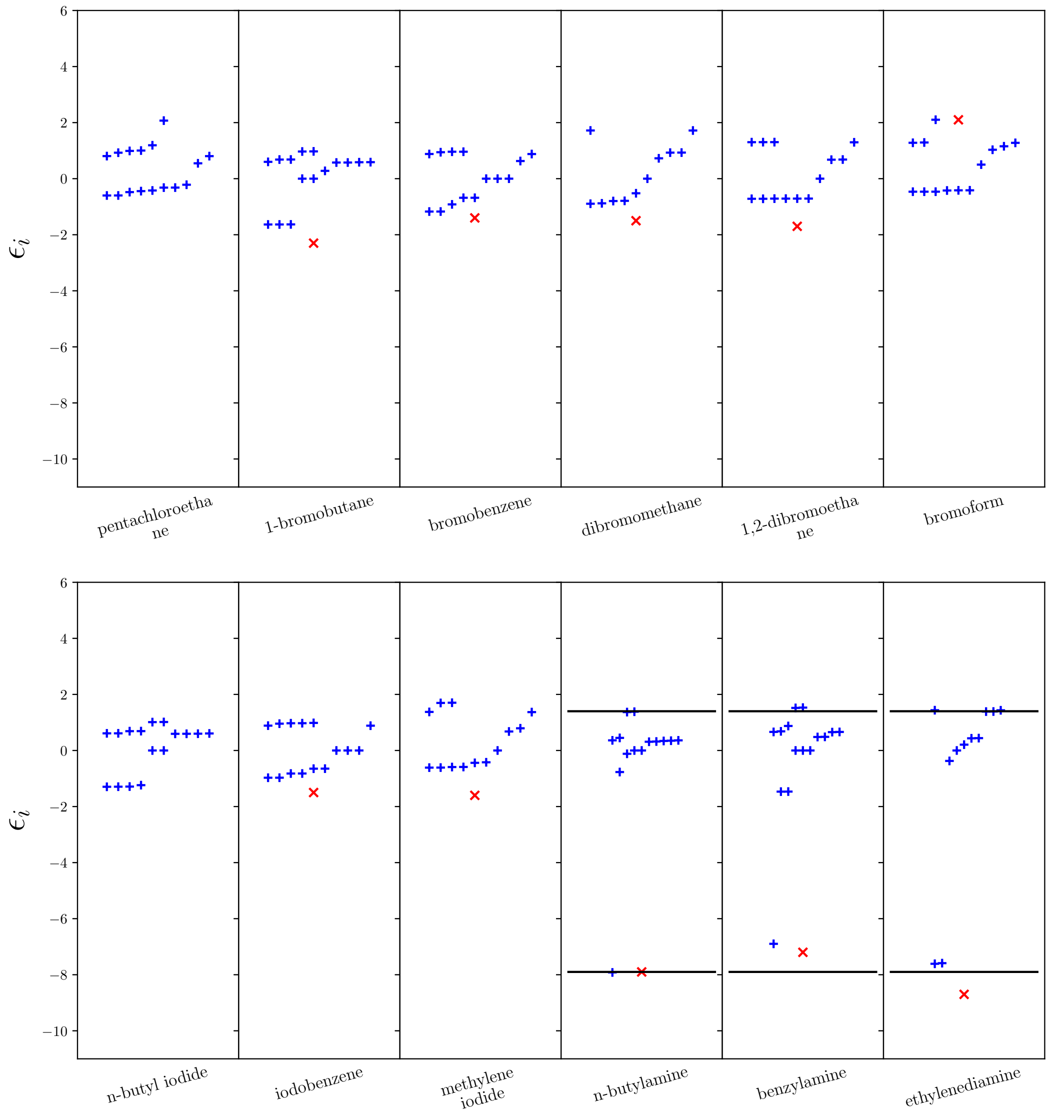

Figure S2: Continued on next page.

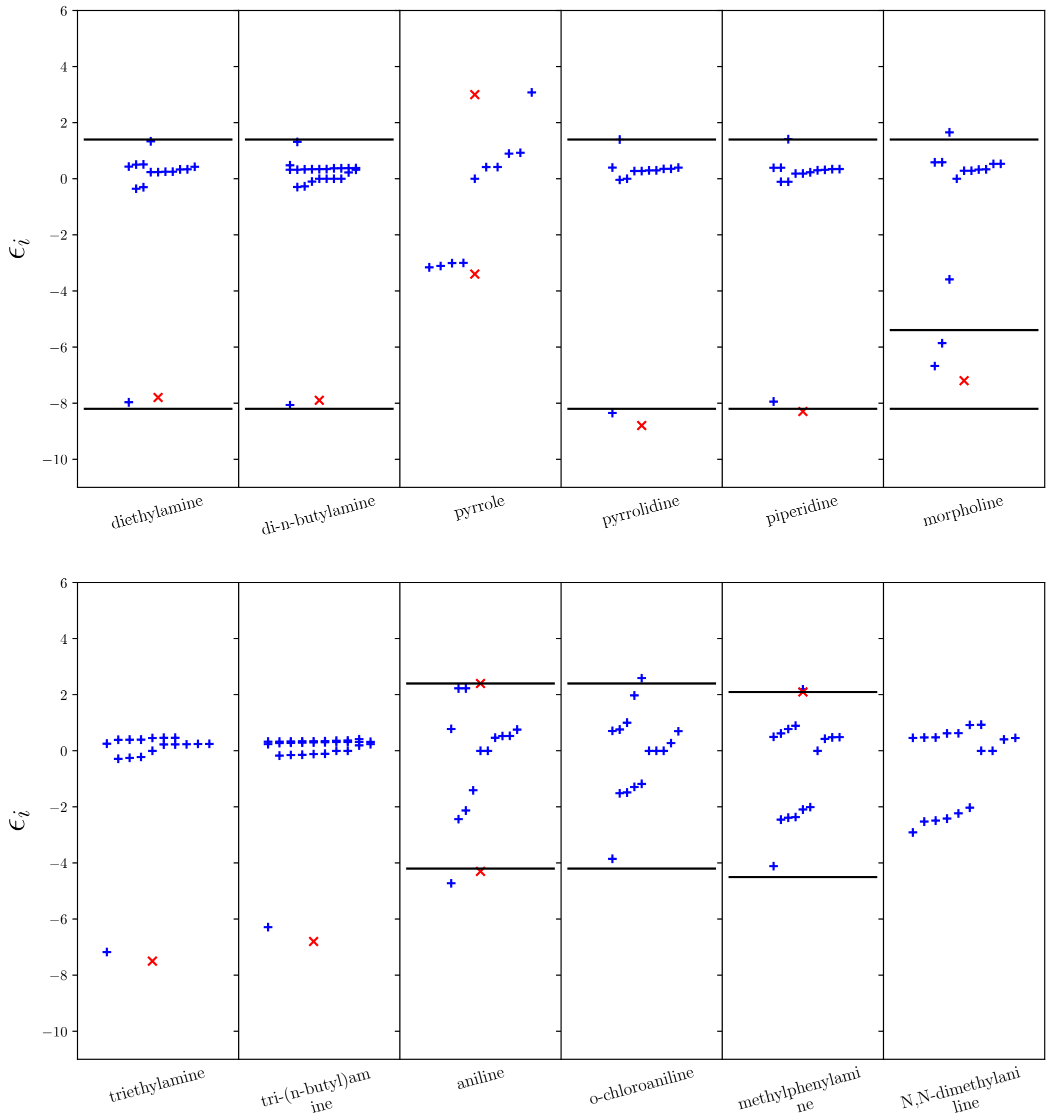

Figure S2: Continued on next page.

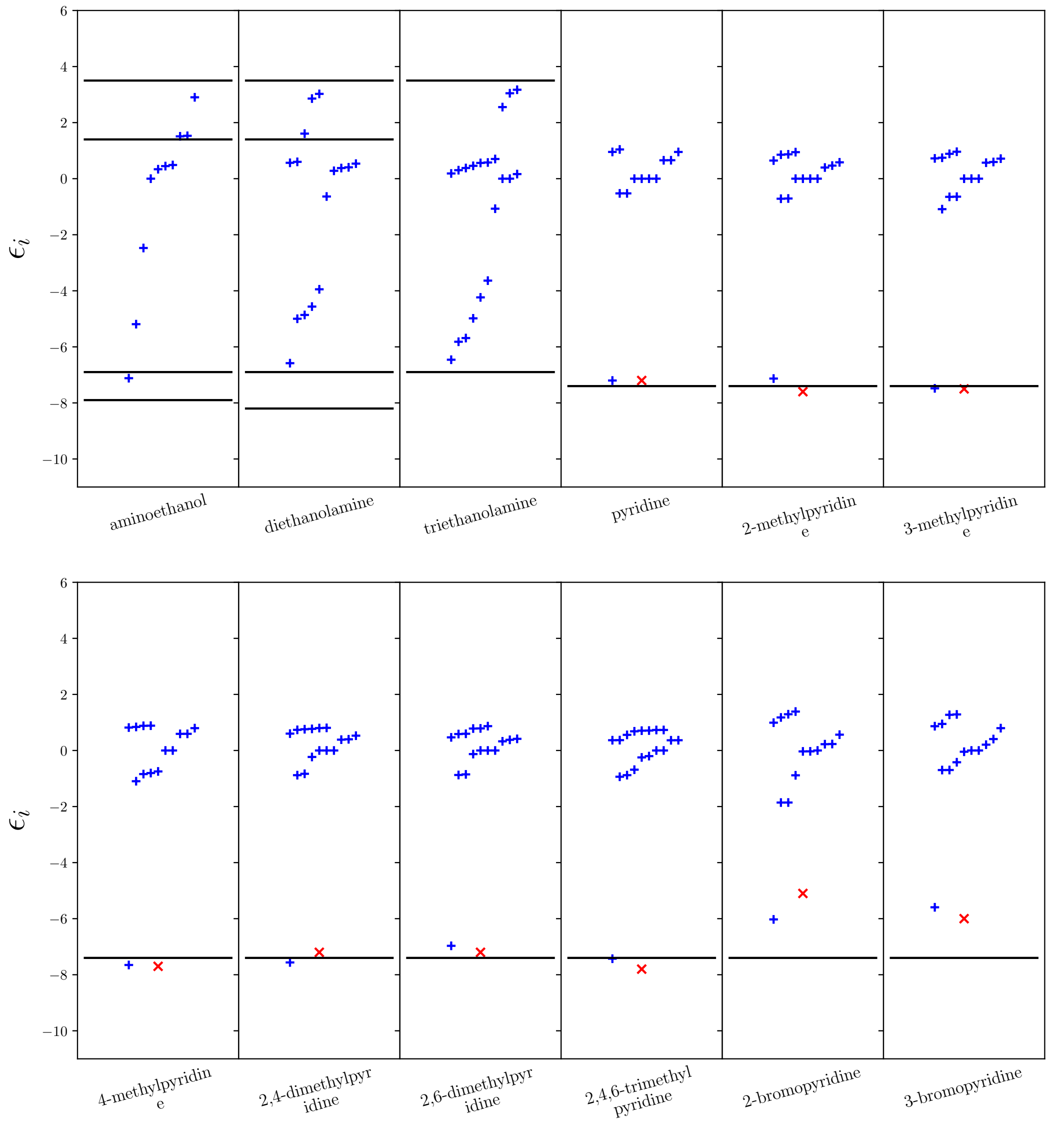

Figure S2: Continued on next page.

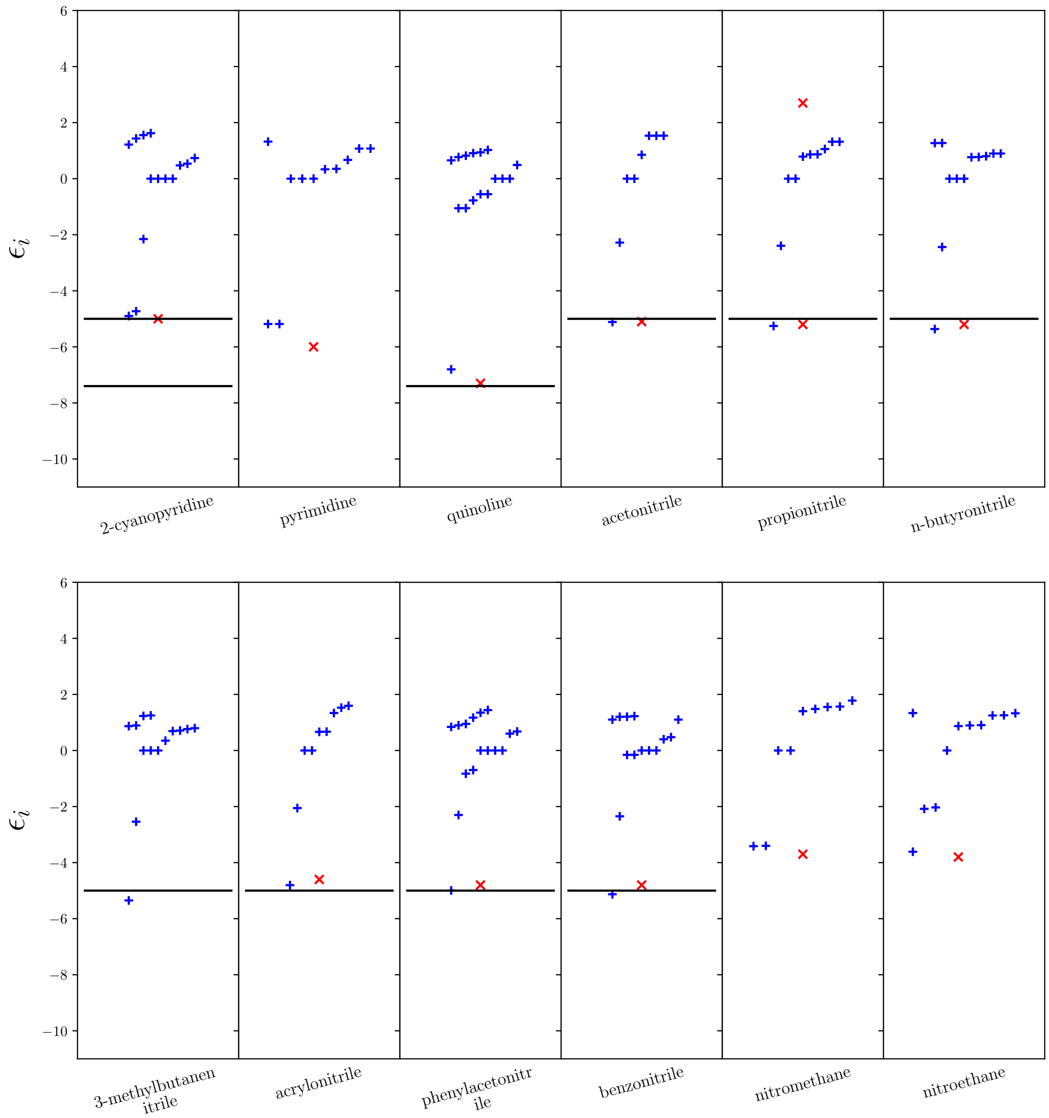

Figure S2: Continued on next page.

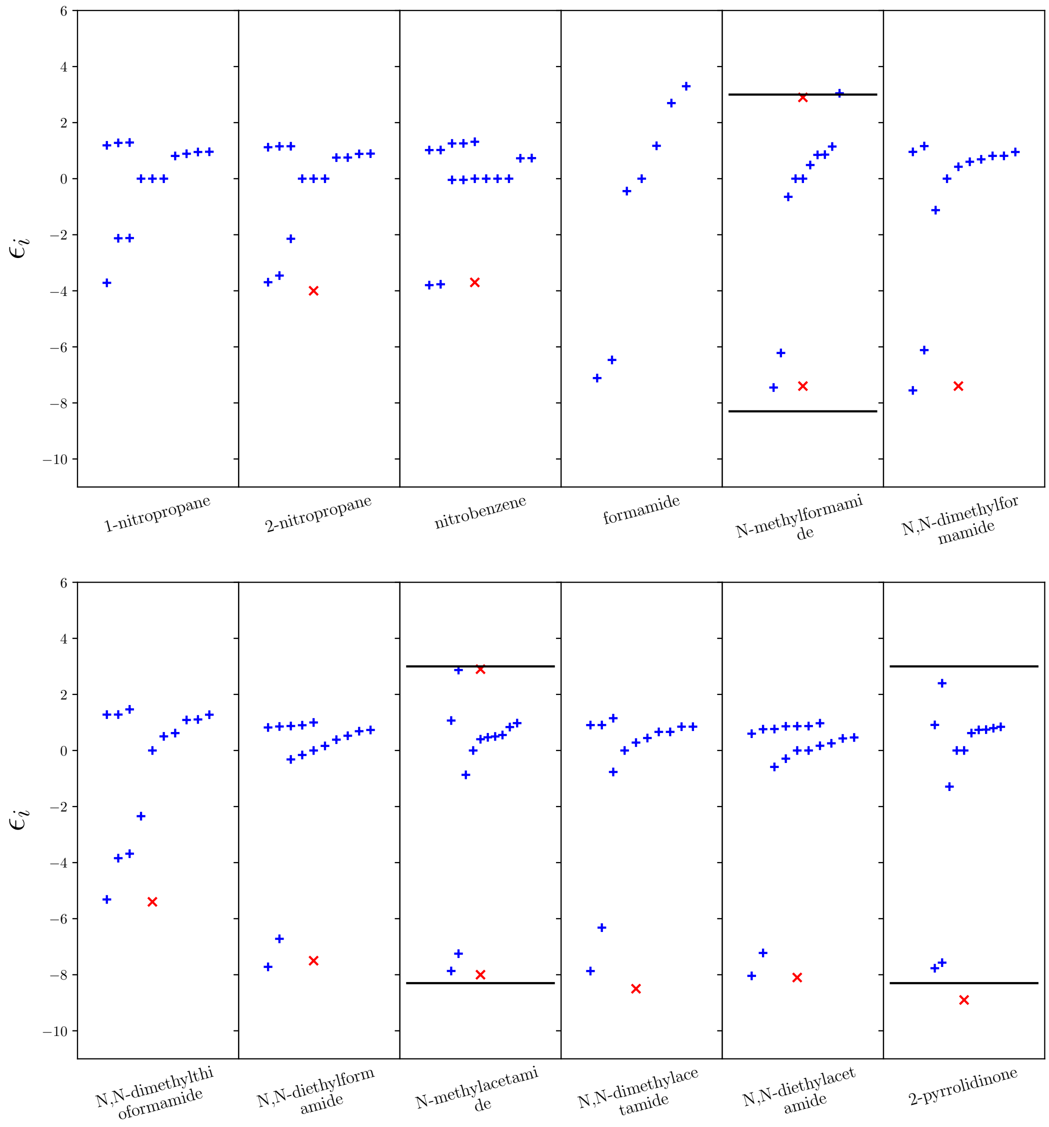

Figure S2: Continued on next page.

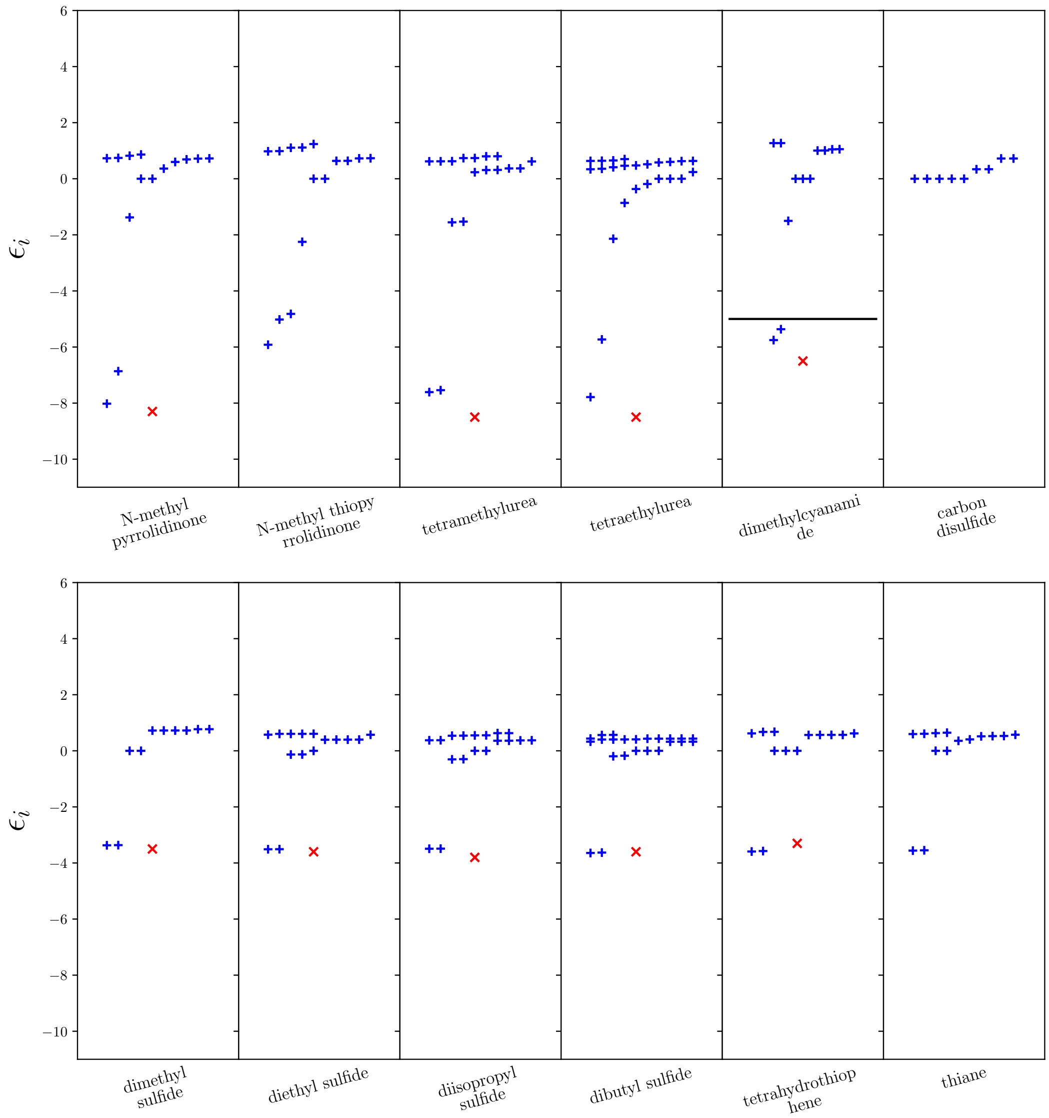

Figure S2: Continued on next page.

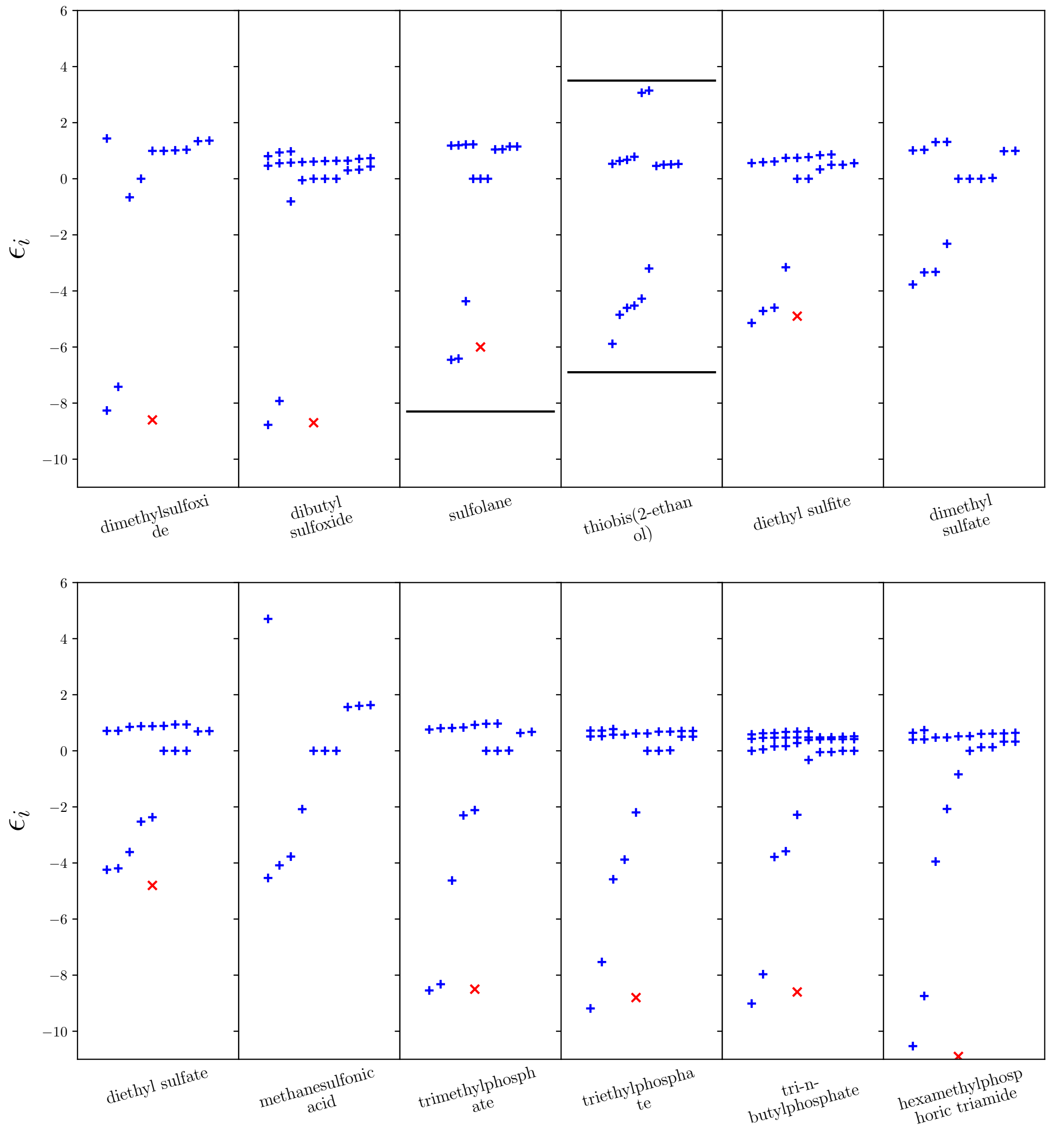

Figure S2: End of figure.

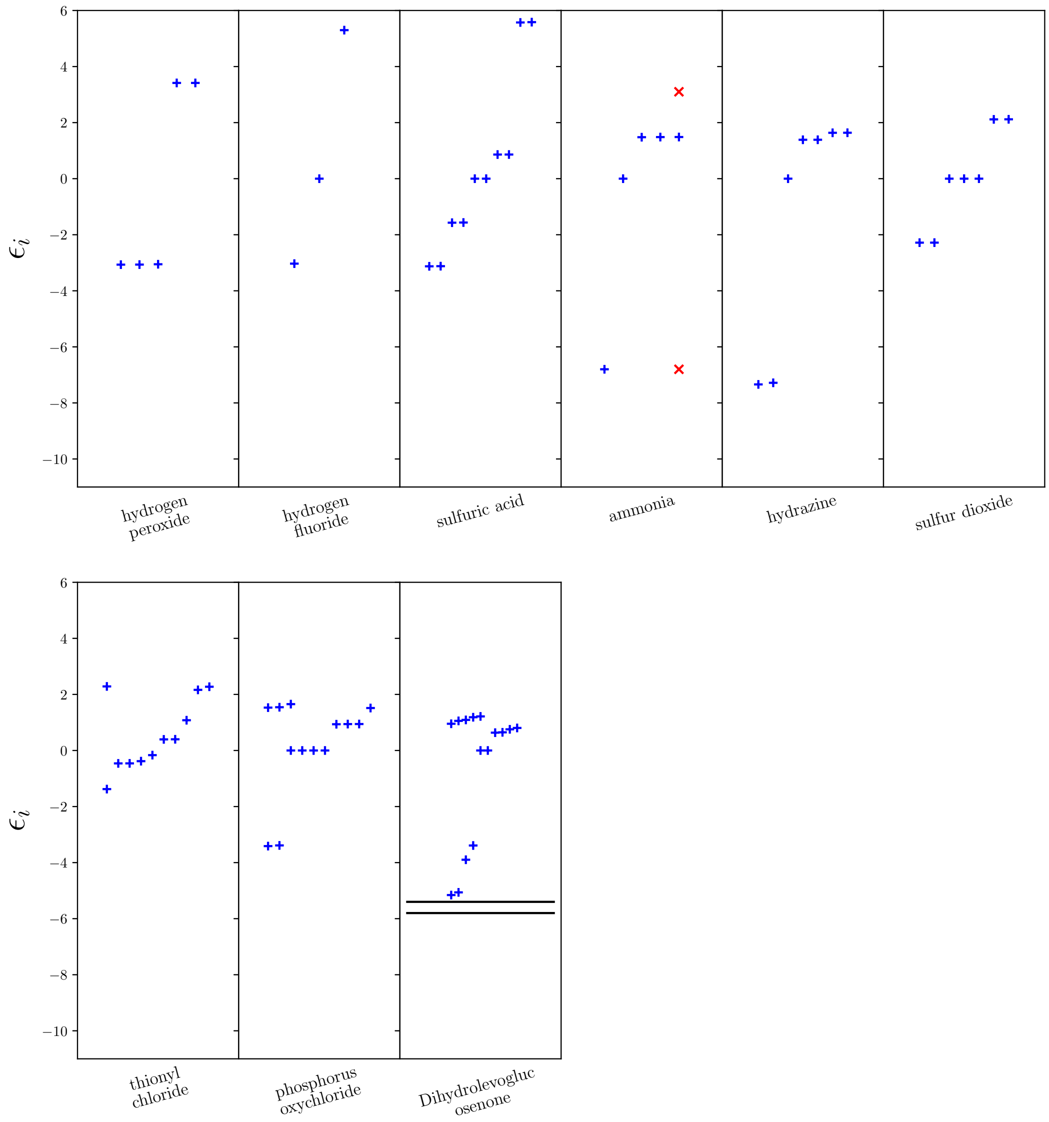

FGIP Plots For Solvents

Figure S3: FGIP for tetramethylsilane at 298K.

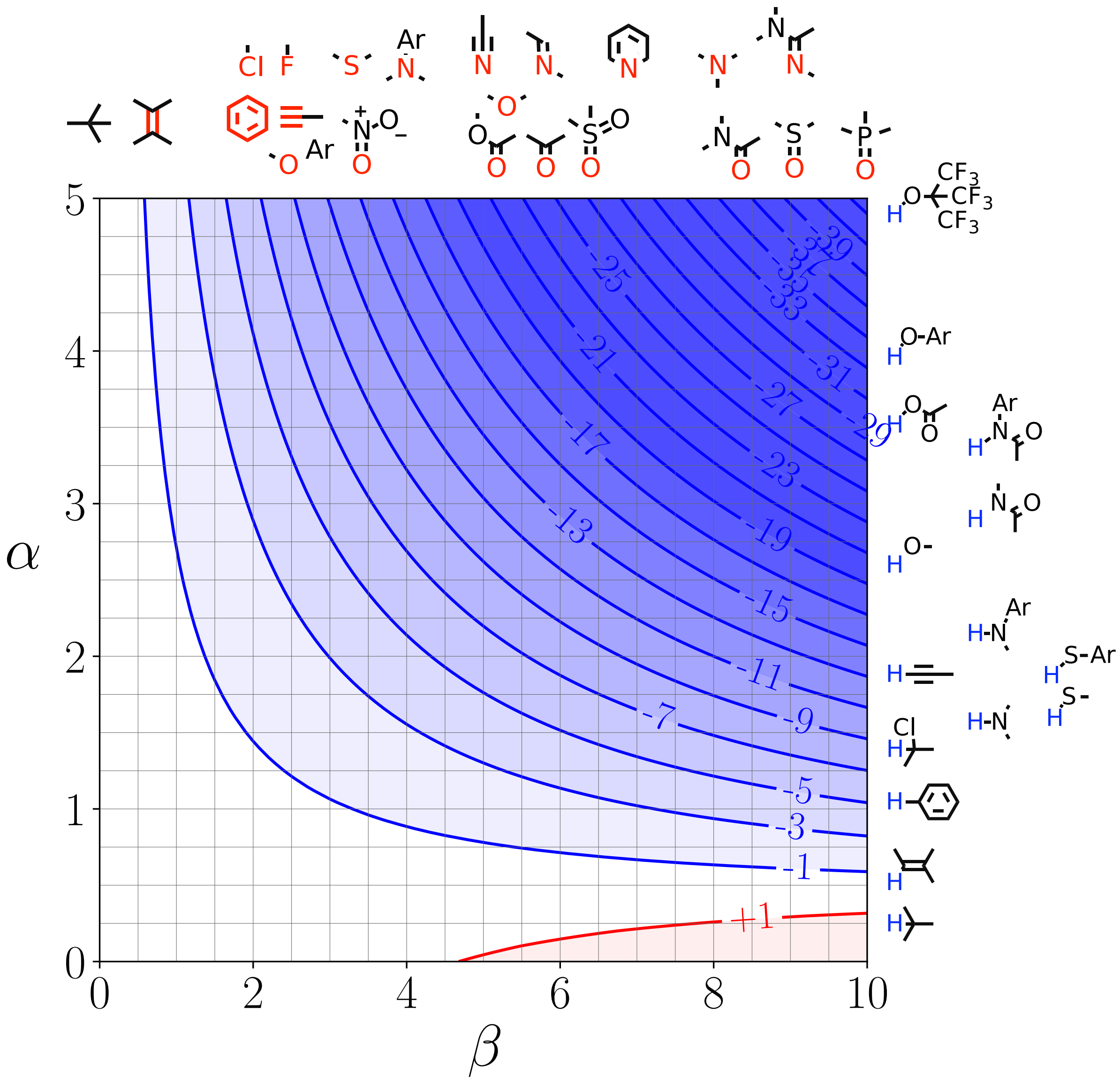

Figure S4: FGIP for n-pentane at 298K.

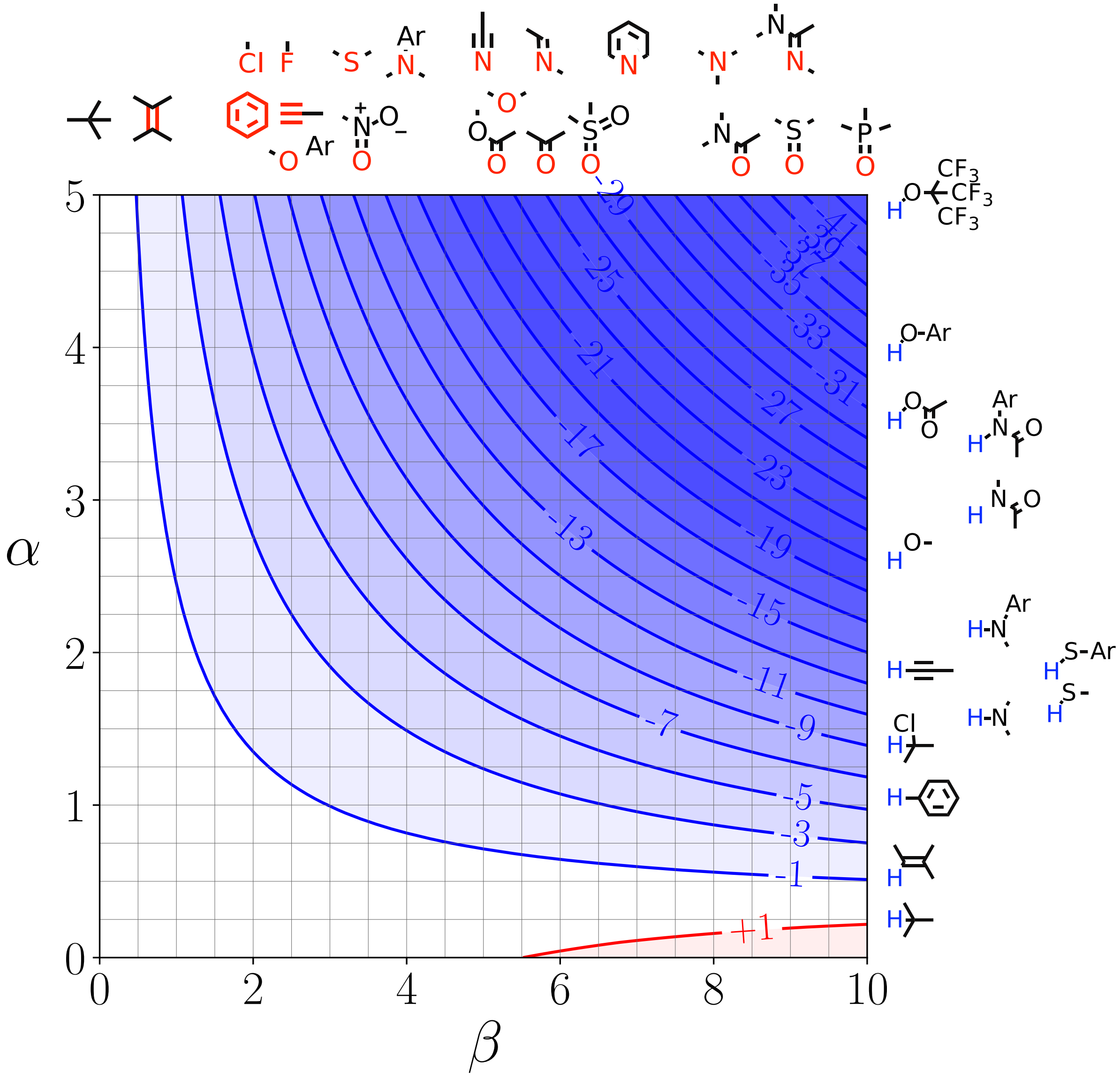

Figure S5: FGIP for 2-methylbutane at 298K.

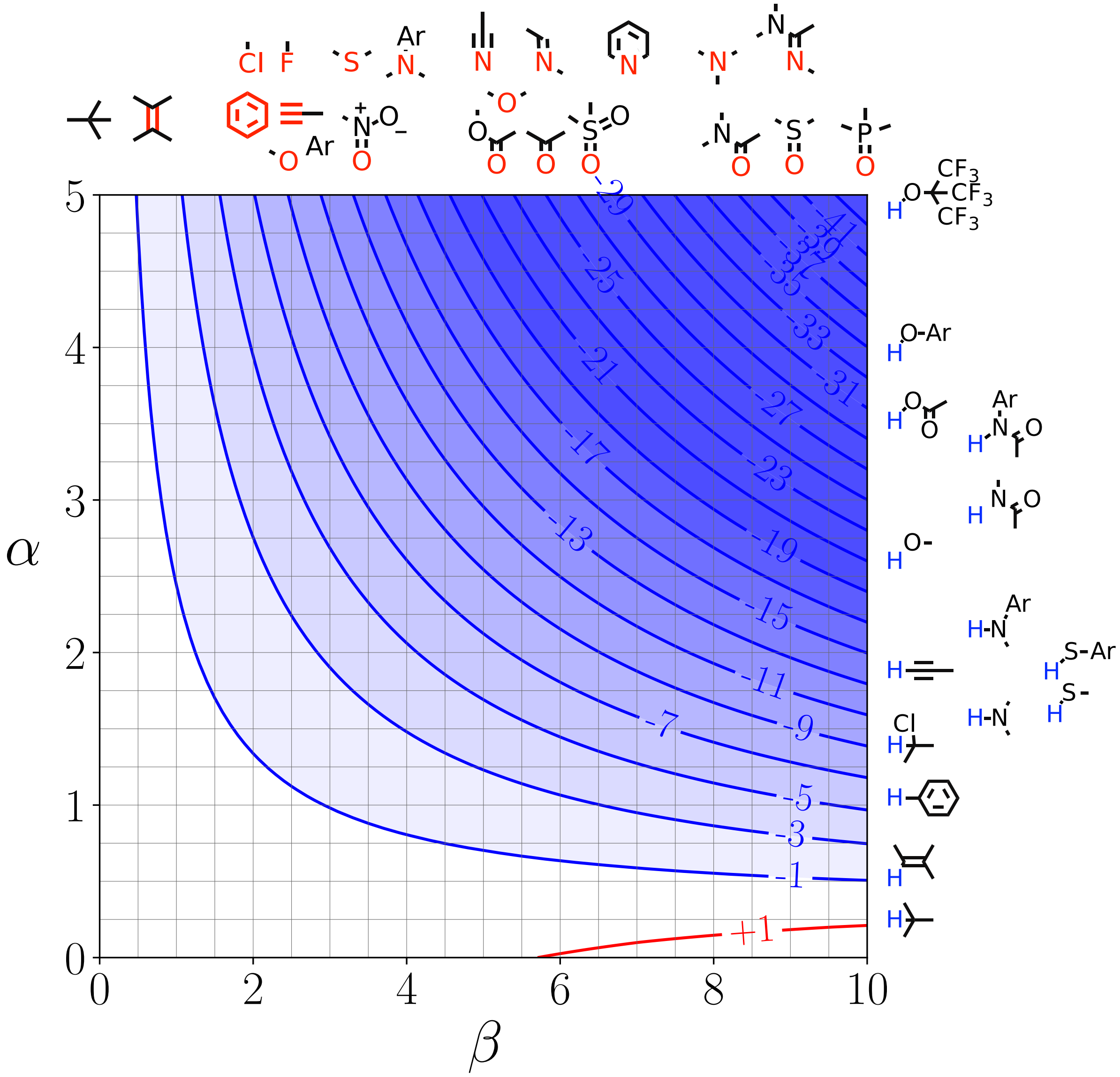

Figure S6: FGIP for n-hexane at 298K.

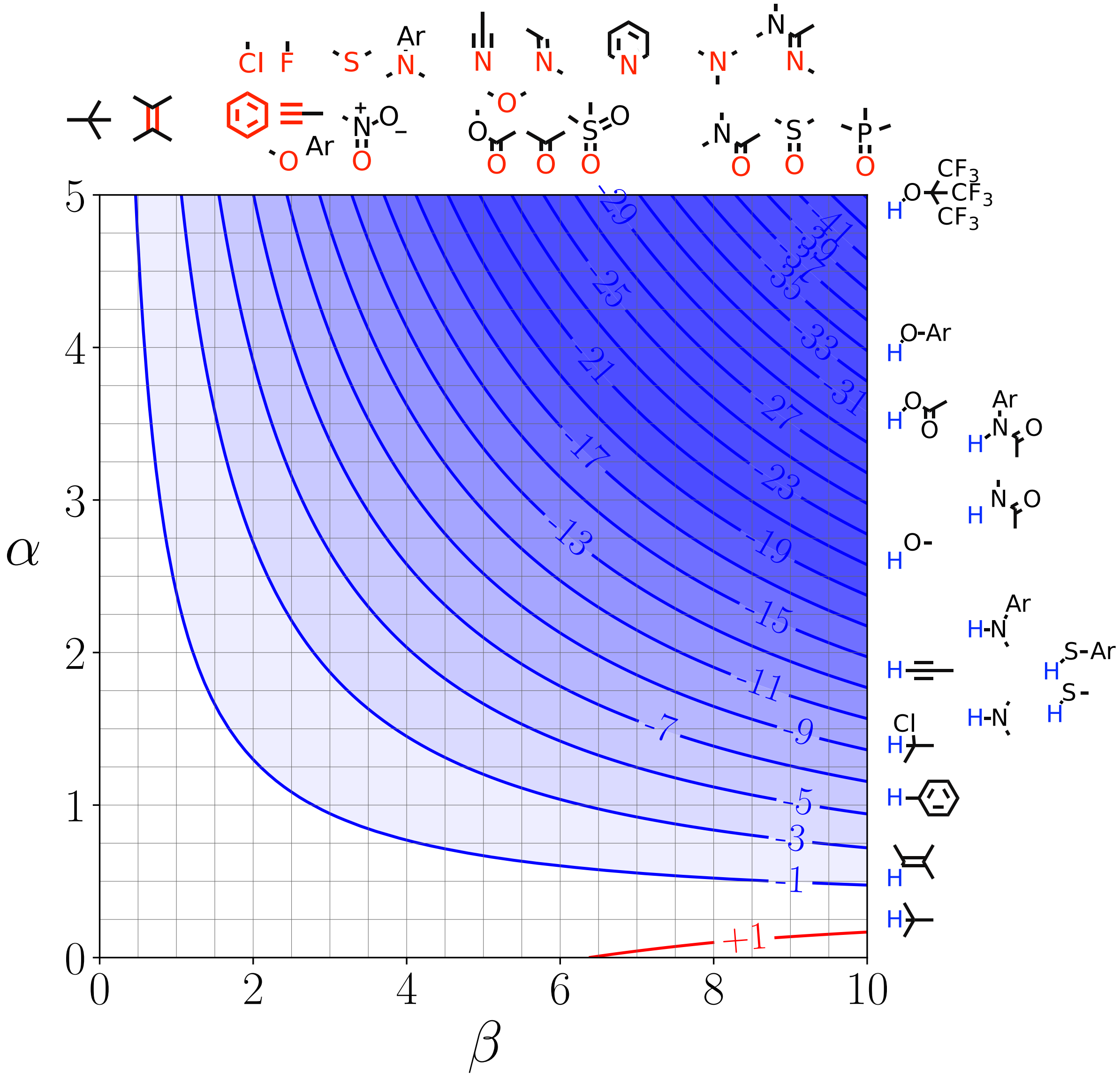

Figure S7: FGIP for cyclohexane at 298K.

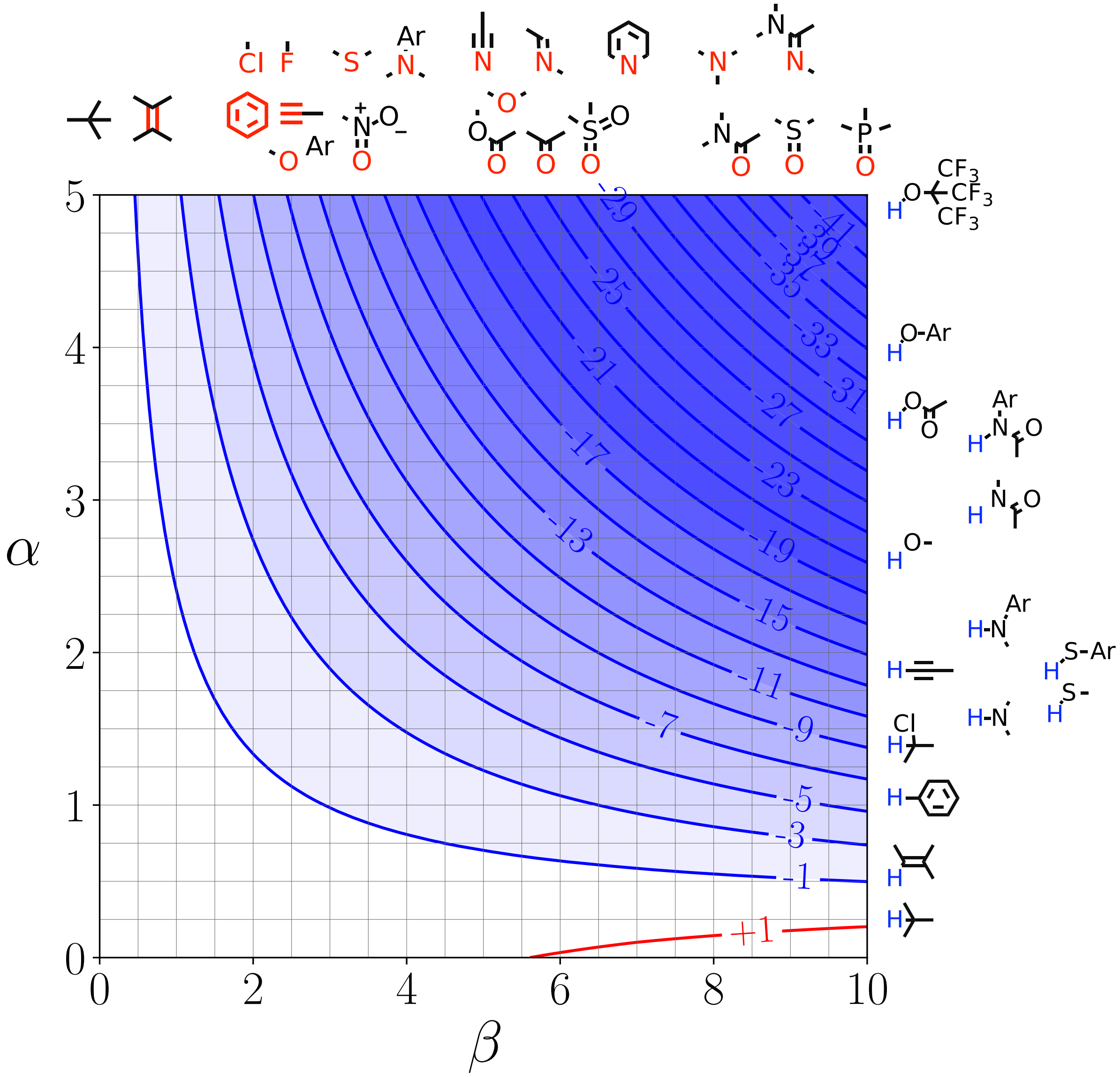

Figure S8: FGIP for n-heptane at 298K.

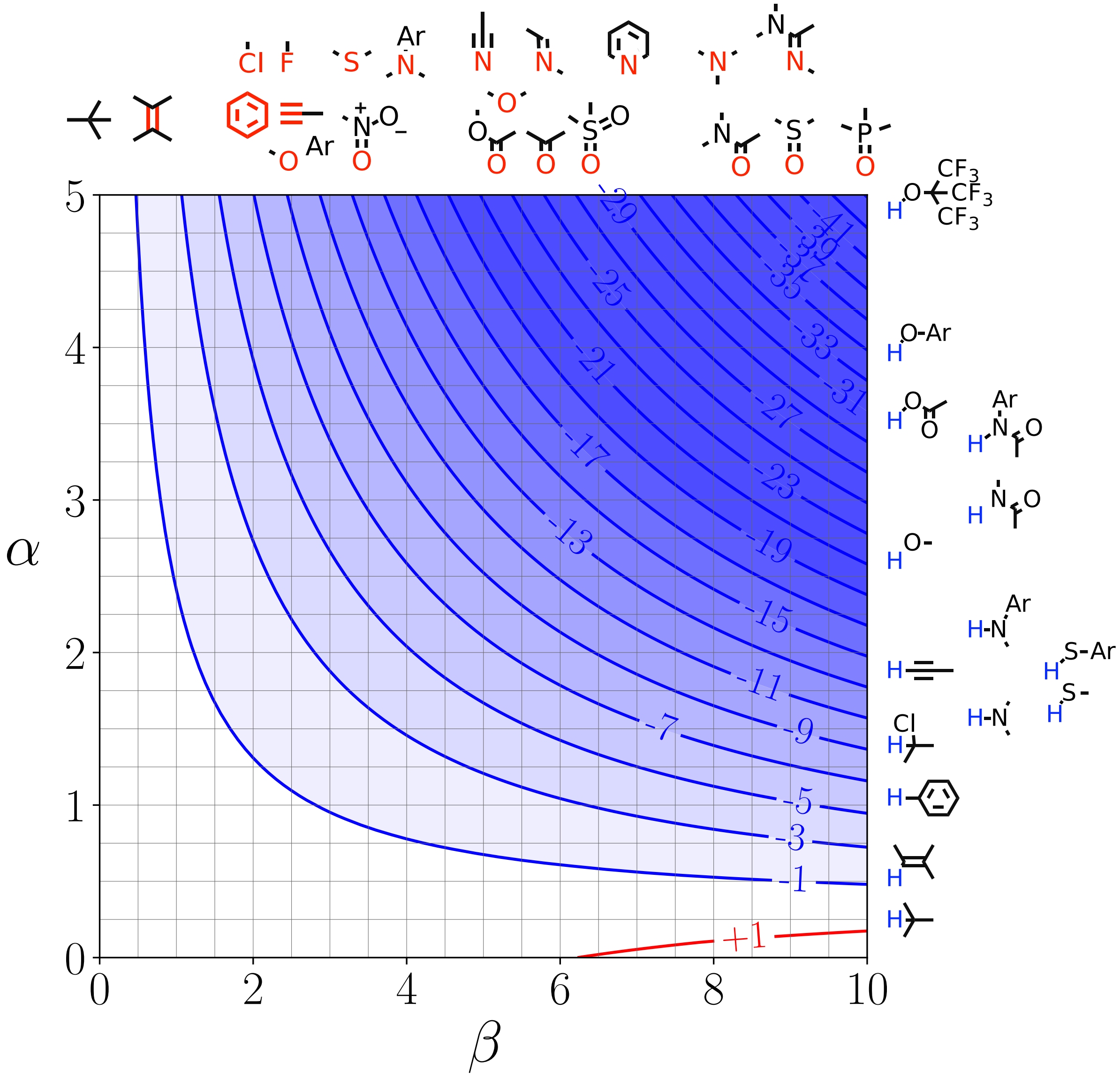

Figure S9: FGIP for n-octane at 298K.

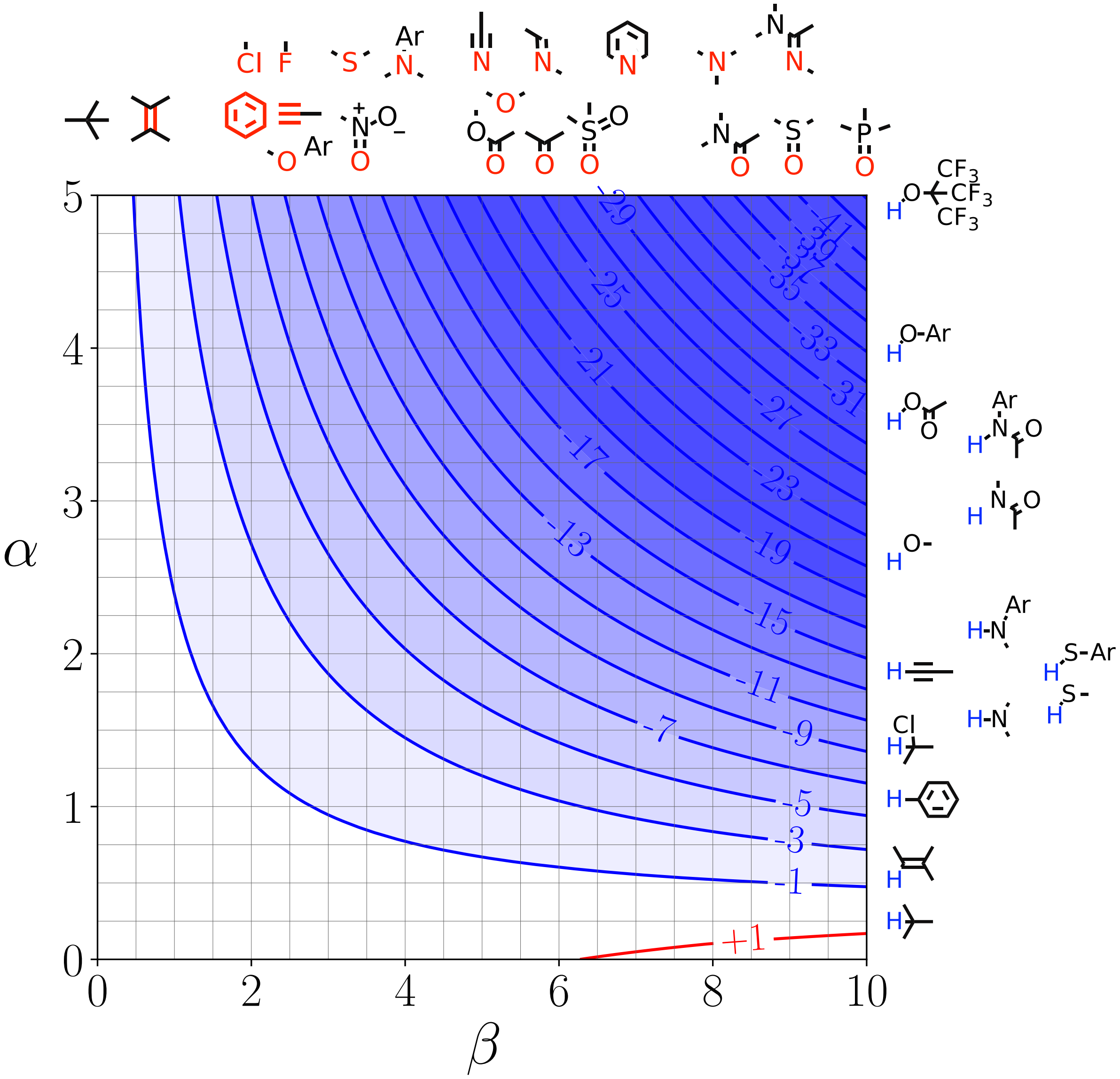

Figure S10: FGIP for 2,2,4-trimethylpentane at 298K.

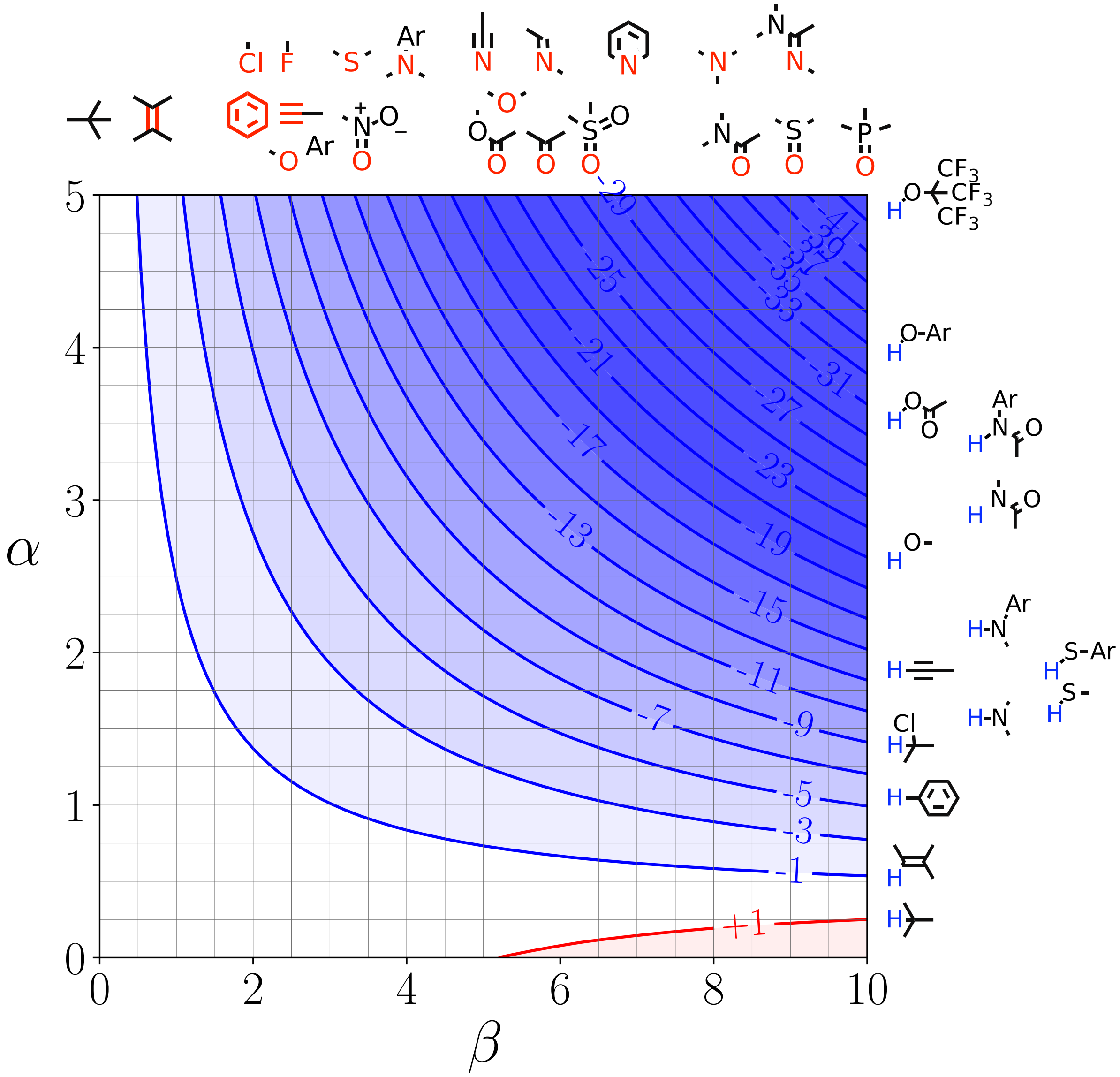

Figure S11: FGIP for n-decane at 298K.

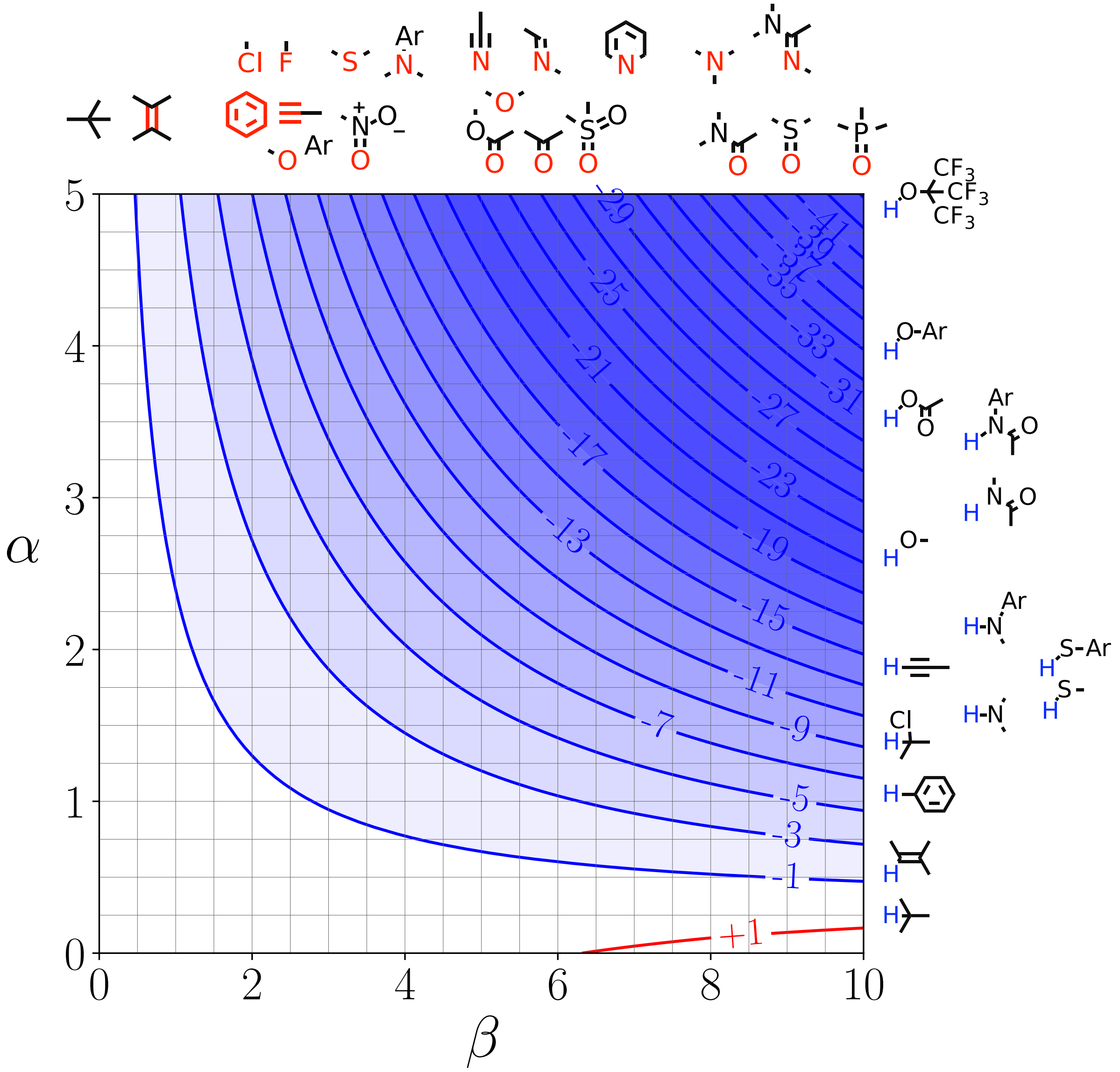

Figure S12: FGIP for n-dodecane at 298K.

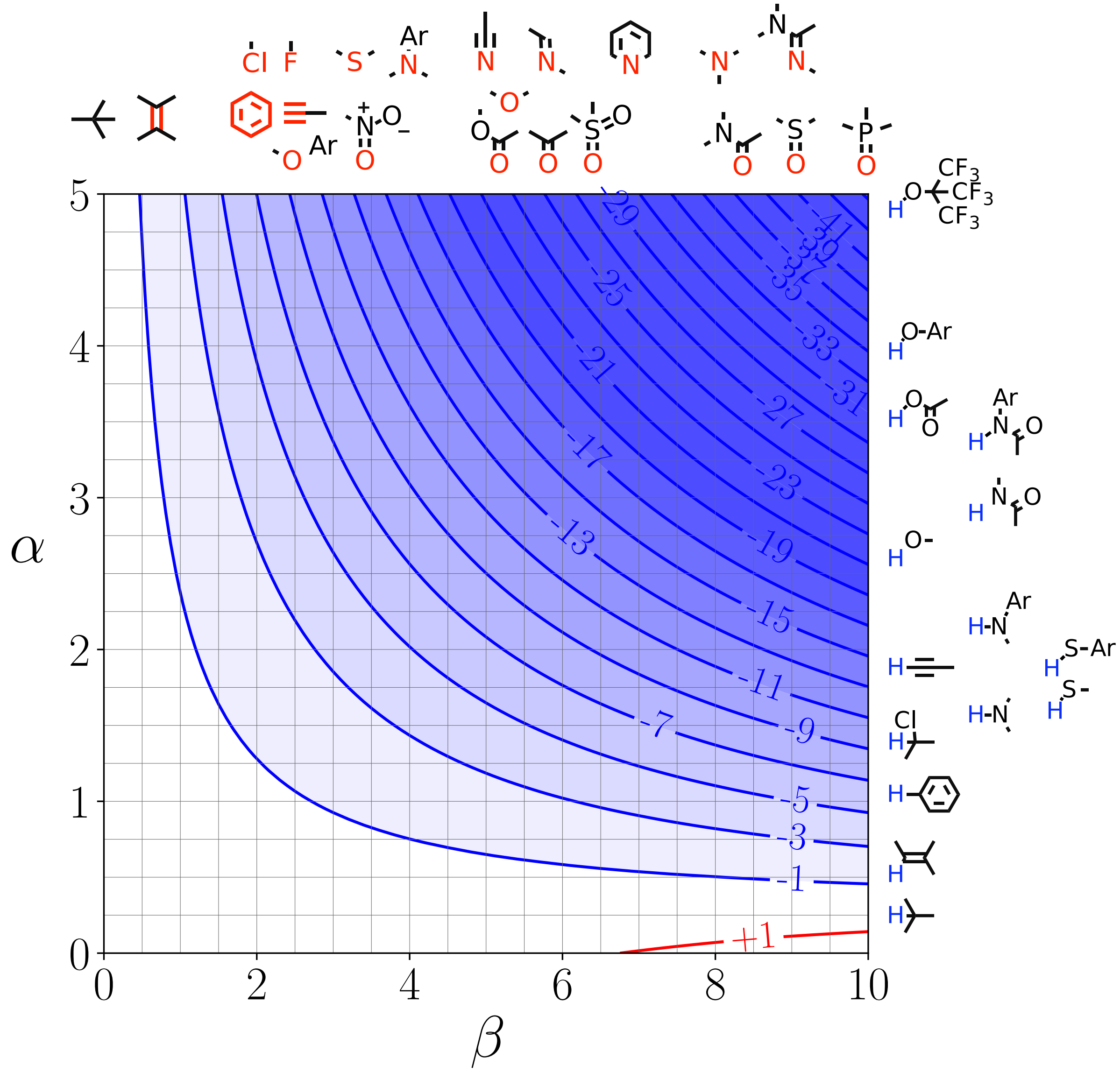

Figure S13: FGIP for n-hexadecane at 298K.

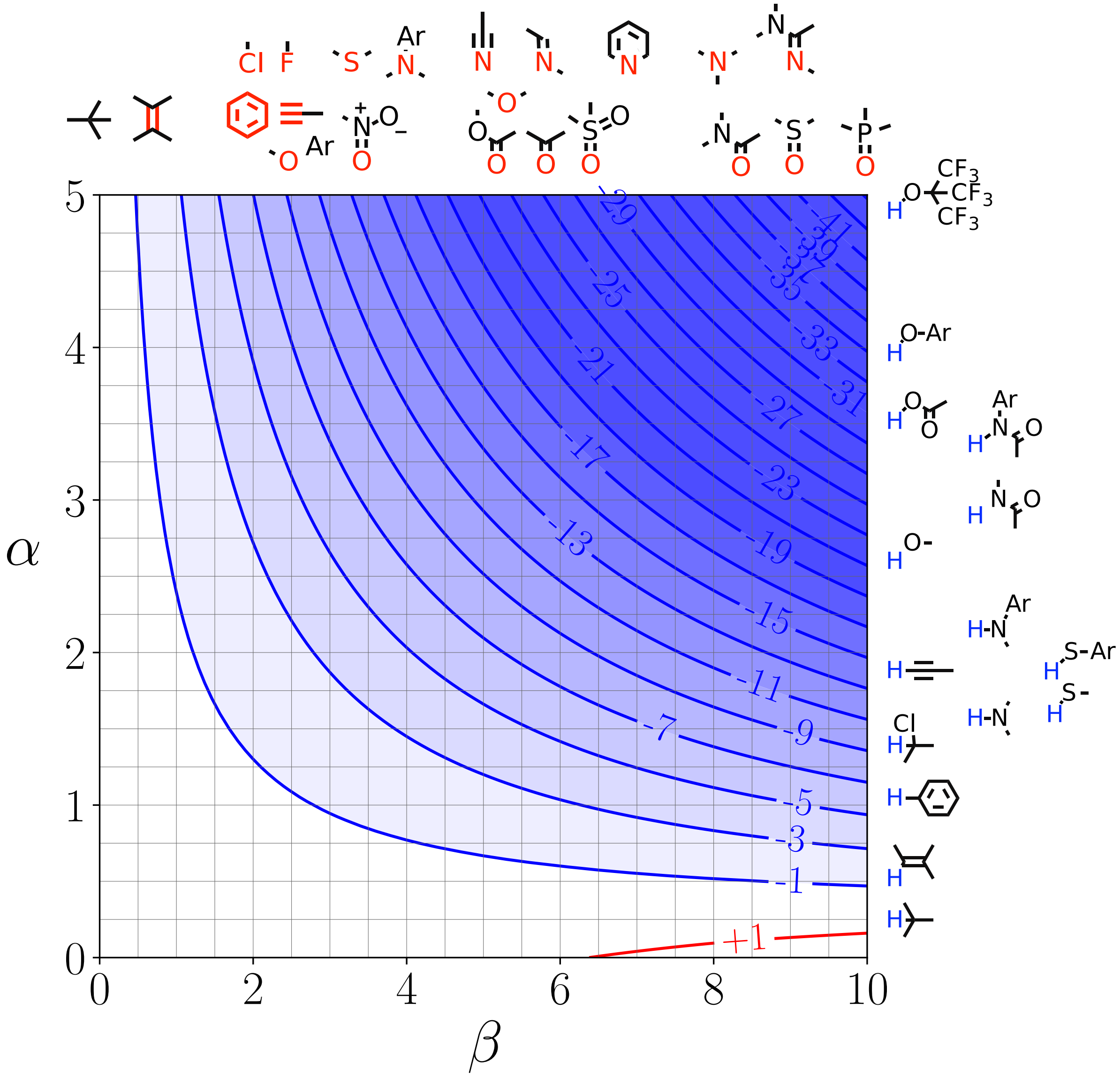

Figure S14: FGIP for benzene at 298K.

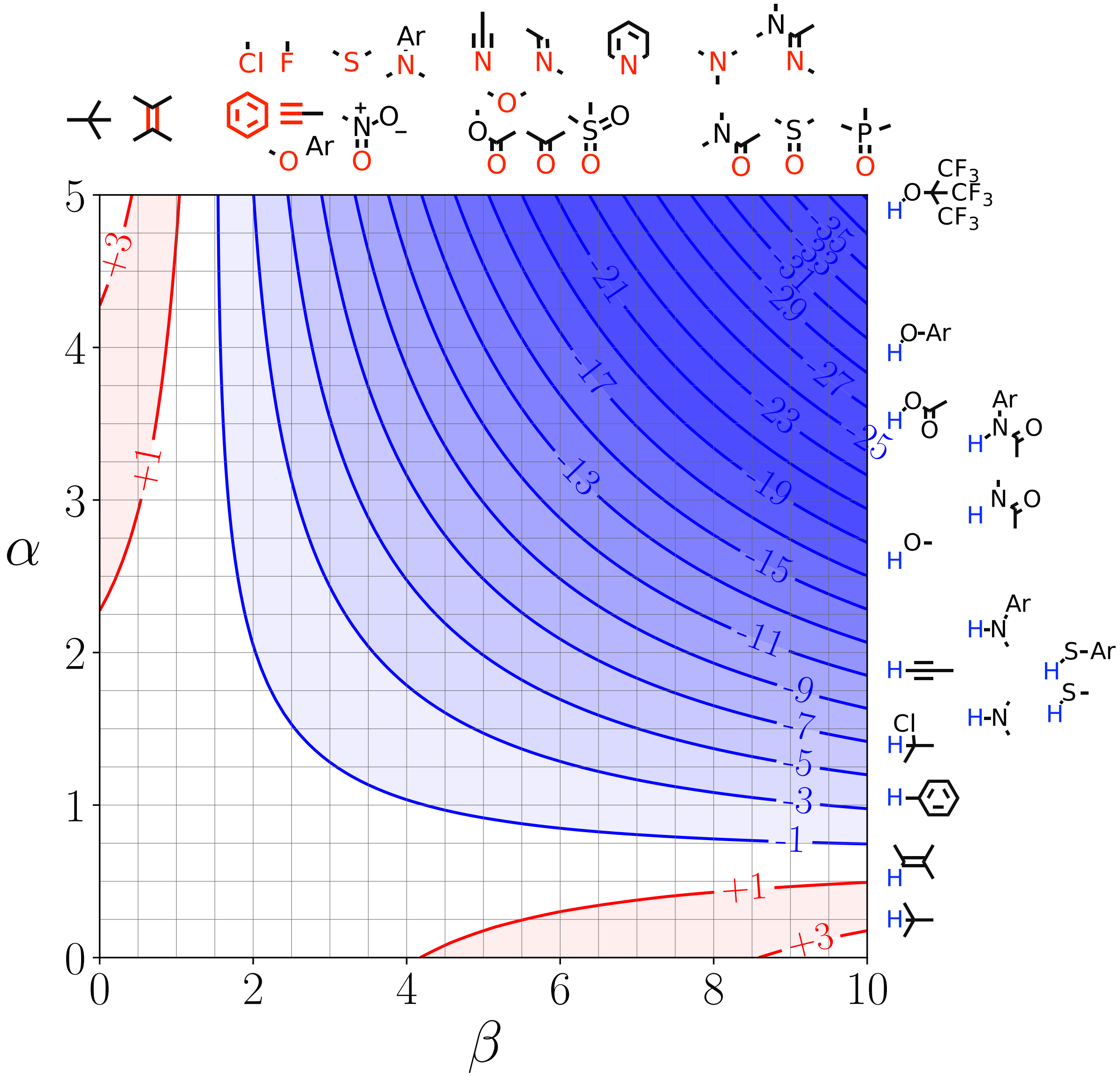

Figure S15: FGIP for toluene at 298K.

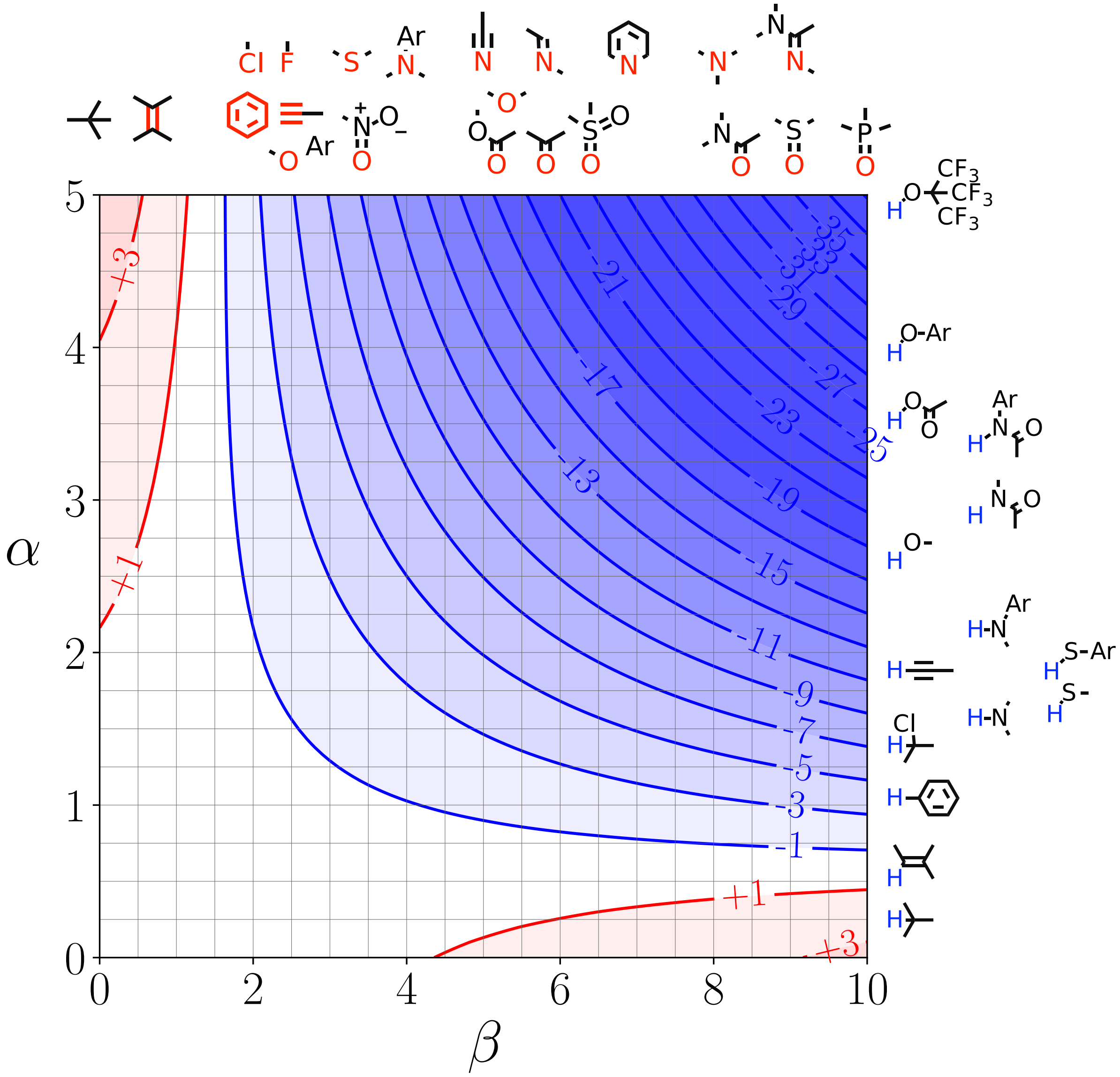

Figure S16: FGIP for ortho-xylene at 298K.

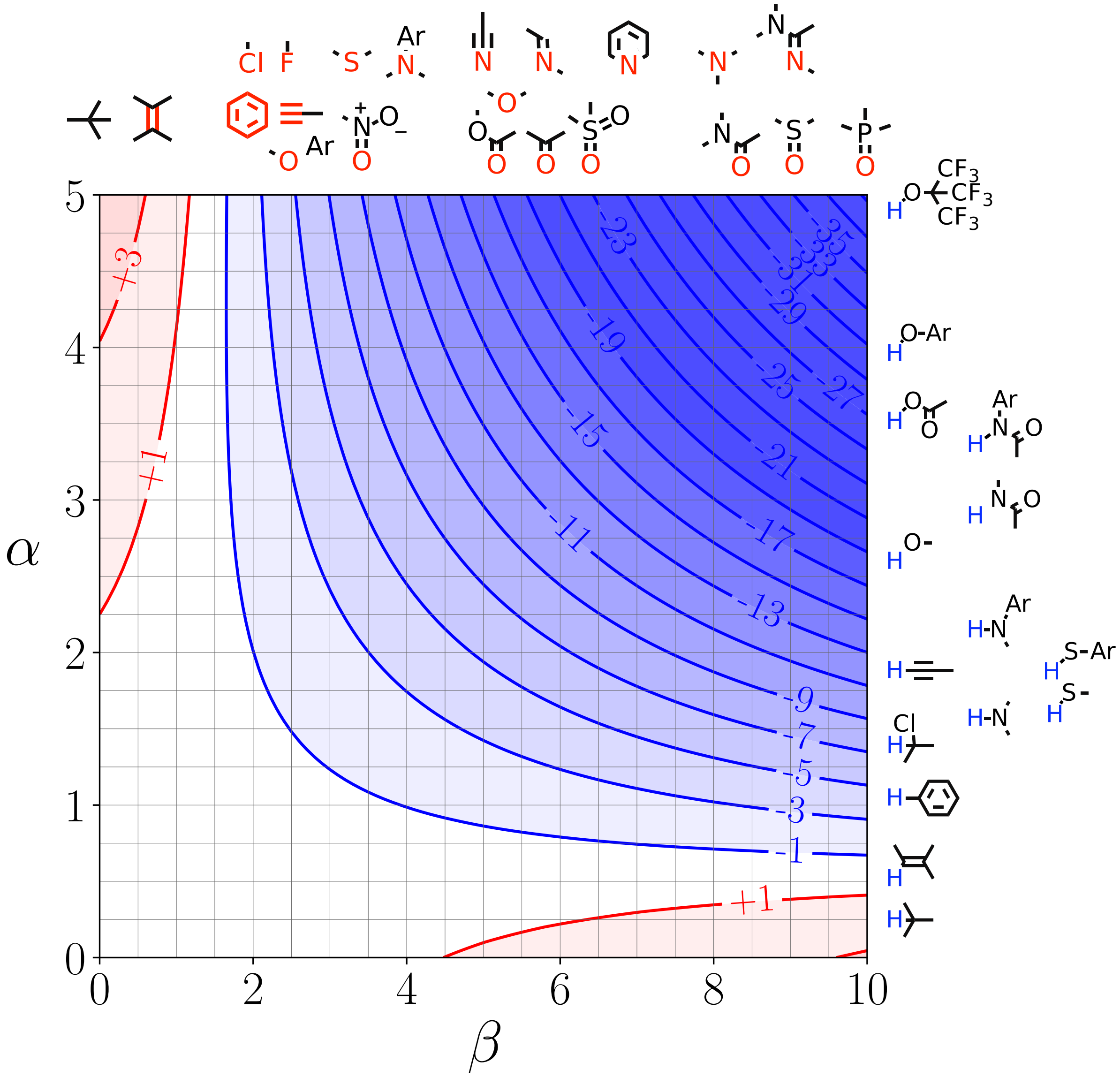

Figure S17: FGIP for meta-xylene at 298K.

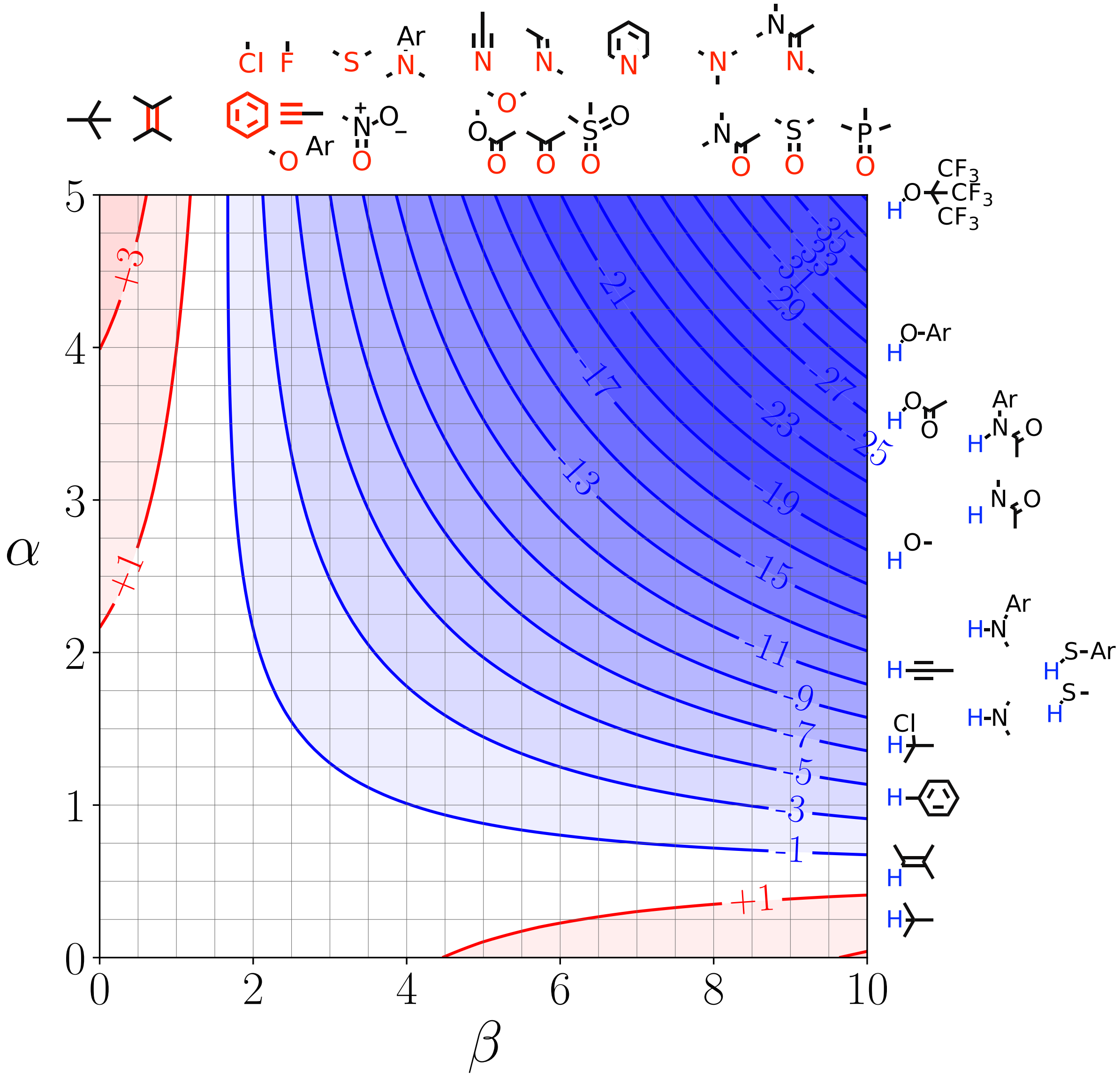

Figure S18: FGIP for para-xylene at 298K.

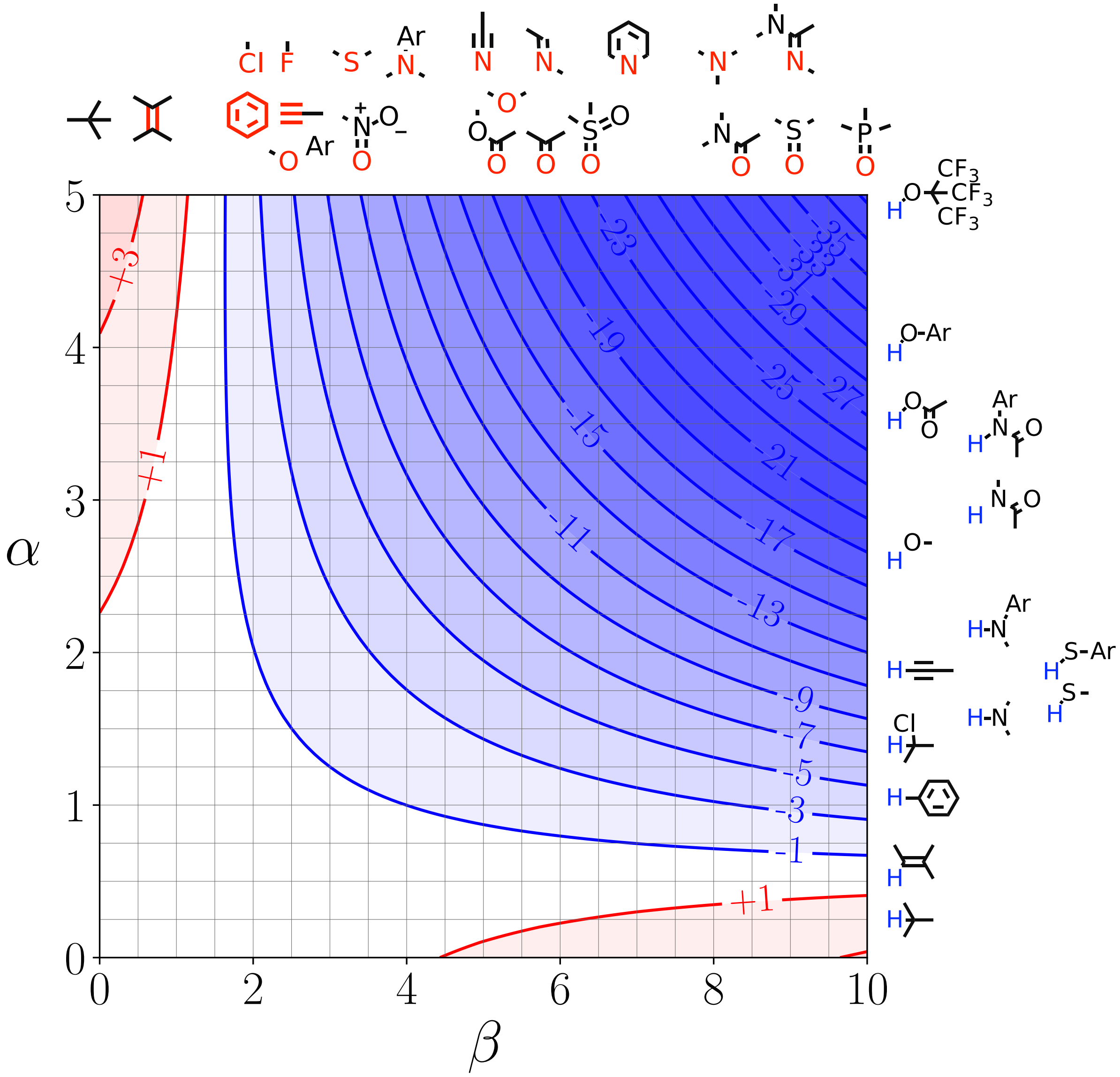

Figure S19: FGIP for ethylbenzene at 298K.

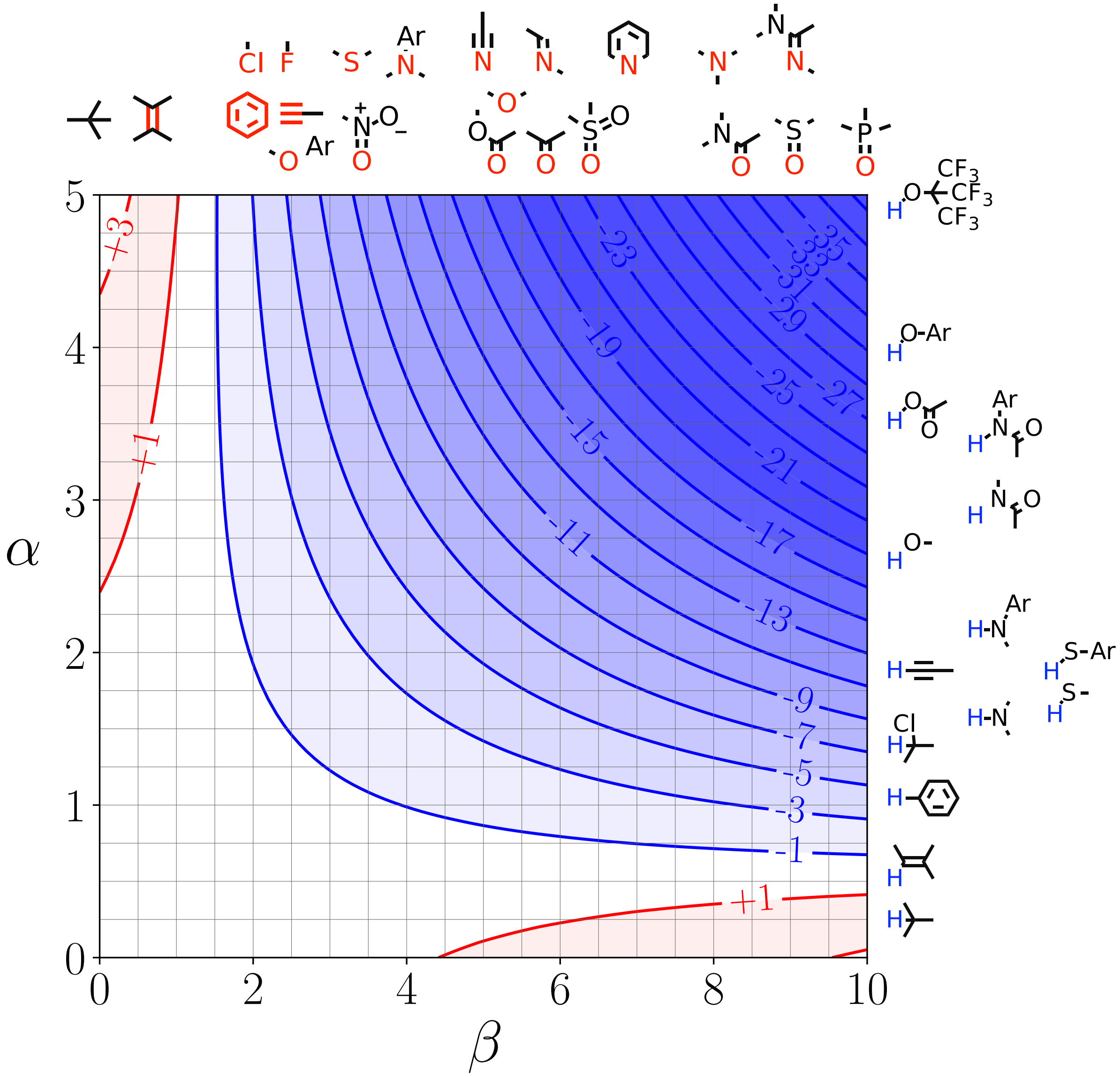

Figure S20: FGIP for isopropylbenzene at 298K.

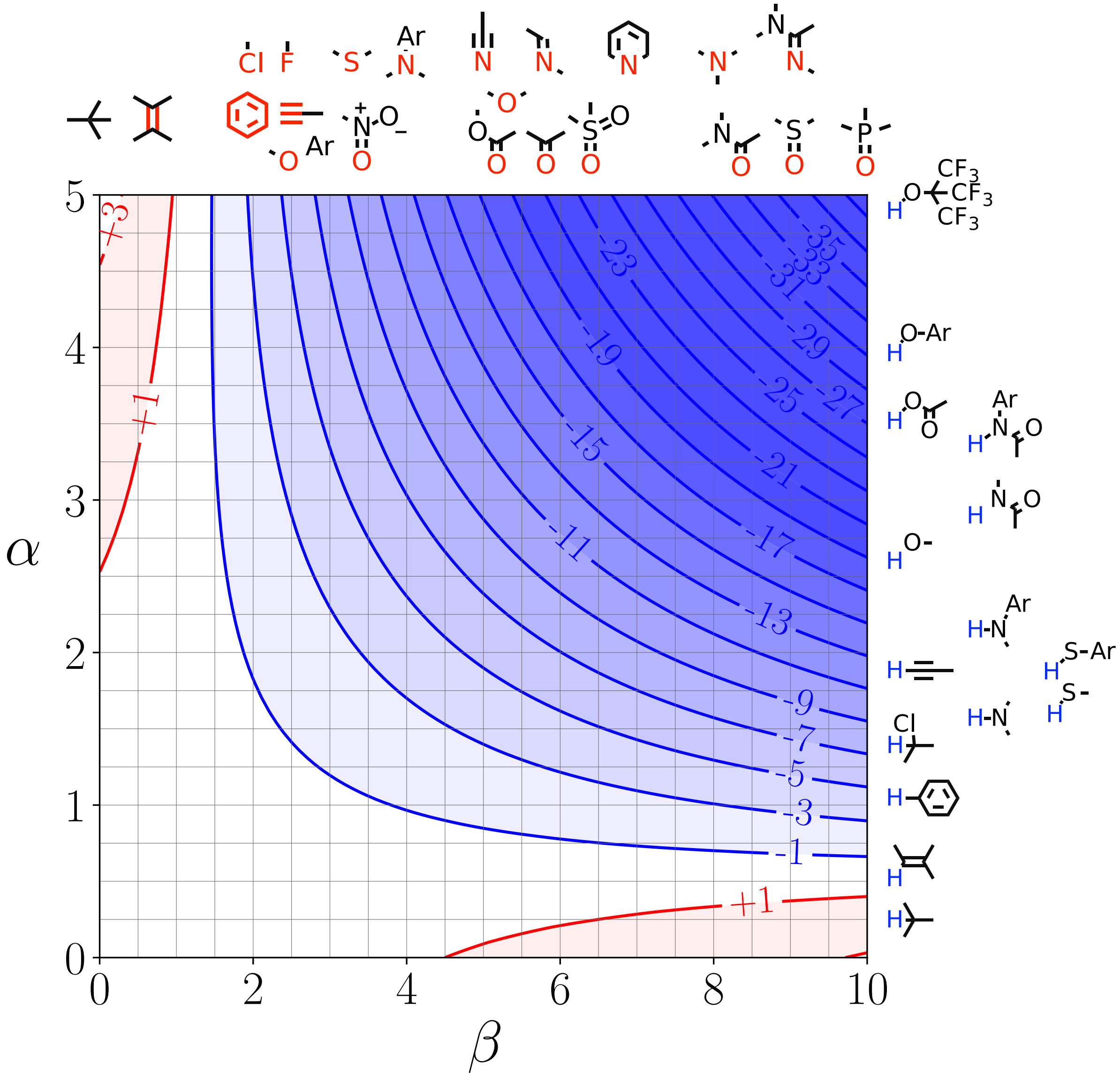

Figure S21: FGIP for 1,3,5-trimethylbenzene at 298K.

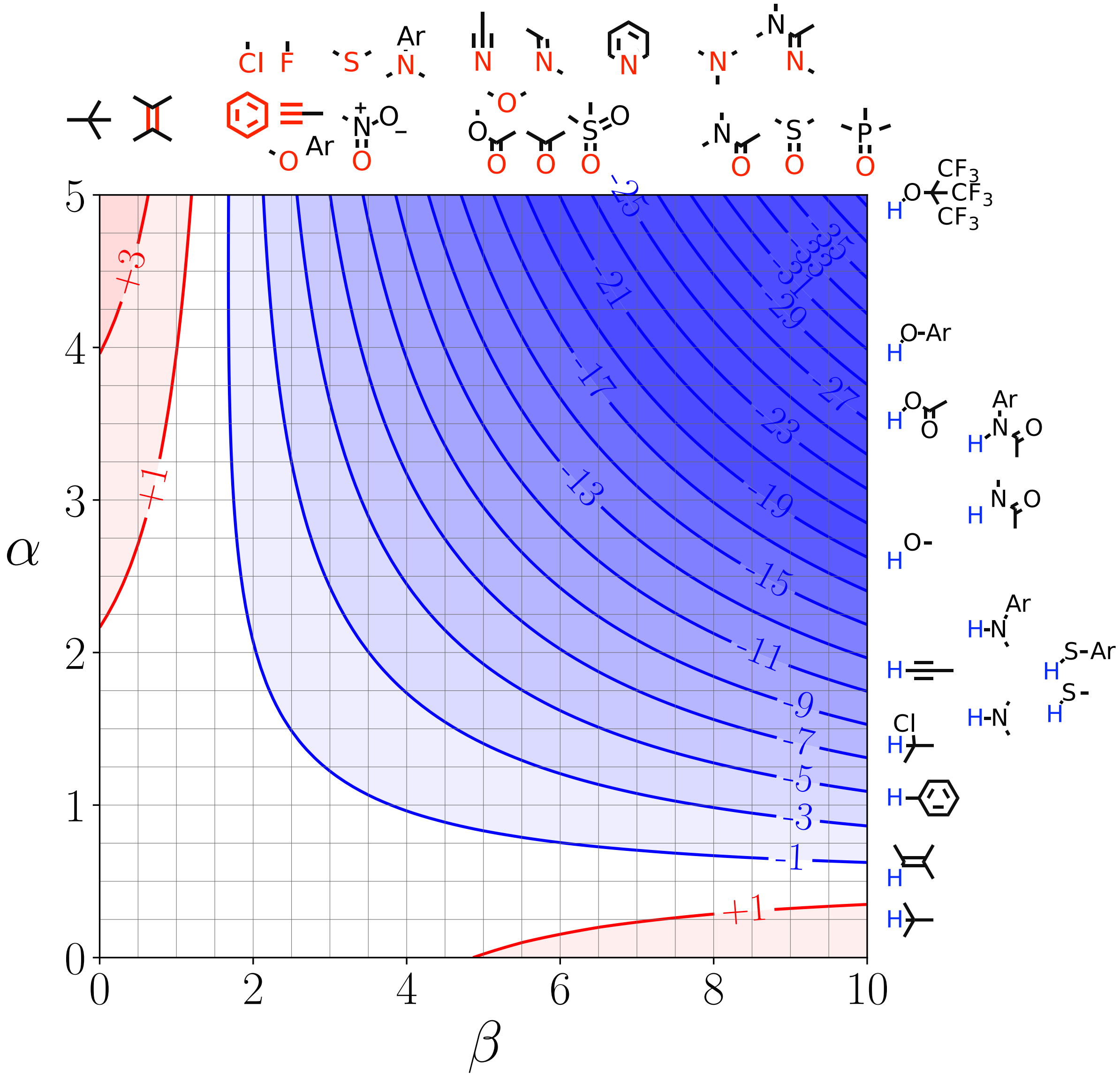

Figure S22: FGIP for styrene at 298K.

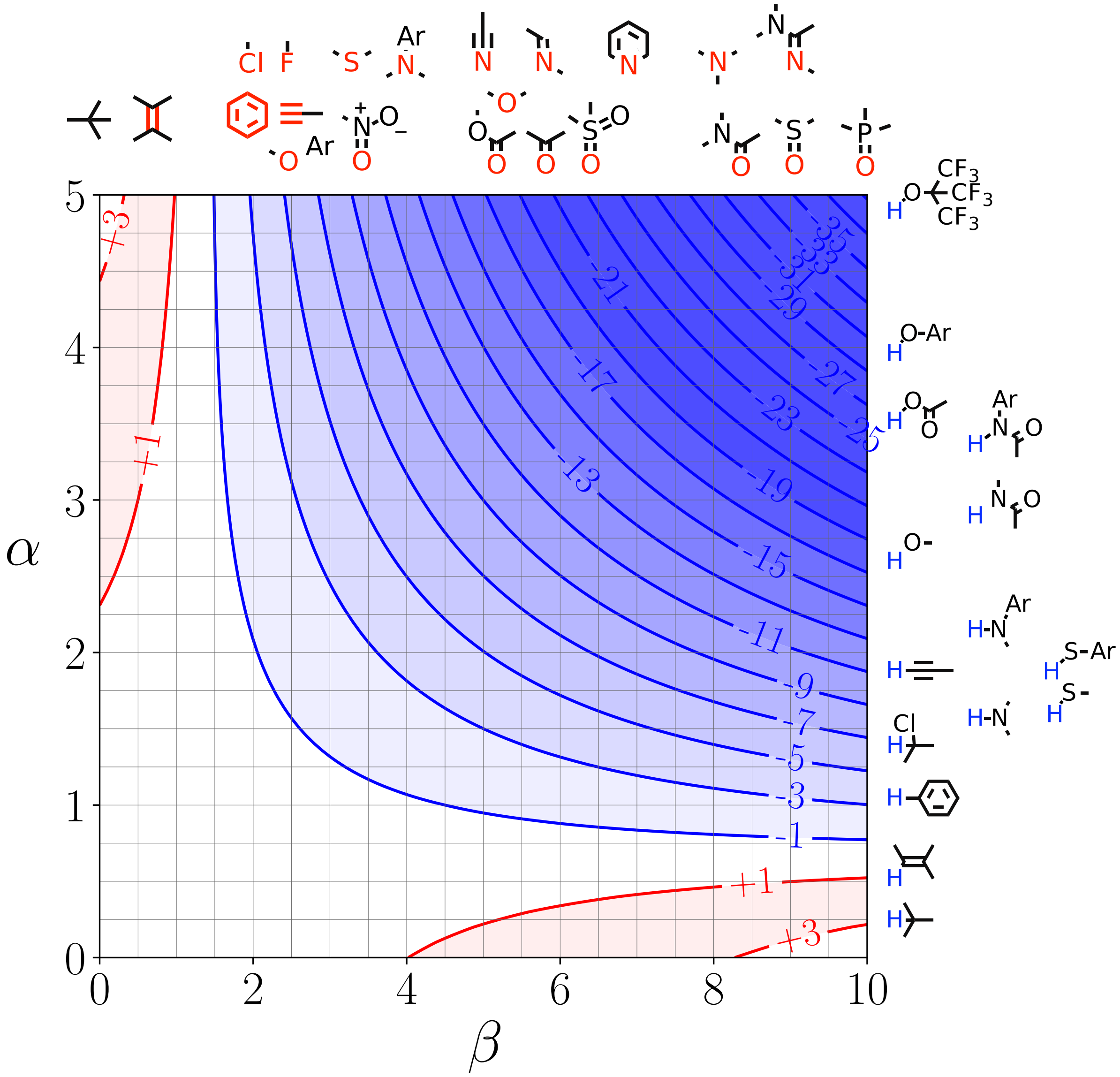

Figure S23: FGIP for 1,2,3,4-tetrahydronaphthalene at 298K.

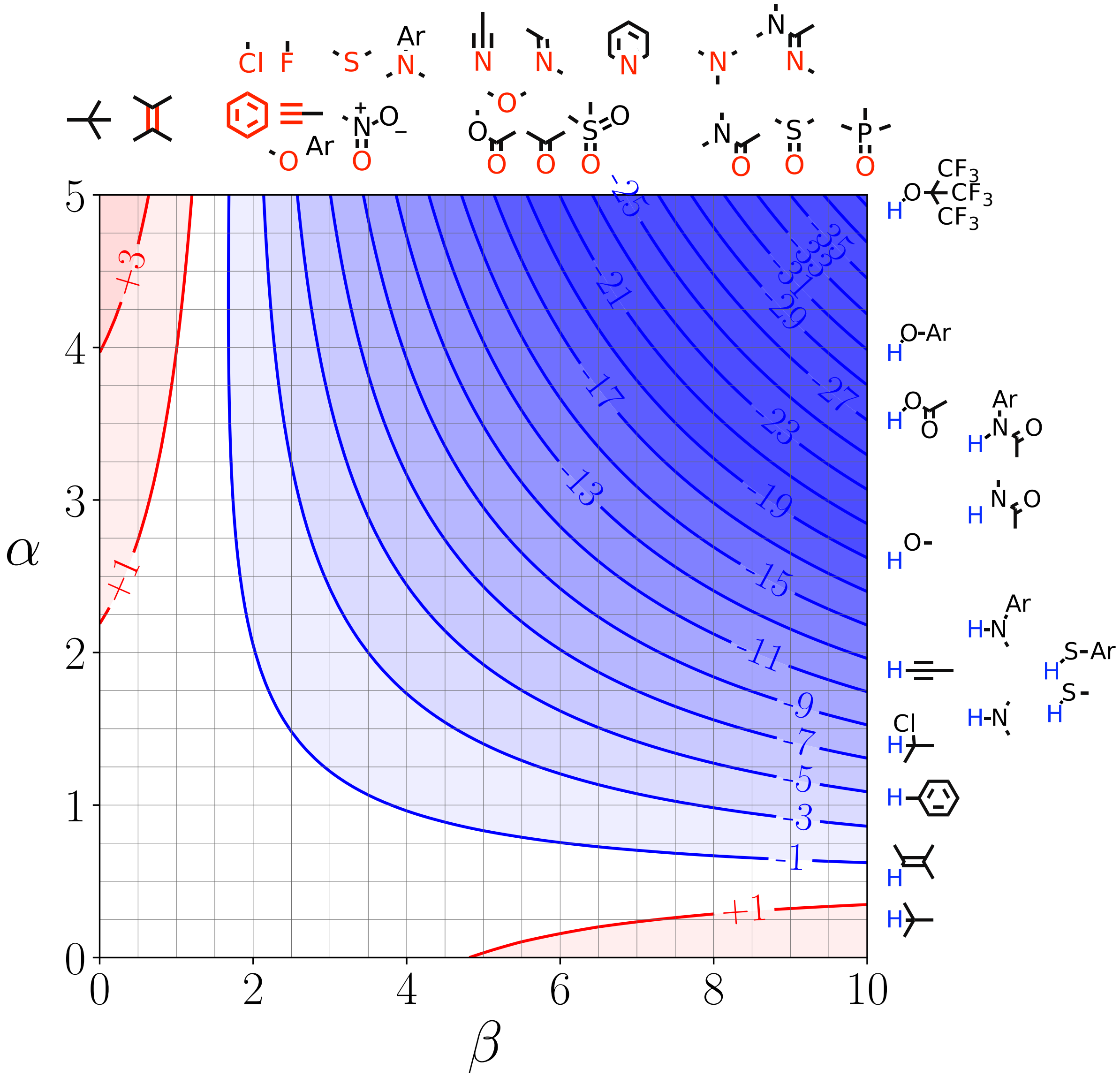

Figure S24: FGIP for cis-decalin at 298K.

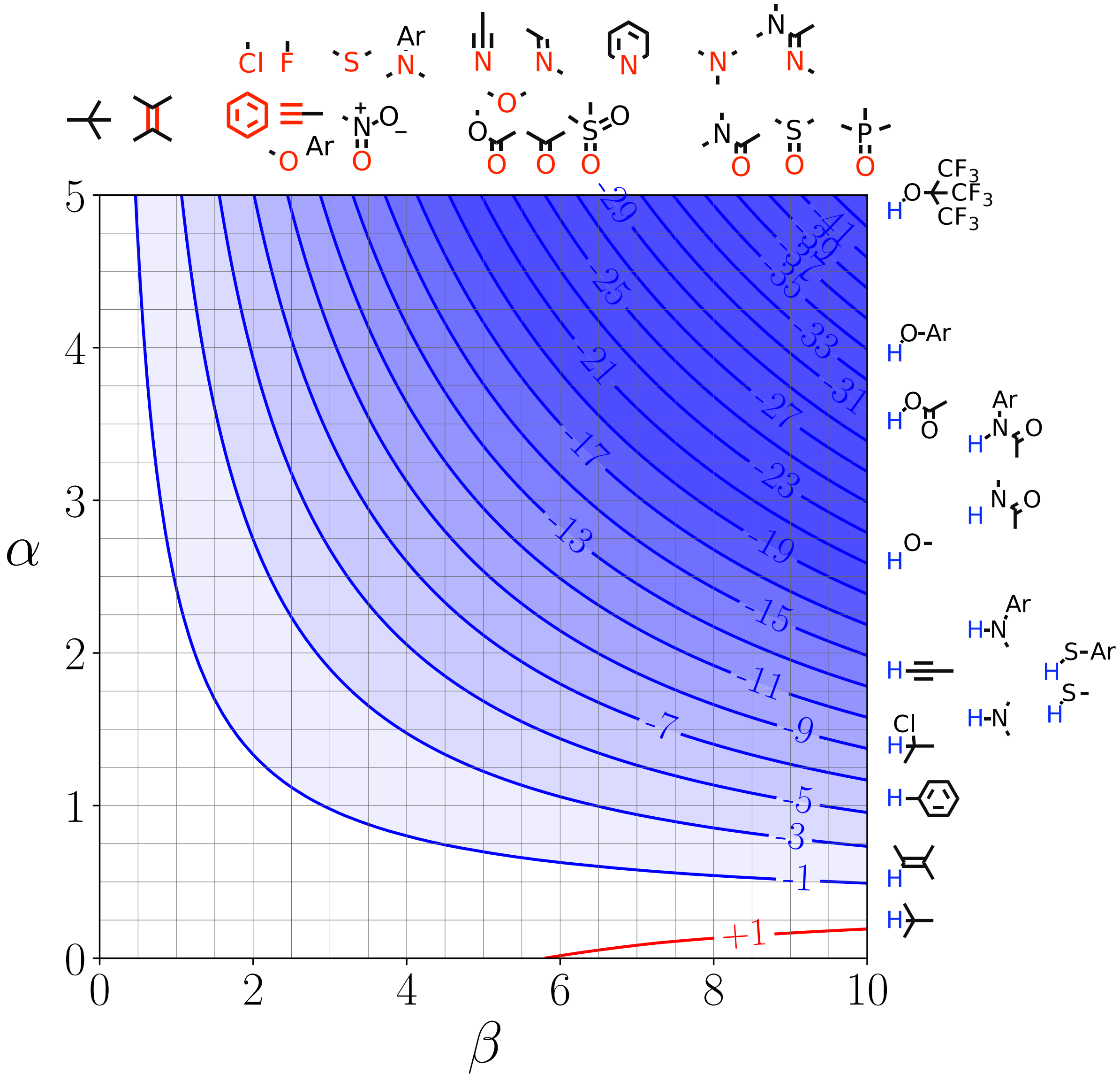

Figure S25: FGIP for water at 298K.

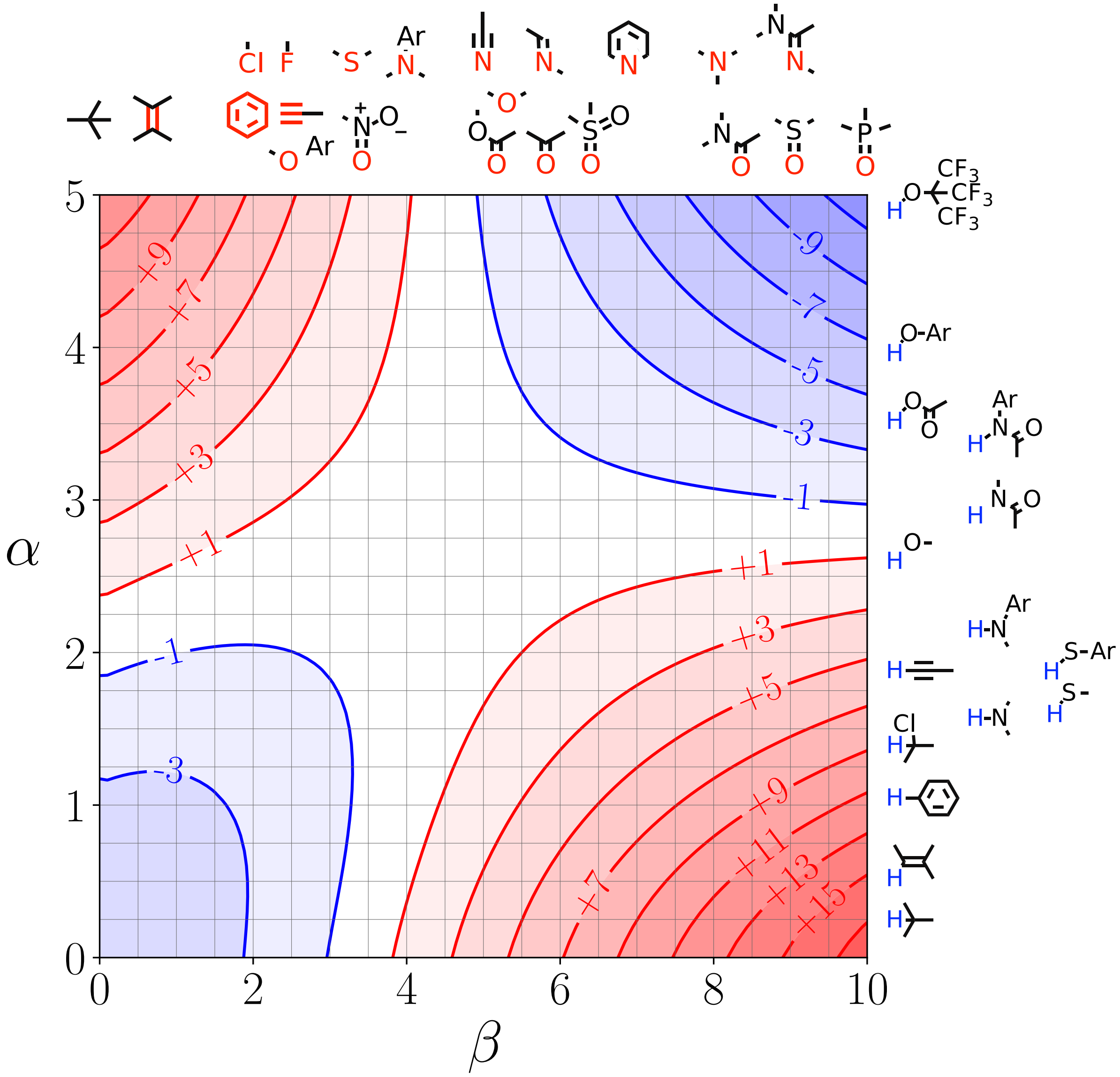

Figure S26: FGIP for methanol at 298K.

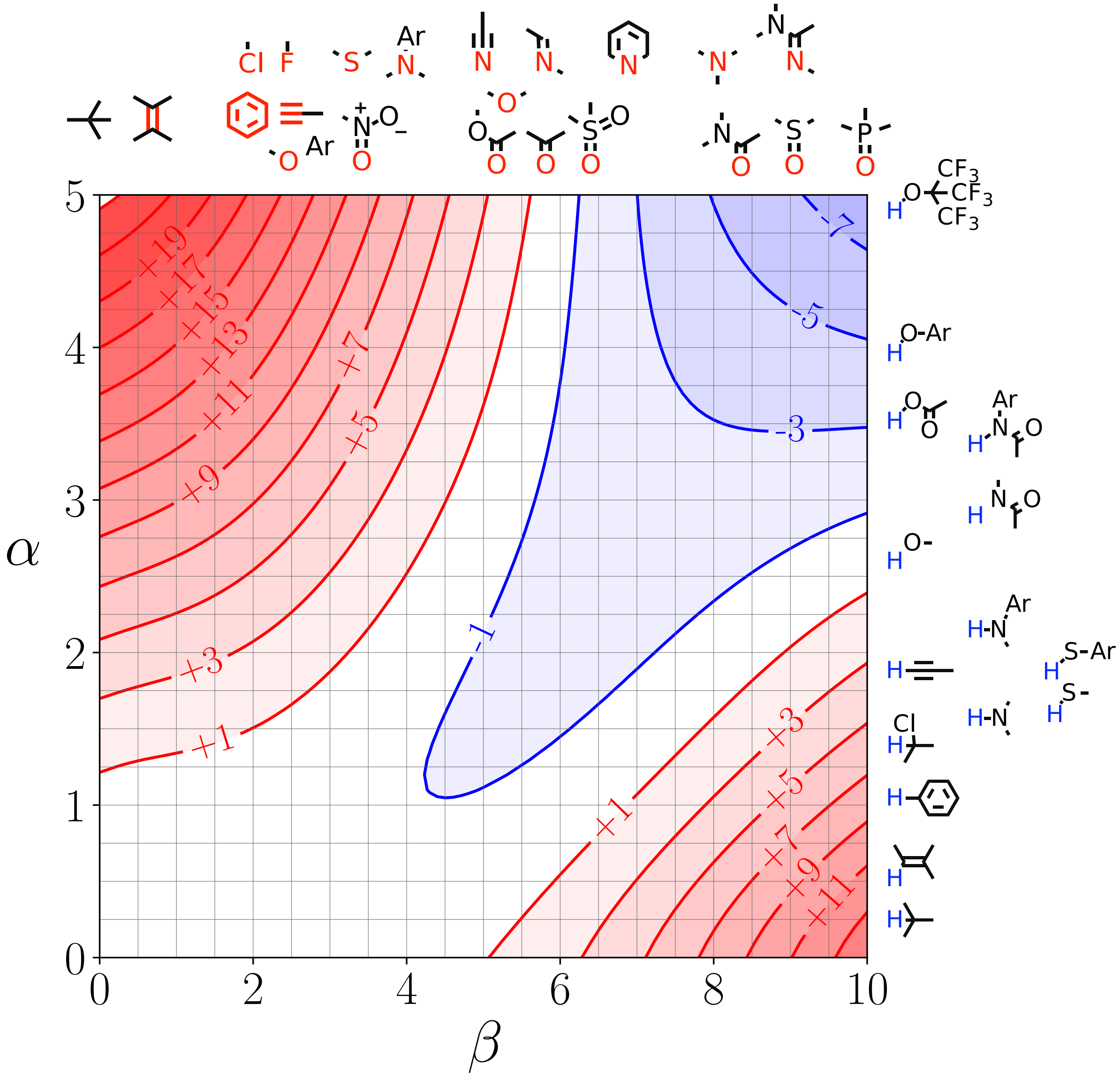

Figure S27: FGIP for ethanol at 298K.

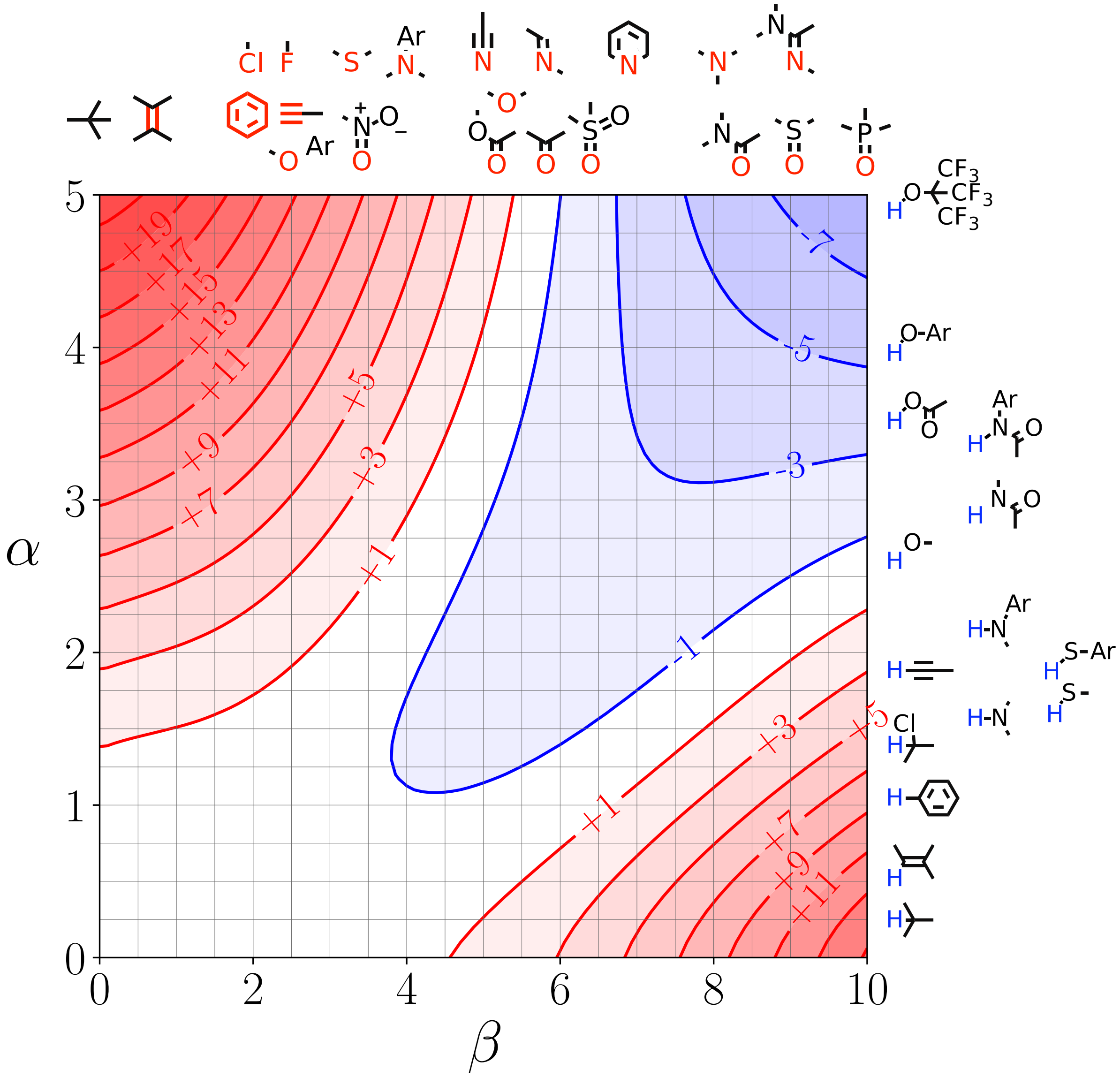

Figure S28: FGIP for 1-propanol at 298K.

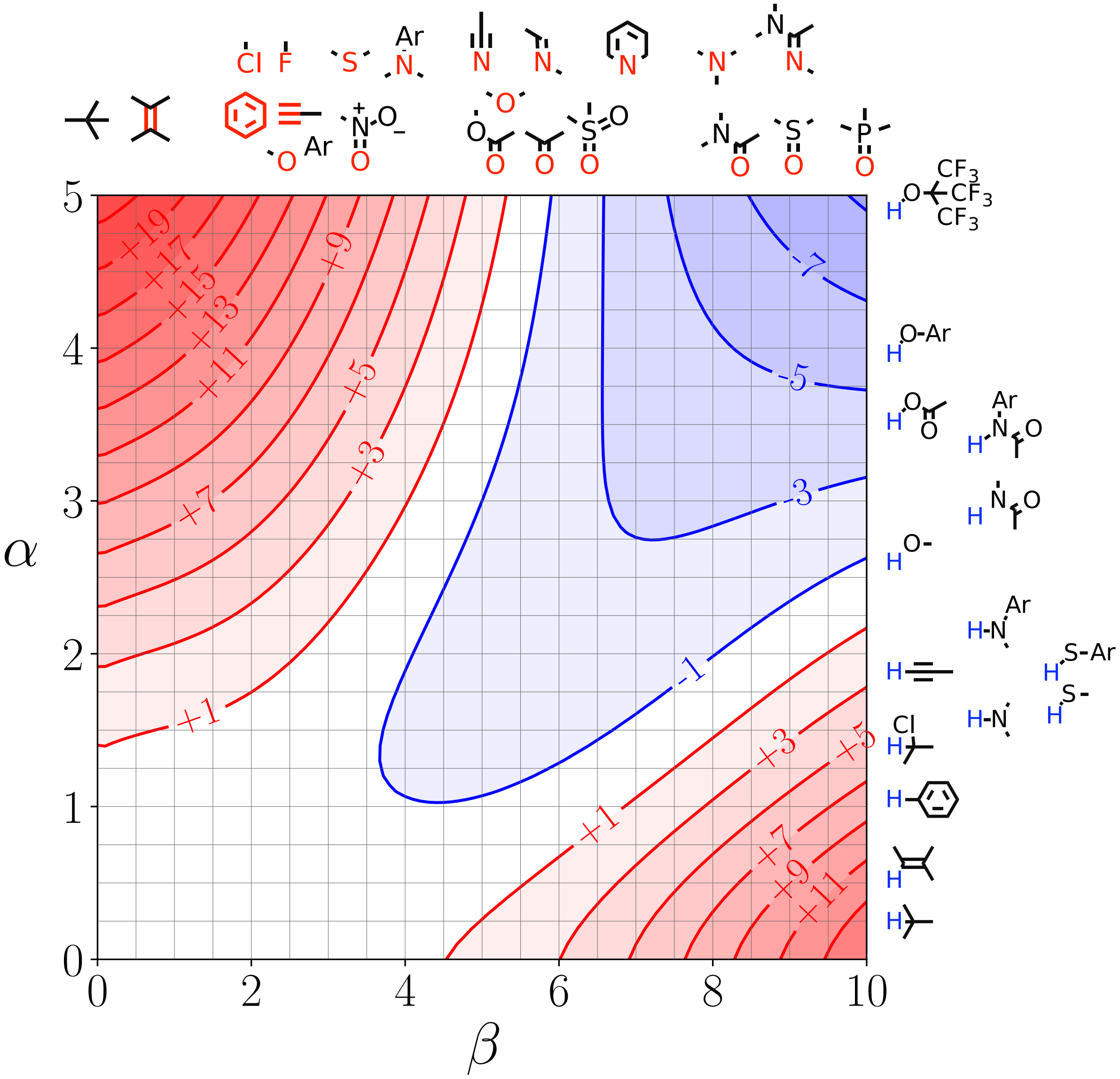

Figure S29: FGIP for 2-propanol at 298K.

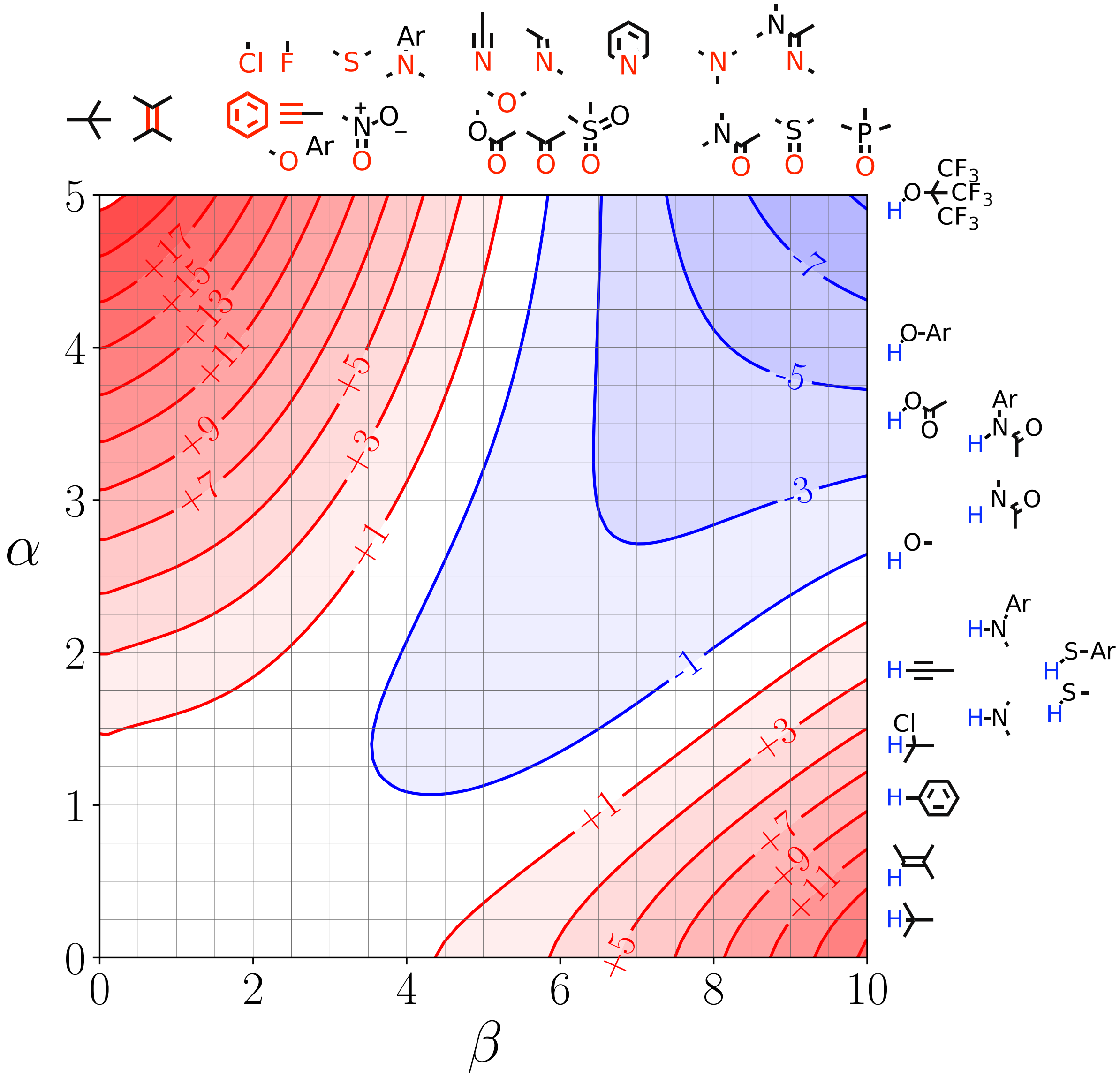

Figure S30: FGIP for 1-butanol at 298K.

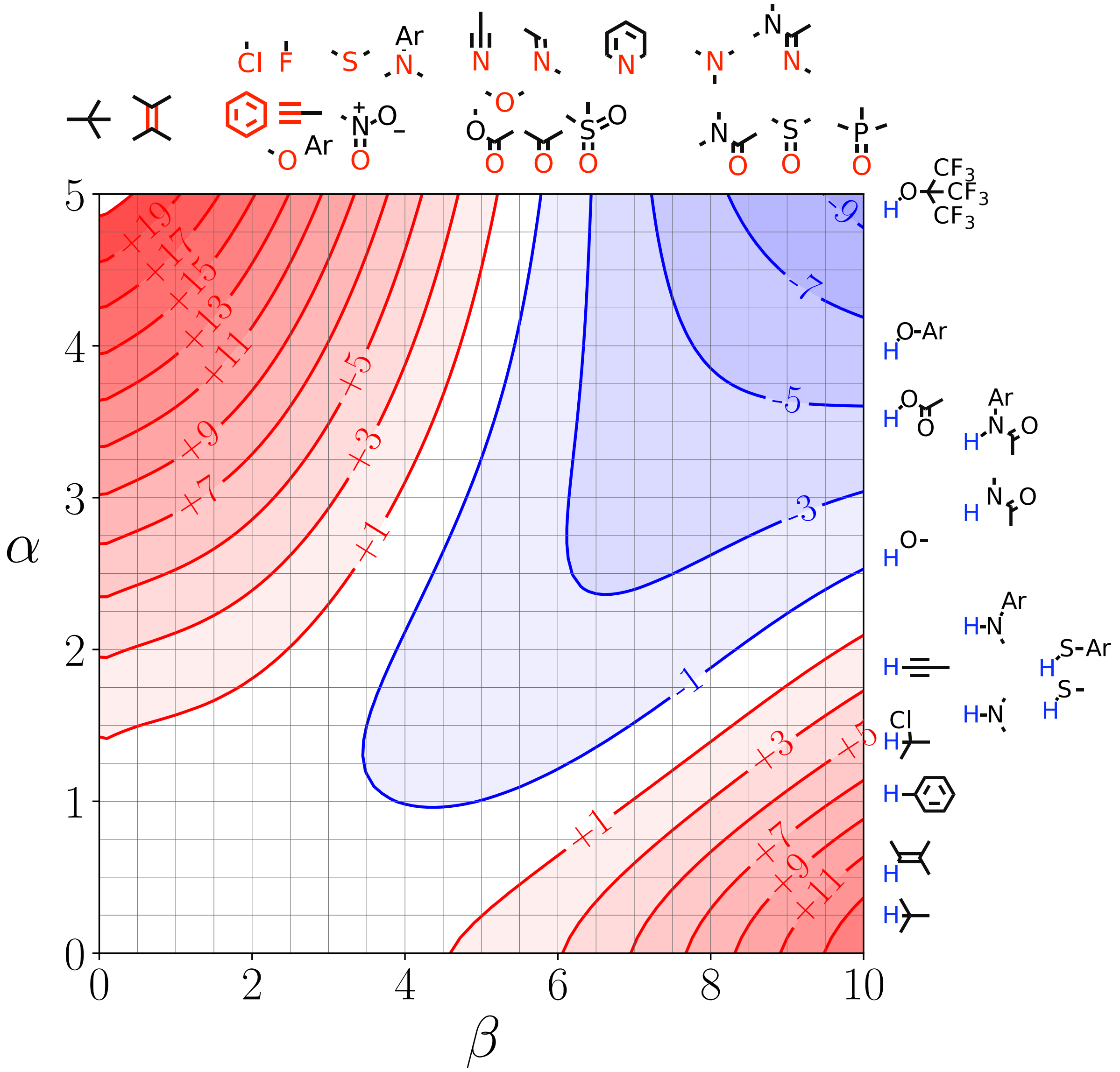

Figure S31: FGIP for 2-methyl-1-propanol at 298K.

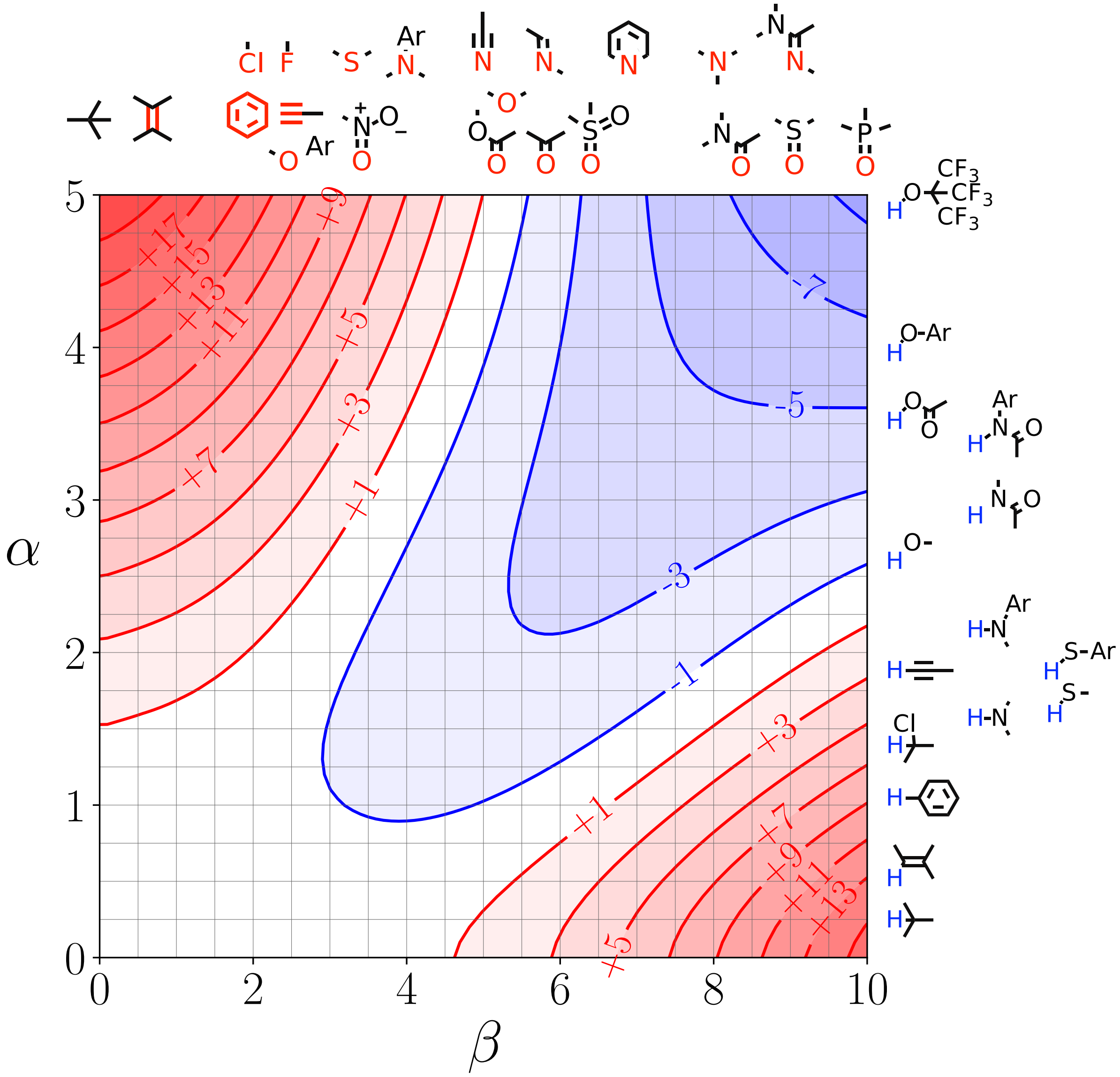

Figure S32: FGIP for 2-butanol at 298K.

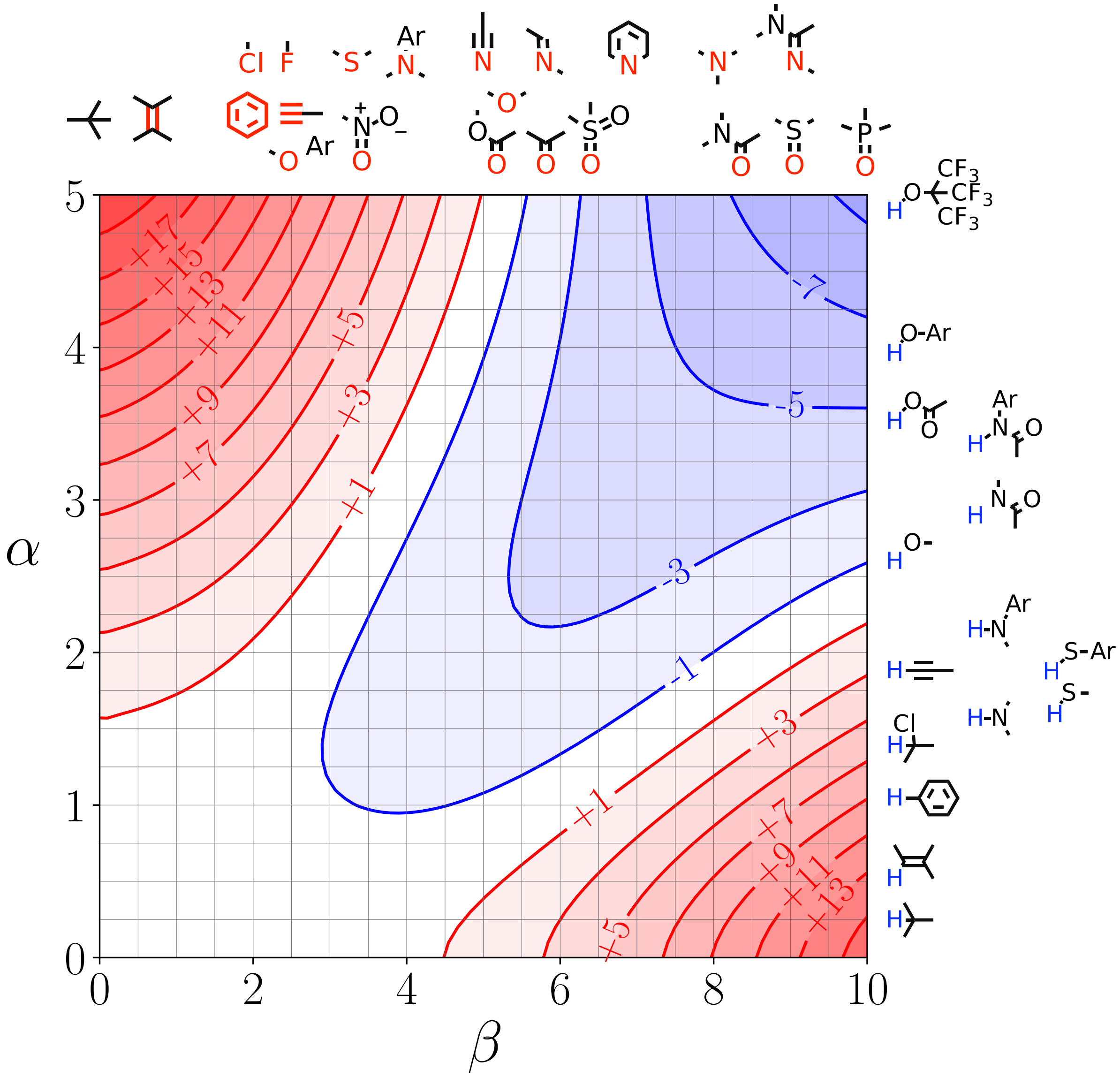

Figure S33: FGIP for 2-methyl-2-propanol at 298K.

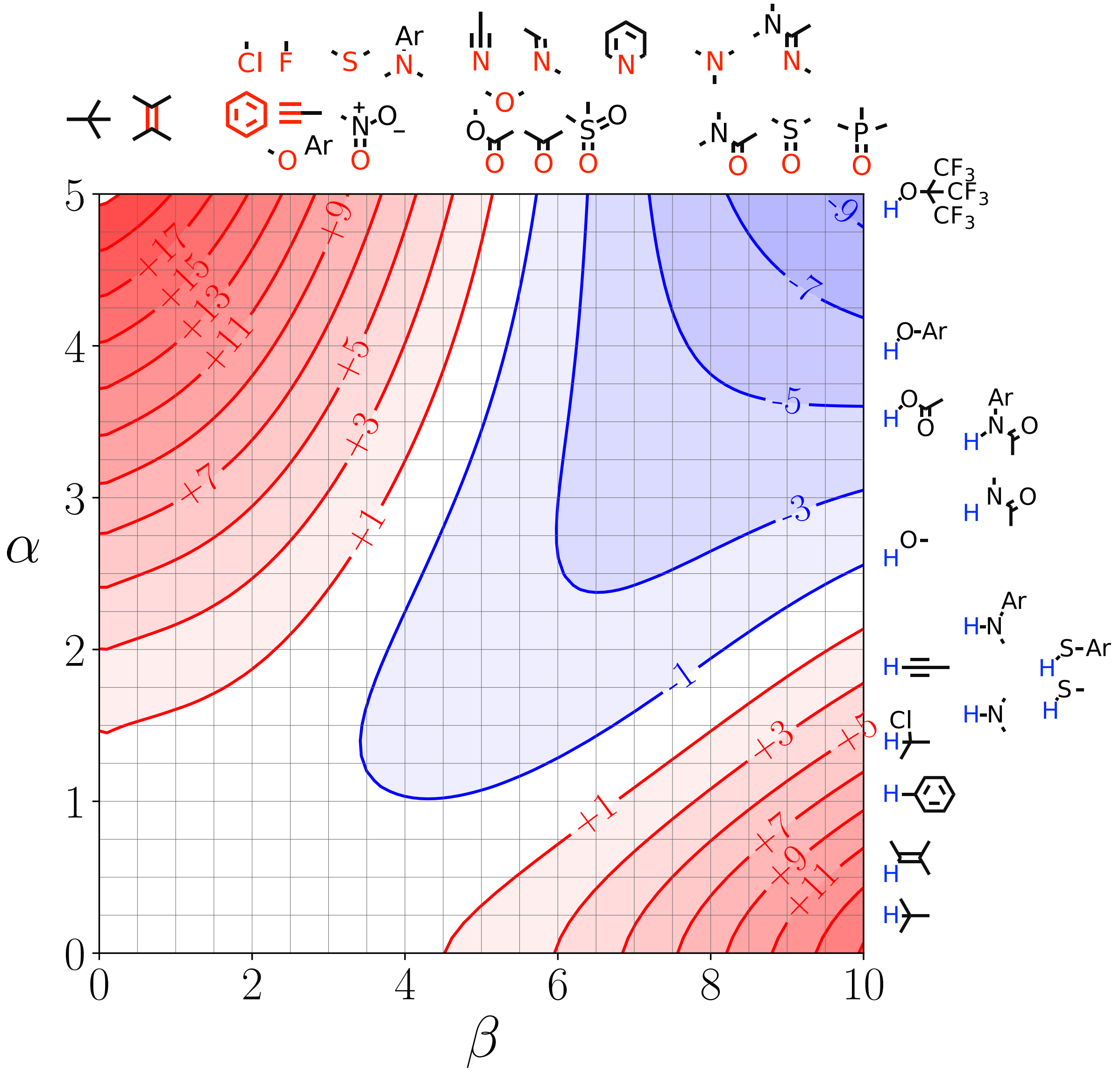

Figure S34: FGIP for 1-pentanol at 298K.

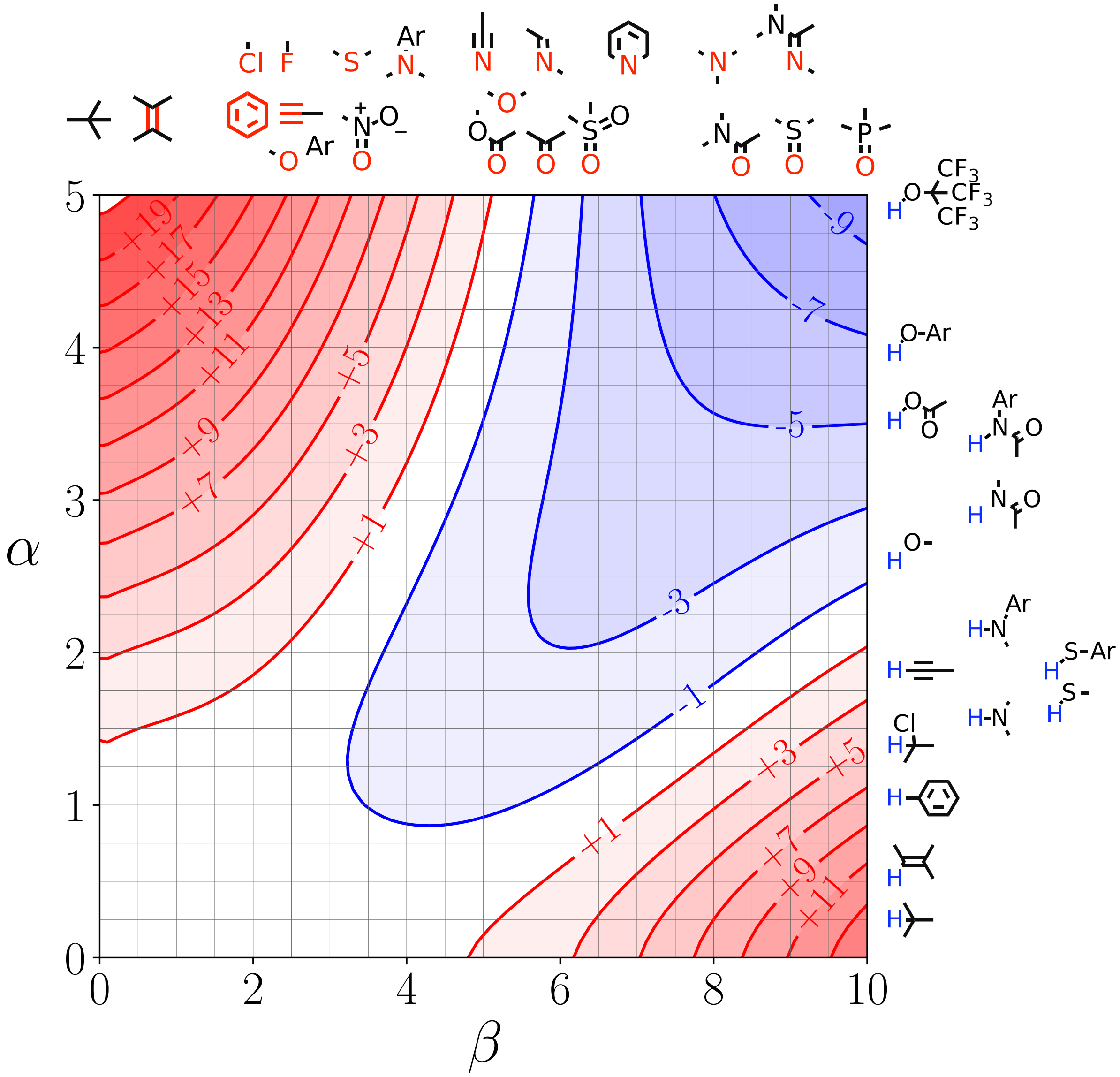

Figure S35: FGIP for 3-methyl-1-butanol at 298K.

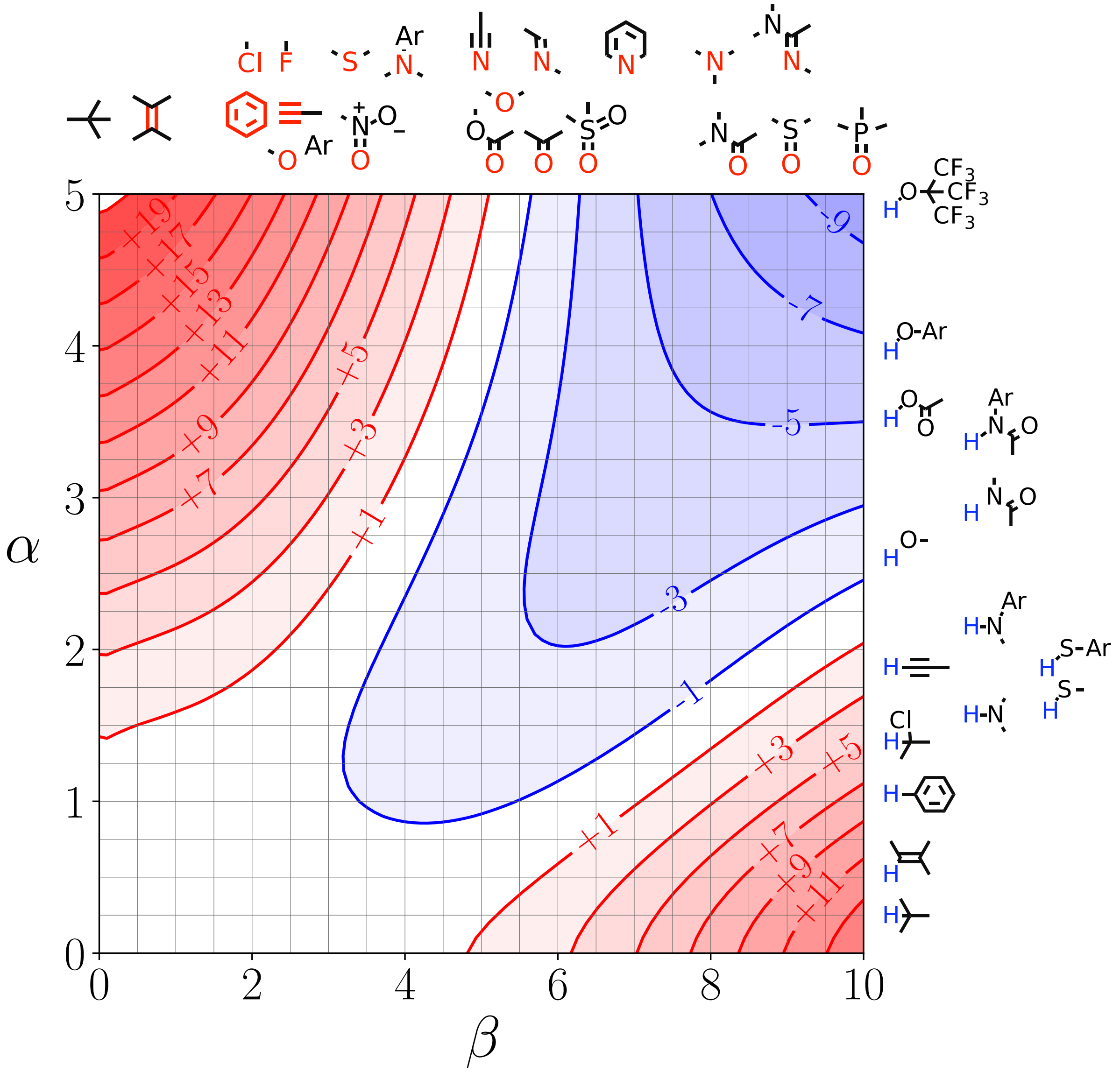

Figure S36: FGIP for 2-methyl-2-butanol at 298K.

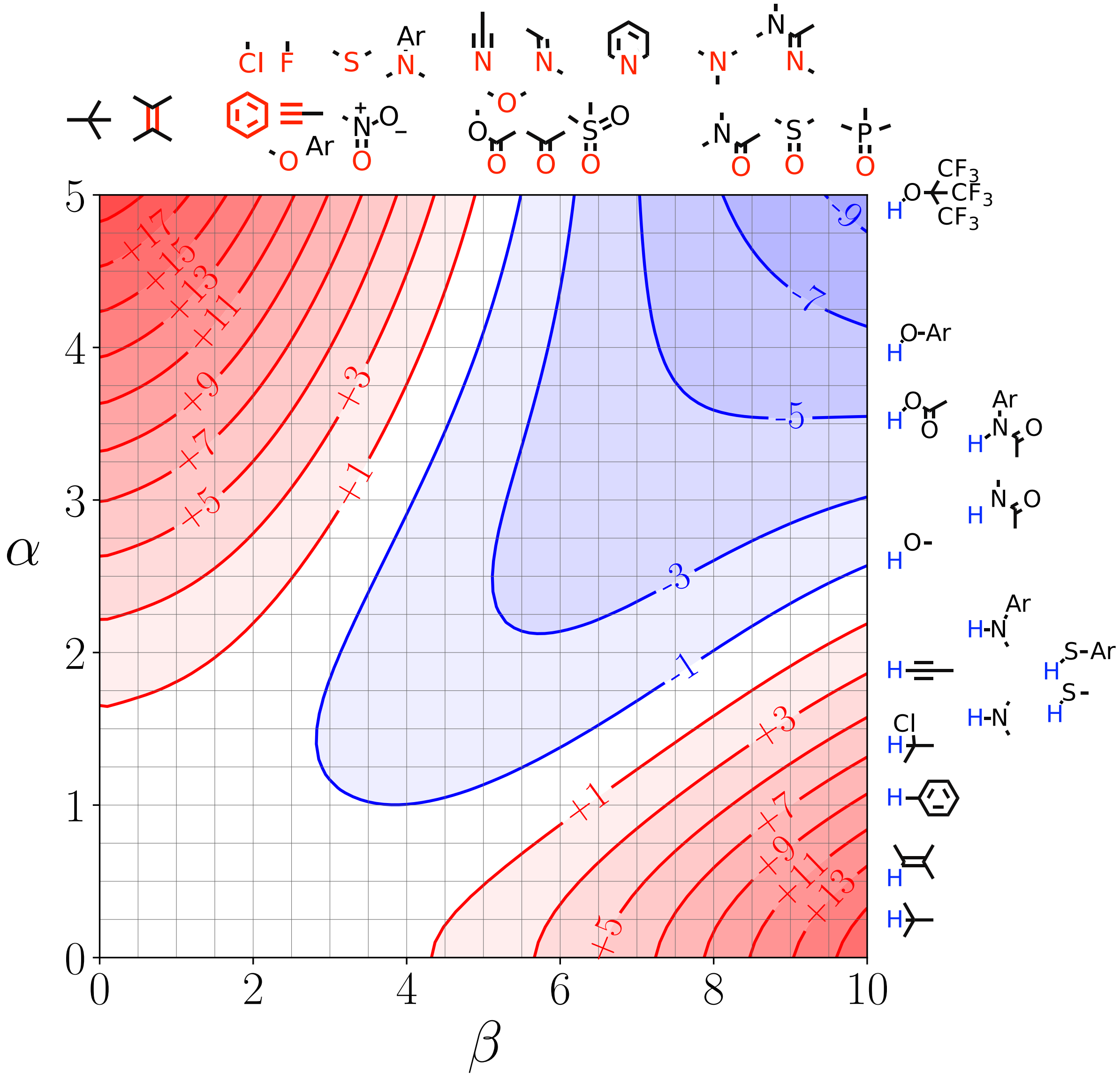

Figure S37: FGIP for 1-hexanol at 298K.

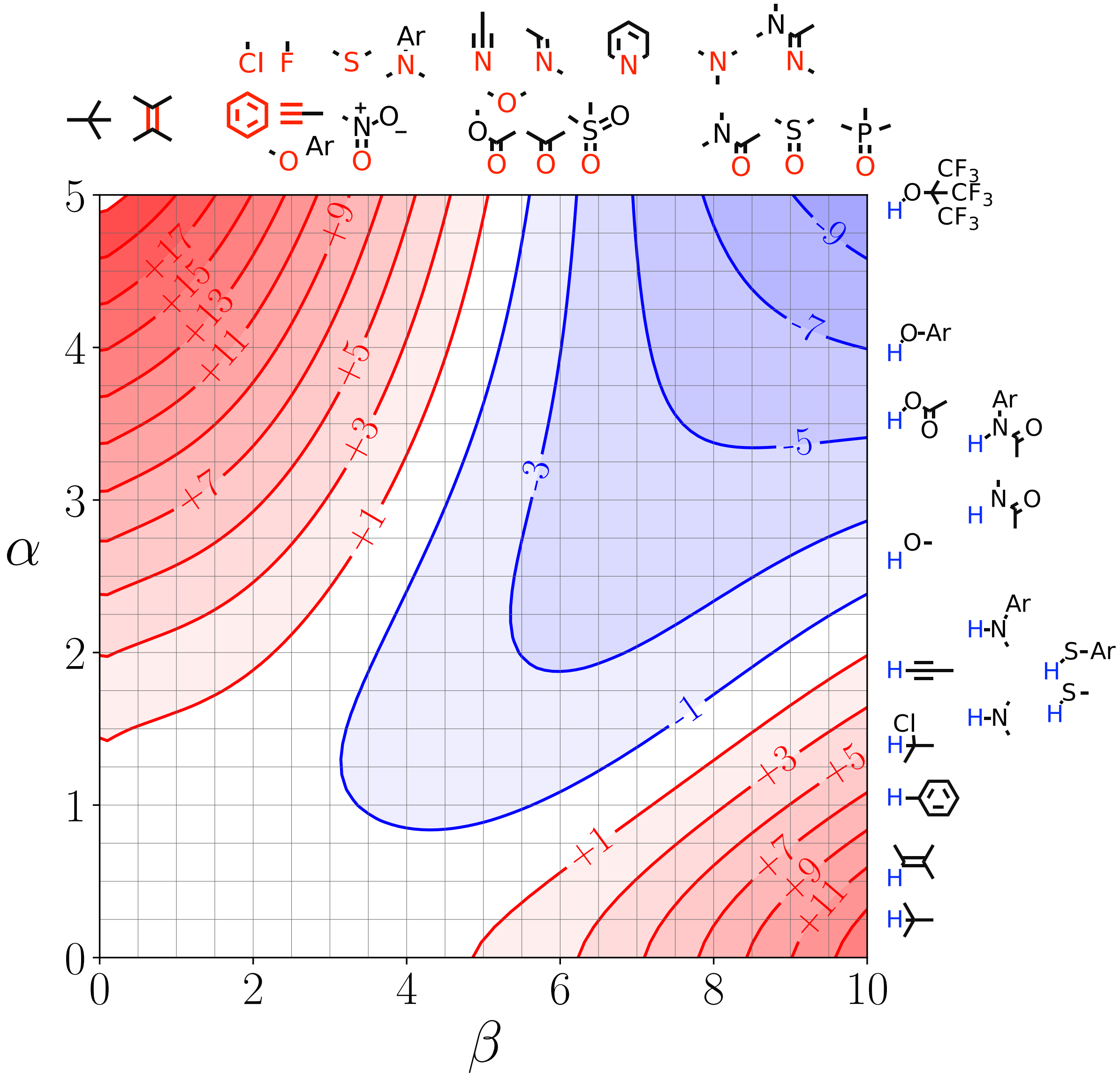

Figure S38: FGIP for cyclohexanol at 298K.

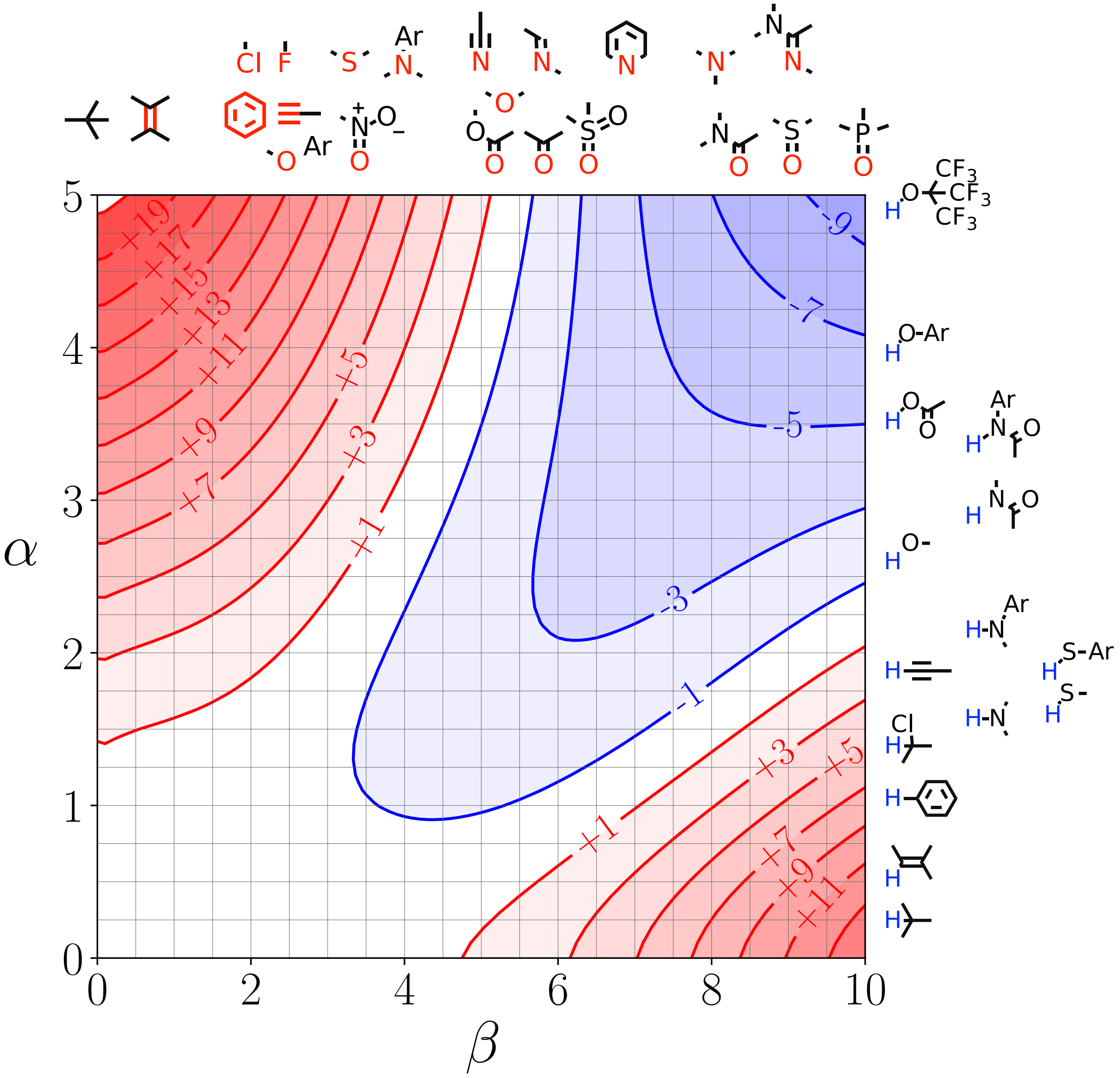

Figure S39: FGIP for 1-octanol at 298K.

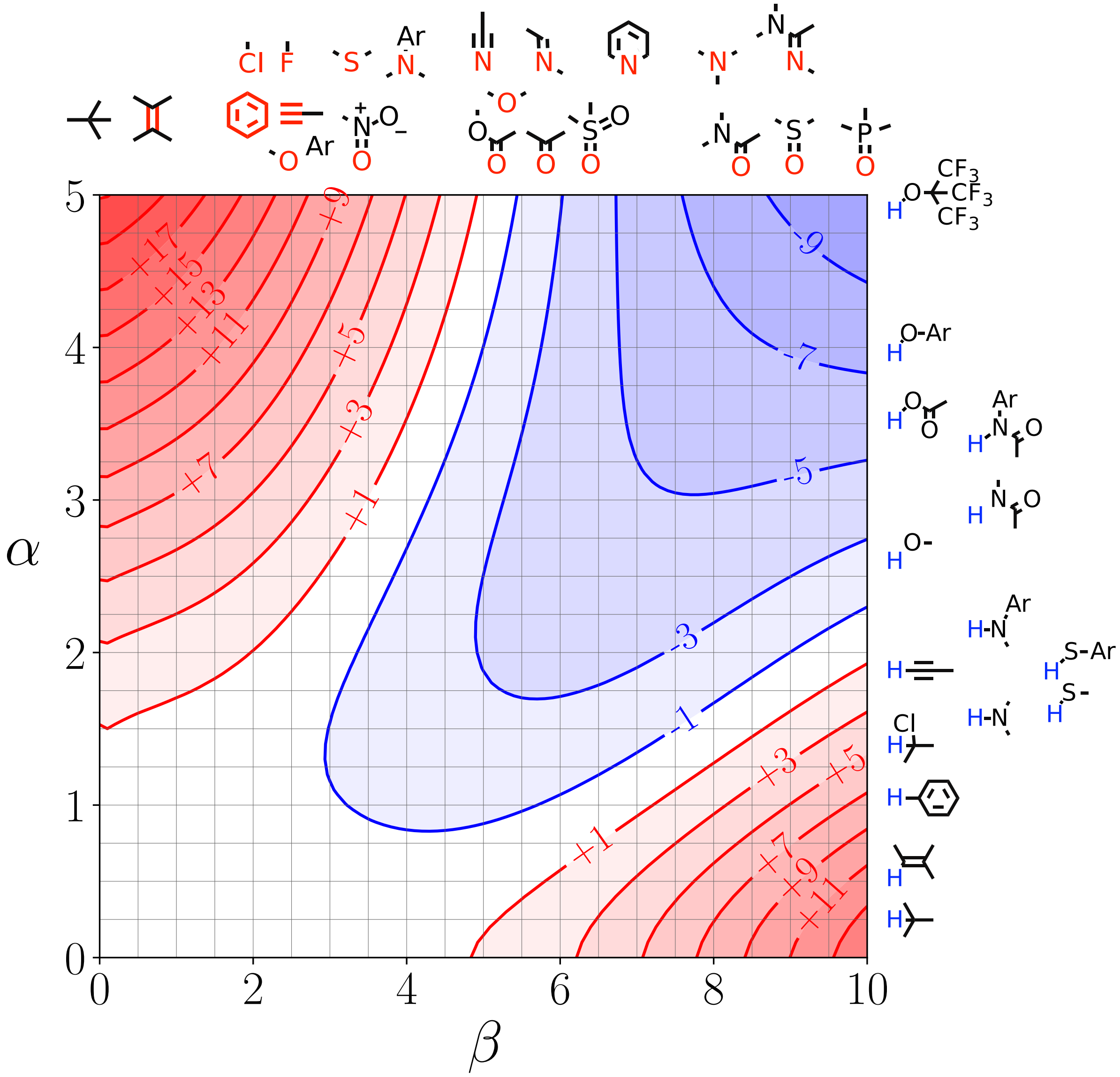

Figure S40: FGIP for 1-decanol at 298K.

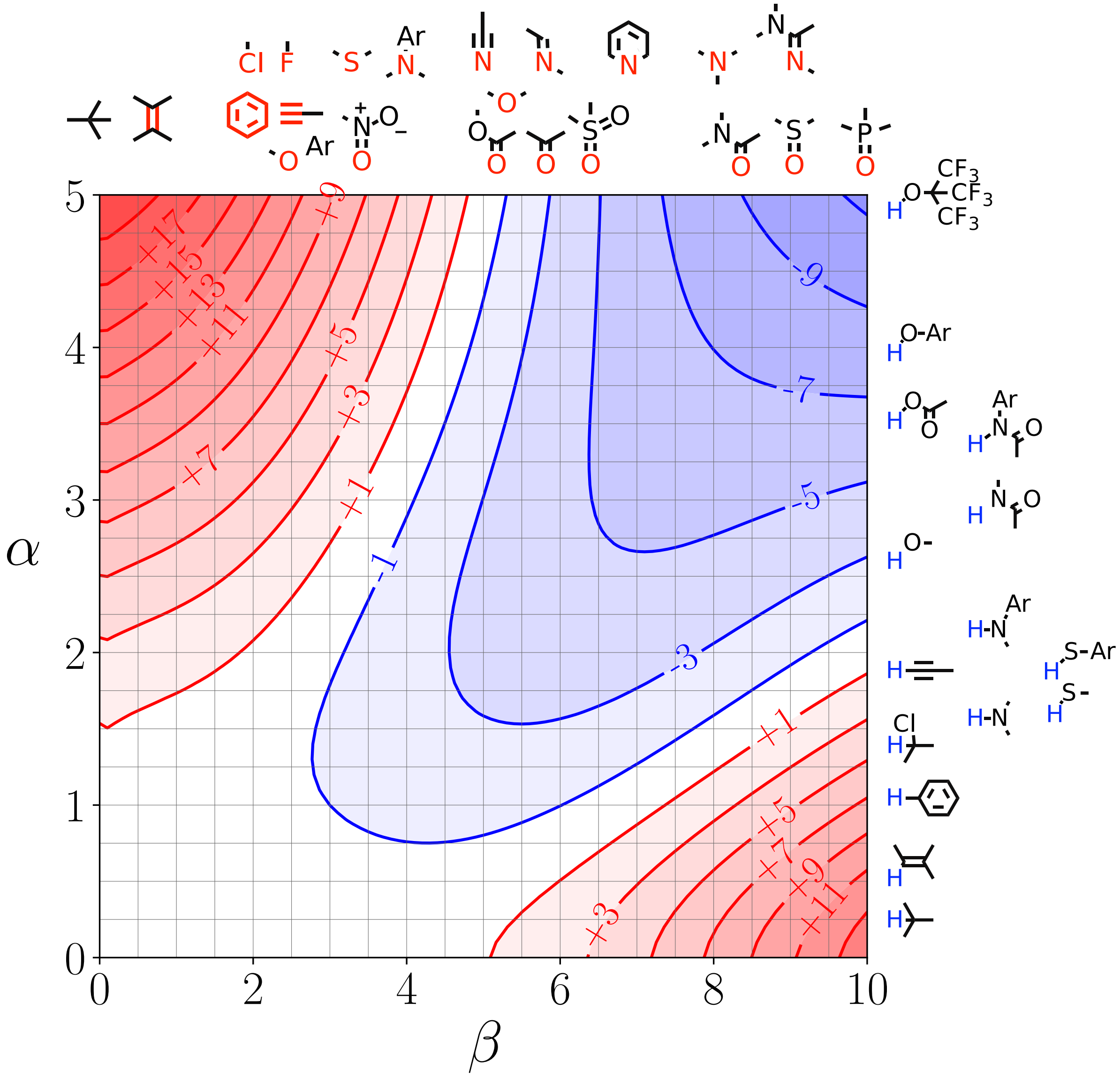

Figure S41: FGIP for 1-dodecanol at 298K.

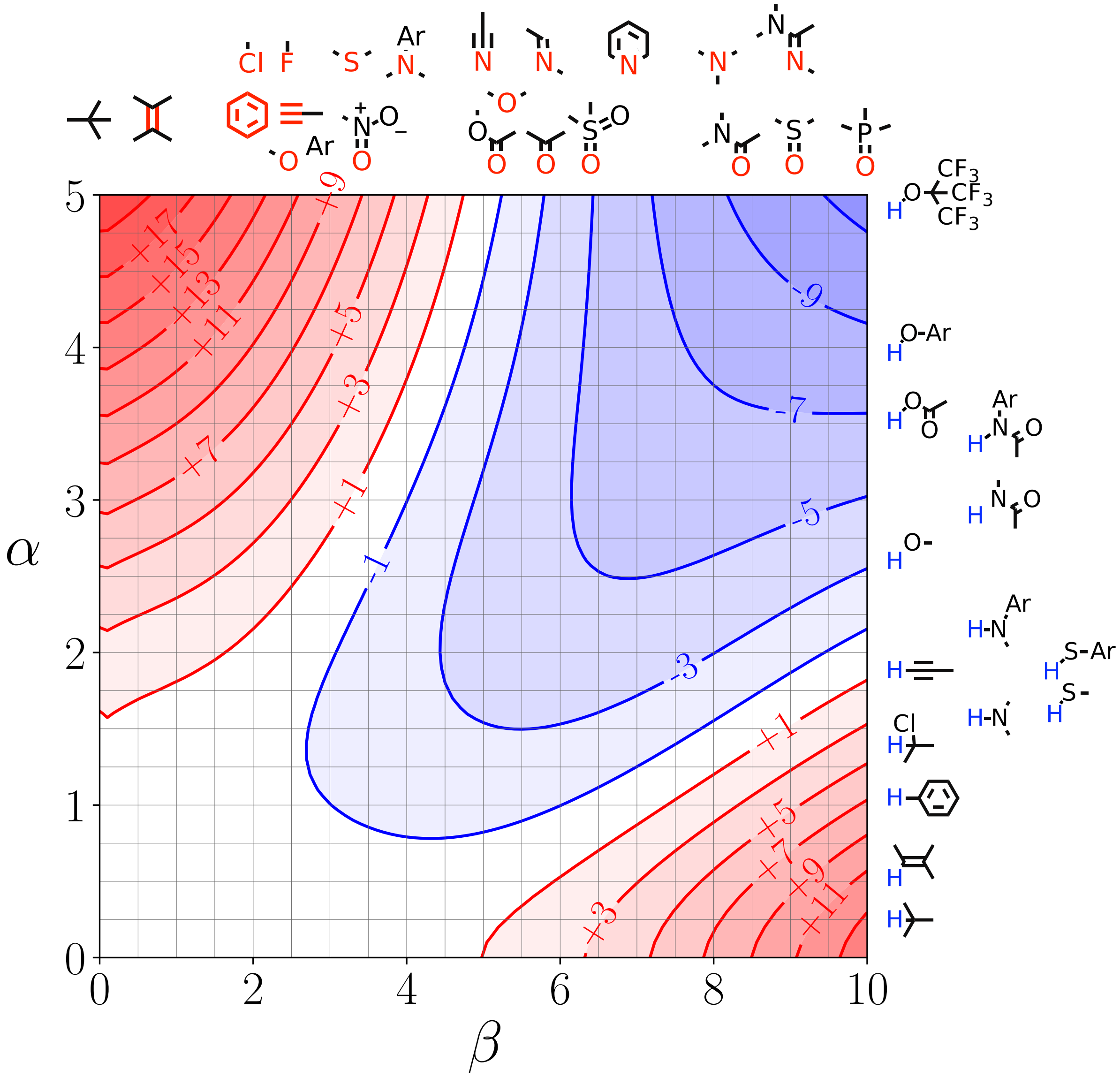

Figure S42: FGIP for benzyl alcohol at 298K.

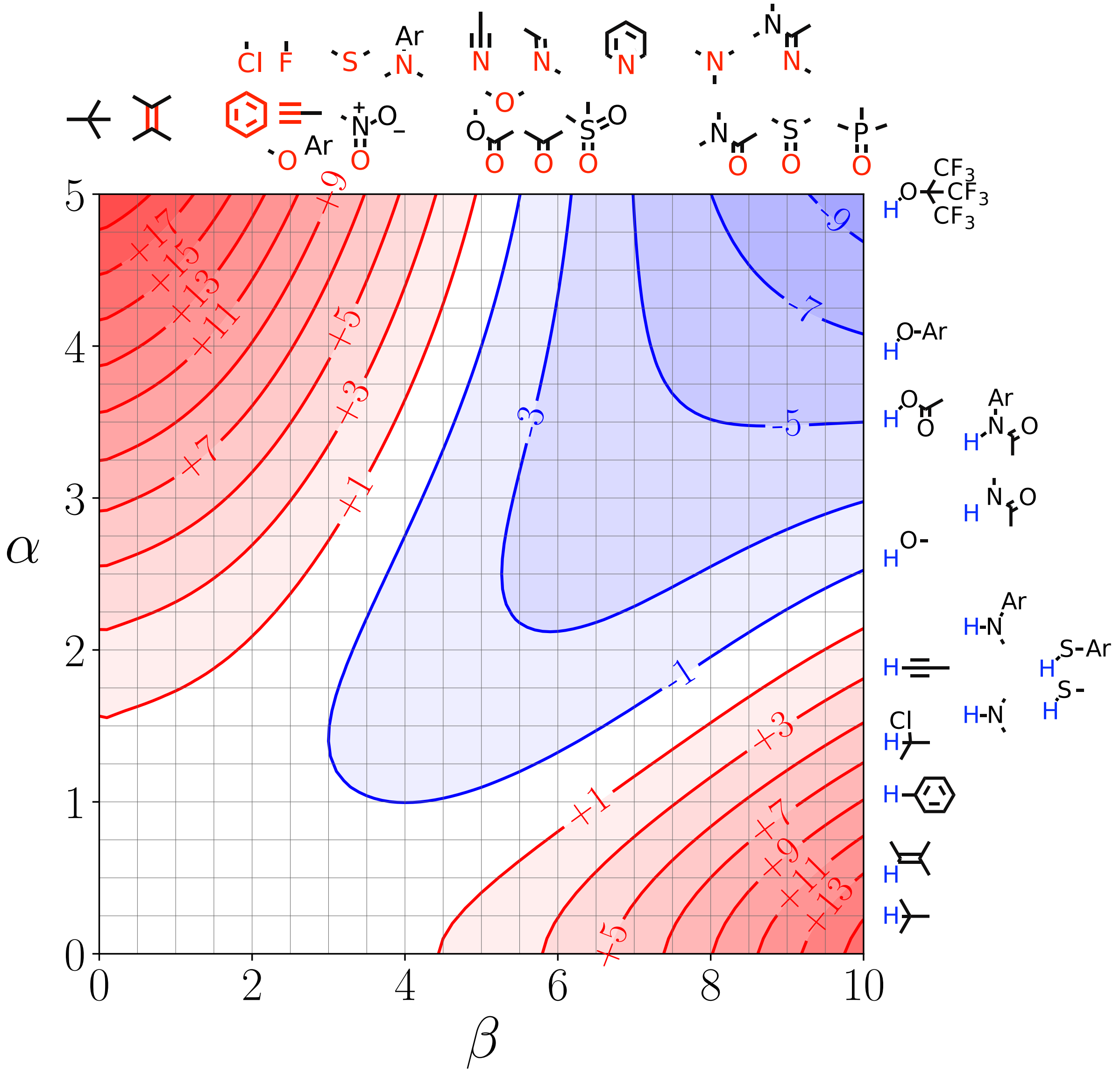

Figure S43: FGIP for 2-phenylethanol at 298K.

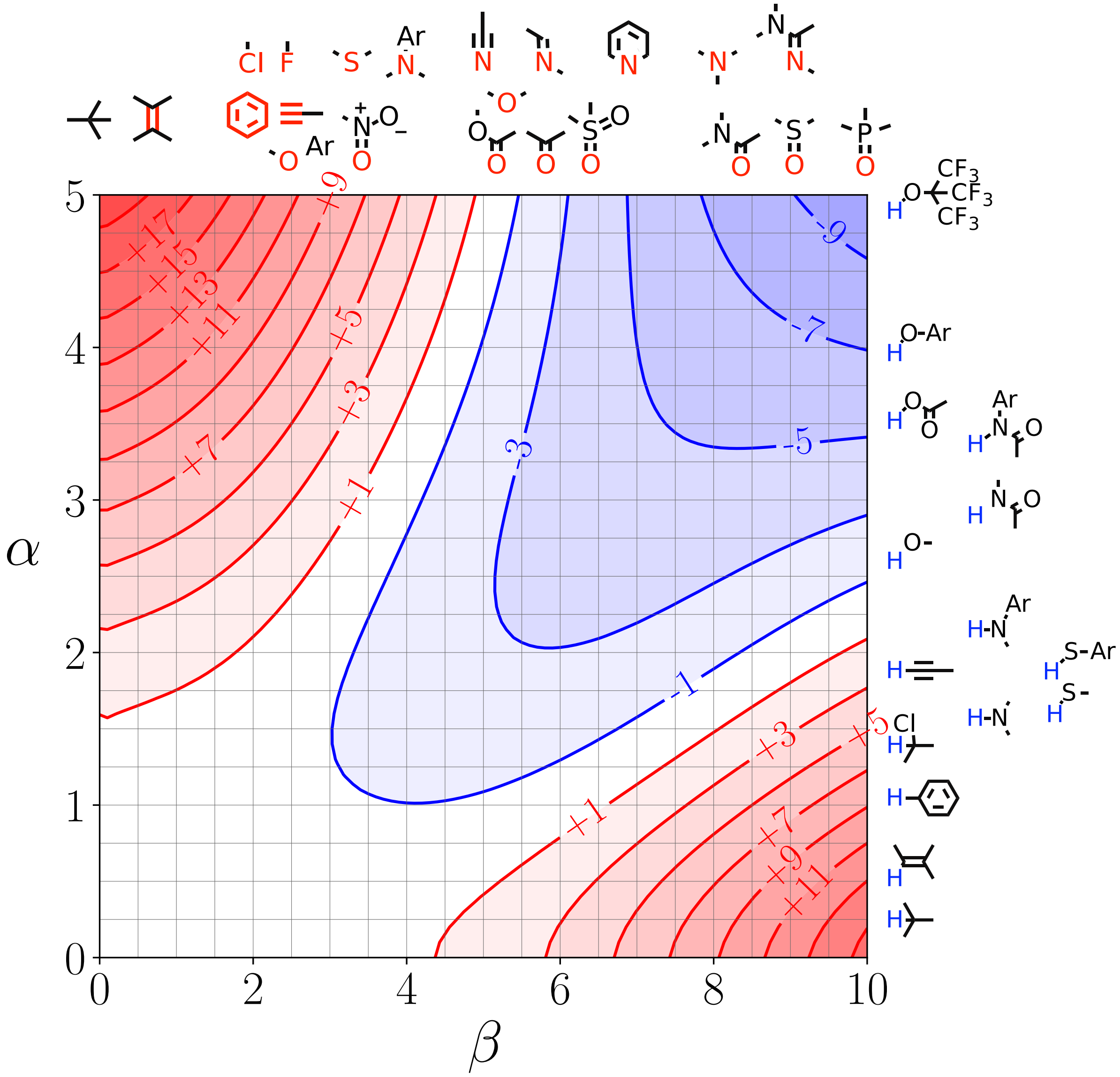

Figure S44: FGIP for allyl alcohol at 298K.

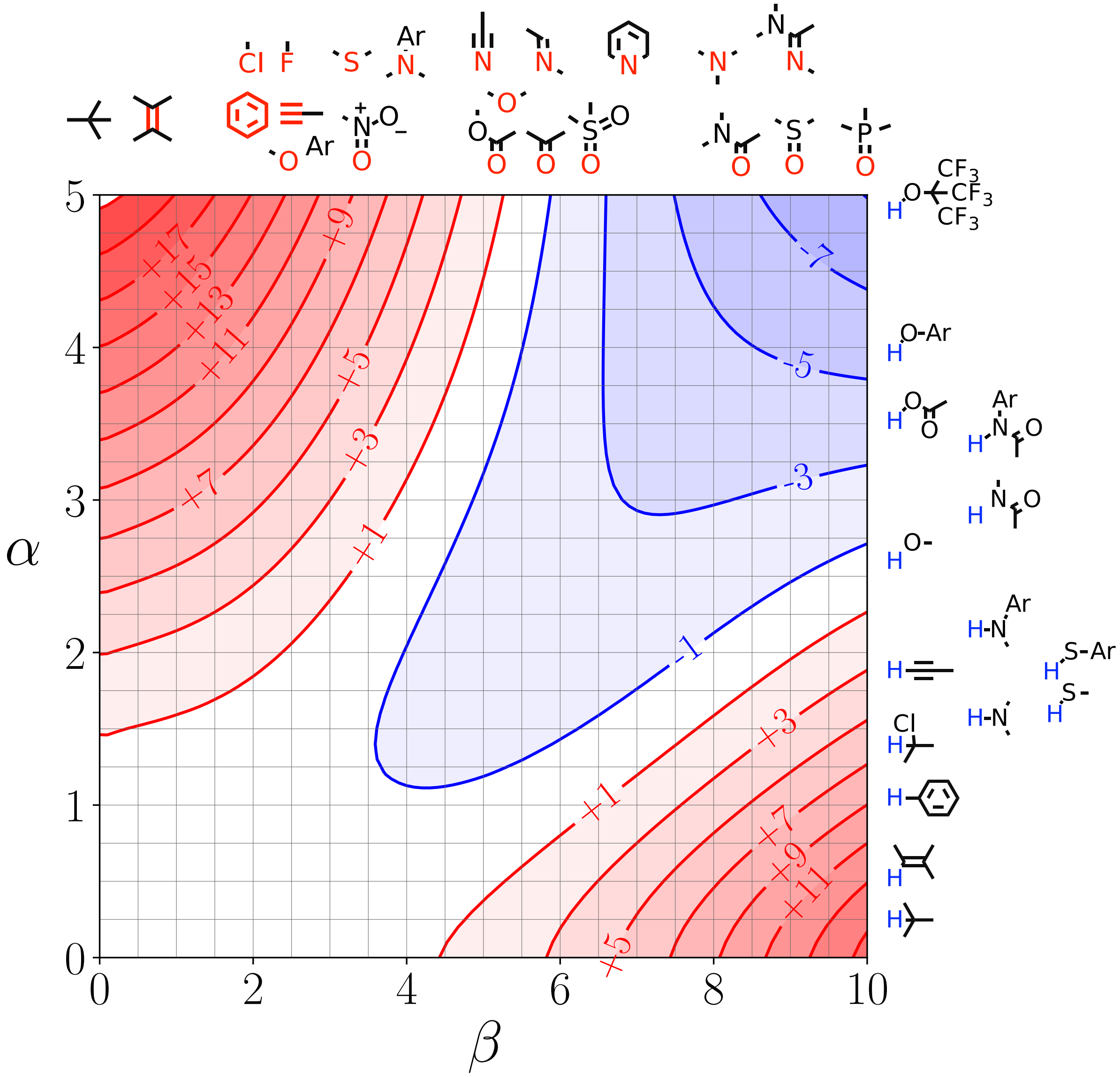

Figure S45: FGIP for 2-chloroethanol at 298K.

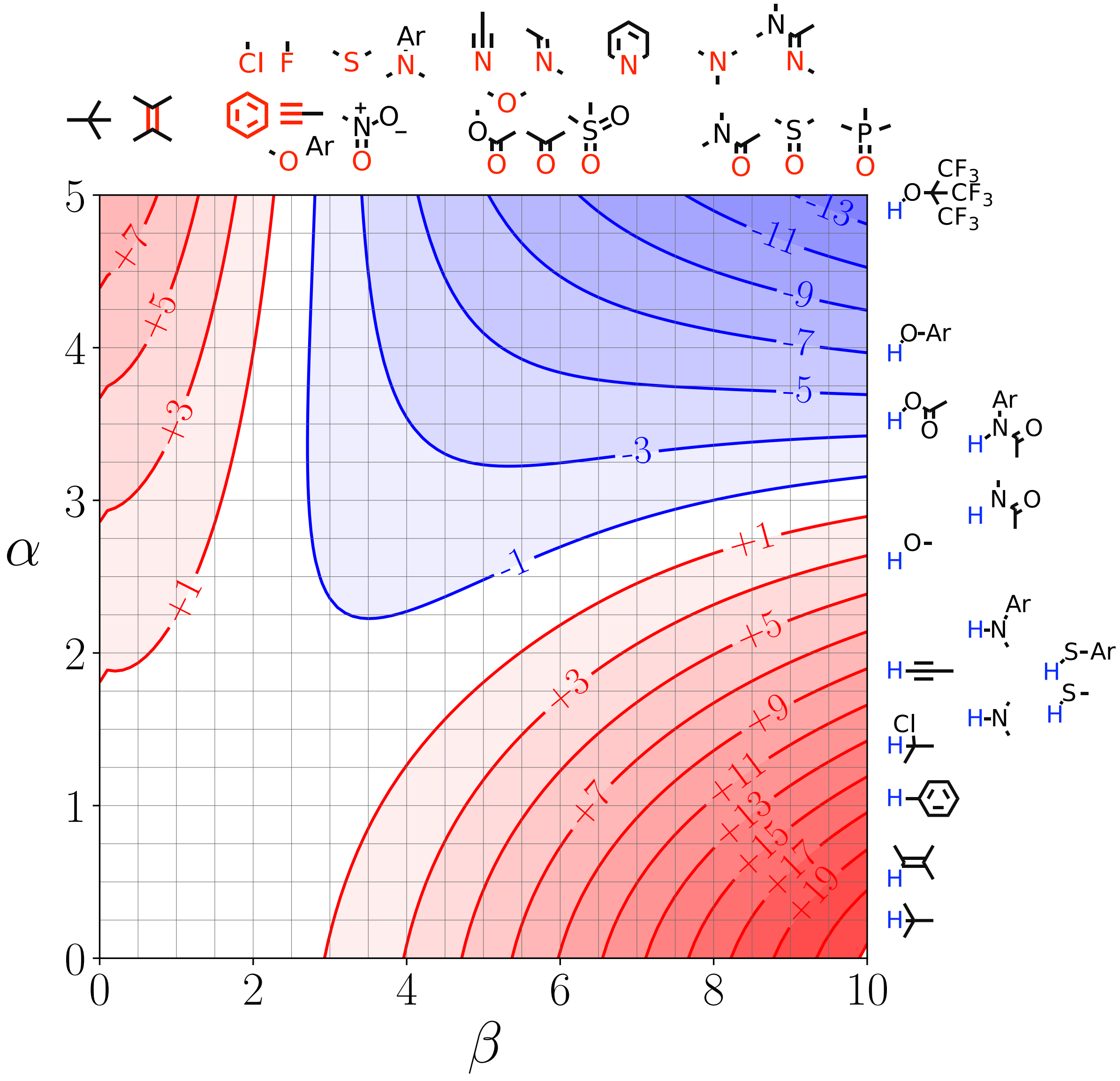

Figure S46: FGIP for 2-cyanoethanol at 298K.

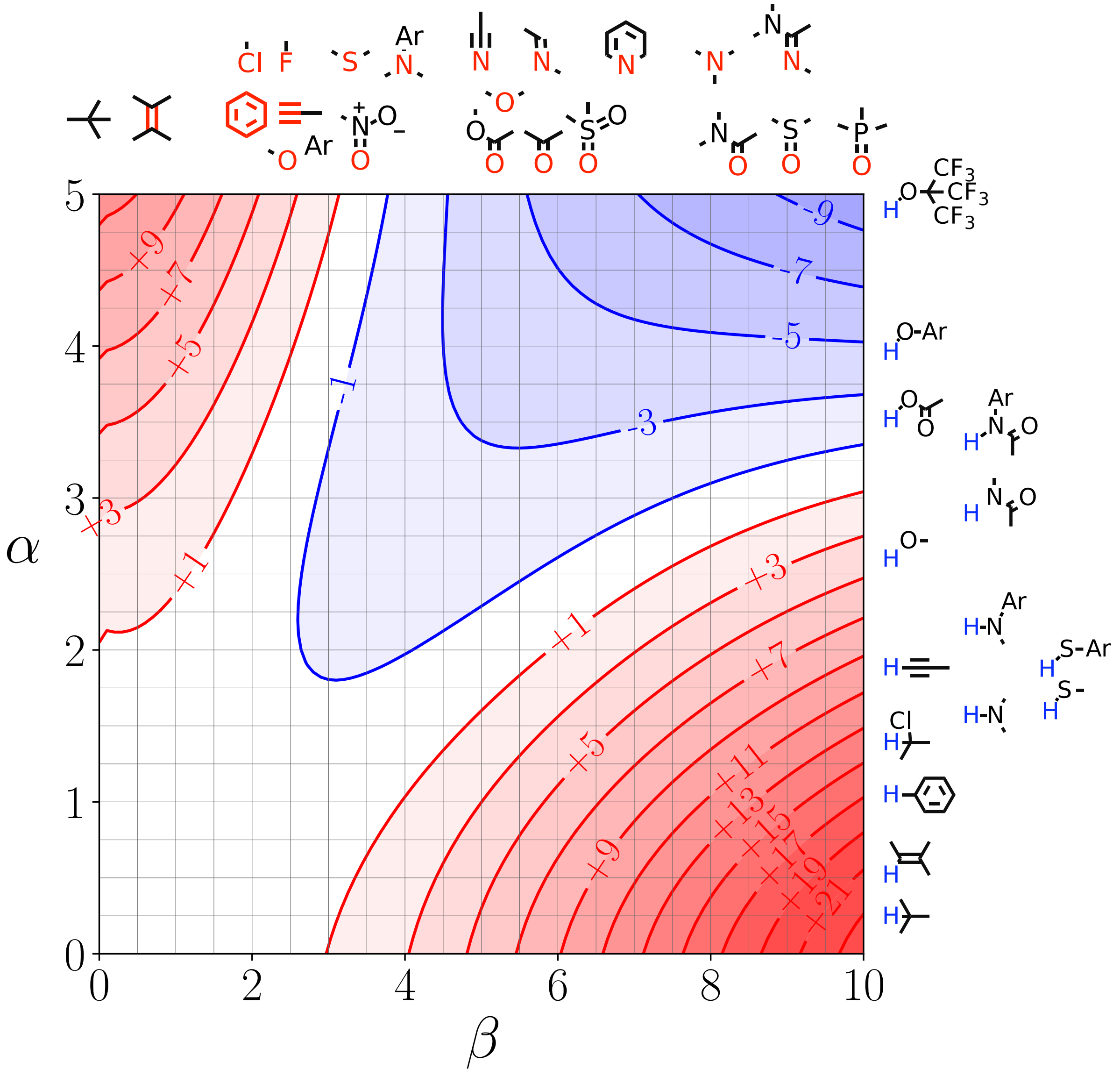

Figure S47: FGIP for 2,2,2-trifluoroethanol at 298K.

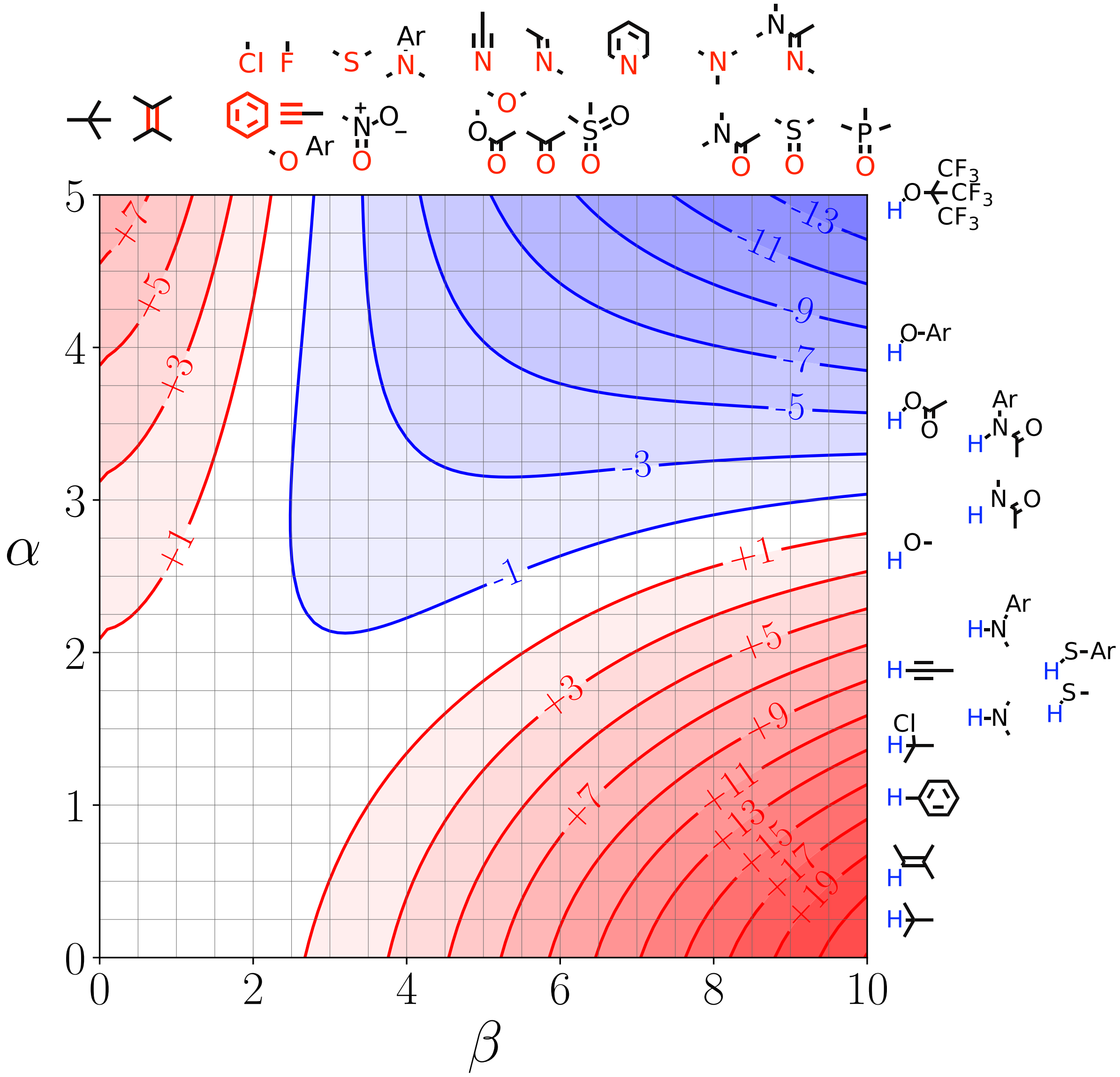

Figure S48: FGIP for 1,1,1,3,3,3-hexafluoro-2-propanol at 298K.

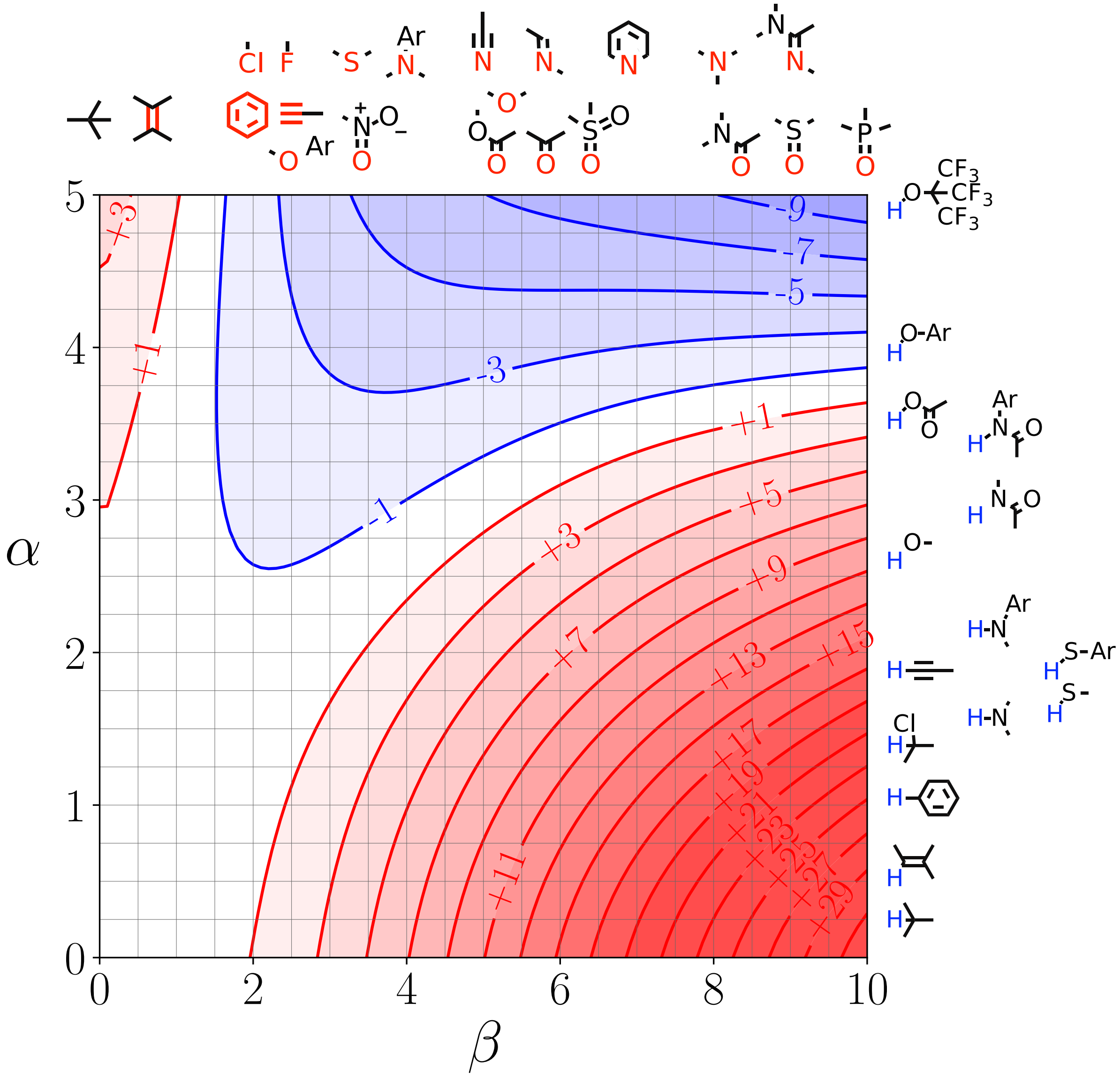

Figure S49: FGIP for 2-methoxyethanol at 298K.

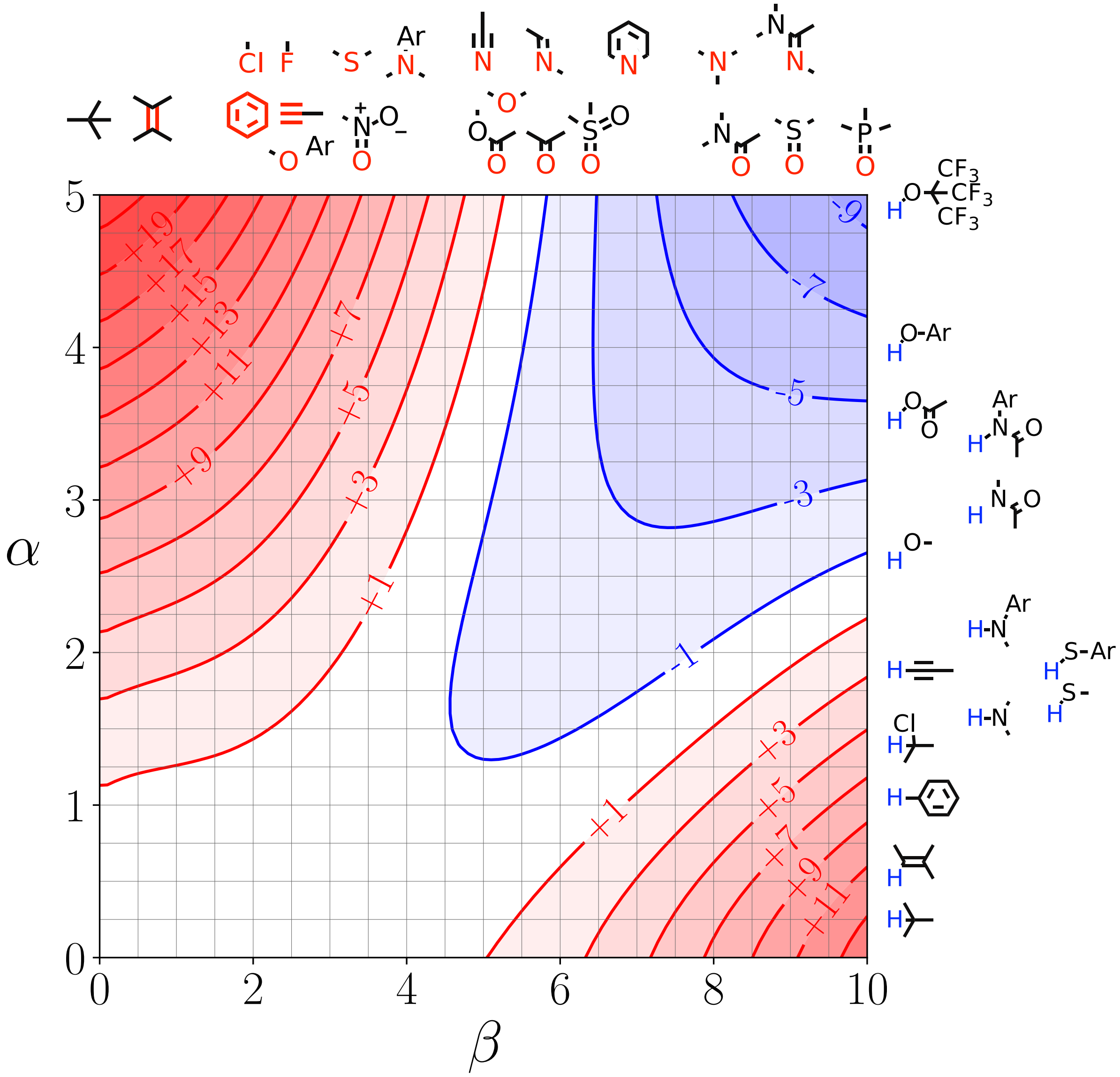

Figure S50: FGIP for 2-ethoxyethanol at 298K.

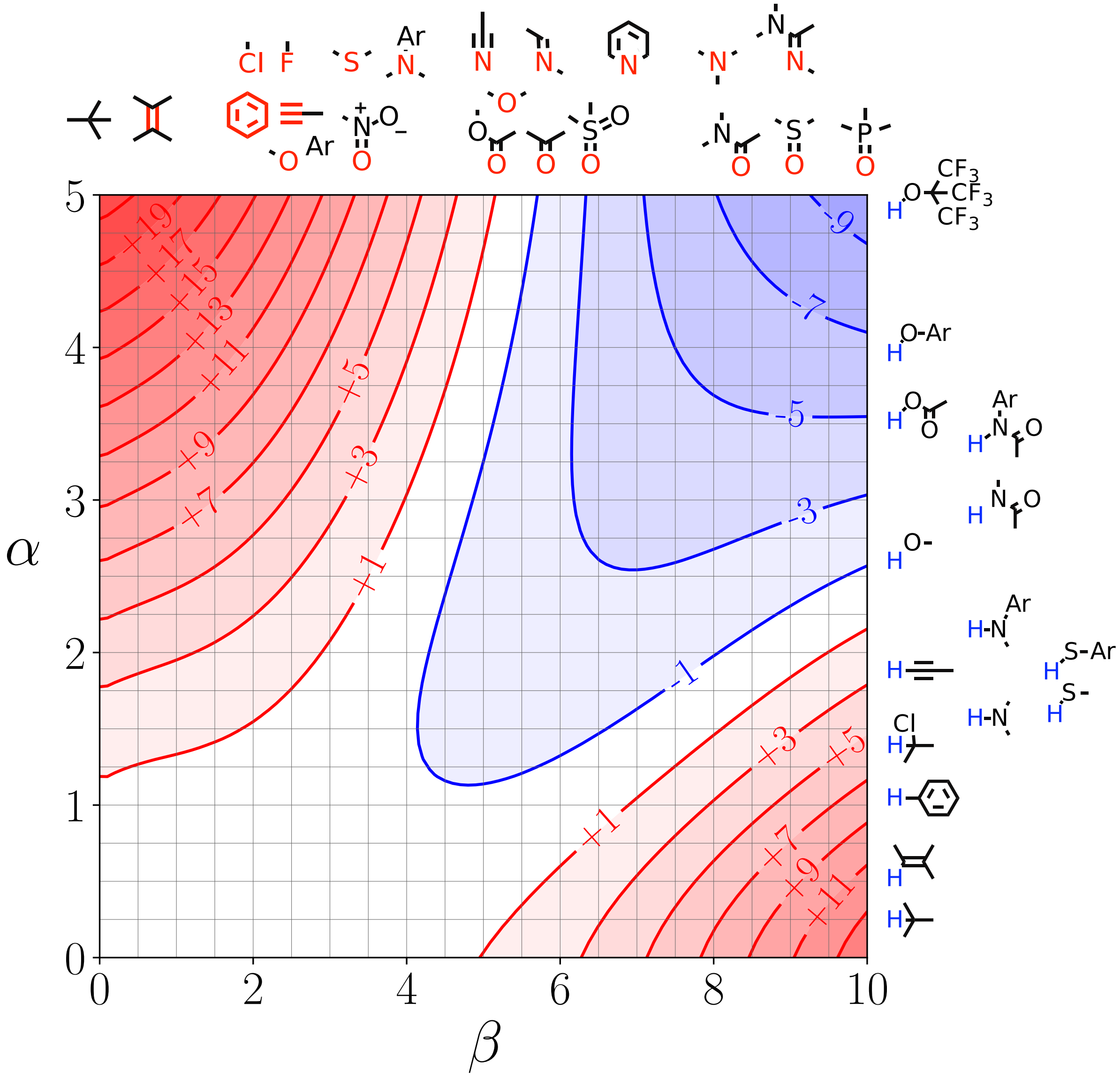

Figure S51: FGIP for ethylene glycol at 298K.

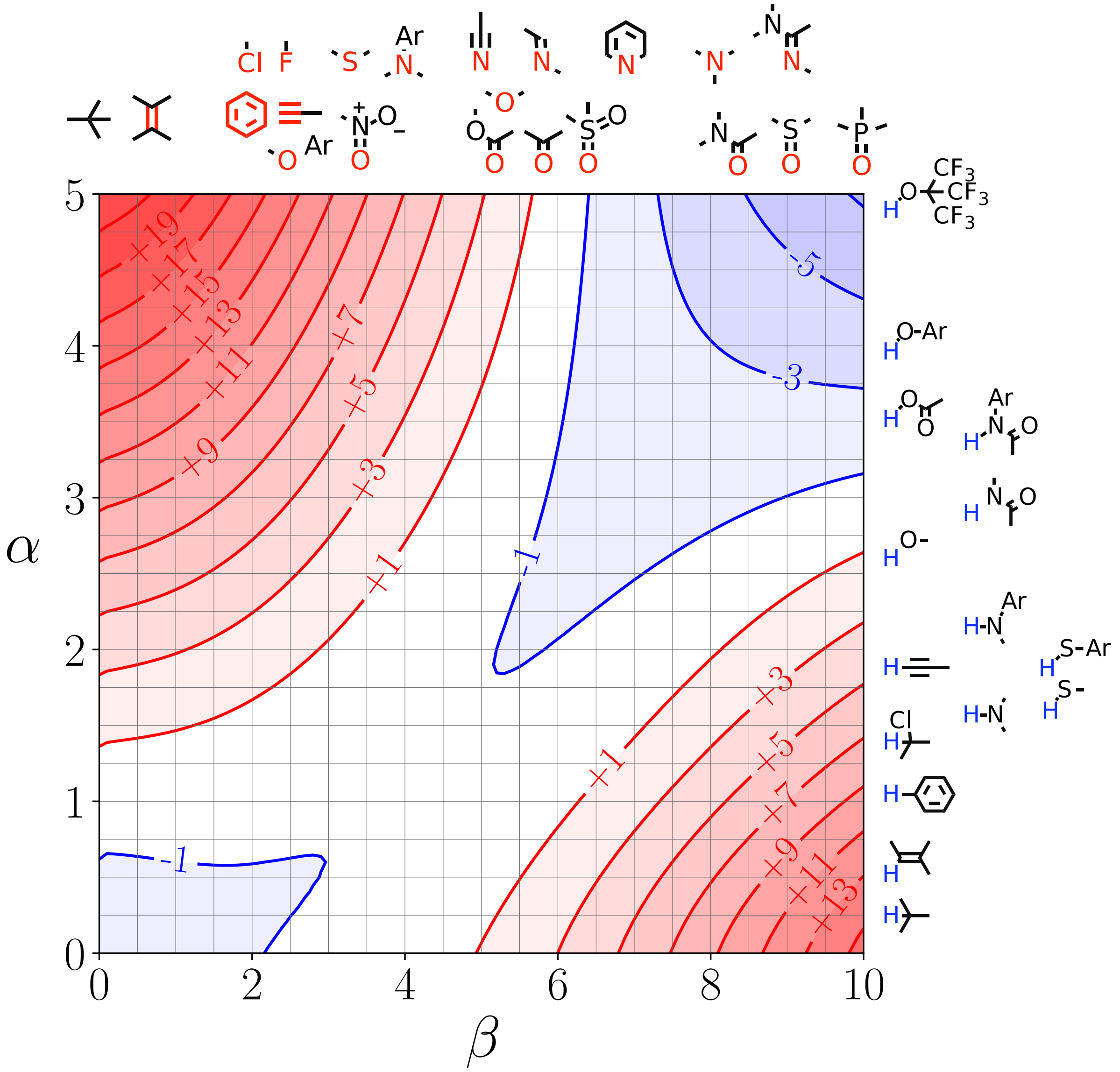

Figure S52: FGIP for 1,2-propanediol at 298K.

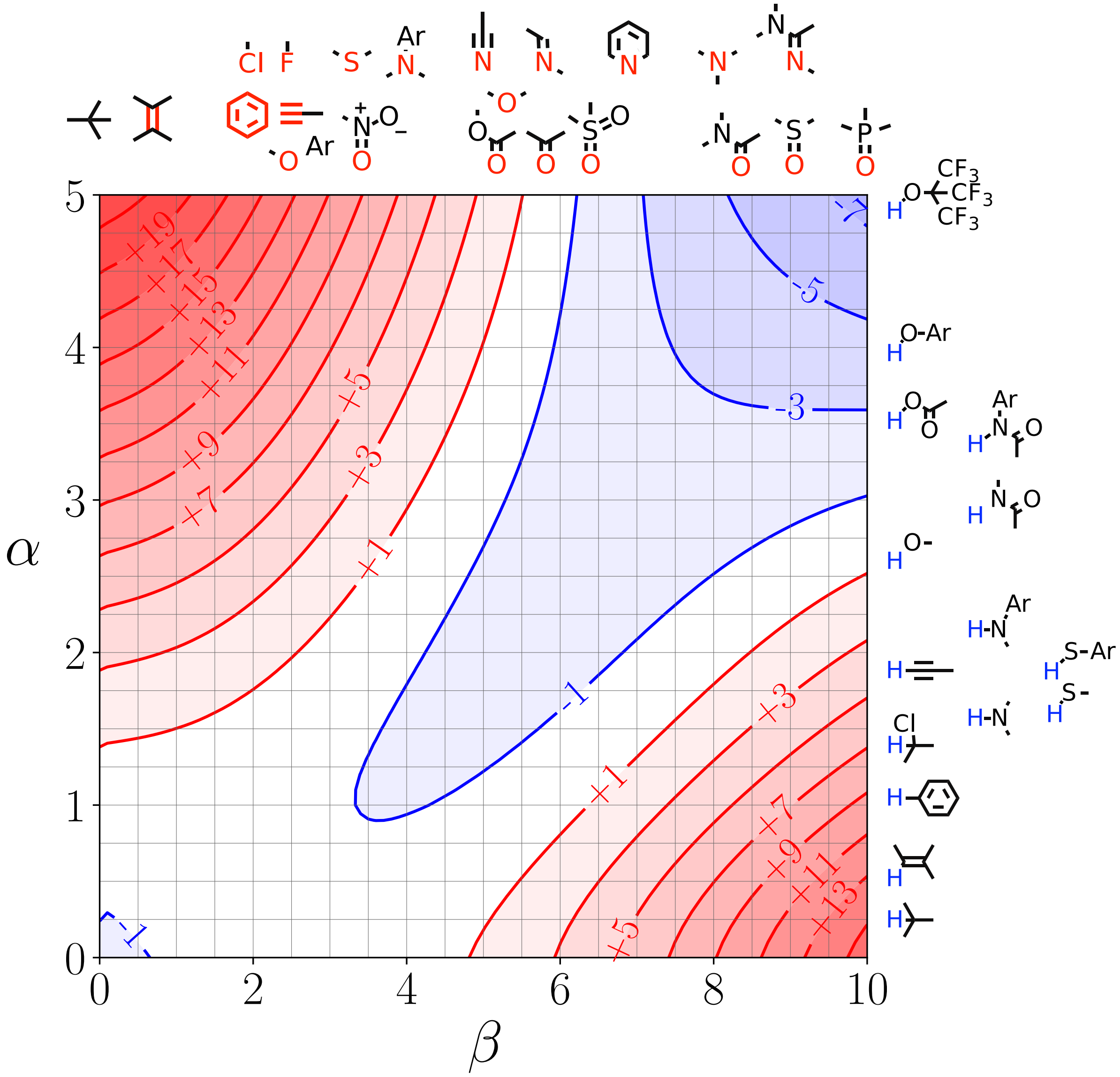

Figure S53: FGIP for 1,3-propanediol at 298K.

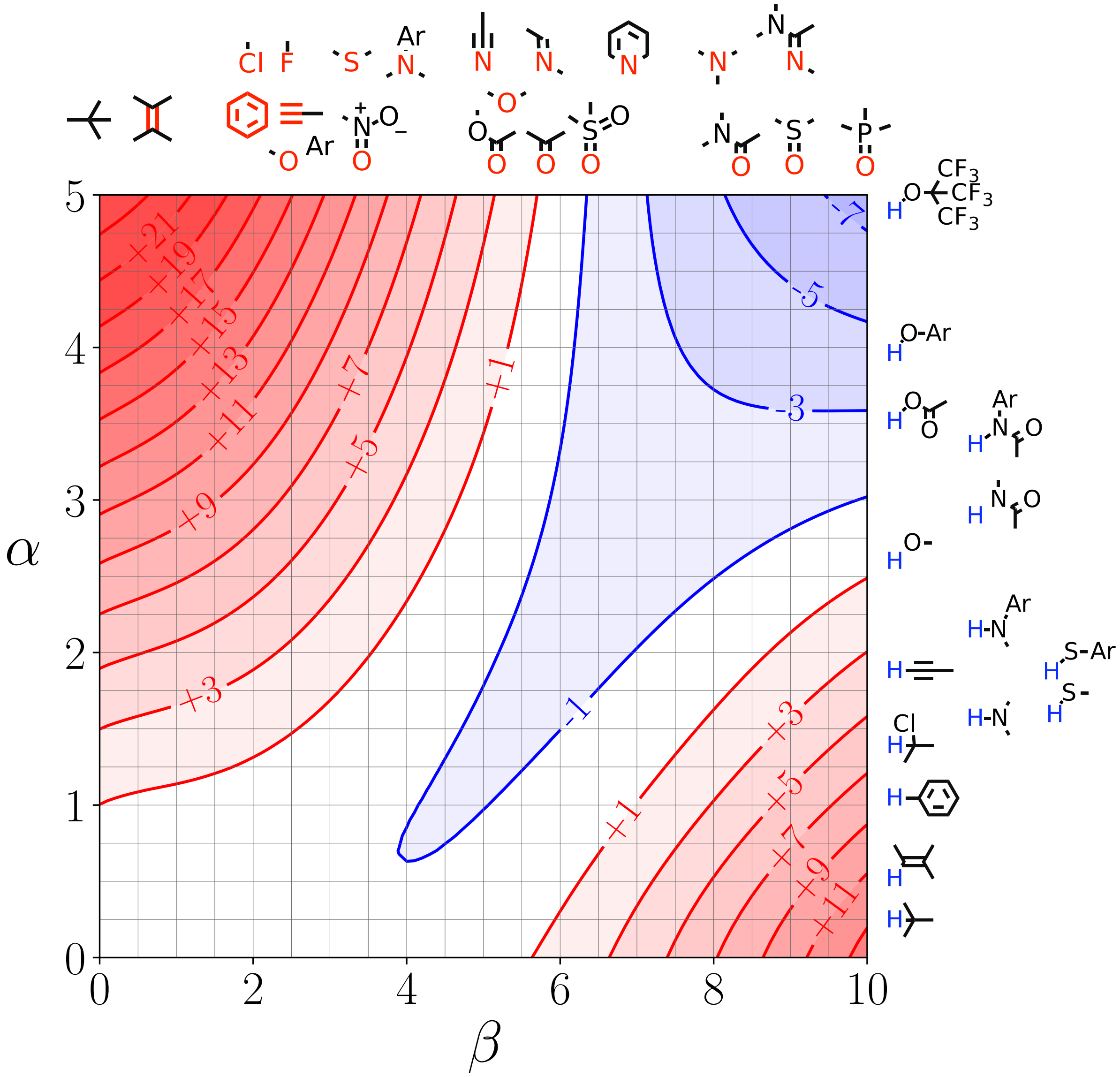

Figure S54: FGIP for 1,2-butanediol at 298K.

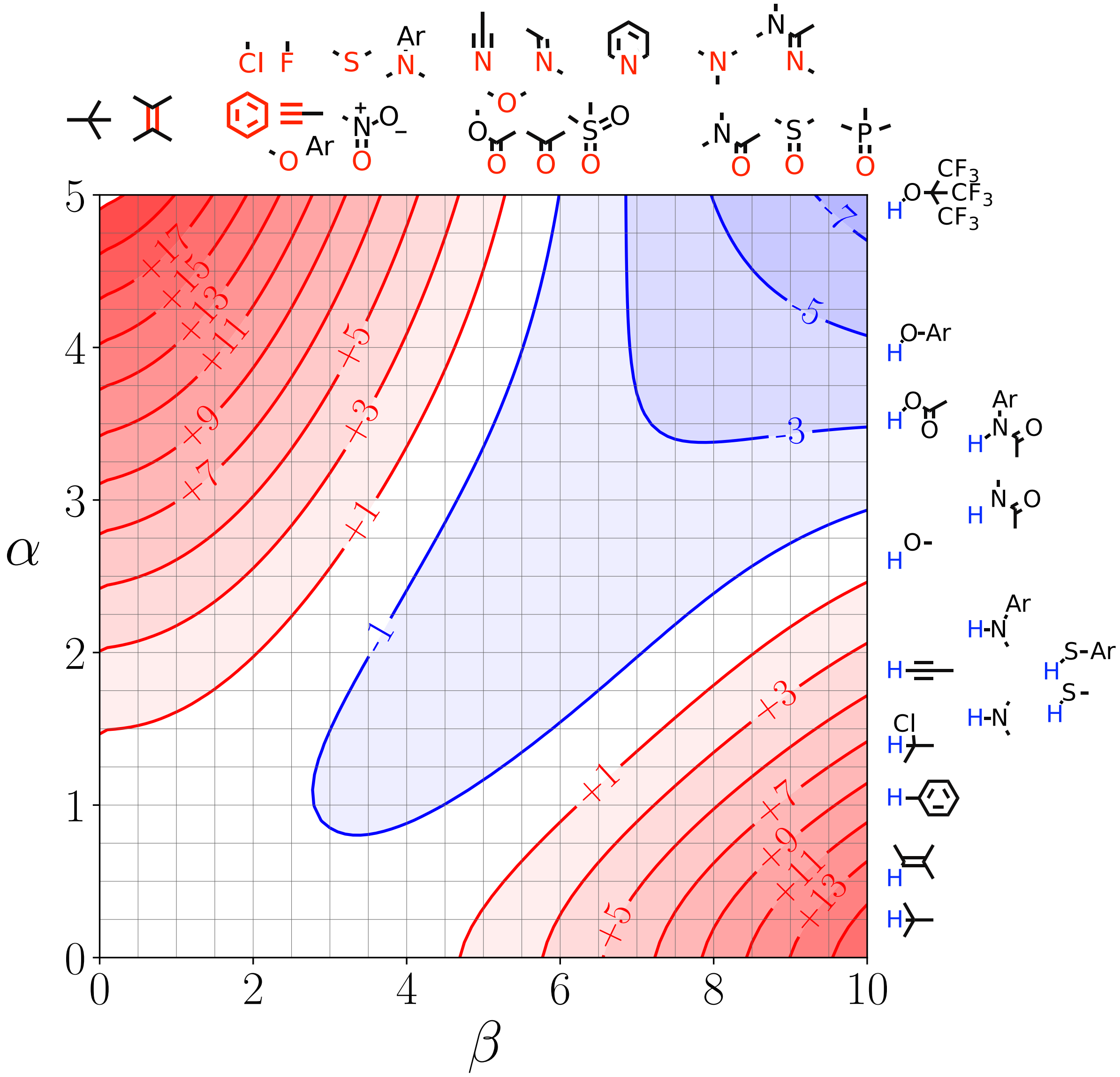

Figure S55: FGIP for (2R,3S)-2,3-butanediol at 298K.

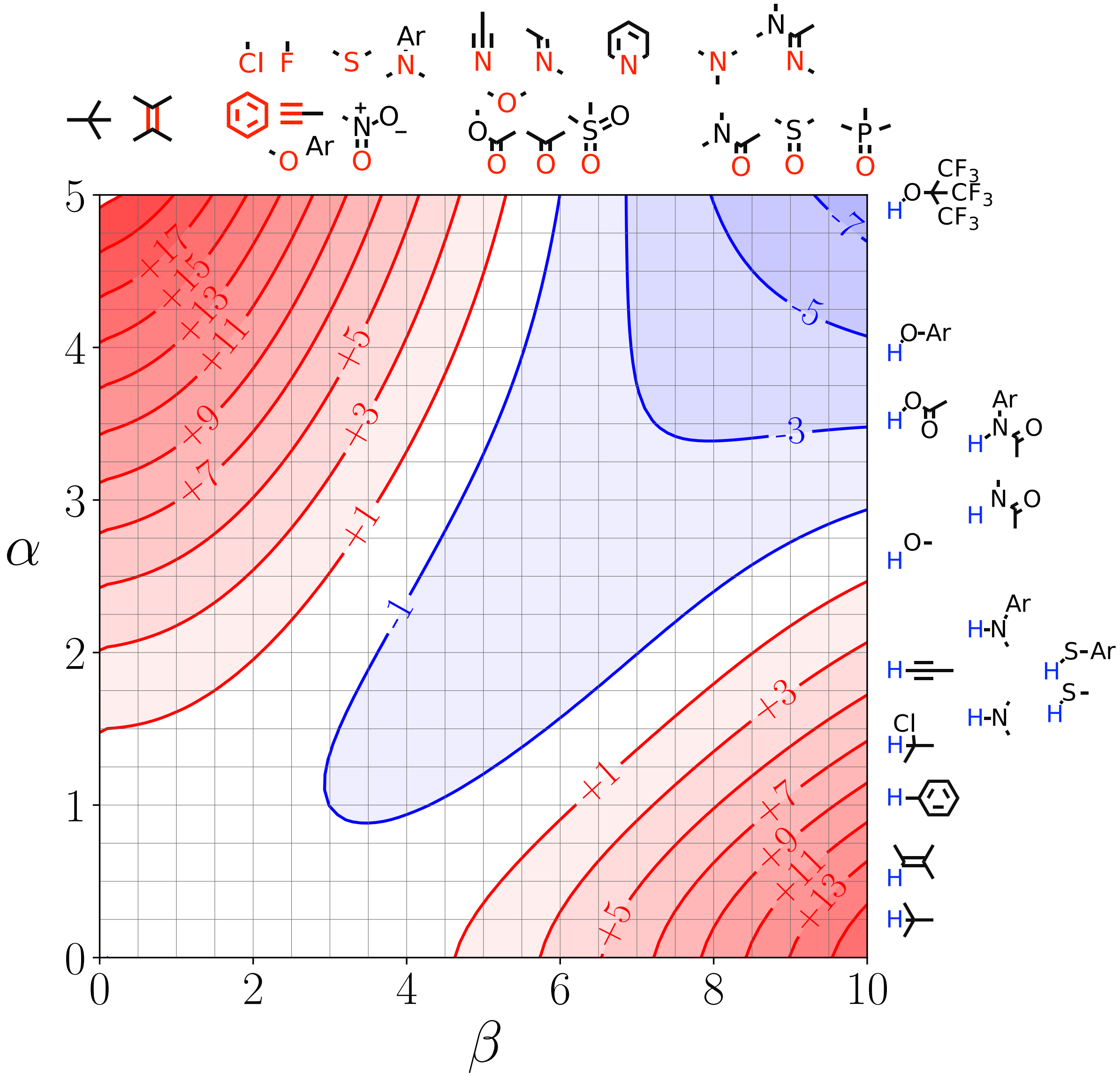

Figure S56: FGIP for 1,4-butanediol at 298K.

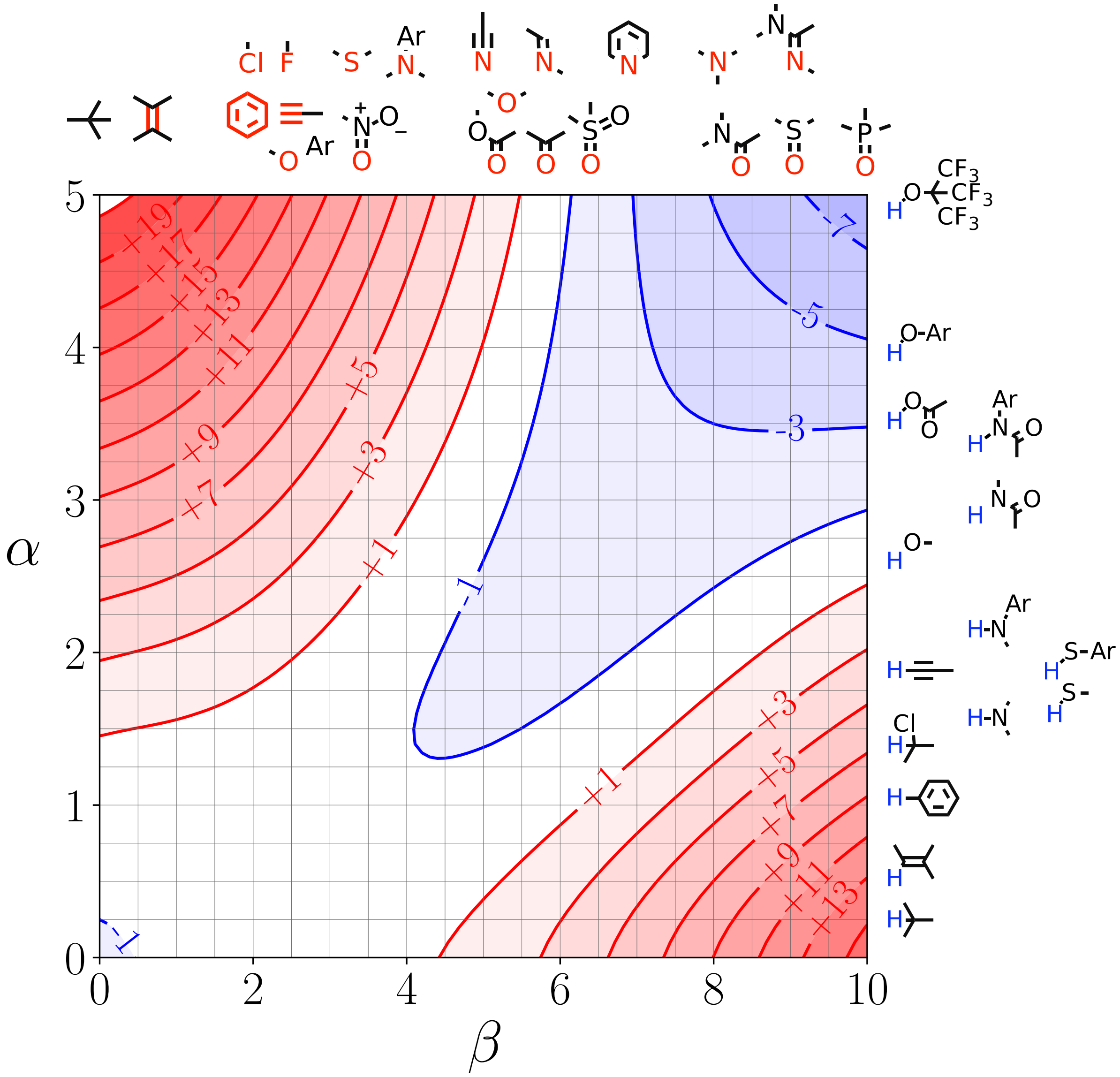

Figure S57: FGIP for 1,5-pentanediol at 298K.

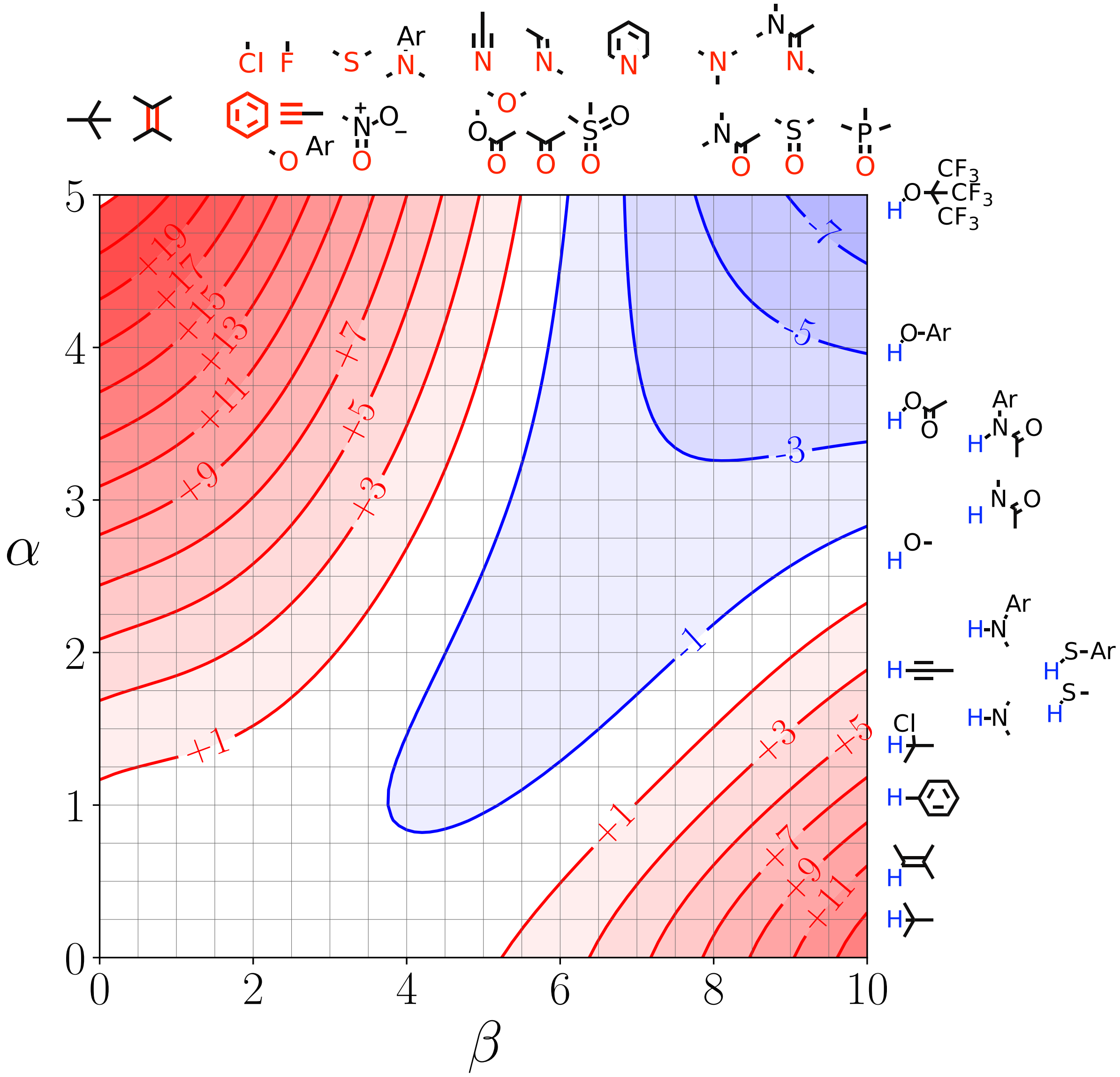

Figure S58: FGIP for diethylene glycol at 298K.

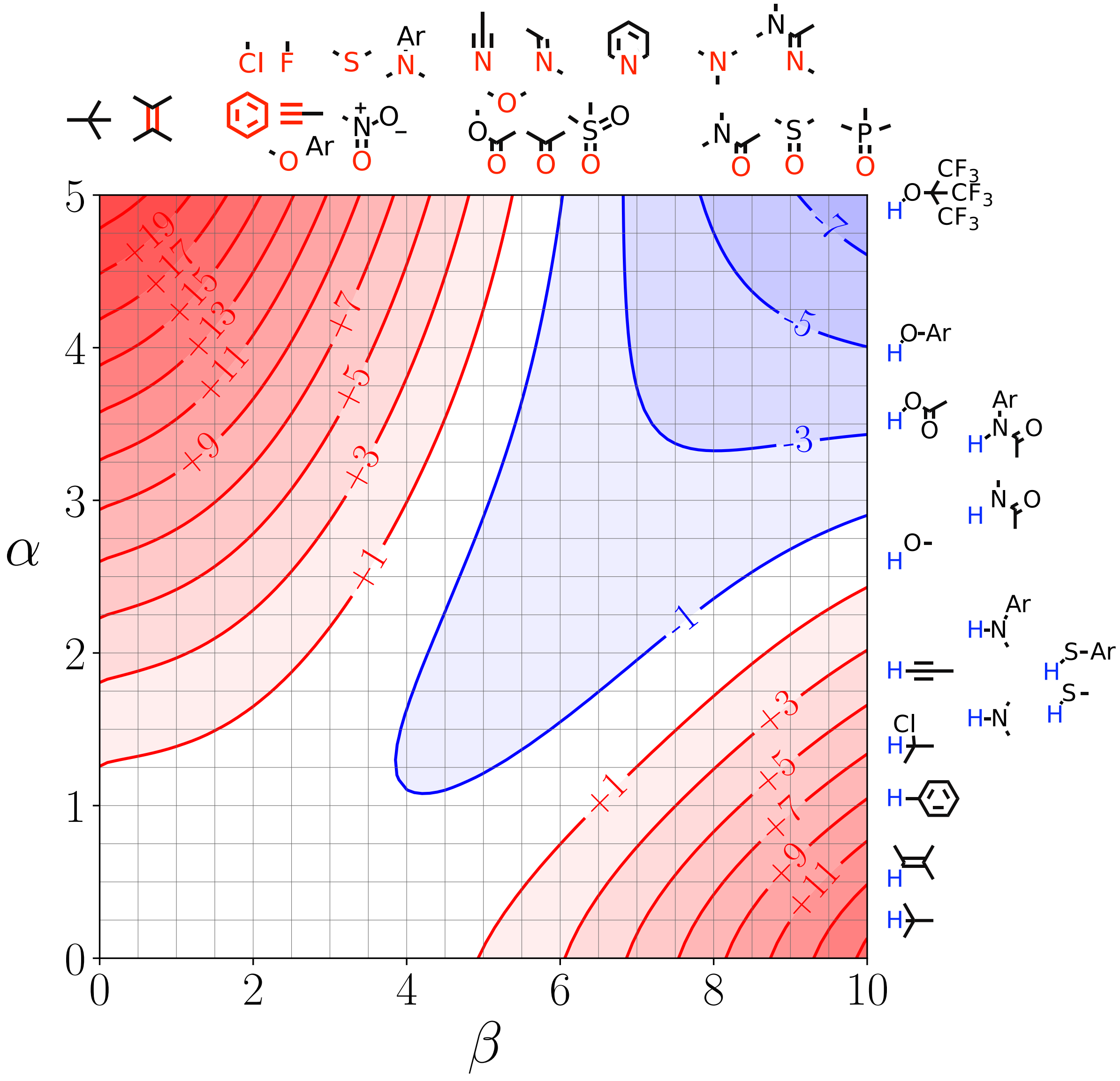

Figure S59: FGIP for triethylene glycol at 298K.

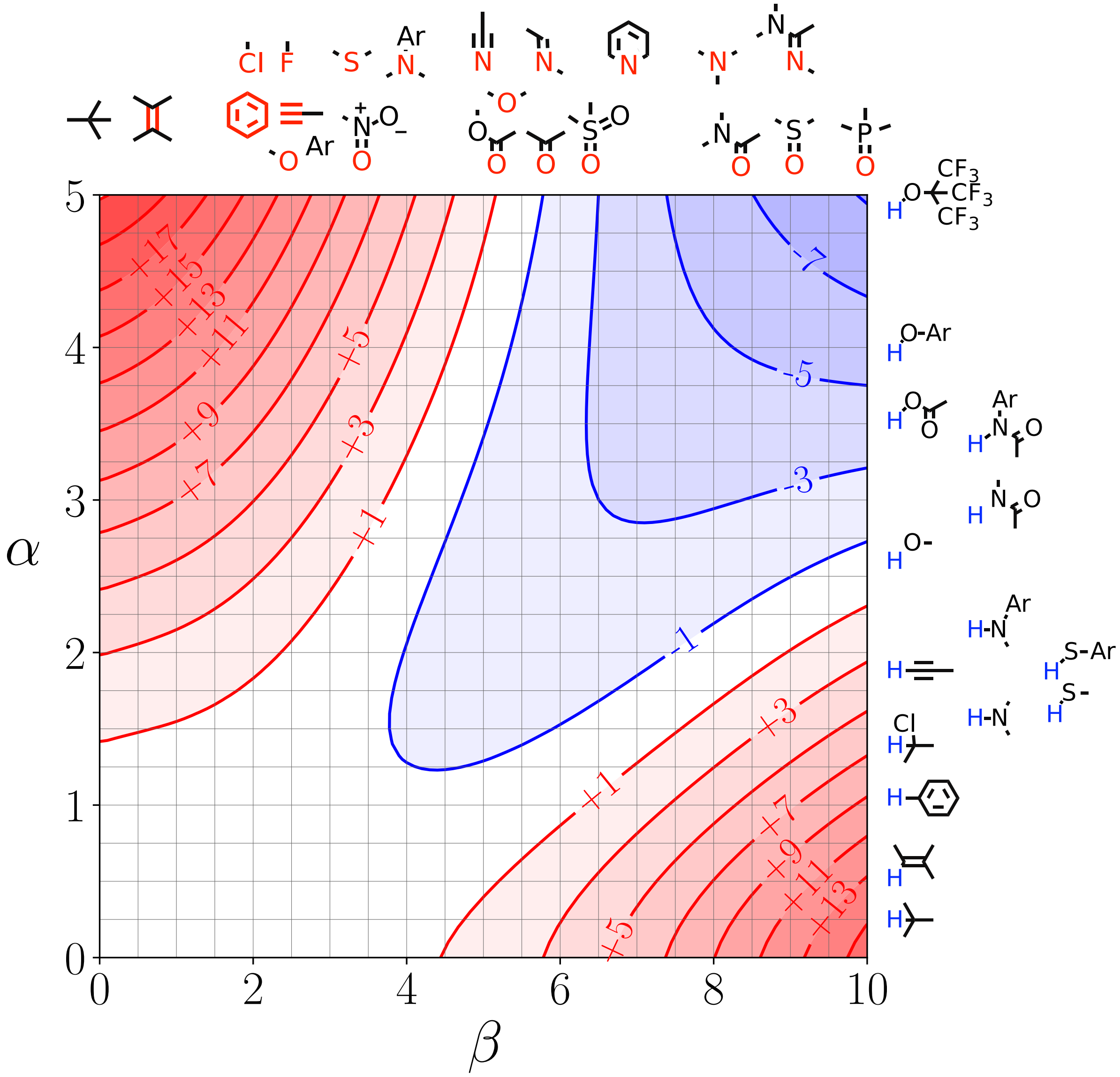

Figure S60: FGIP for glycerol at 298K.

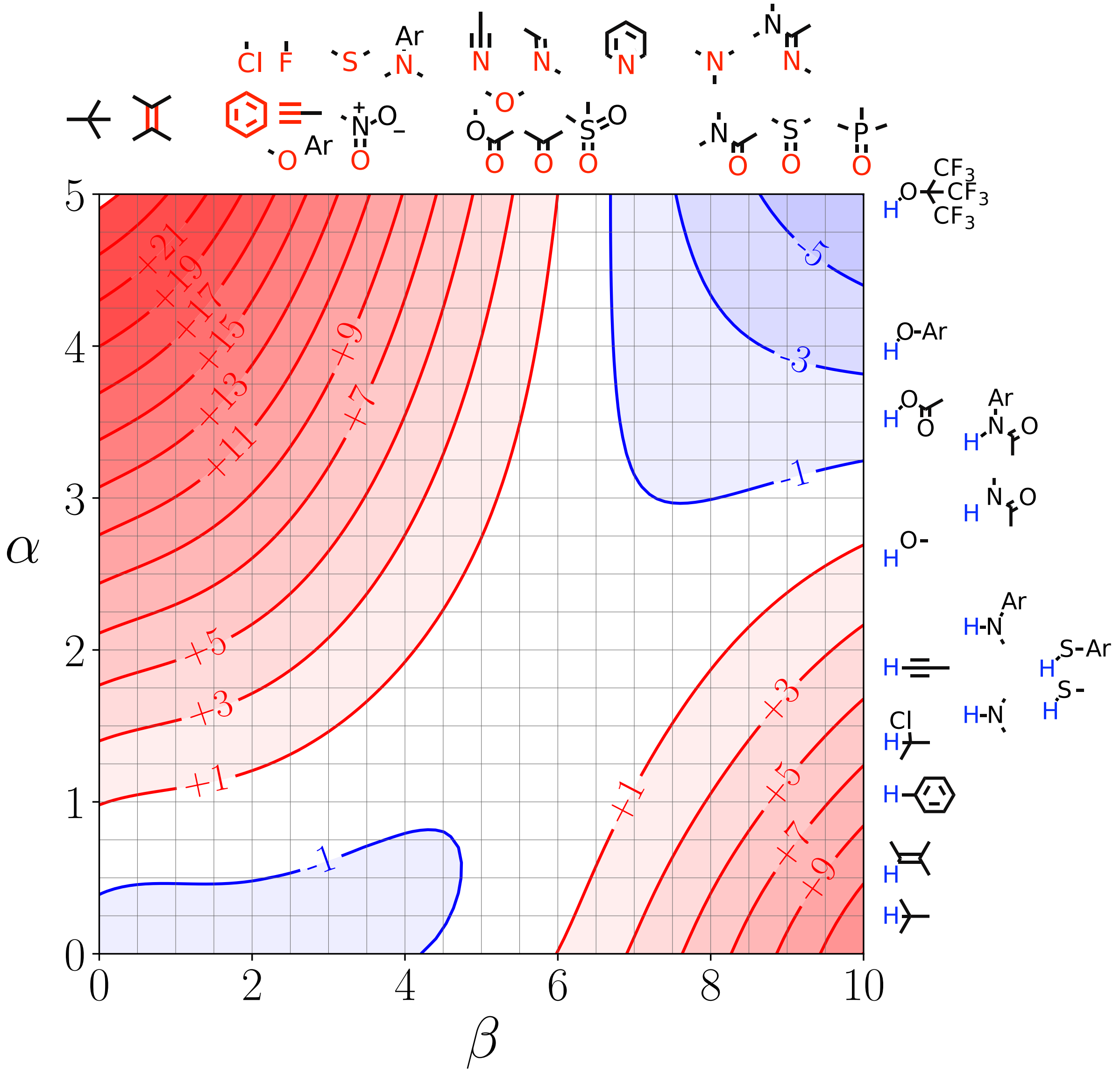

Figure S61: FGIP for phenol at 298K.

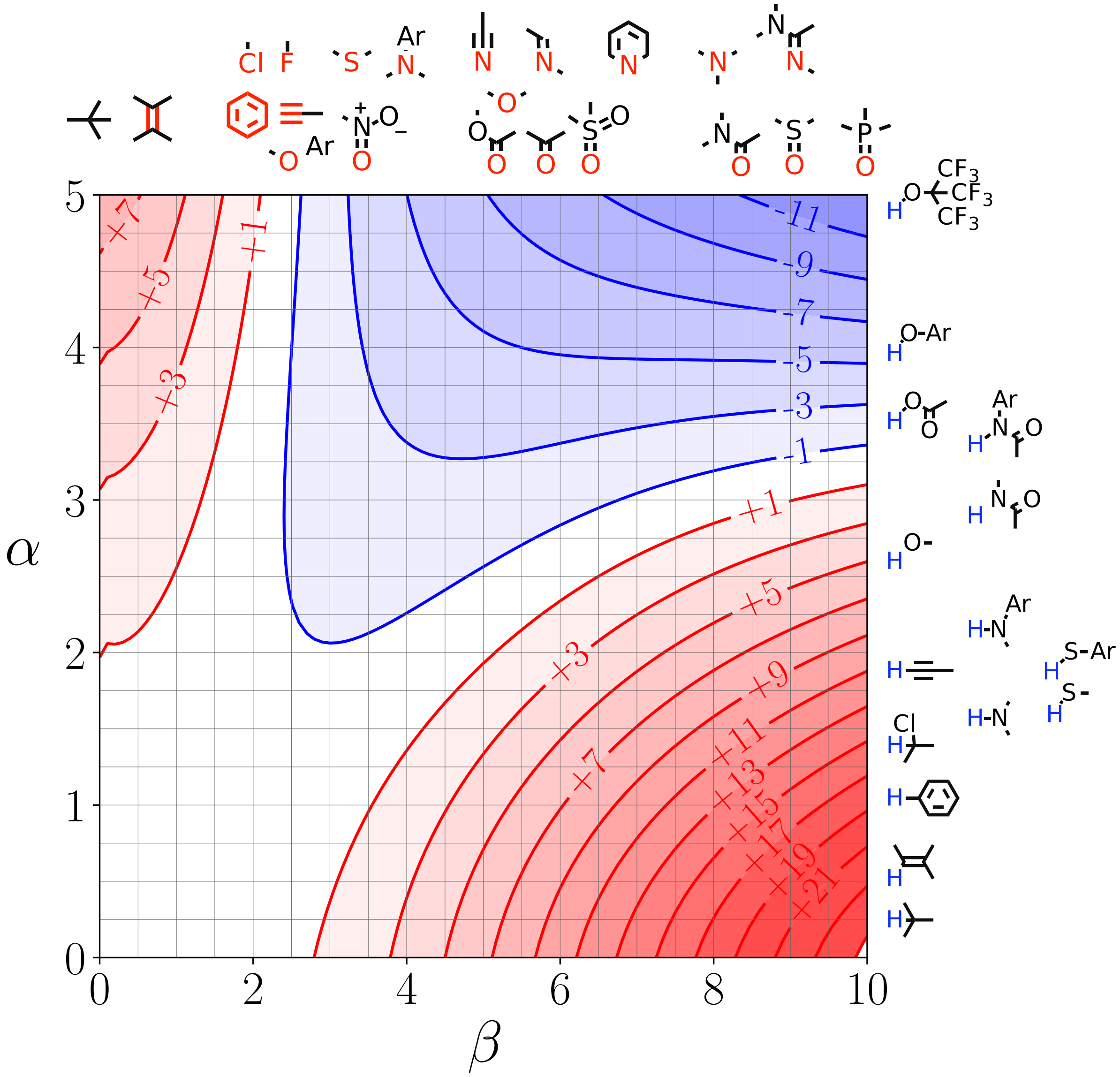

Figure S62: FGIP for ortho-cresol at 298K.

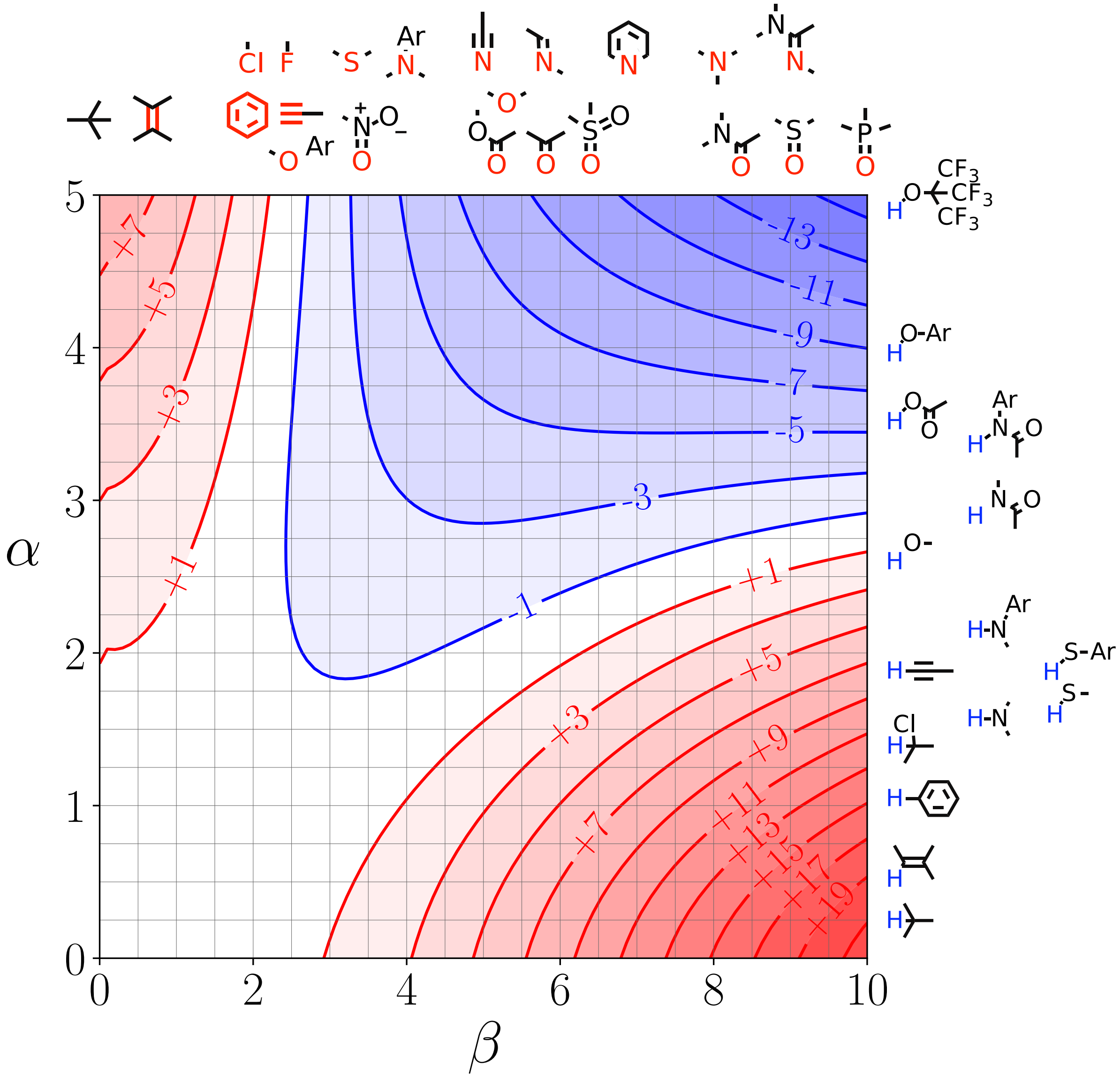

Figure S63: FGIP for meta-cresol at 298K.

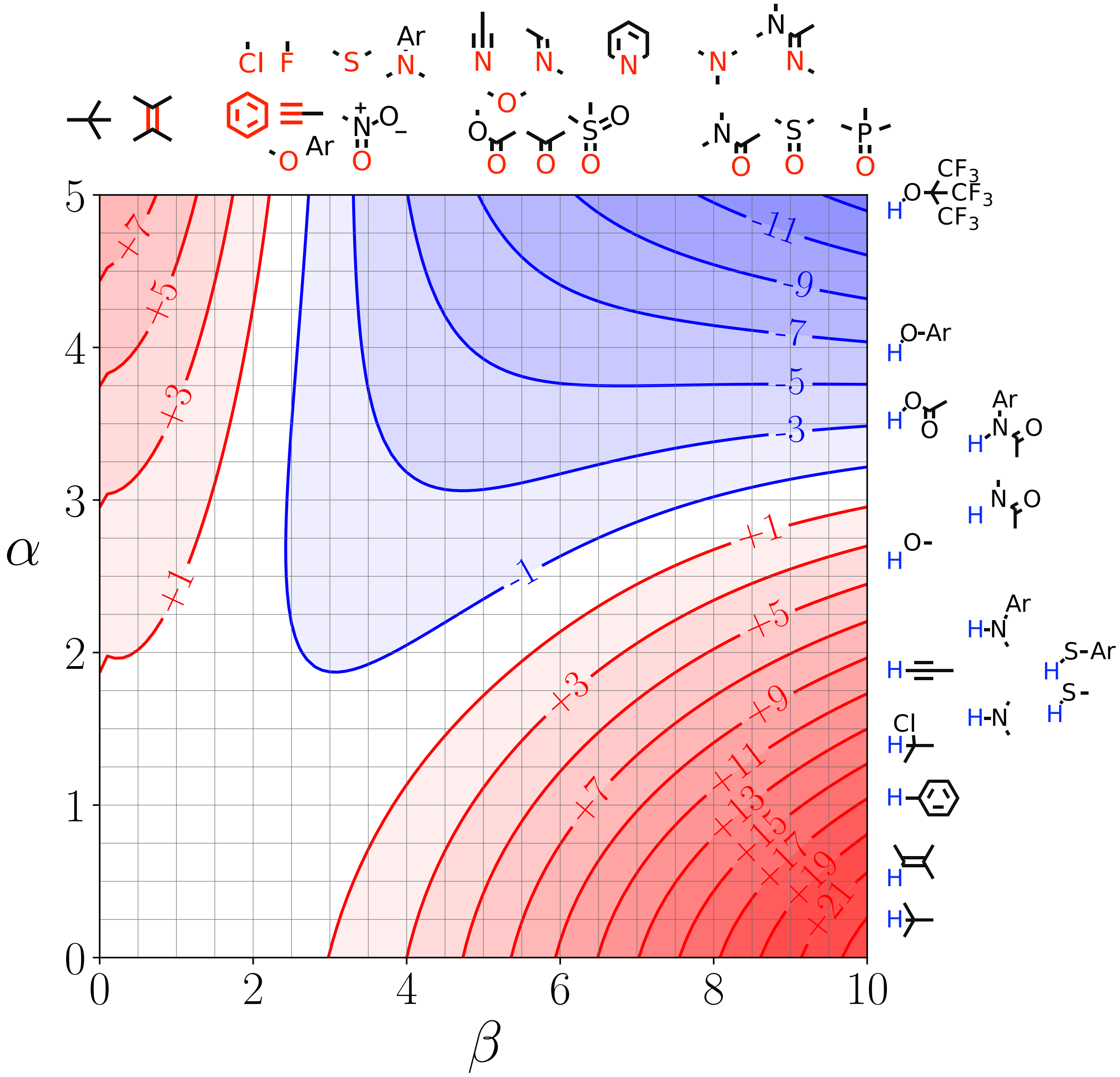

Figure S64: FGIP for para-cresol at 298K.

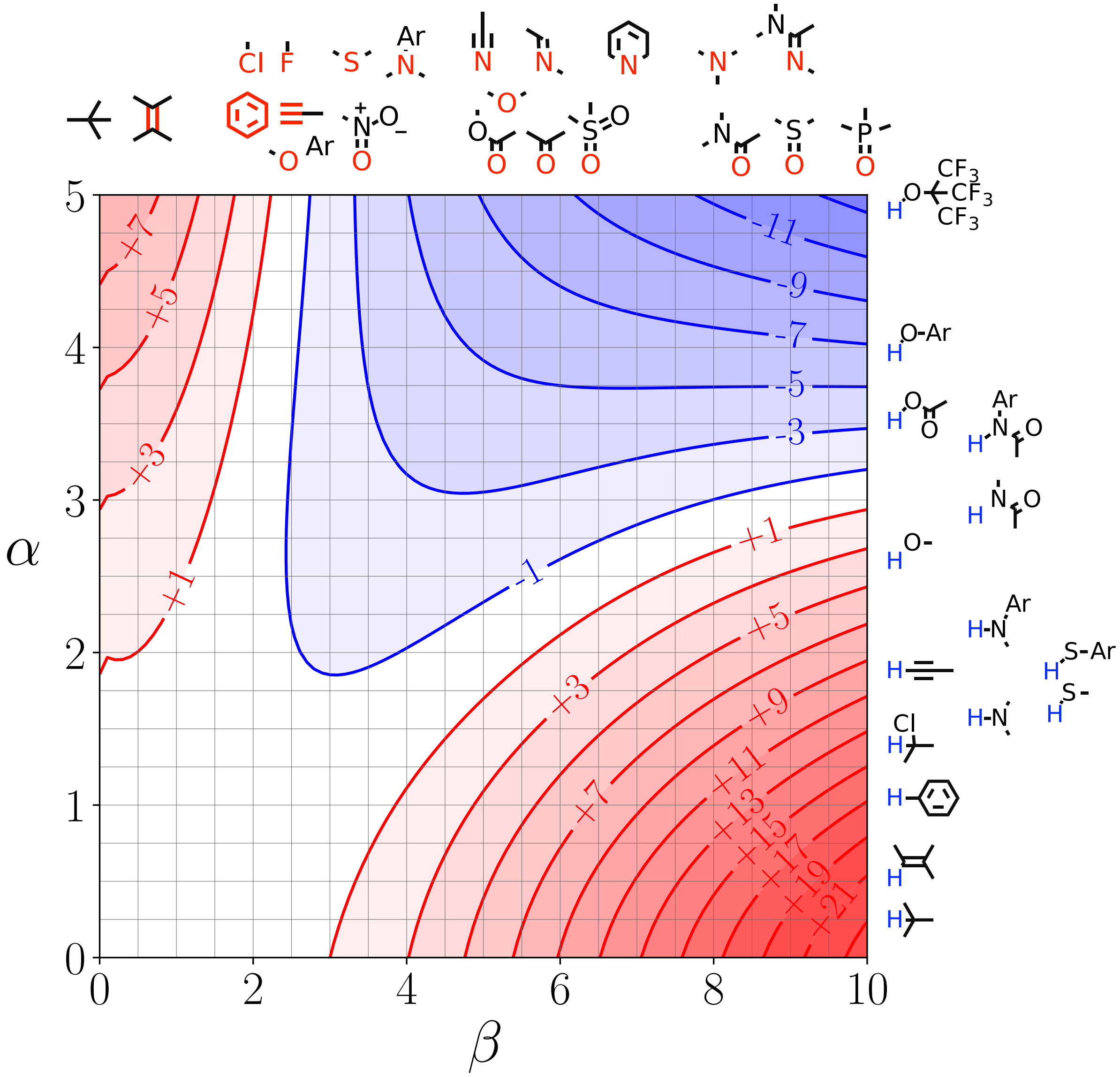

Figure S65: FGIP for 2-methoxyphenol at 298K.

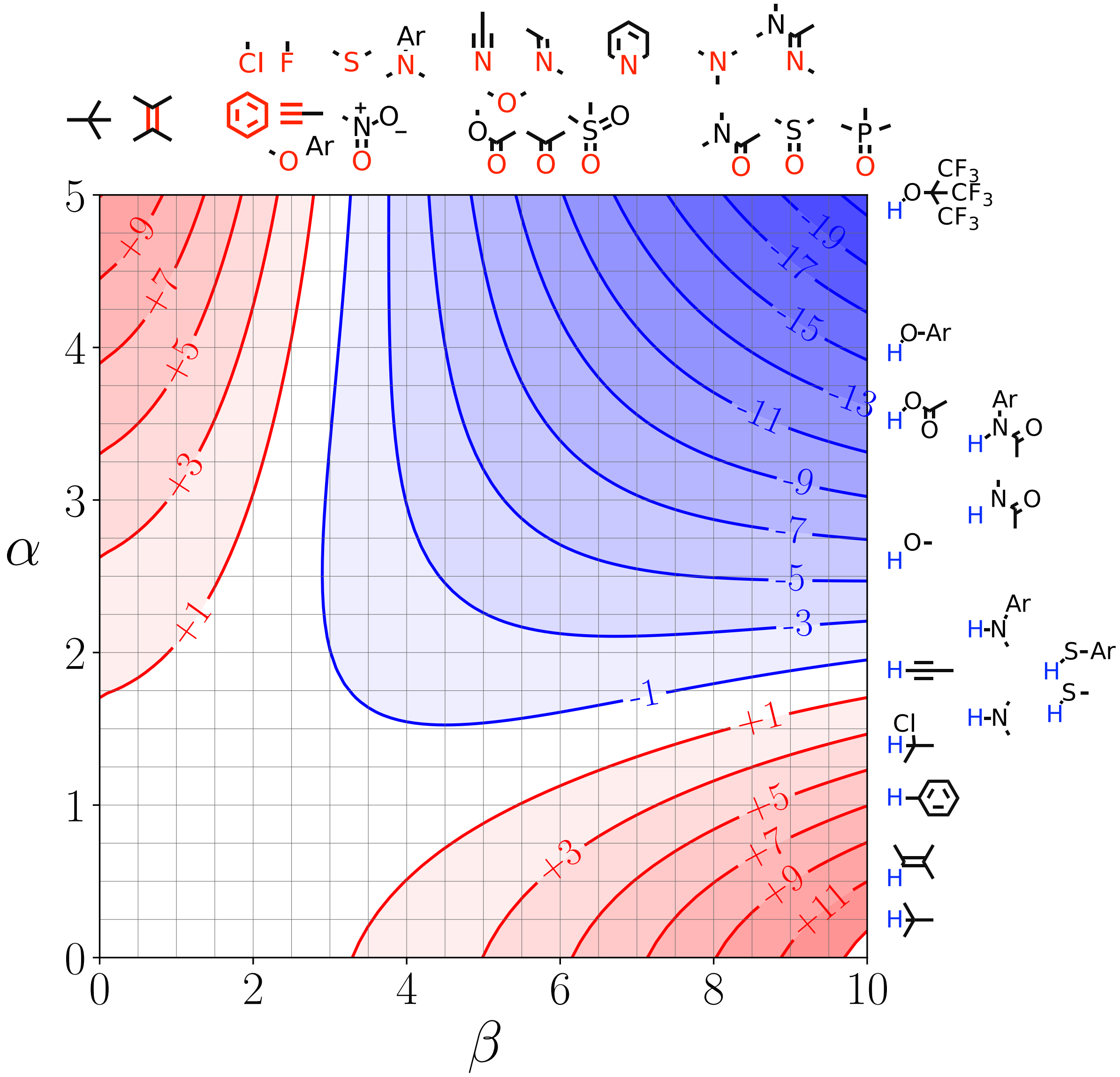

Figure S66: FGIP for 2,4-dimethylphenol at 298K.

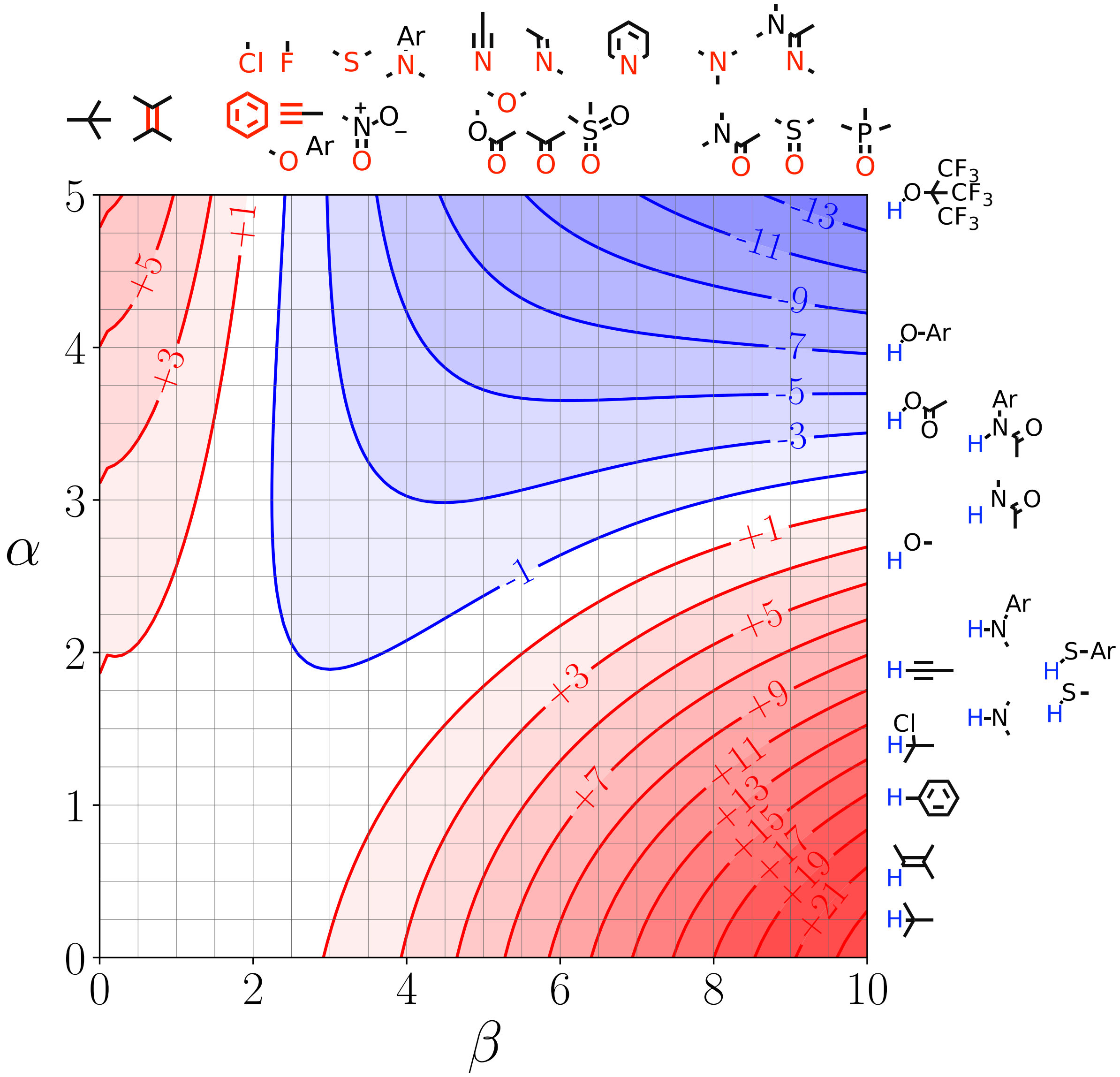

Figure S67: FGIP for 3-chlorophenol at 298K.

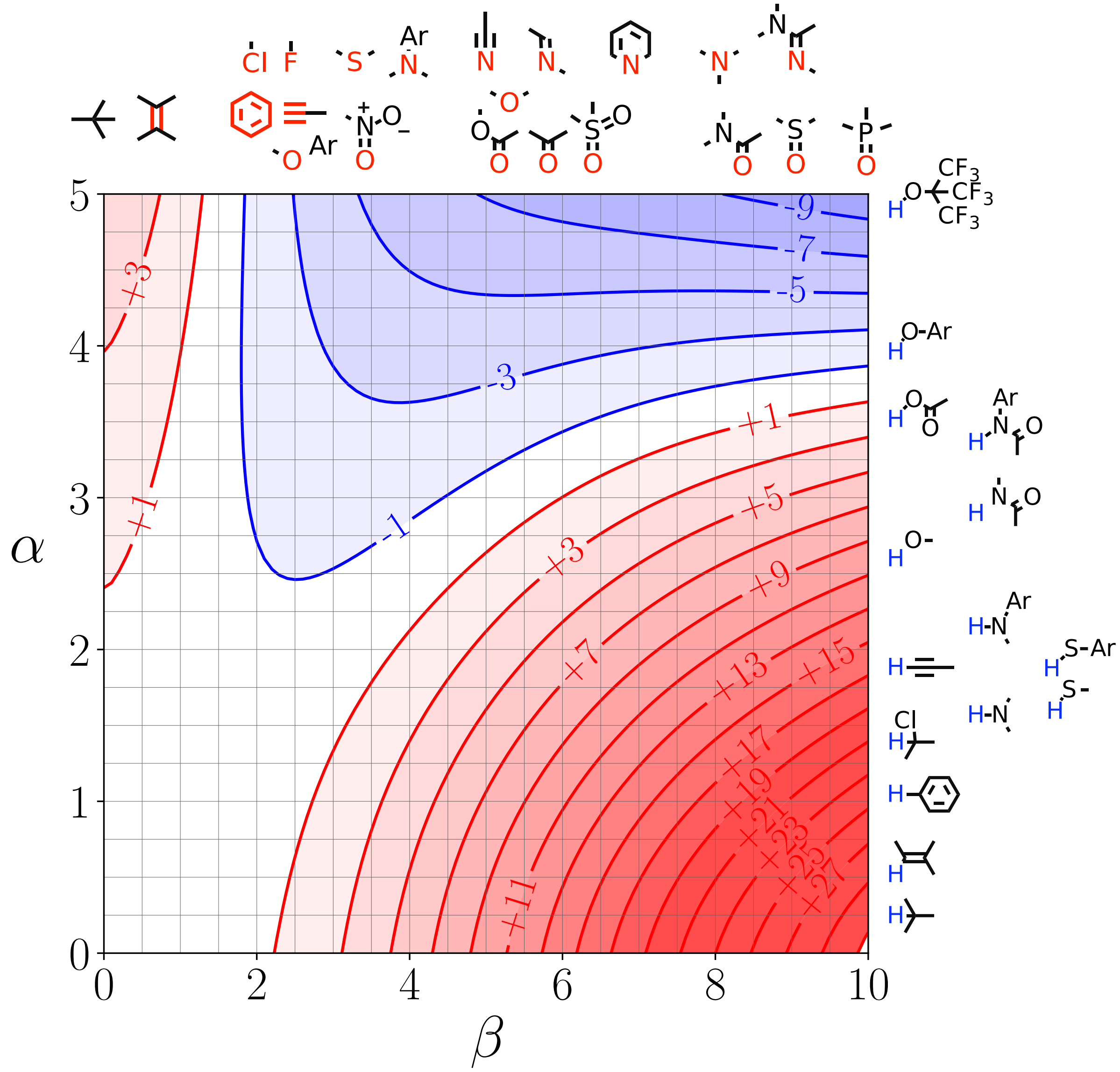

Figure S68: FGIP for diethyl ether at 298K.

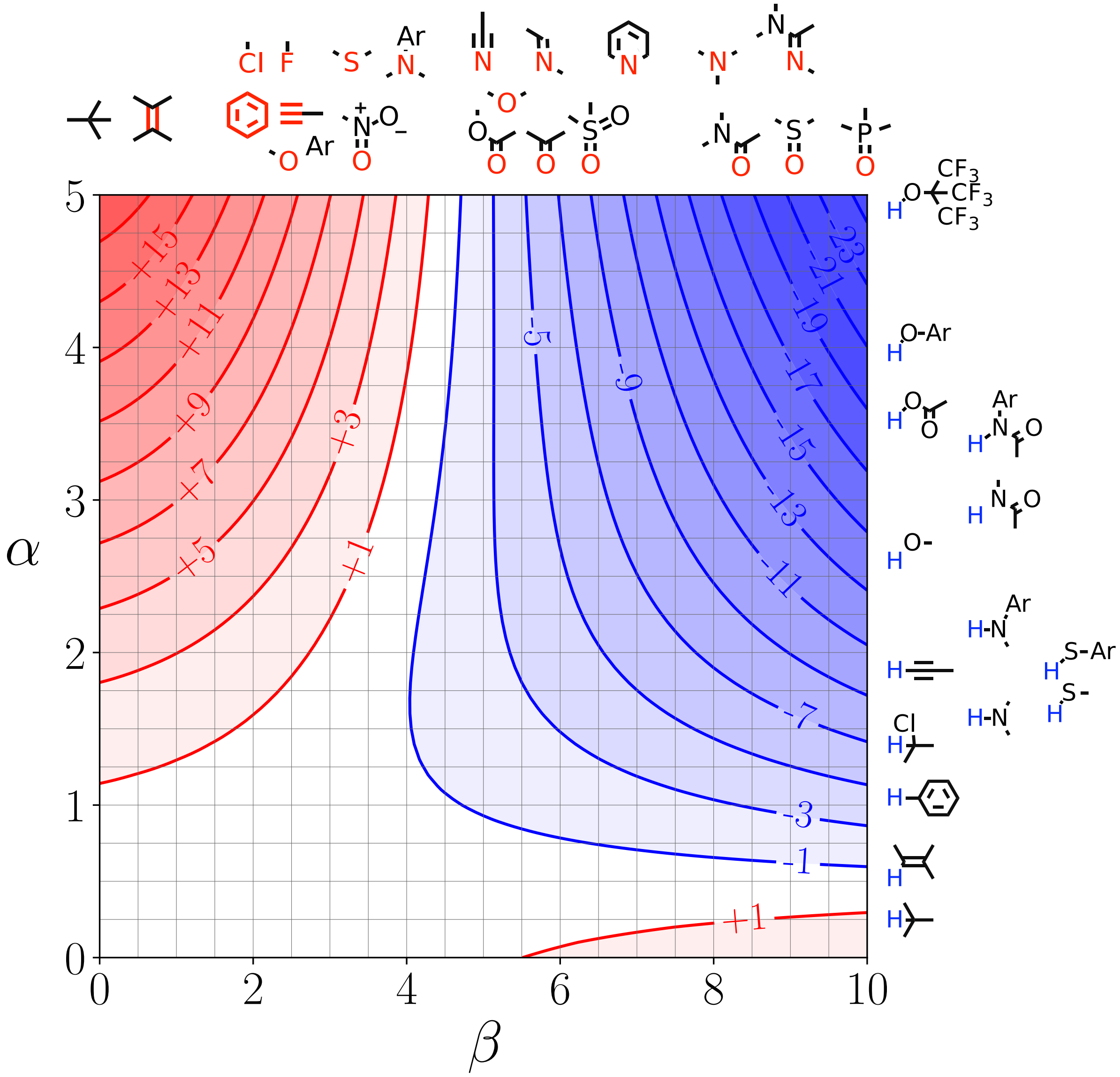

Figure S69: FGIP for di-n-propyl ether at 298K.

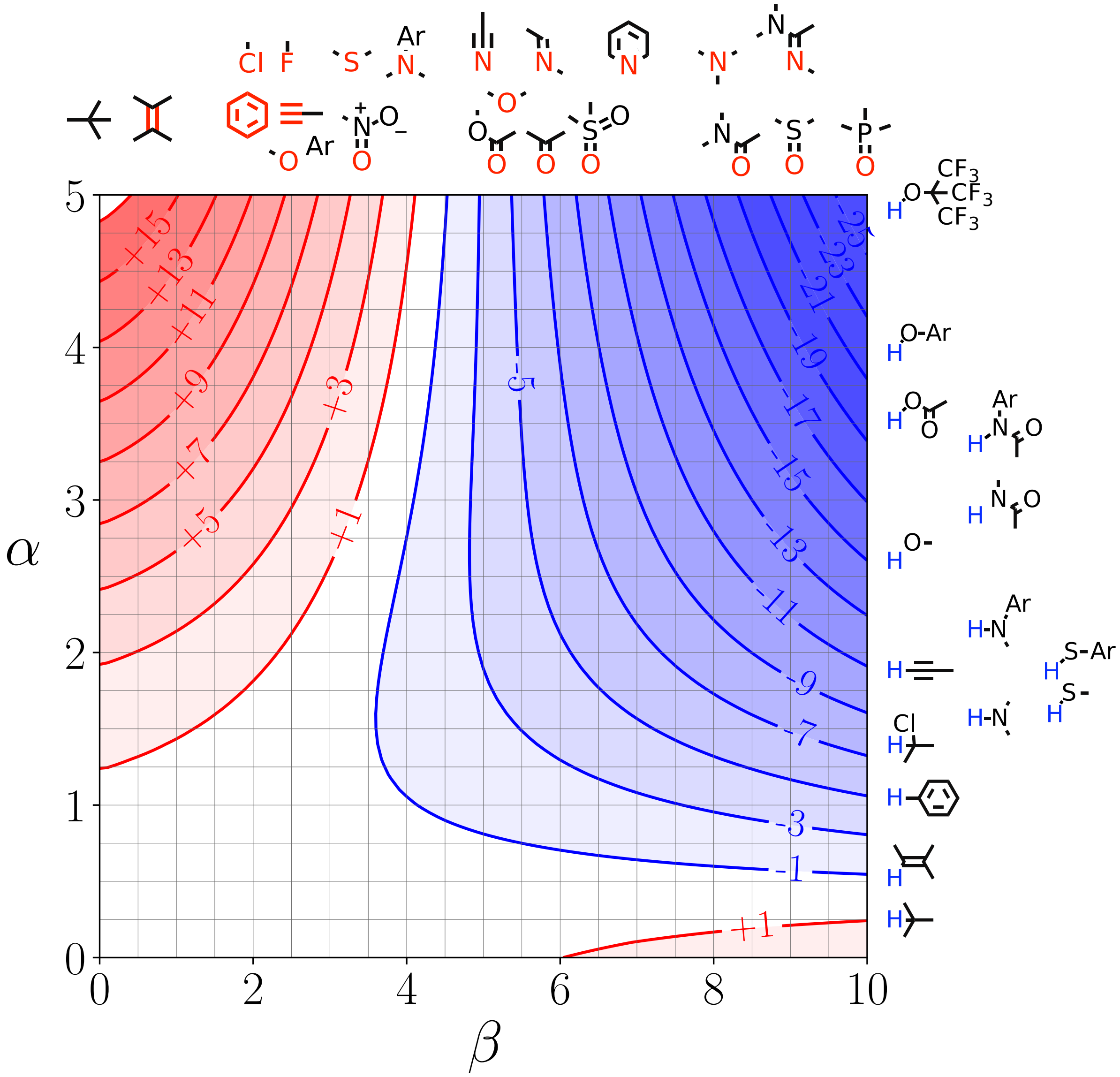

Figure S70: FGIP for diisopropyl ether at 298K.

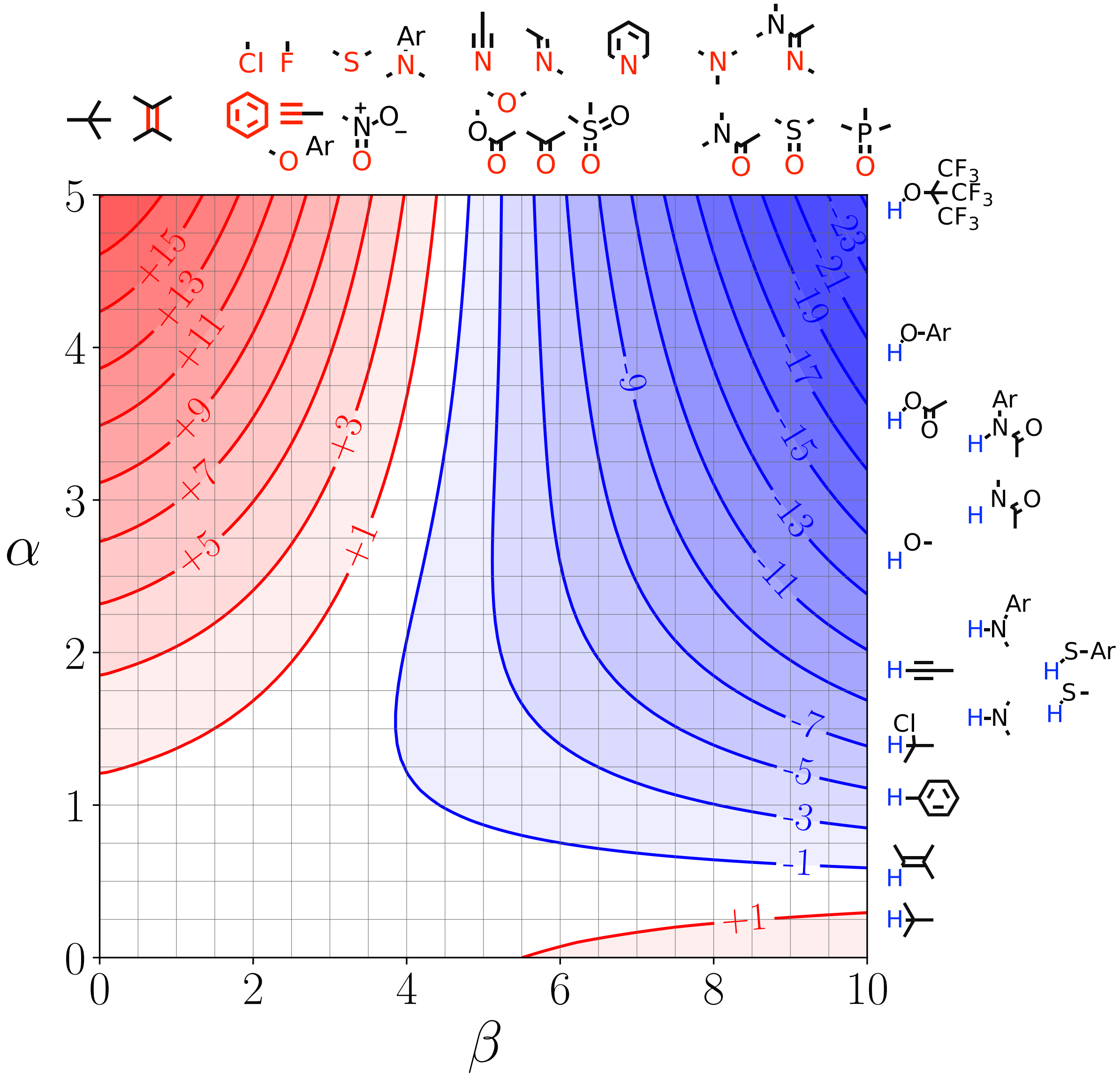

Figure S71: FGIP for dibutyl ether at 298K.

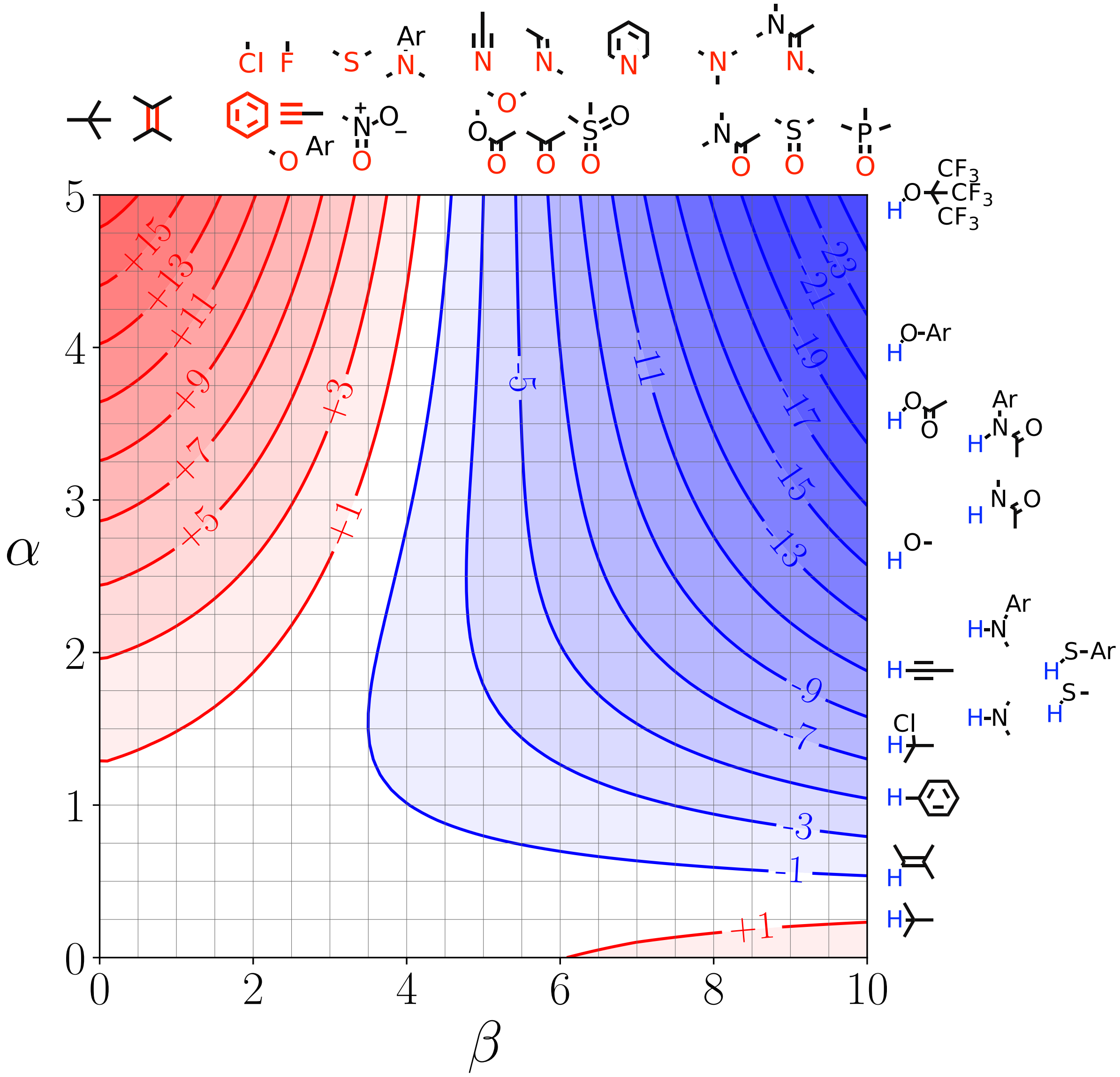

Figure S72: FGIP for bis(2-chloroethyl) ether at 298K.

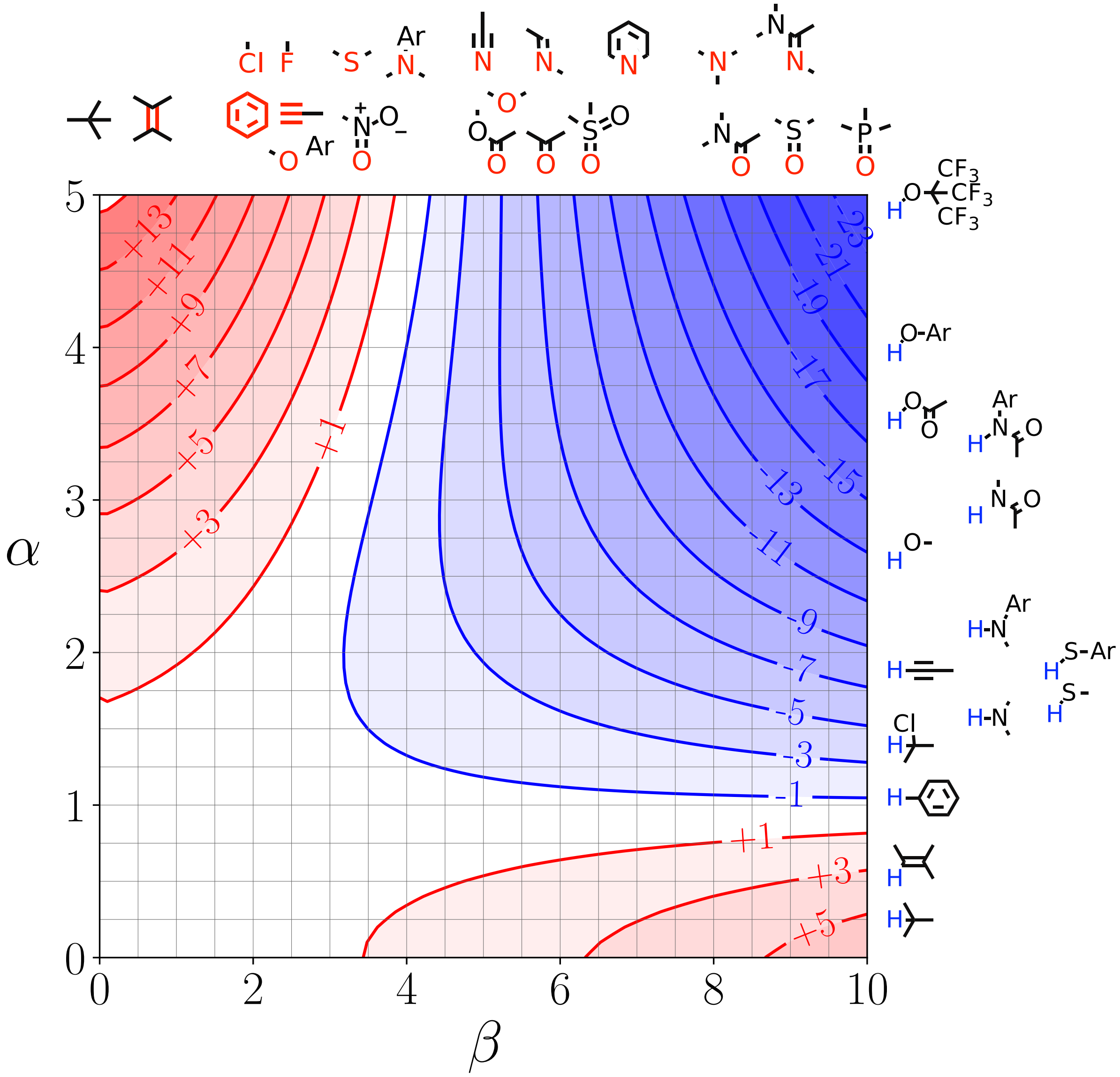

Figure S73: FGIP for 1,2-dimethyoxyethane at 298K.

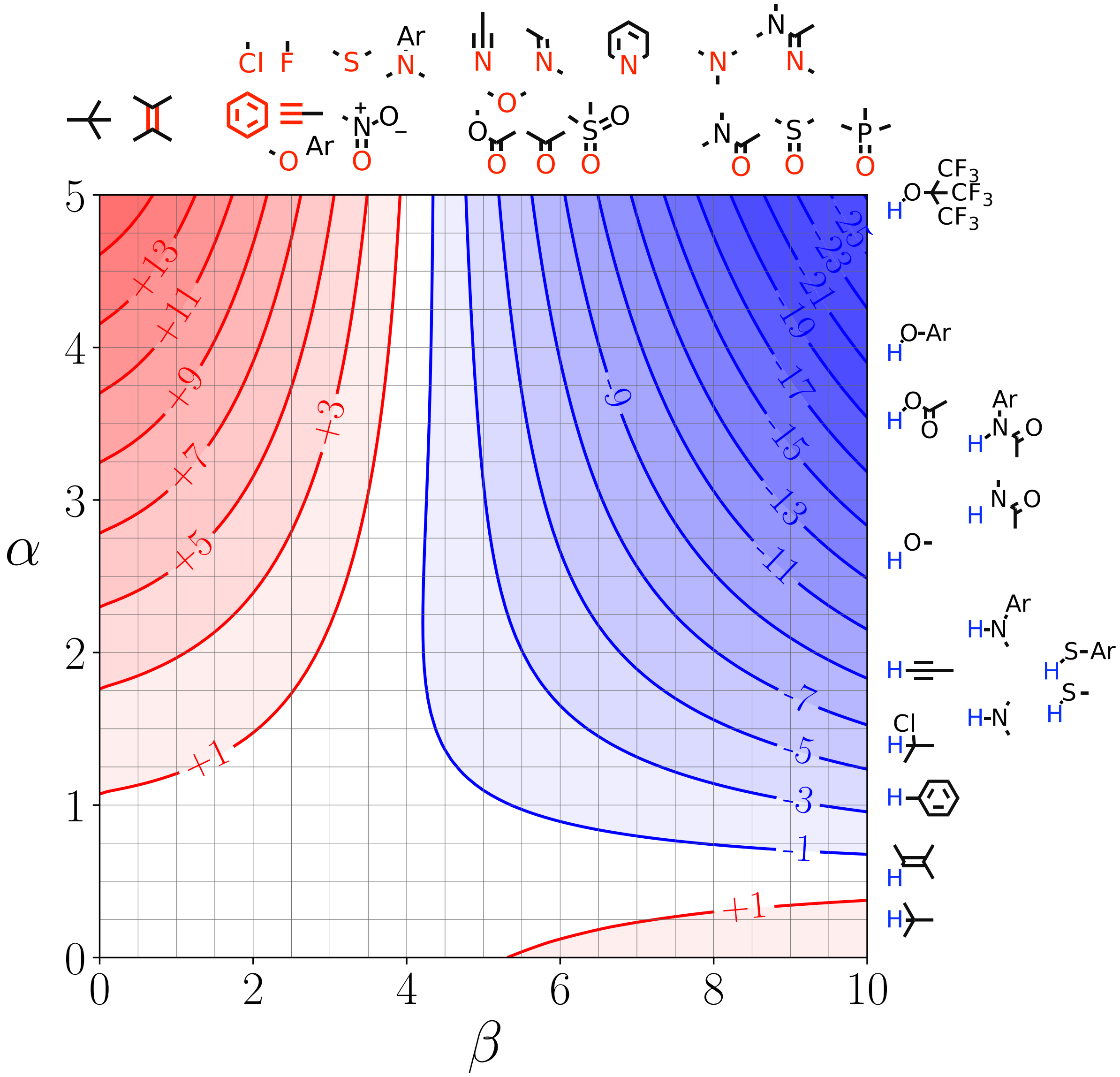

Figure S74: FGIP for diethylene glycol dimethyl ether at 298K.

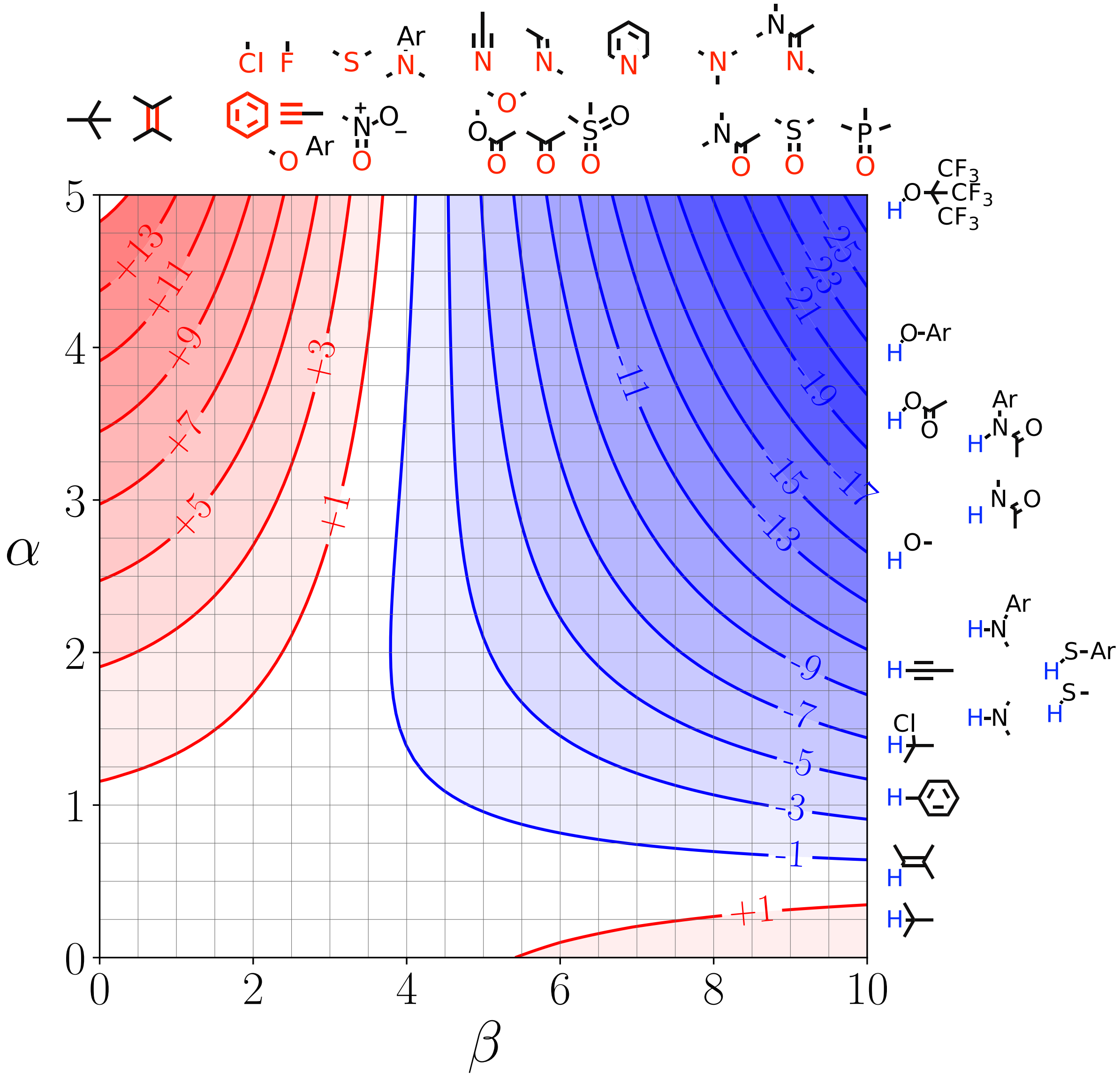

Figure S75: FGIP for furan at 298K.

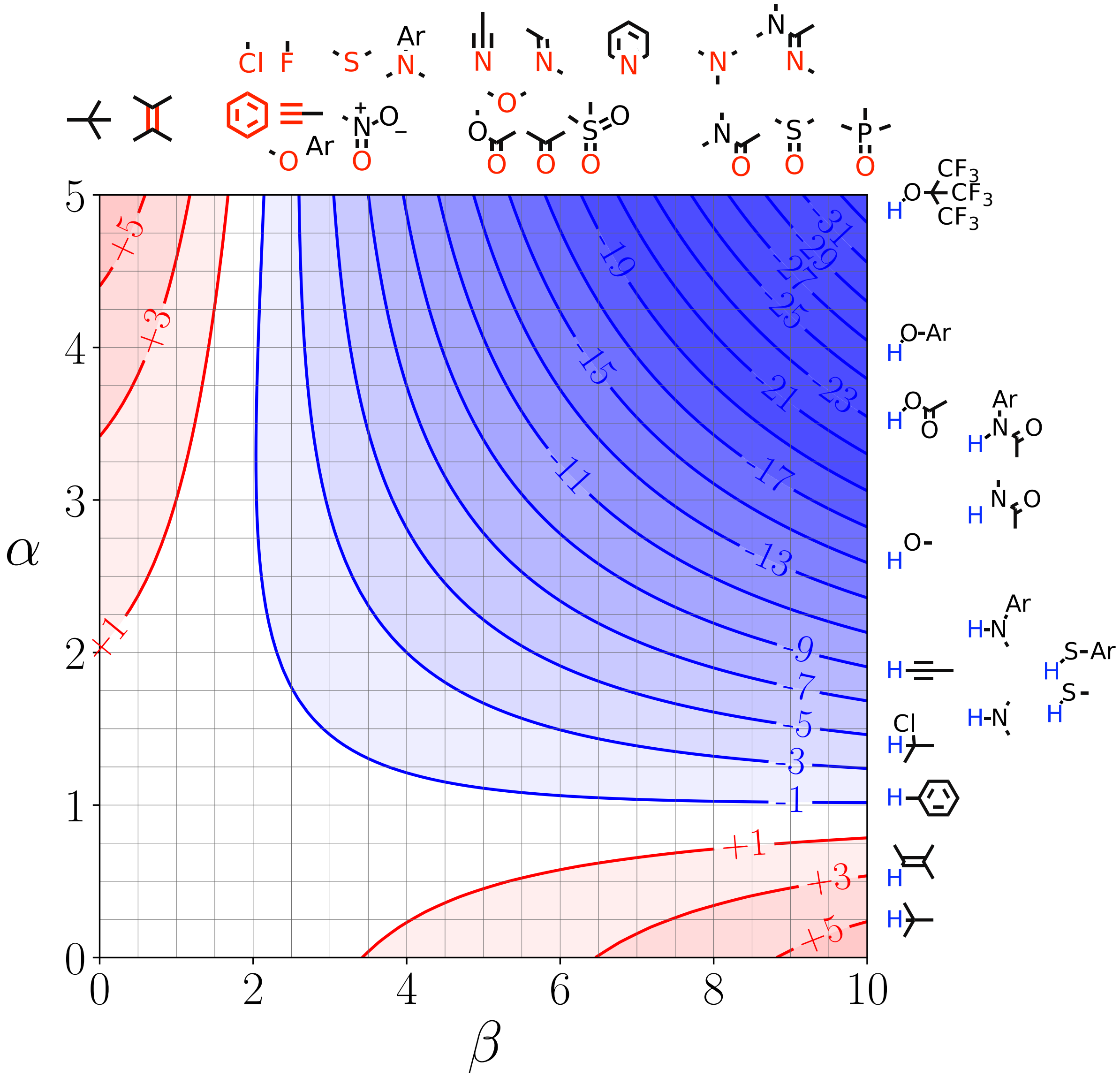

Figure S76: FGIP for tetrahydrofuran at 298K.

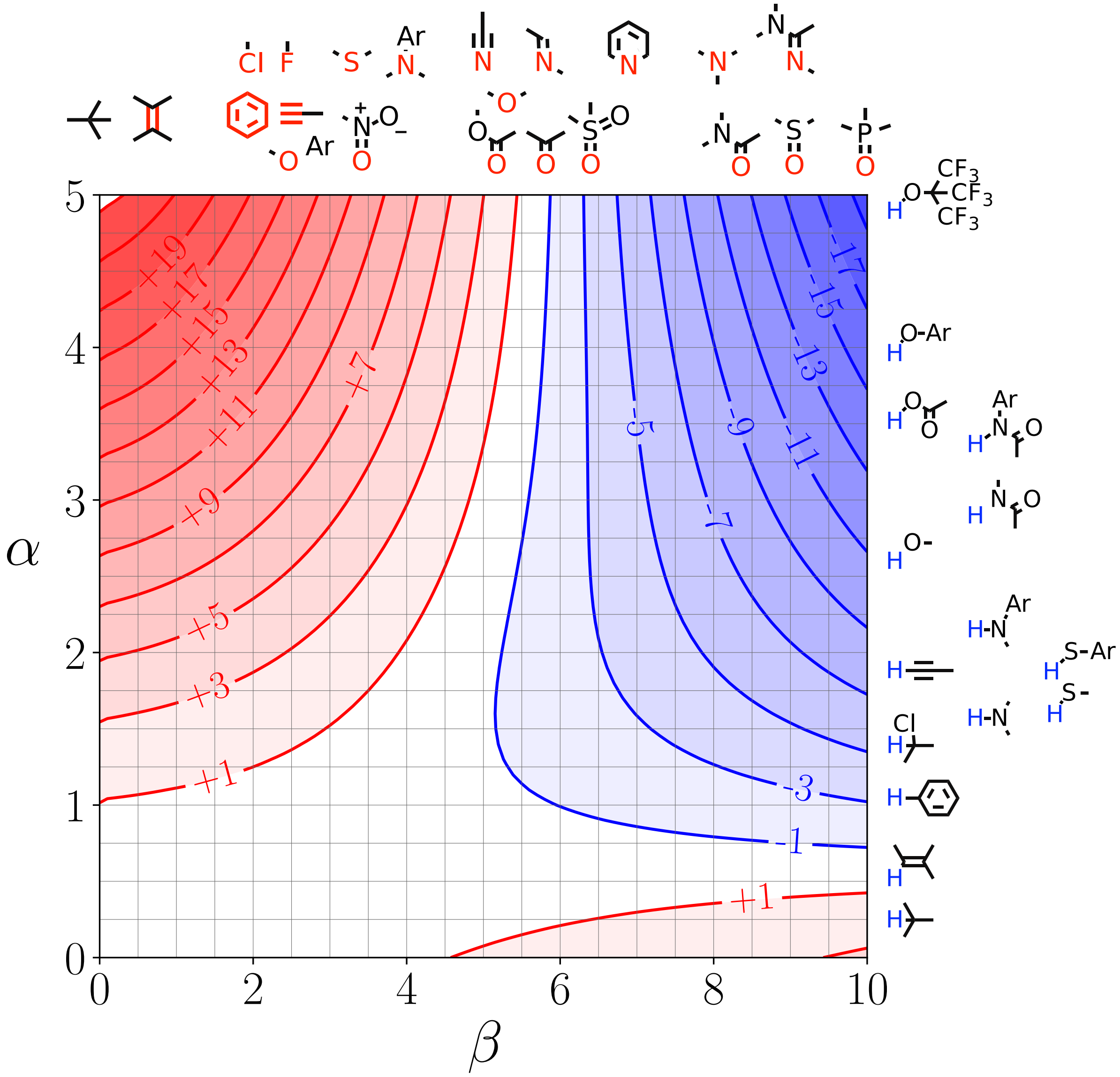

Figure S77: FGIP for 2-methyltetrahydrofuran at 298K.

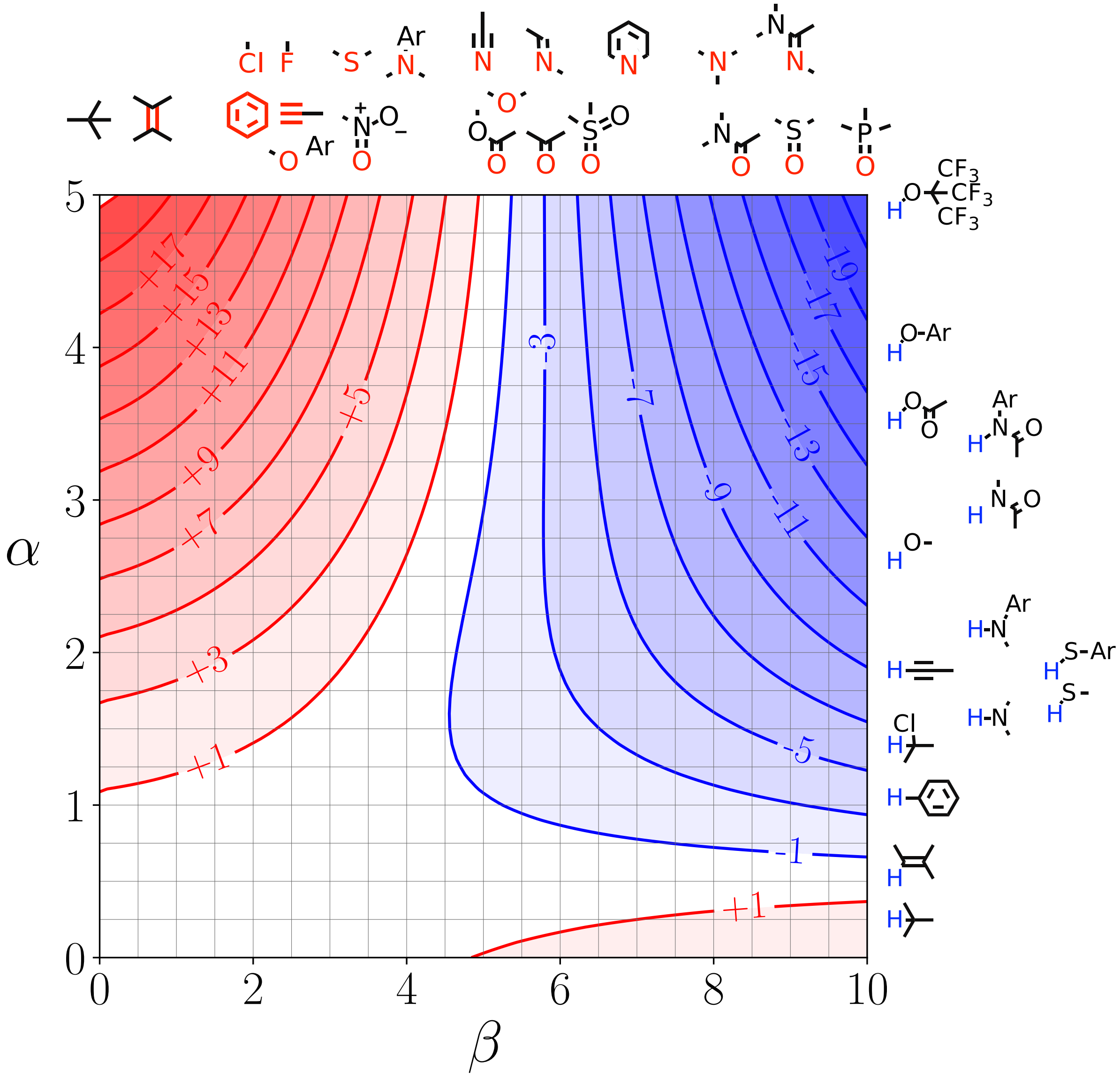

Figure S78: FGIP for tetrahydropyran at 298K.

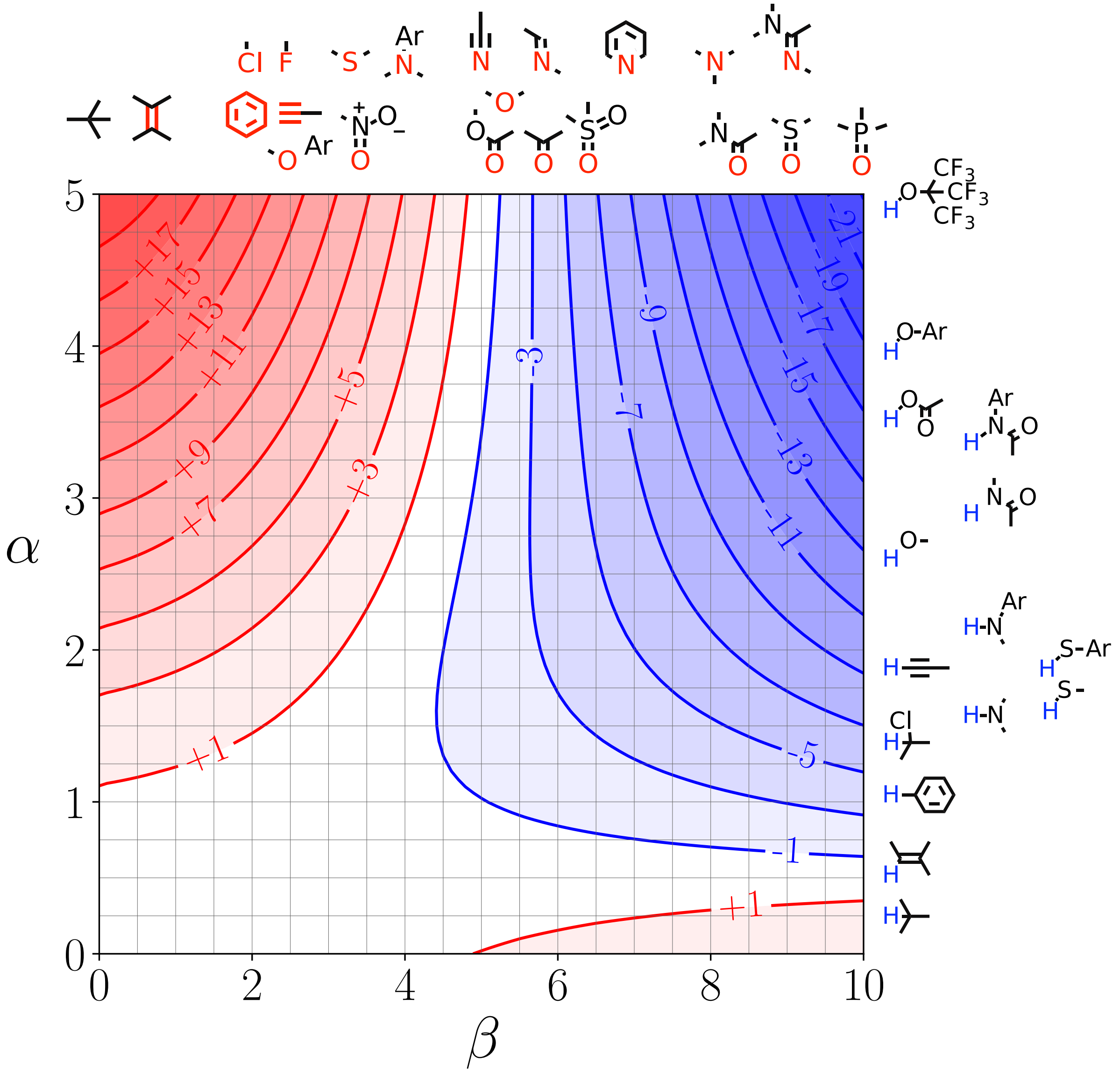

Figure S79: FGIP for 1,3-dioxane at 298K.

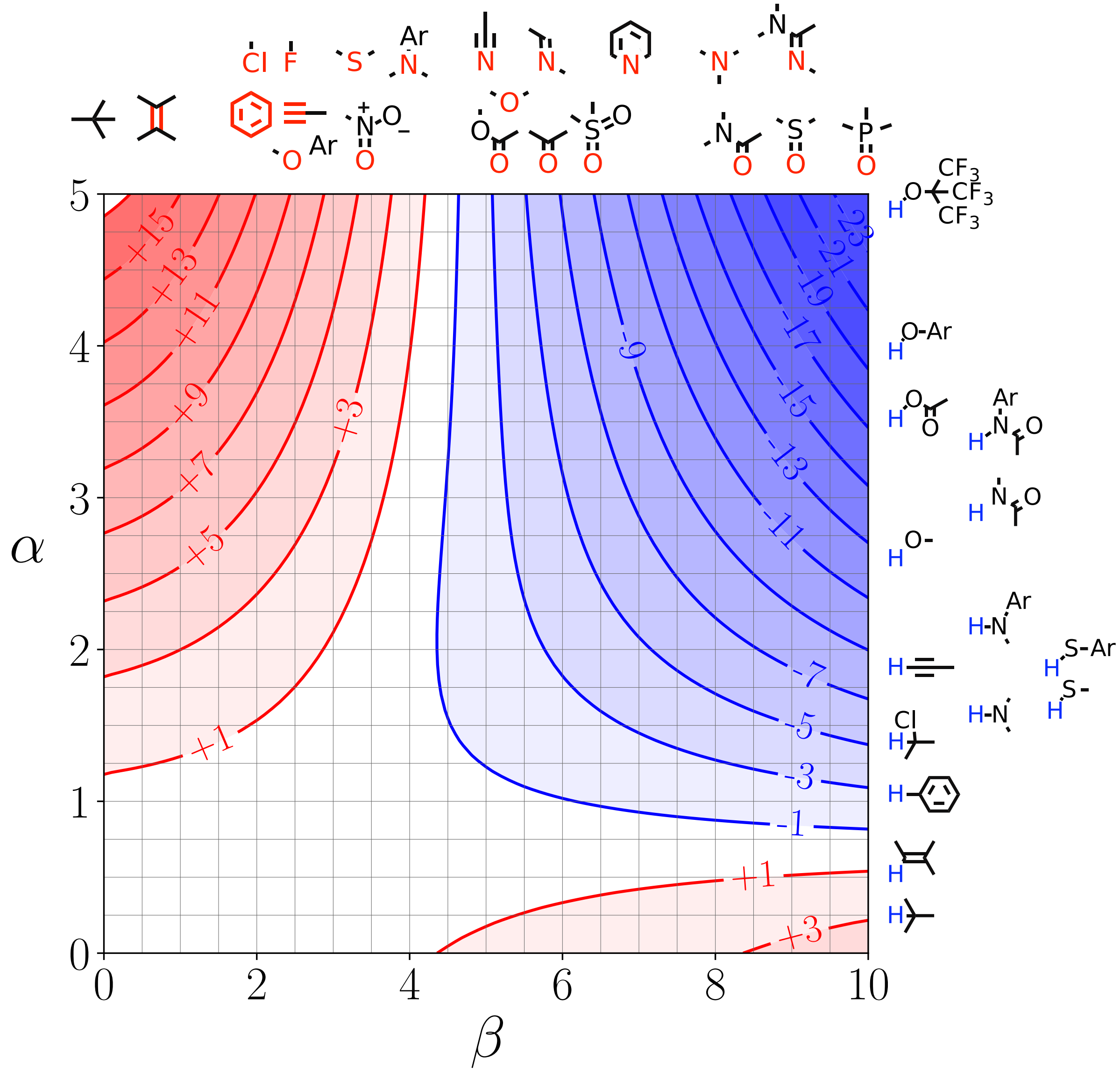

Figure S80: FGIP for 1,3-dioxolan at 298K.

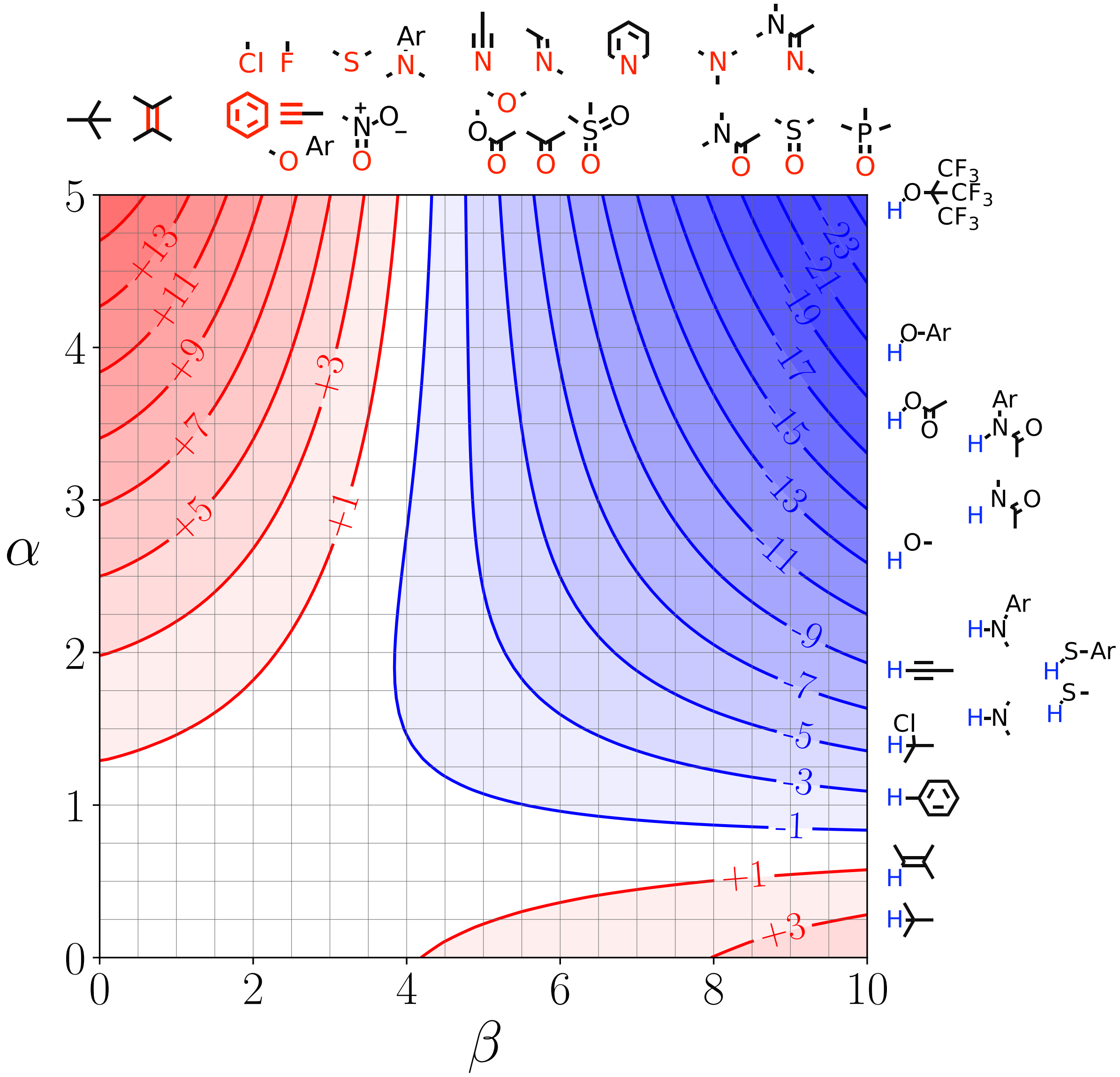

Figure S81: FGIP for 1,8-cineole at 298K.

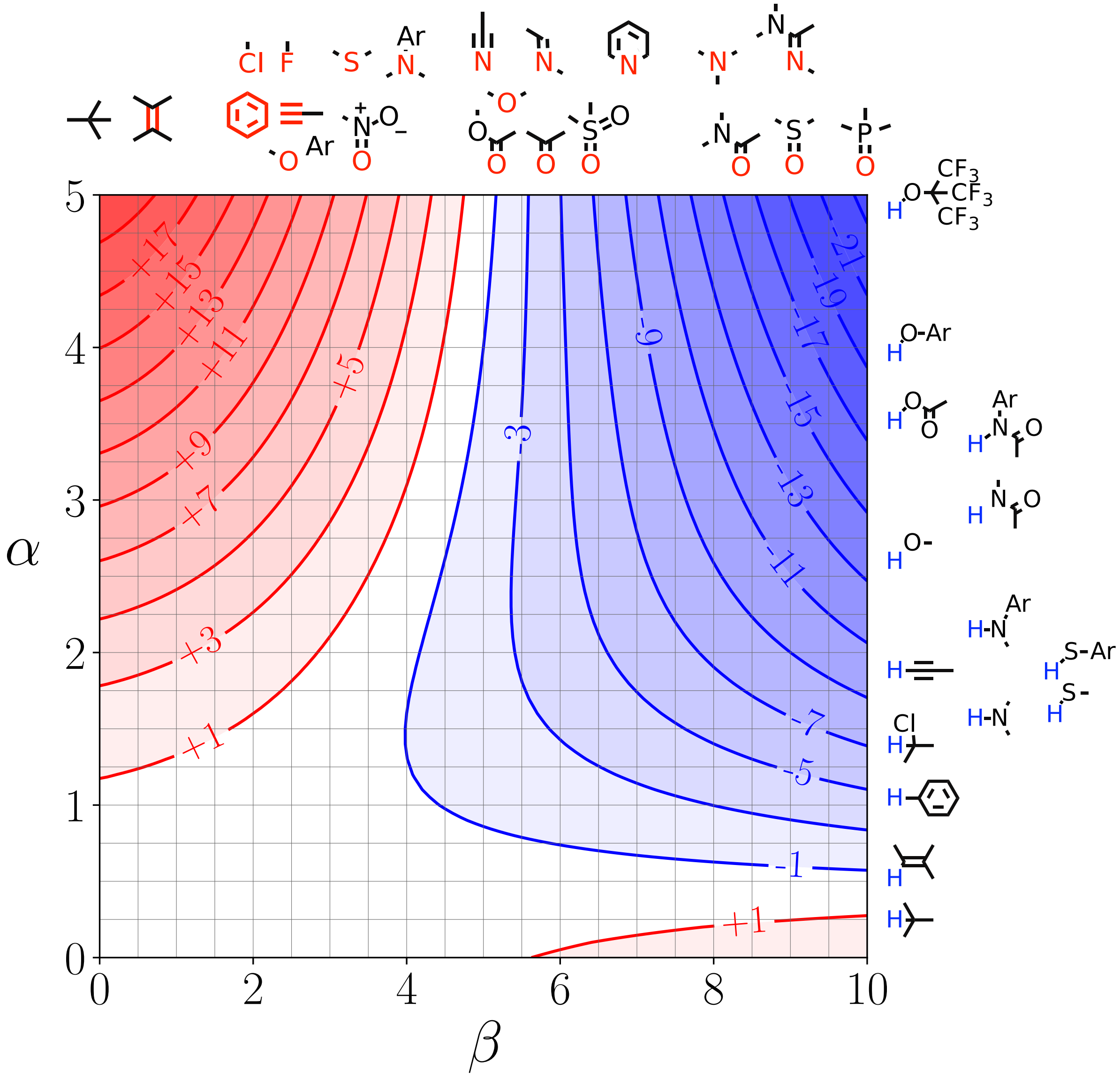

Figure S82: FGIP for anisole at 298K.

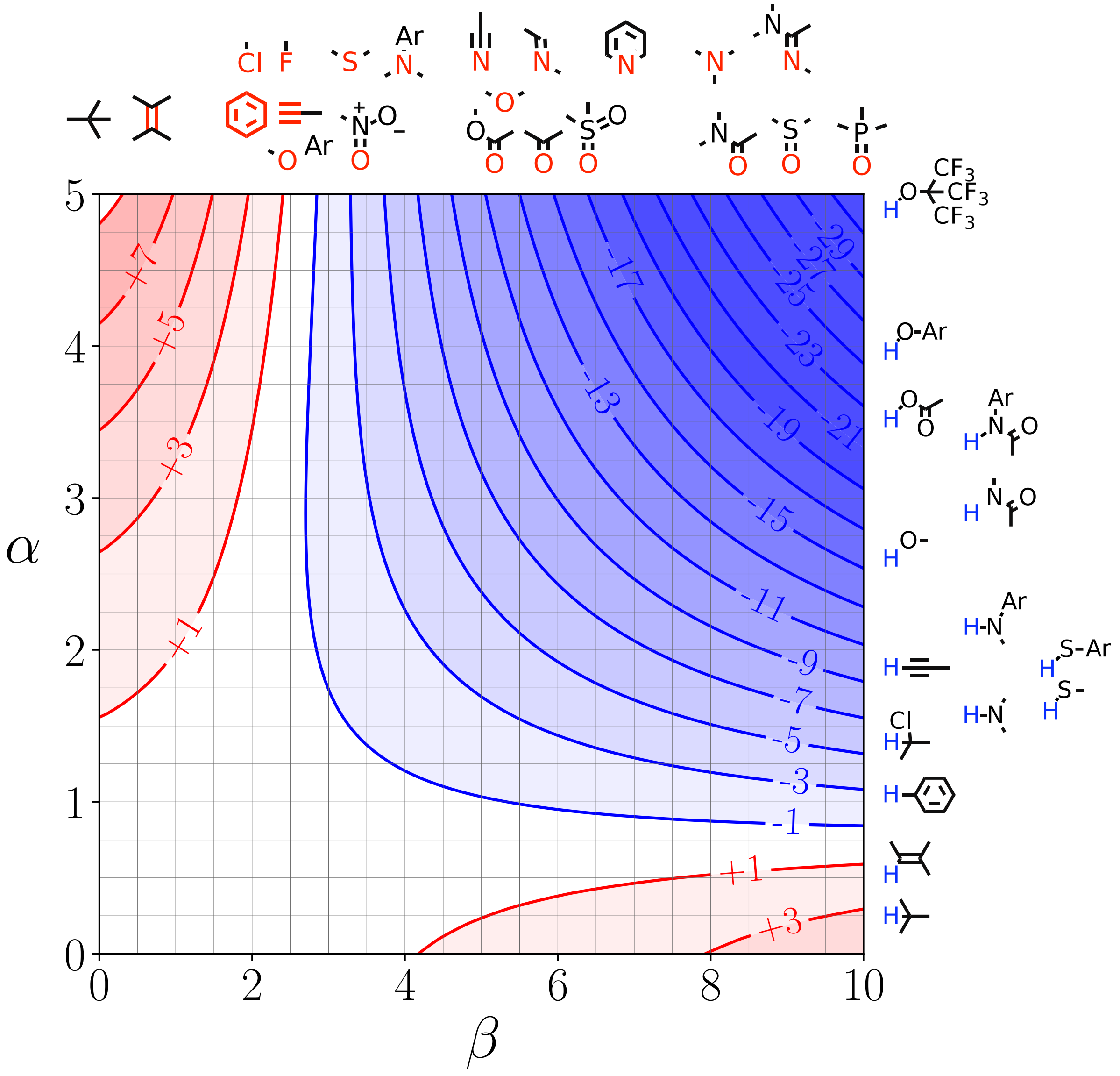

Figure S83: FGIP for ethyl phenyl ether at 298K.

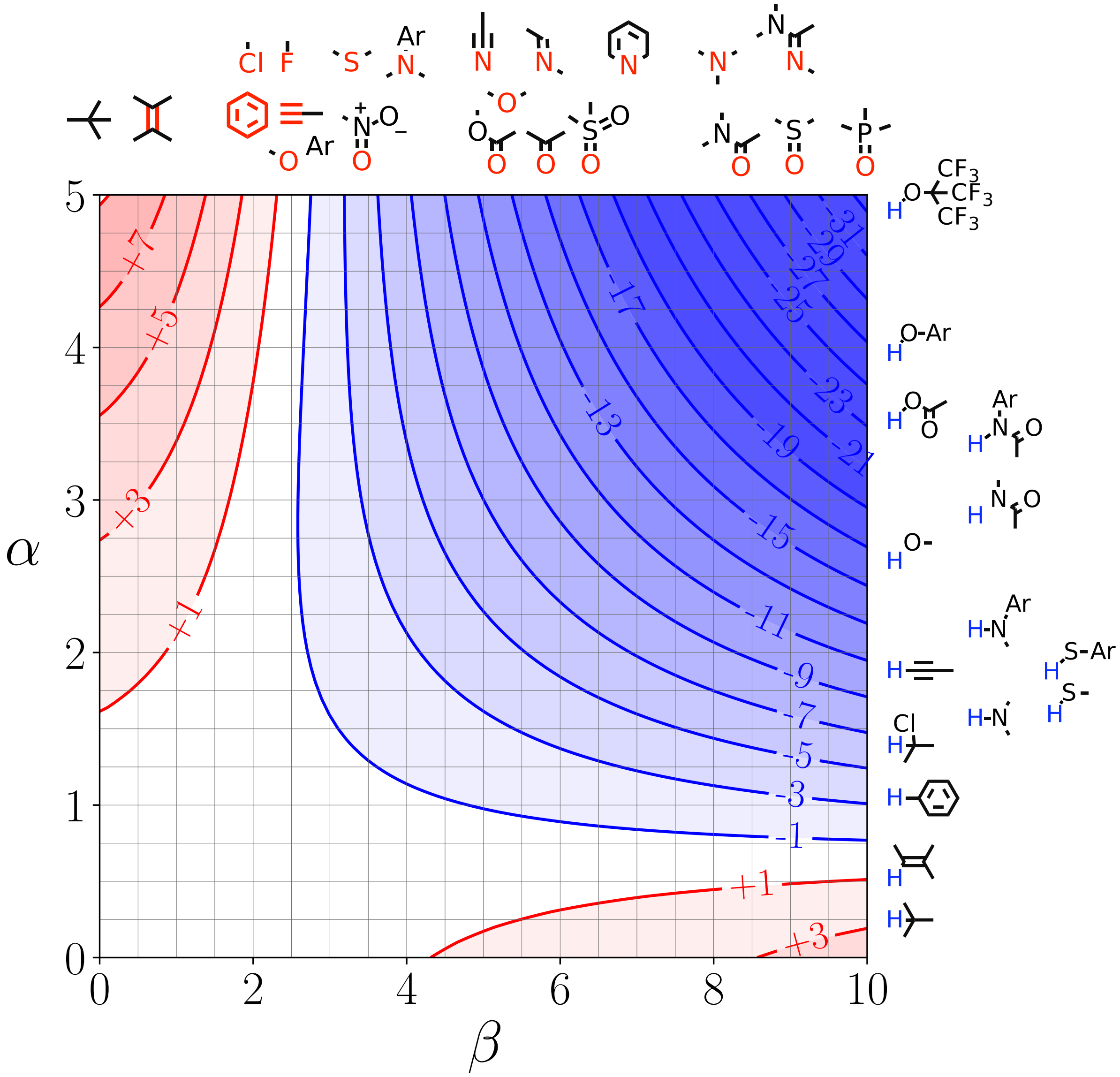

Figure S84: FGIP for diphenyl ether at 298K.

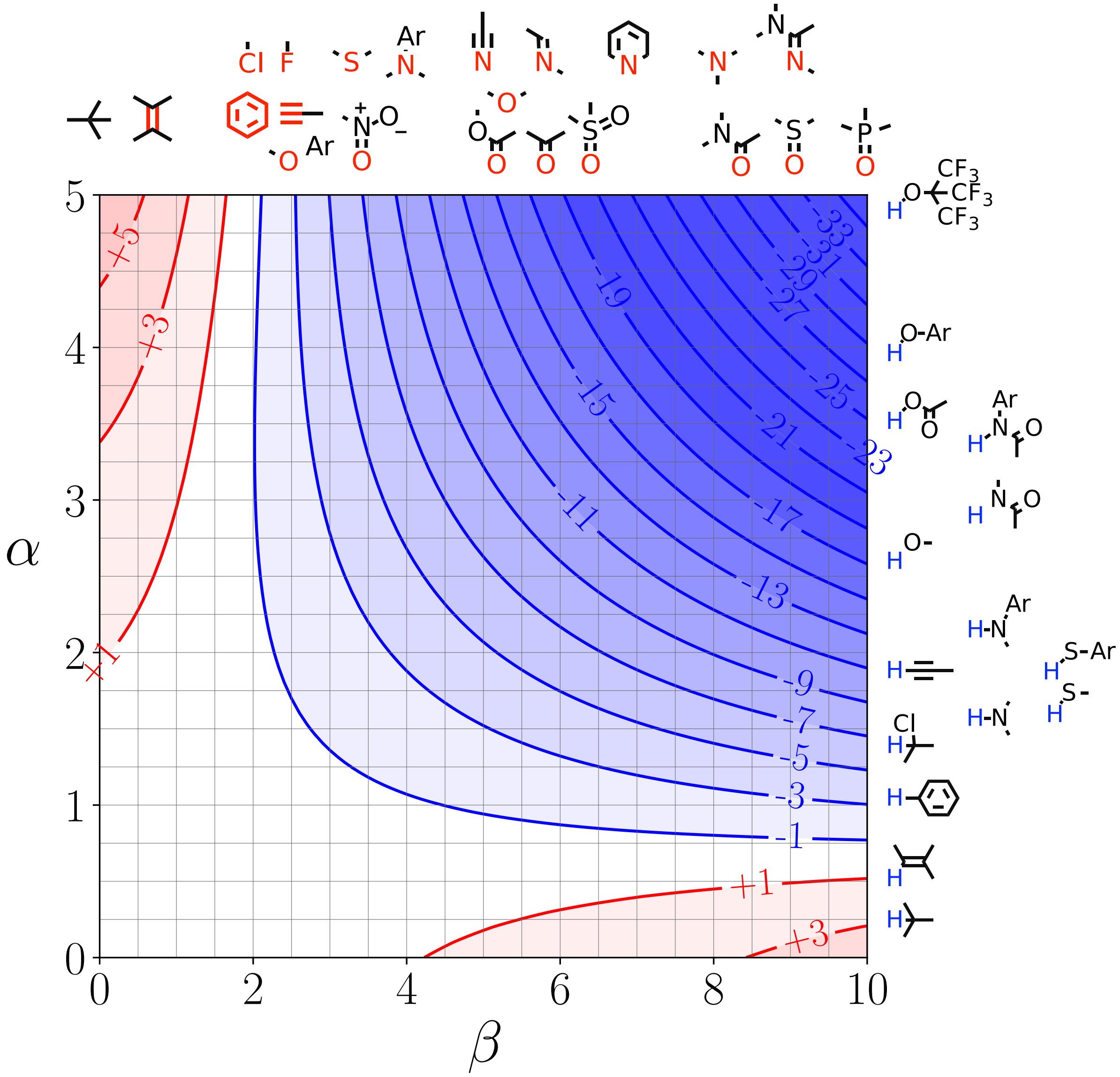

Figure S85: FGIP for dibenzyl ether at 298K.

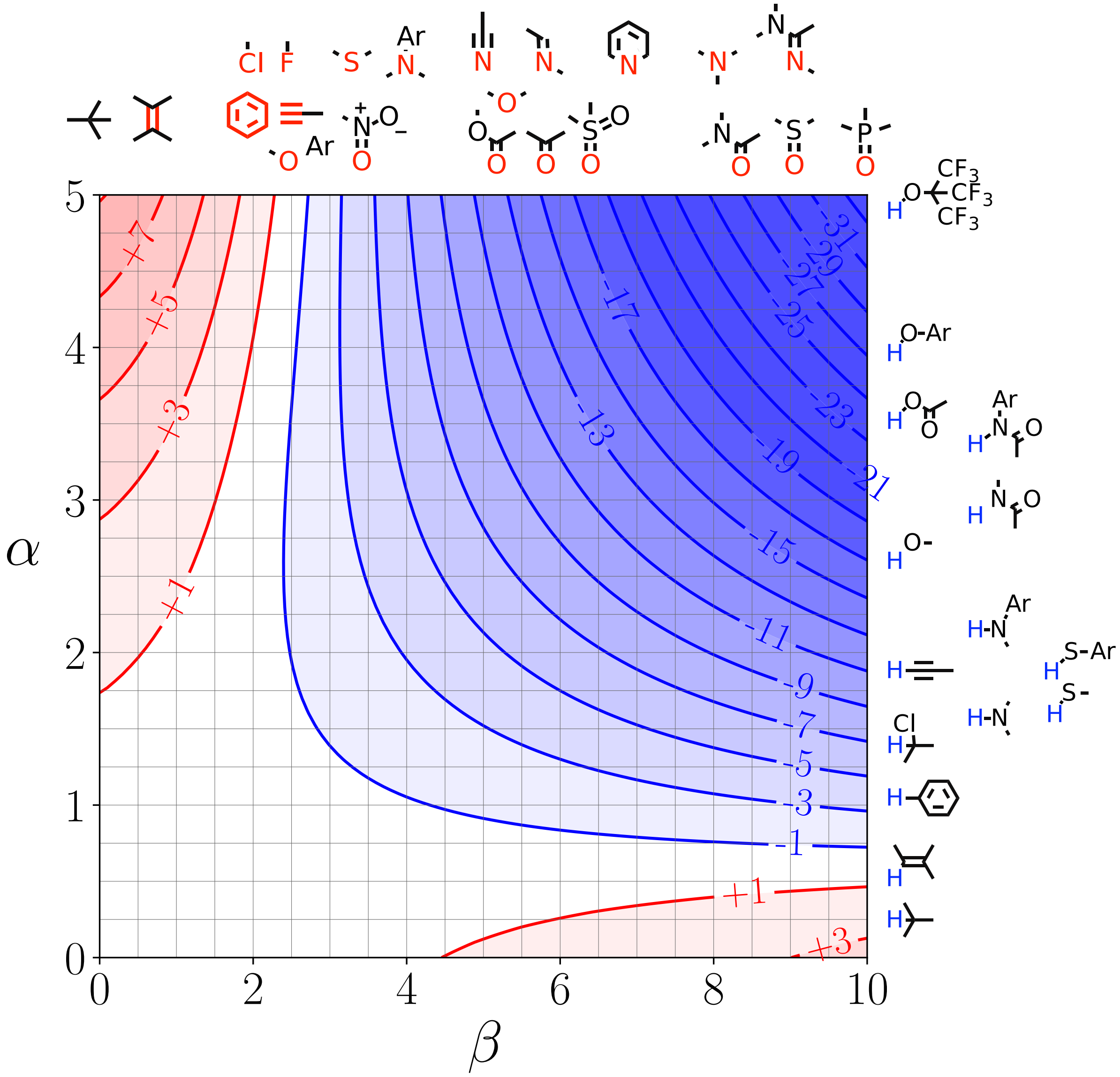

Figure S86: FGIP for 1,2-dimethoxybenzene at 298K.

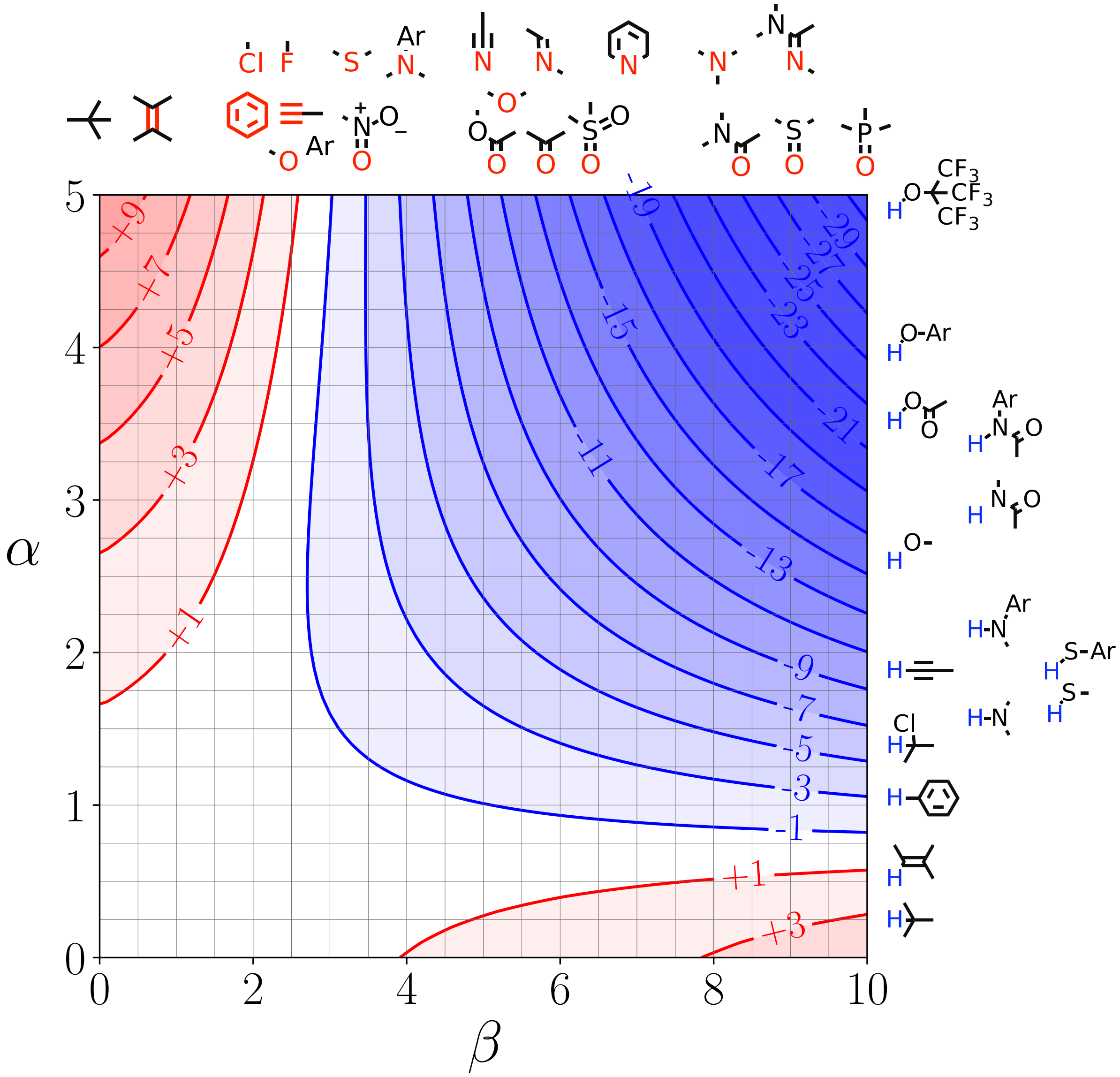

Figure S87: FGIP for methyl orthoformate at 298K.

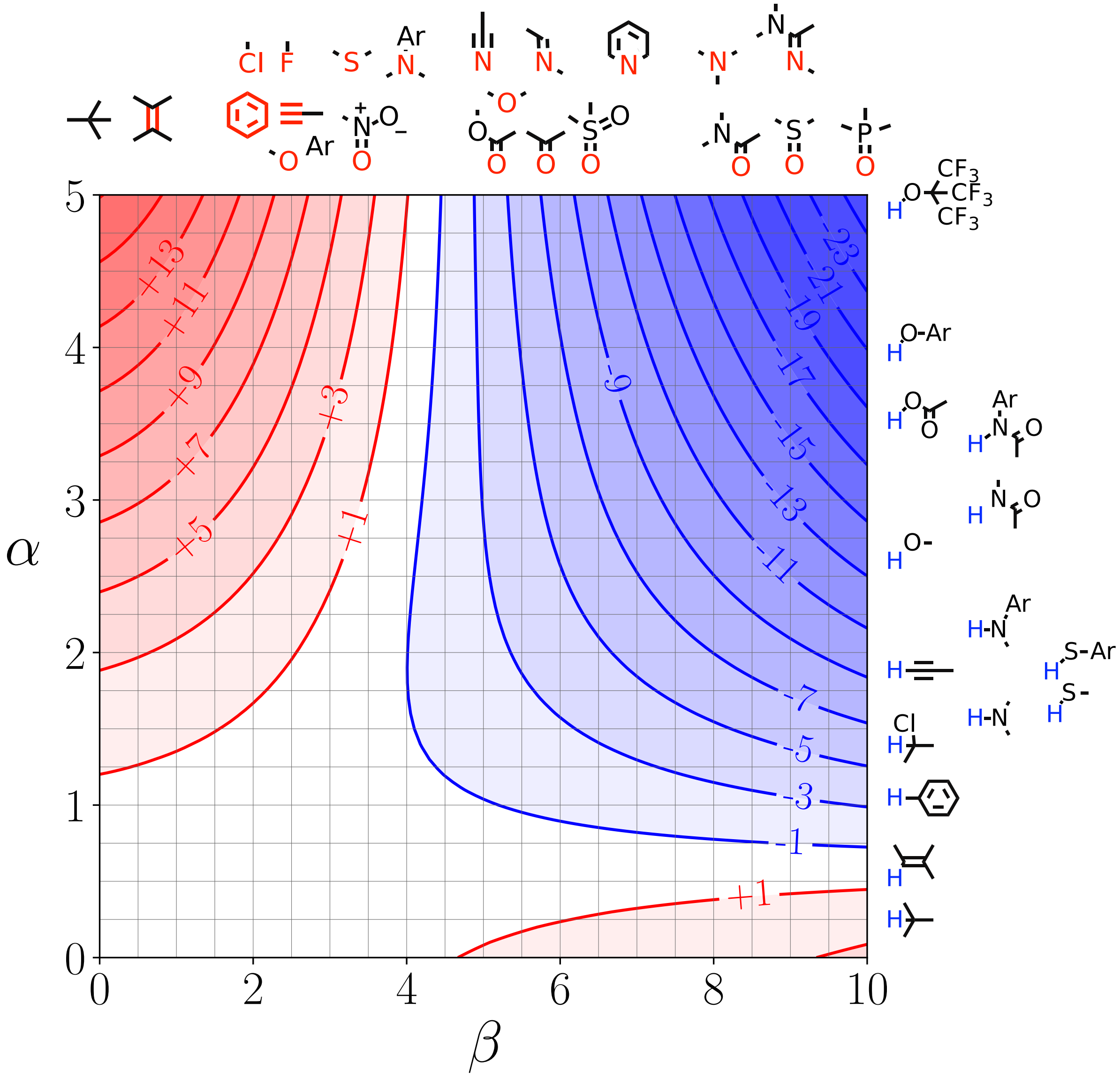

Figure S88: FGIP for methyl orthoacetate at 298K.

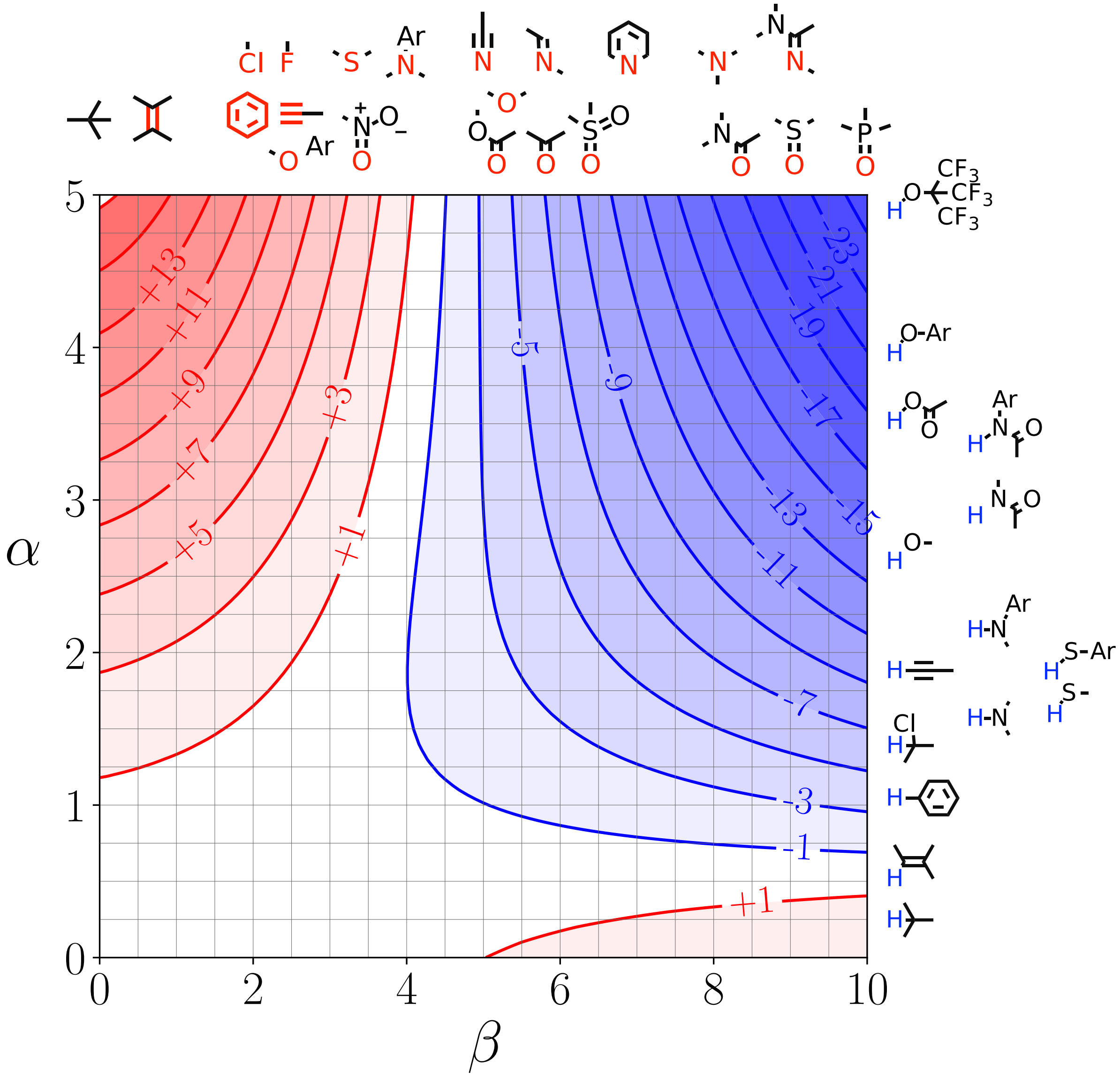

Figure S89: FGIP for propionaldehyde at 298K.

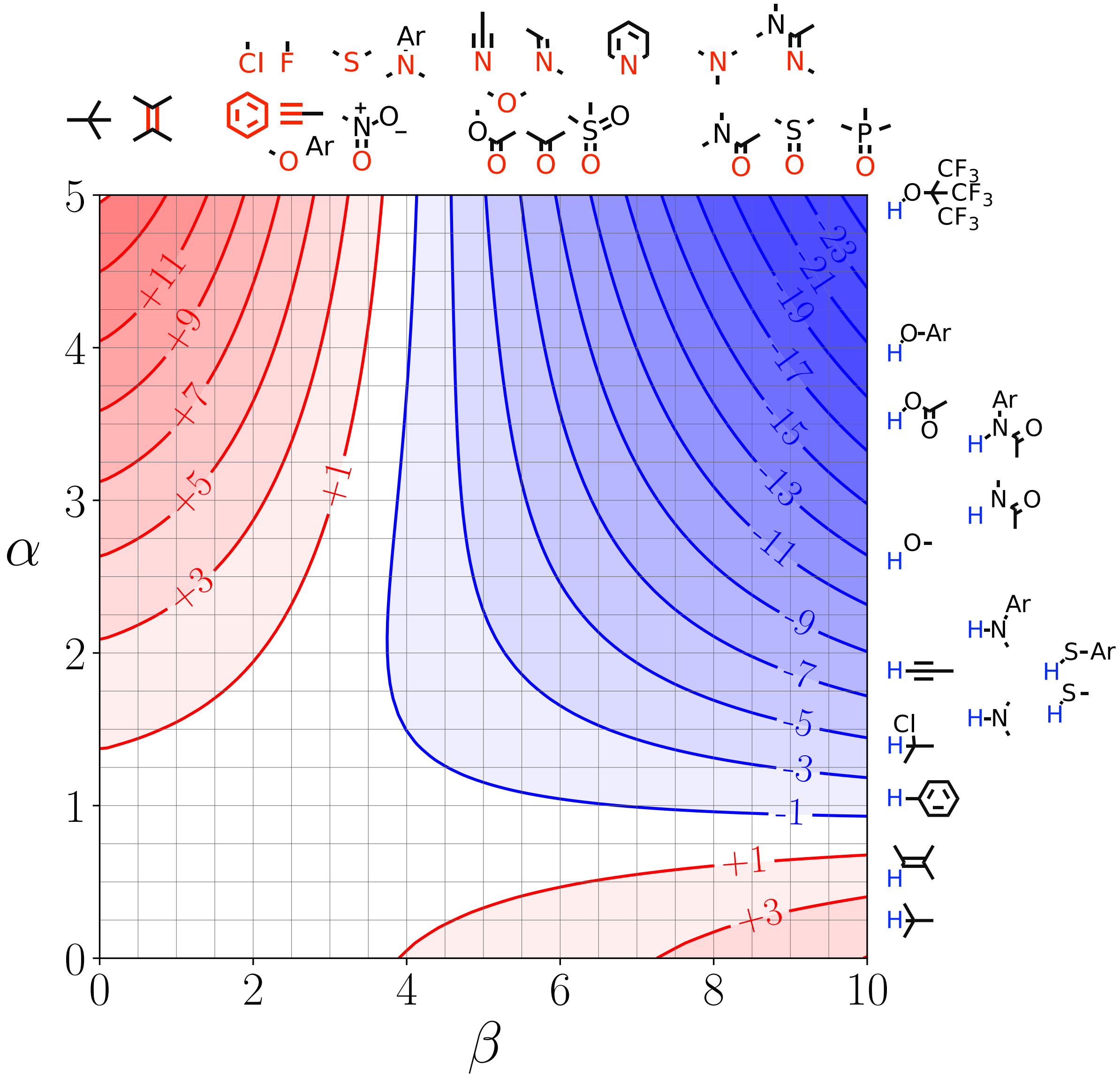

Figure S90: FGIP for butyraldehyde at 298K.

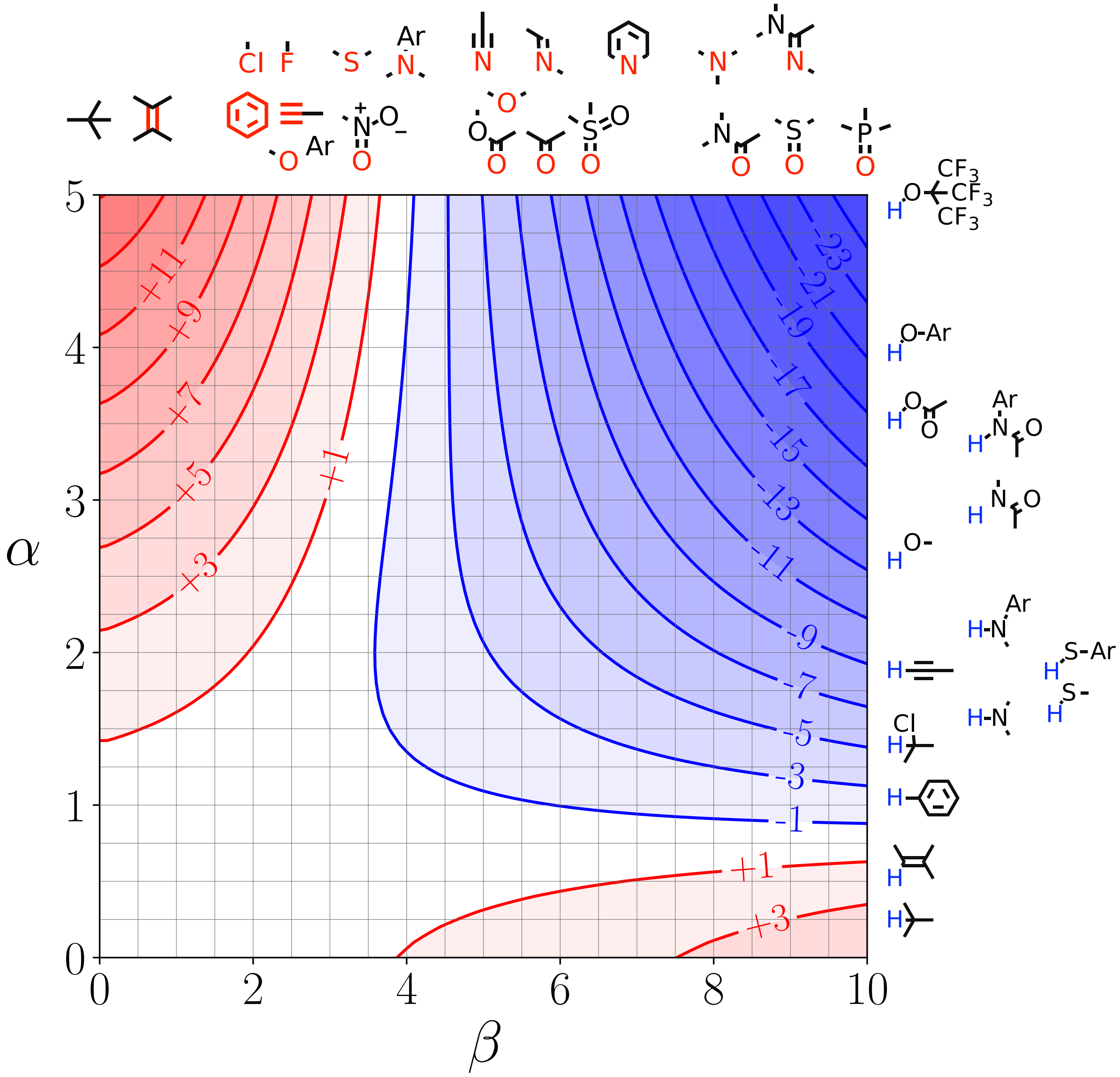

Figure S91: FGIP for benzaldehyde at 298K.

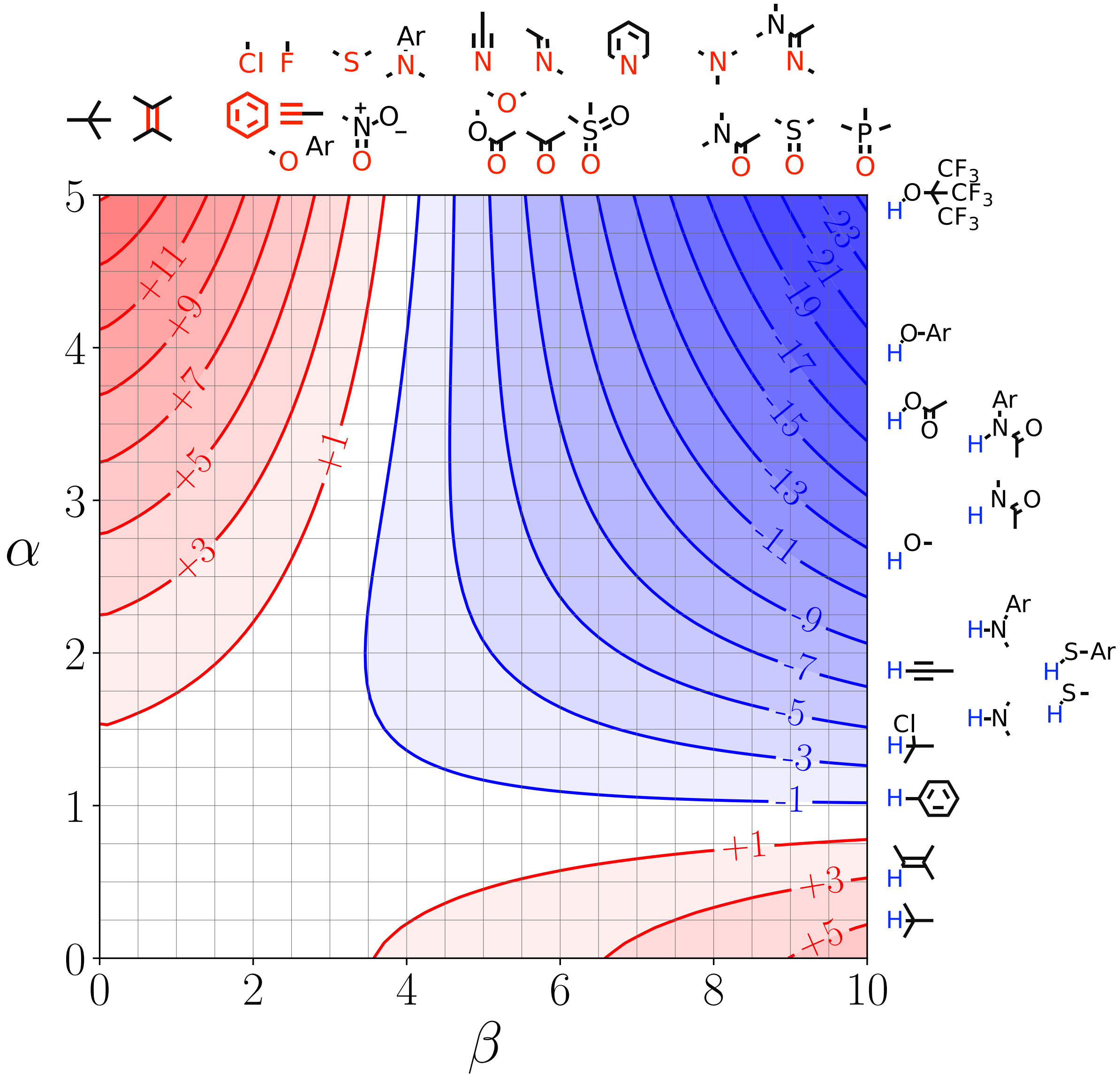

Figure S92: FGIP for p-methoxybenzaldehyde at 298K.

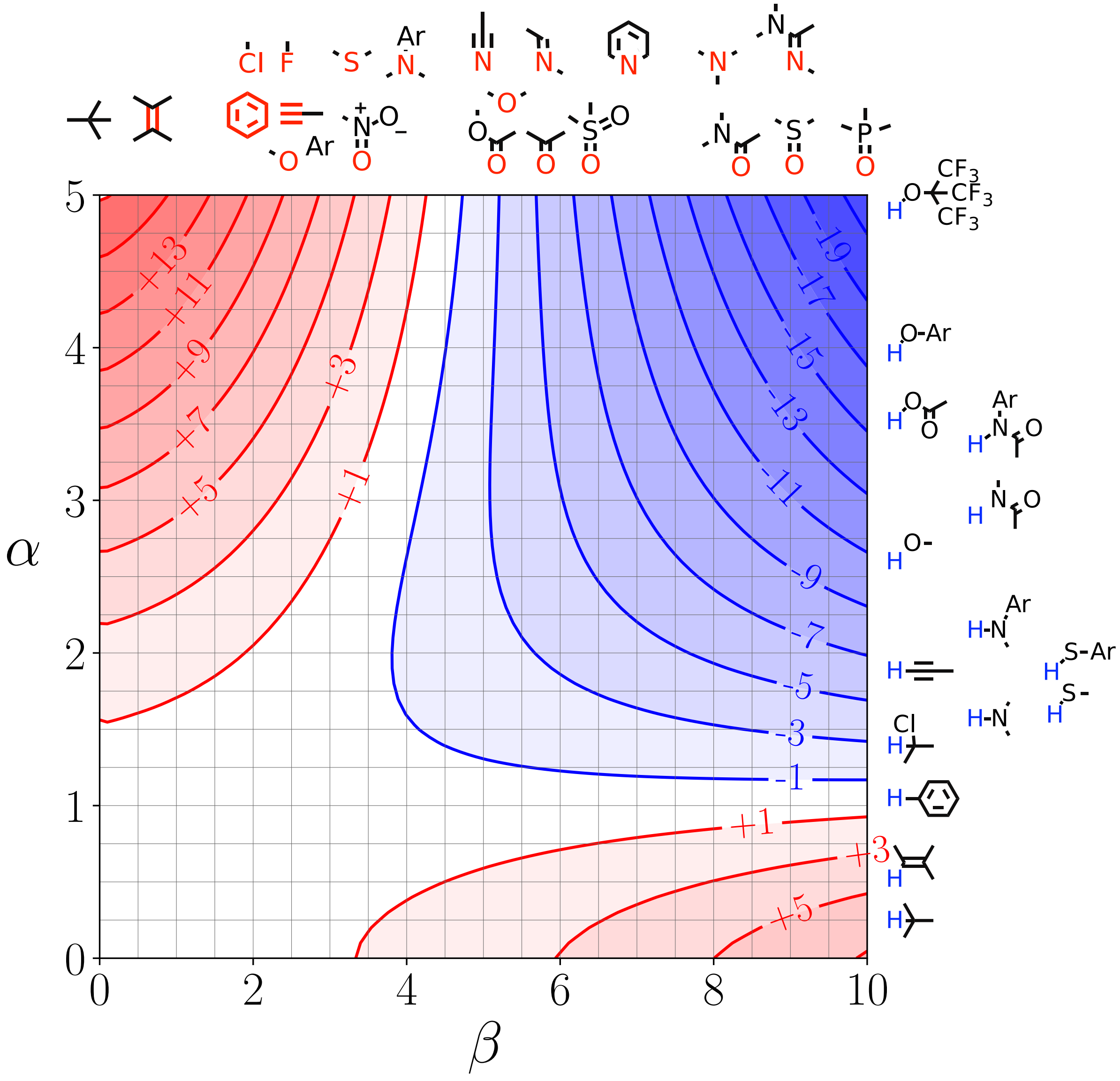

Figure S93: FGIP for cinnamaldehyde at 298K.

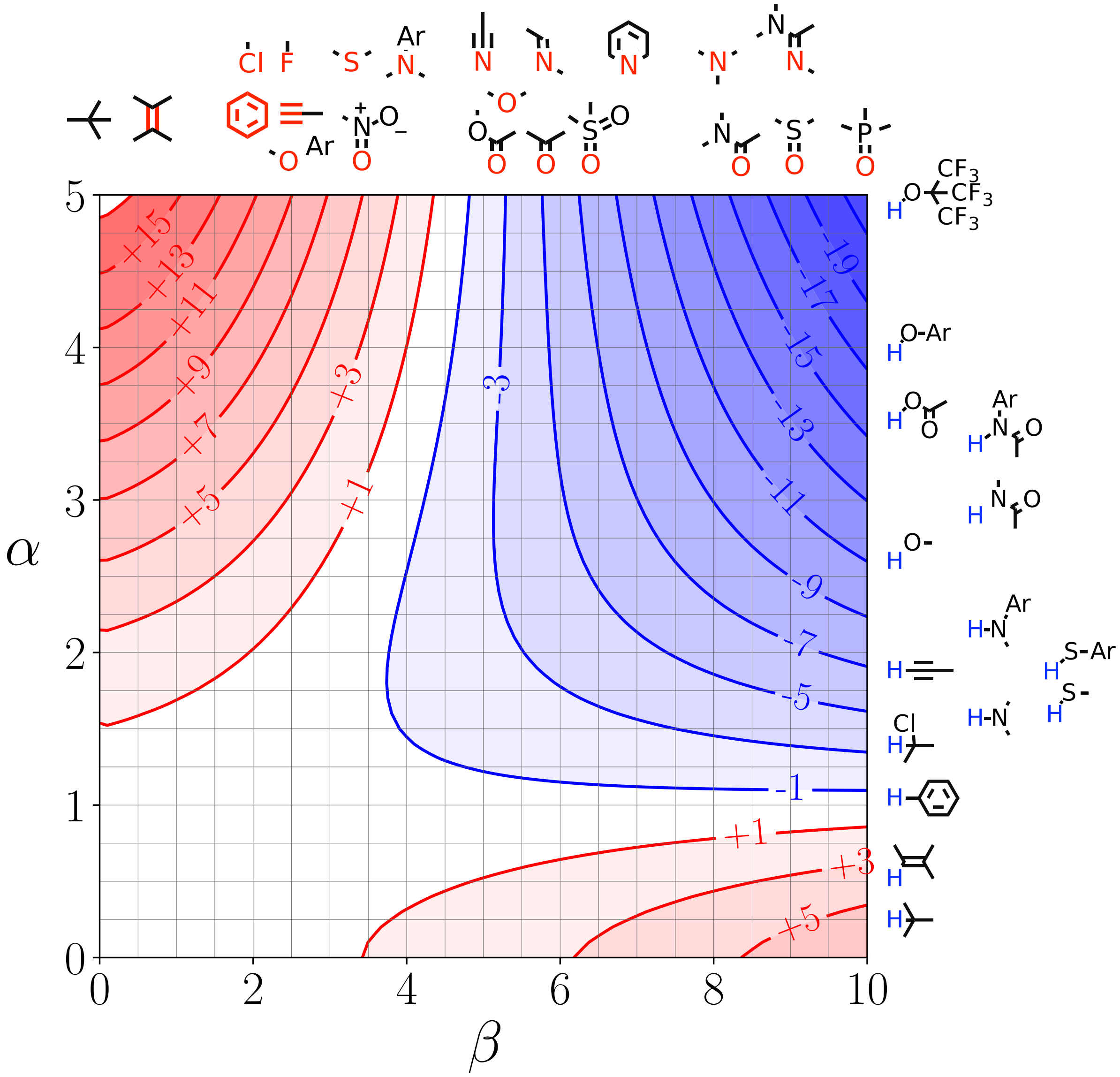

Figure S94: FGIP for acetone at 298K.

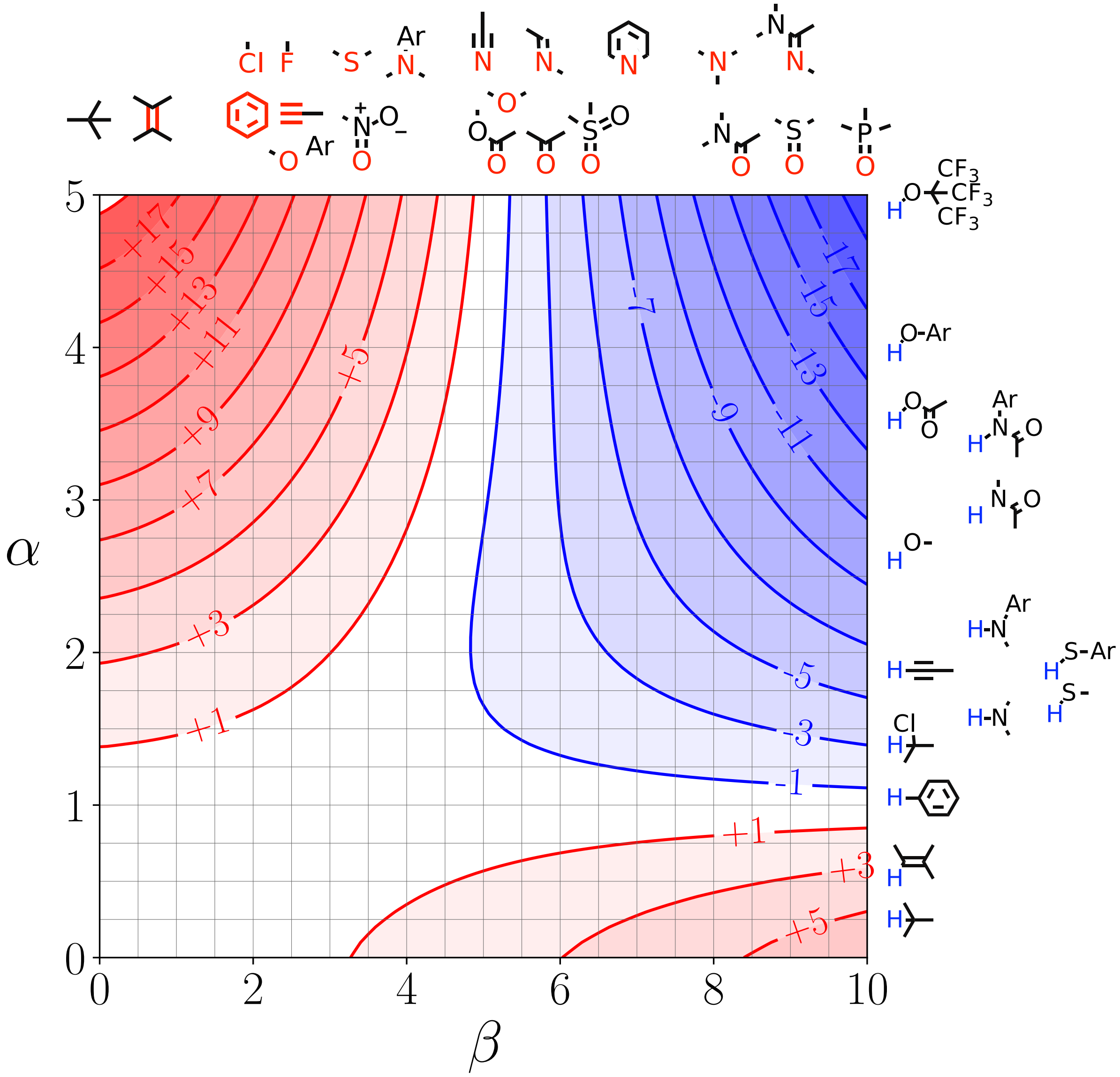

Figure S95: FGIP for 2-butanone at 298K.

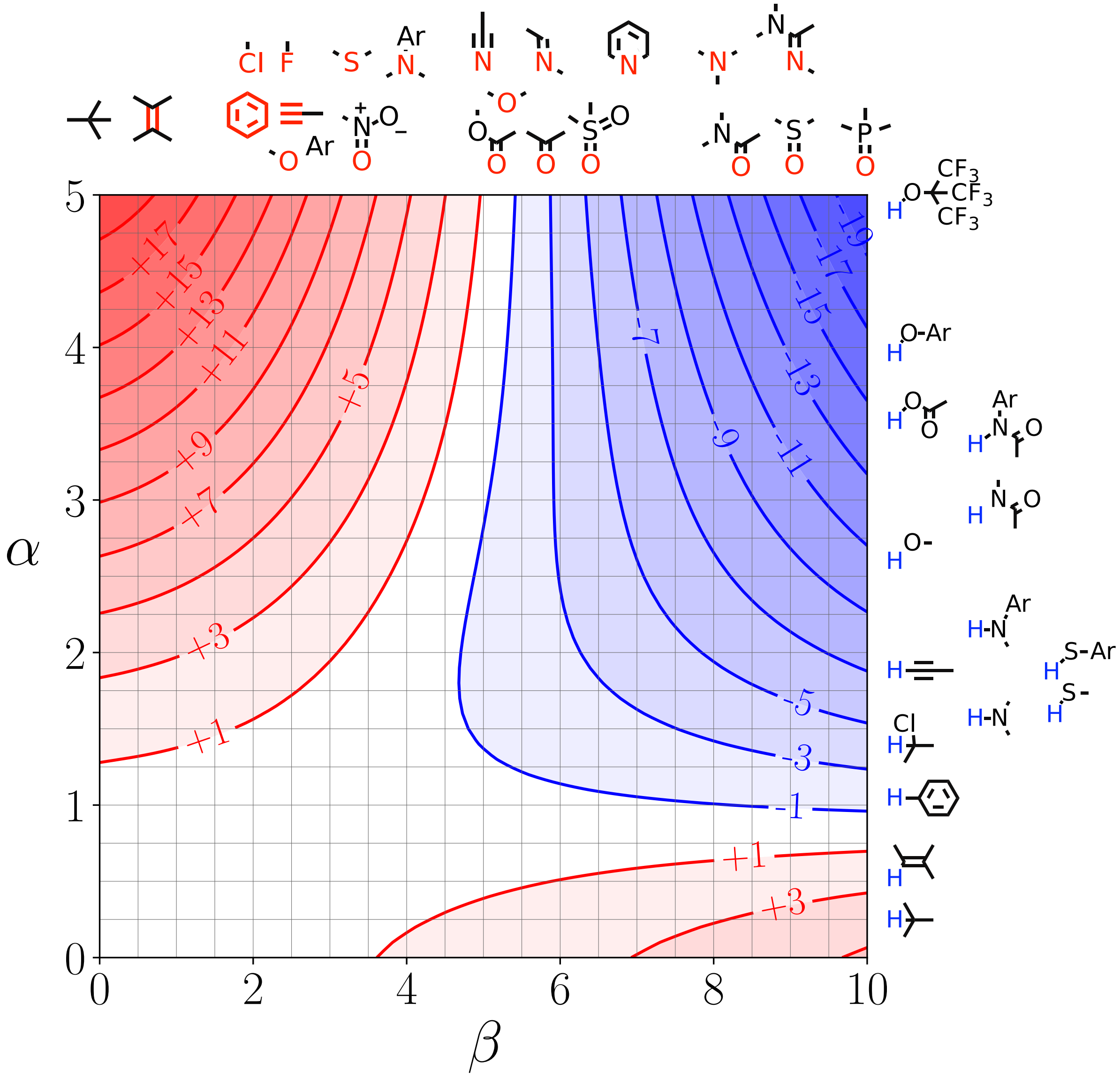

Figure S96: FGIP for 2-pentanone at 298K.

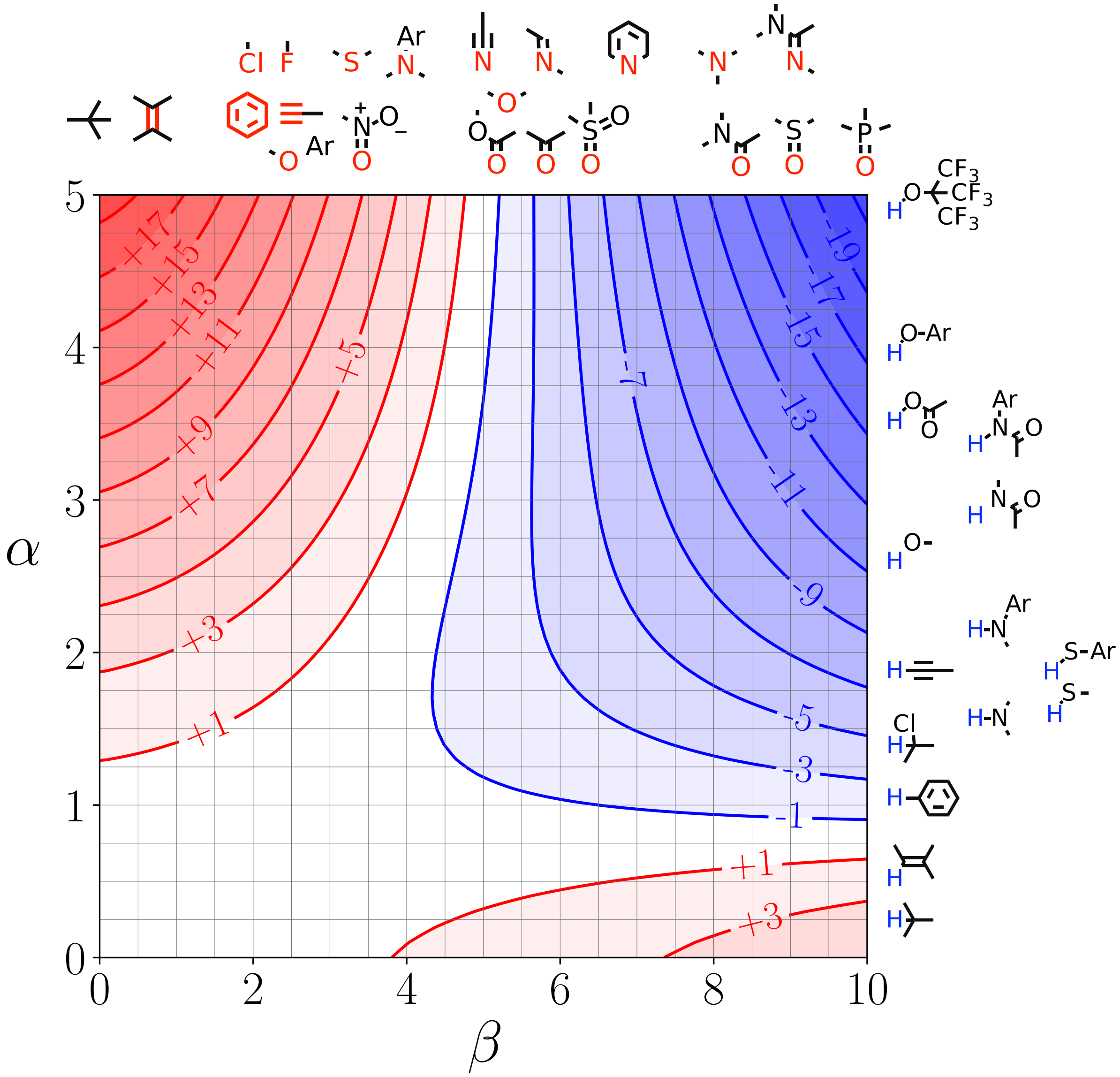

Figure S97: FGIP for 3-methyl-2-butanone at 298K.

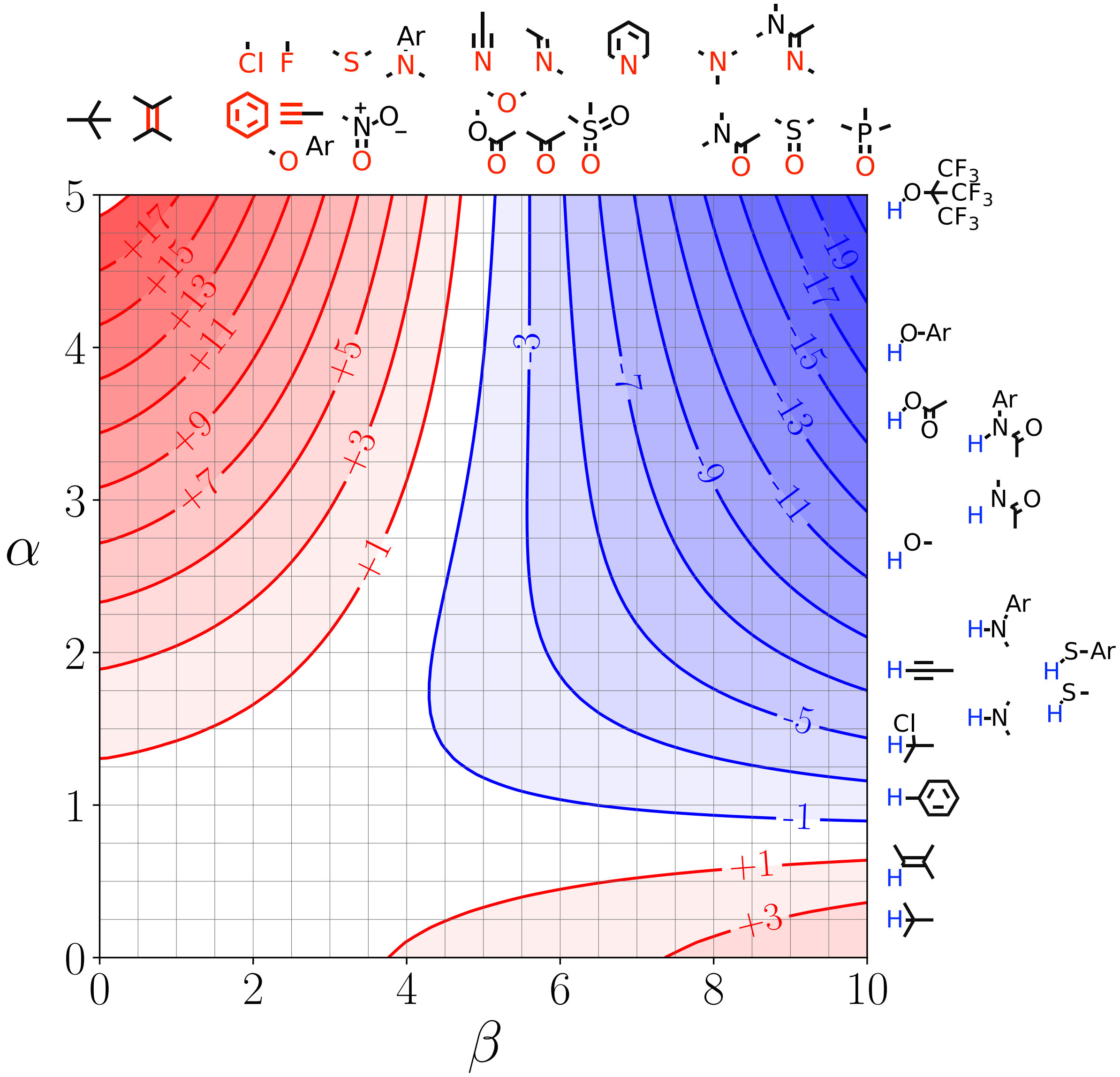

Figure S98: FGIP for 3-pentanone at 298K.

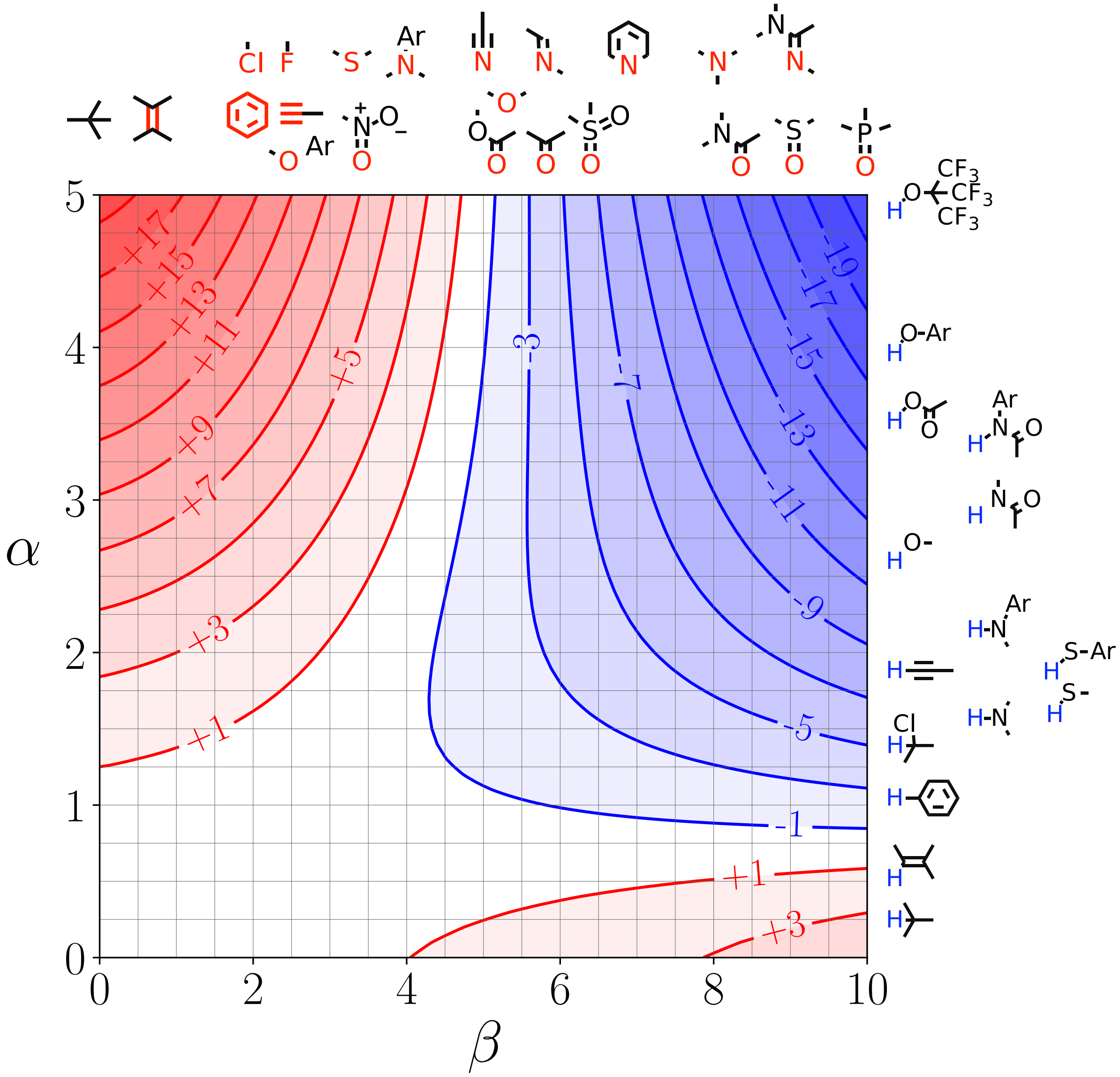

Figure S99: FGIP for cyclopentanone at 298K.

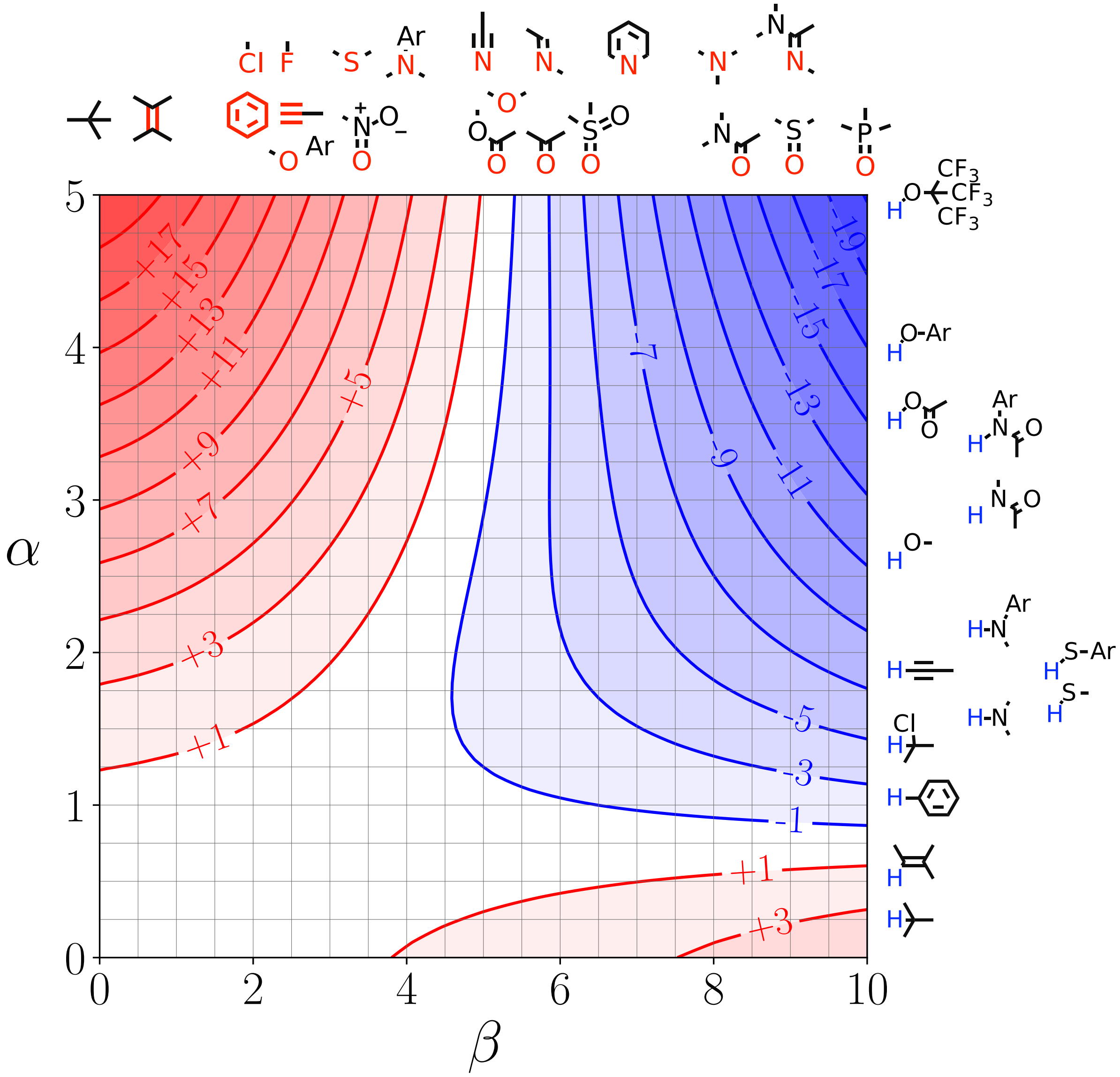

Figure S100: FGIP for 4-methyl-2-pentanone at 298K.

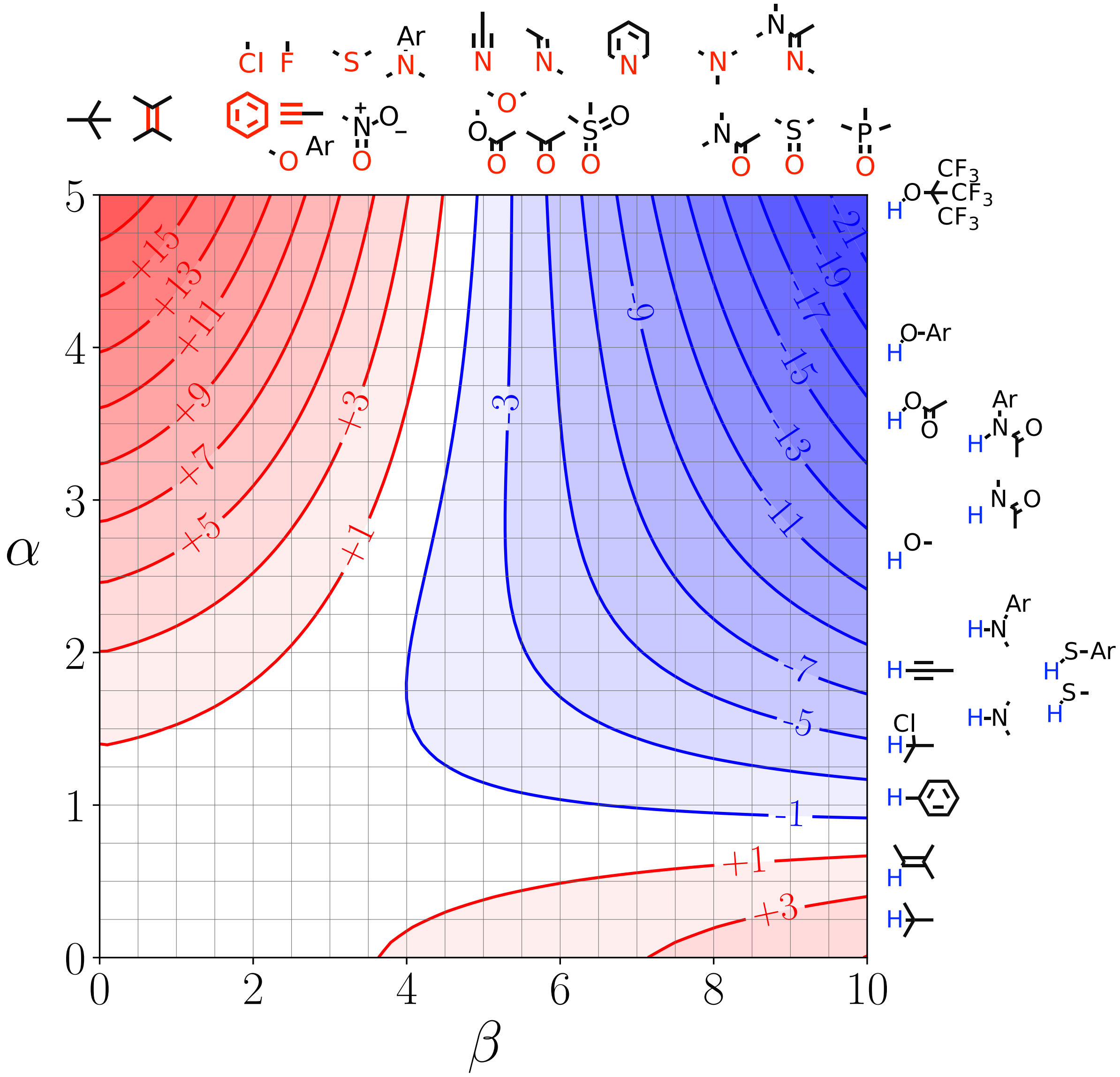

Figure S101: FGIP for 3,3-dimethyl-2-butanone at 298K.

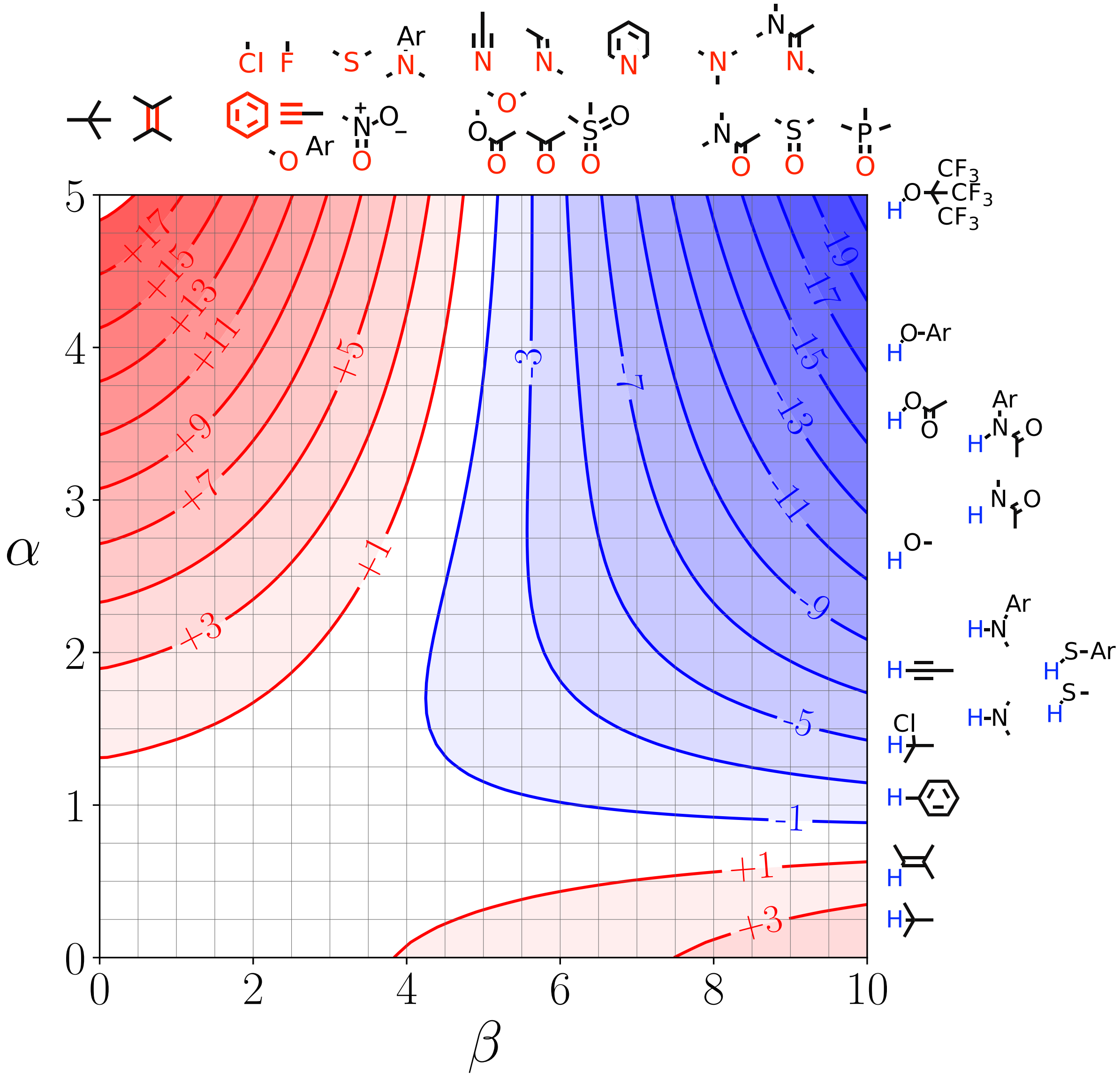

Figure S102: FGIP for perfluorooctane at 298K.

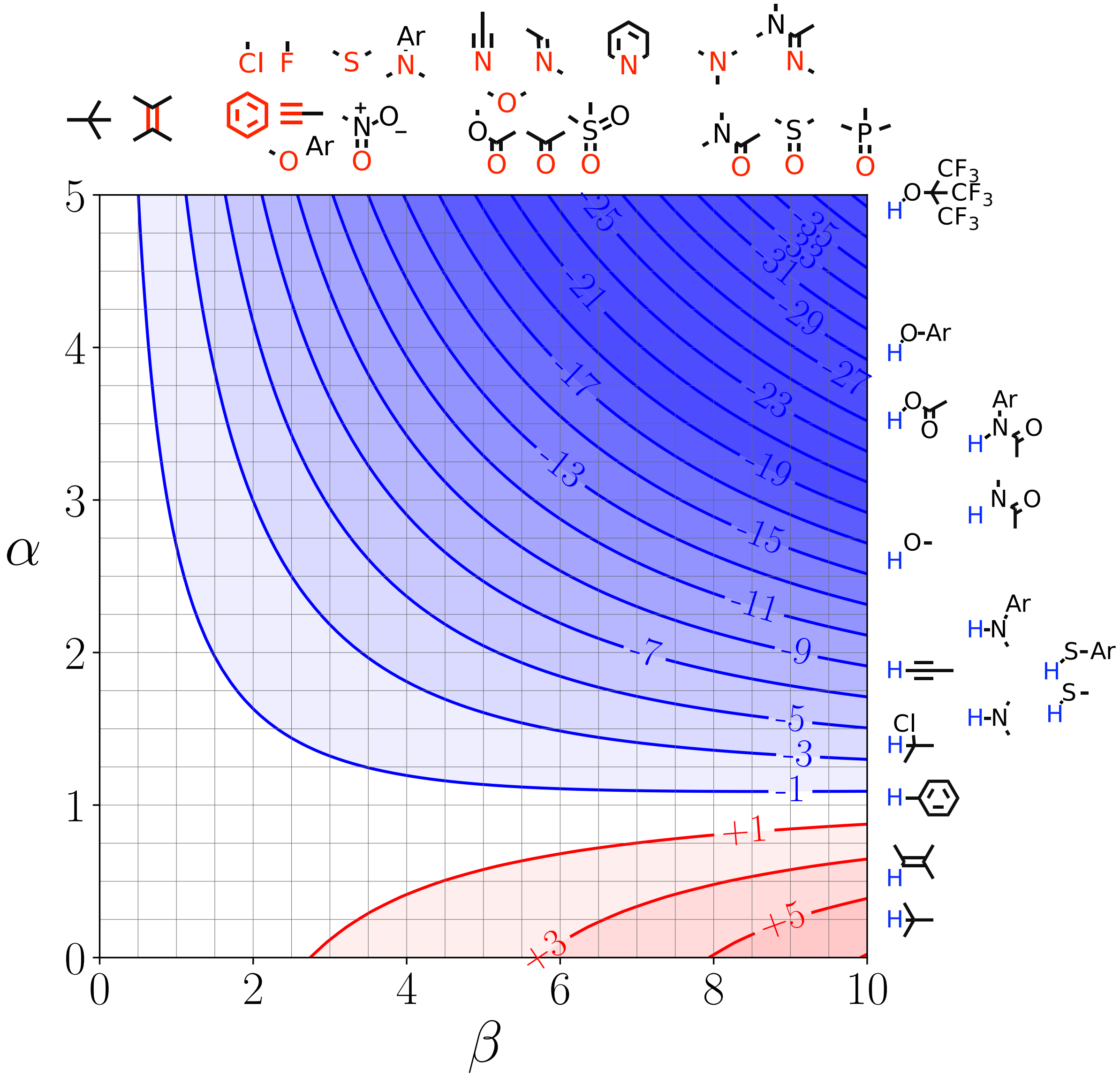

Figure S103: FGIP for cyclohexanone at 298K.

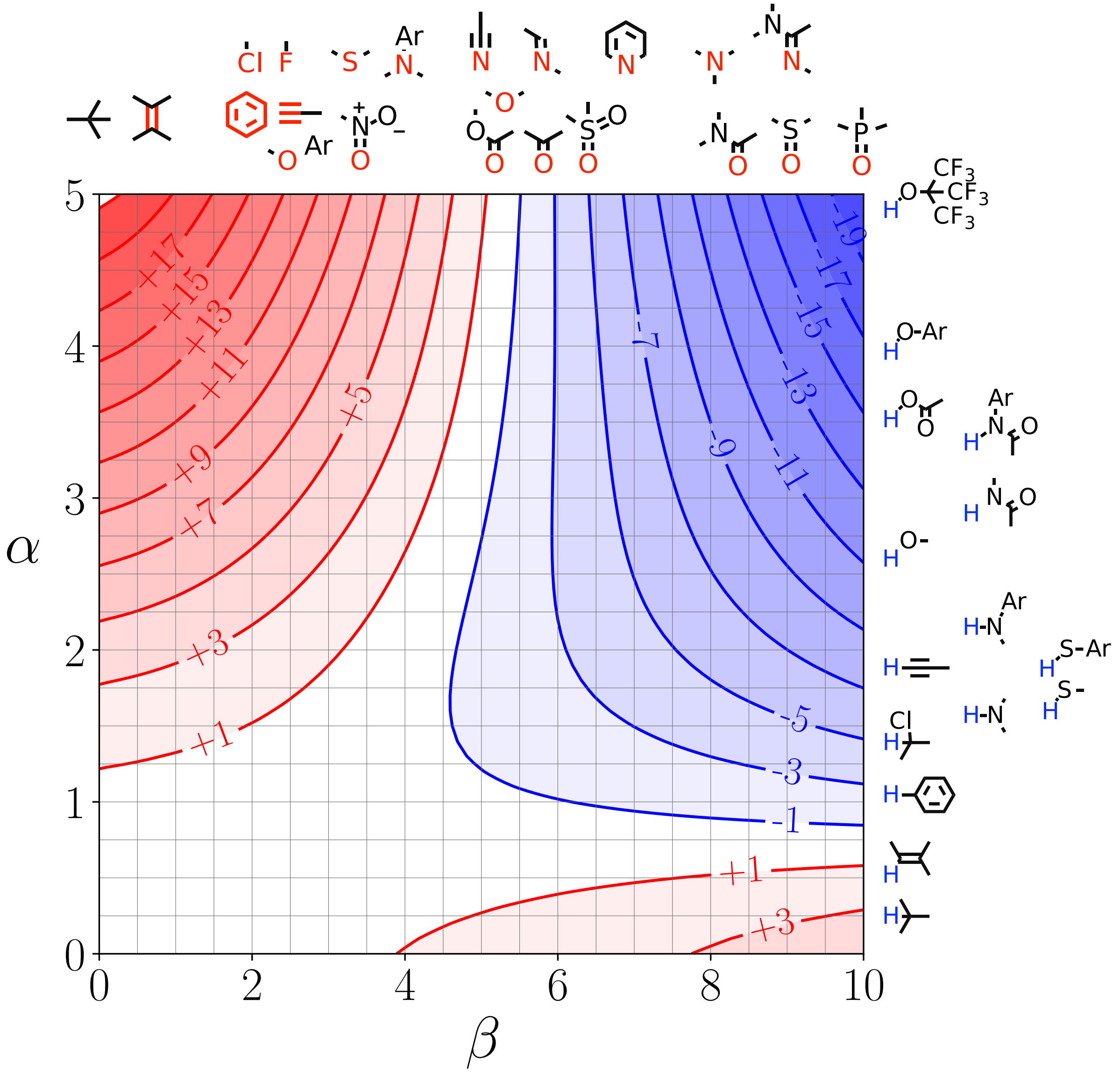

Figure S104: FGIP for 2-heptanone at 298K.

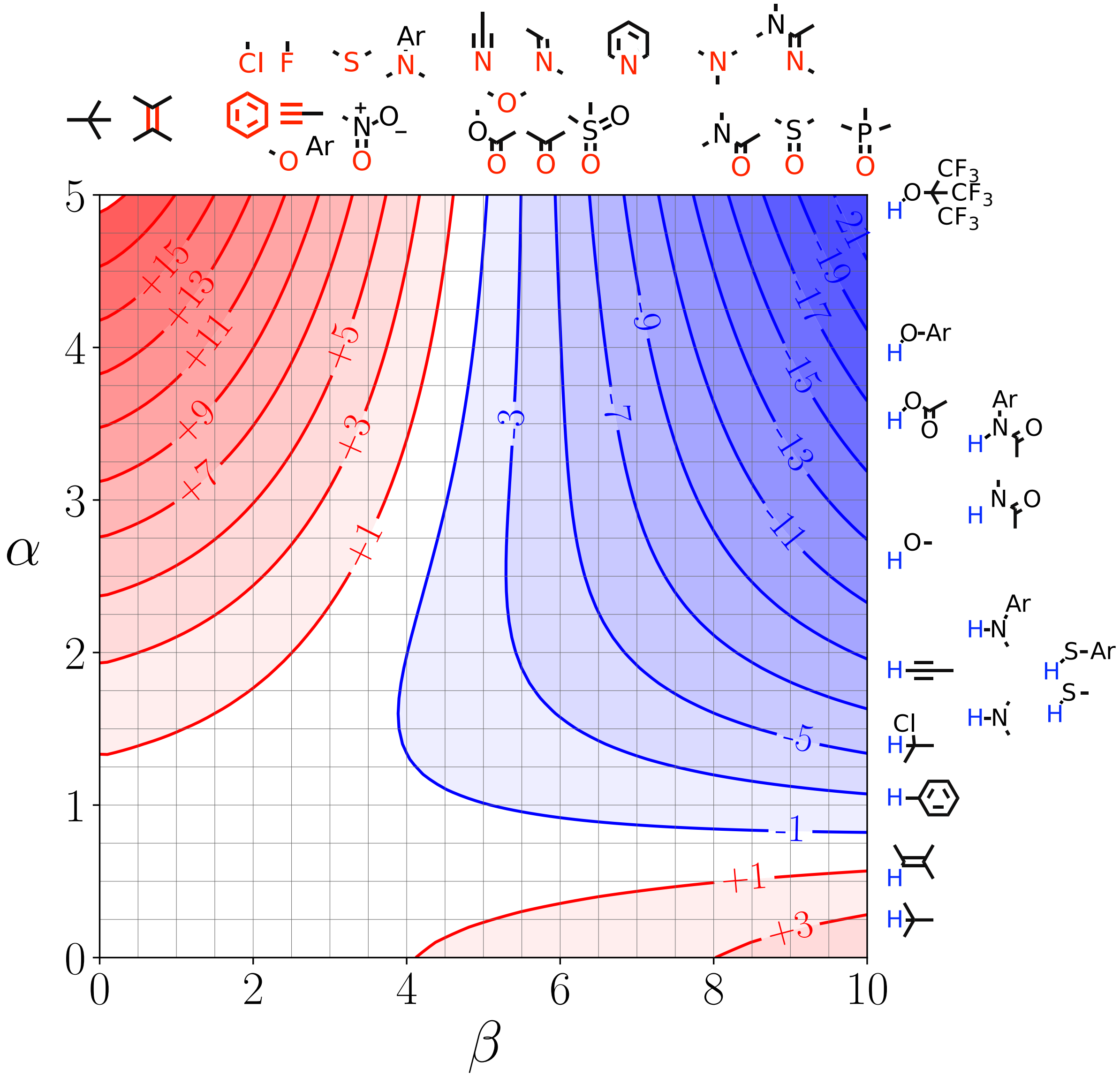

Figure S105: FGIP for 3-heptanone at 298K.

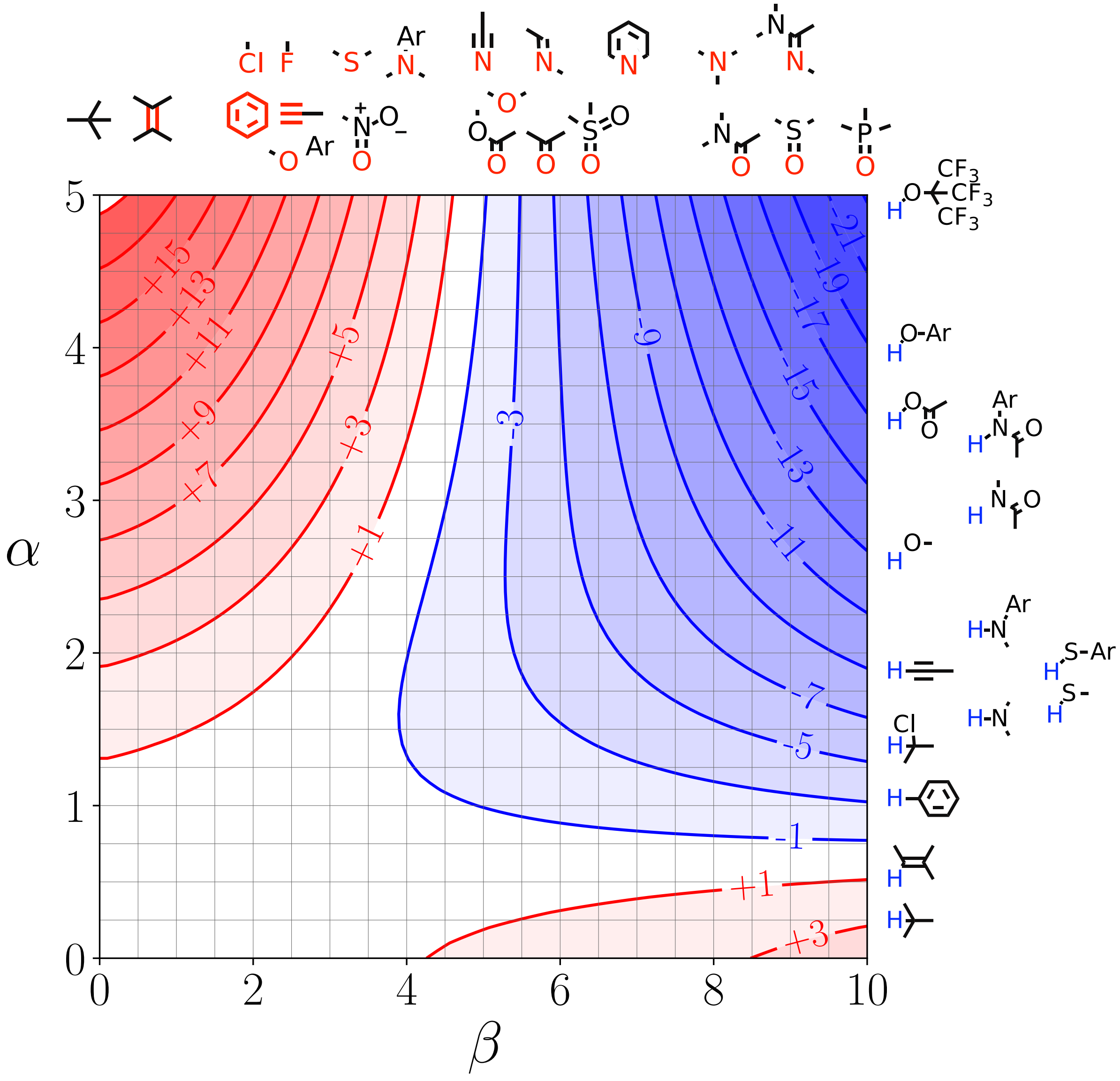

Figure S106: FGIP for 2,2,4,4-tetramethyl-3-pentanone at 298K.

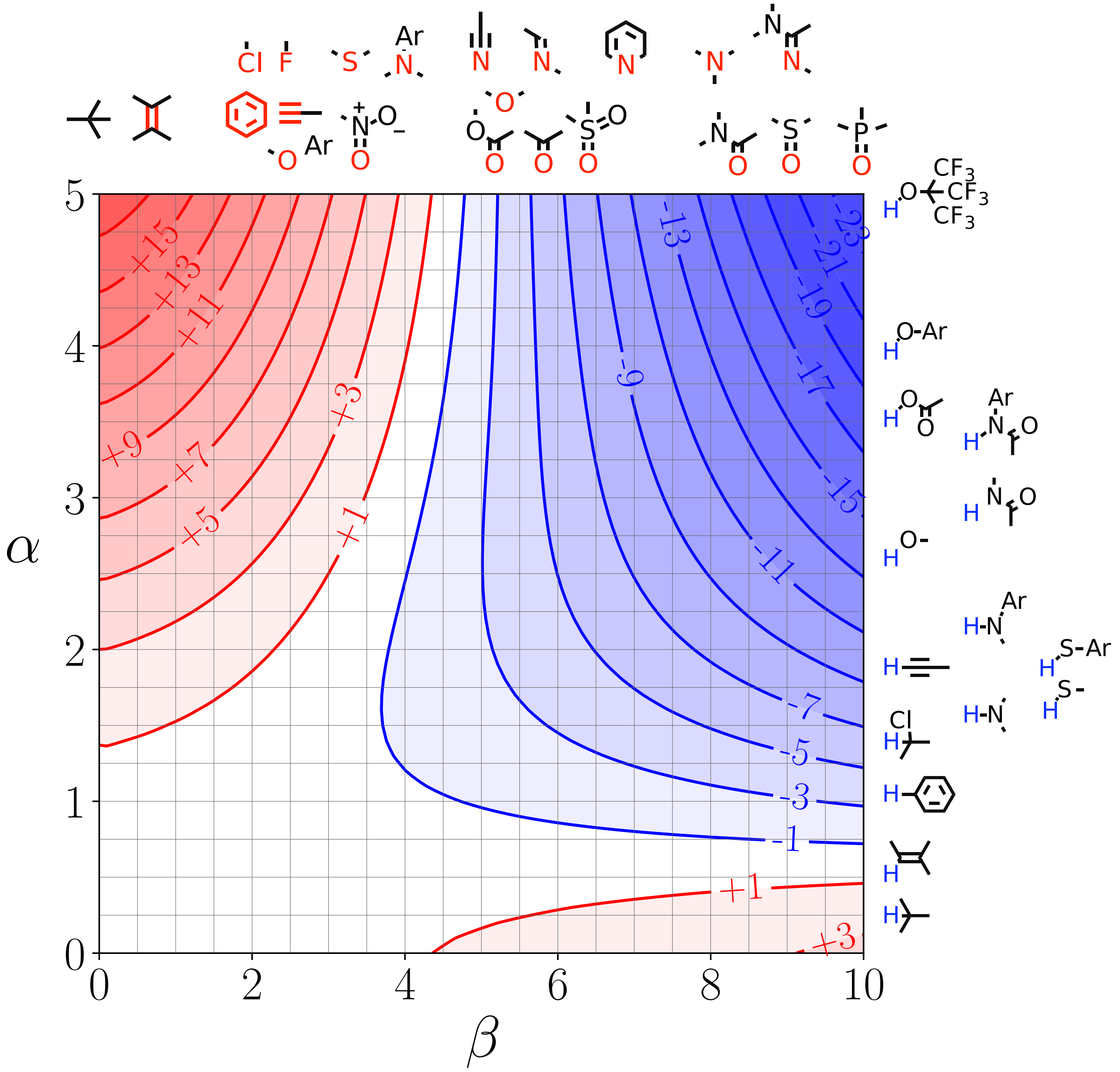

Figure S107: FGIP for acetophenone at 298K.

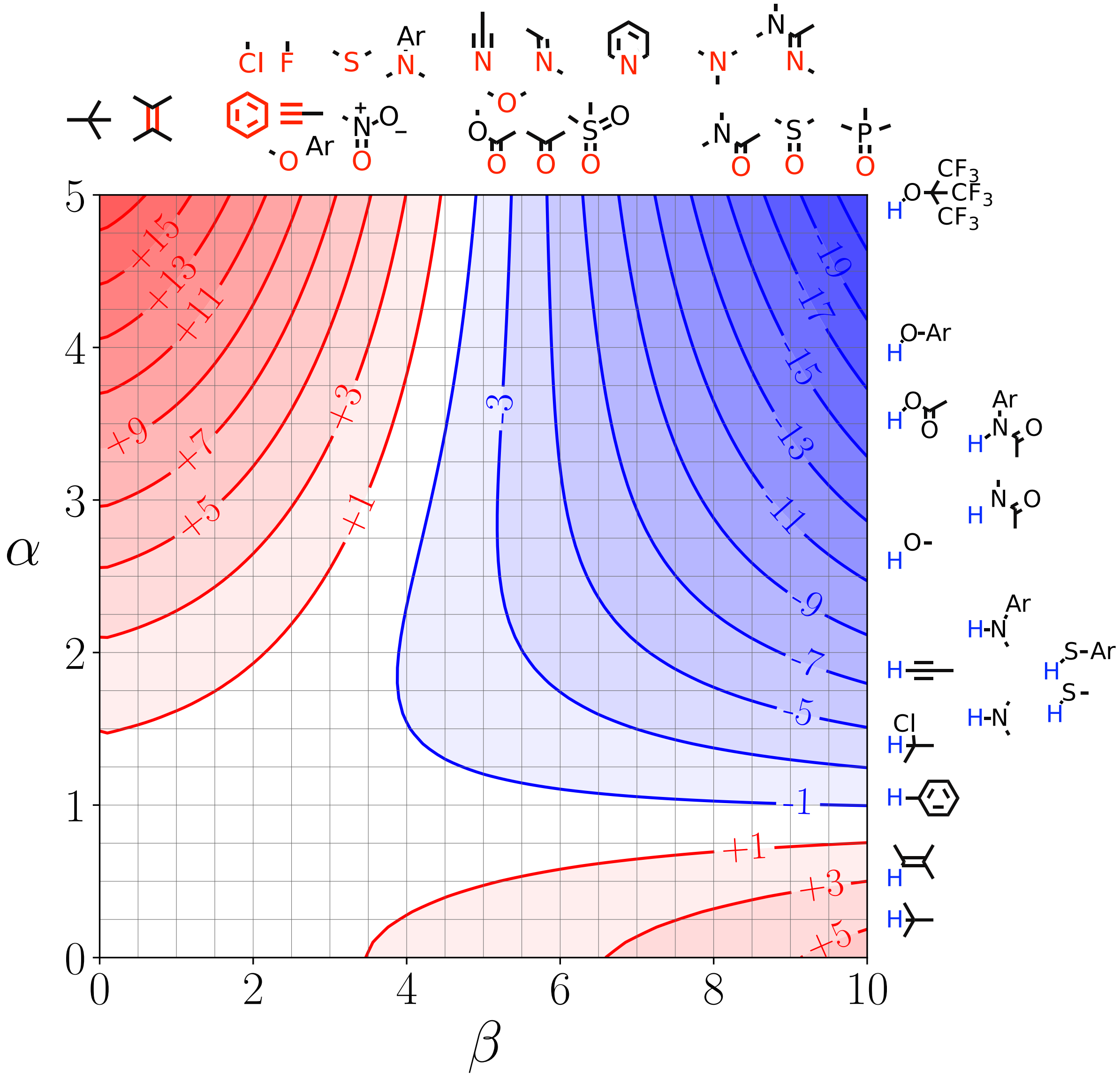

Figure S108: FGIP for ethyl phenyl ketone at 298K.

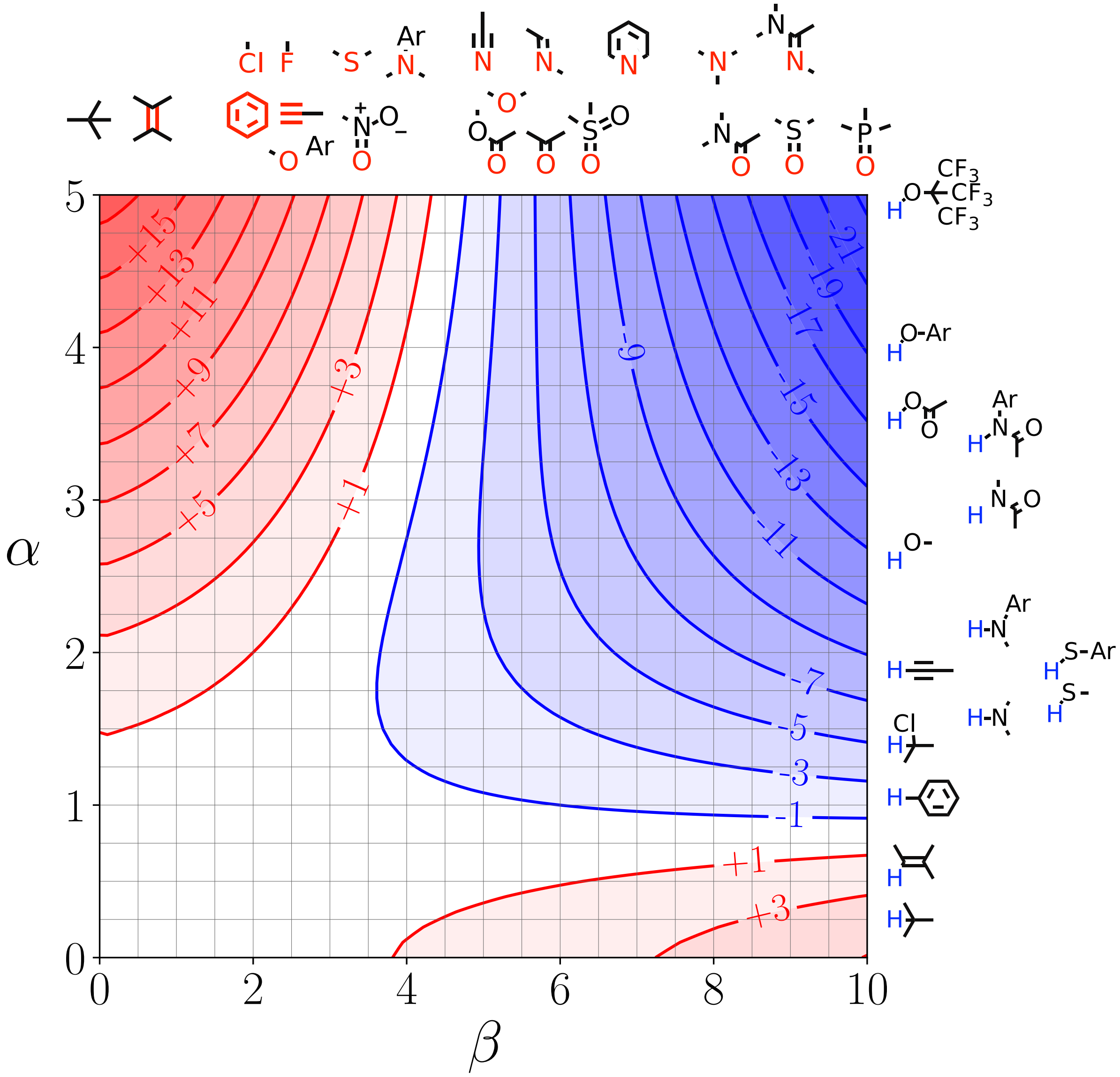

Figure S109: FGIP for benzyl methyl ketone at 298K.

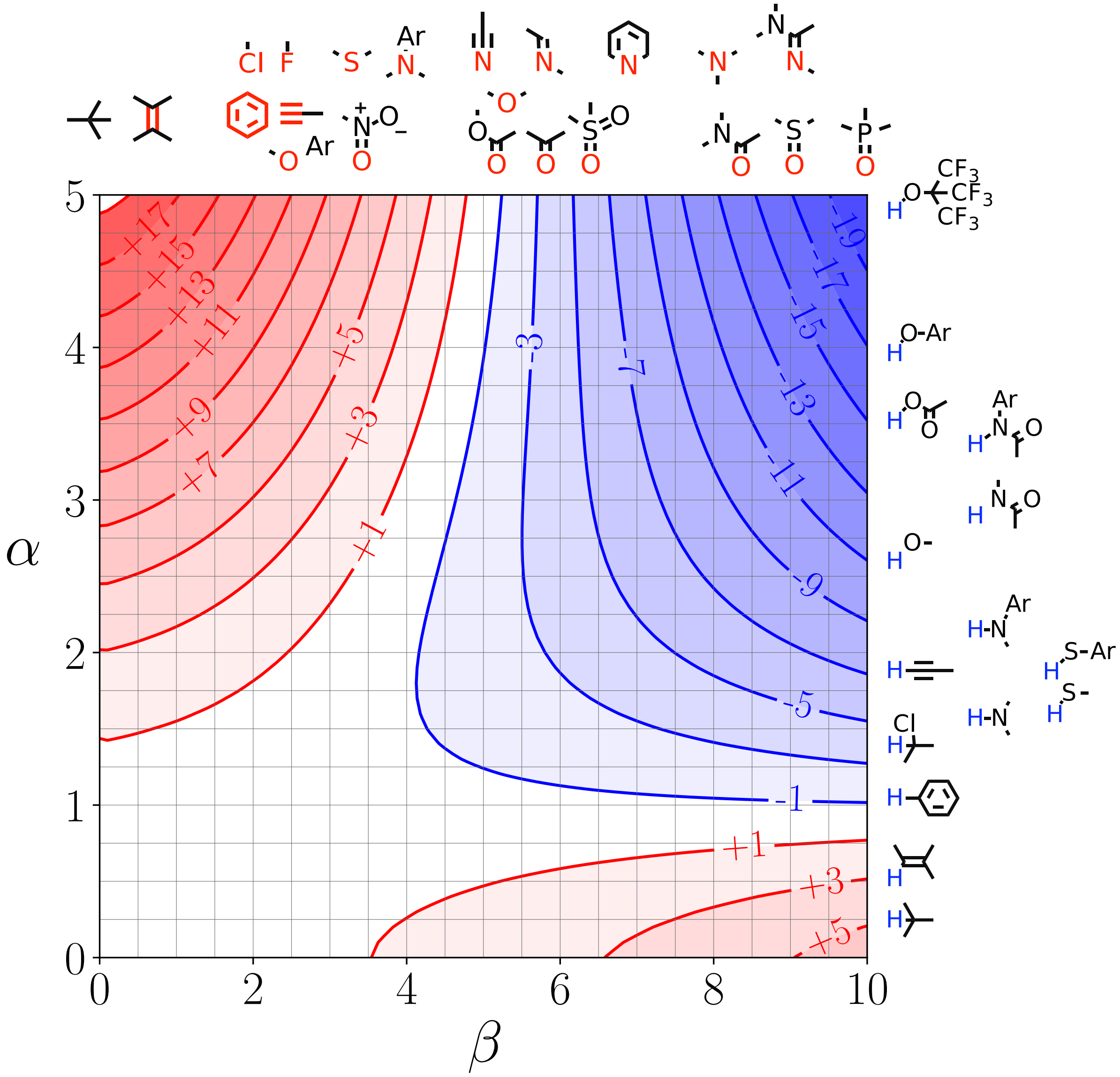

Figure S110: FGIP for 2,4,5-trimethylacetophenone at 298K.

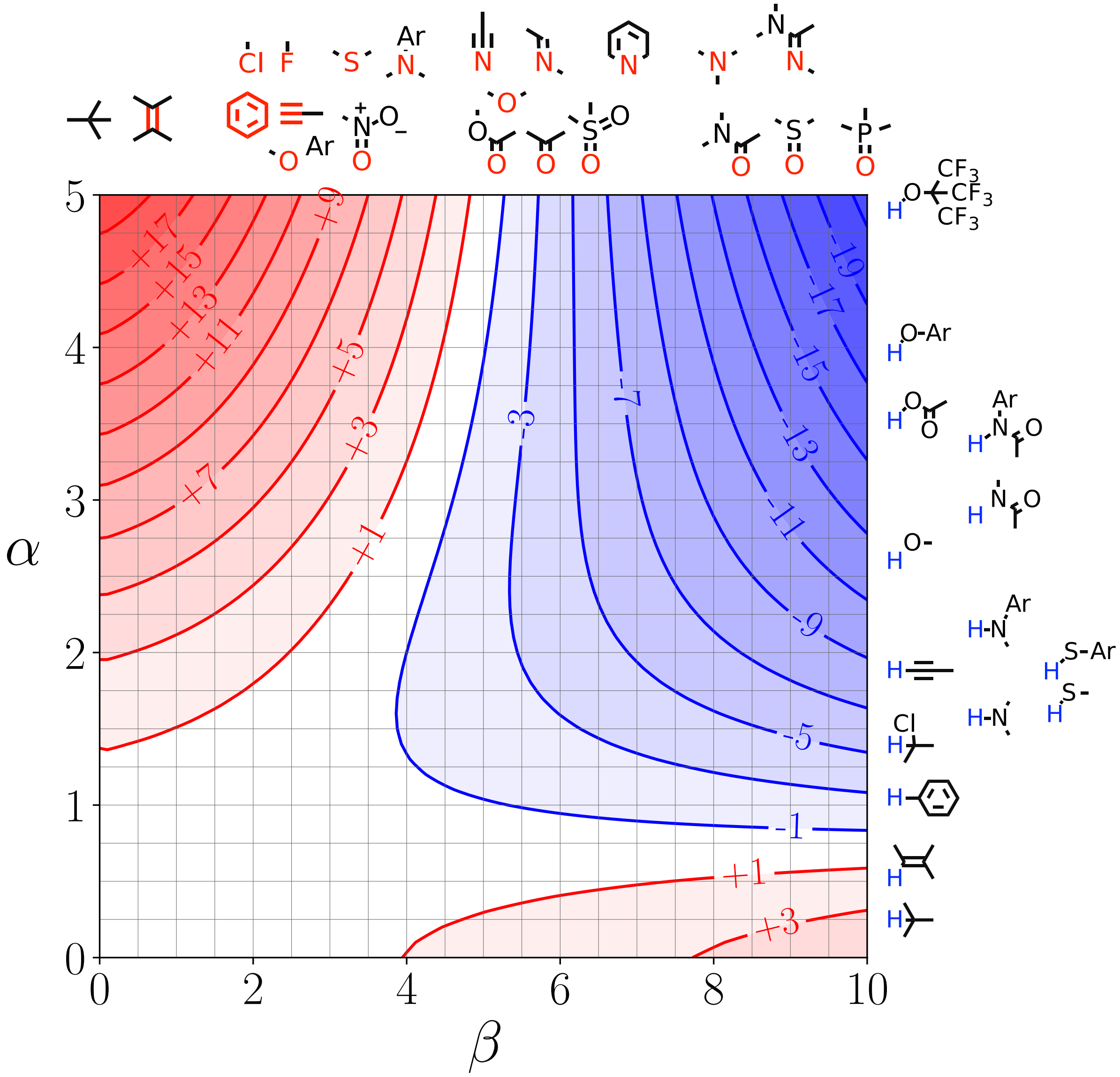

Figure S111: FGIP for p-chloroacetophenone at 298K.

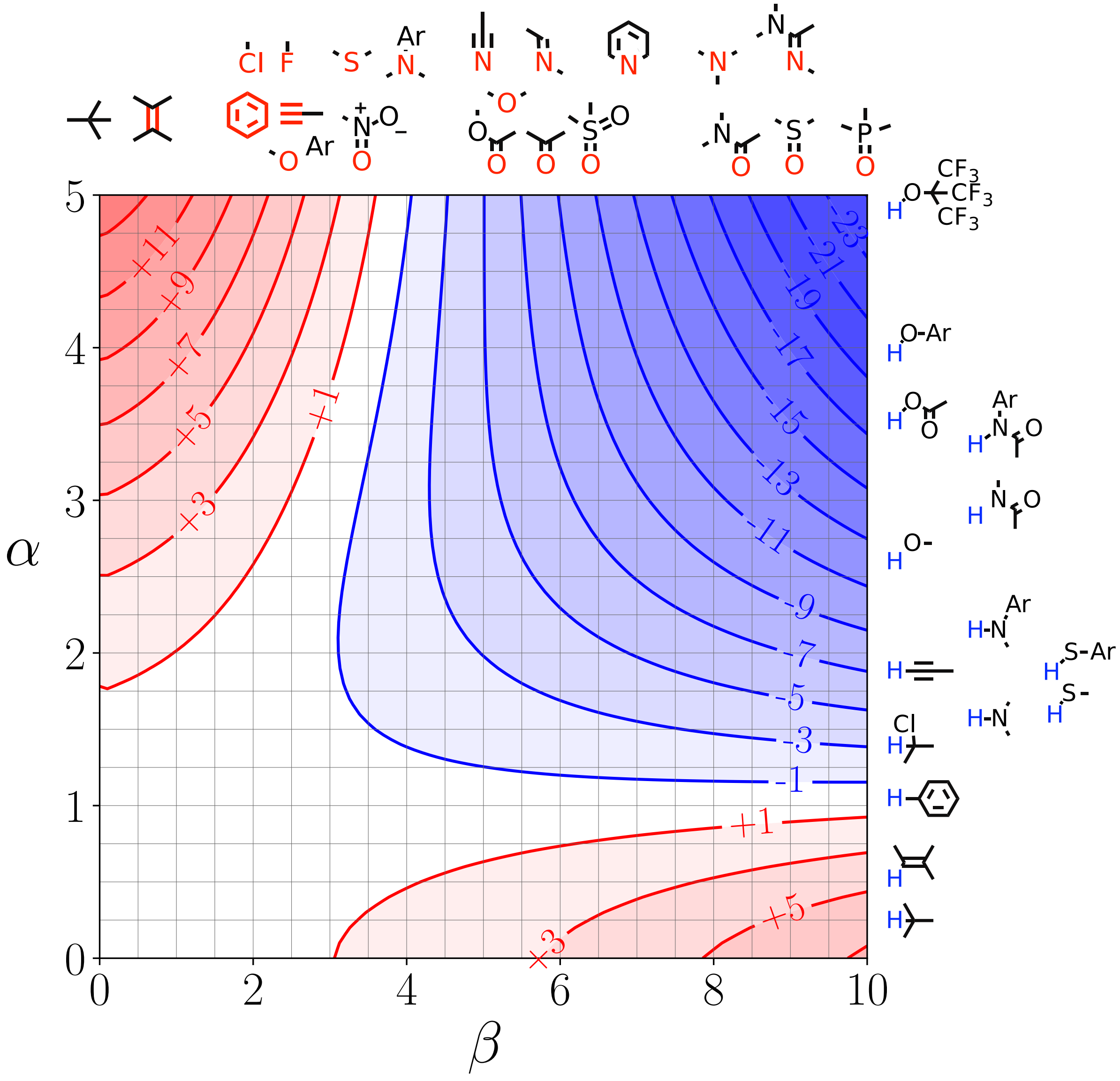

Figure S112: FGIP for diphenyl ketone at 298K.

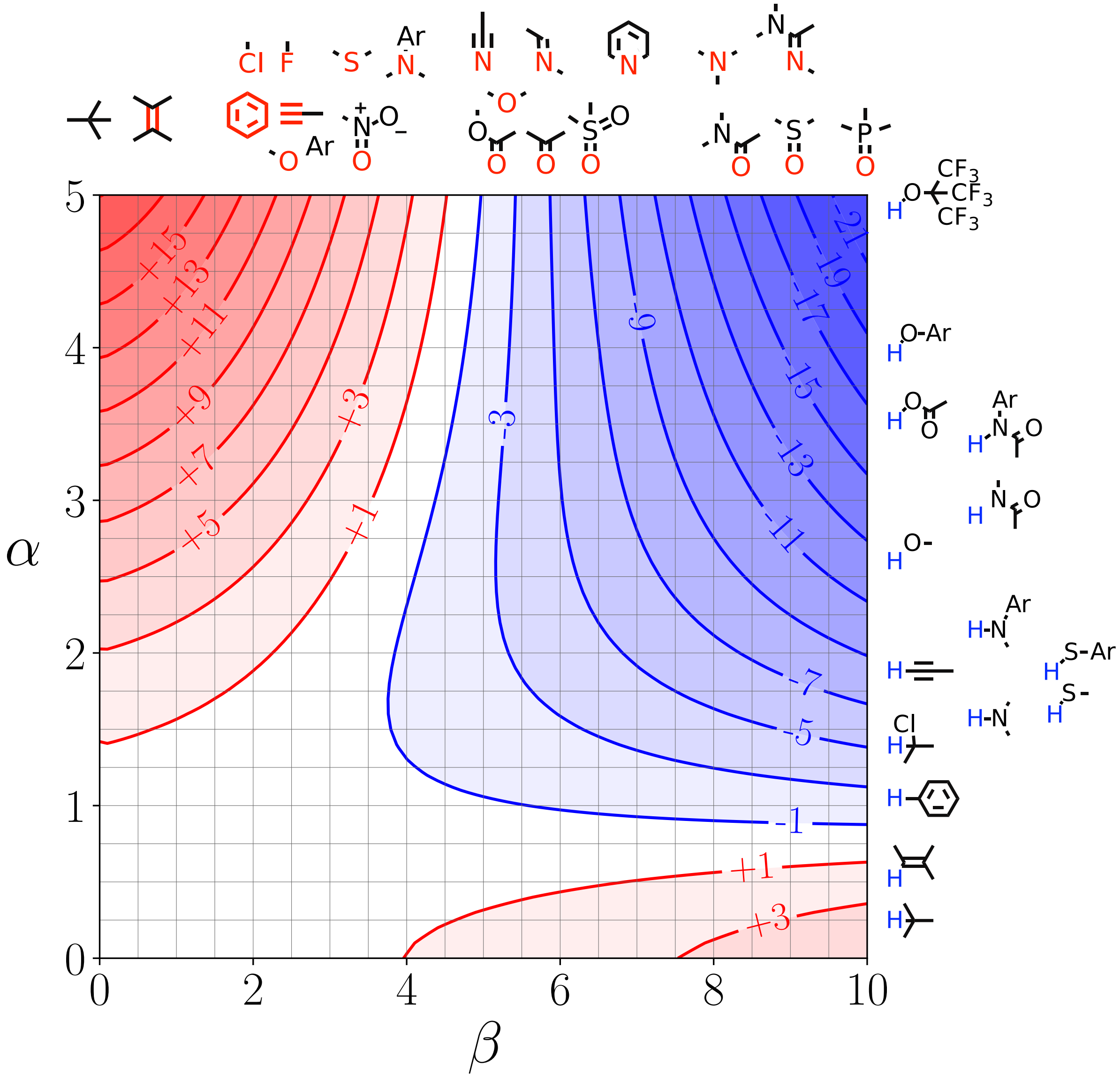

Figure S113: FGIP for 2,4-pentanedione at 298K.

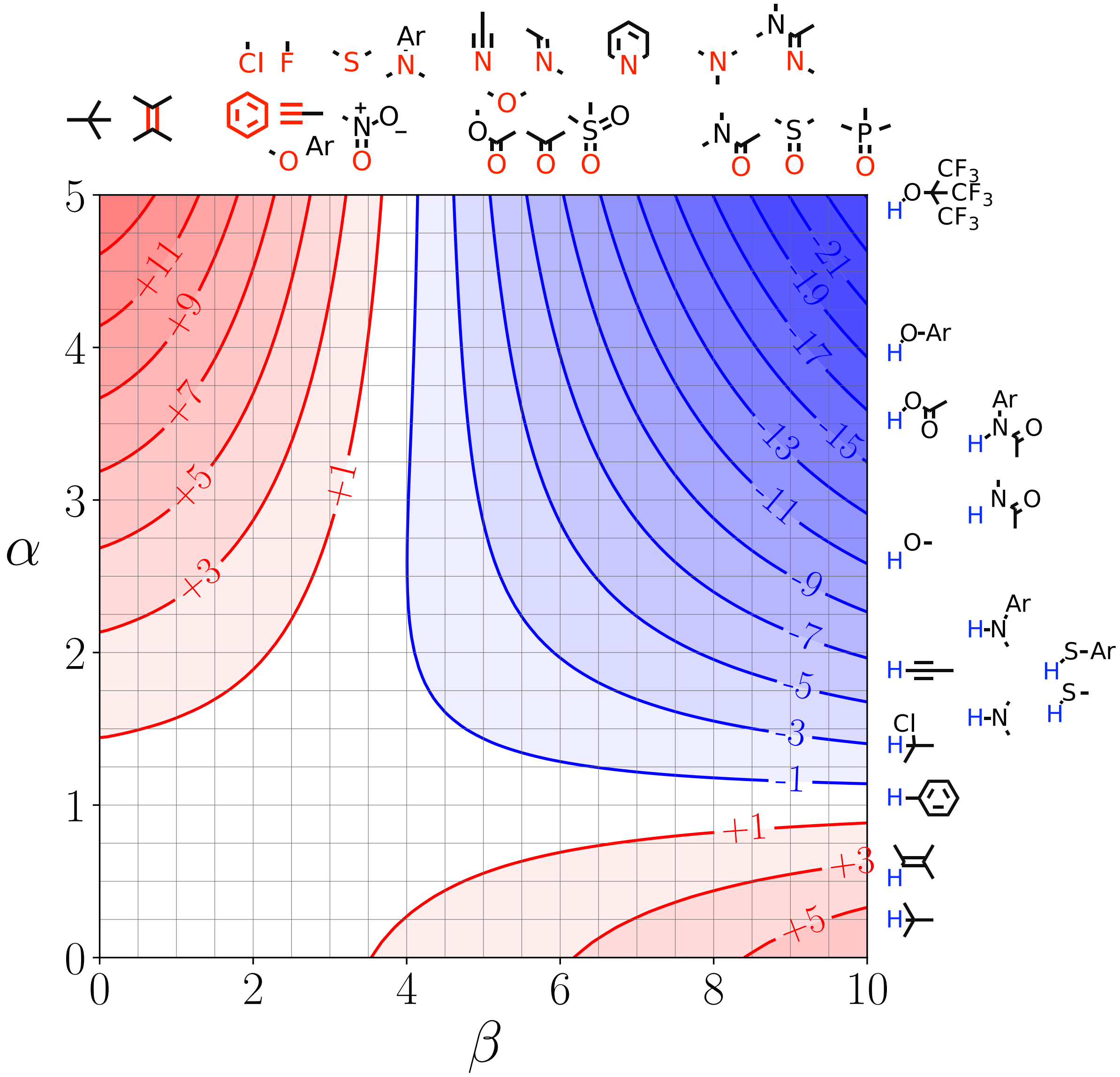

Figure S114: FGIP for 2,3-butanedione at 298K.

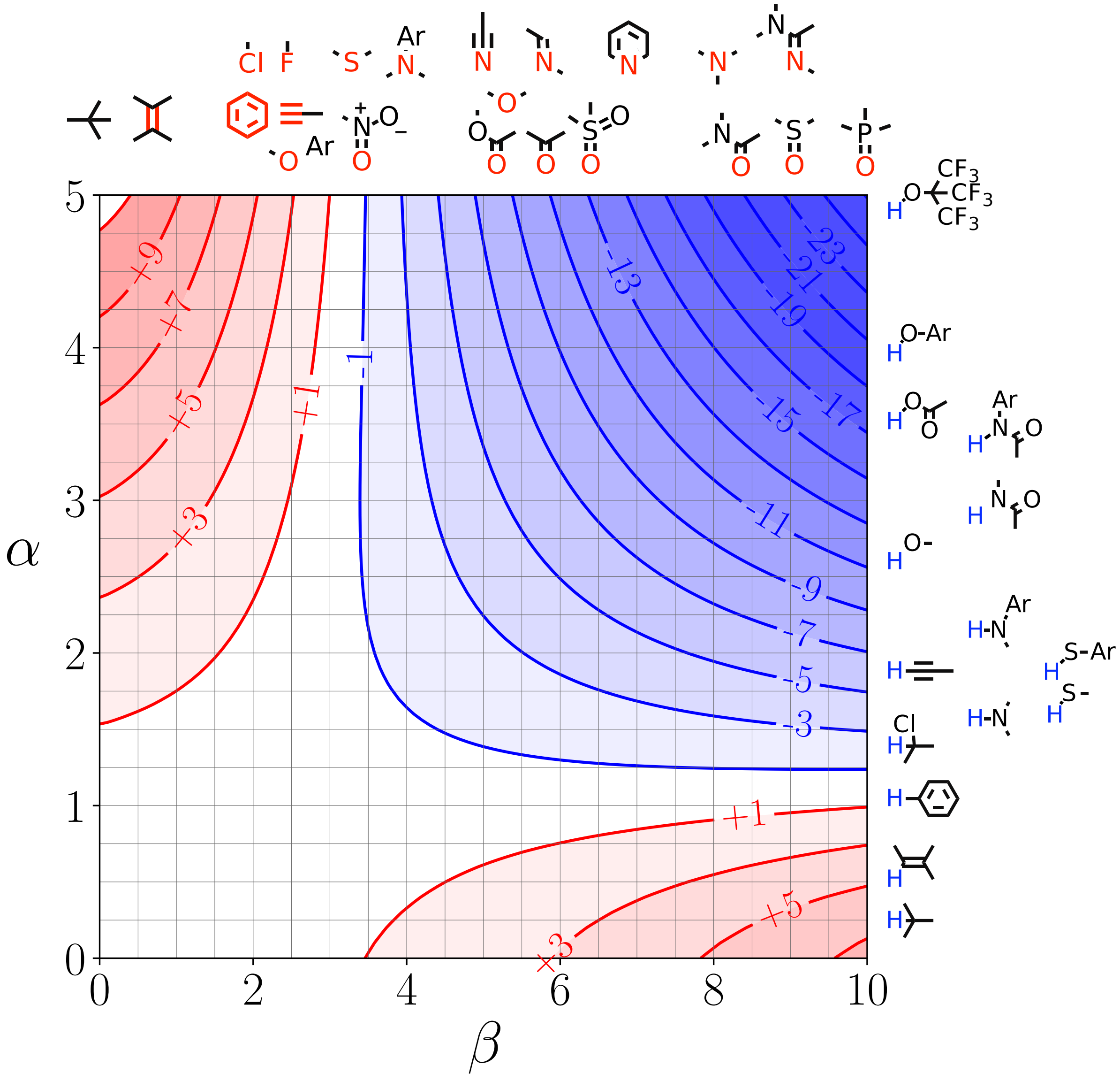

Figure S115: FGIP for formic acid at 298K.

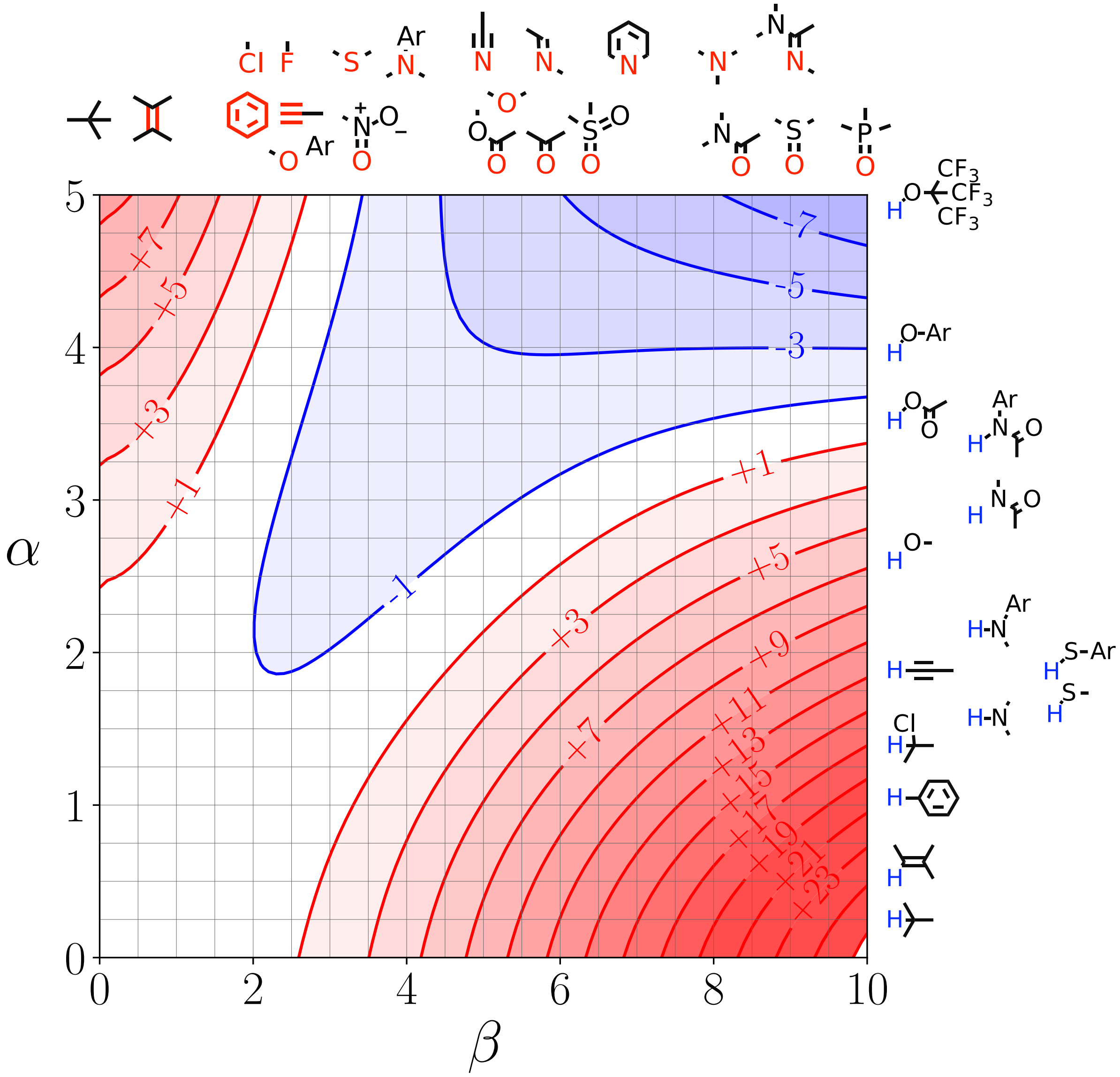

Figure S116: FGIP for acetic acid at 298K.

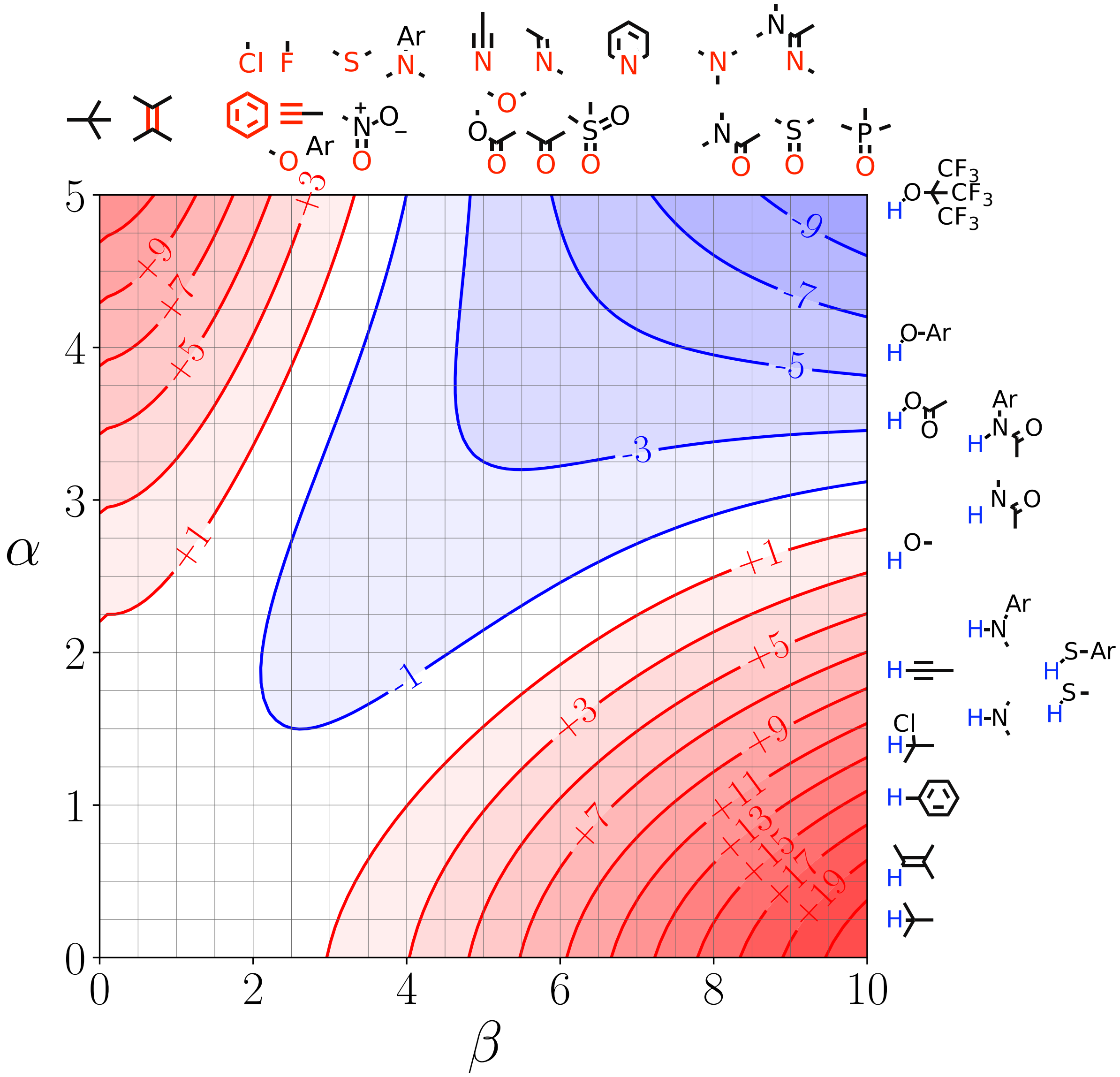

Figure S117: FGIP for propanoic acid at 298K.

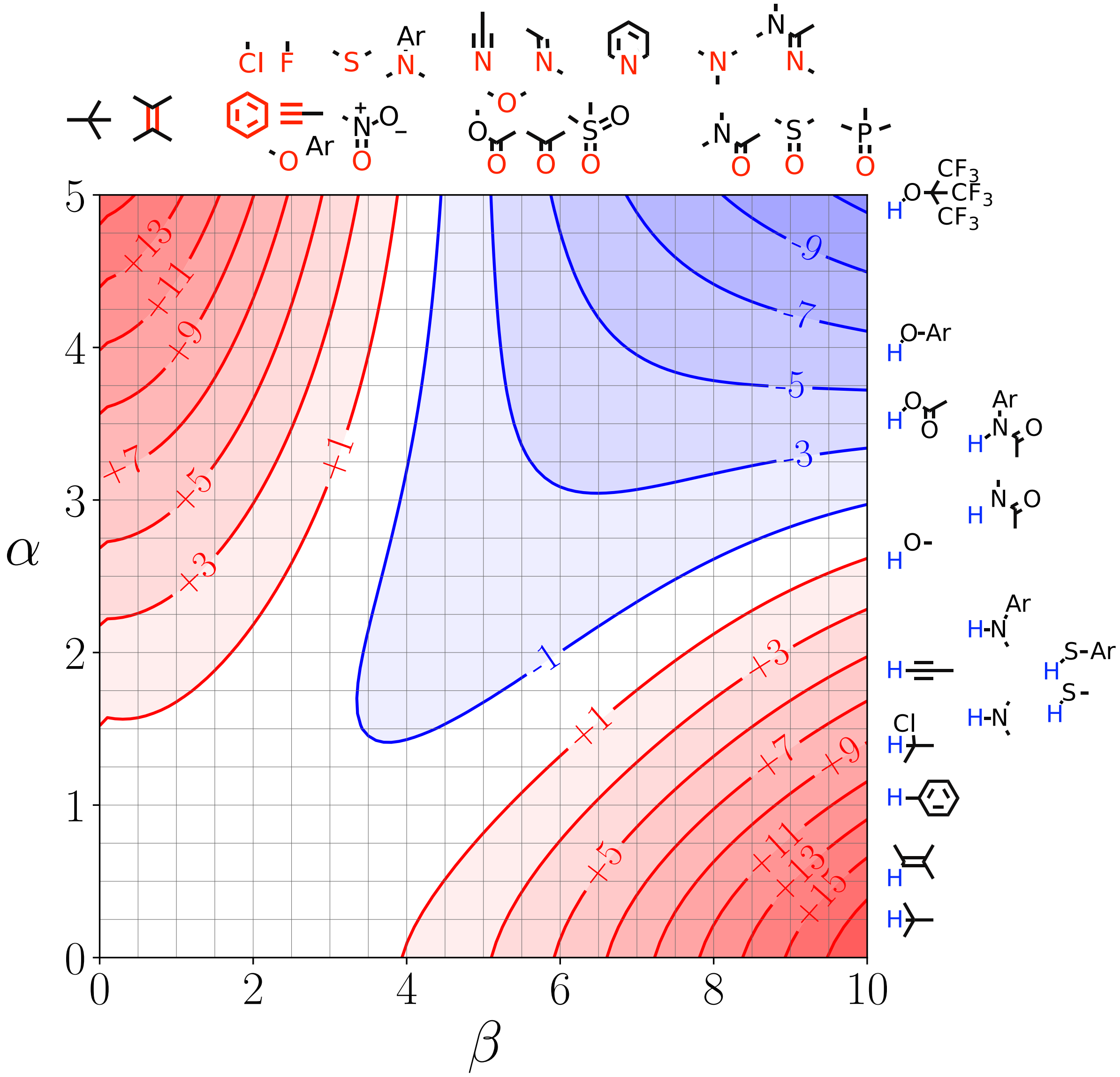

Figure S118: FGIP for butanoic acid at 298K.

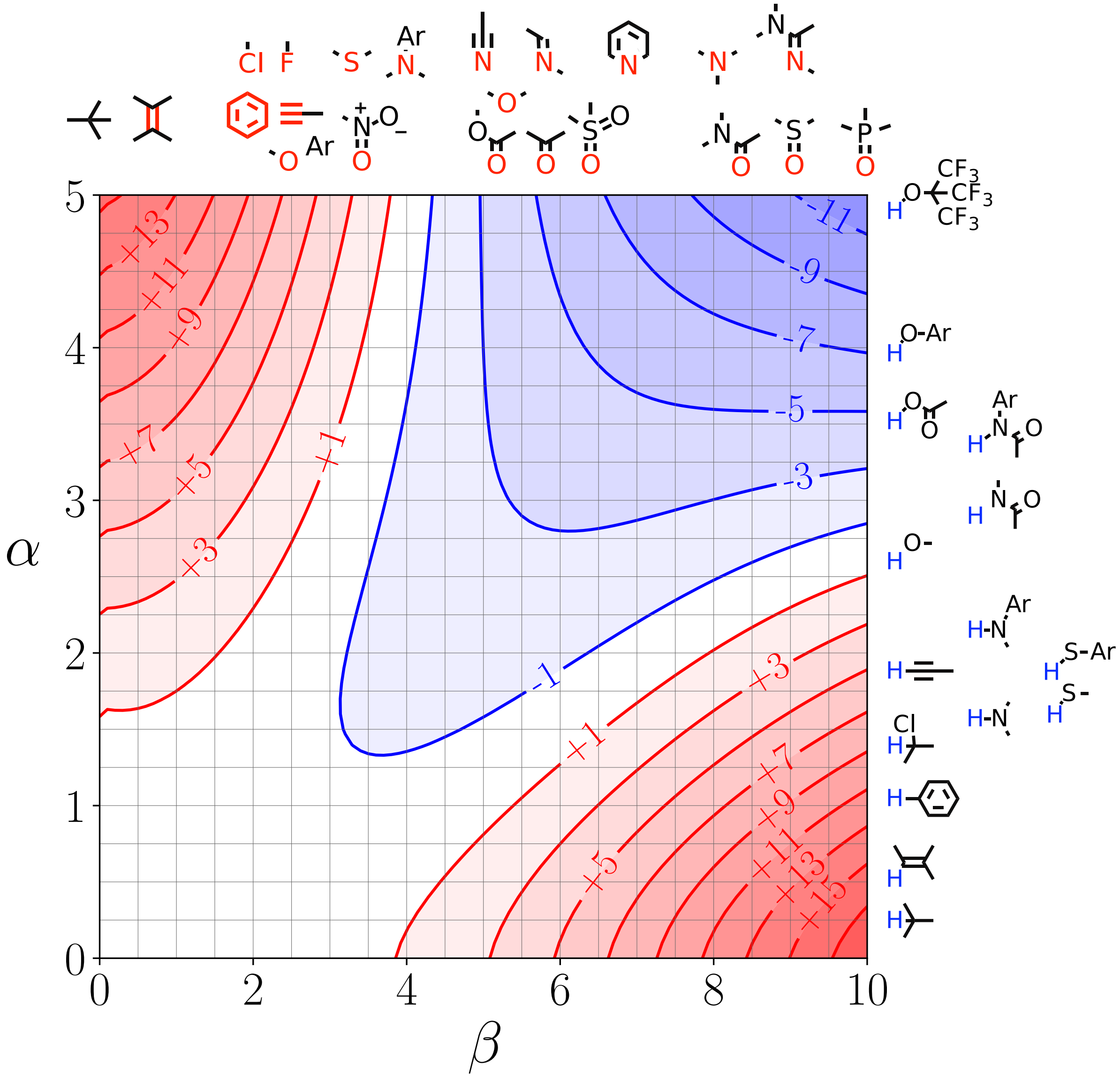

Figure S119: FGIP for pentanoic acid at 298K.

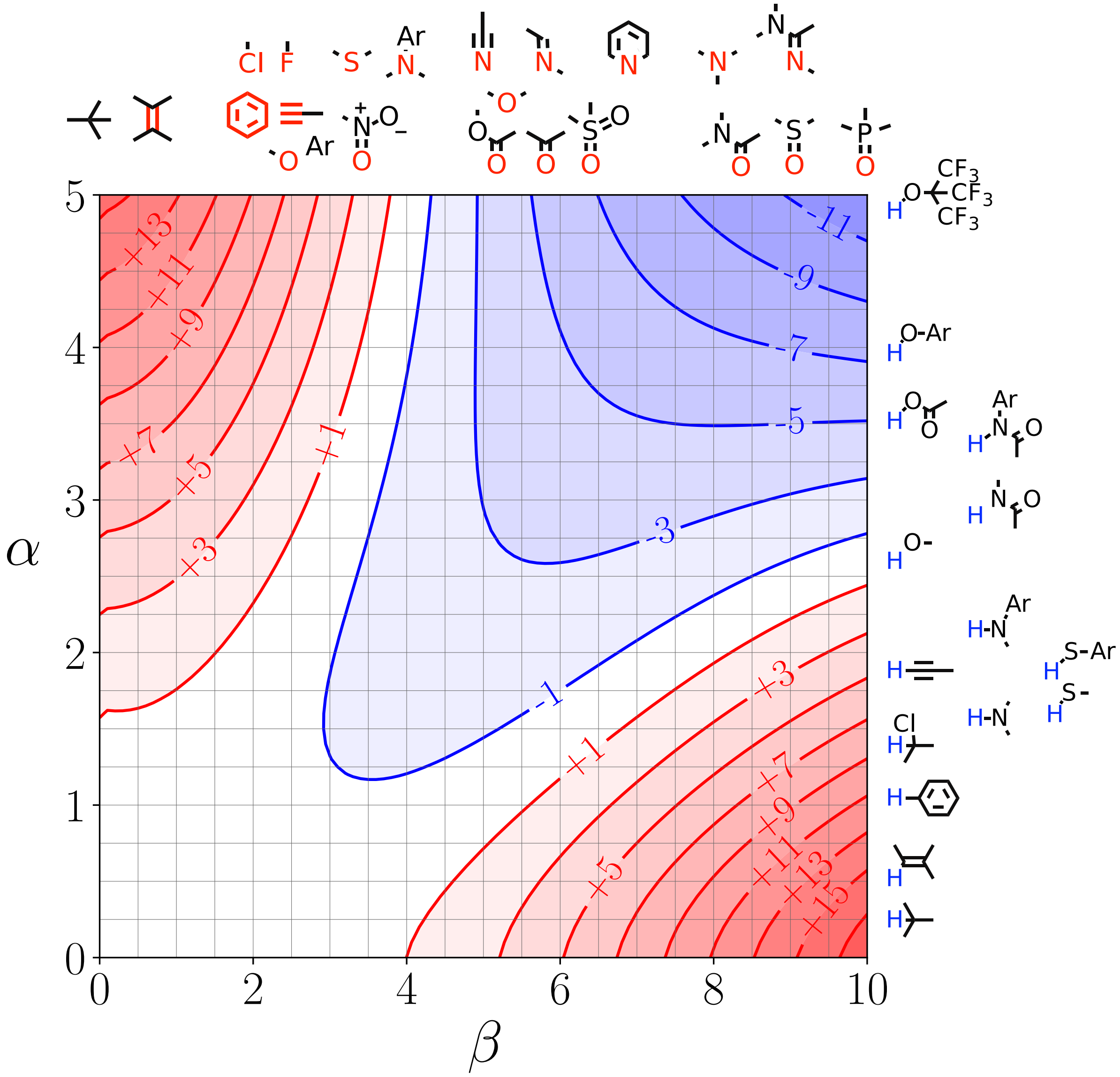

Figure S120: FGIP for hexanoic acid at 298K.

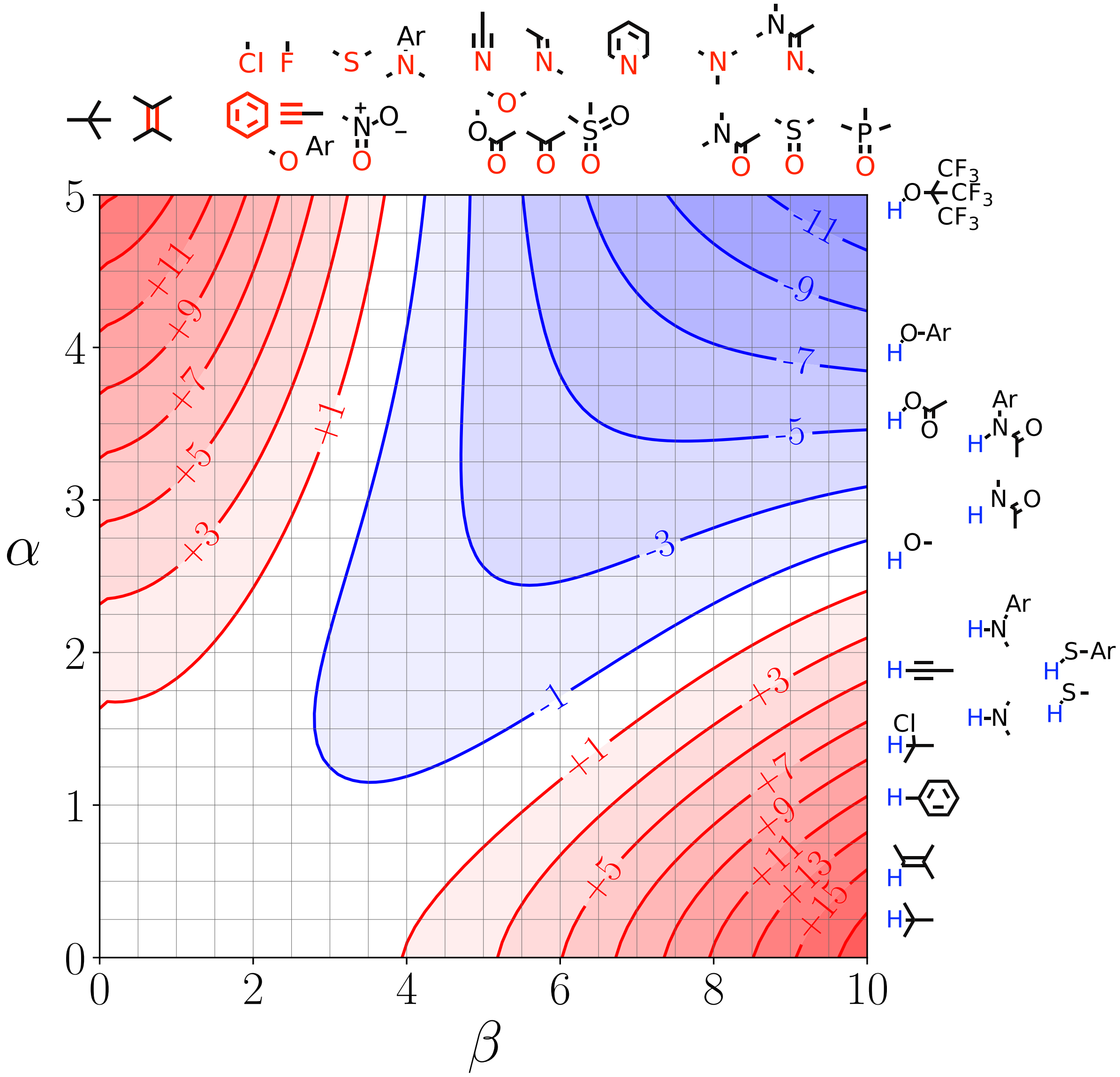

Figure S121: FGIP for heptanoic acid at 298K.

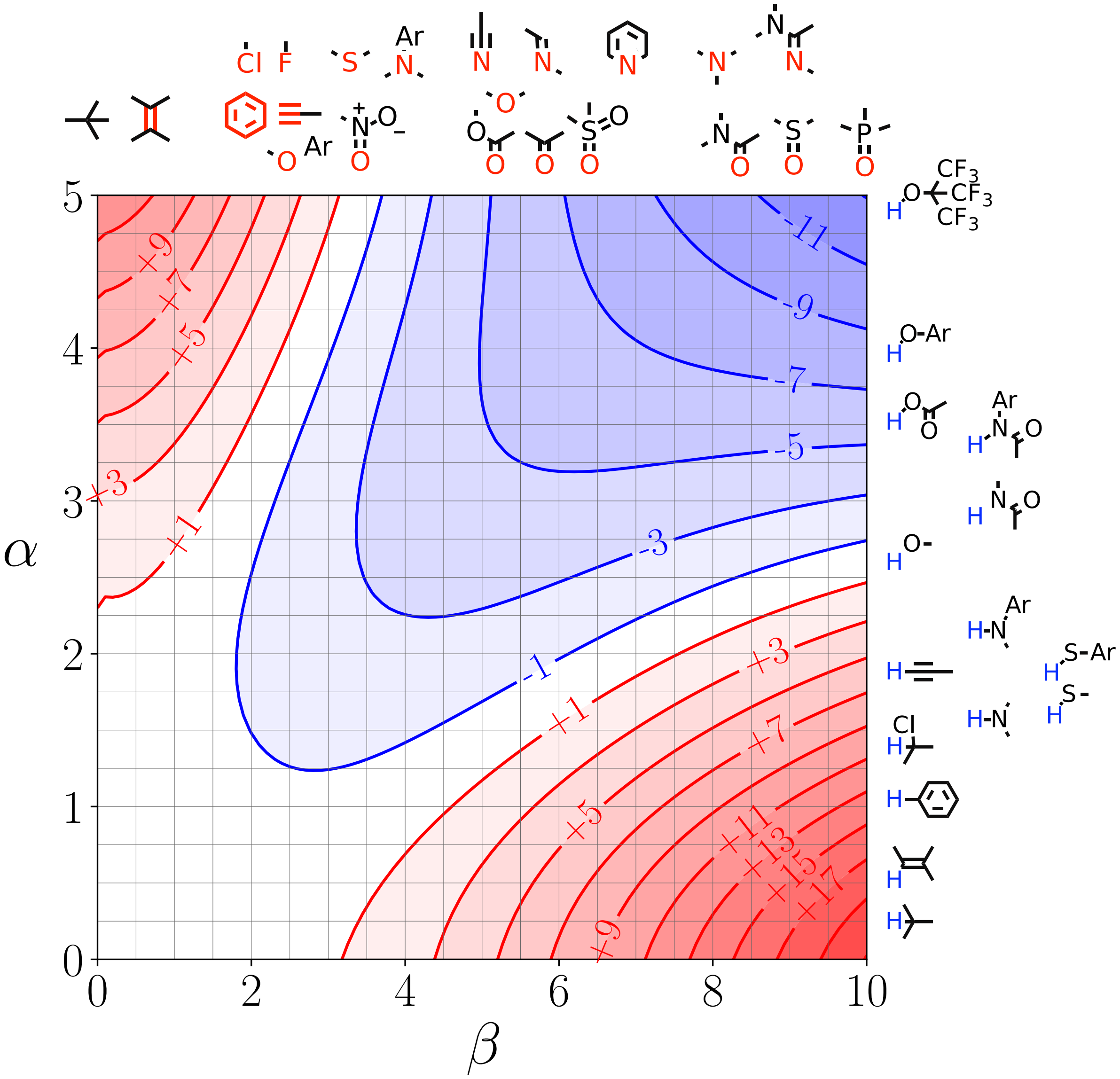

Figure S122: FGIP for dichloroacetic acid at 298K.

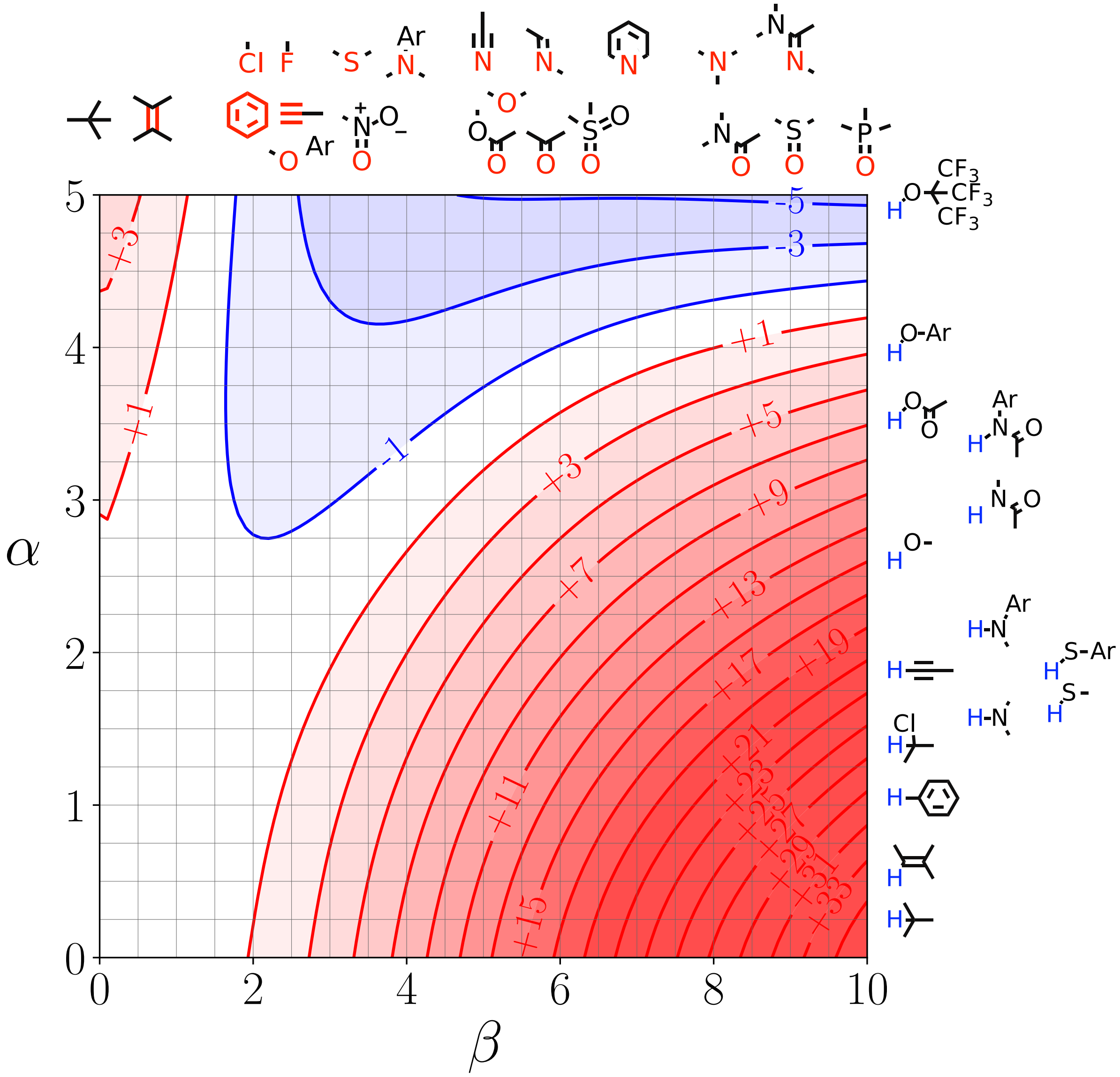

Figure S123: FGIP for trifluoroacetic acid at 298K.

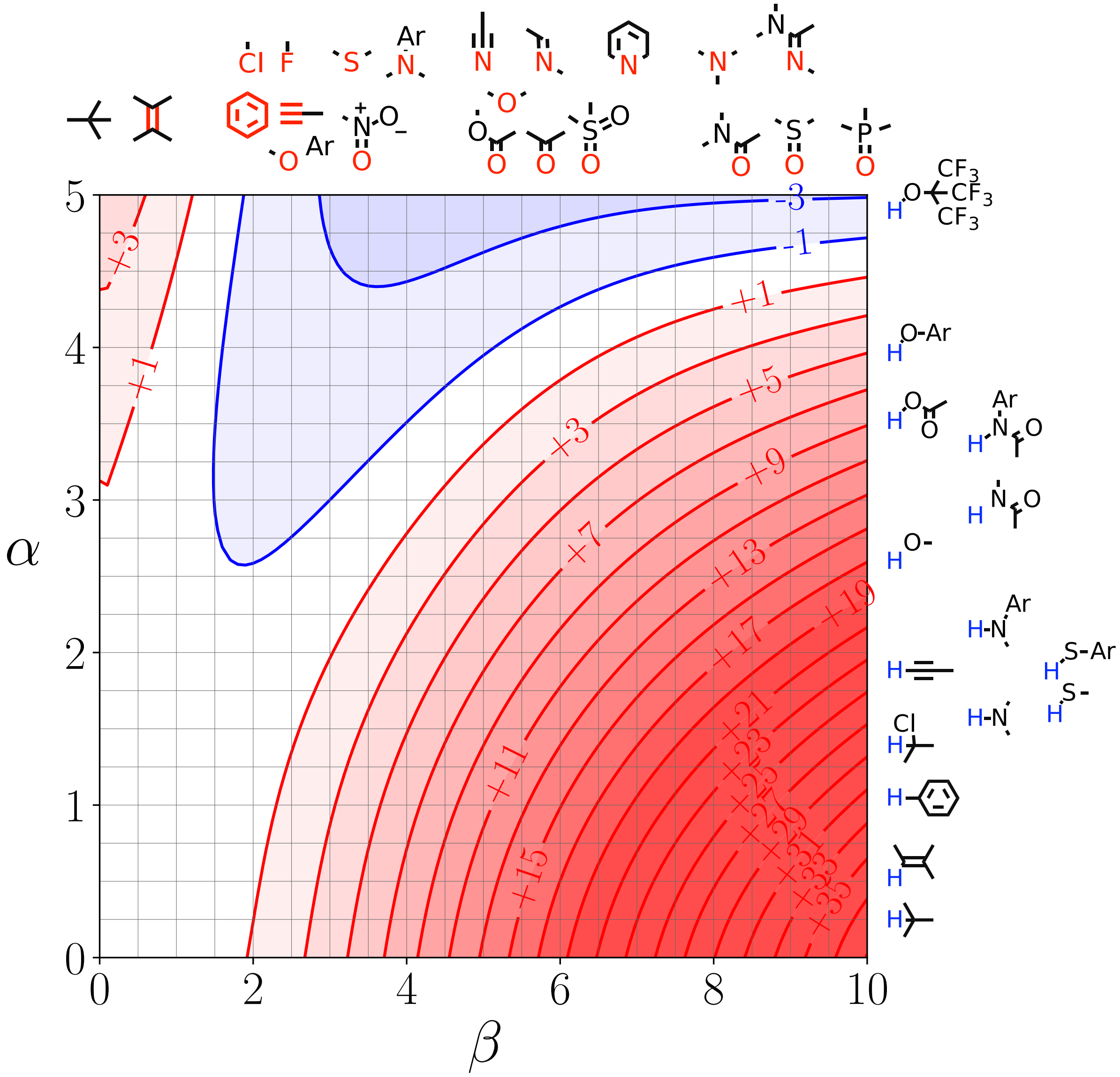

Figure S124: FGIP for acetic anhydride at 298K.

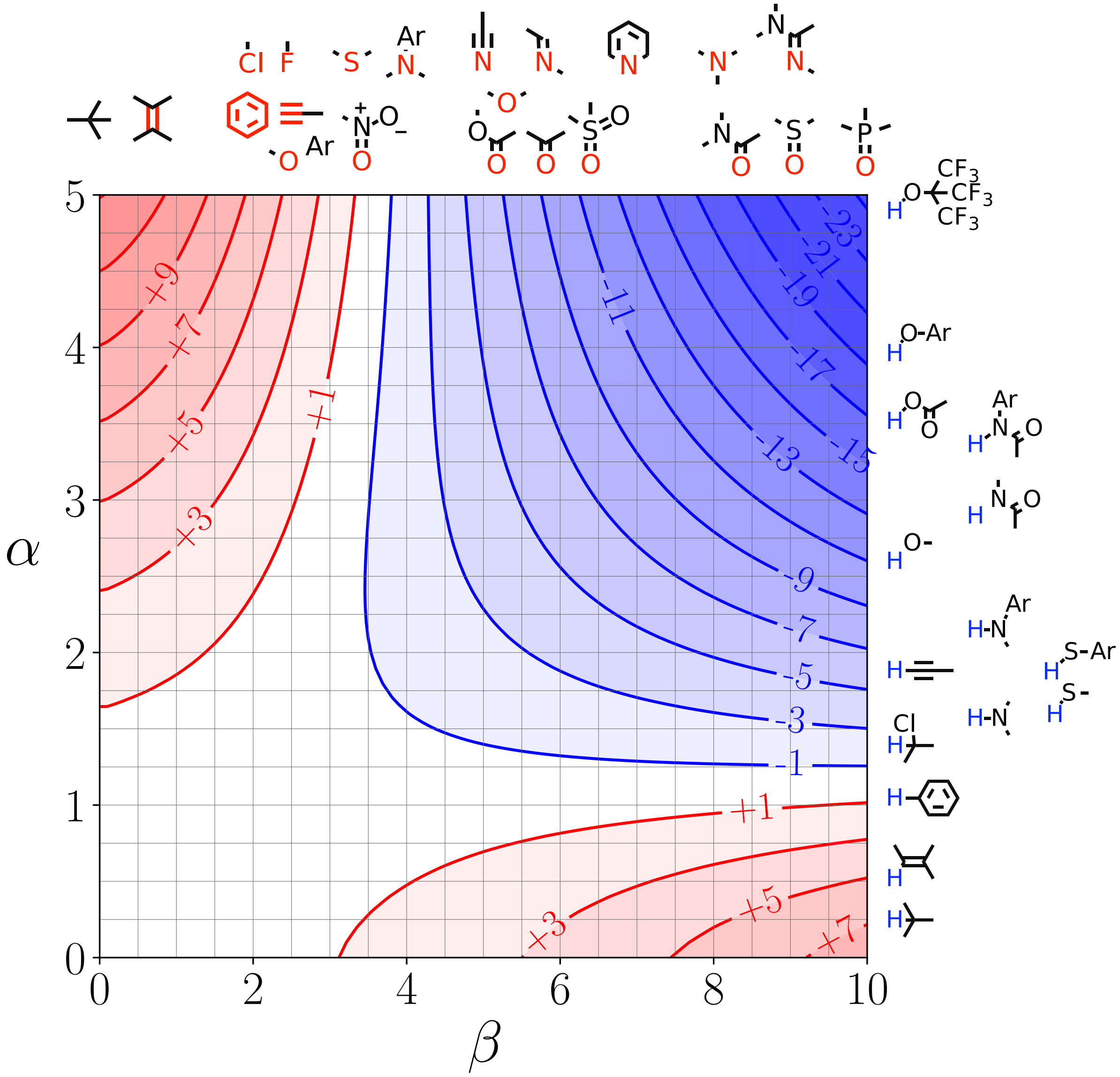

Figure S125: FGIP for benzoyl chloride at 298K.

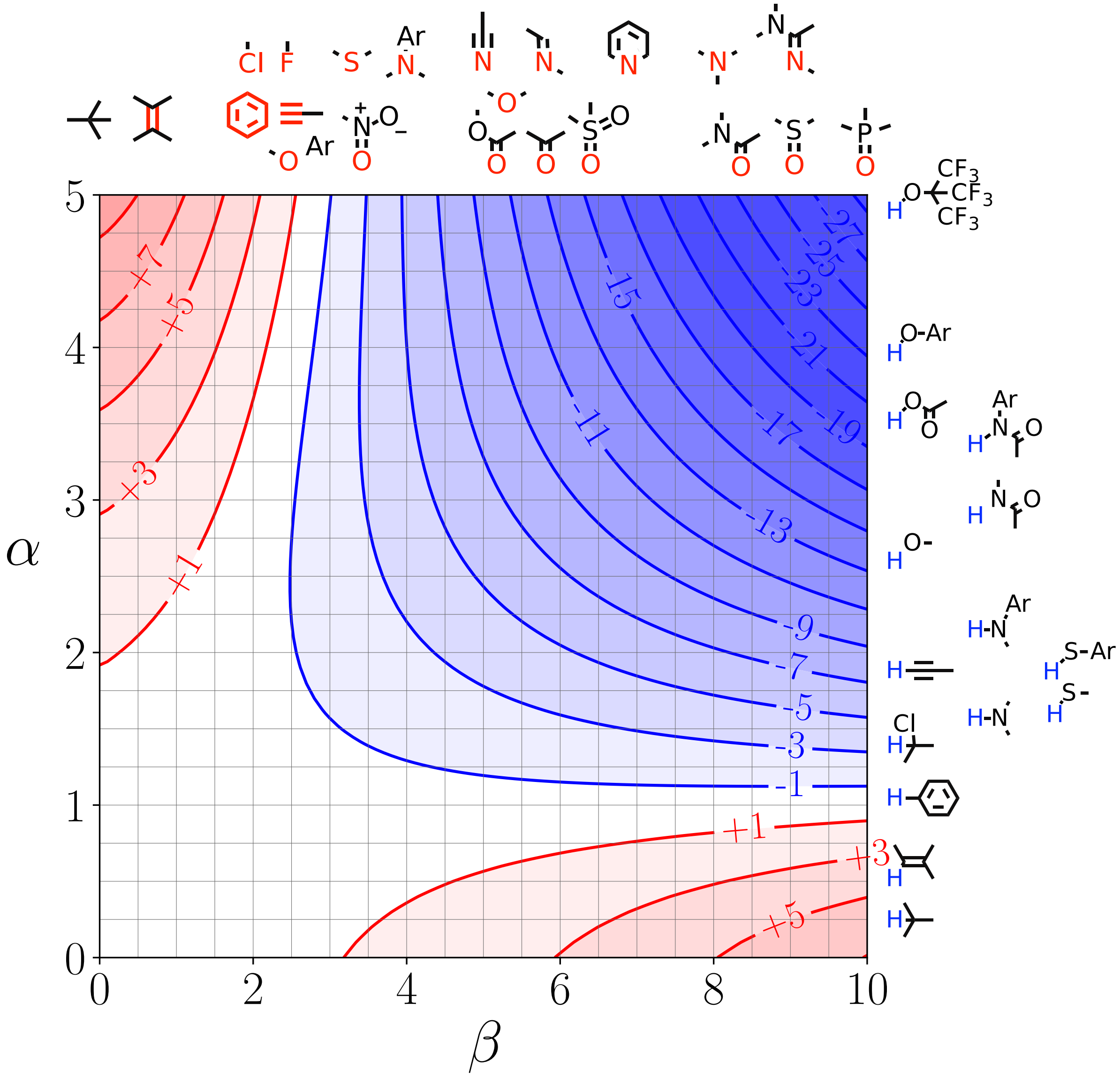

Figure S126: FGIP for benzoyl bromide at 298K.

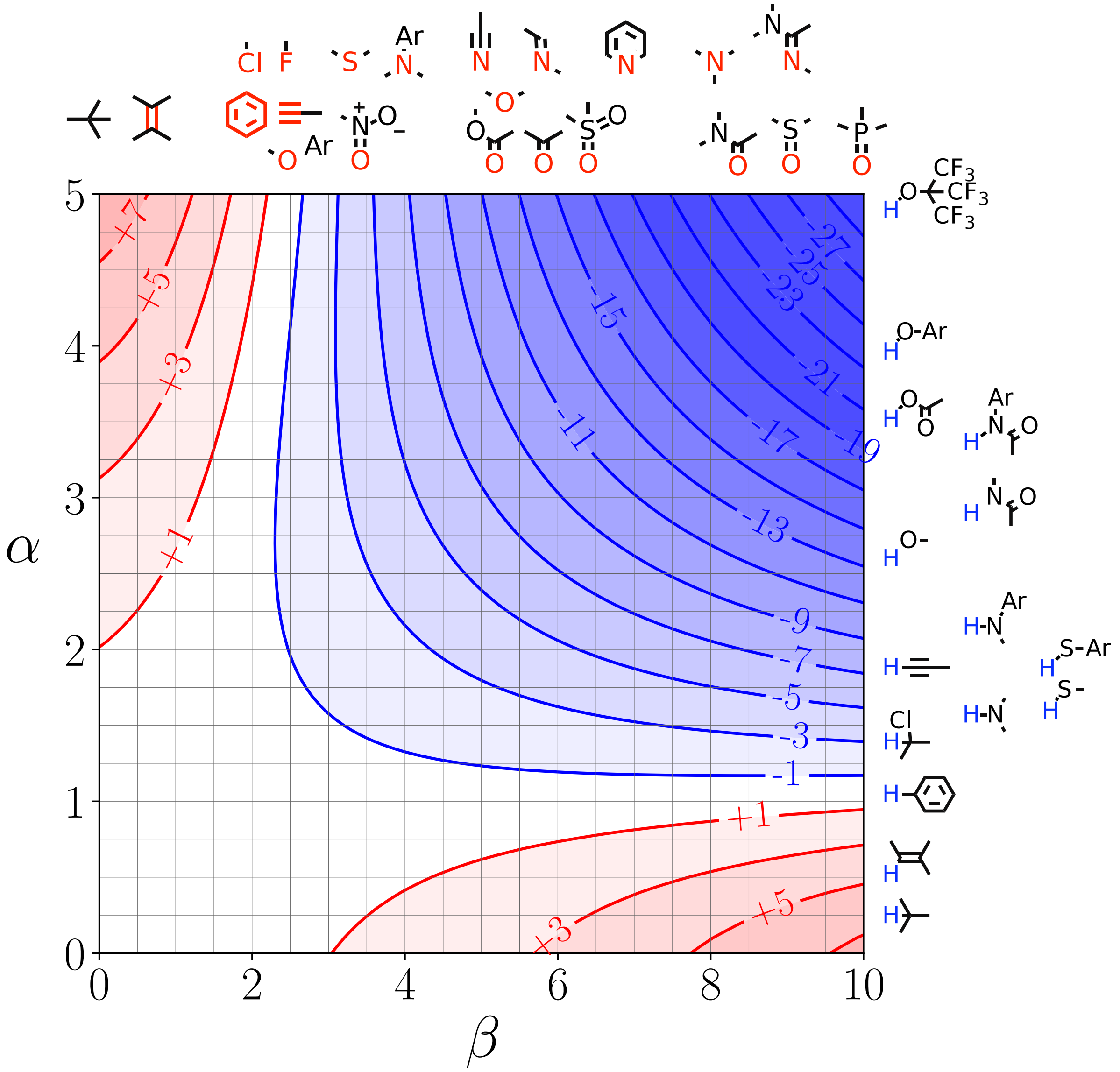

Figure S127: FGIP for methyl formate at 298K.

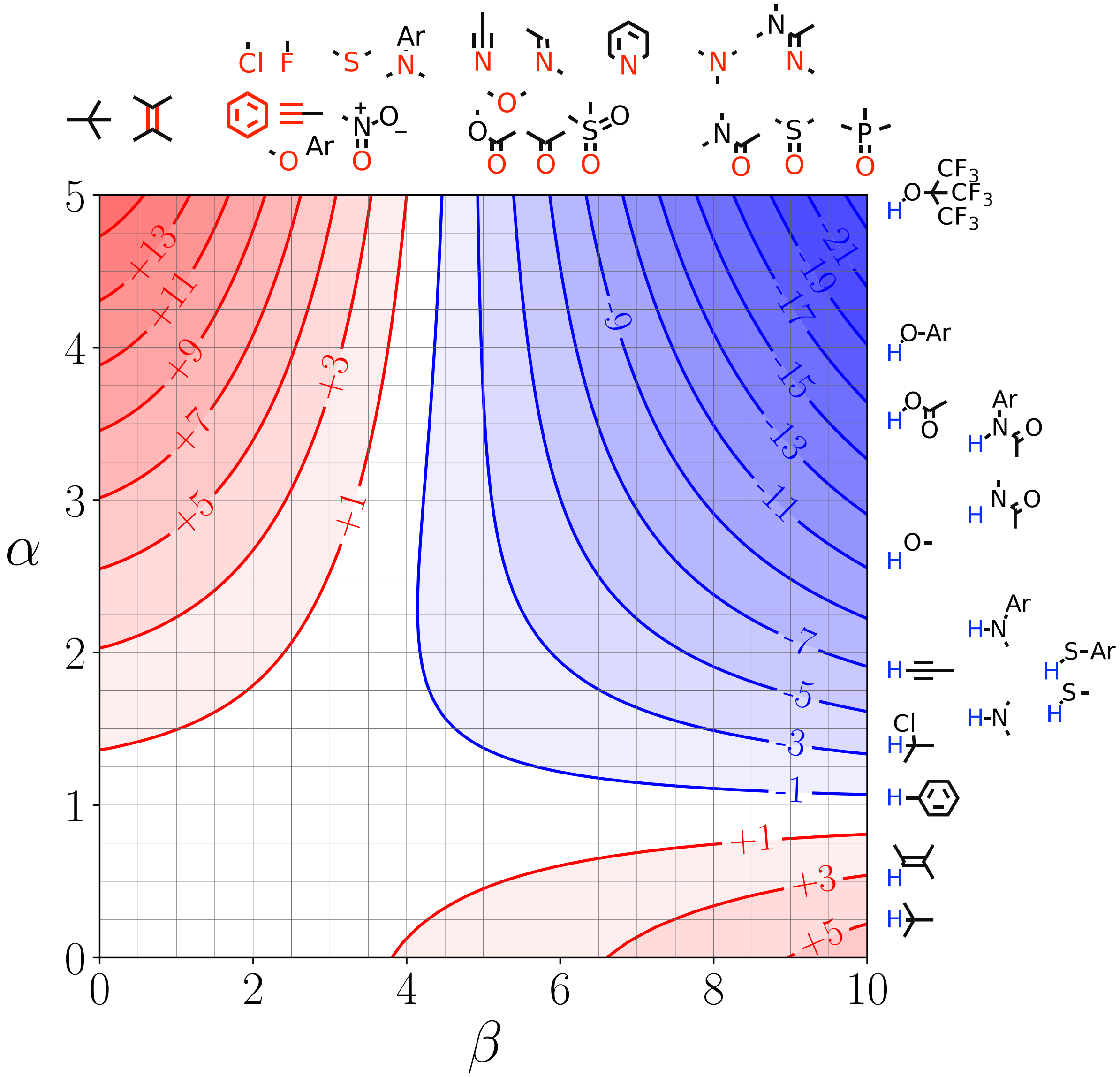

Figure S128: FGIP for ethyl formate at 298K.

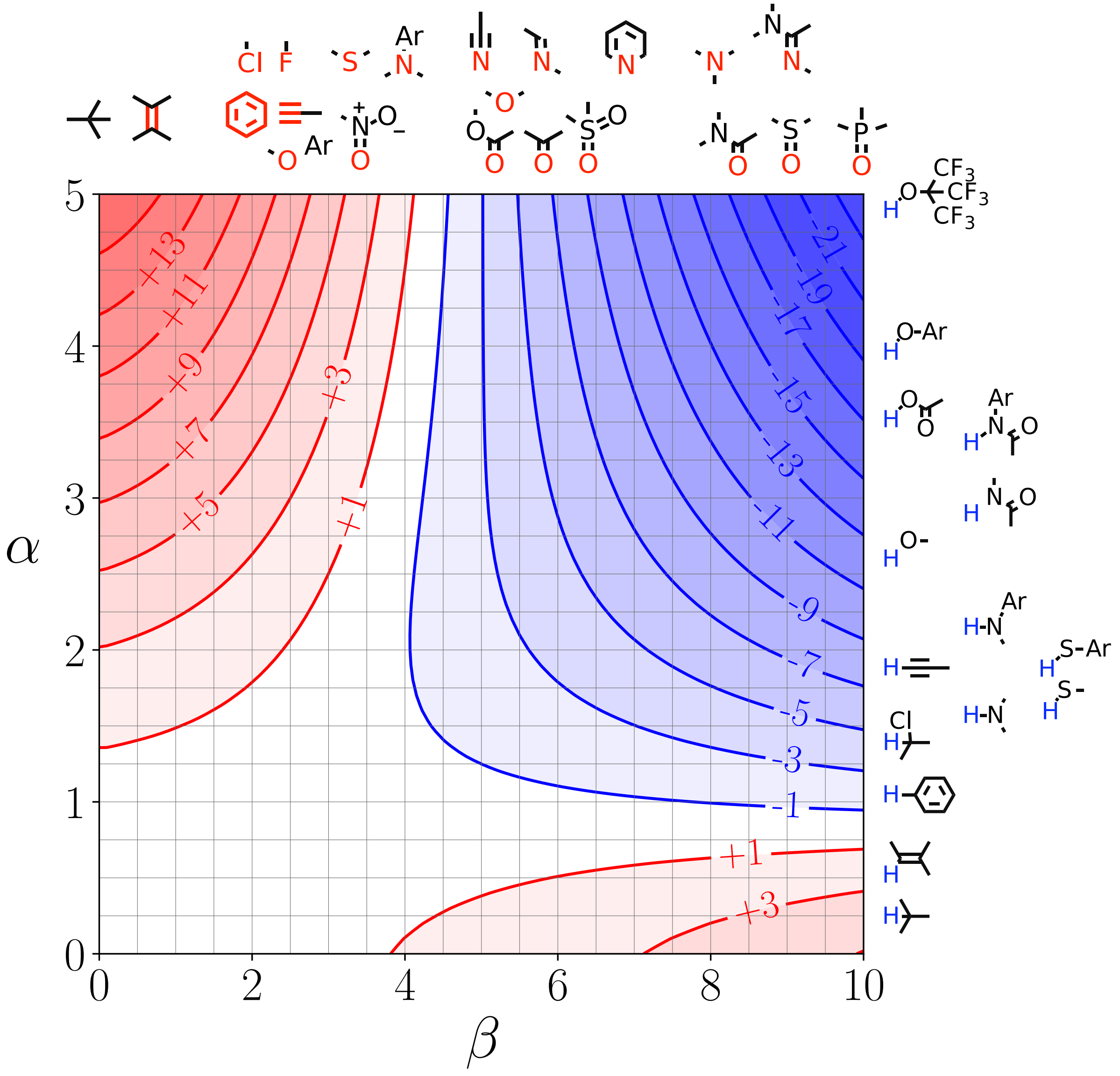

Figure S129: FGIP for methyl acetate at 298K.

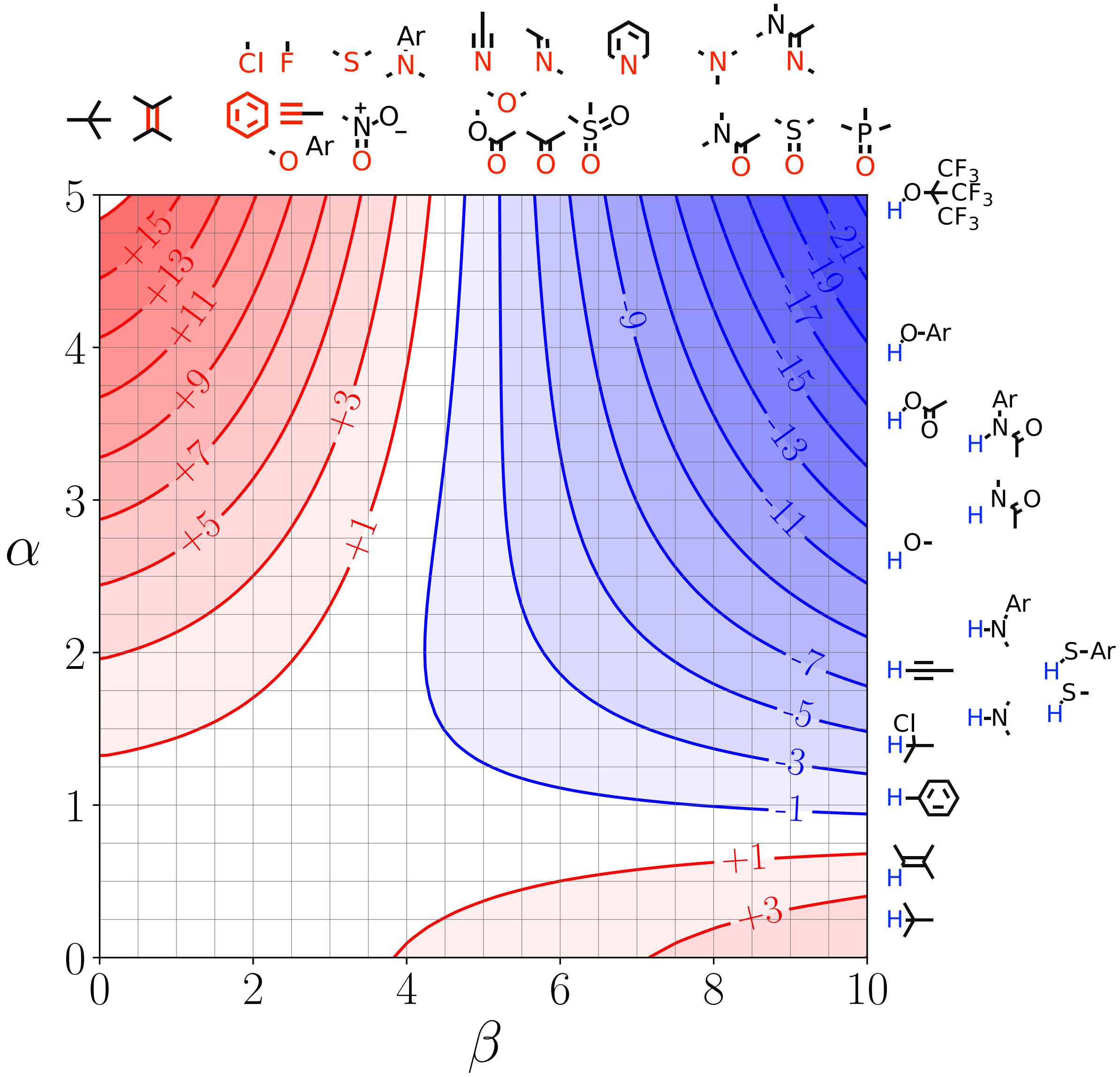

Figure S130: FGIP for ethyl acetate at 298K.

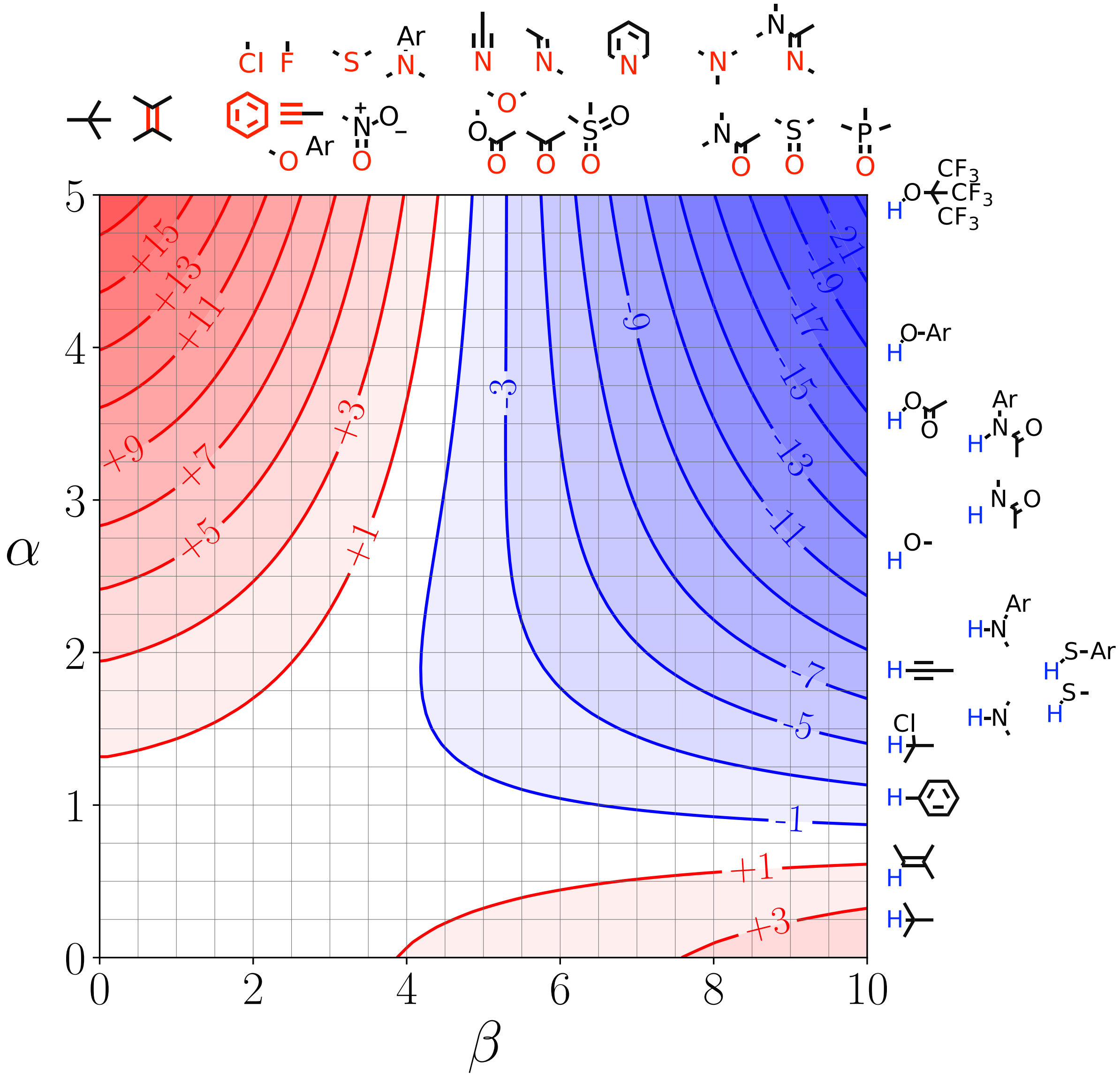

Figure S131: FGIP for n-propyl acetate at 298K.

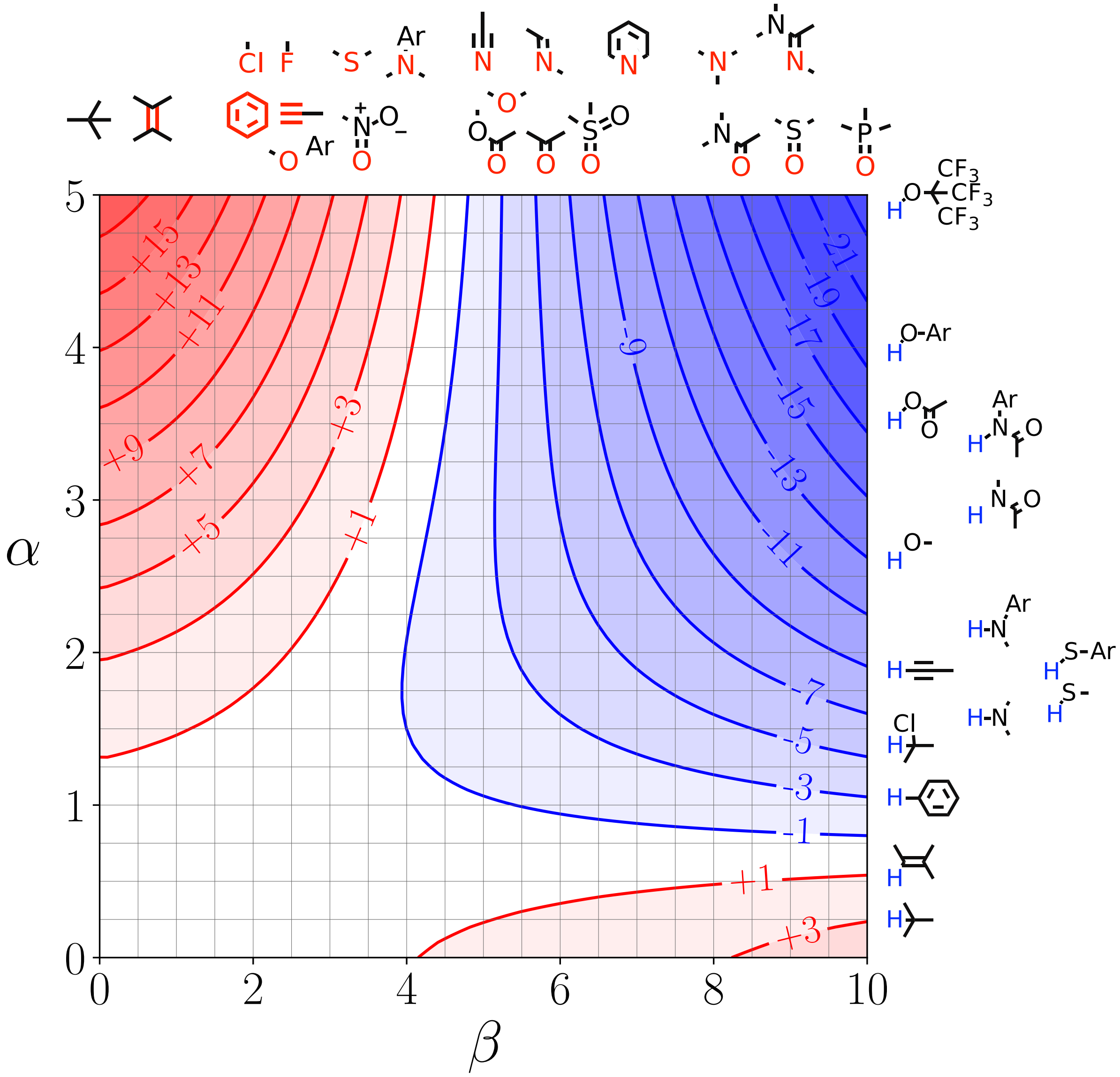

Figure S132: FGIP for butyl acetate at 298K.

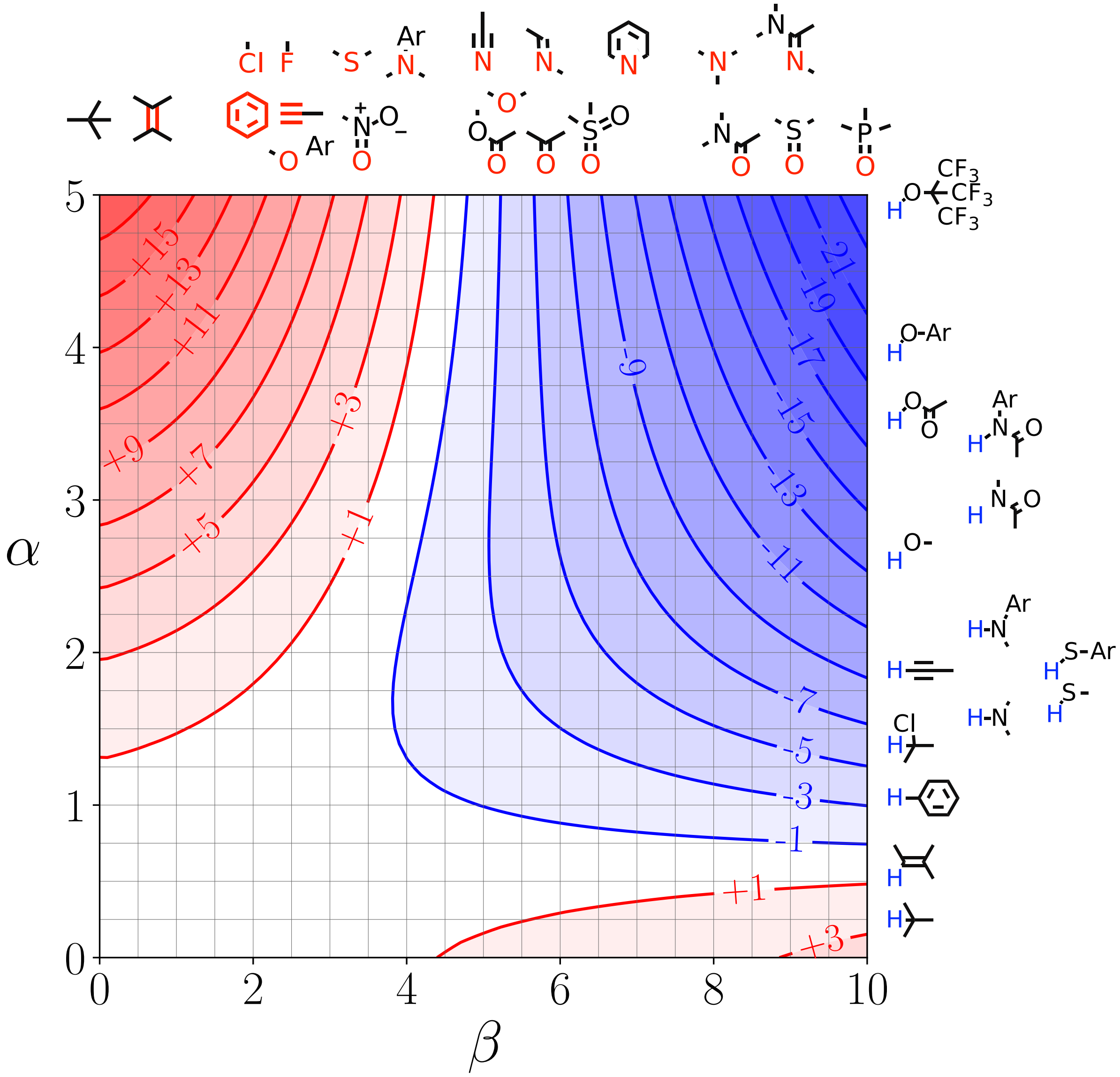

Figure S133: FGIP for isopentyl acetate at 298K.

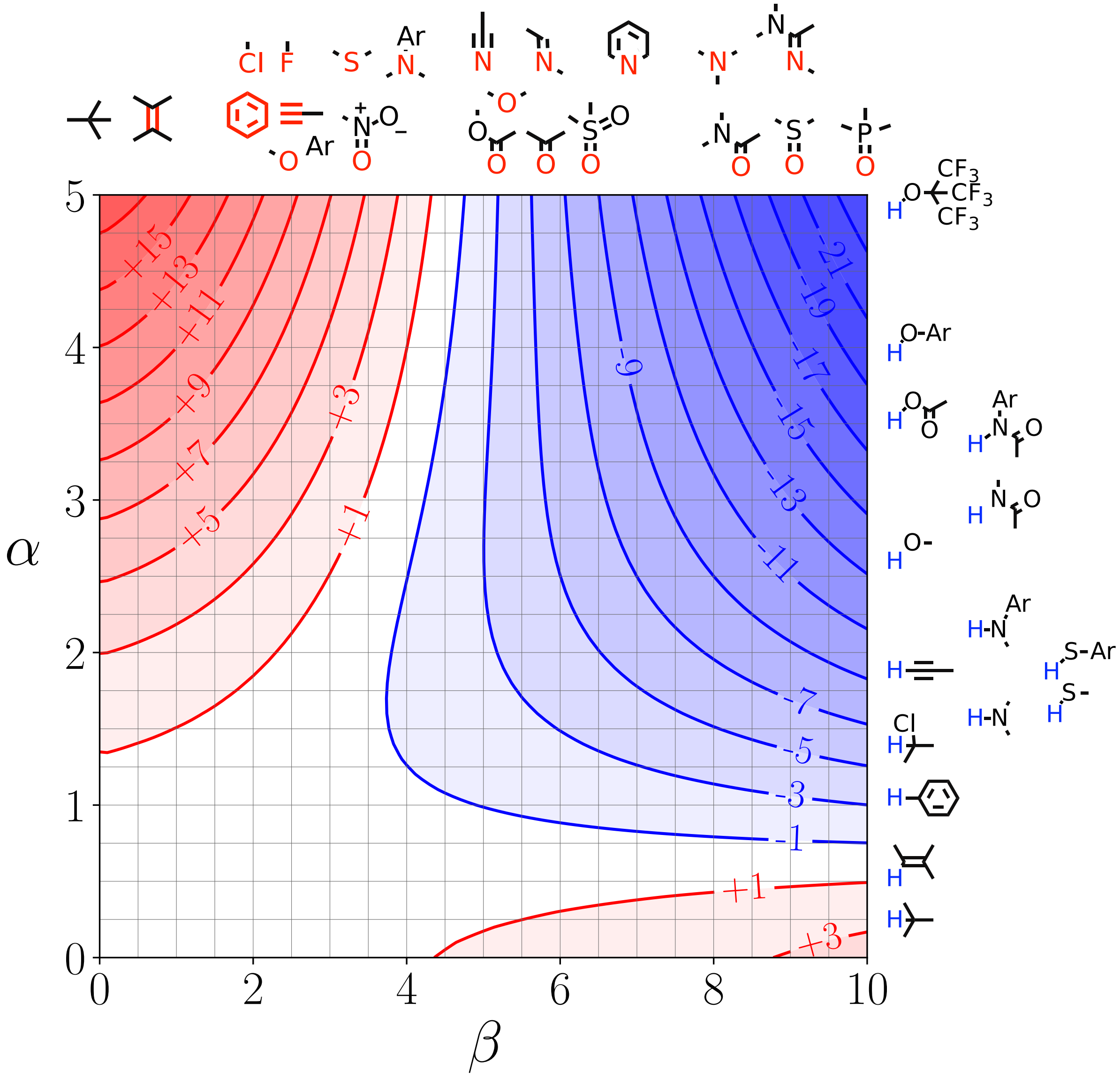

Figure S134: FGIP for methyl propionate at 298K.

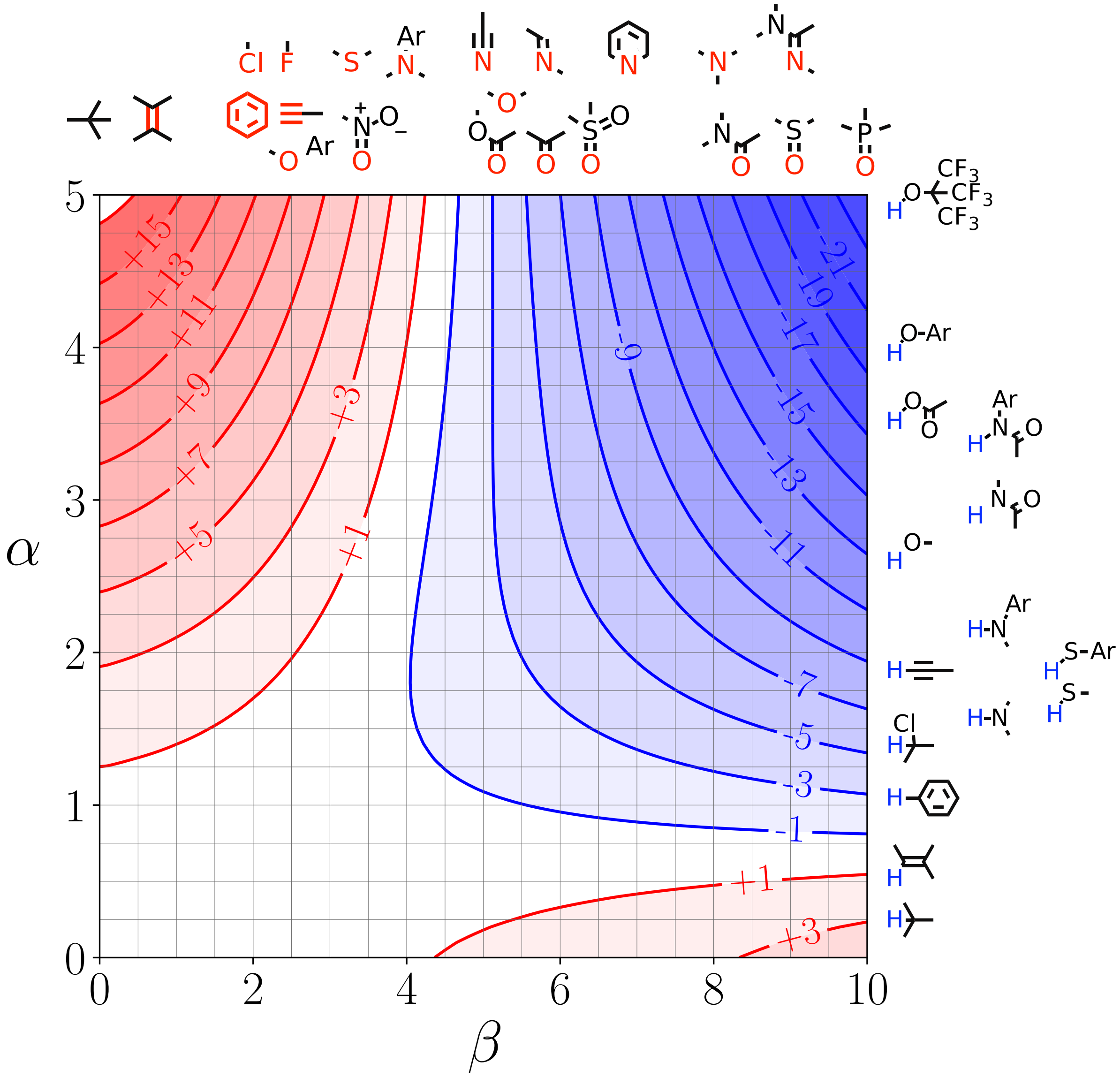

Figure S135: FGIP for ethyl propionate at 298K.

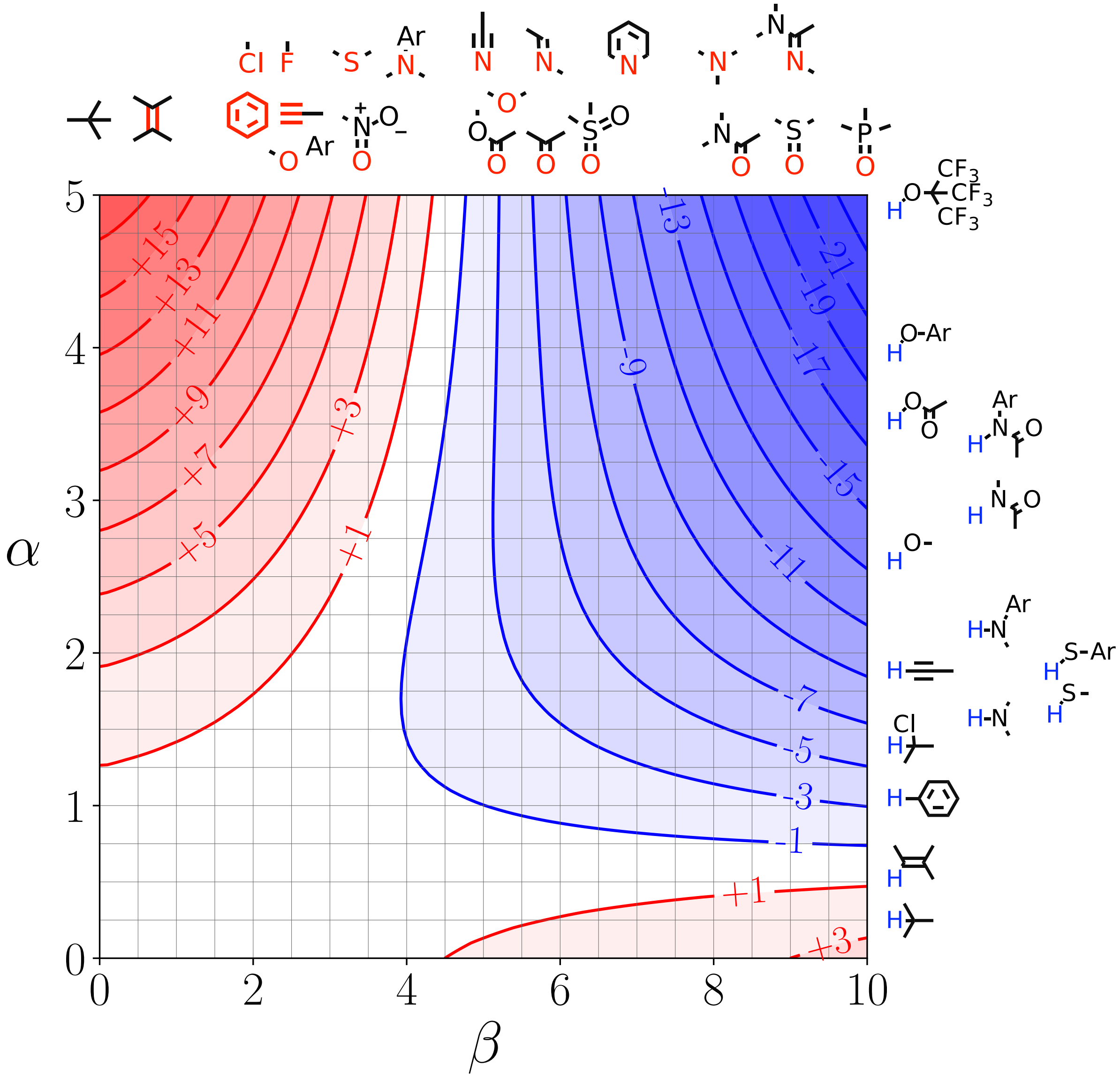

Figure S136: FGIP for dimethyl carbonate at 298K.

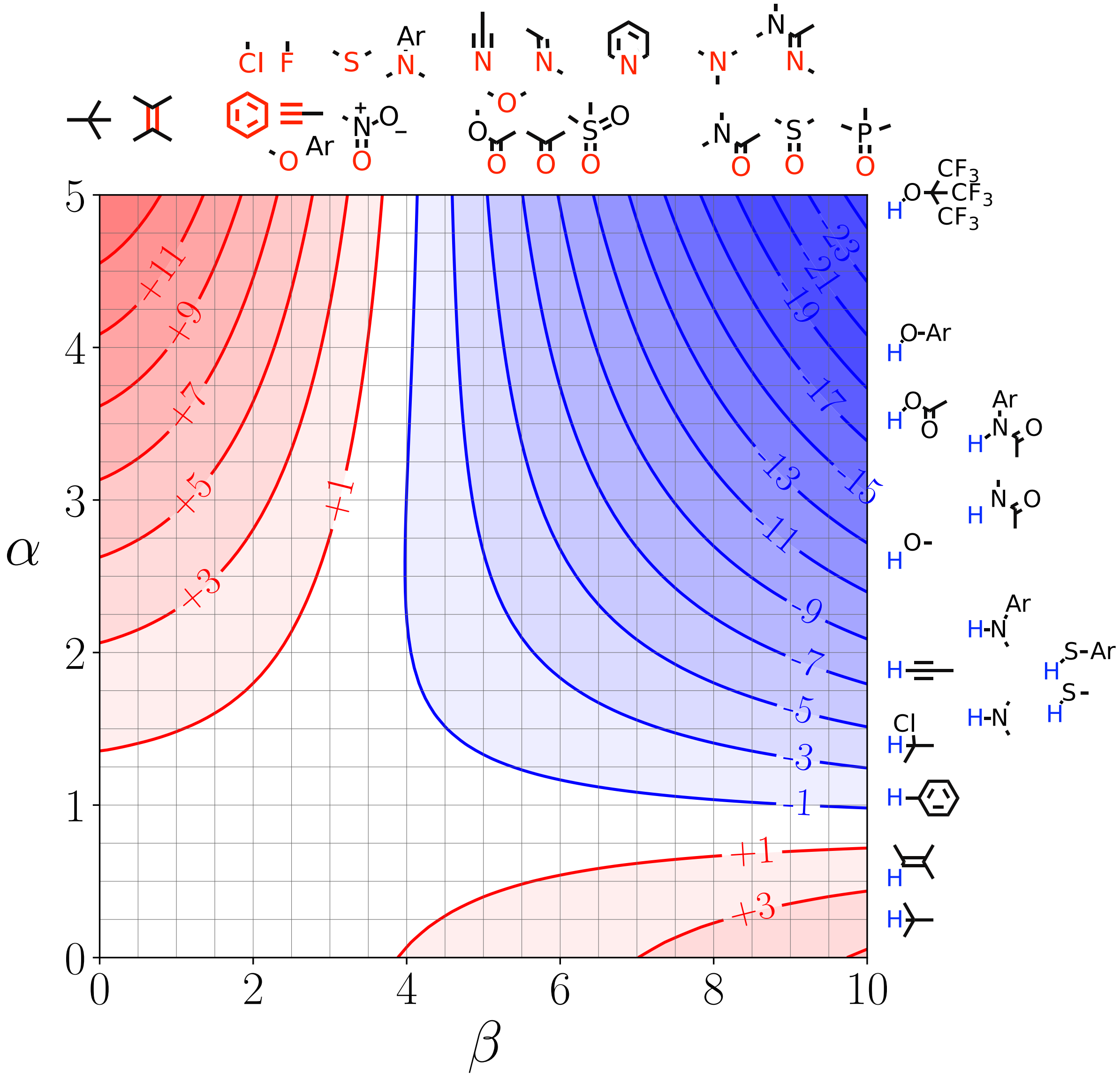

Figure S137: FGIP for diethyl carbonate at 298K.

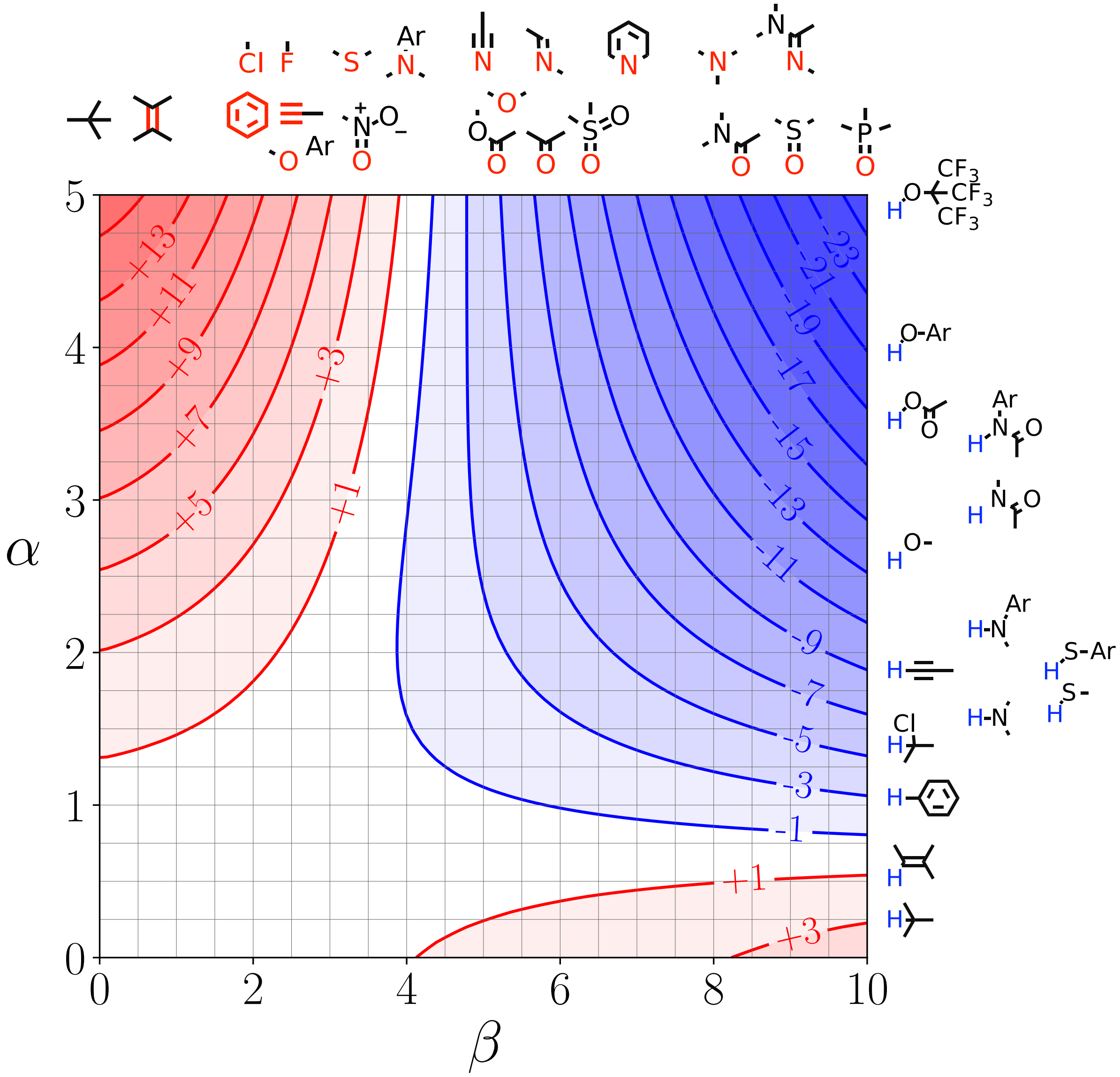

Figure S138: FGIP for ethylene carbonate at 298K.

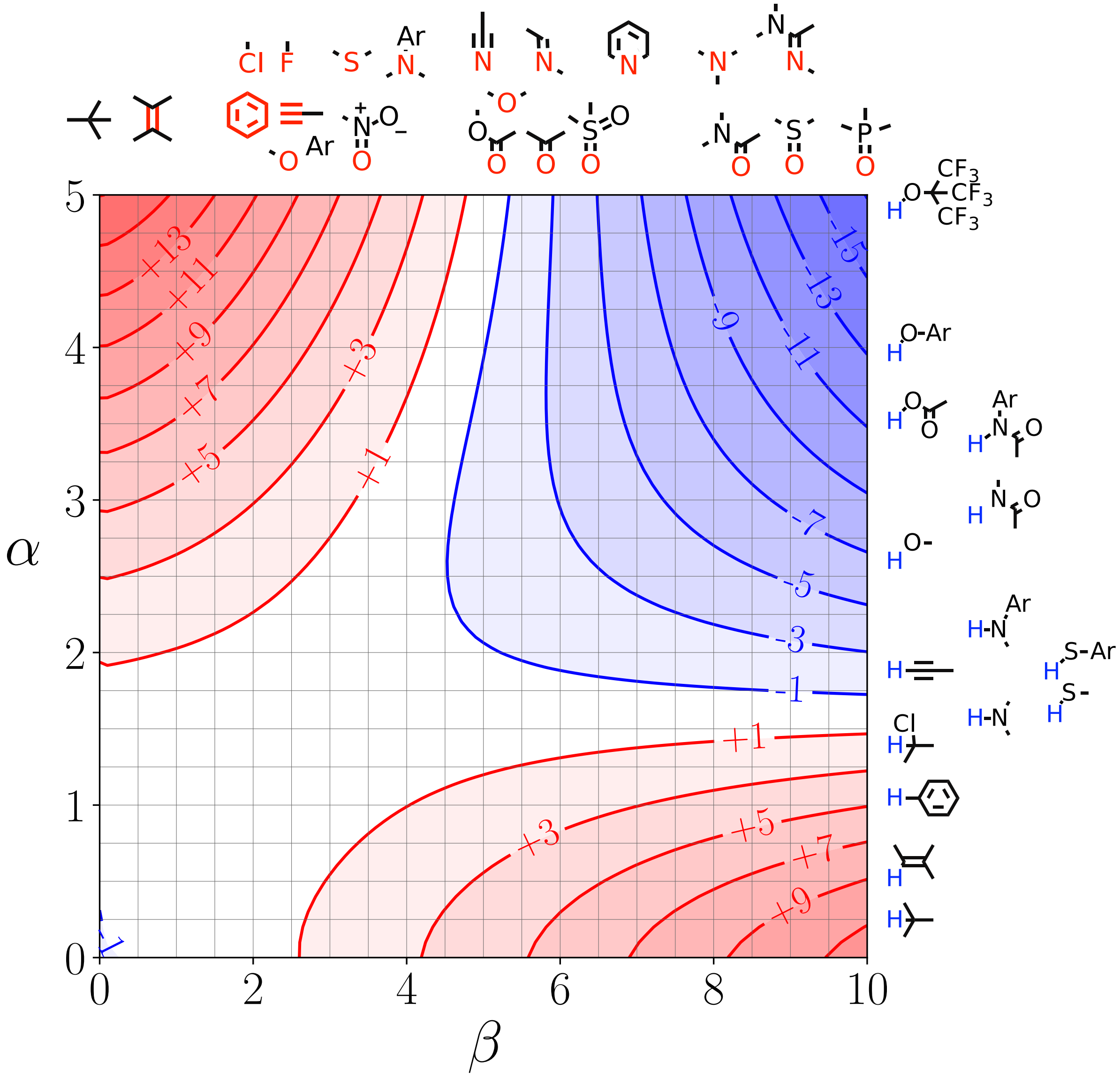

Figure S139: FGIP for 4-methyl-1,3-dioxolan-2-one at 298K.

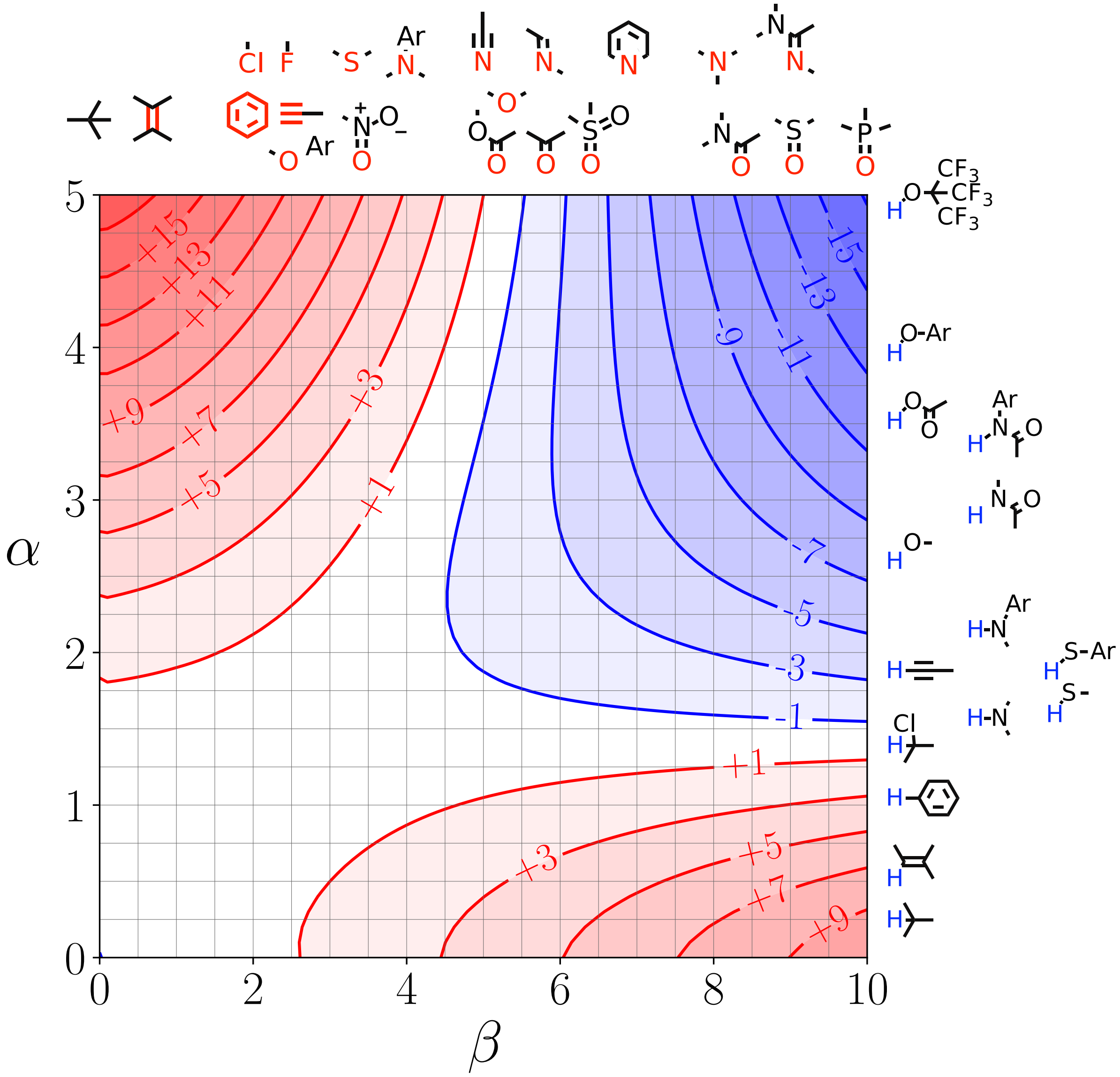

Figure S140: FGIP for diethyl malonate at 298K.

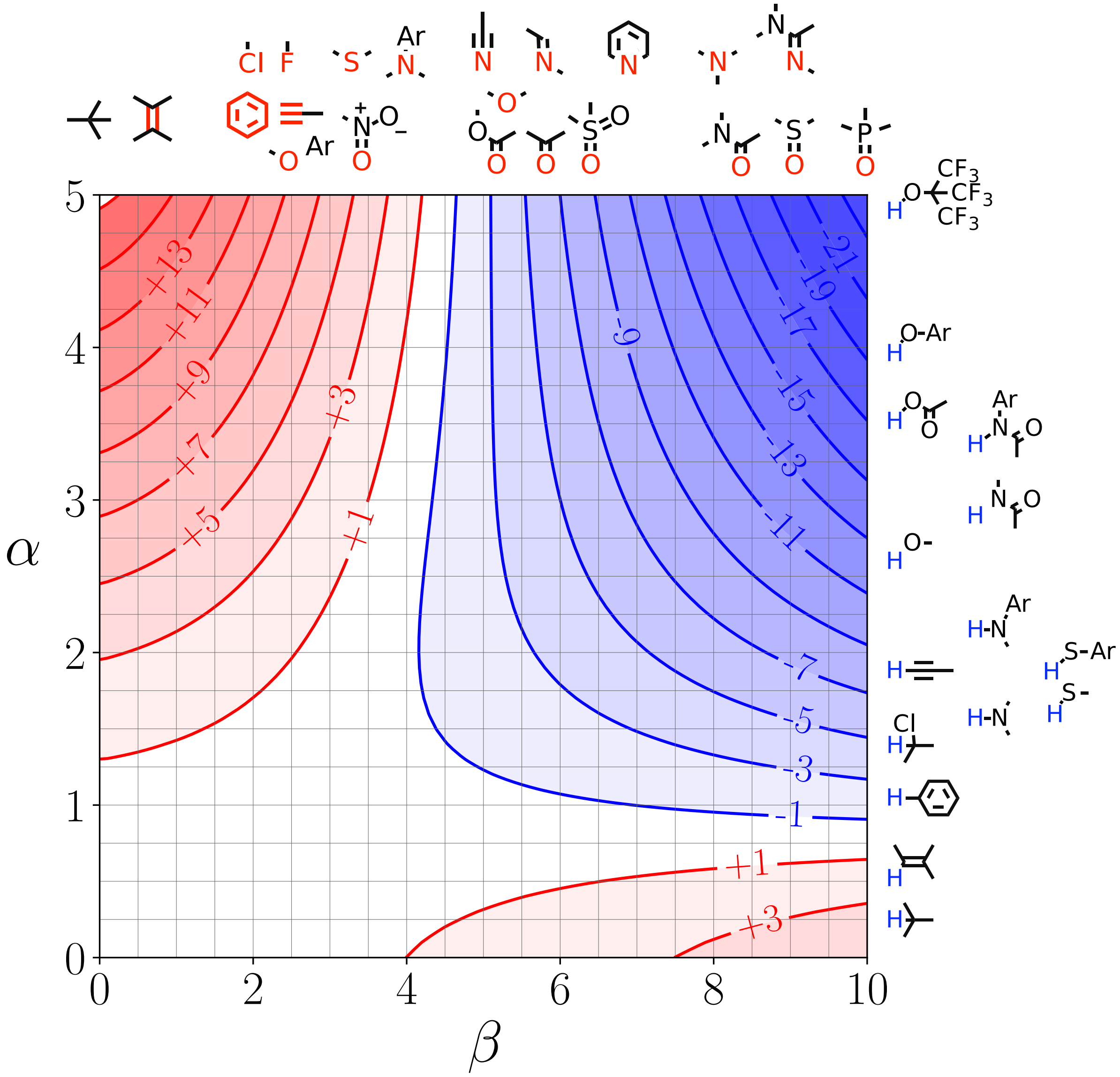

Figure S141: FGIP for methyl benzoate at 298K.

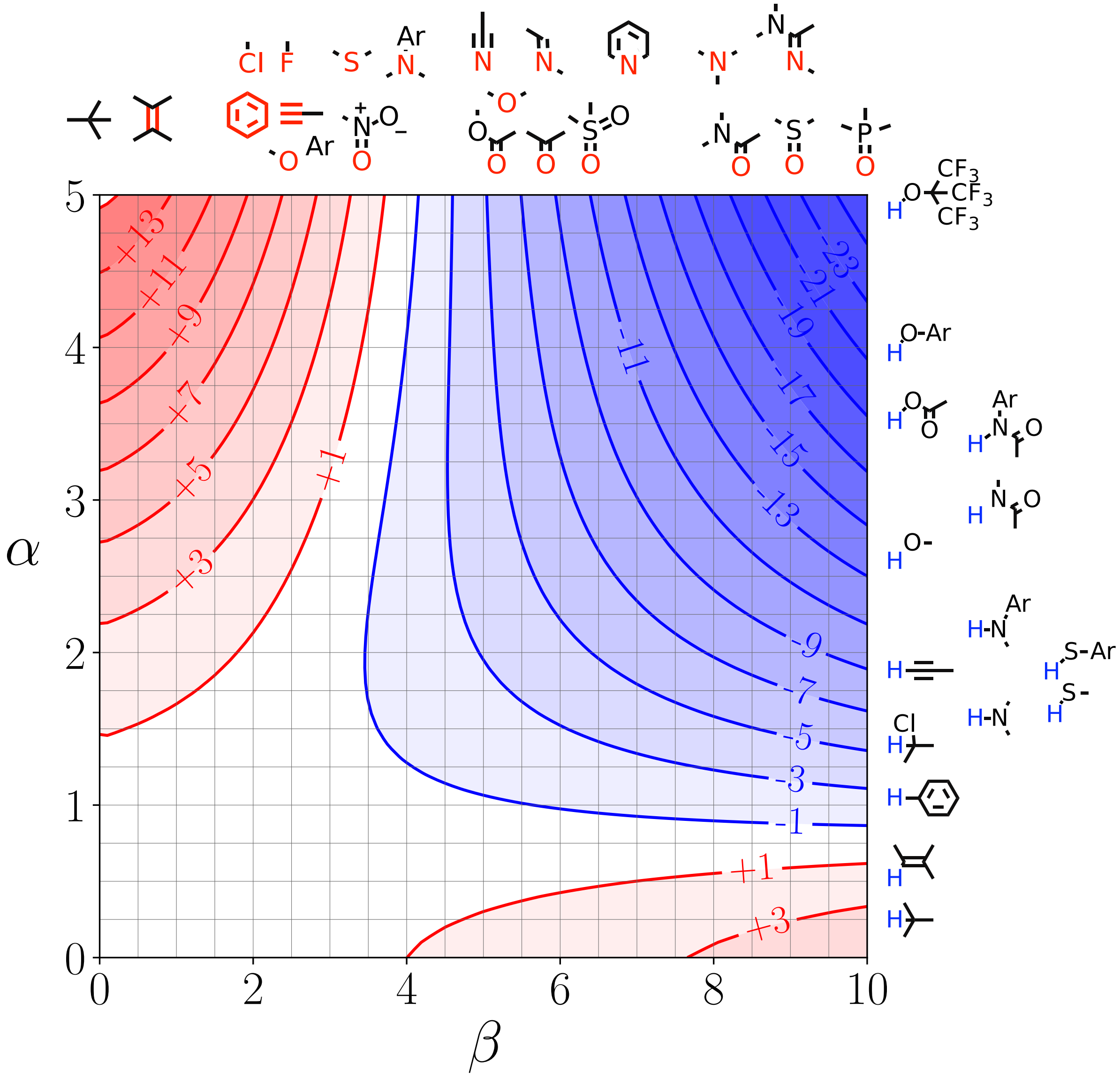

Figure S142: FGIP for ethyl benzoate at 298K.

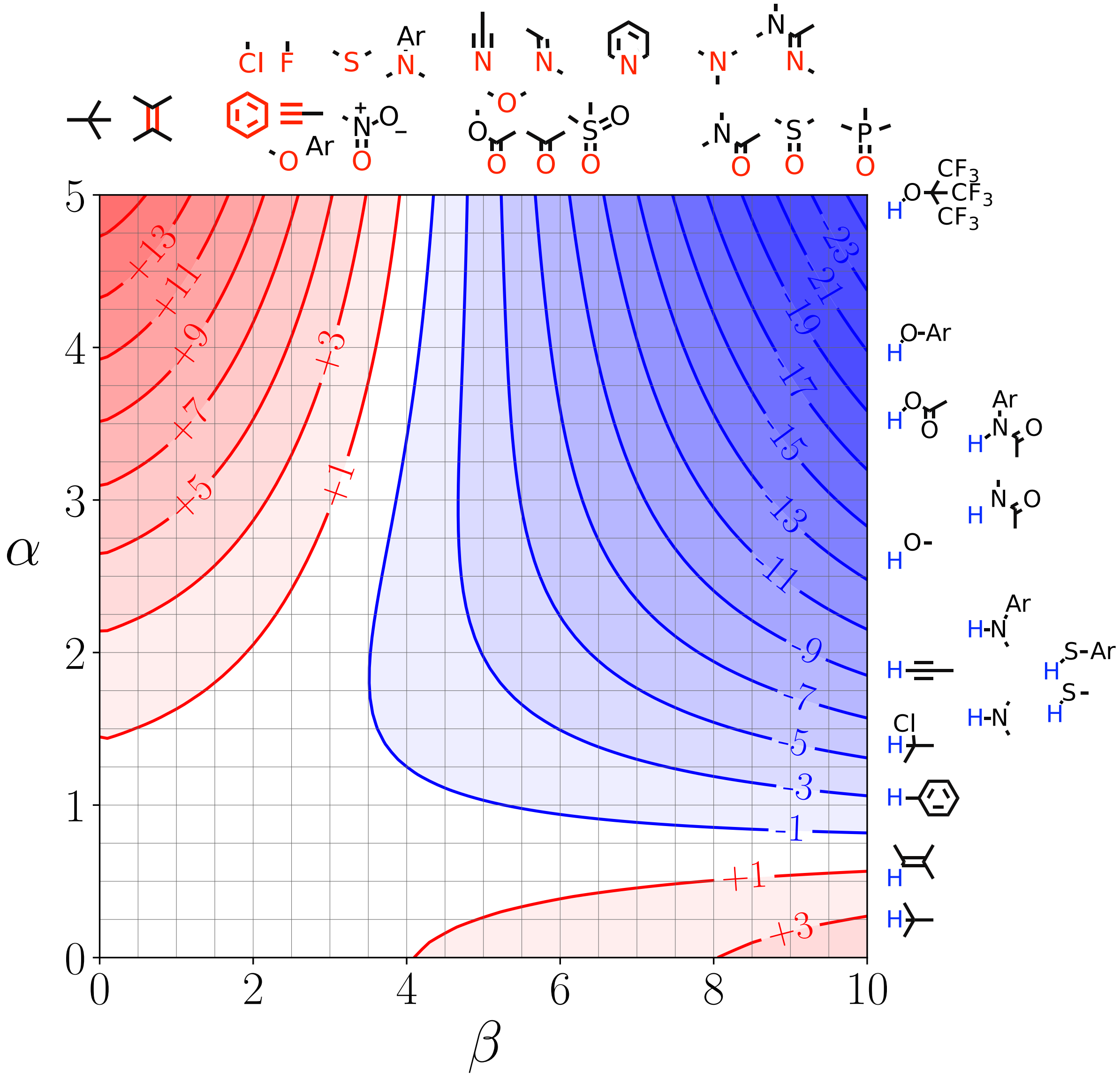

Figure S143: FGIP for dimethylphthalate at 298K.

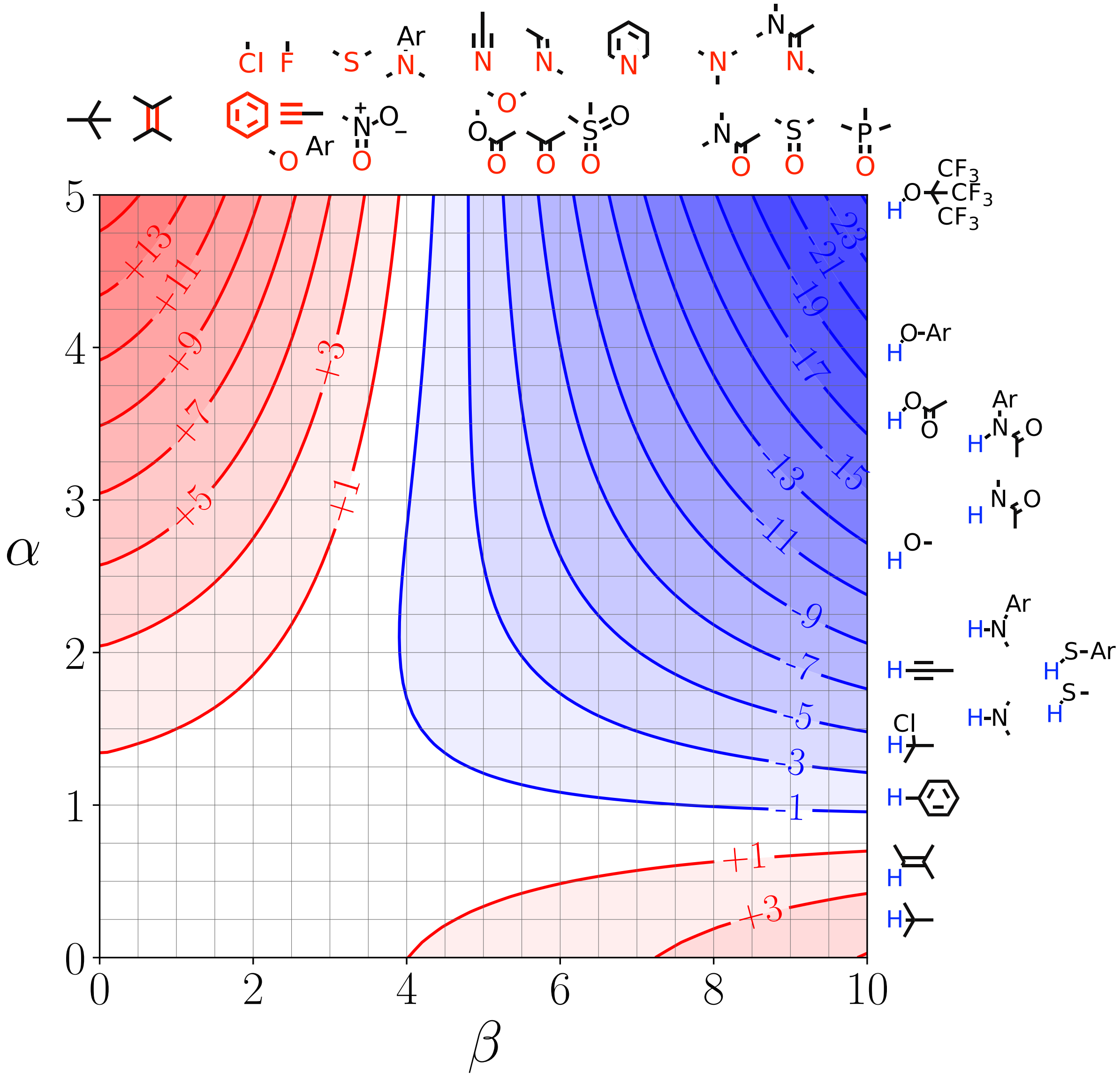

Figure S144: FGIP for di-n-butylorthophthalate at 298K.

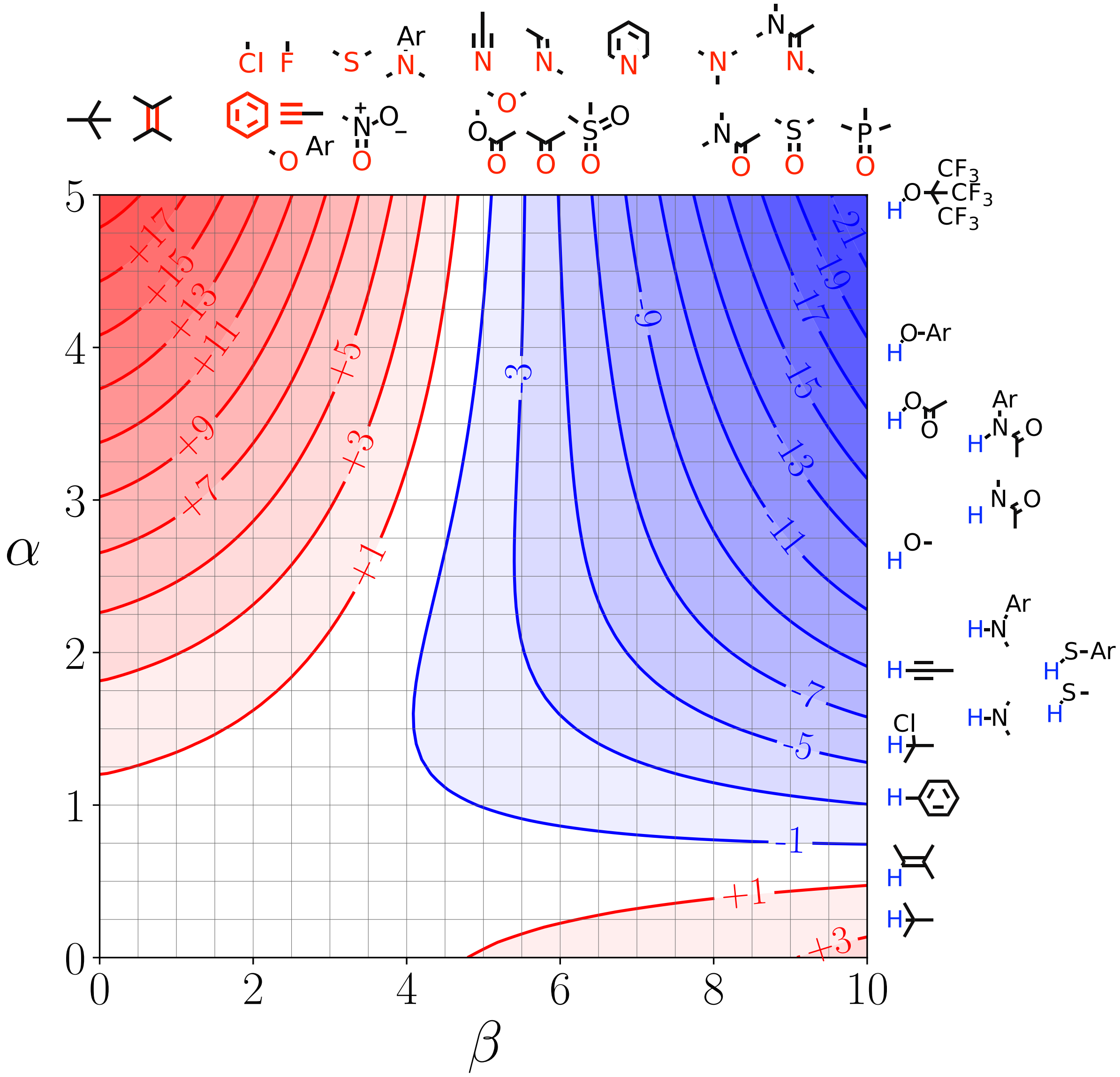

Figure S145: FGIP for ethyl chloroacetate at 298K.

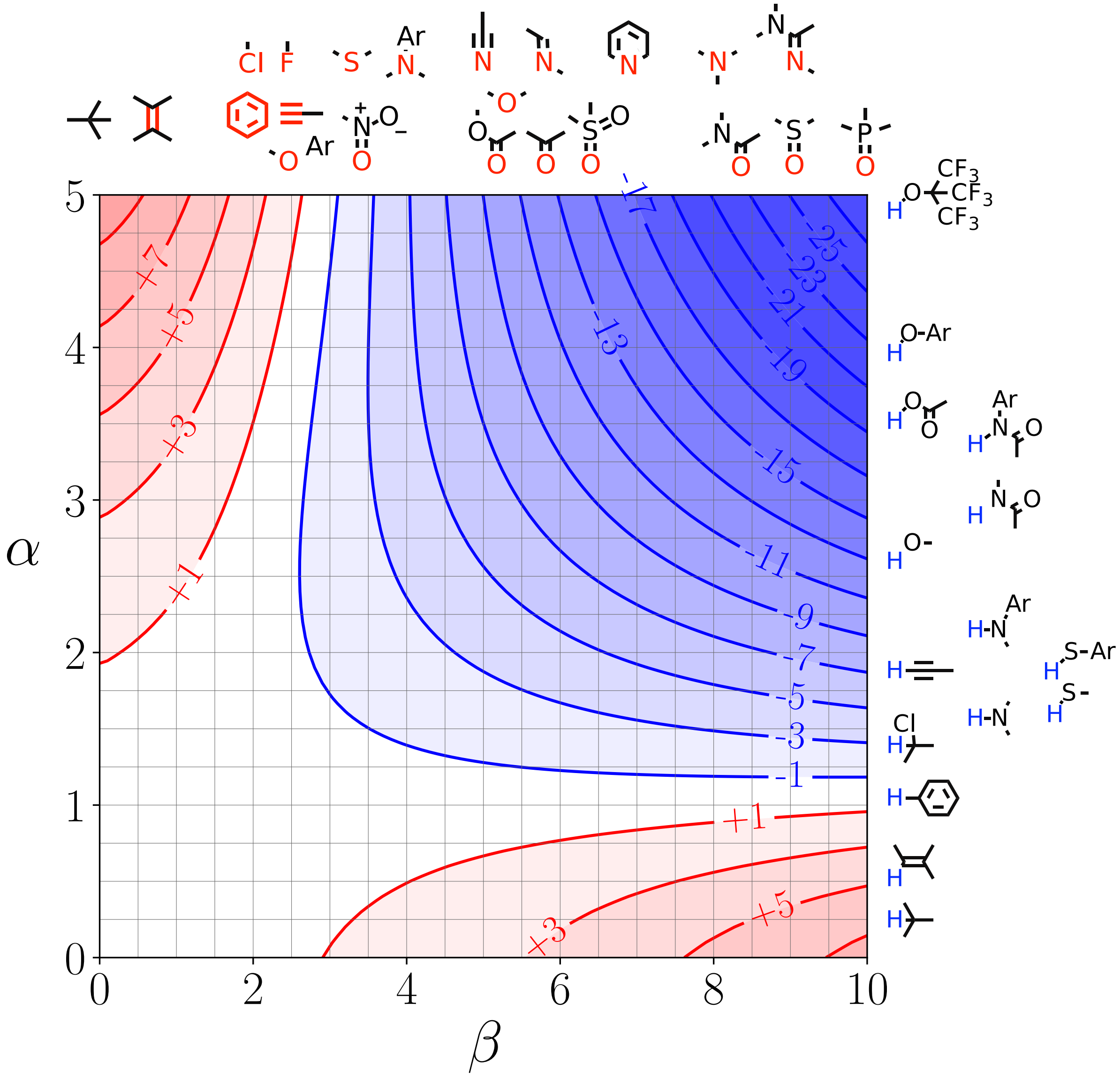

Figure S146: FGIP for ethyl trichloroacetate at 298K.

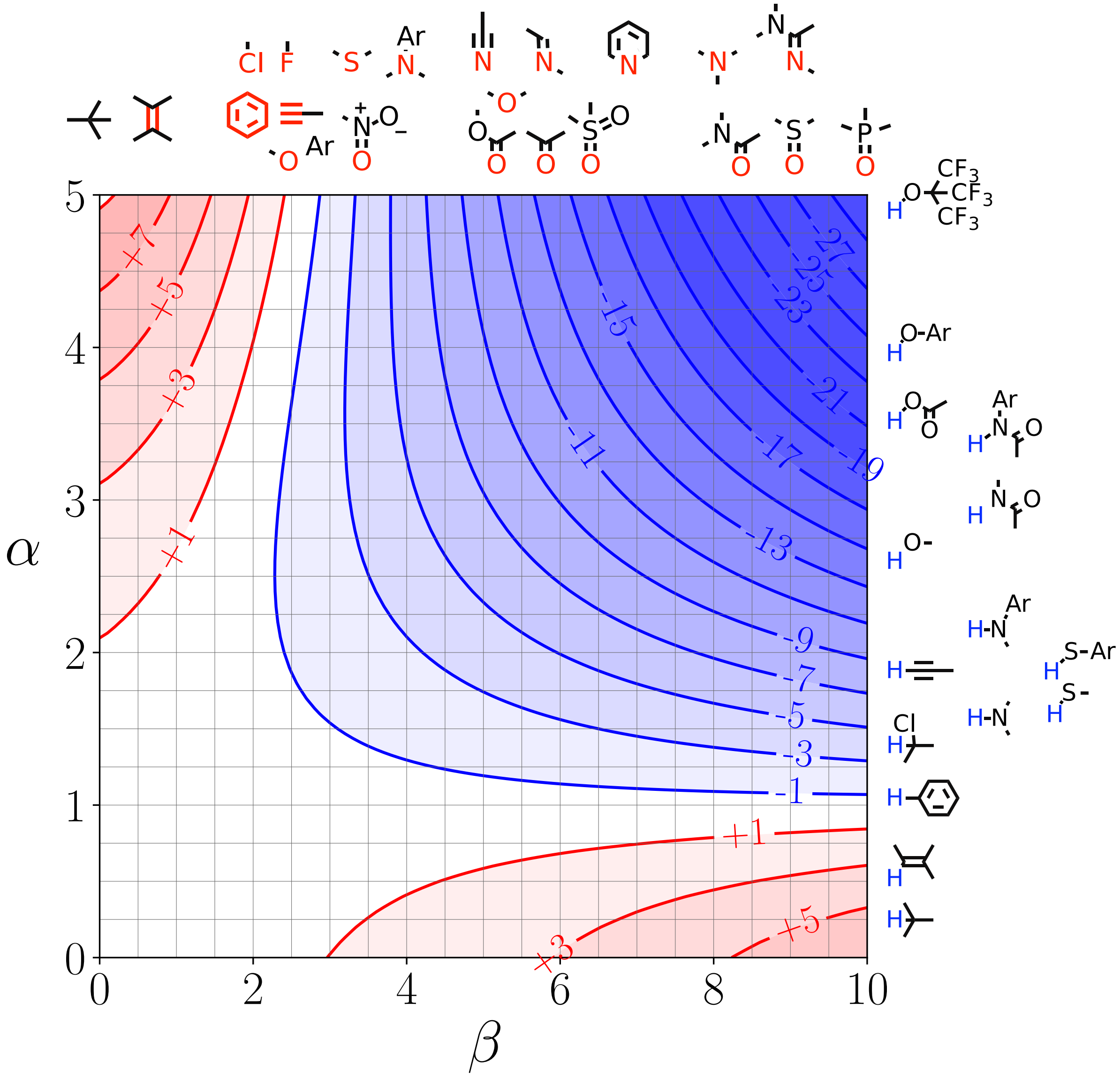

Figure S147: FGIP for ethyl acetoacetate at 298K.

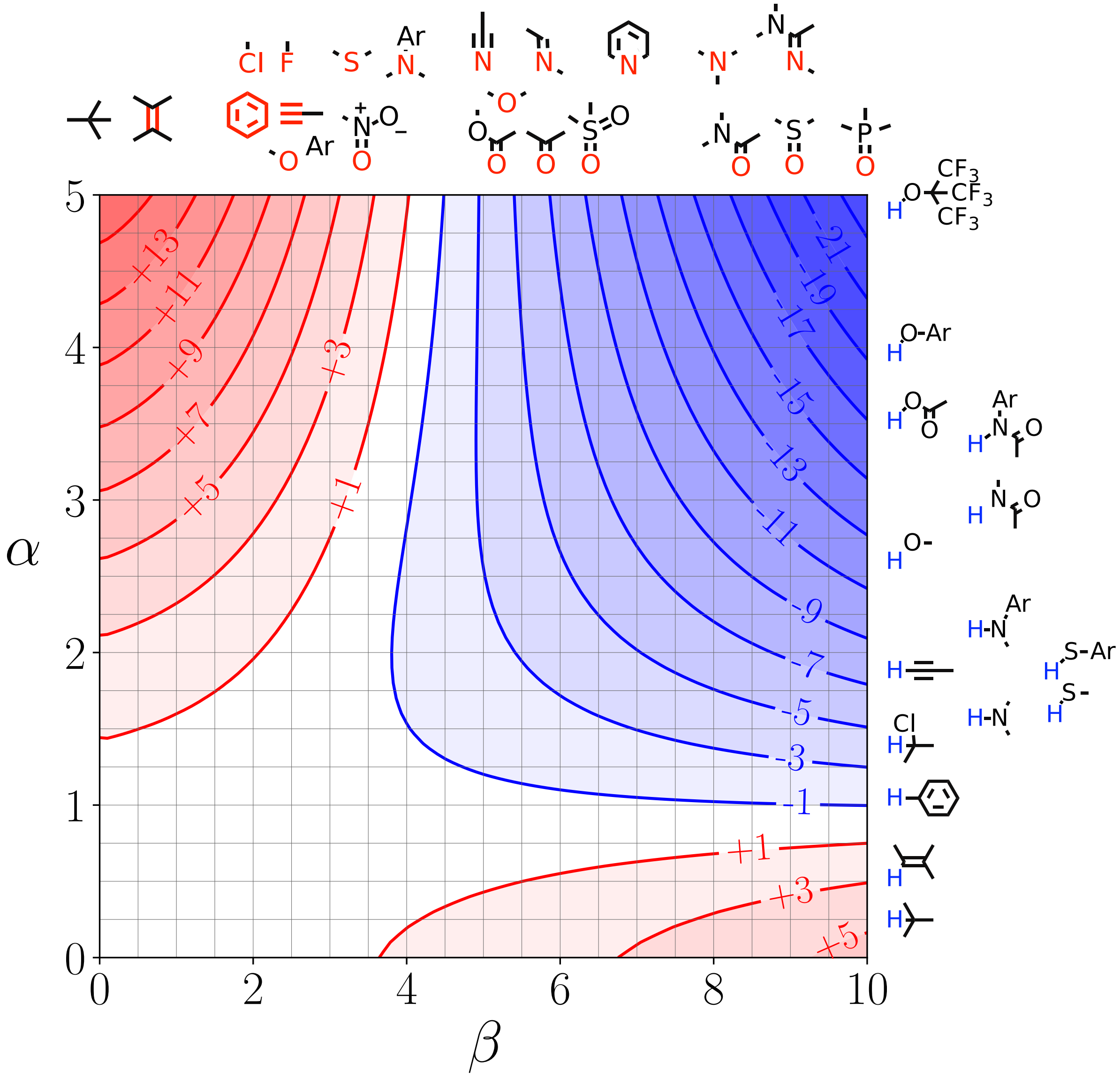

Figure S148: FGIP for gamma-butyrolactone at 298K.

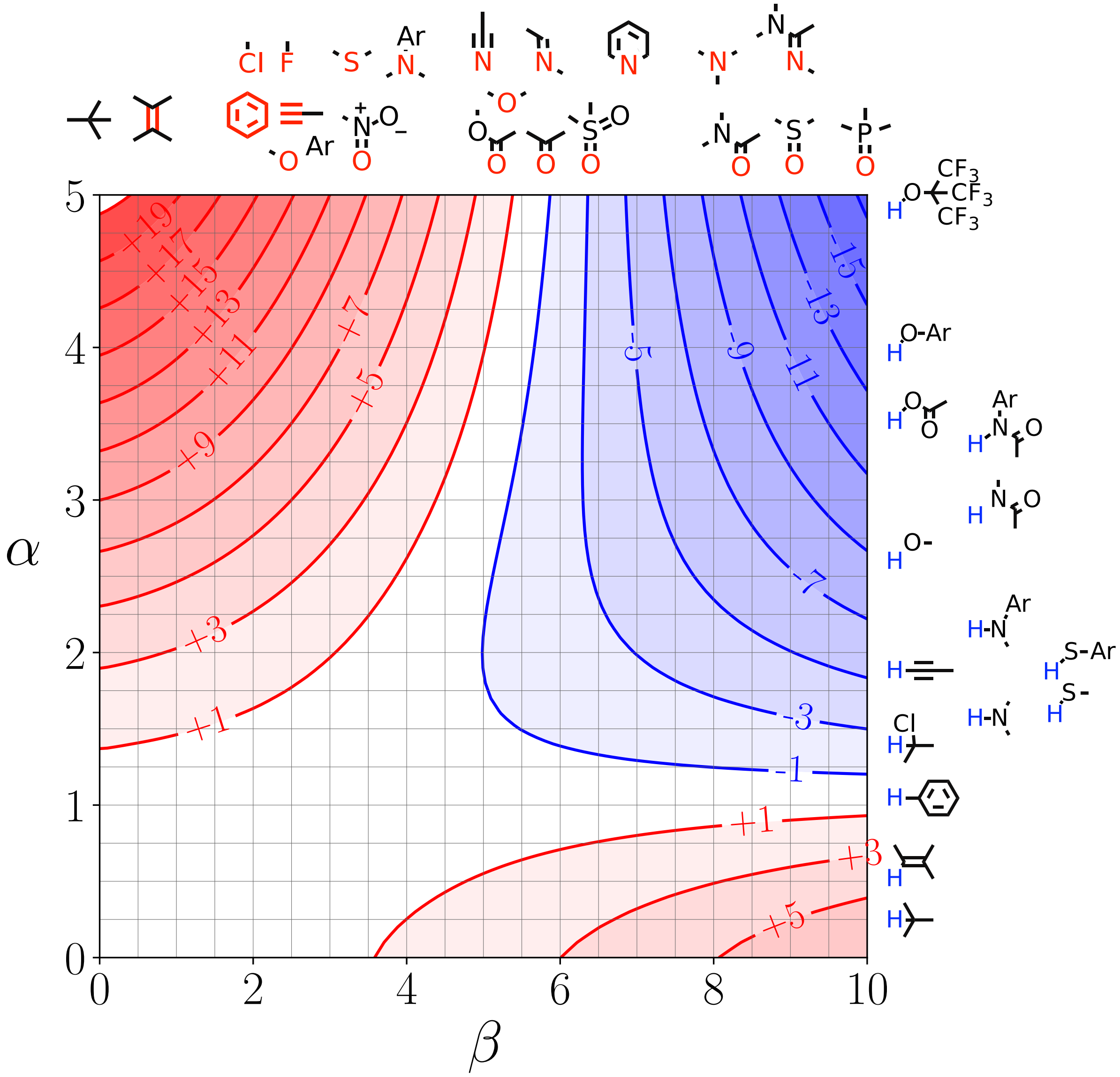

Figure S149: FGIP for n-perfluorohexane at 298K.

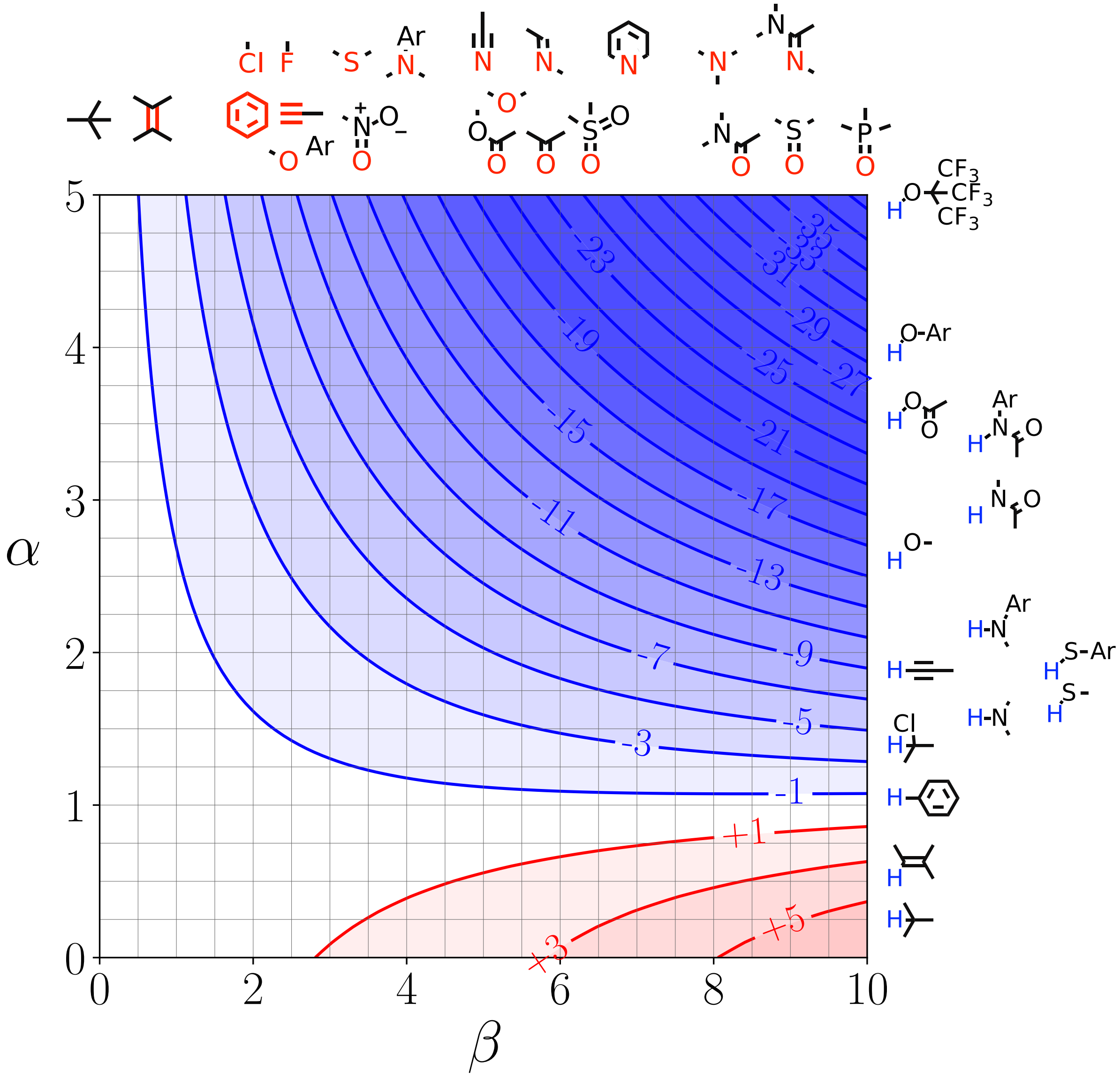

Figure S150: FGIP for perfluoromethylcyclohexane at 298K.

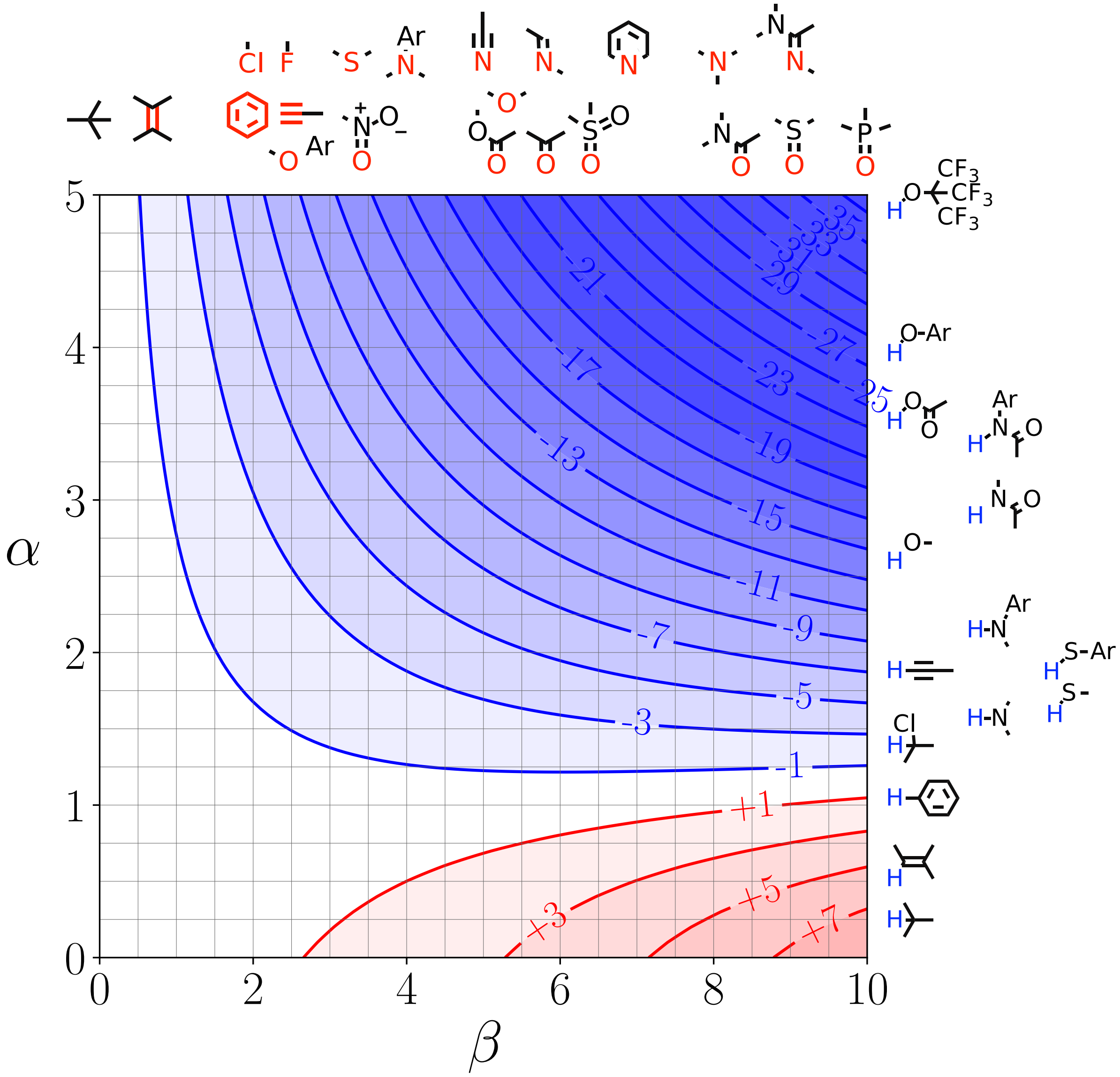

Figure S151: FGIP for perfluoroheptane at 298K.

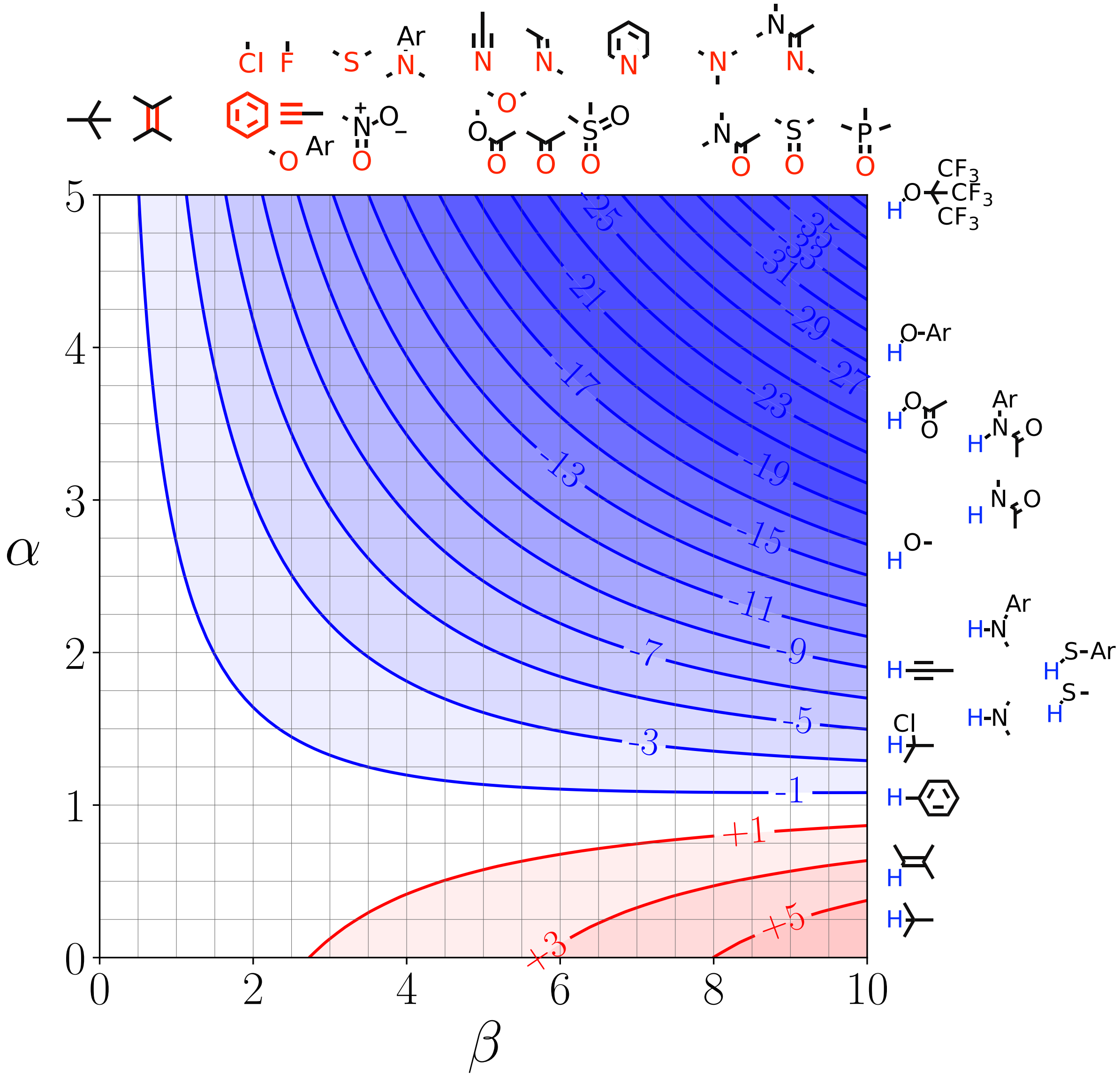

Figure S152: FGIP for cis-perfluorodecalin at 298K.

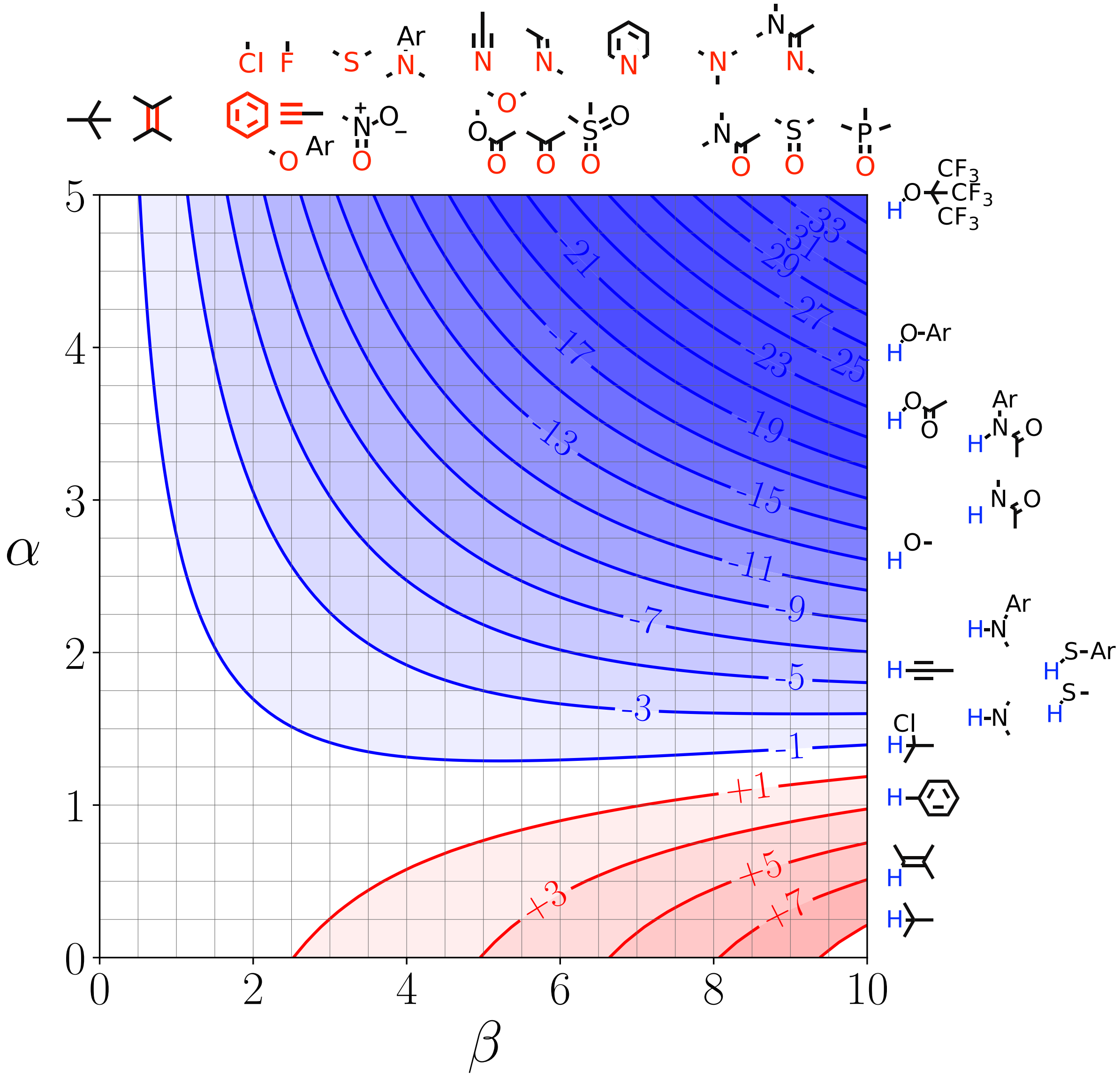

Figure S153: FGIP for fluorobenzene at 298K.

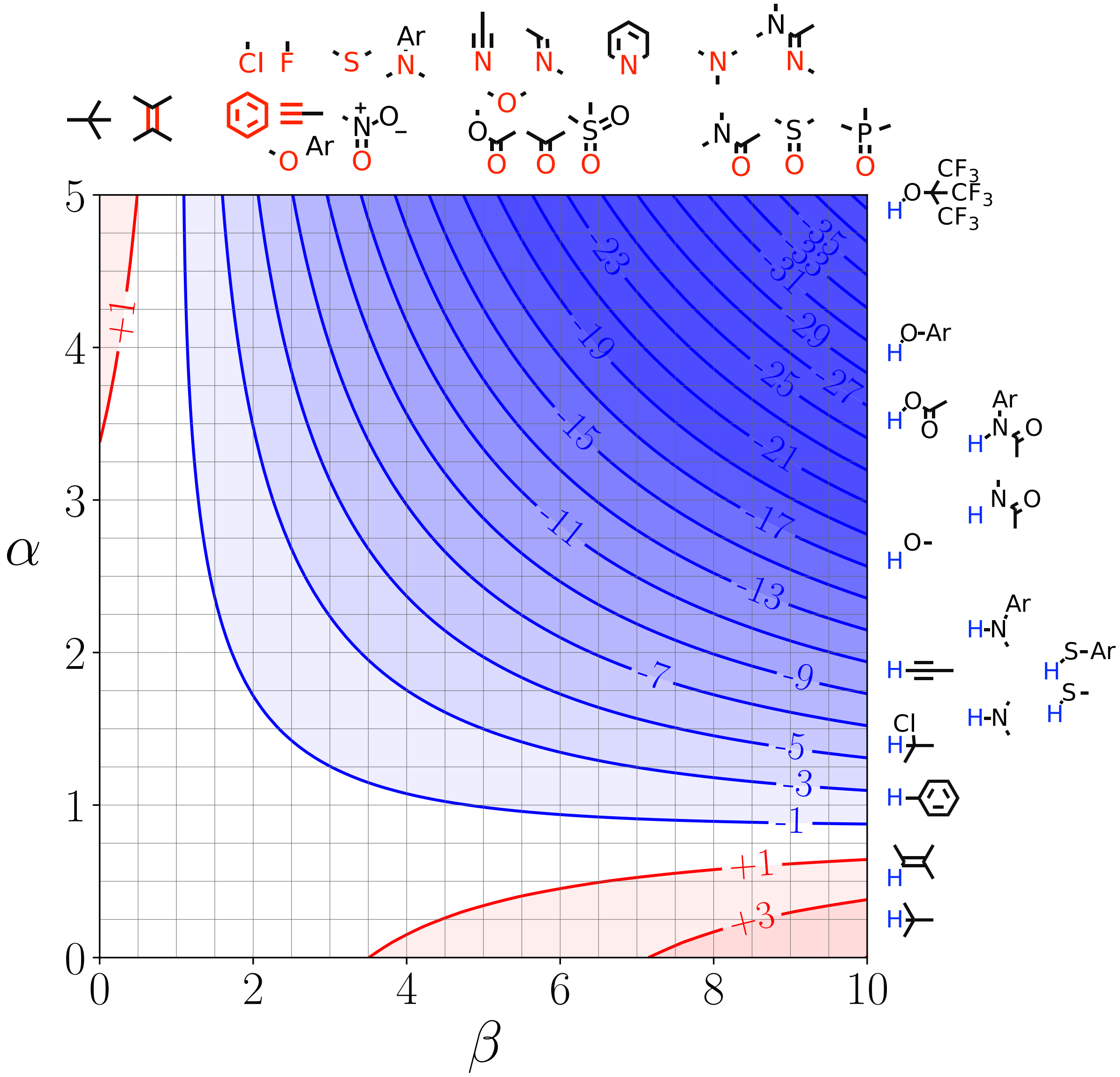

Figure S154: FGIP for hexafluorobenzene at 298K.

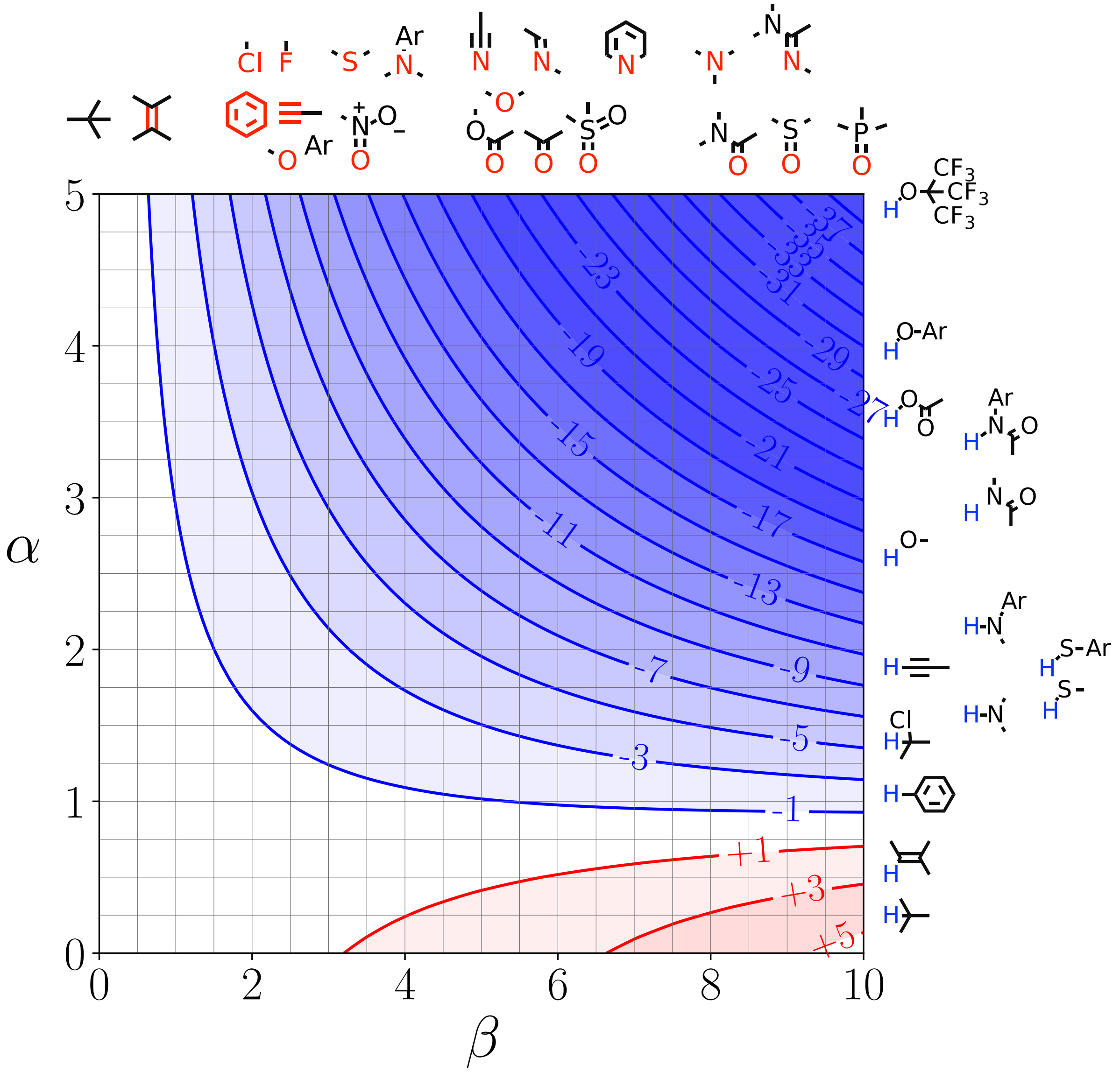

Figure S155: FGIP for 1,4-dichlorobutane at 298K.

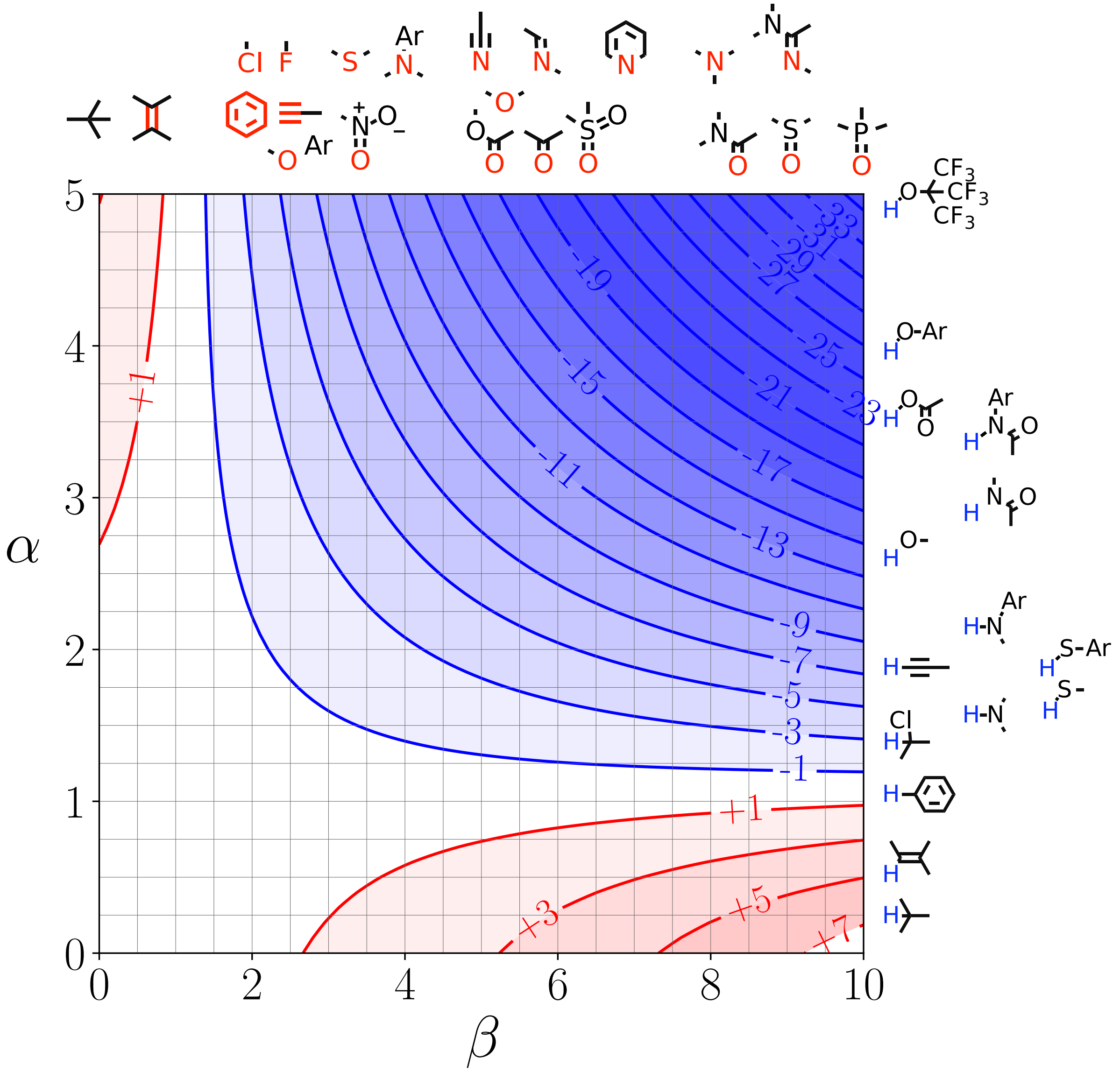

Figure S156: FGIP for chlorobenzene at 298K.

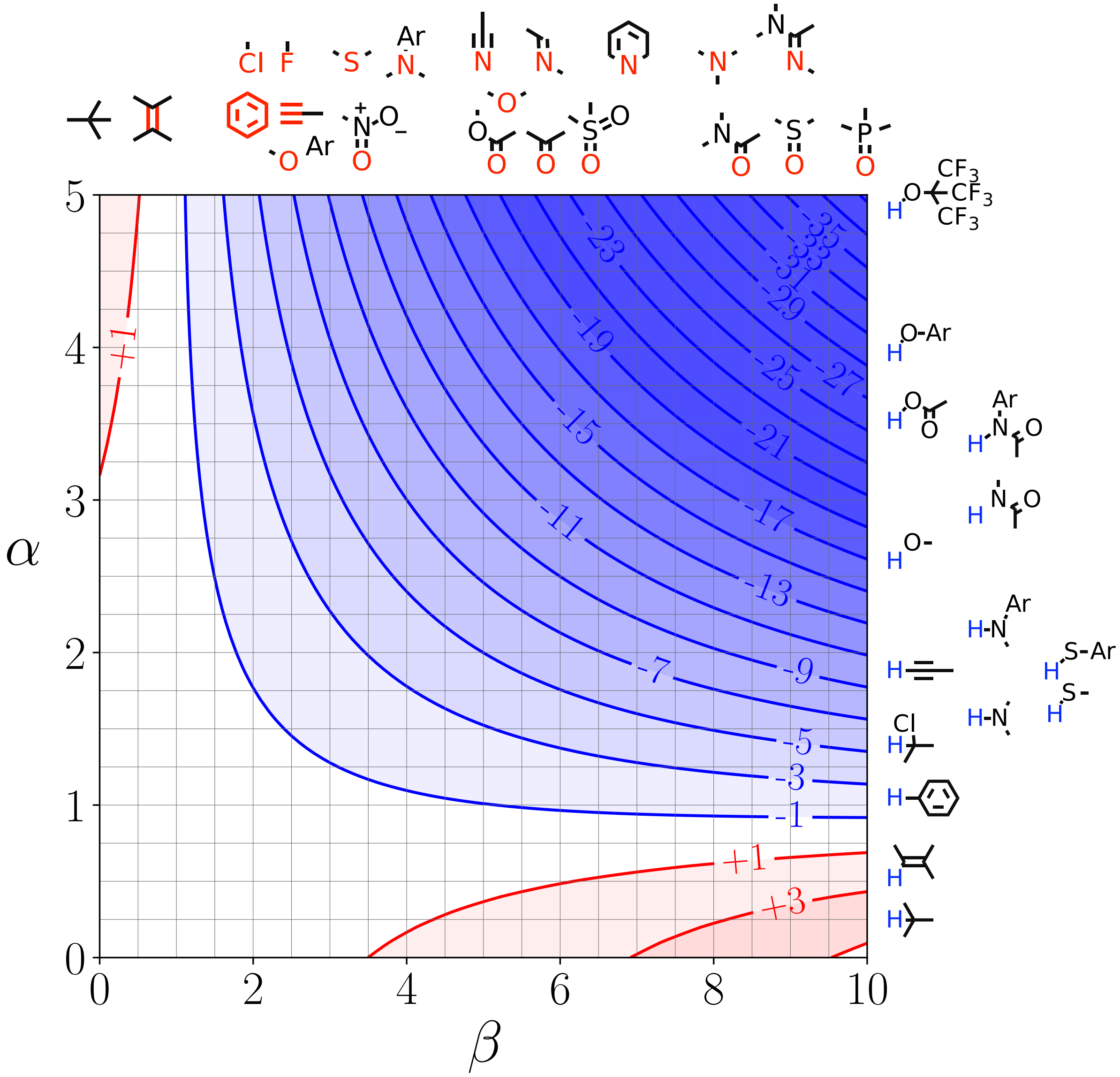

Figure S157: FGIP for dichloromethane at 298K.

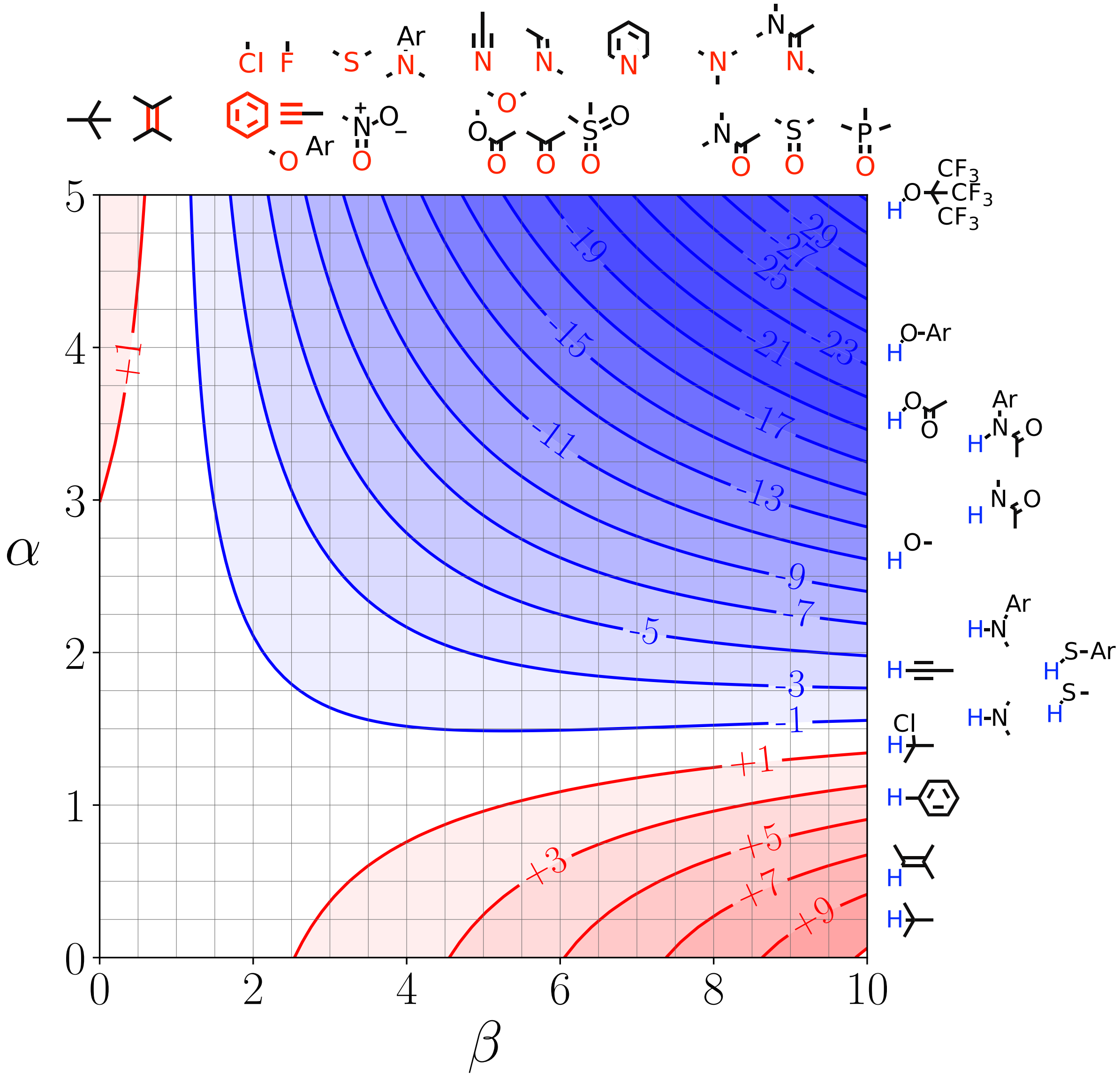

Figure S158: FGIP for 1,1-dichloroethane at 298K.

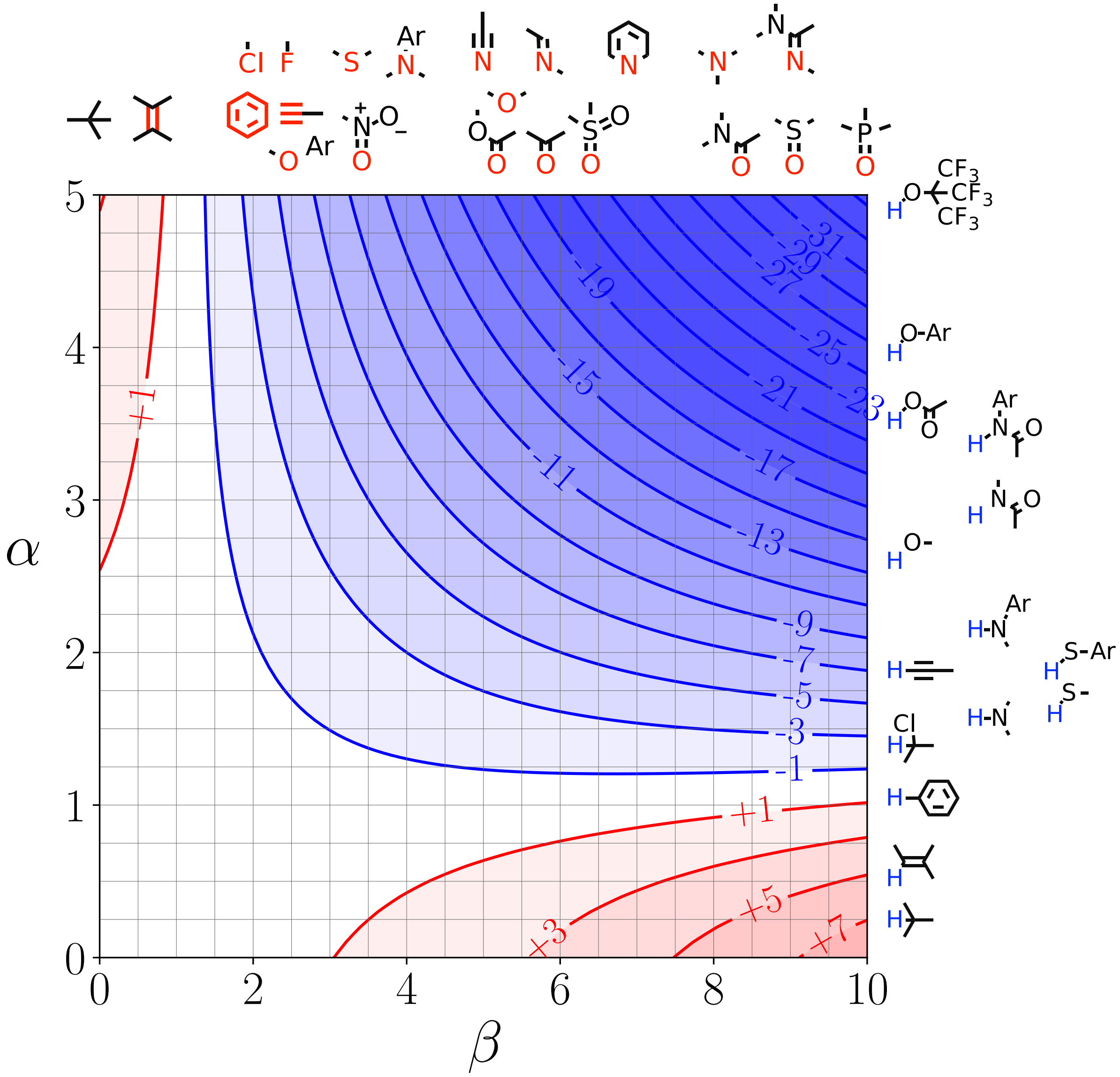

Figure S159: FGIP for 1,2-dichloroethane at 298K.

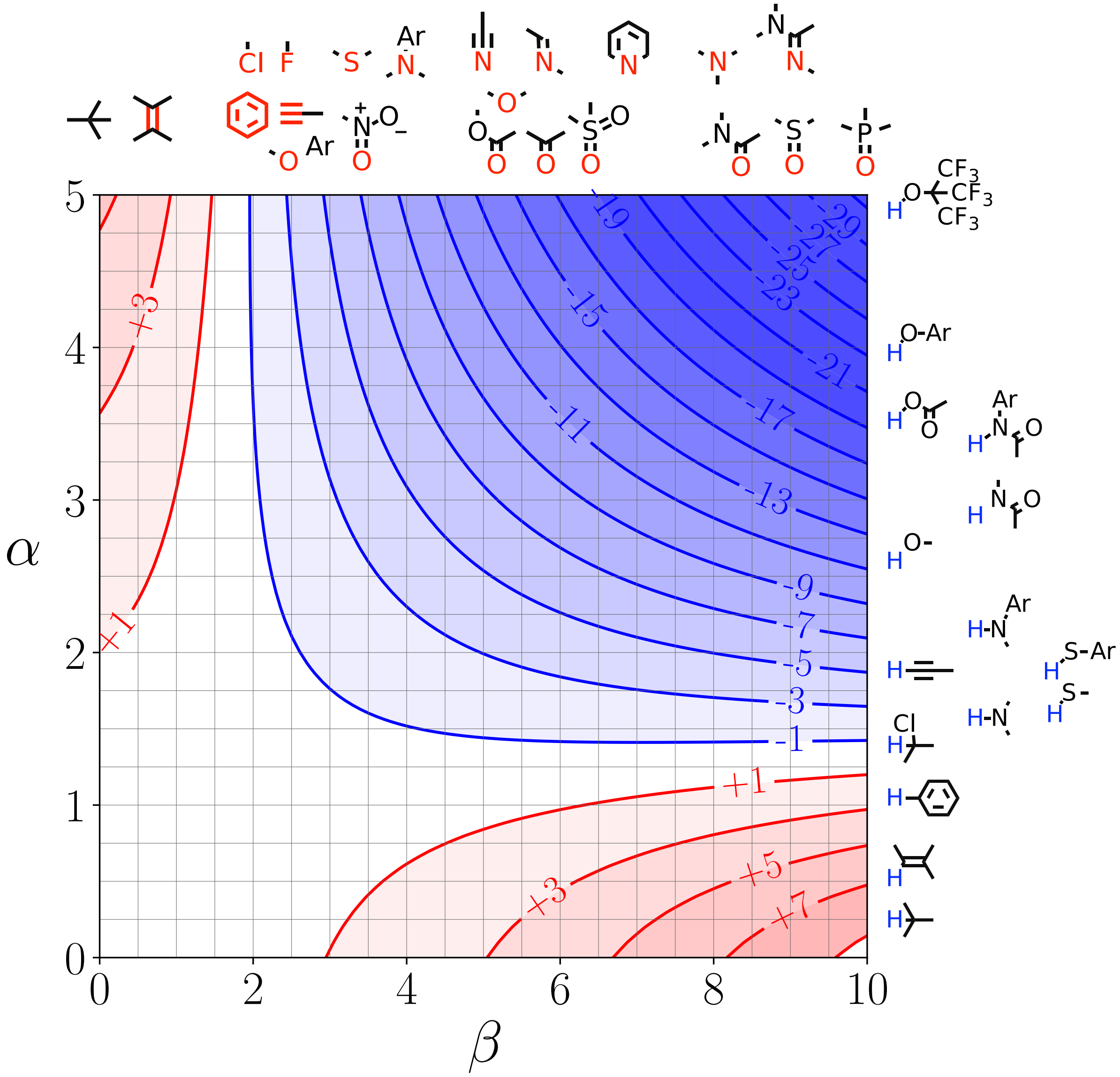

Figure S160: FGIP for trans-1,2-dichloroethylene at 298K.

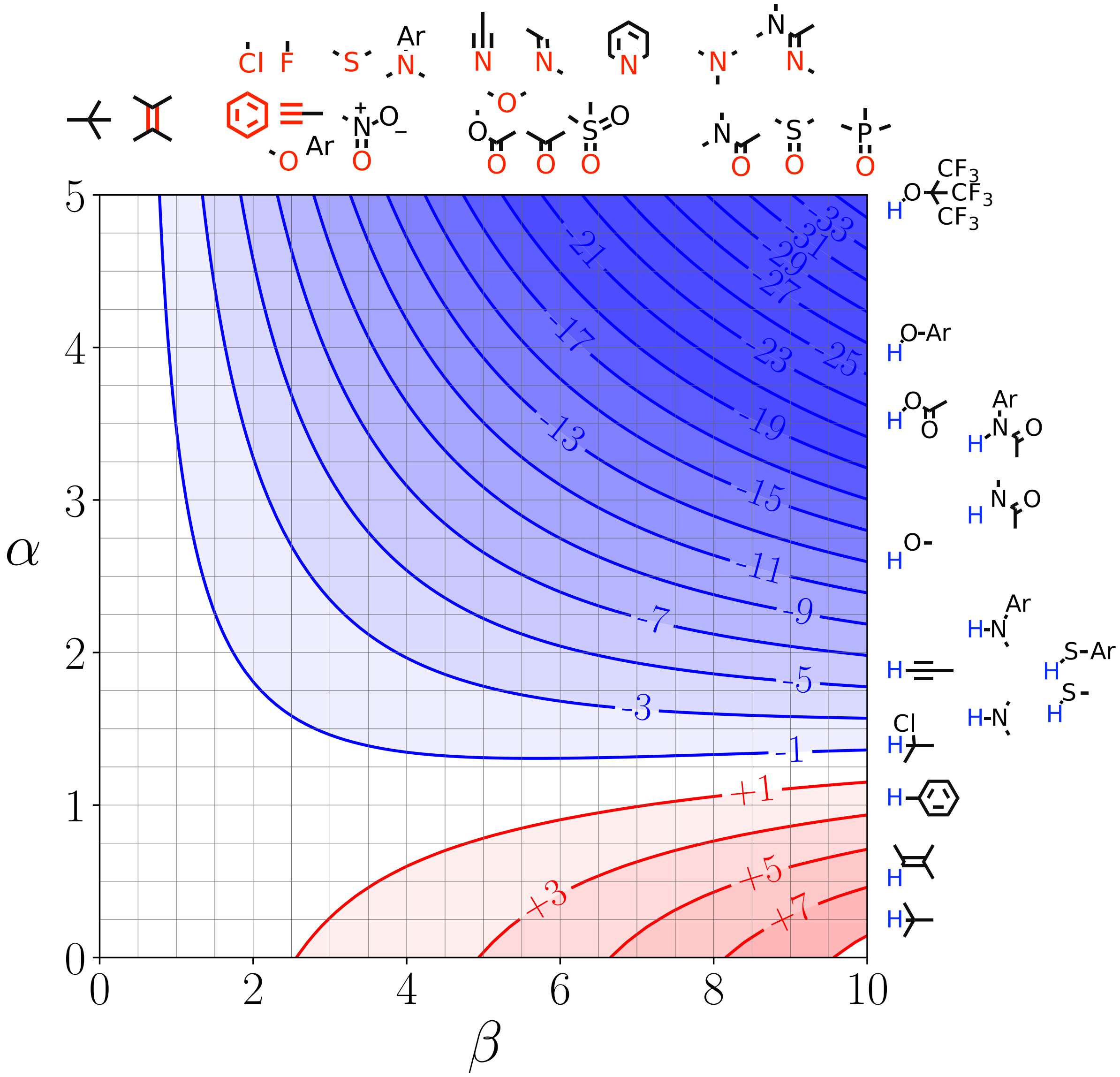

Figure S161: FGIP for ortho-dichlorobenzene at 298K.

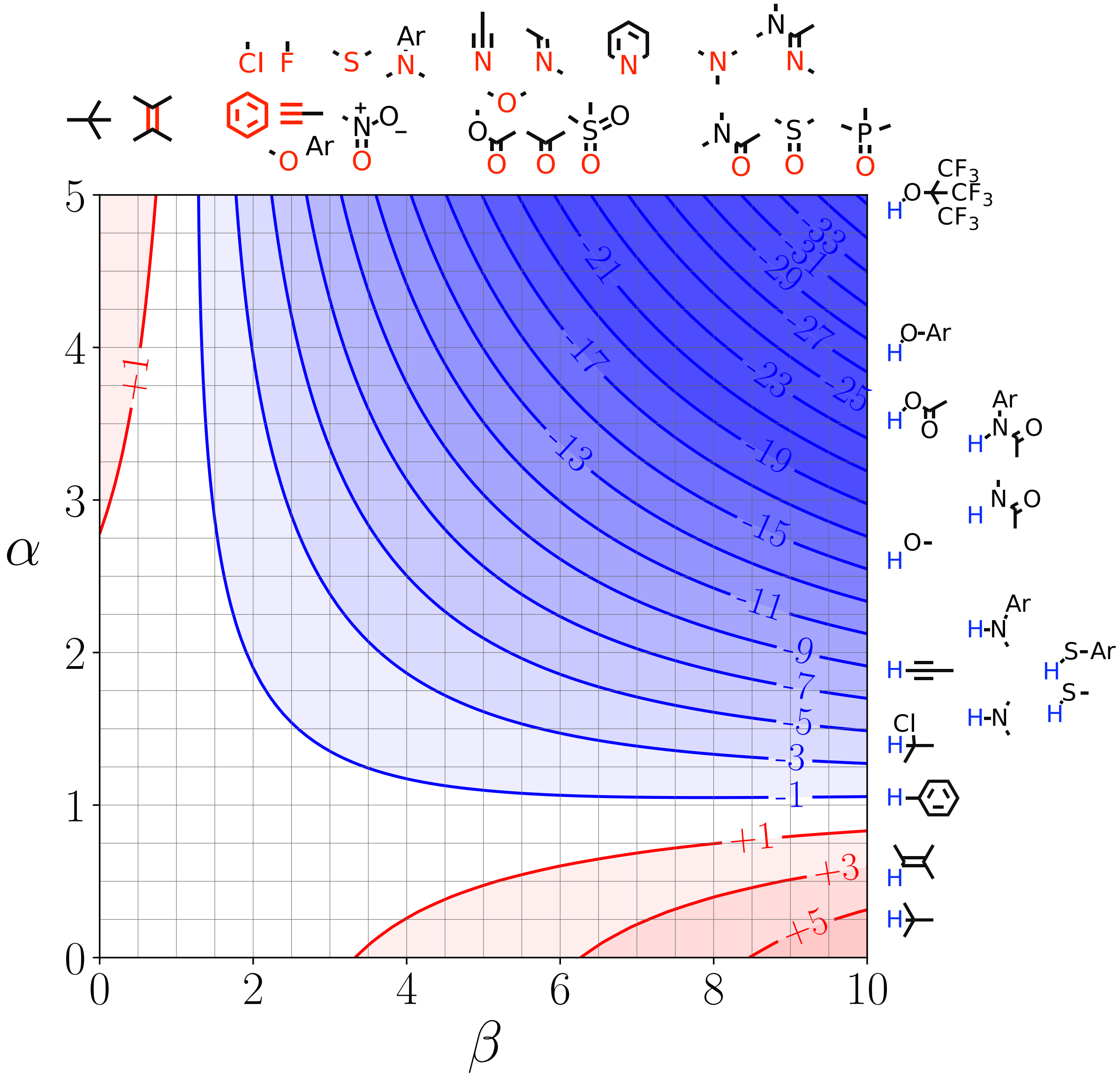

Figure S162: FGIP for meta-dichlorobenzene at 298K.

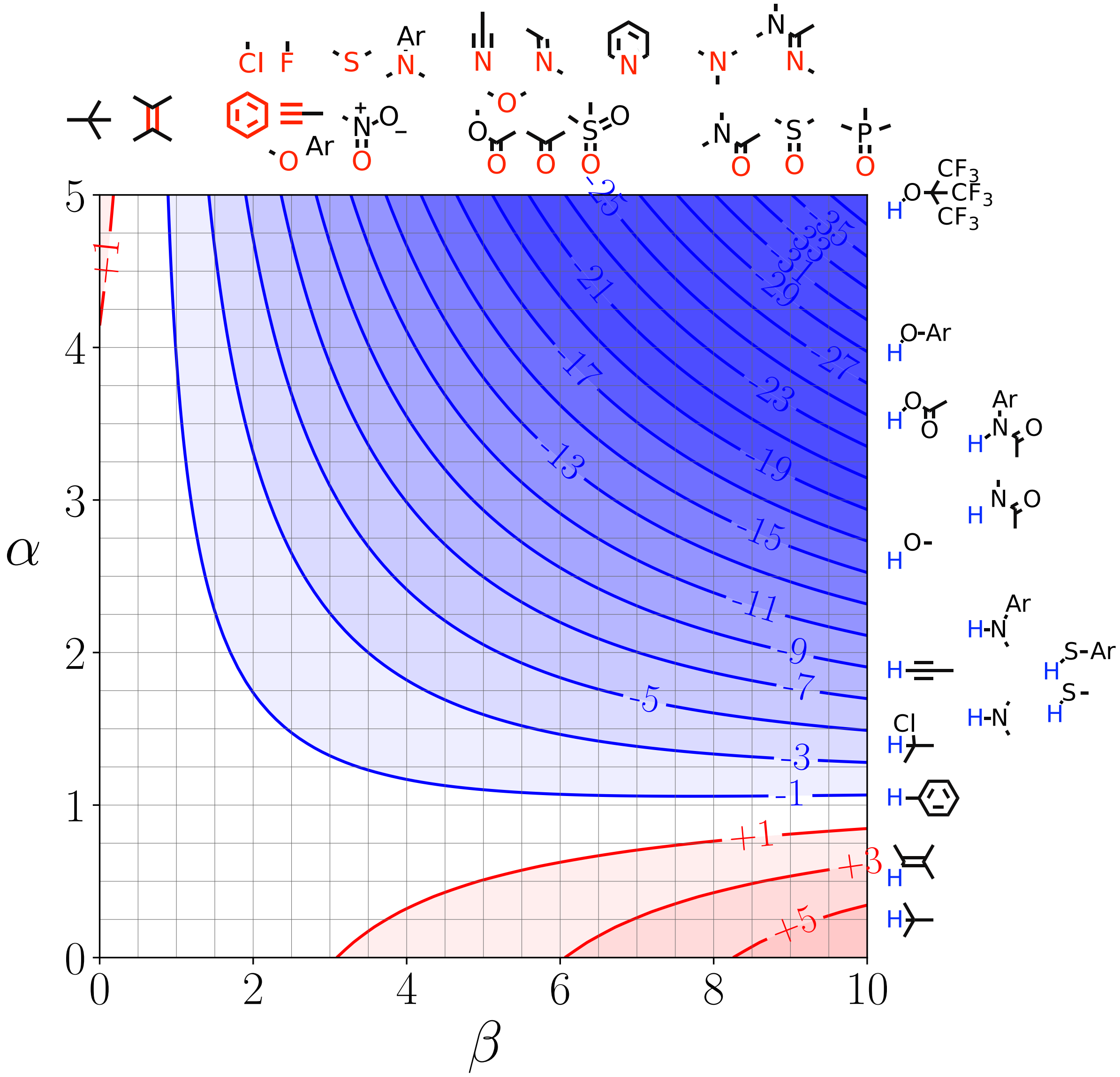

Figure S163: FGIP for chloroform at 298K.

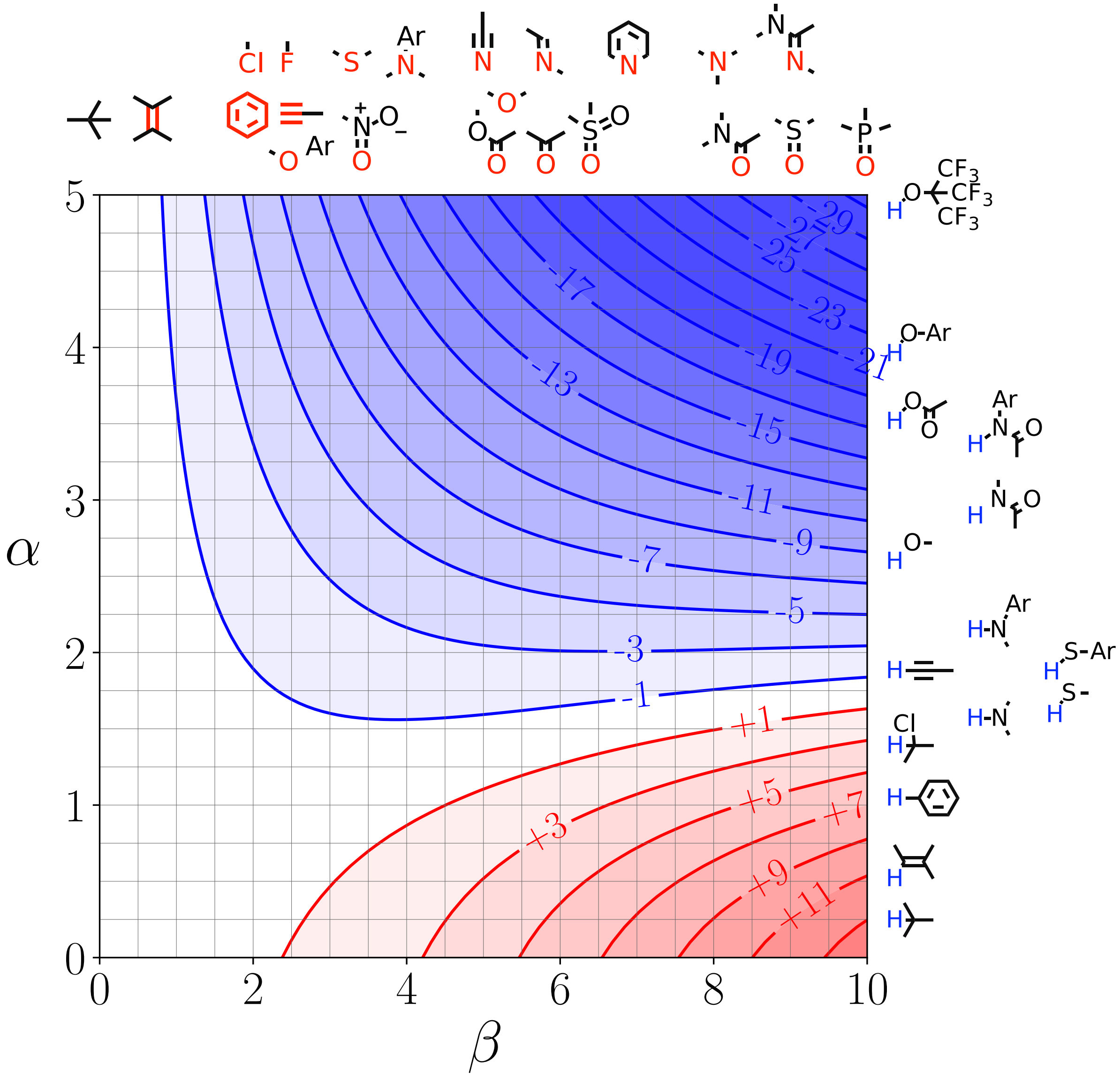

Figure S164: FGIP for 1,1,1-trichloroethane at 298K.

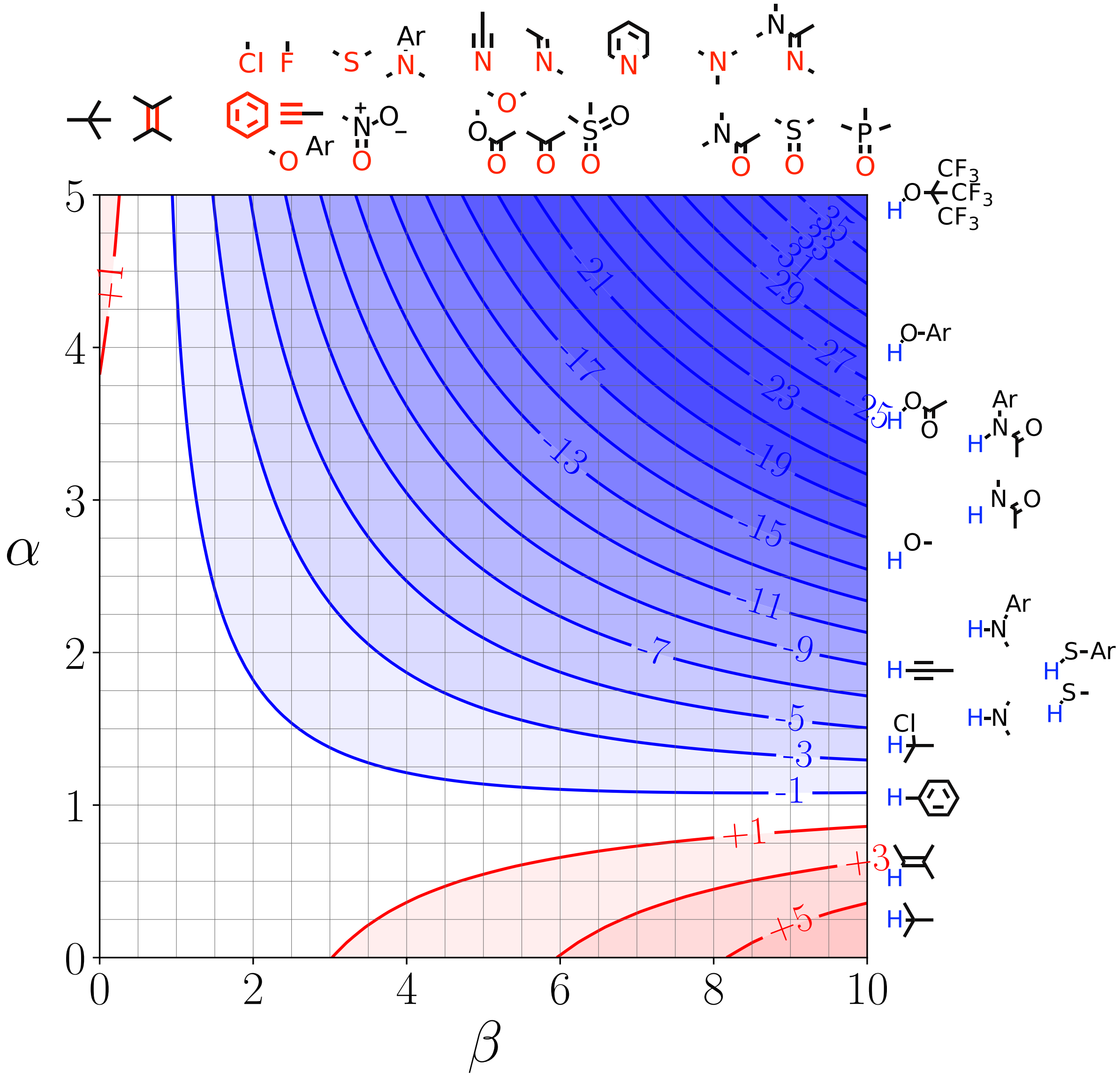

Figure S165: FGIP for 1,1,2-trichloroethane at 298K.

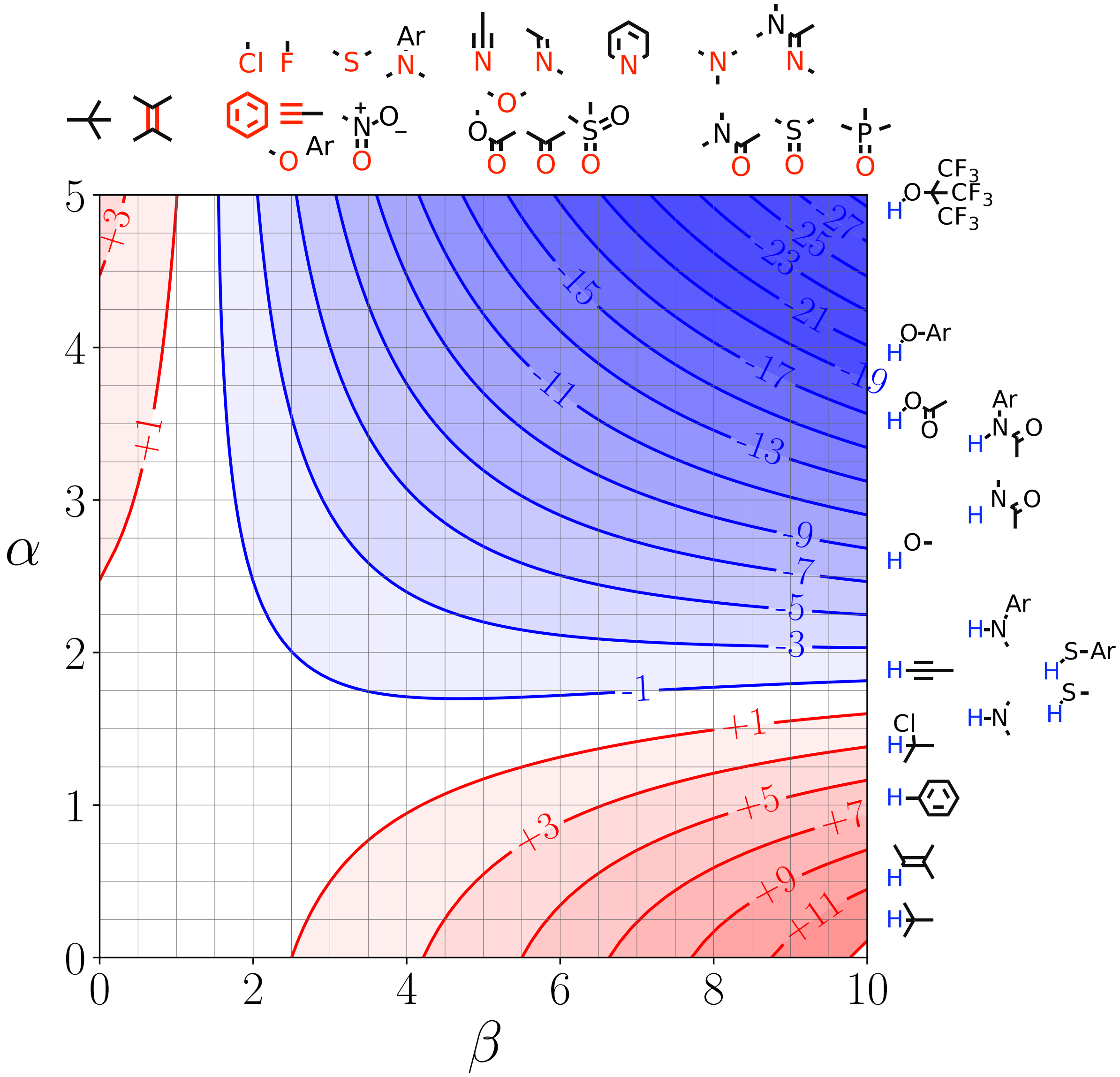

Figure S166: FGIP for trichloroethylene at 298K.

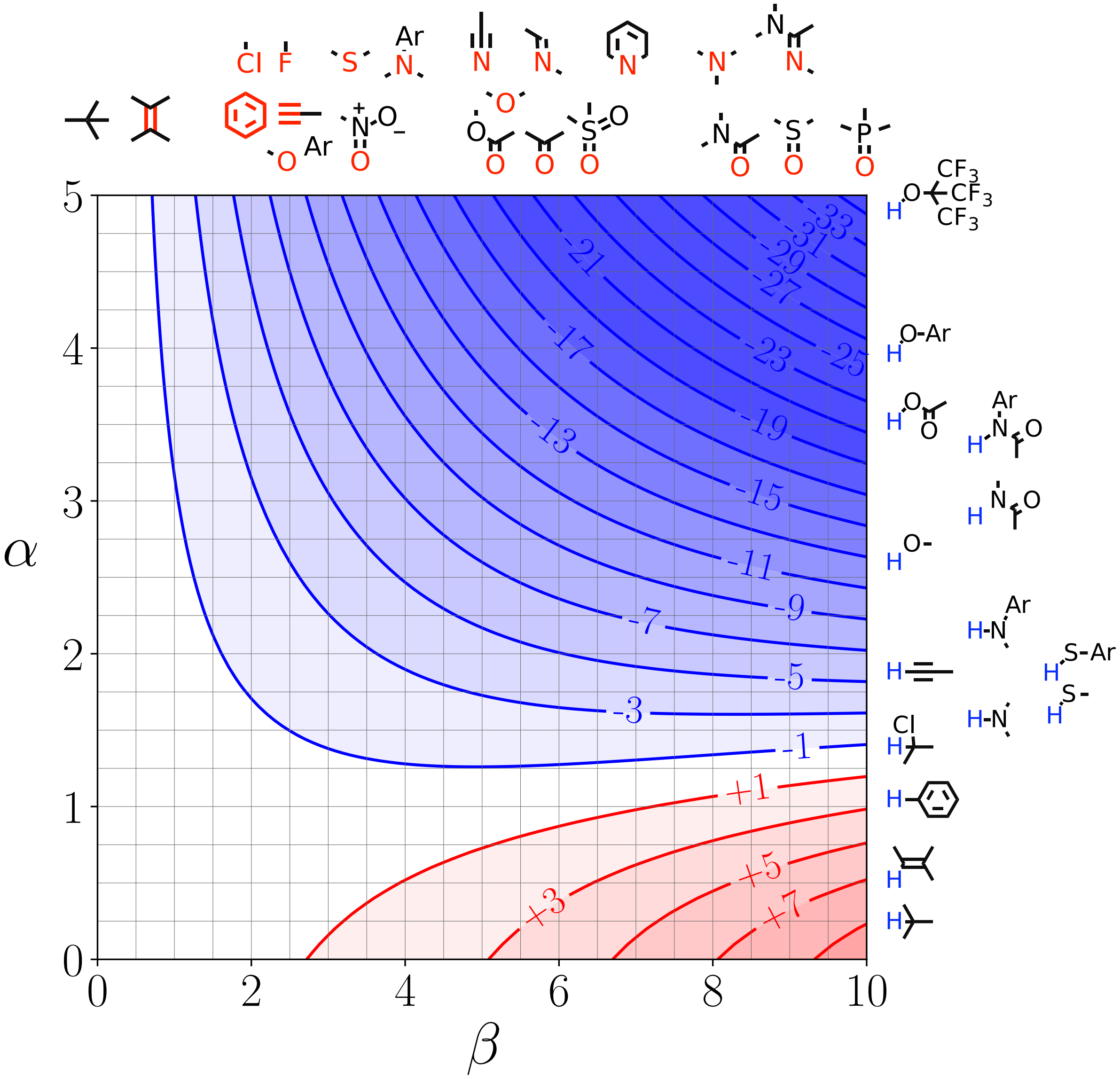

Figure S167: FGIP for 1,2,4-trichlorobenzene at 298K.

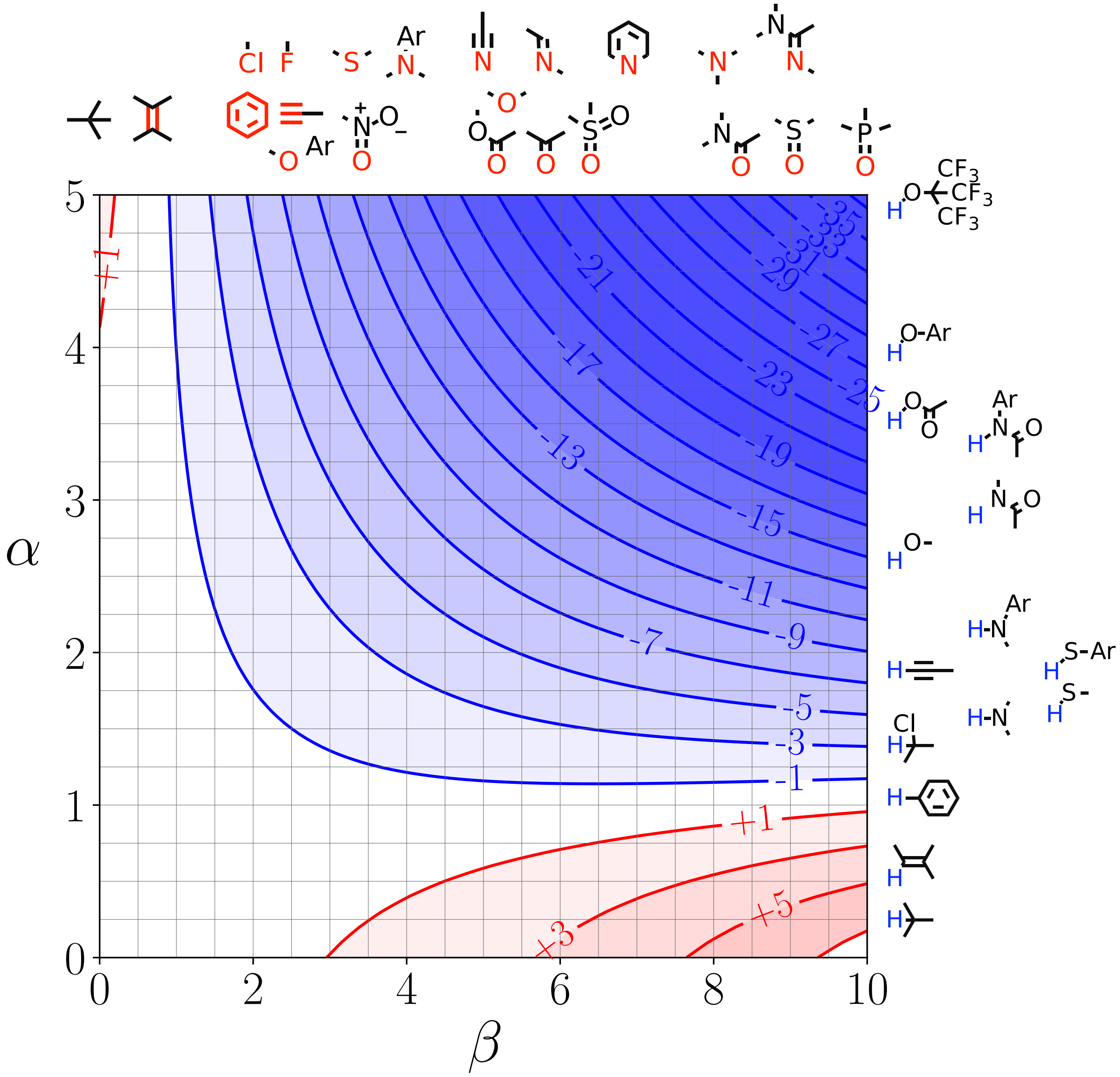

Figure S168: FGIP for carbon tetrachloride at 298K.

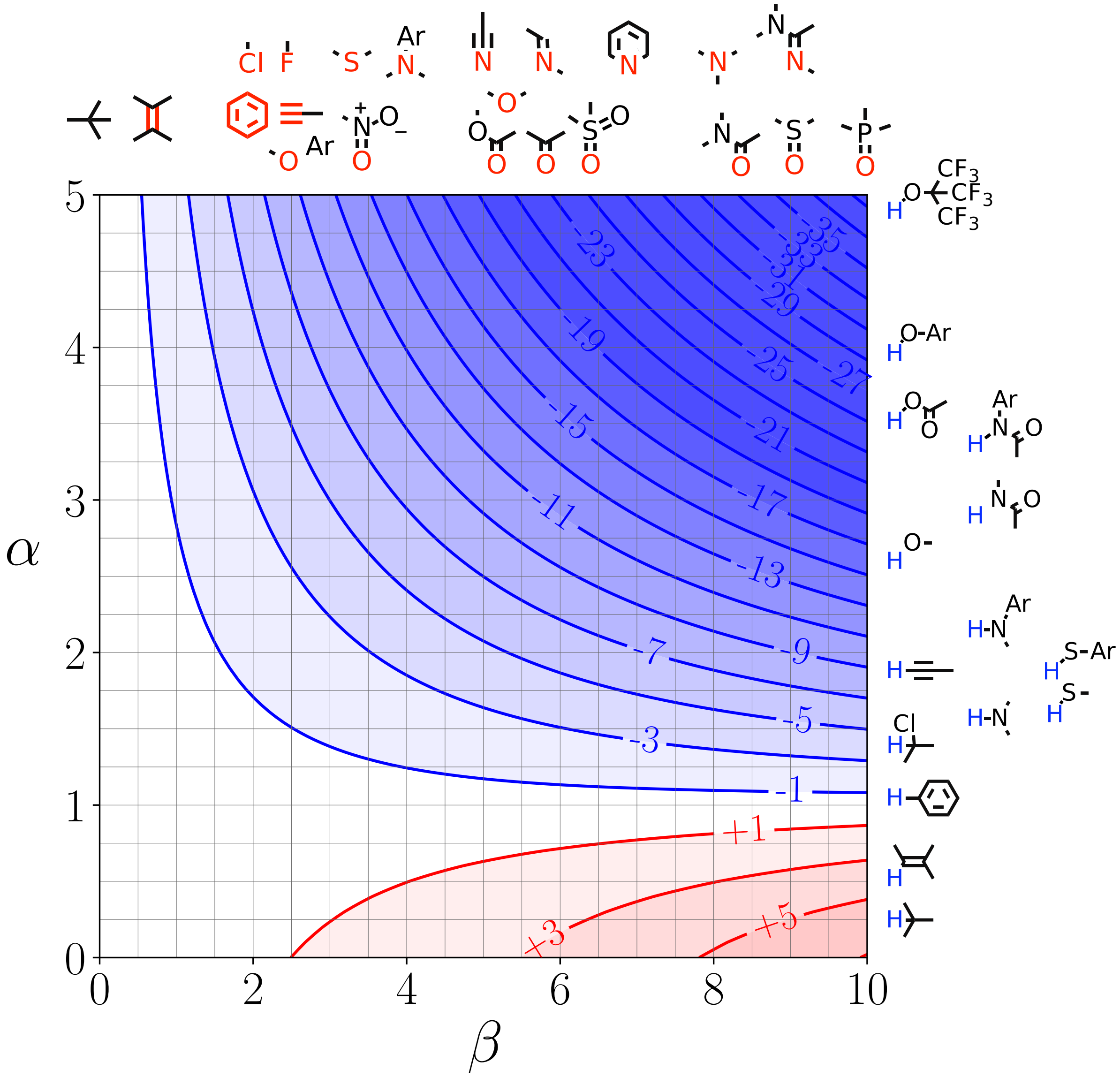

Figure S169: FGIP for tetrachloroethylene at 298K.

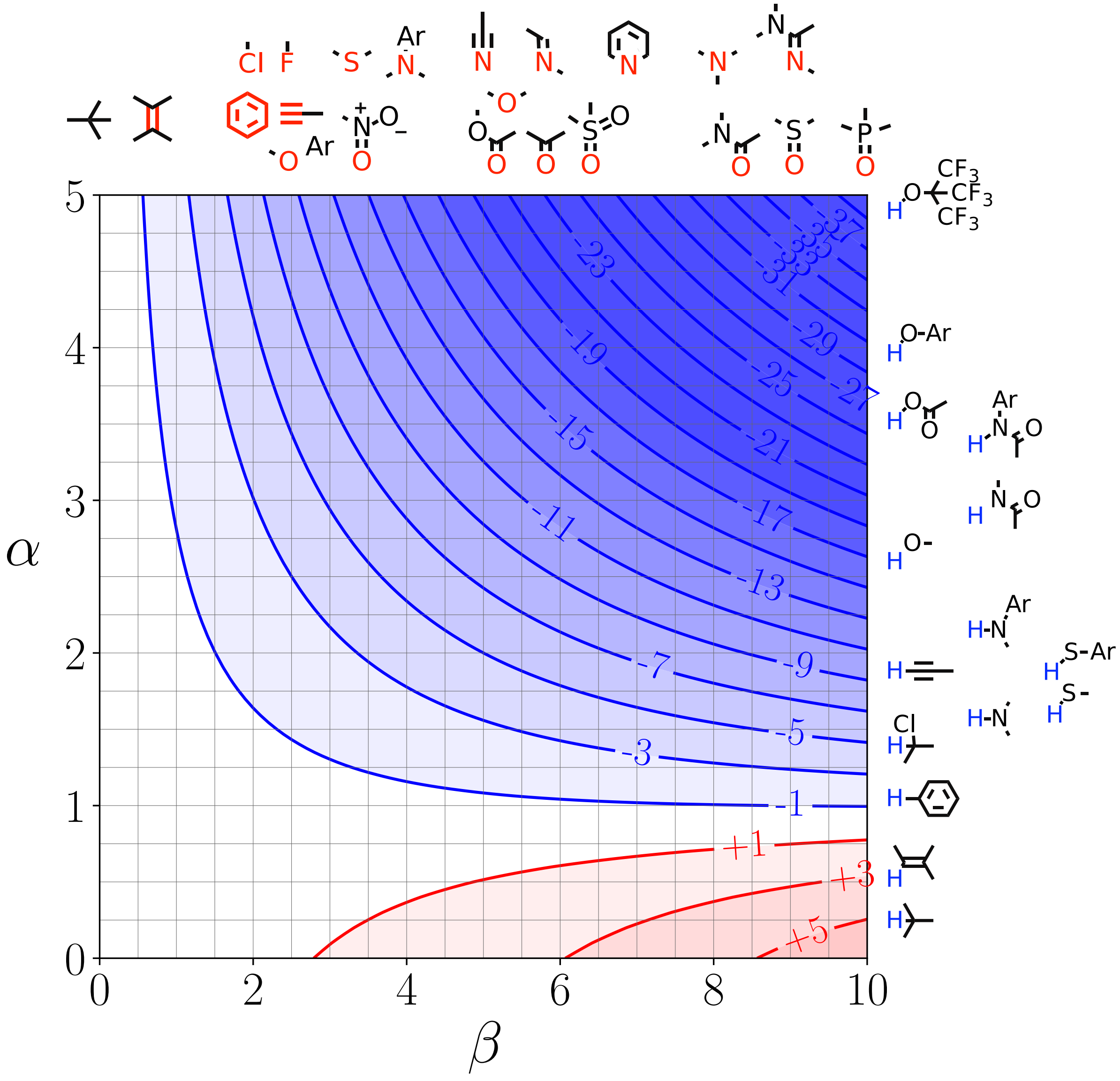

Figure S170: FGIP for 1,1,2,2-tetrachloroethane at 298K.

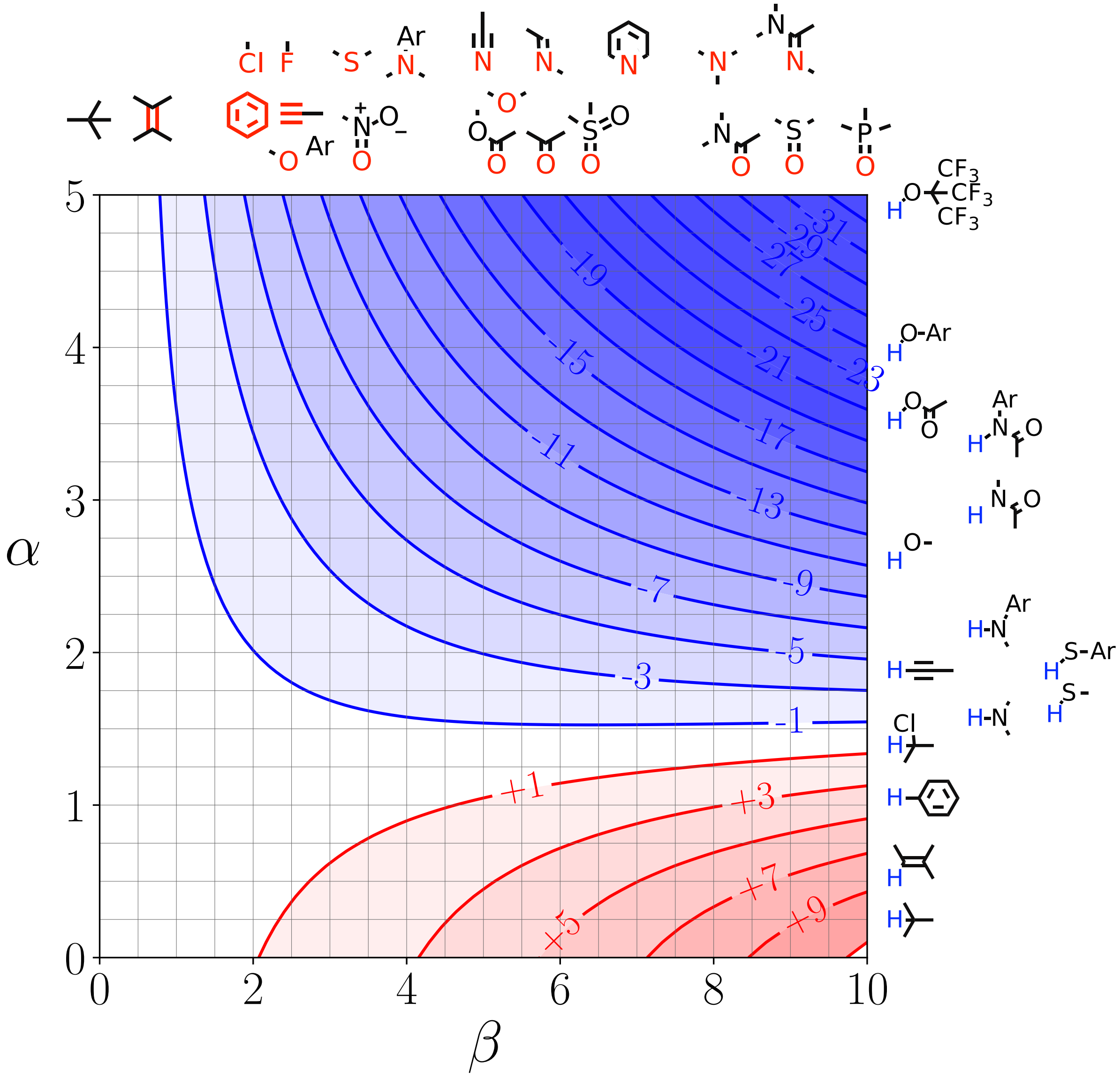

Figure S171: FGIP for pentachloroethane at 298K.

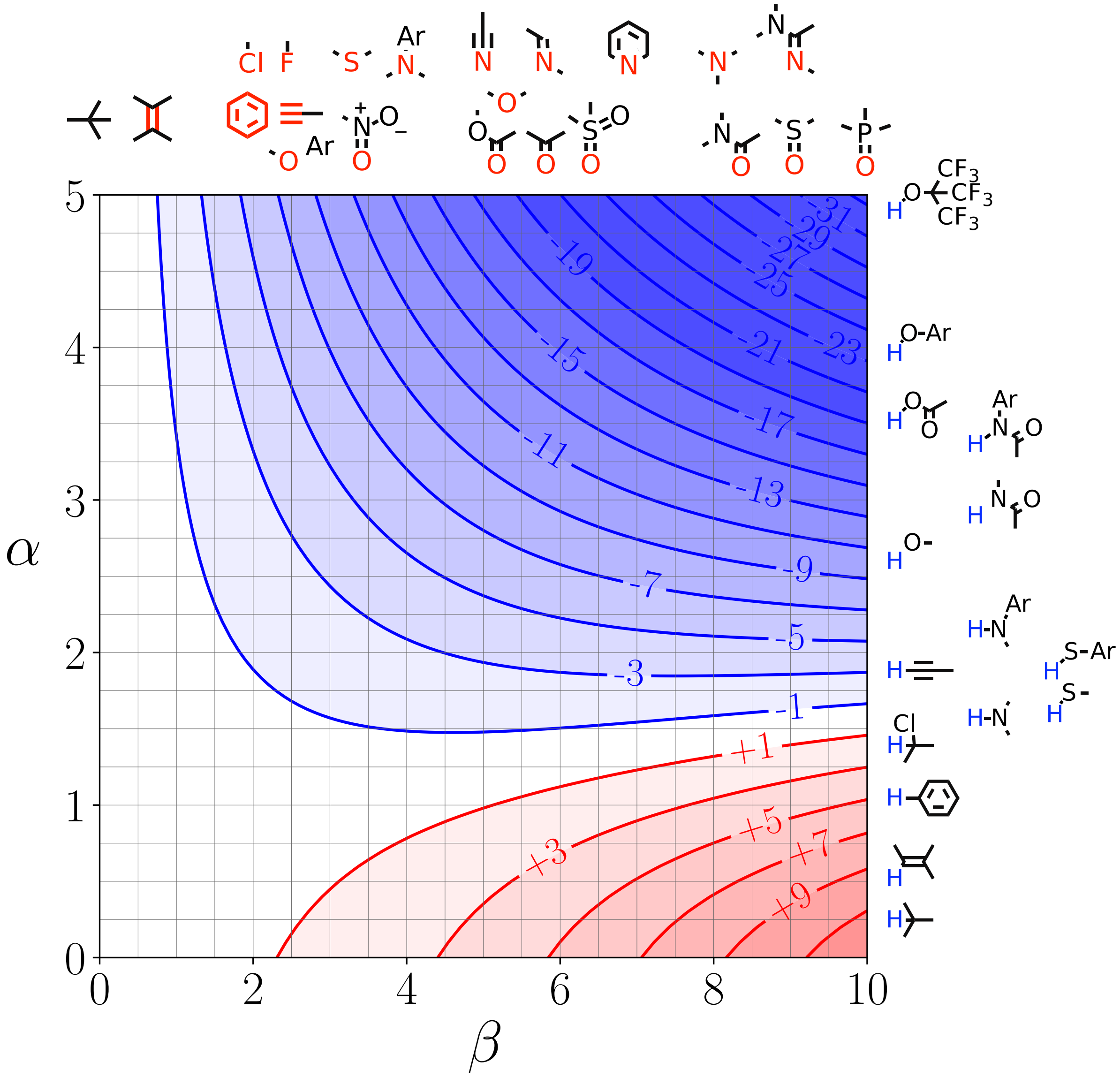

Figure S172: FGIP for 1-bromobutane at 298K.

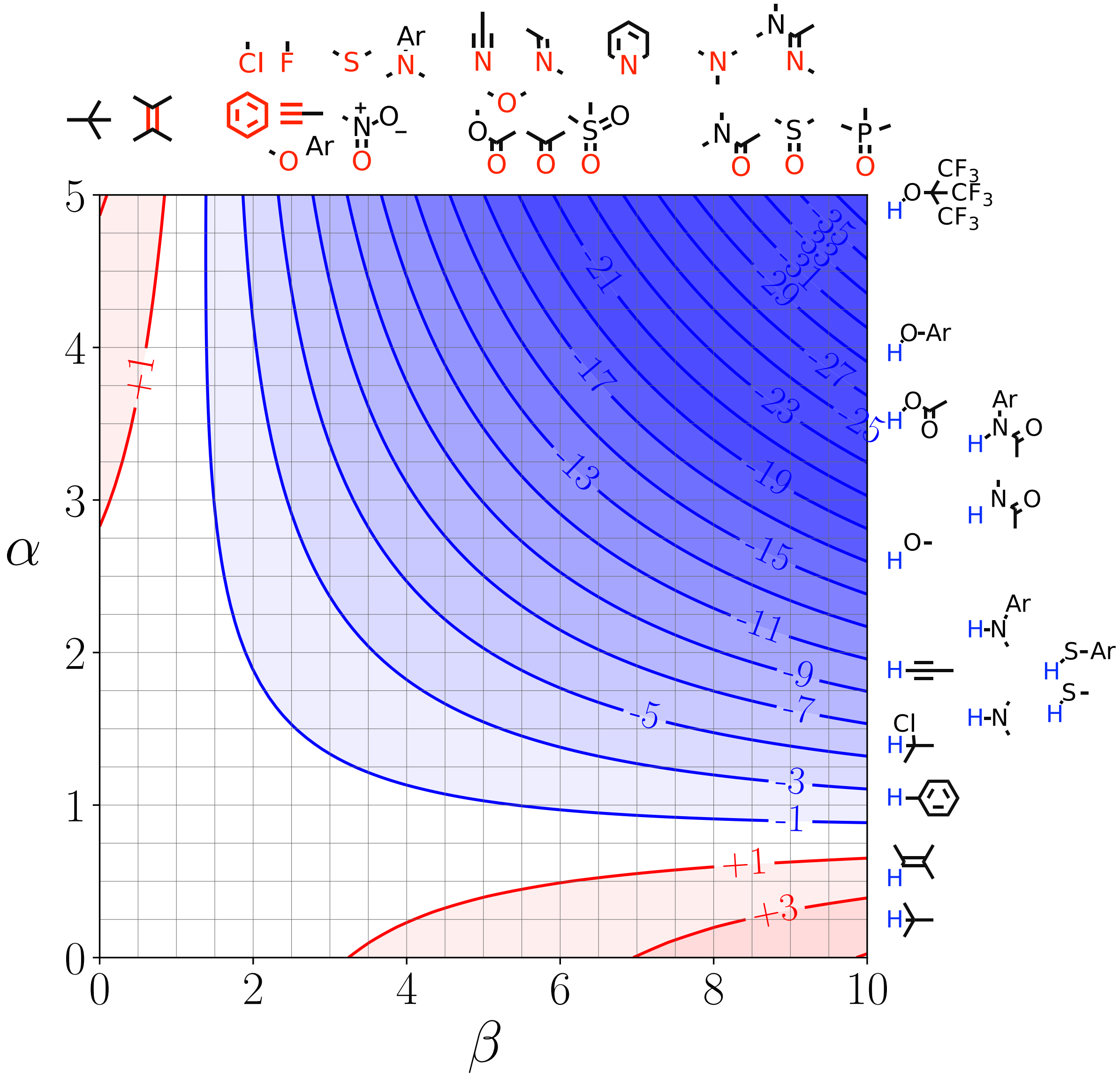

Figure S173: FGIP for bromobenzene at 298K.

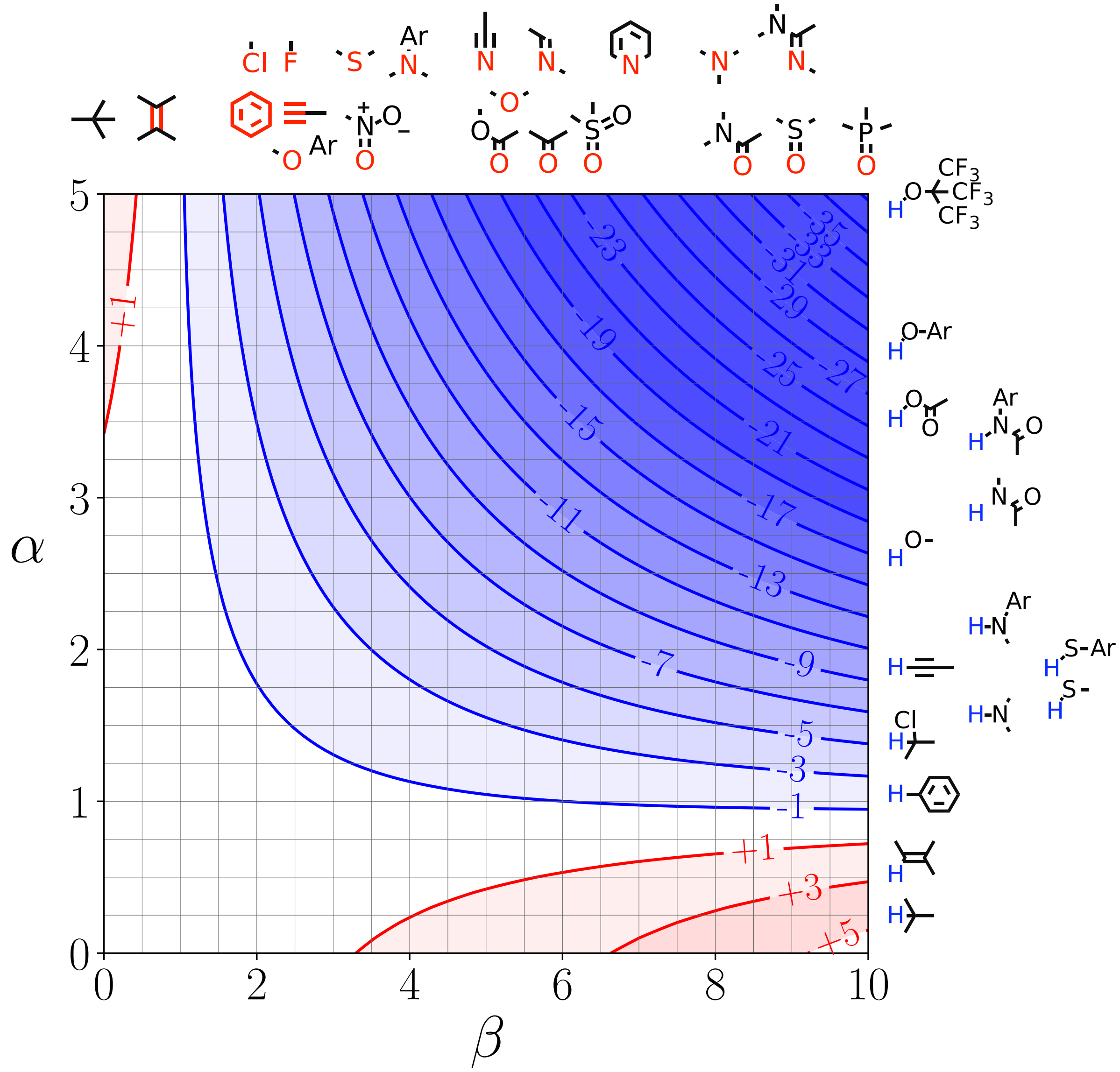

Figure S174: FGIP for dibromomethane at 298K.

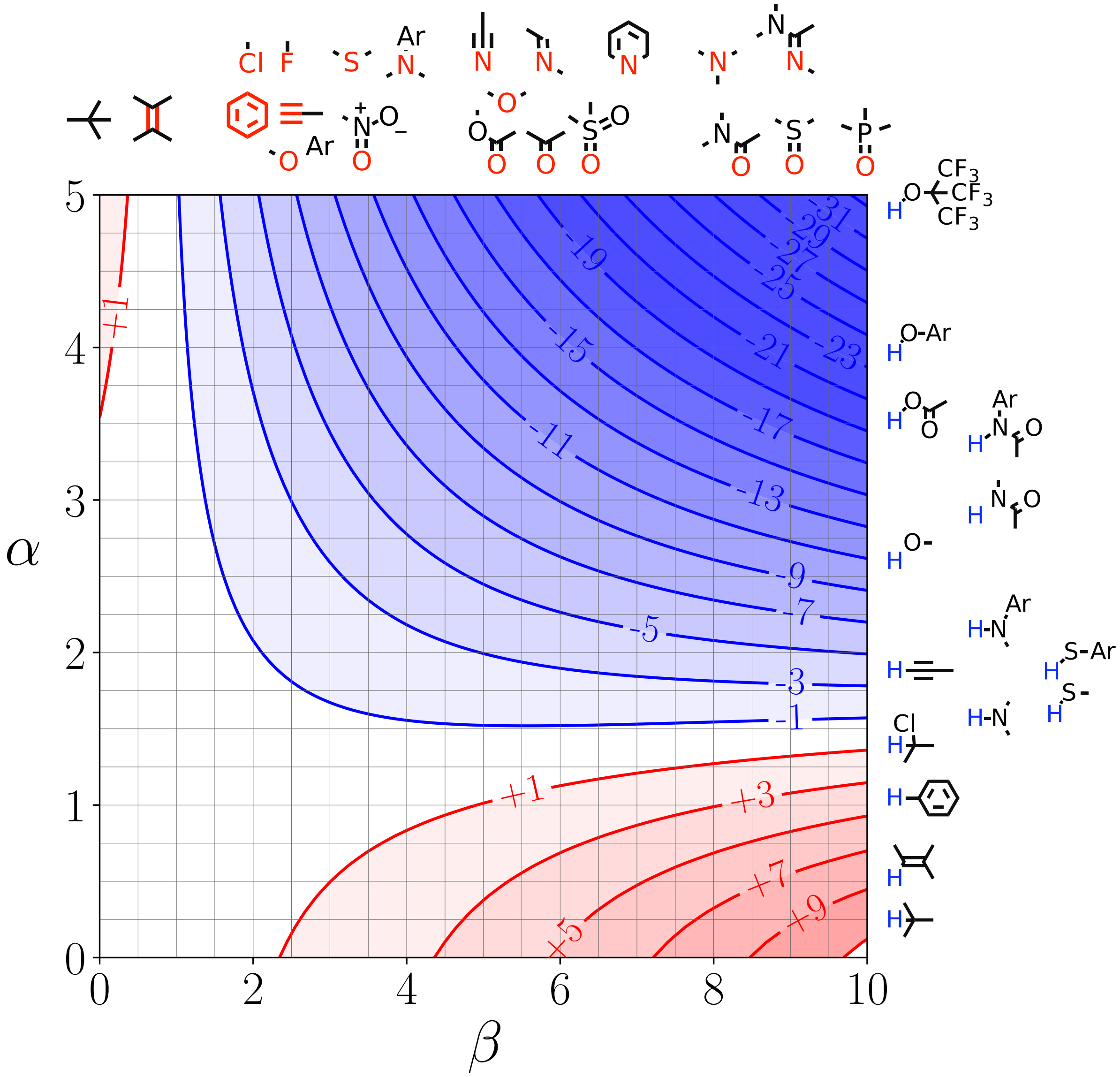

Figure S175: FGIP for 1,2-dibromoethane at 298K.

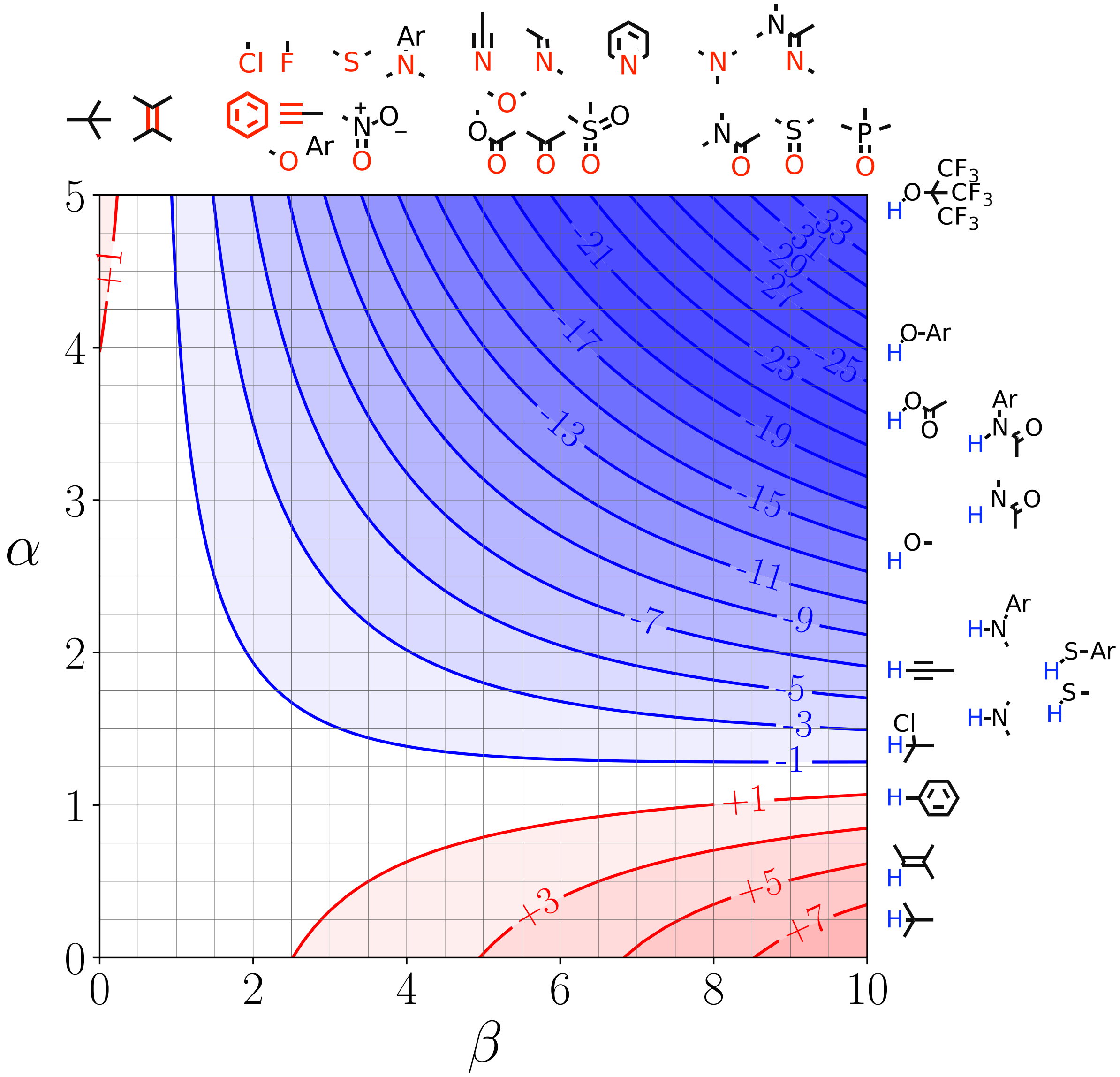

Figure S176: FGIP for bromoform at 298K.

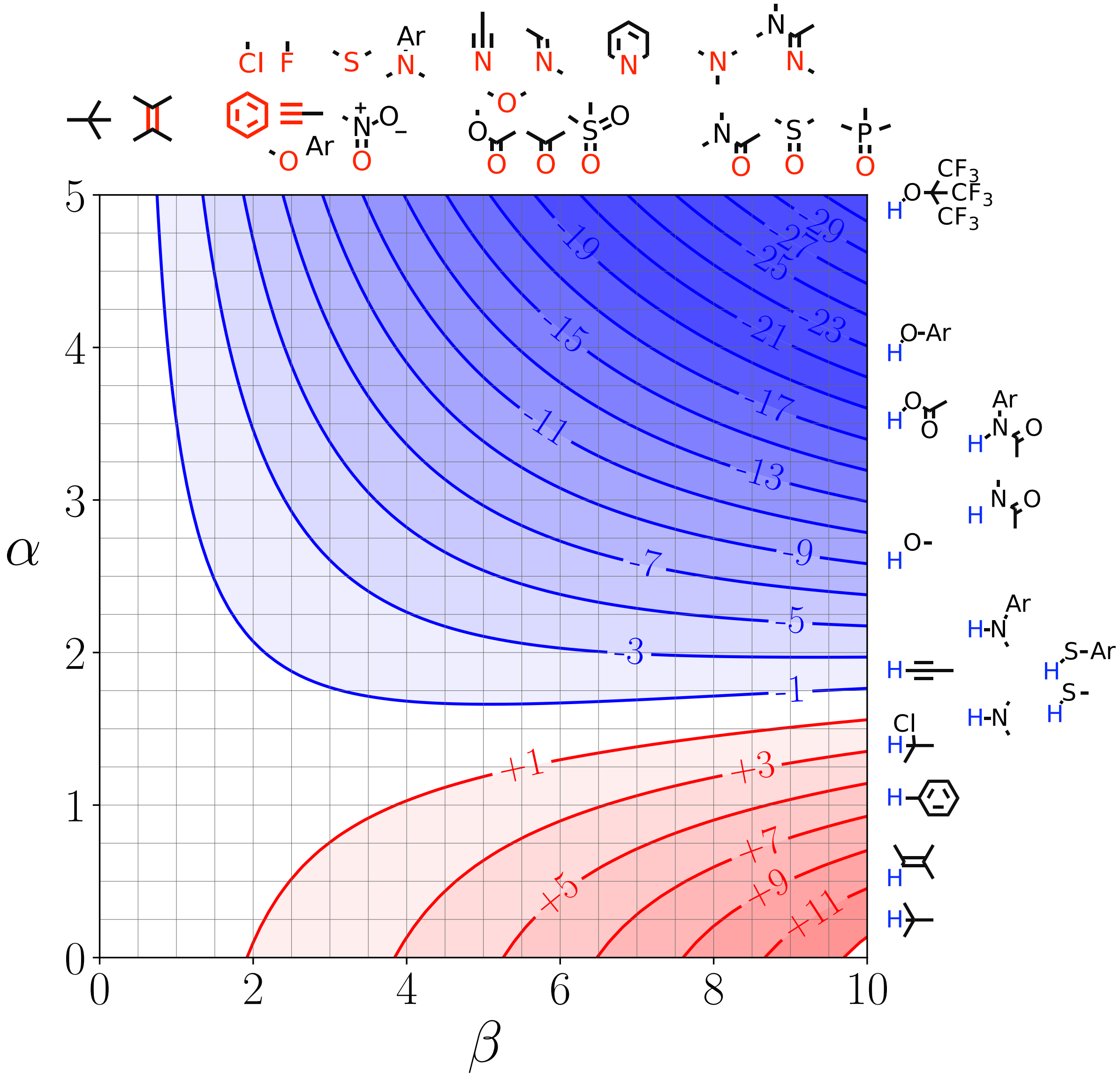

Figure S177: FGIP for n-butyl iodide at 298K.

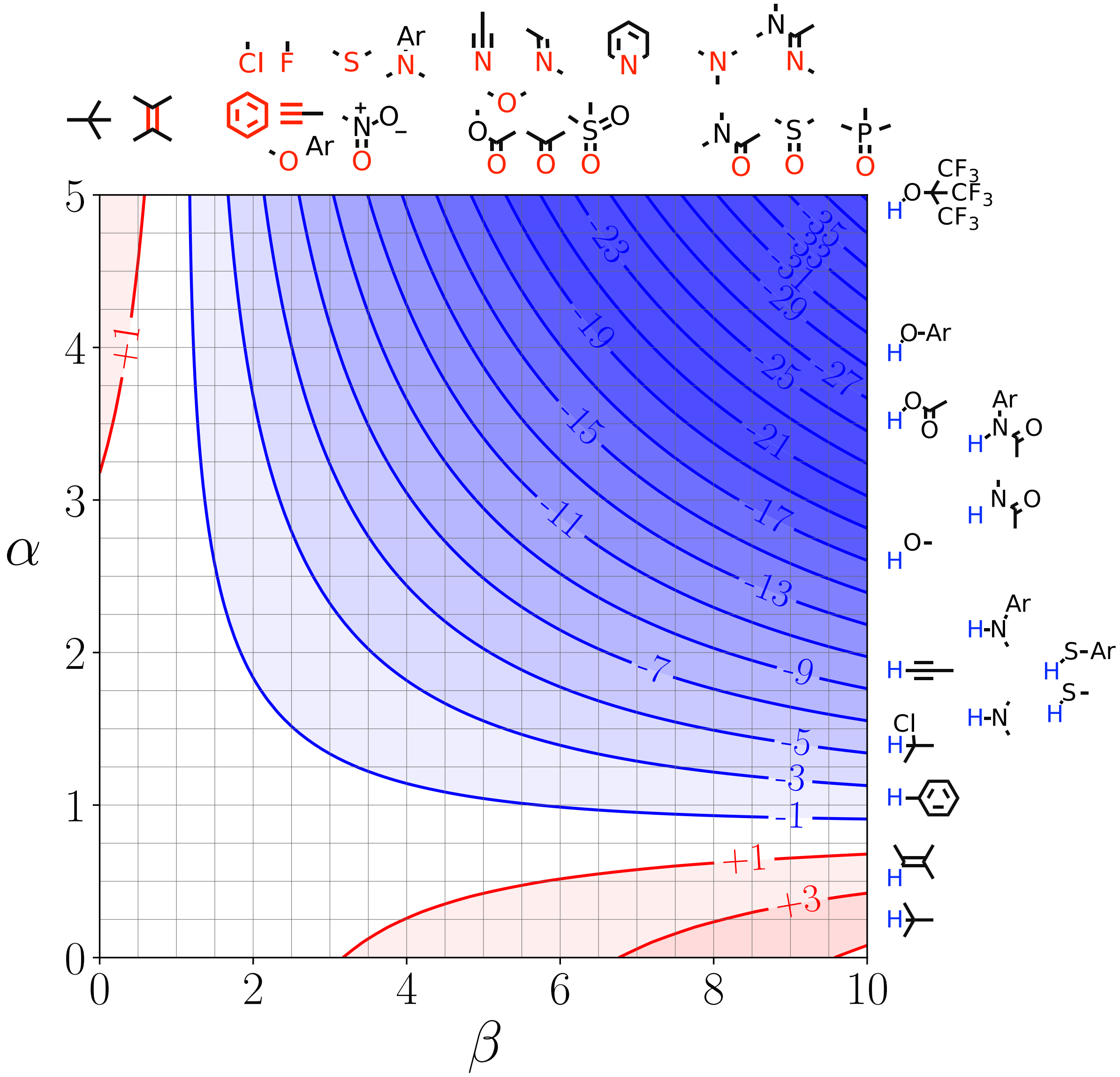

Figure S178: FGIP for iodobenzene at 298K.

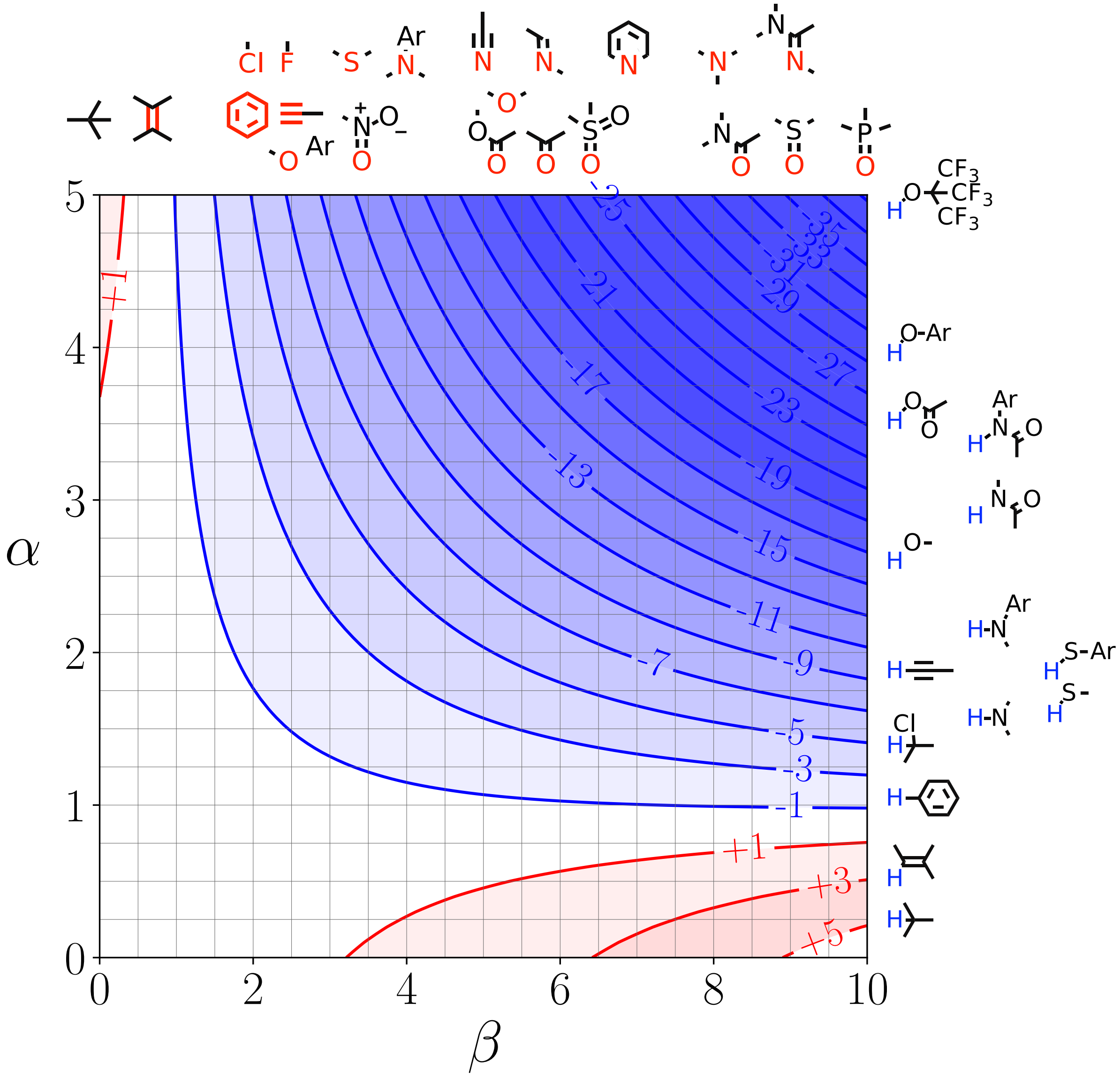

Figure S179: FGIP for methylene iodide at 298K.

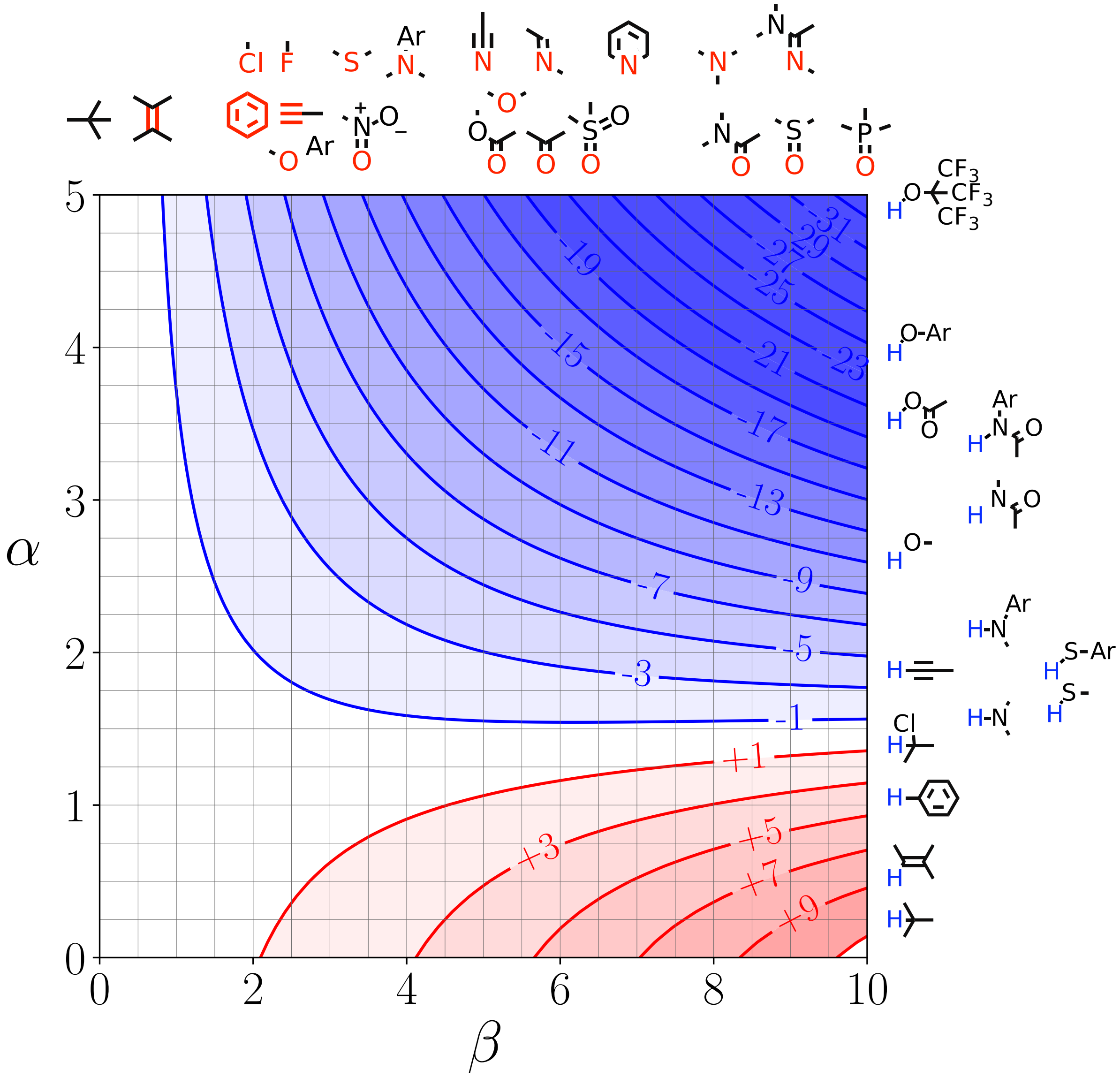

Figure S180: FGIP for n-butylamine at 298K.

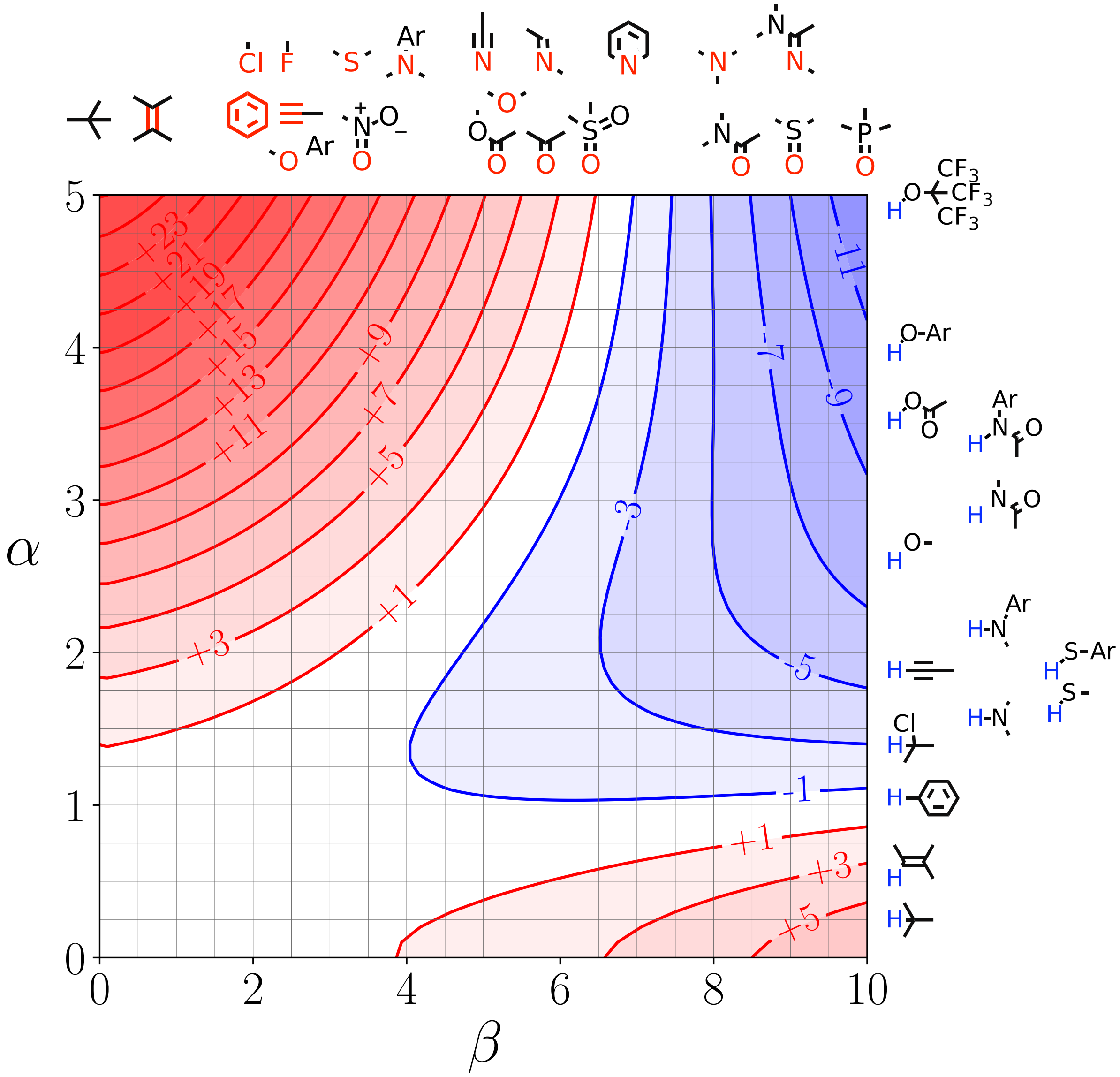

Figure S181: FGIP for benzylamine at 298K.

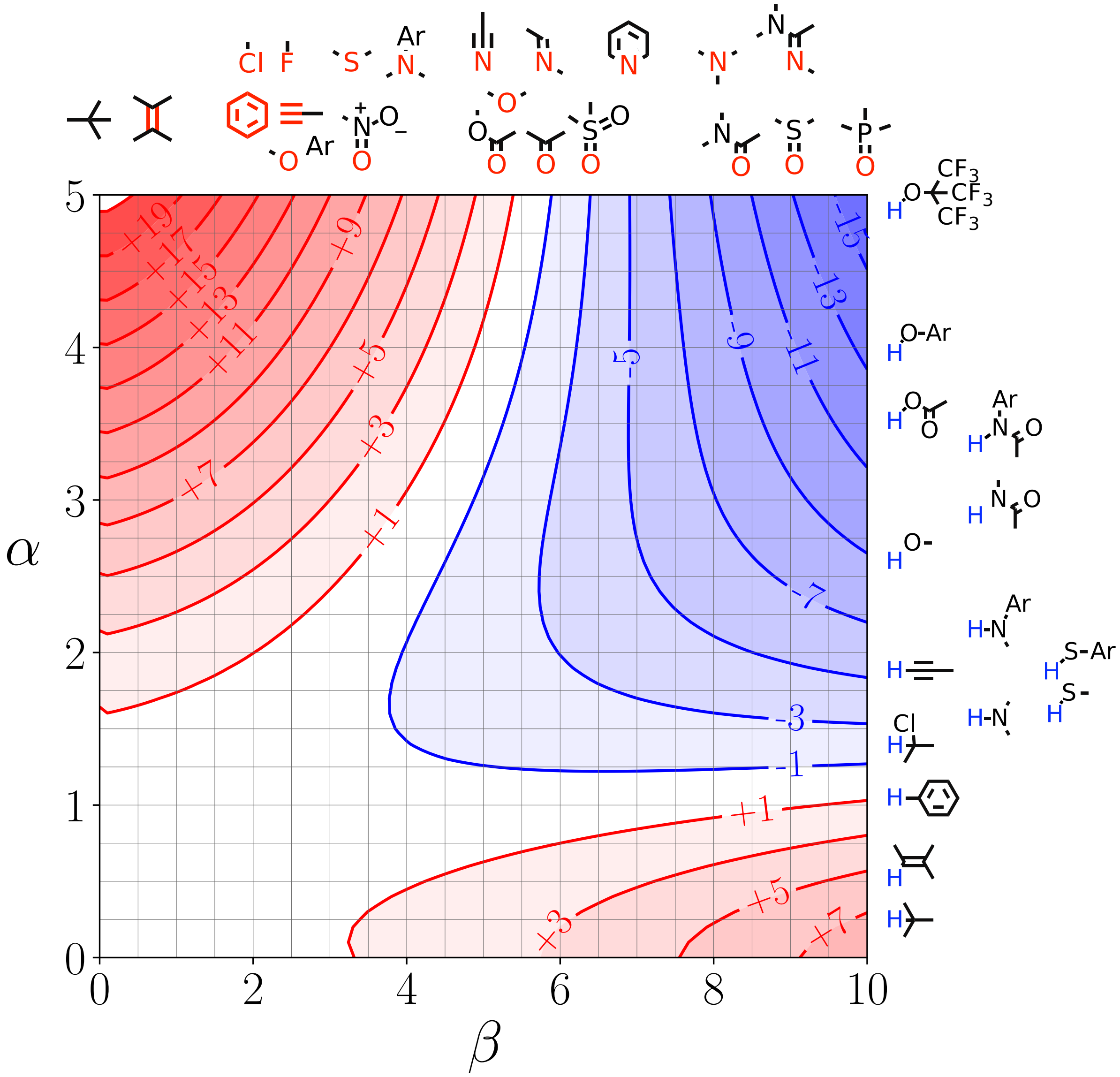

Figure S182: FGIP for ethylenediamine at 298K.

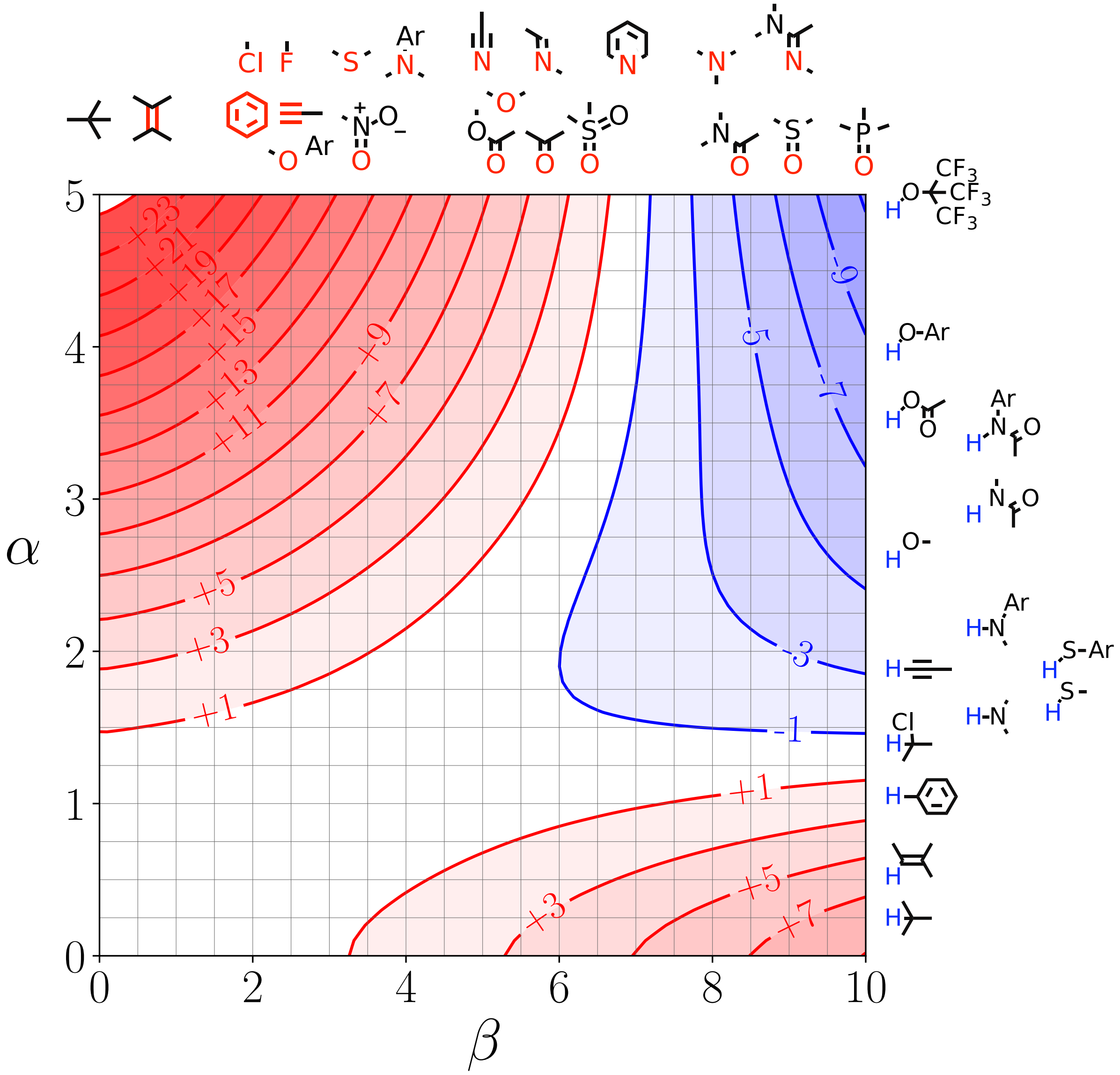

Figure S183: FGIP for diethylamine at 298K.

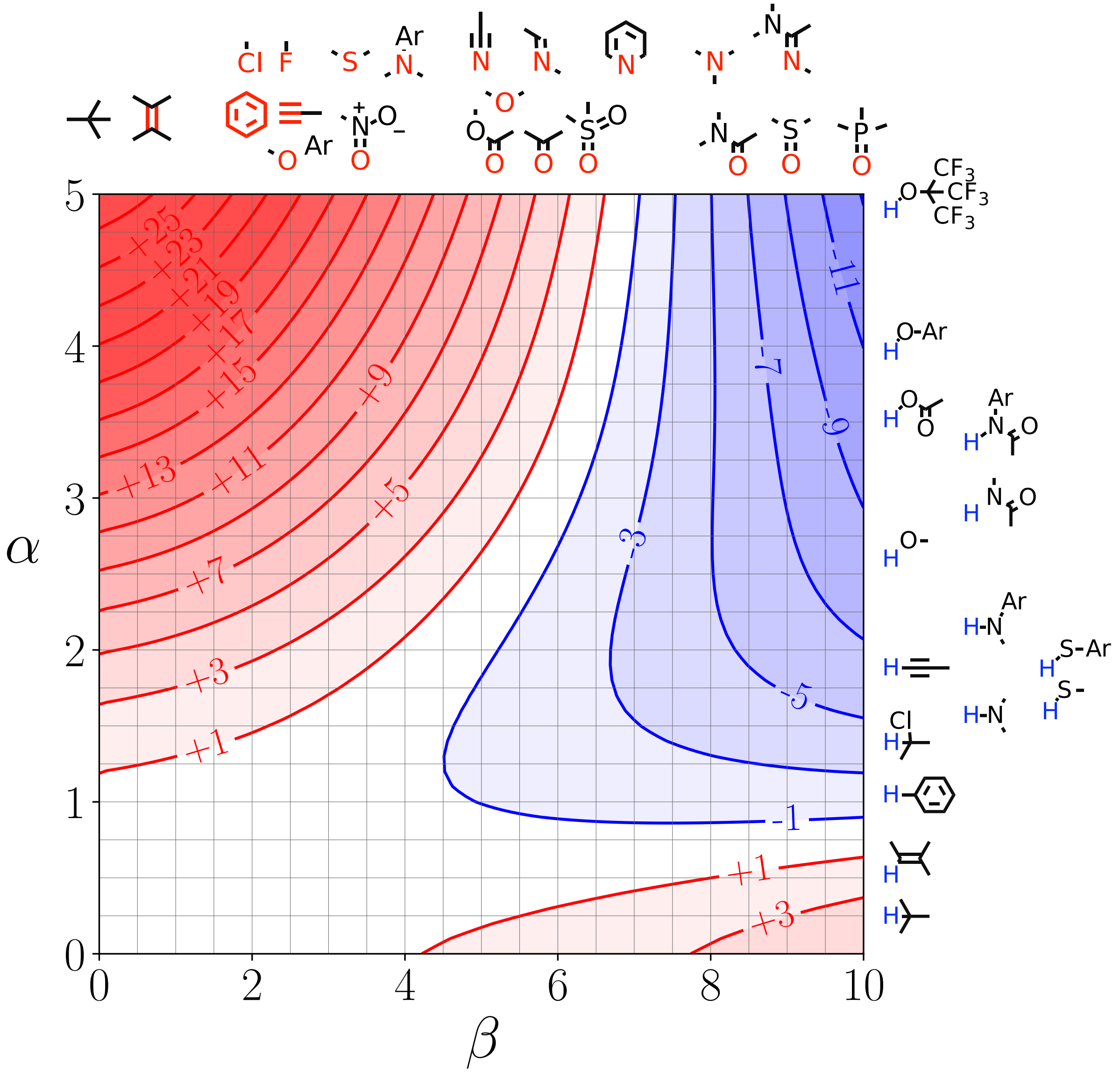

Figure S184: FGIP for di-n-butylamine at 298K.

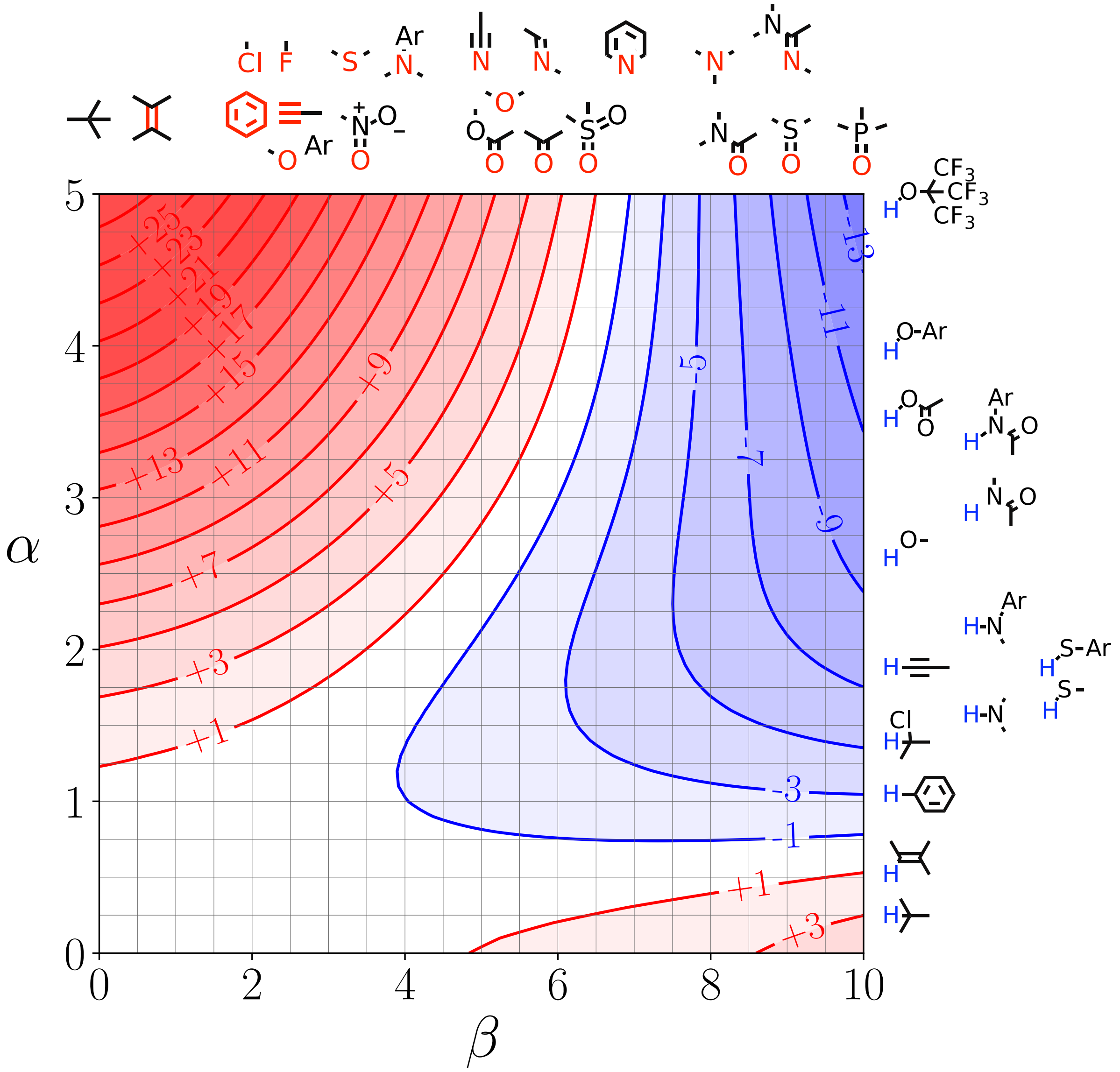

Figure S185: FGIP for pyrrole at 298K.

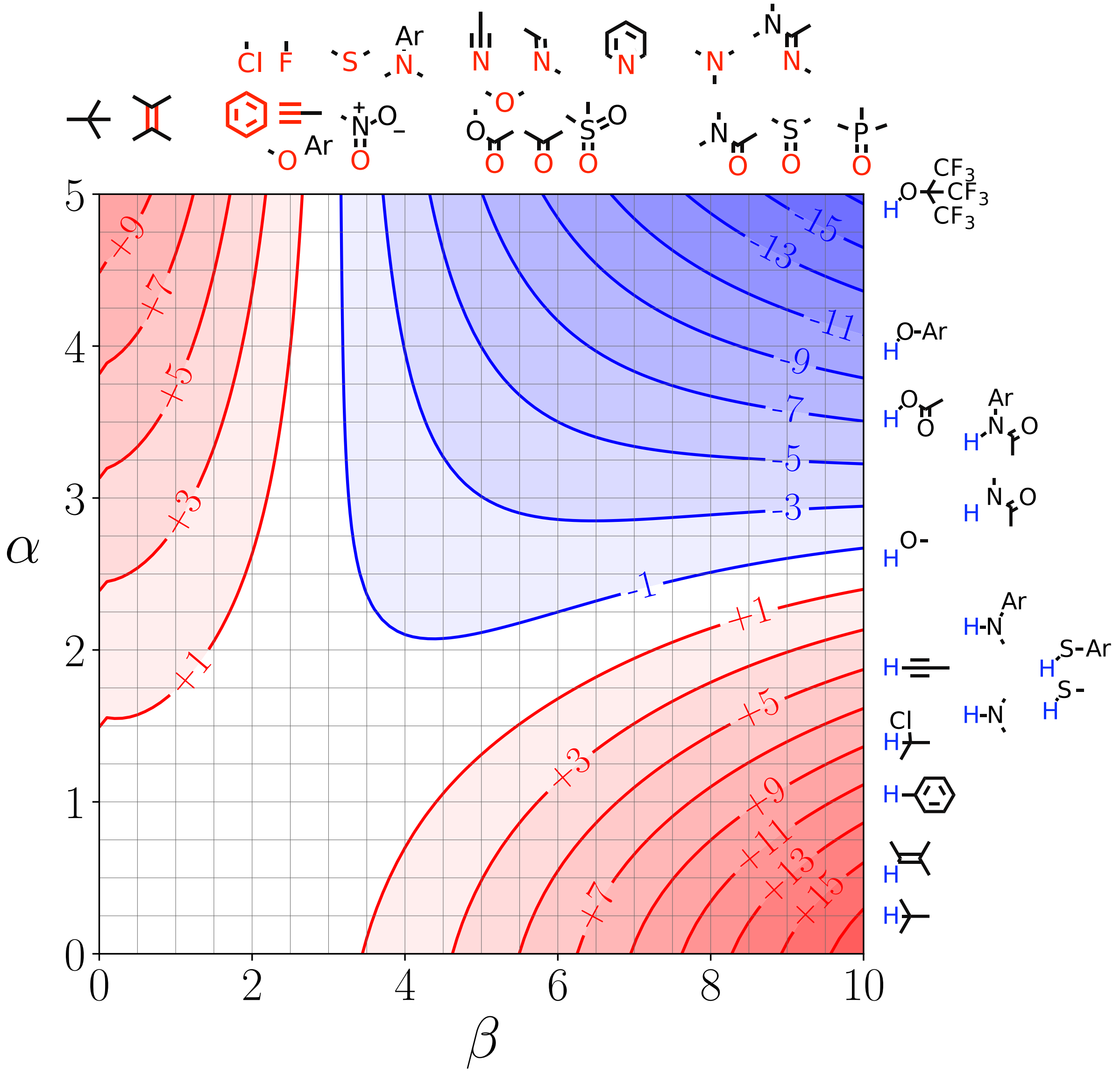

Figure S186: FGIP for pyrrolidine at 298K.

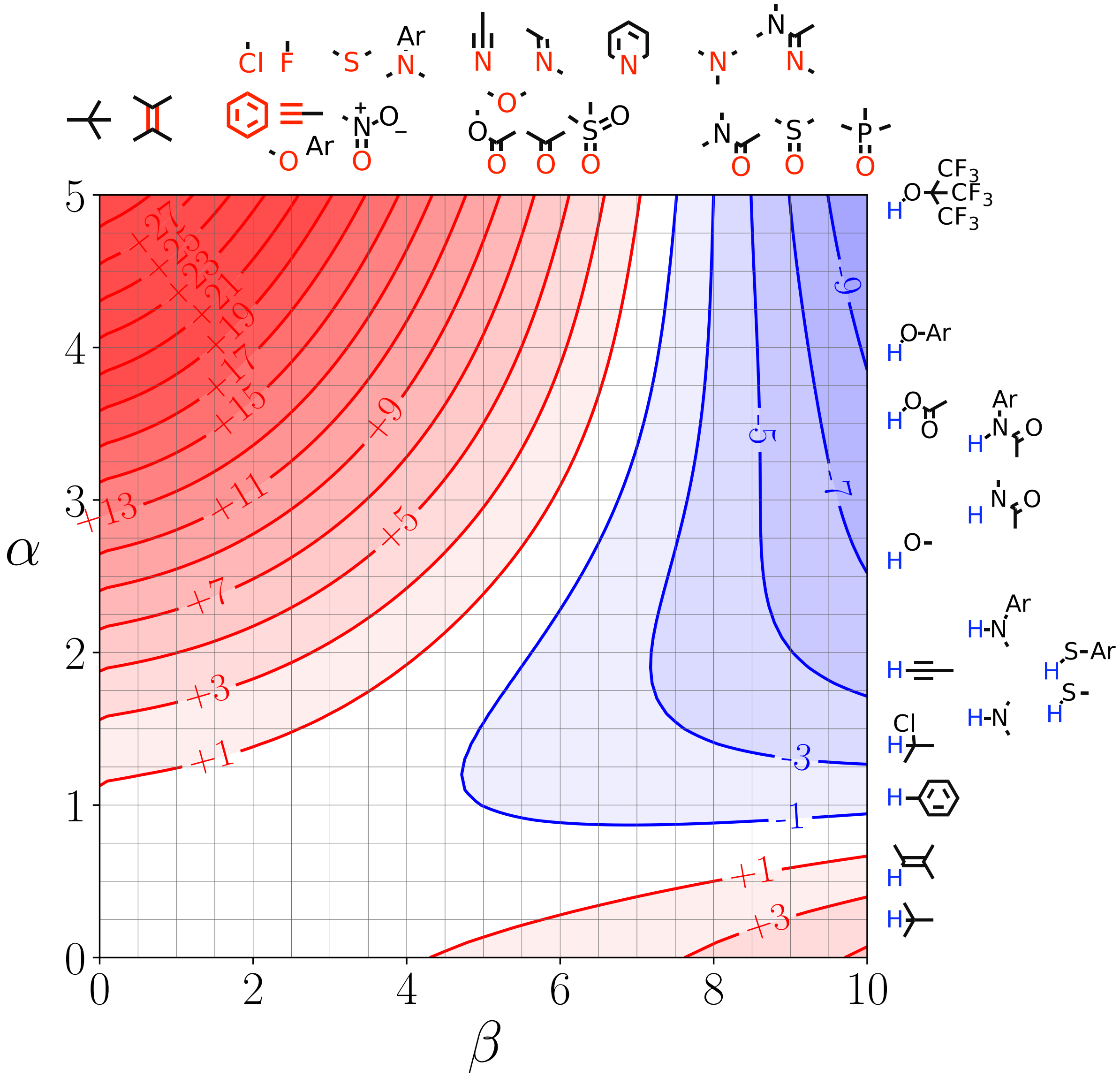

Figure S187: FGIP for piperidine at 298K.

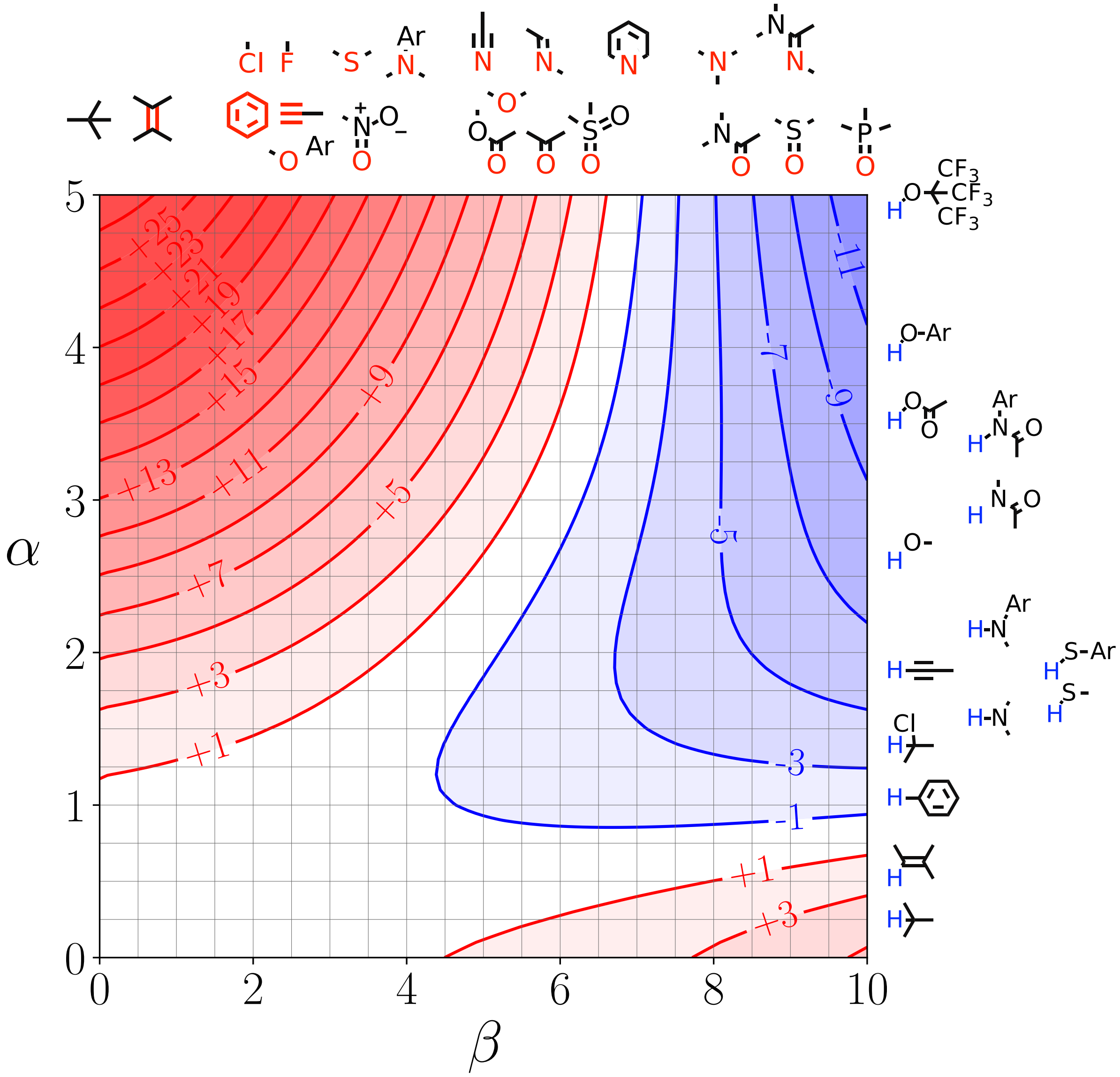

Figure S188: FGIP for morpholine at 298K.

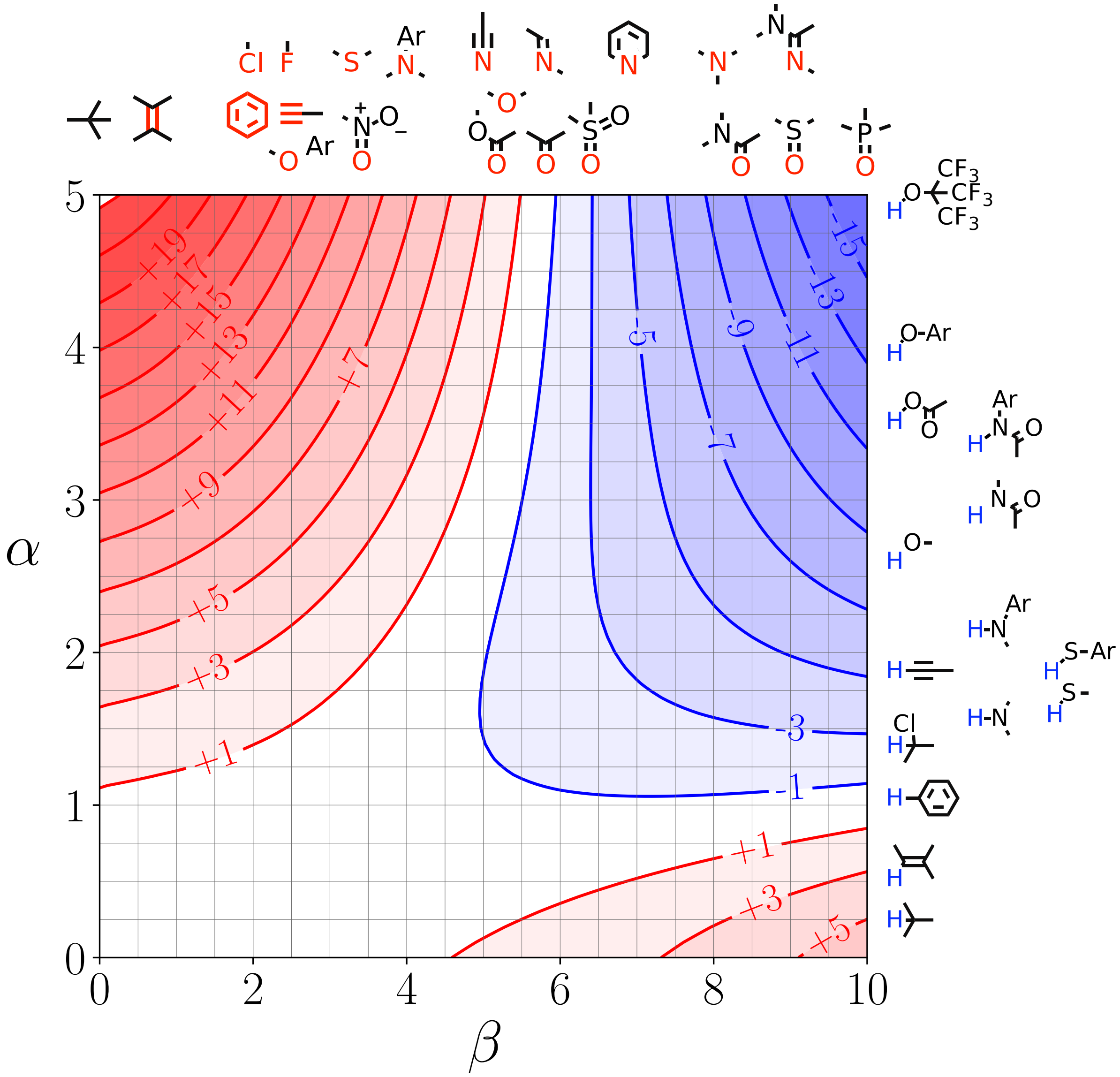

Figure S189: FGIP for triethylamine at 298K.

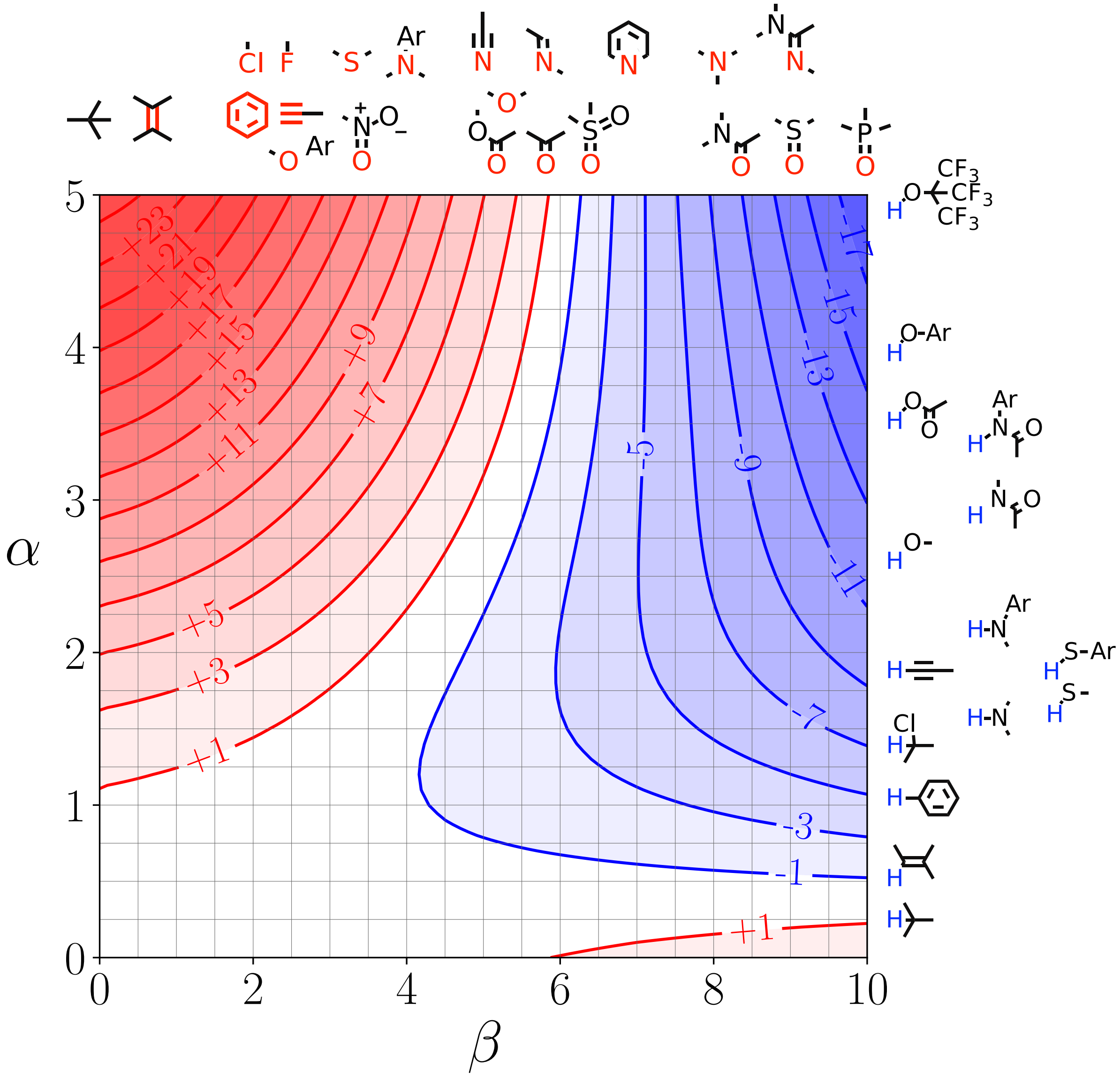

Figure S190: FGIP for tri-(n-butyl)amine at 298K.

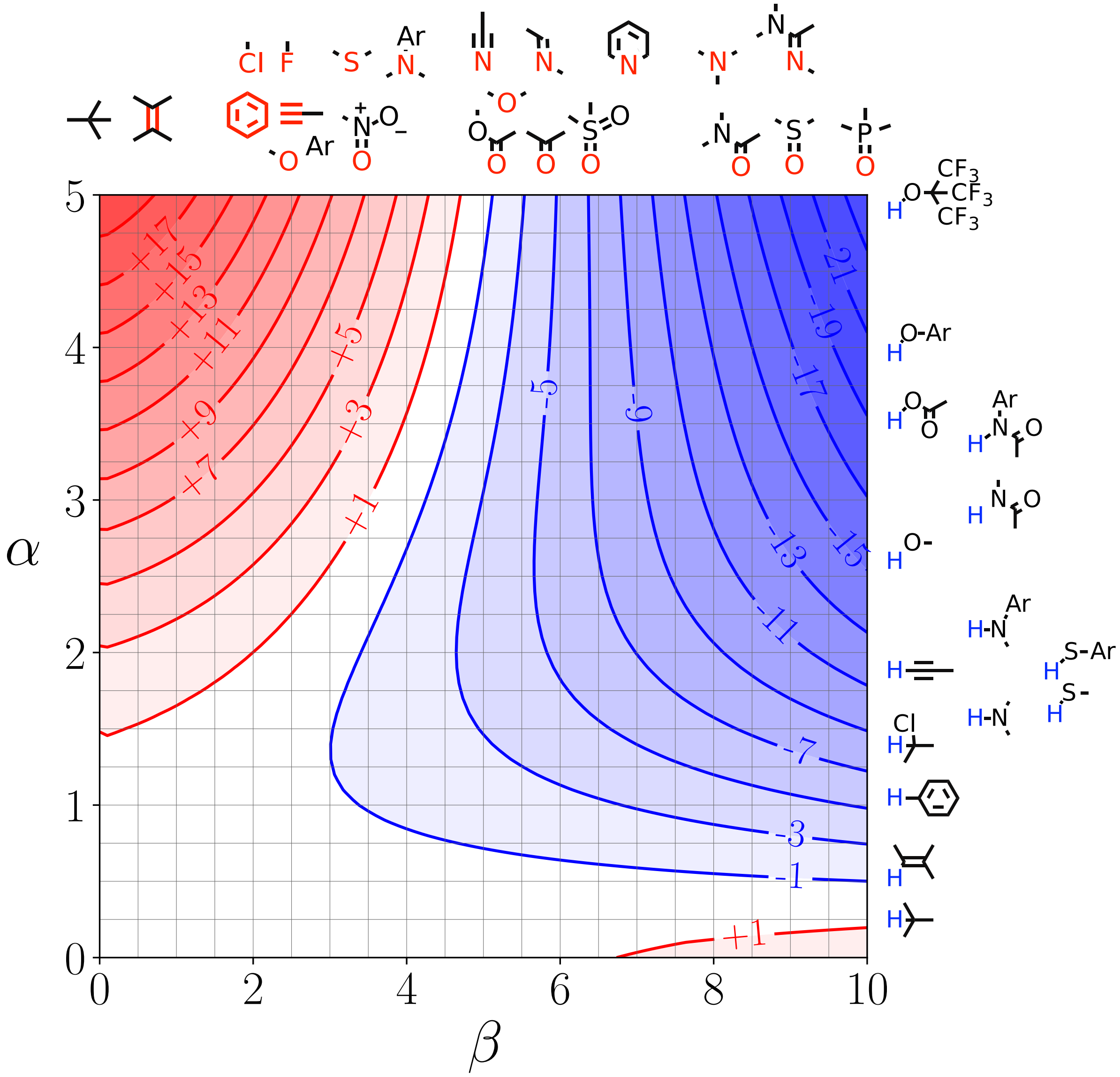

Figure S191: FGIP for aniline at 298K.

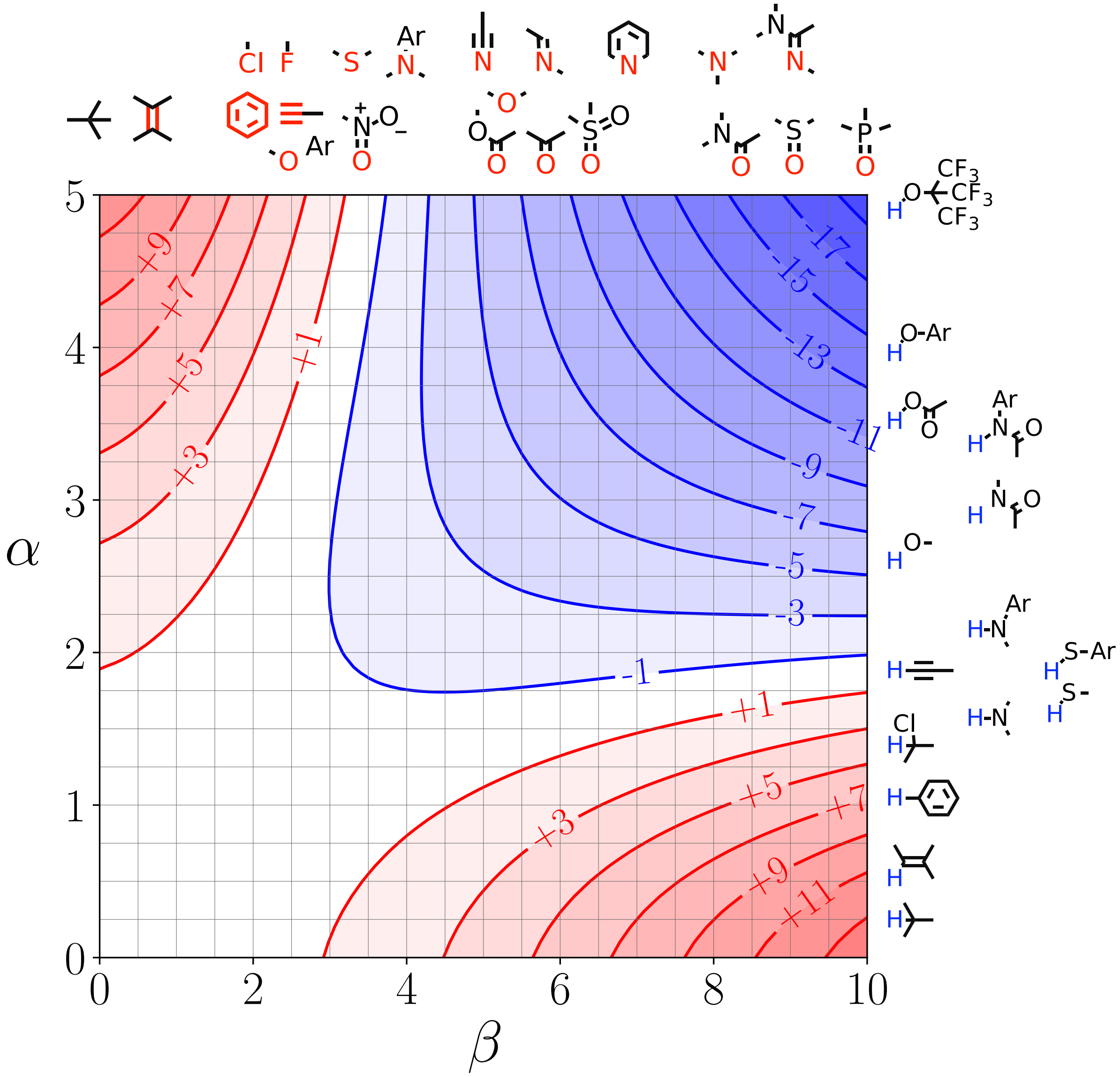

Figure S192: FGIP for o-chloroaniline at 298K.

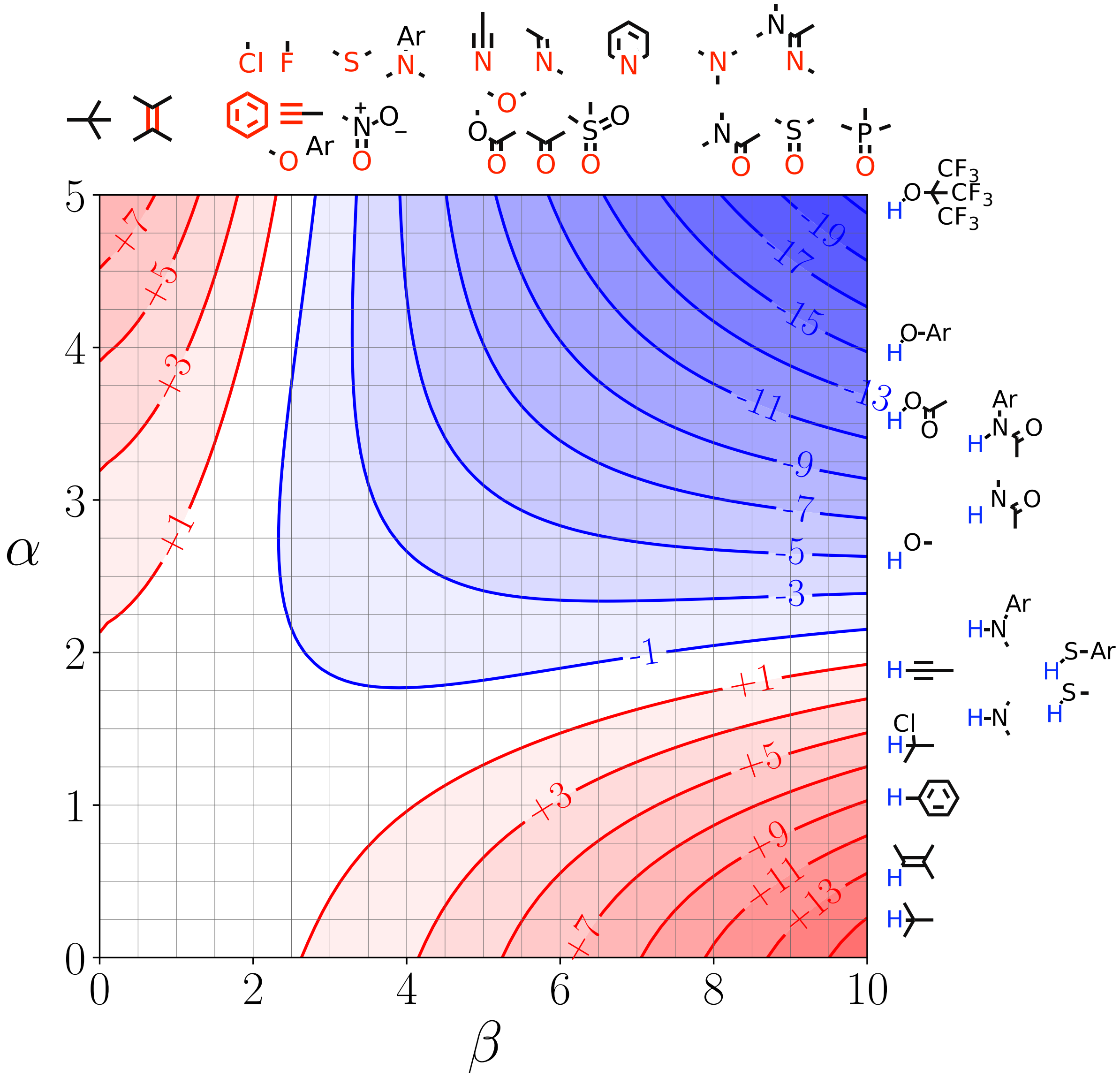

Figure S193: FGIP for methylphenylamine at 298K.

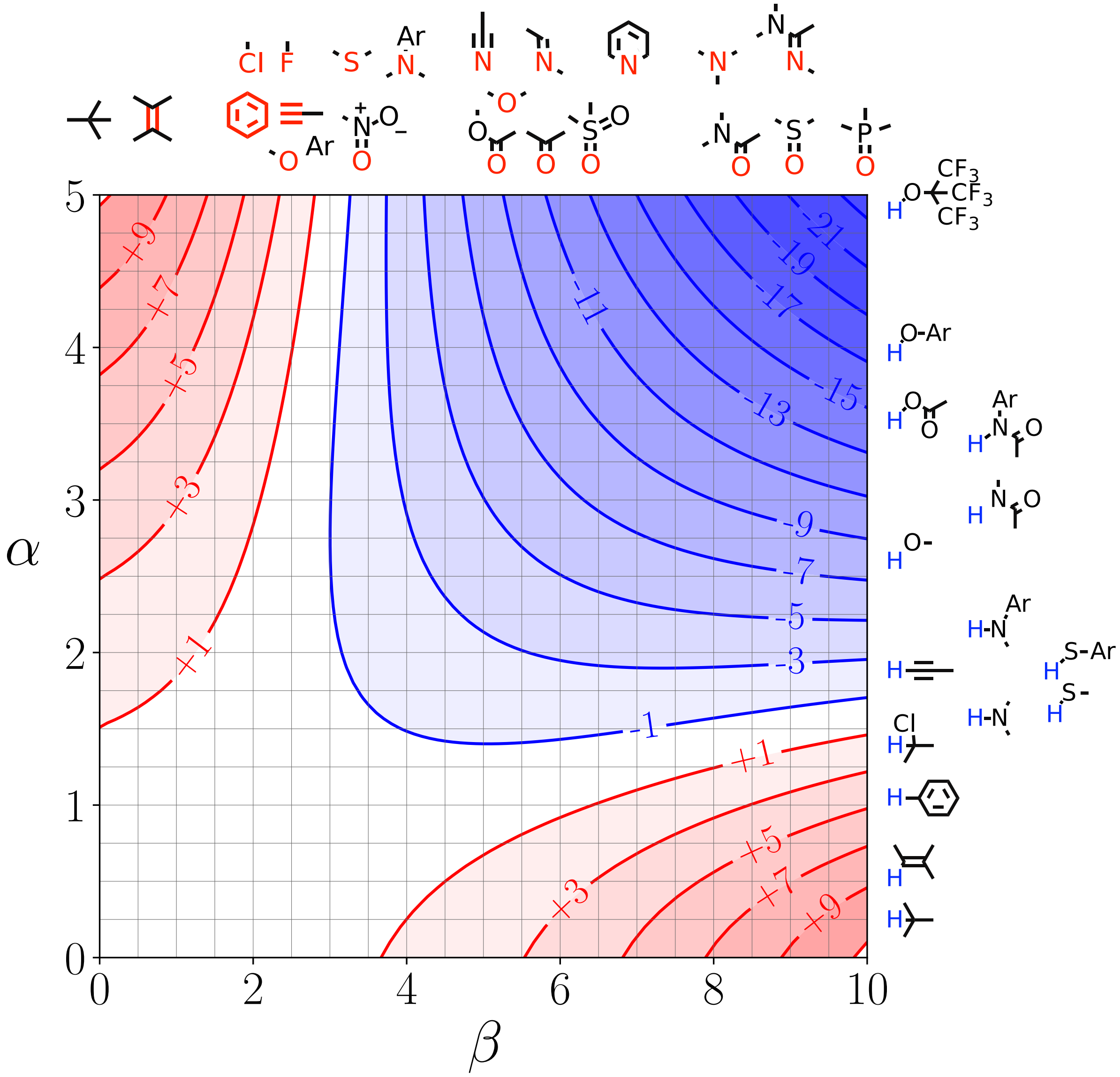

Figure S194: FGIP for N,N-dimethylaniline at 298K.

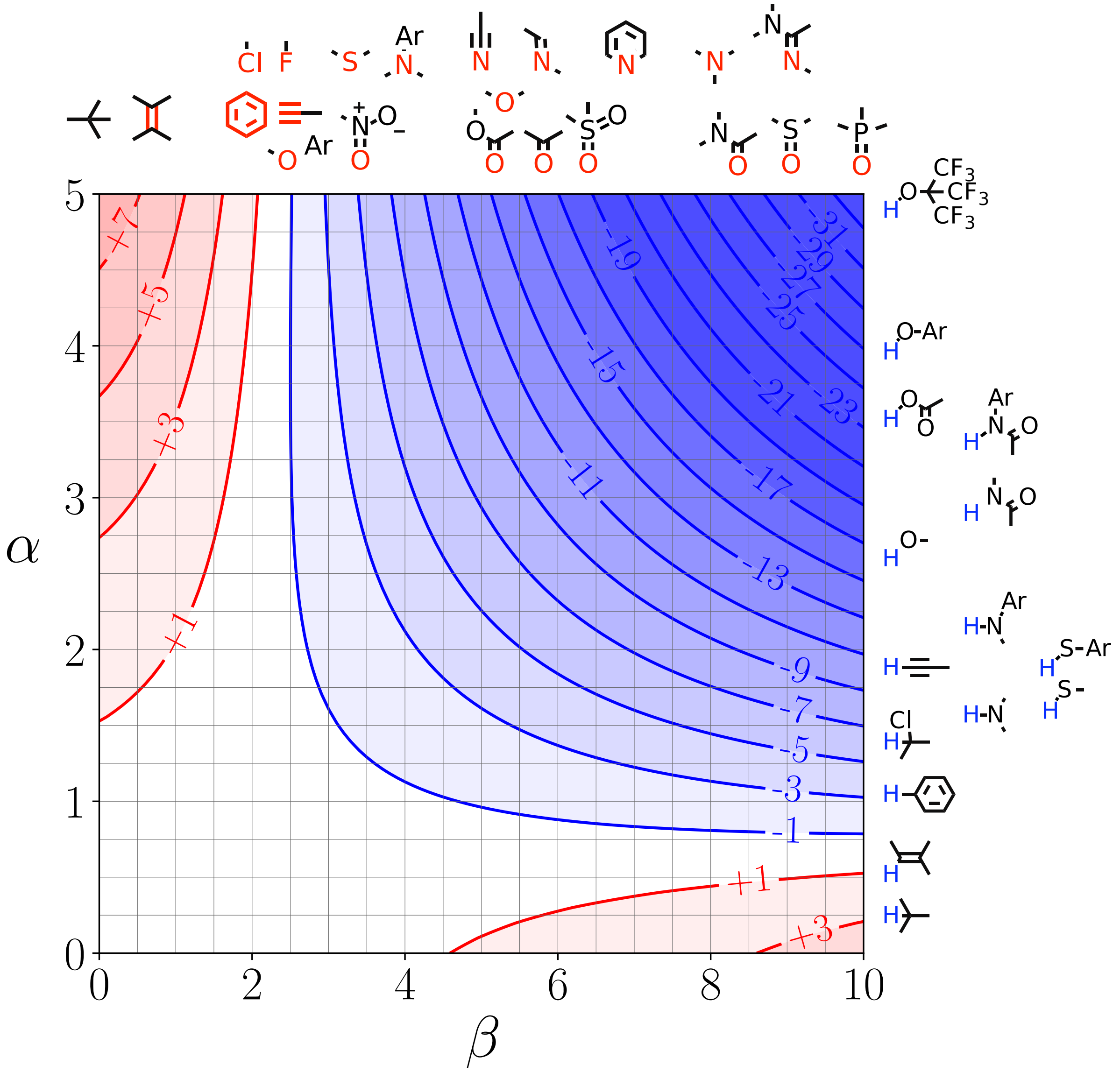

Figure S195: FGIP for aminoethanol at 298K.

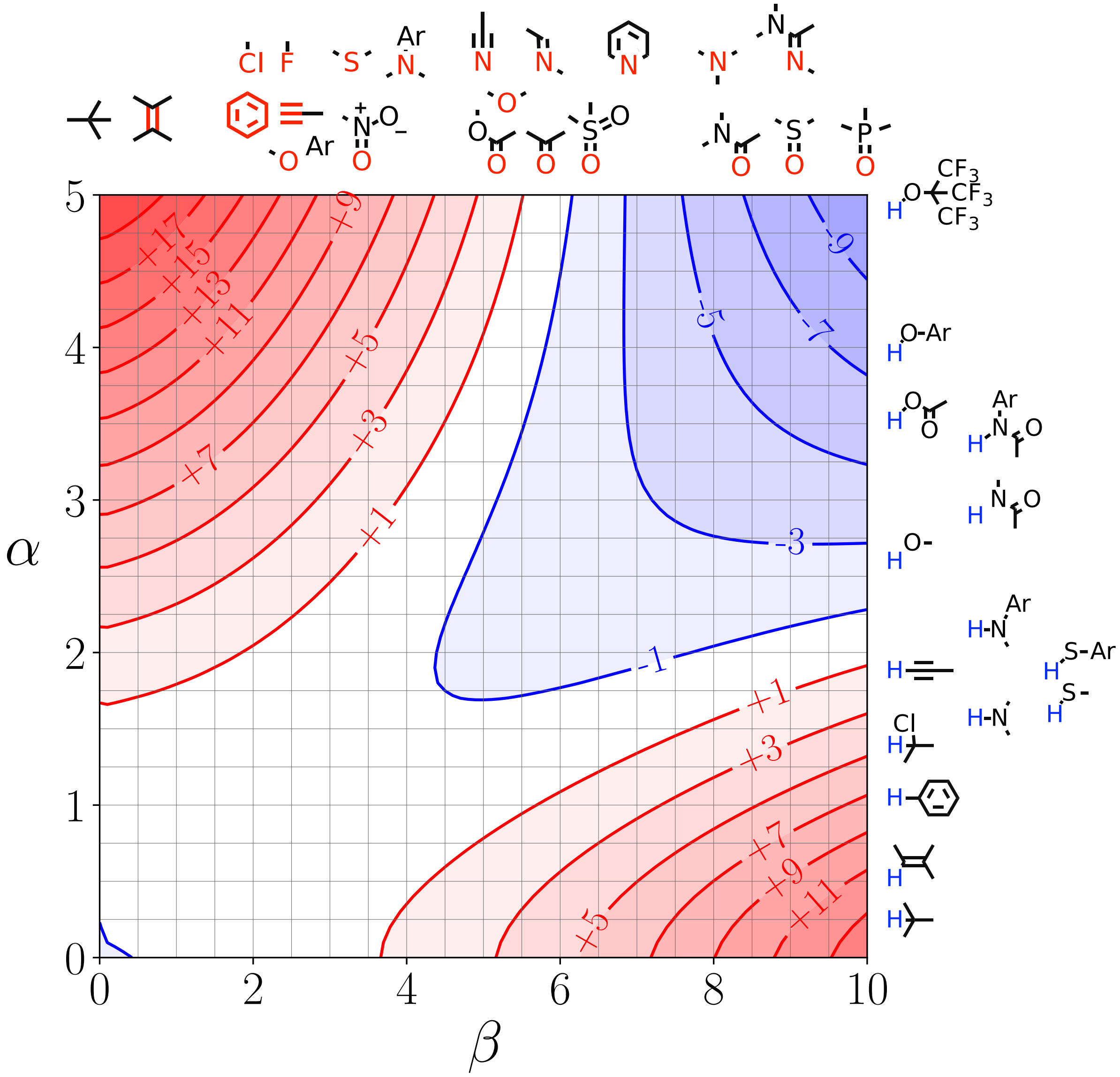

Figure S196: FGIP for diethanolamine at 298K.

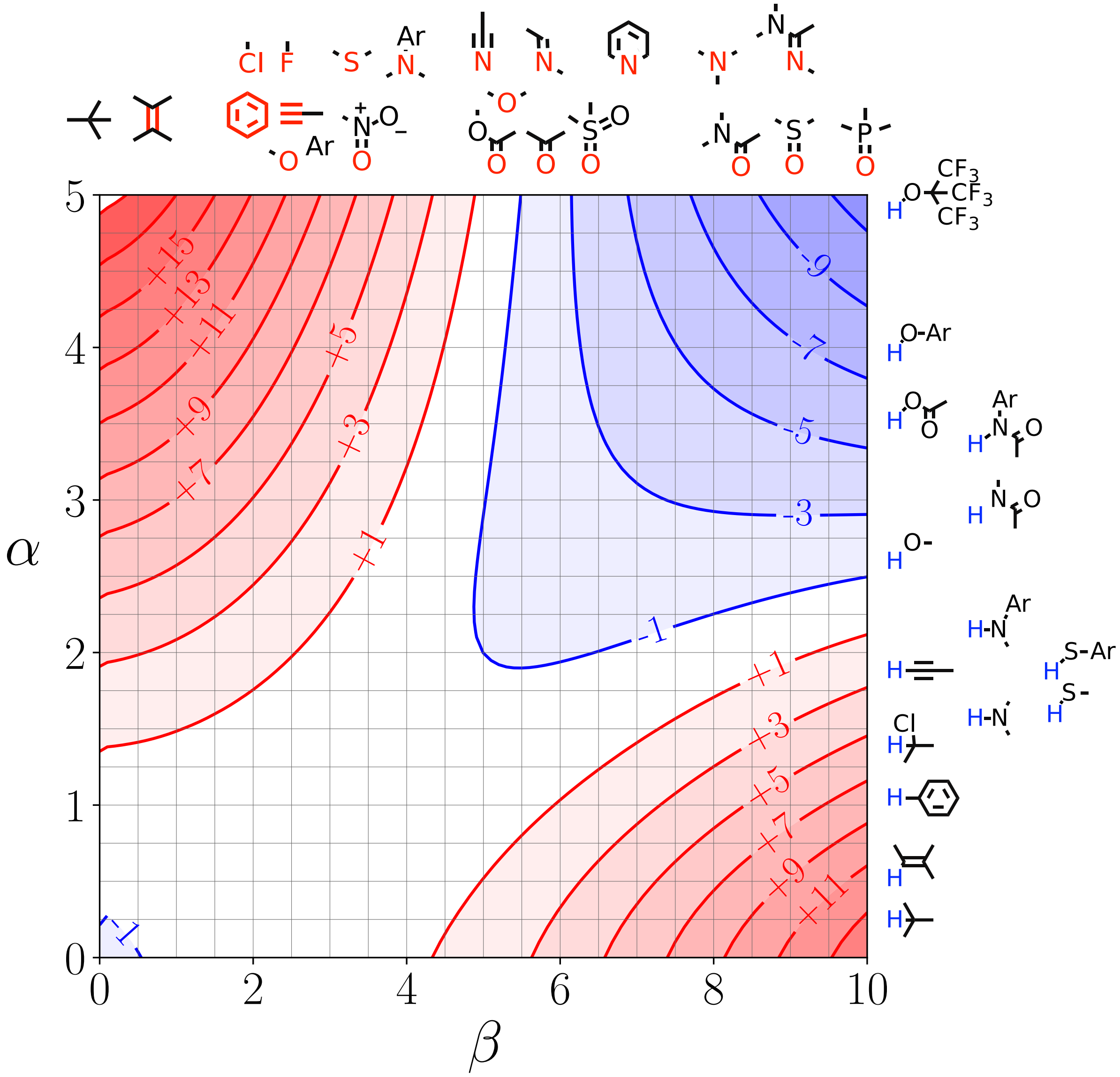

Figure S197: FGIP for triethanolamine at 298K.

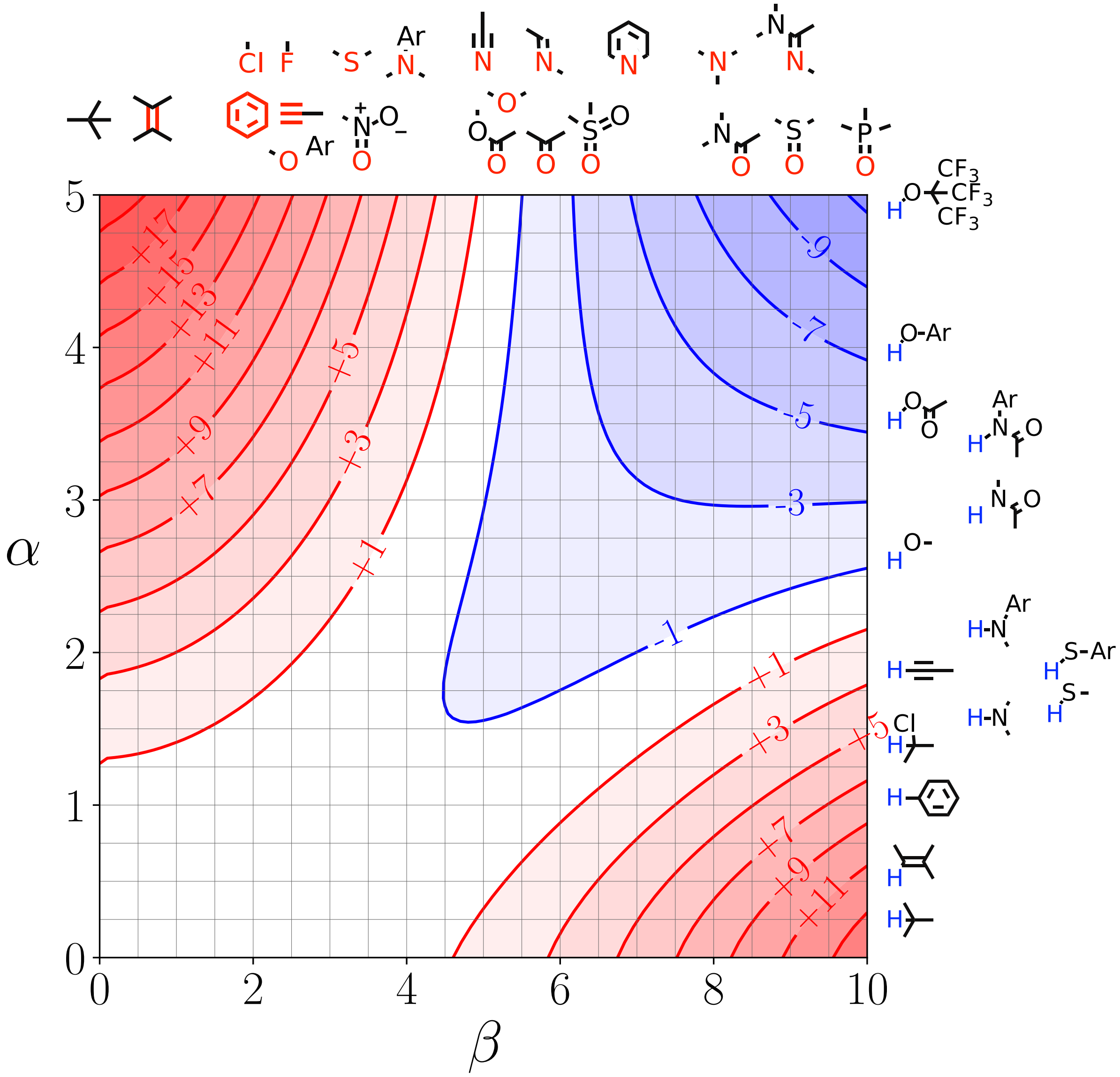

Figure S198: FGIP for pyridine at 298K.

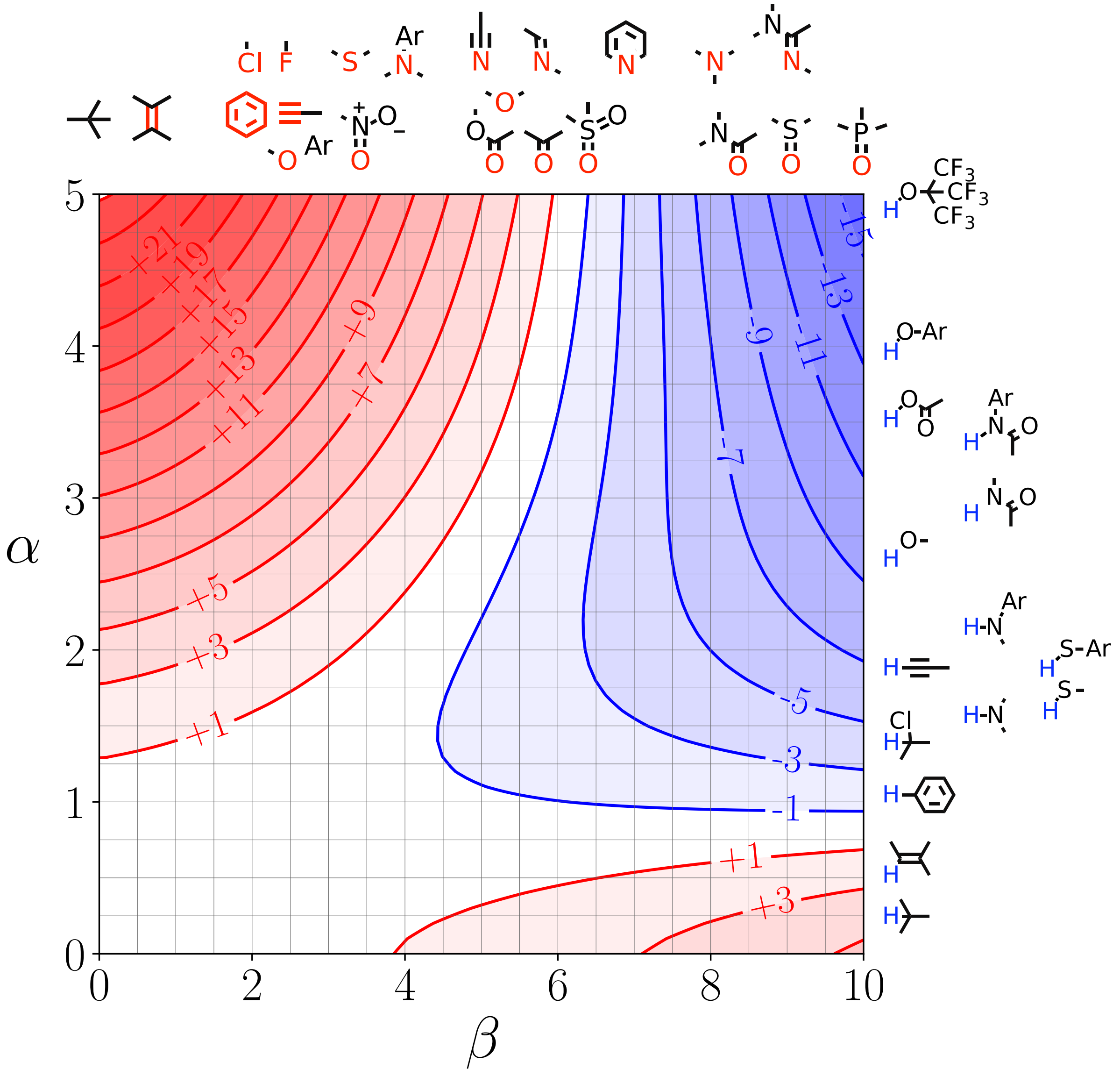

Figure S199: FGIP for 2-methylpyridine at 298K.

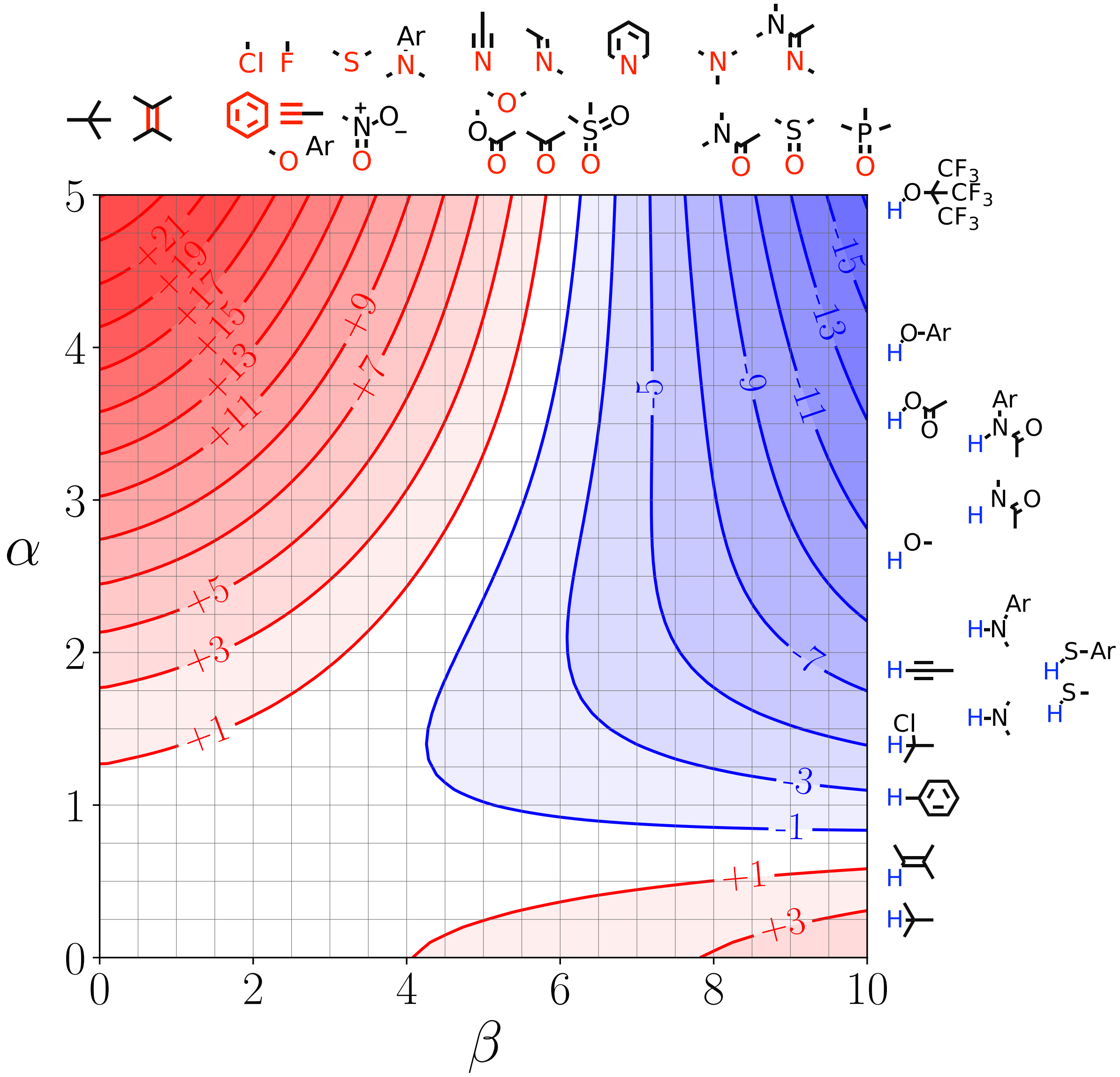

Figure S200: FGIP for 3-methylpyridine at 298K.

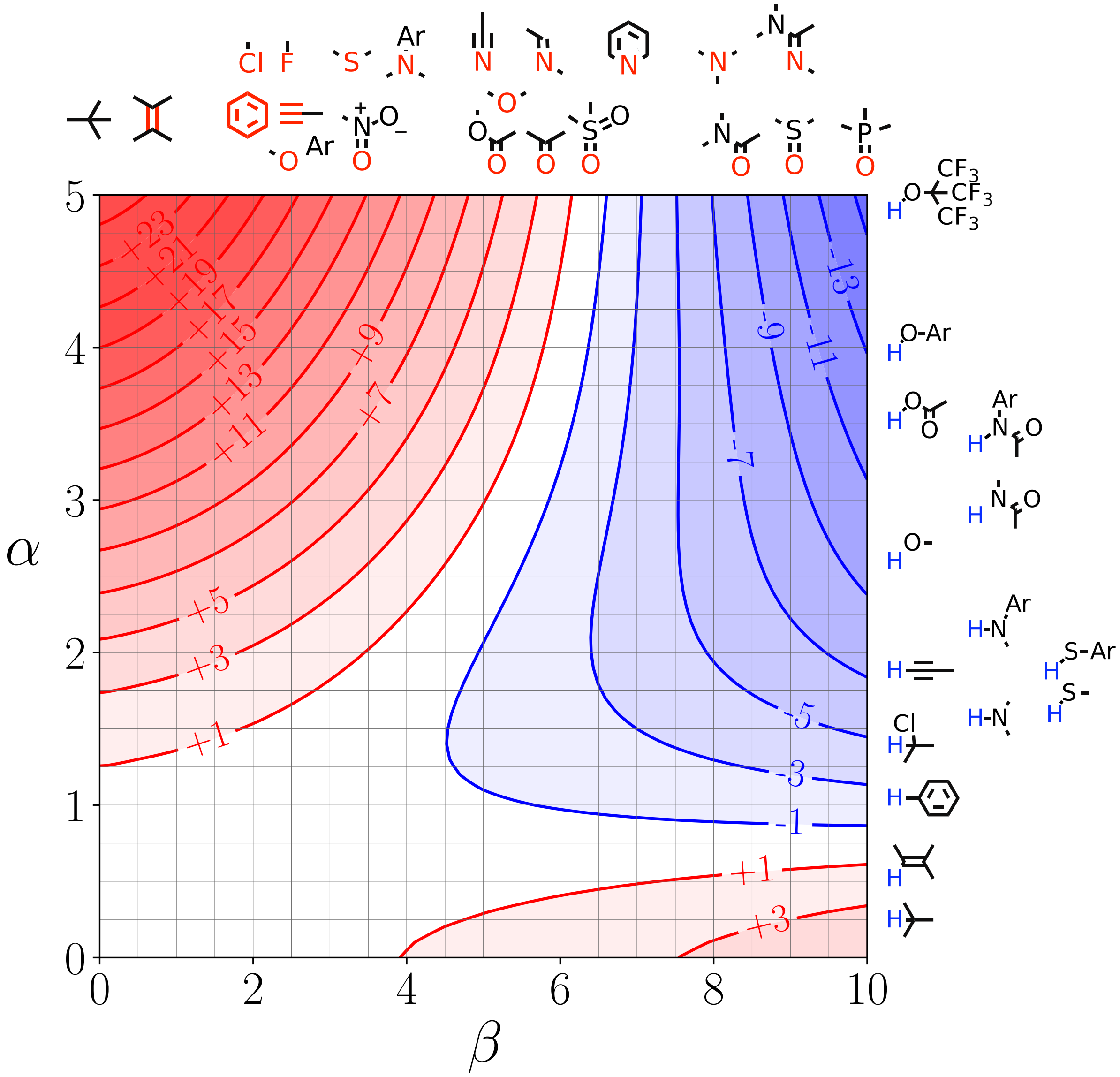

Figure S201: FGIP for 4-methylpyridine at 298K.

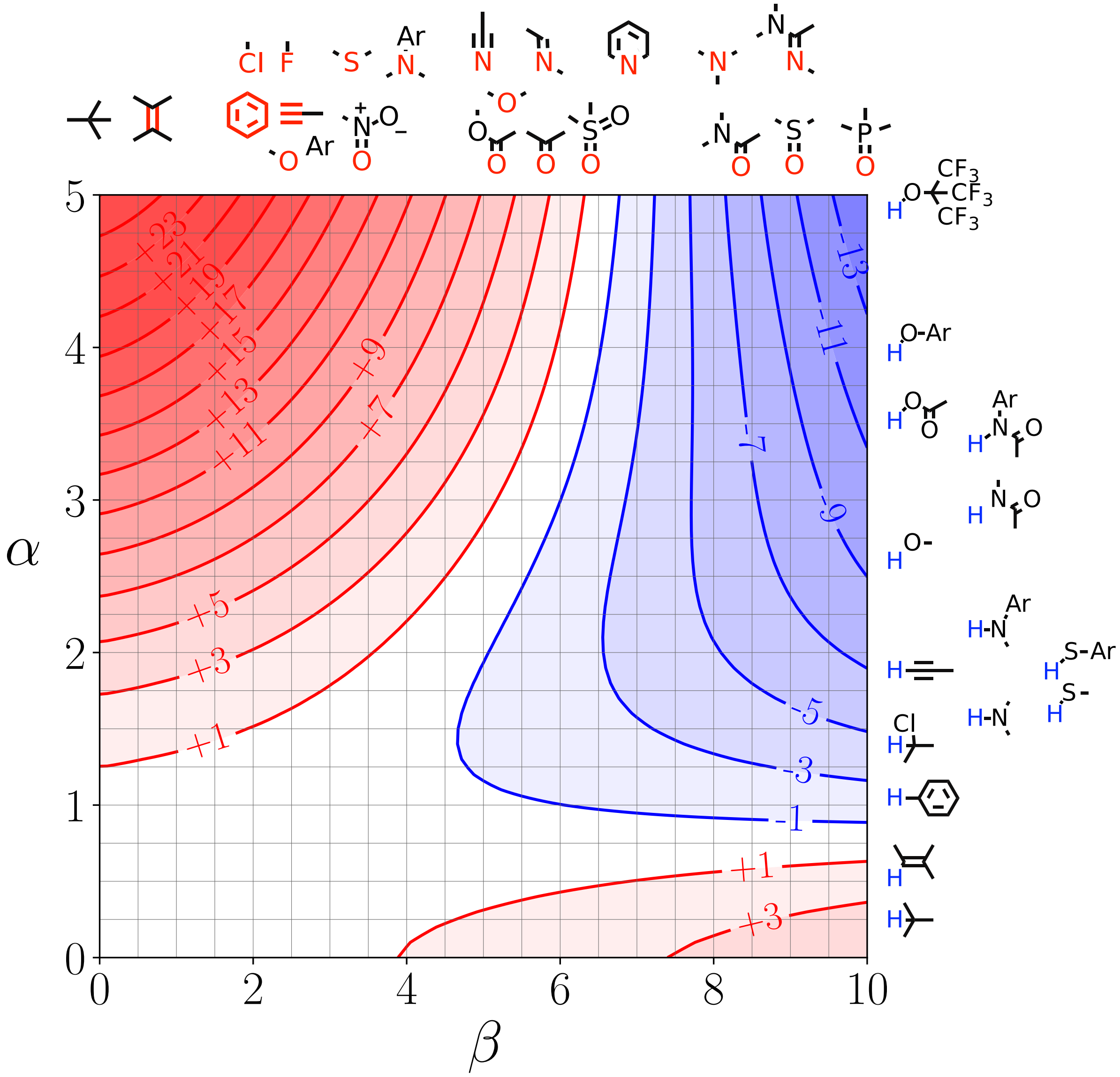

Figure S202: FGIP for 2,4-dimethylpyridine at 298K.

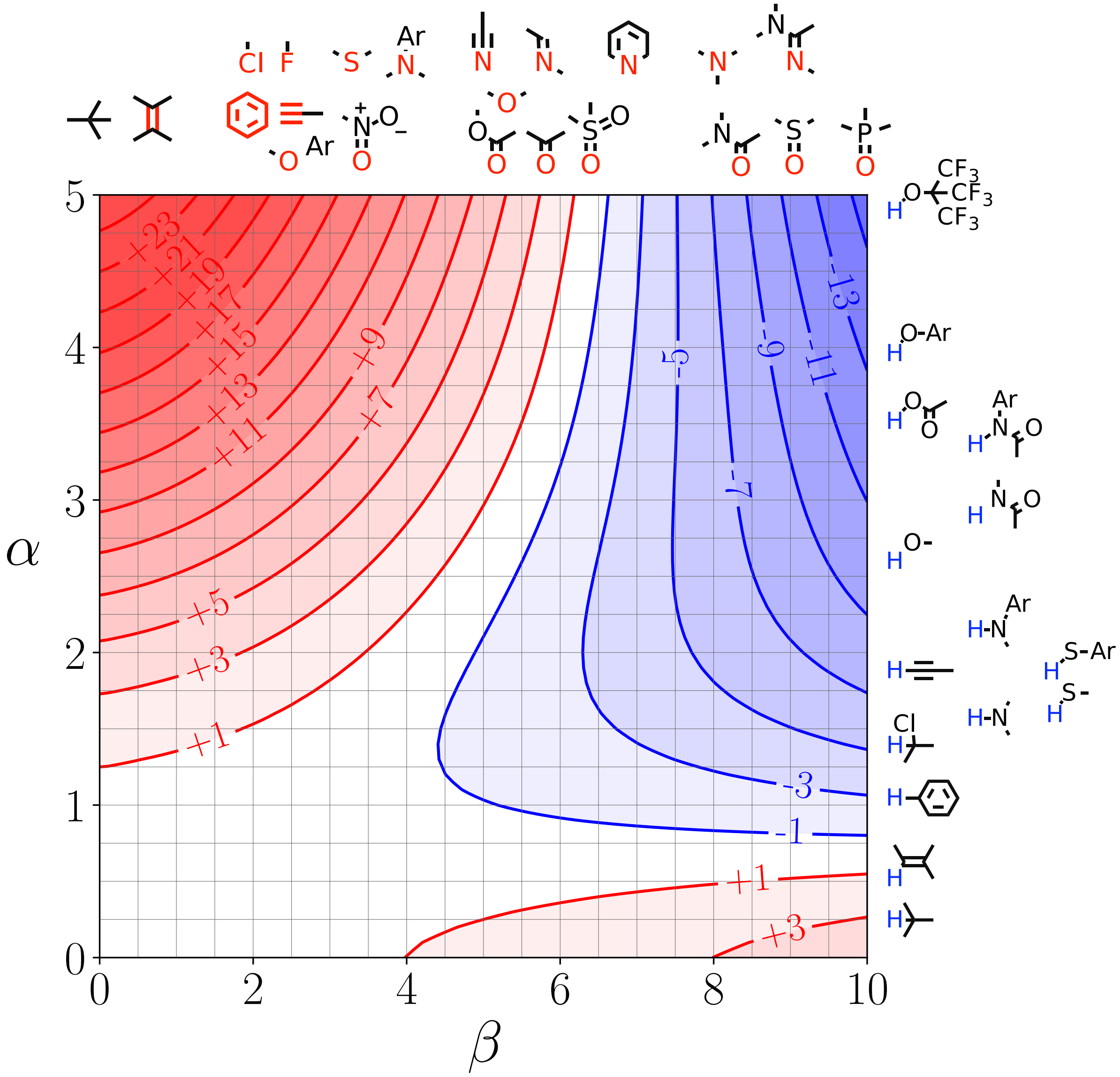

Figure S203: FGIP for 2,6-dimethylpyridine at 298K.

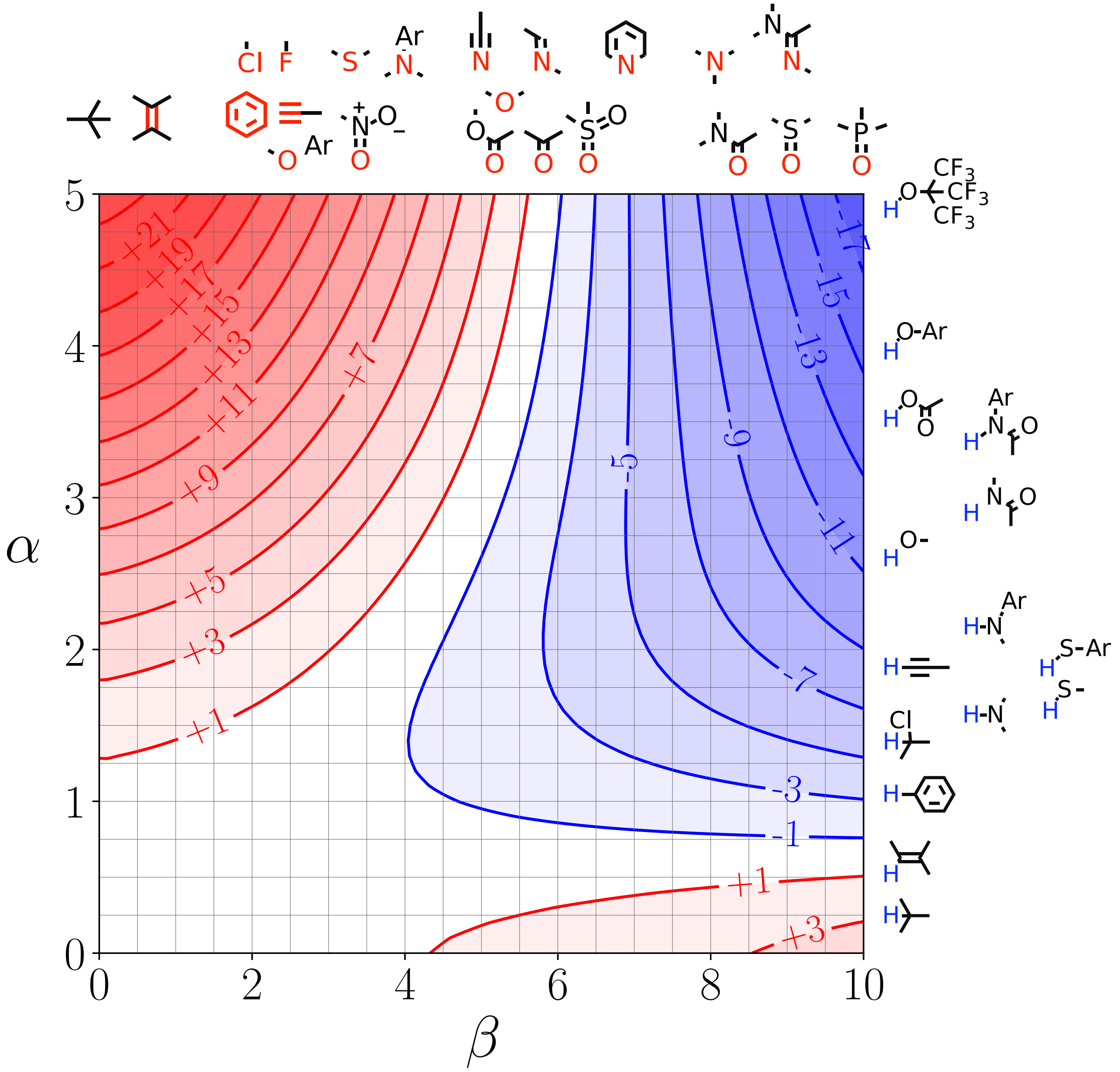

Figure S204: FGIP for 2,4,6-trimethylpyridine at 298K.

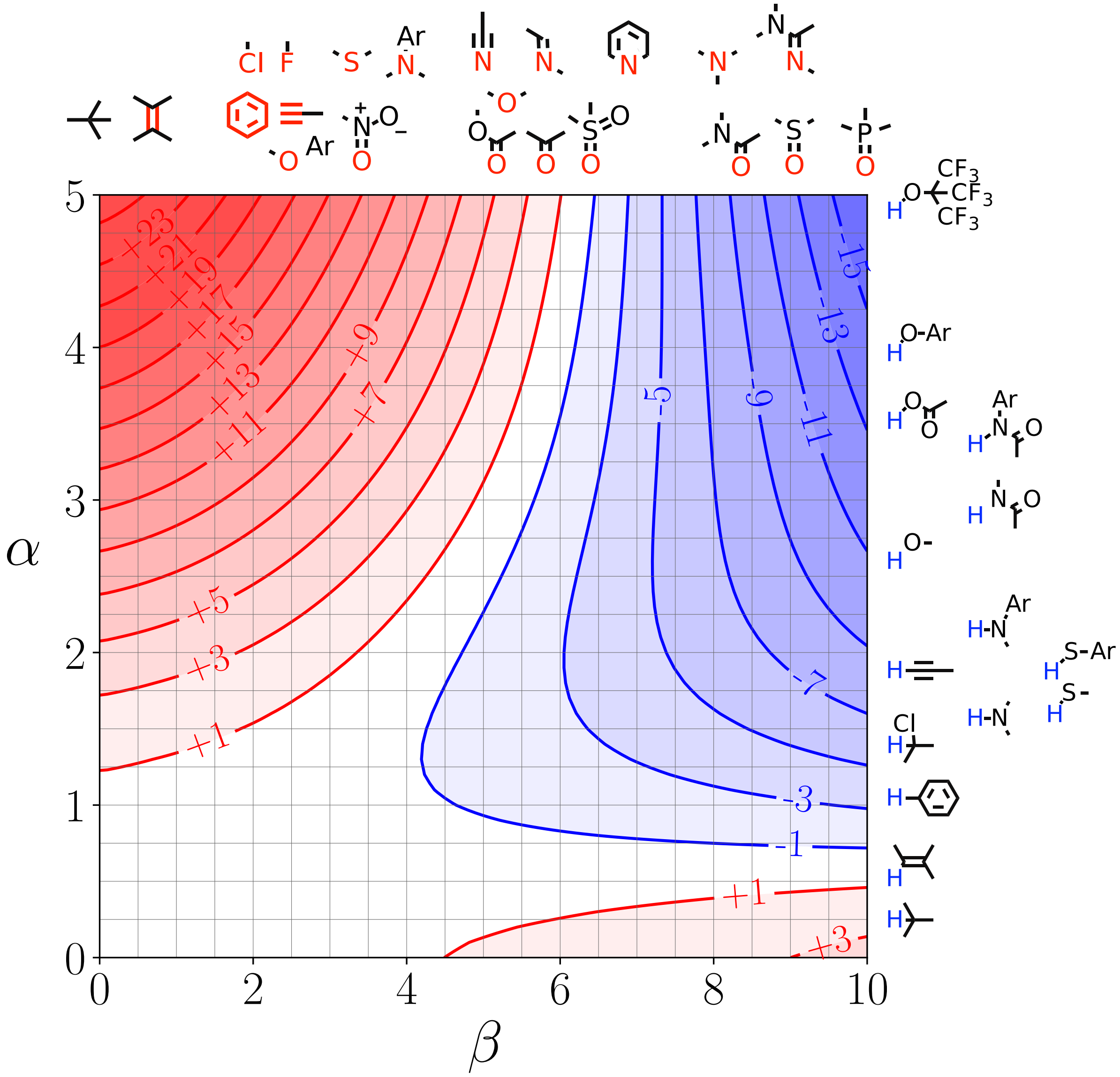

Figure S205: FGIP for 2-bromopyridine at 298K.

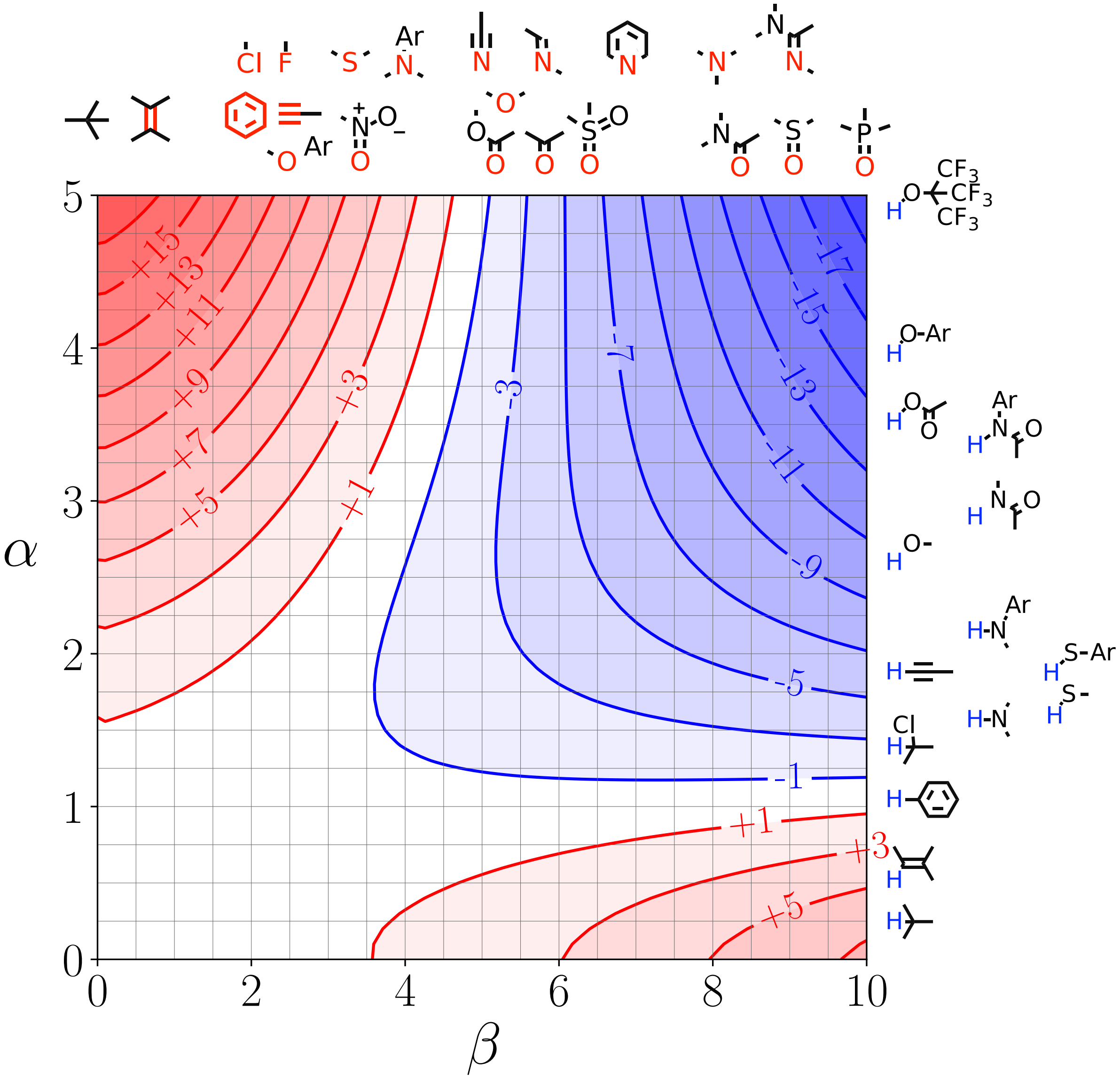

Figure S206: FGIP for 3-bromopyridine at 298K.

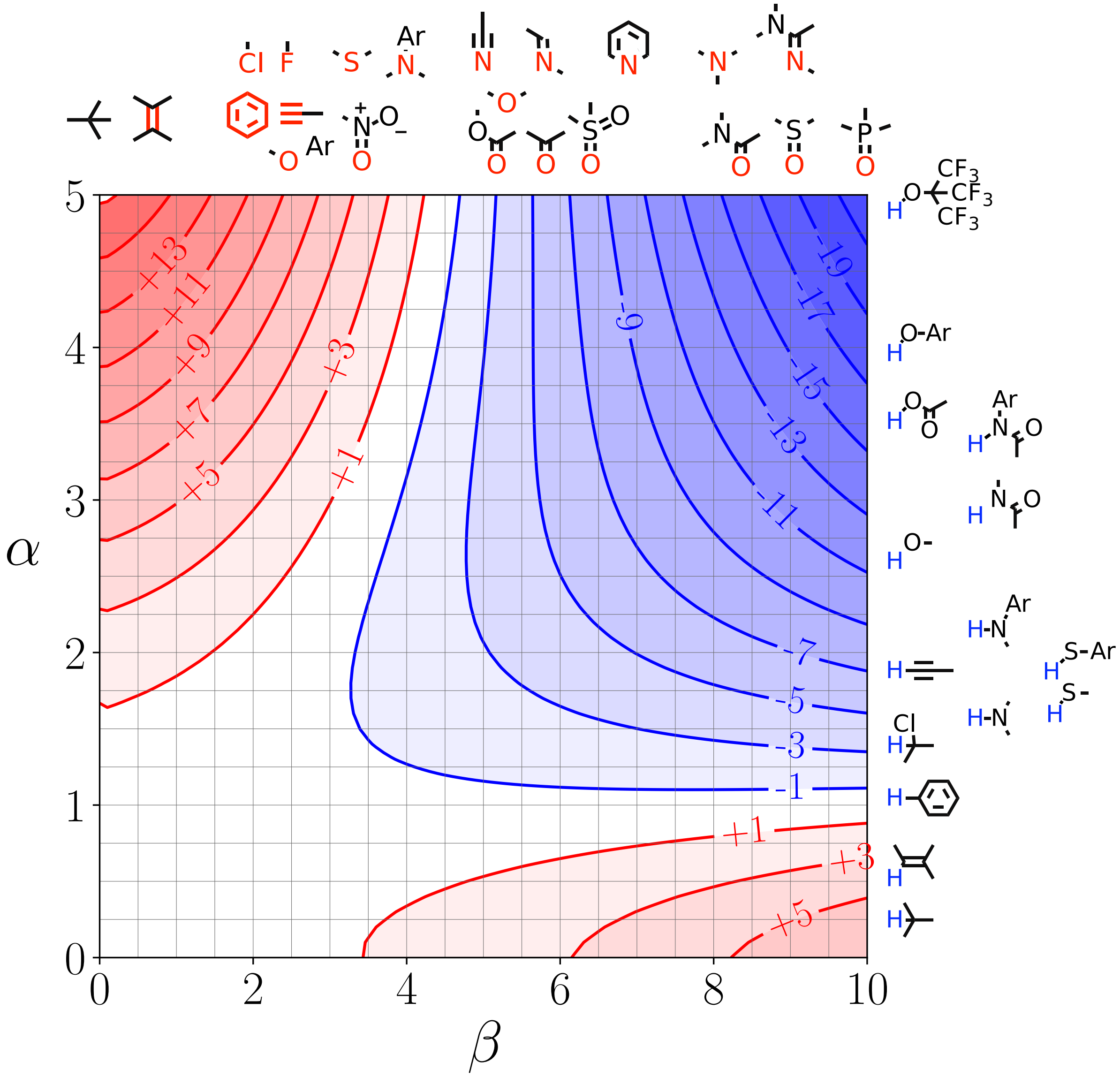

Figure S207: FGIP for 2-cyanopyridine at 298K.

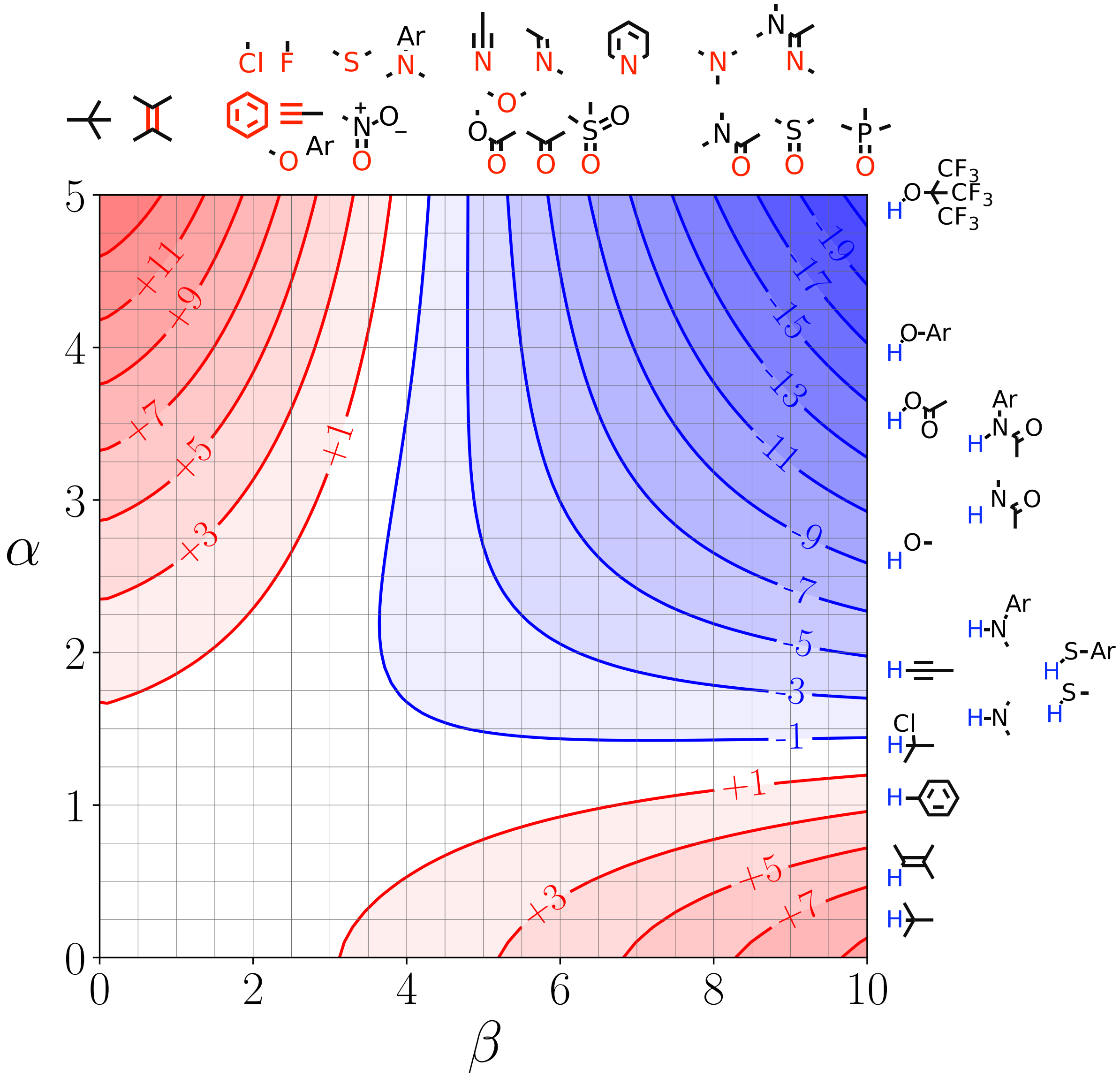

Figure S208: FGIP for pyrimidine at 298K.

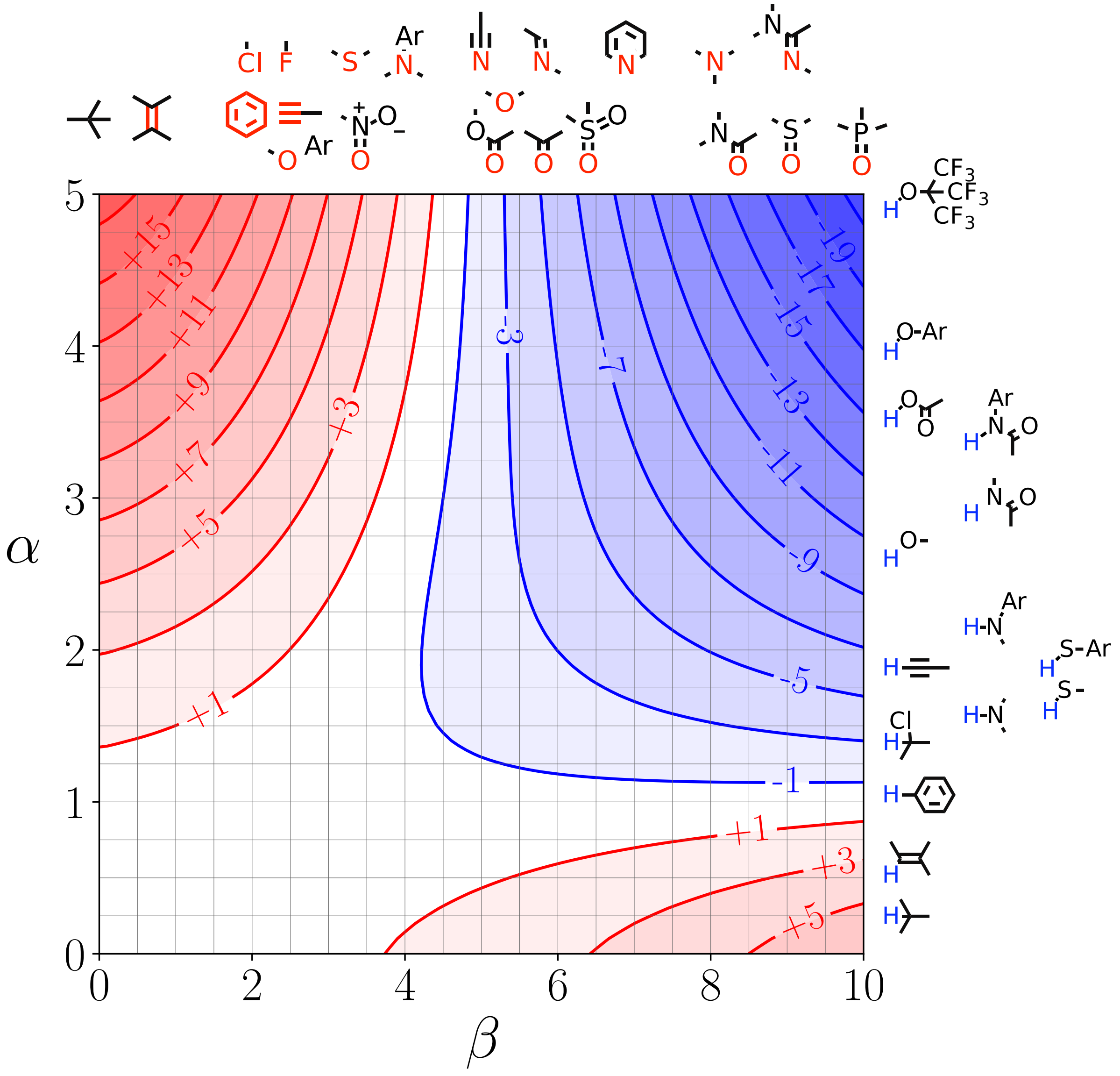

Figure S209: FGIP for quinoline at 298K.

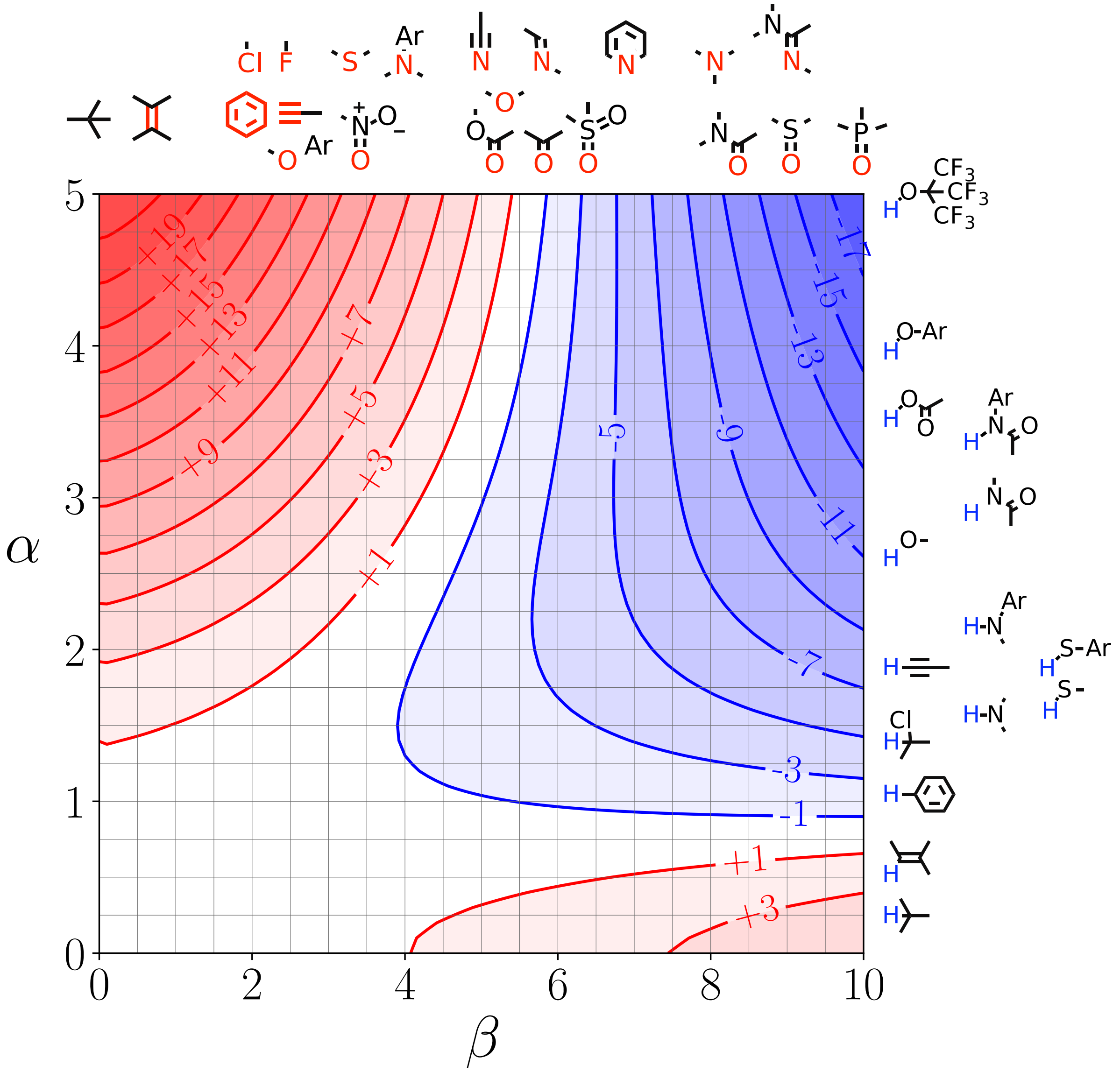

Figure S210: FGIP for acetonitrile at 298K.

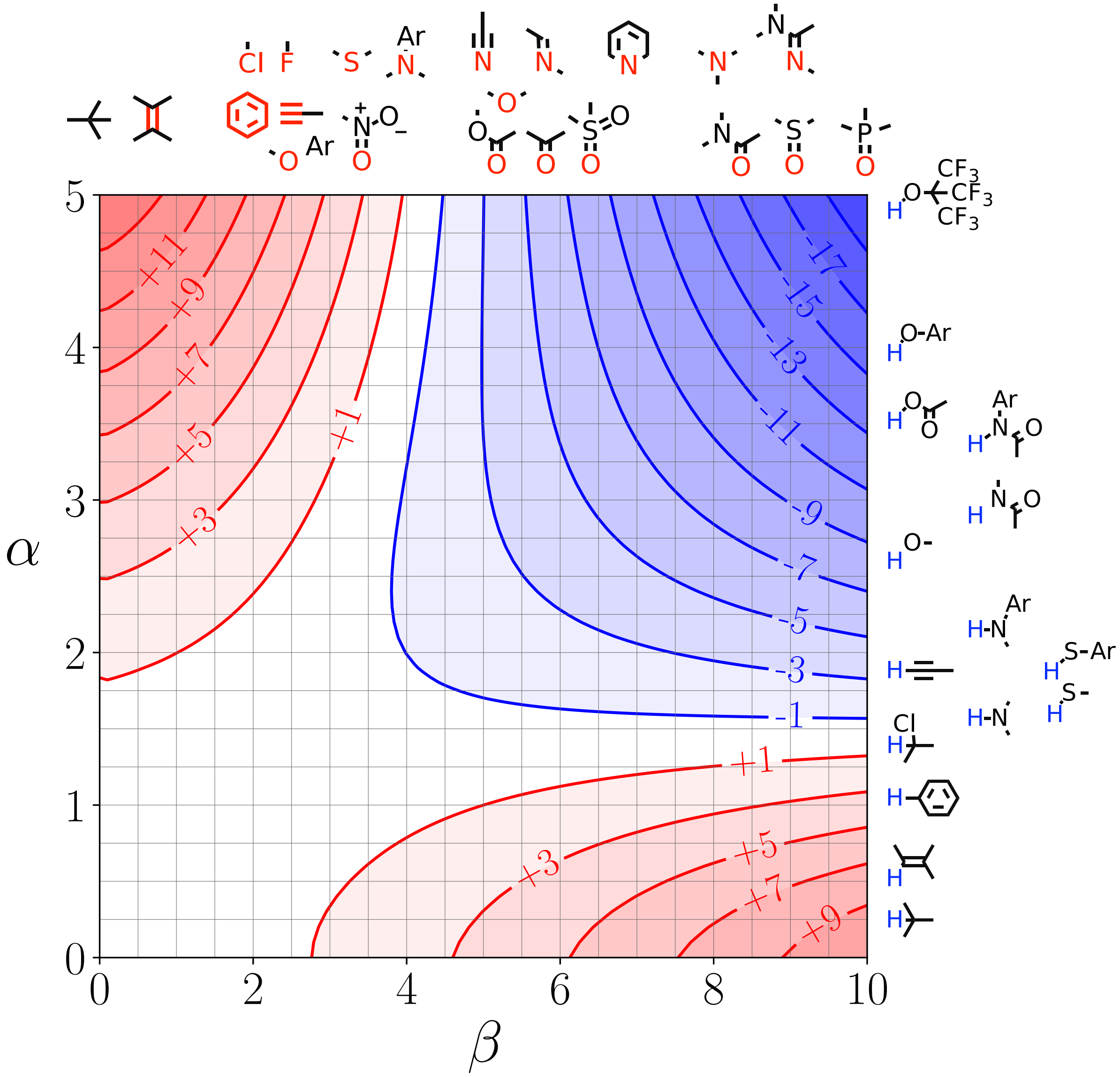

Figure S211: FGIP for propionitrile at 298K.

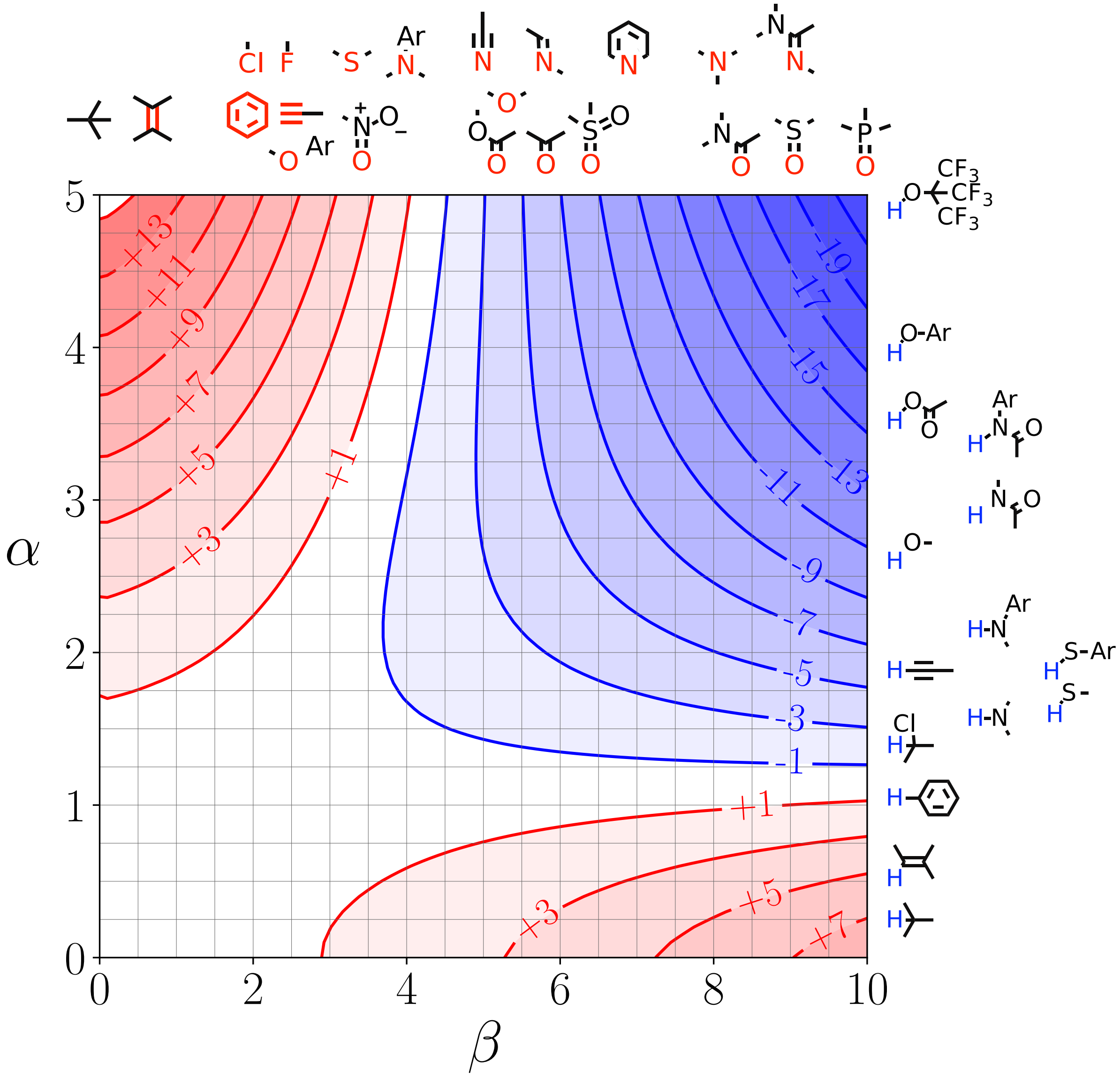

Figure S212: FGIP for n-butyronitrile at 298K.

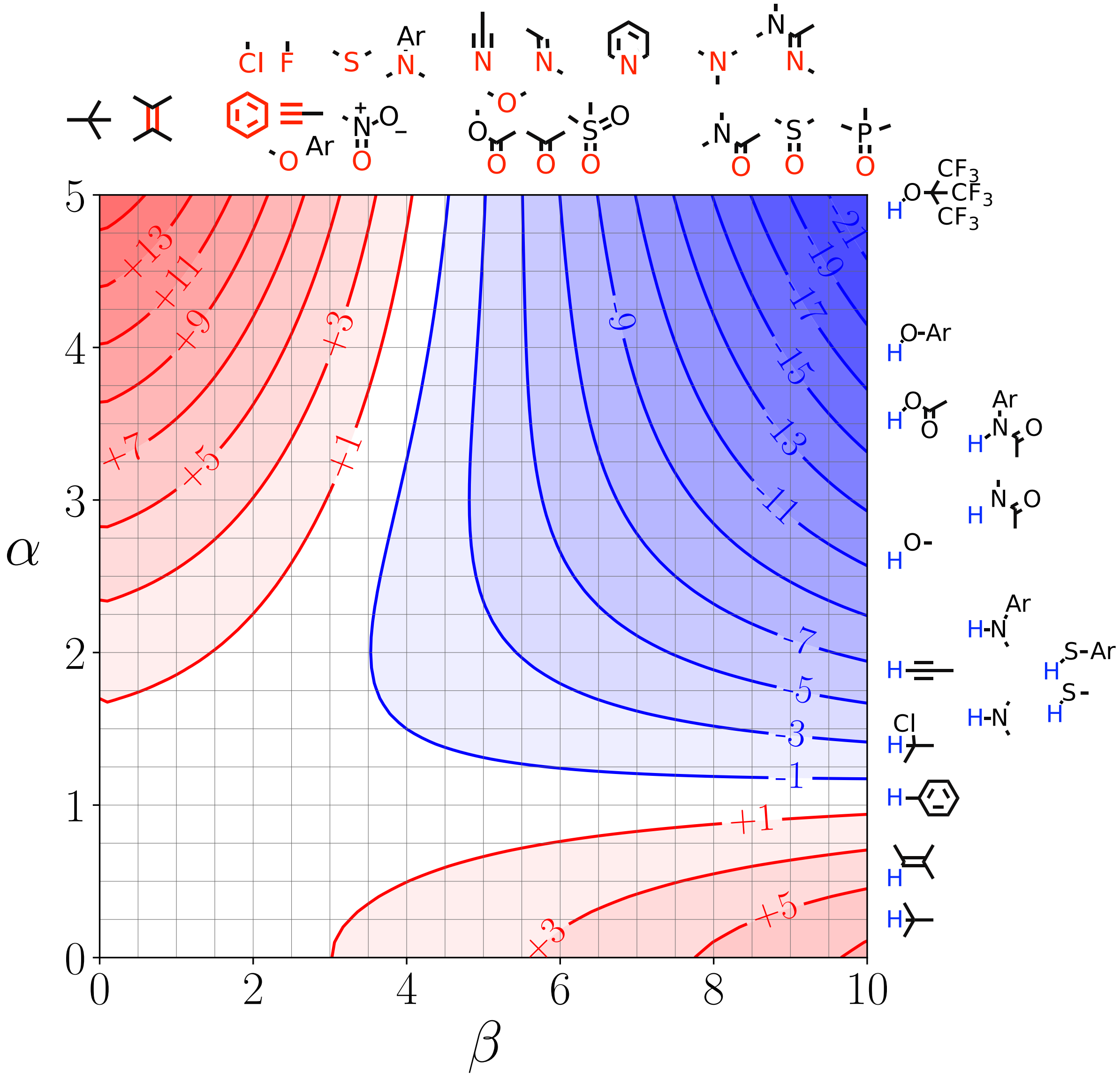

Figure S213: FGIP for 3-methylbutanenitrile at 298K.

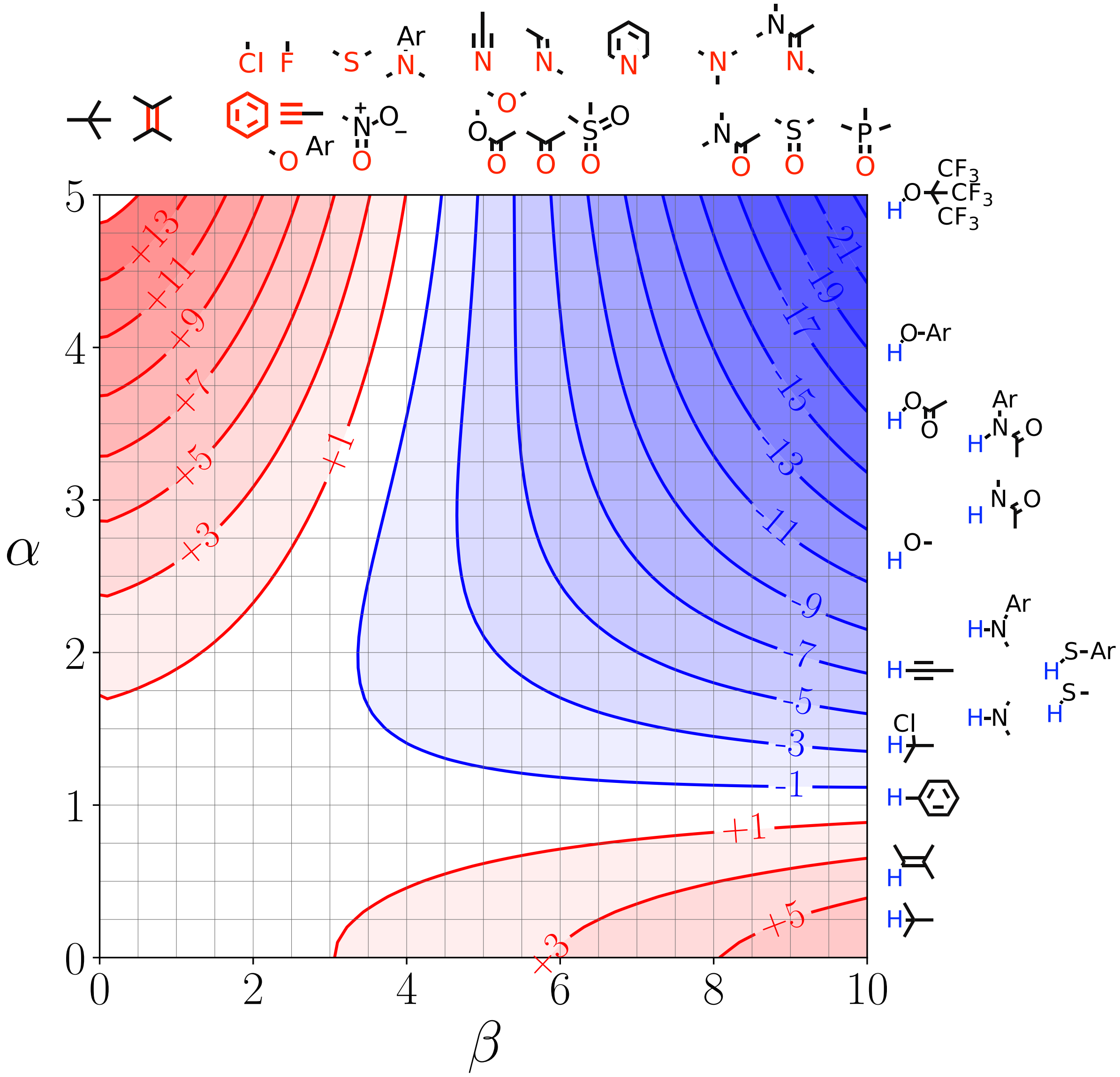

Figure S214: FGIP for acrylonitrile at 298K.

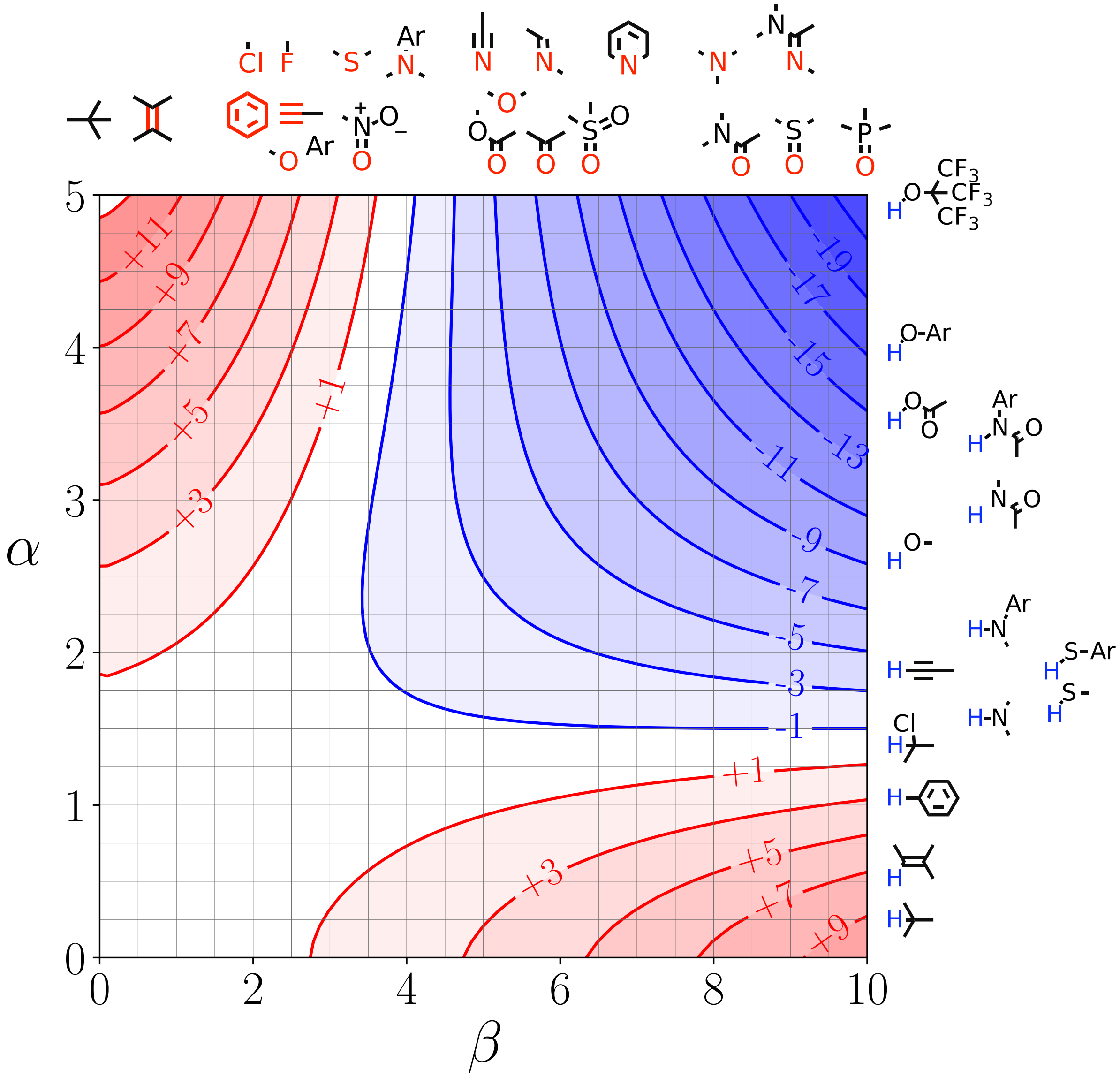

Figure S215: FGIP for phenylacetonitrile at 298K.

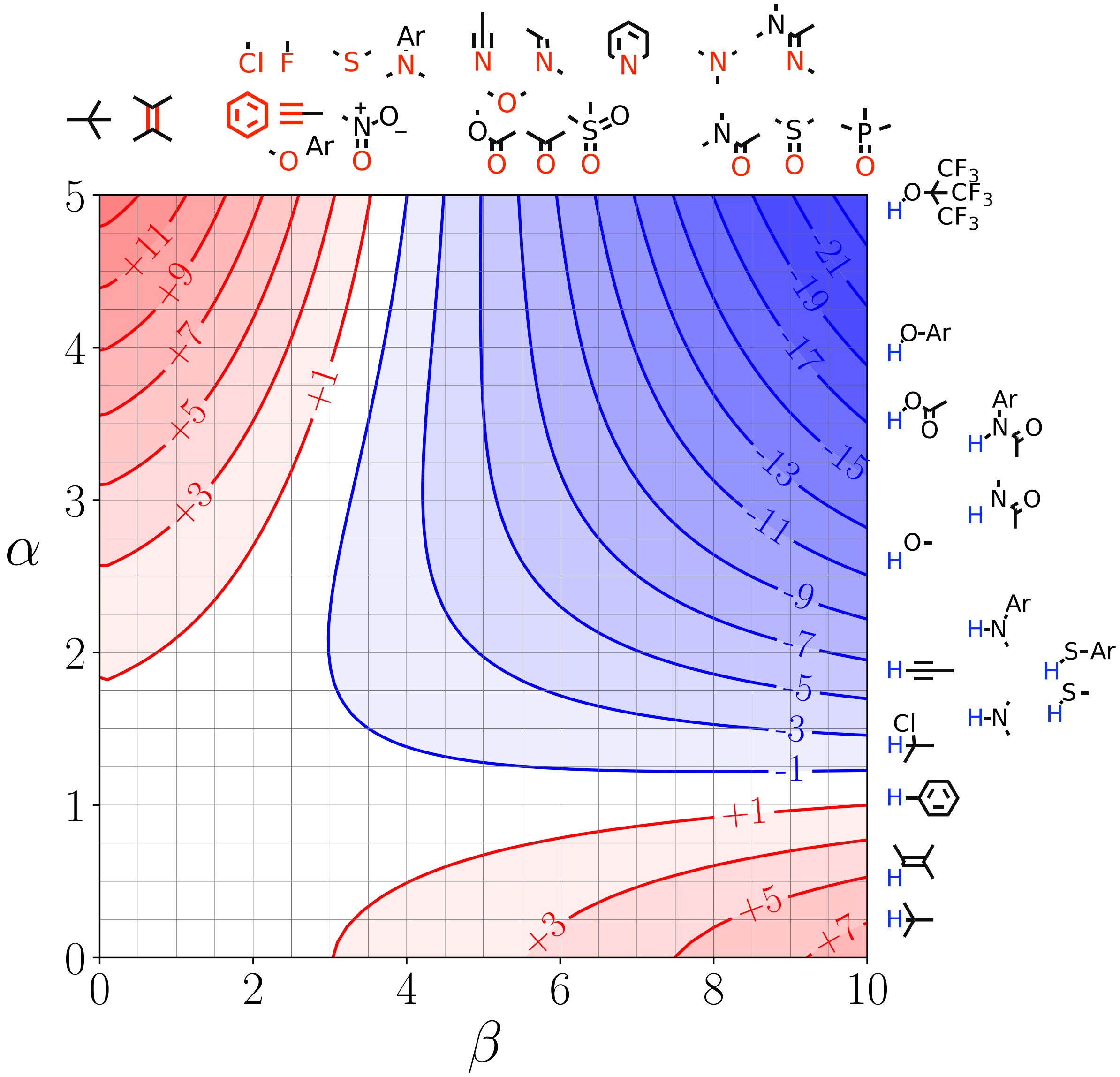

Figure S216: FGIP for benzonitrile at 298K.

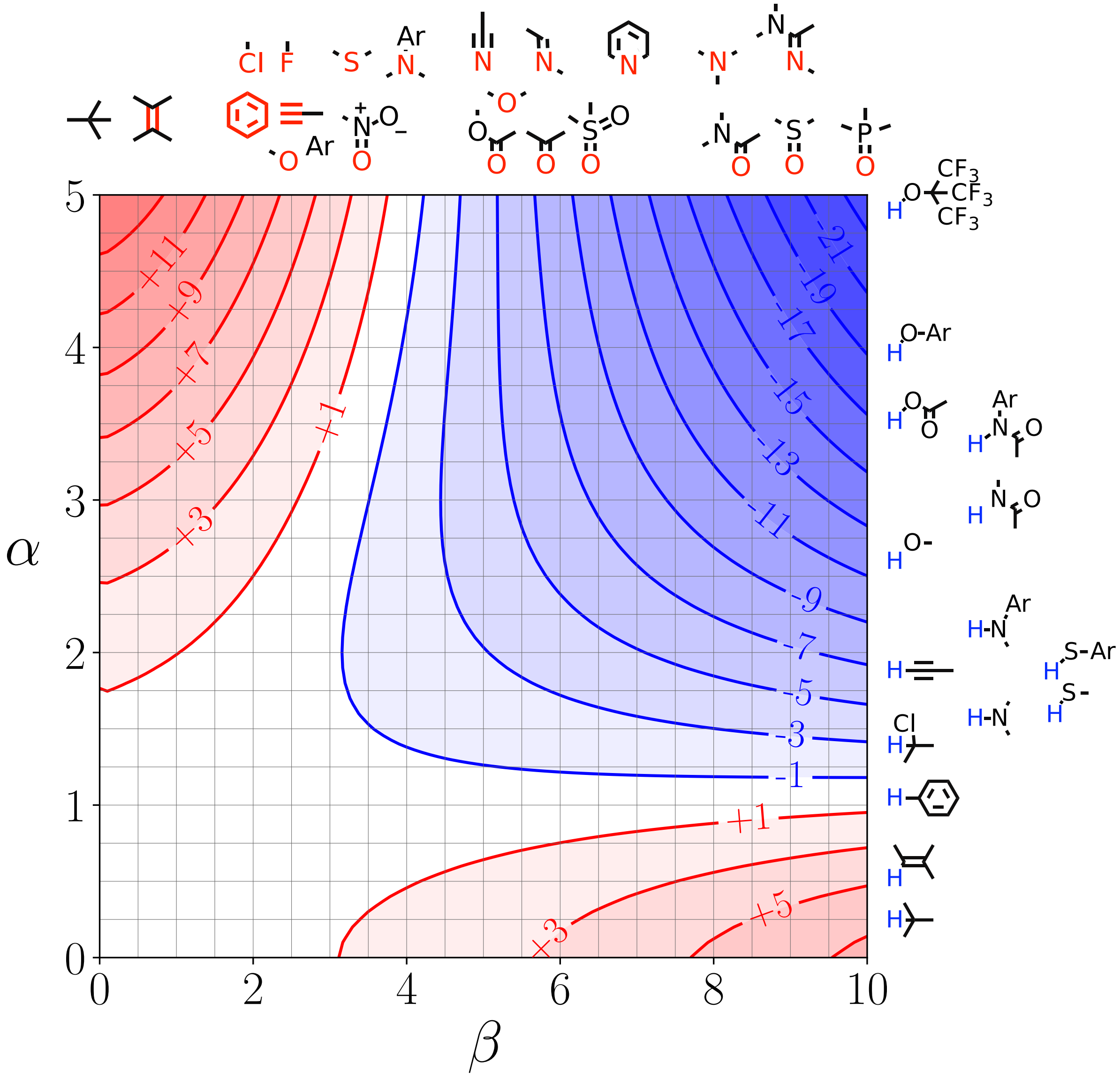

Figure S217: FGIP for nitromethane at 298K.

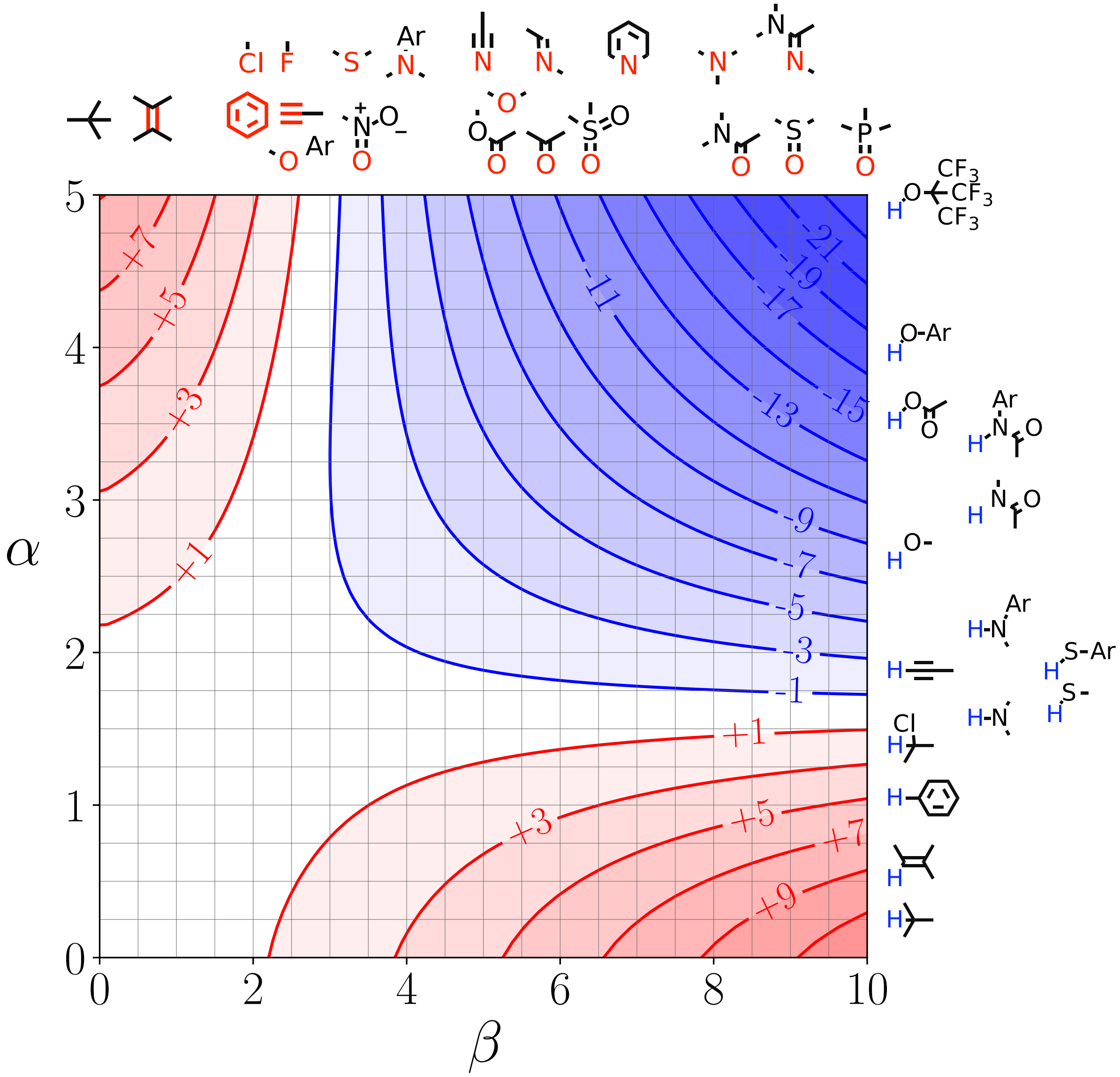

Figure S218: FGIP for nitroethane at 298K.

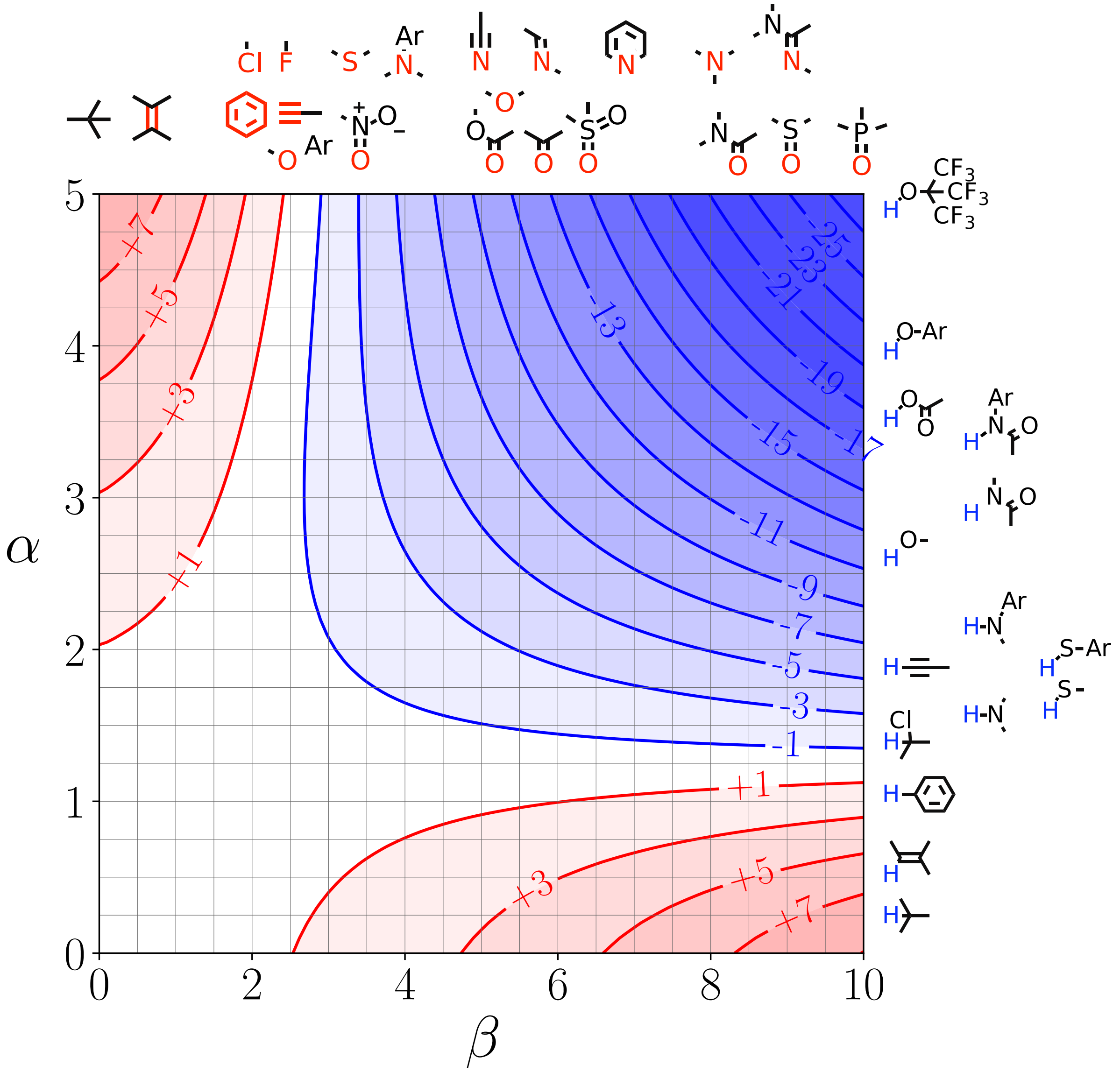

Figure S219: FGIP for 1-nitropropane at 298K.

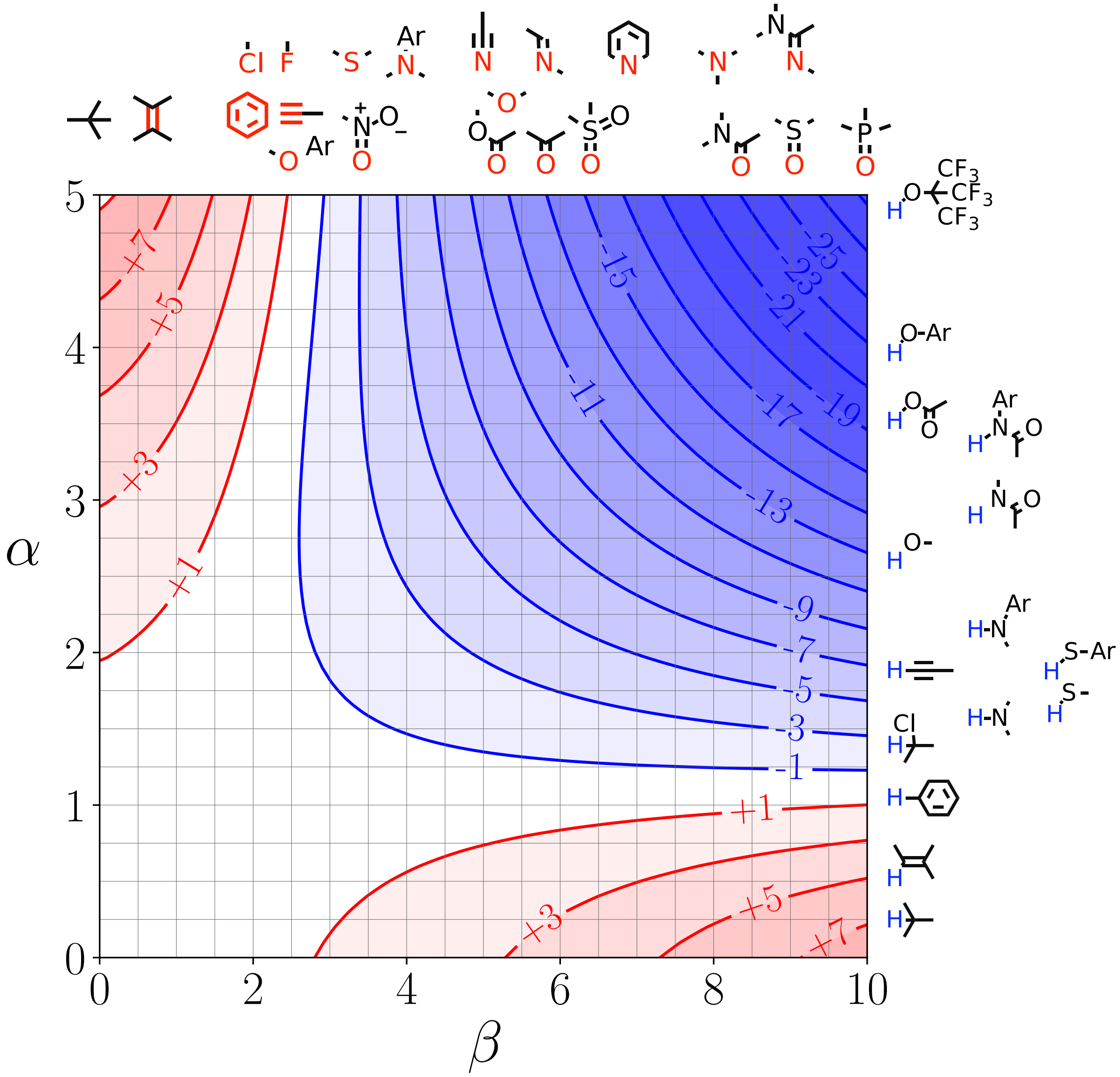

Figure S220: FGIP for 2-nitropropane at 298K.

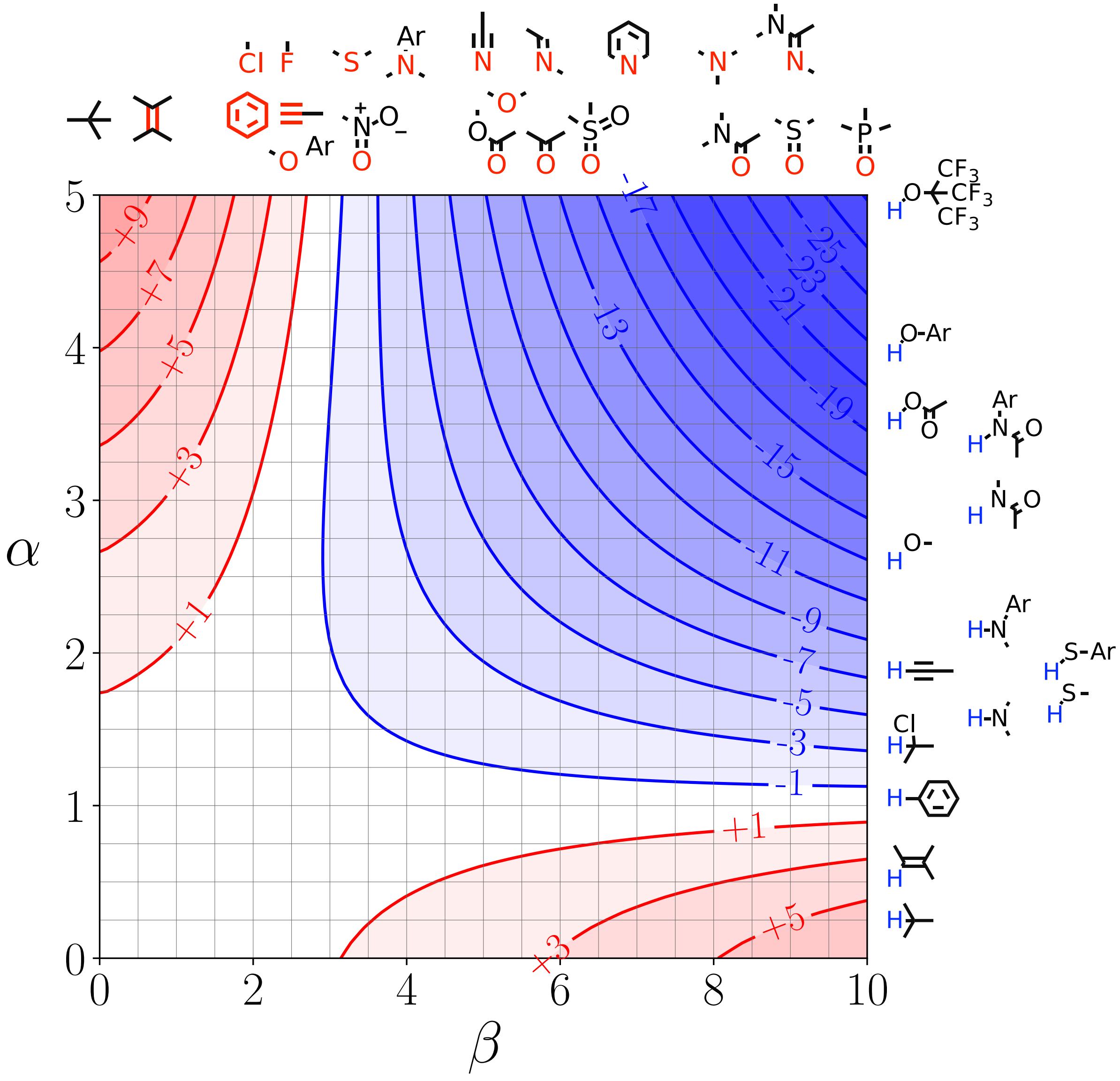

Figure S221: FGIP for nitrobenzene at 298K.

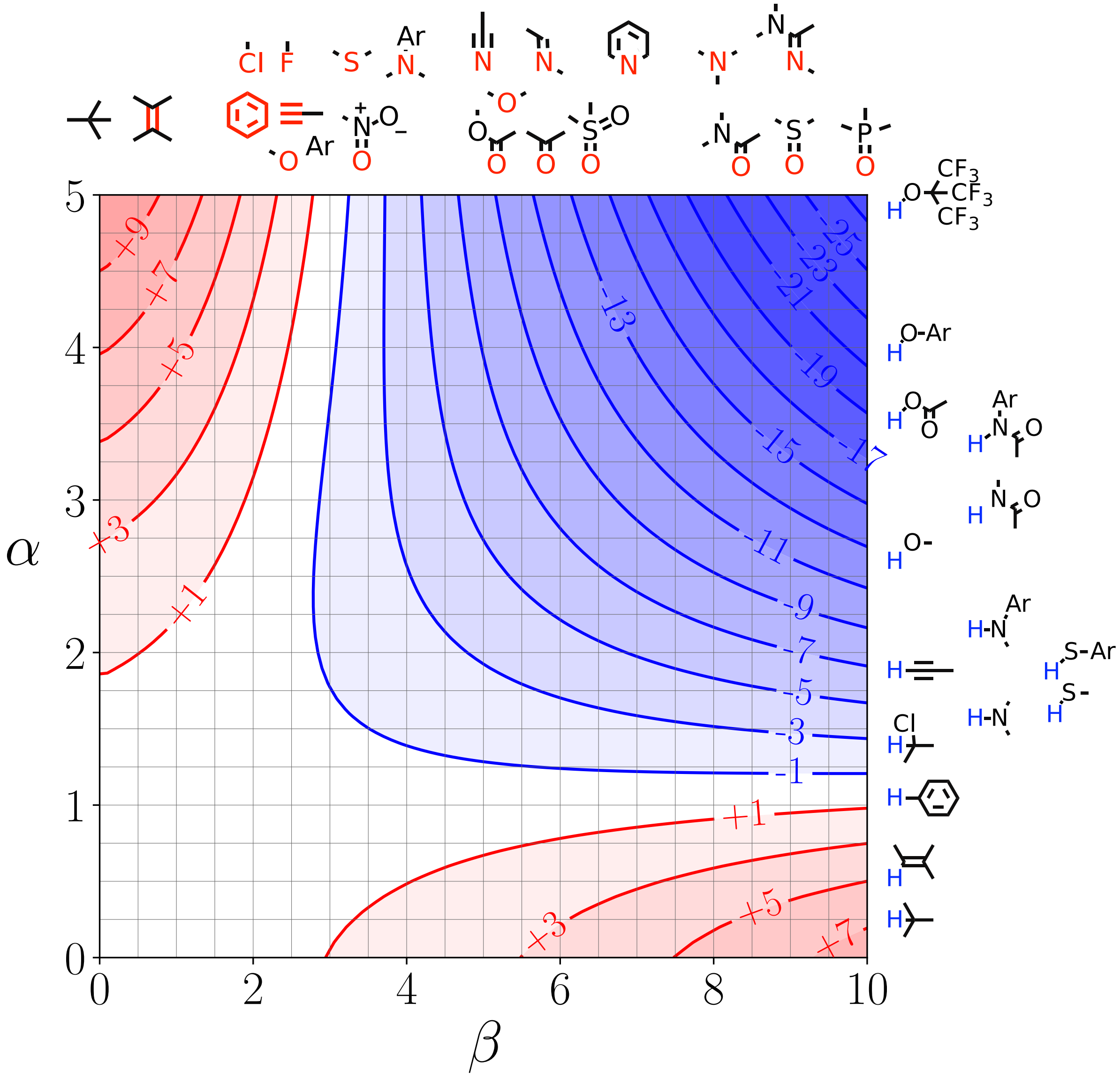

Figure S222: FGIP for formamide at 298K.

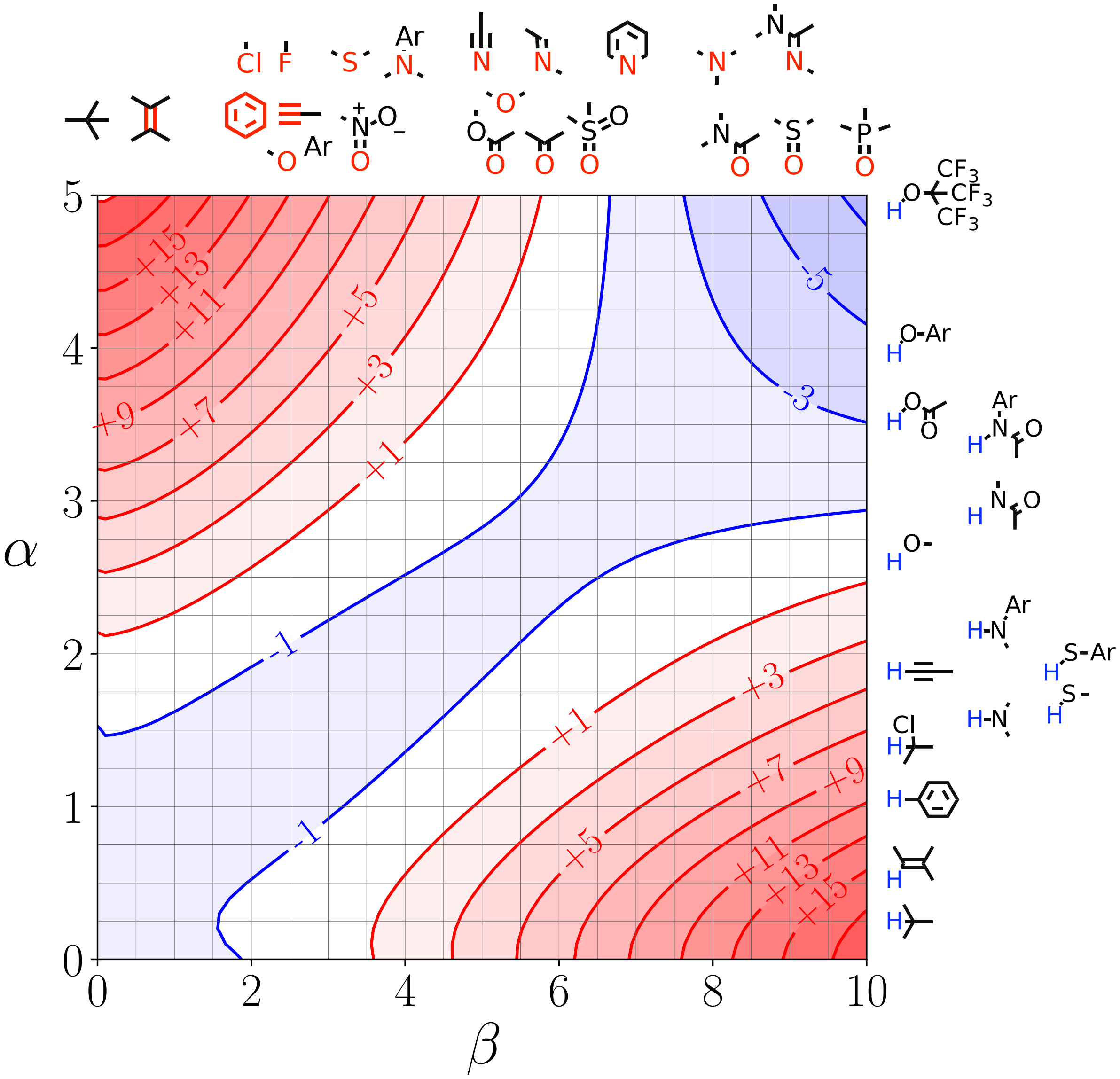

Figure S223: FGIP for N-methylformamide at 298K.

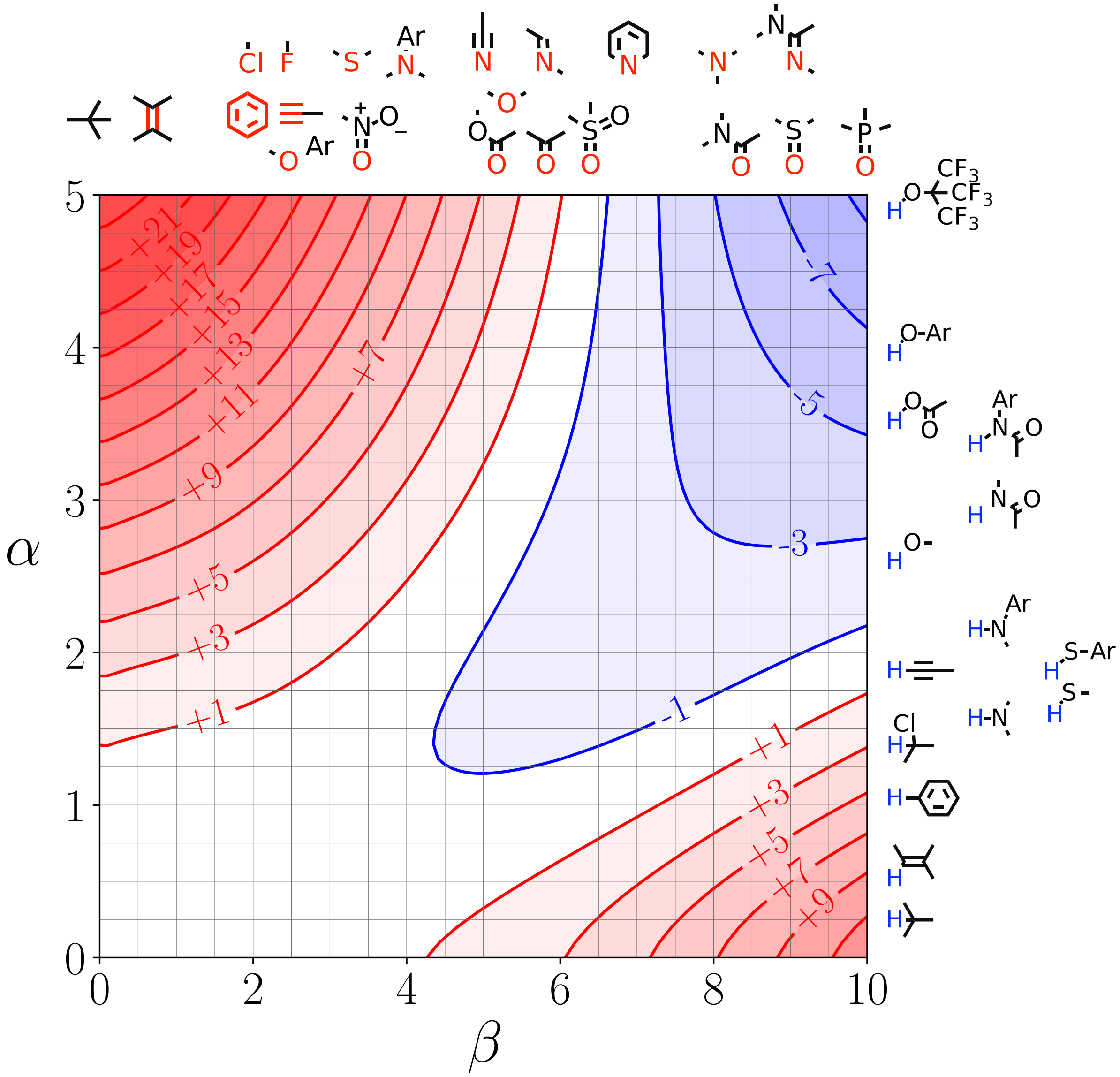

Figure S224: FGIP for N,N-dimethylformamide at 298K.

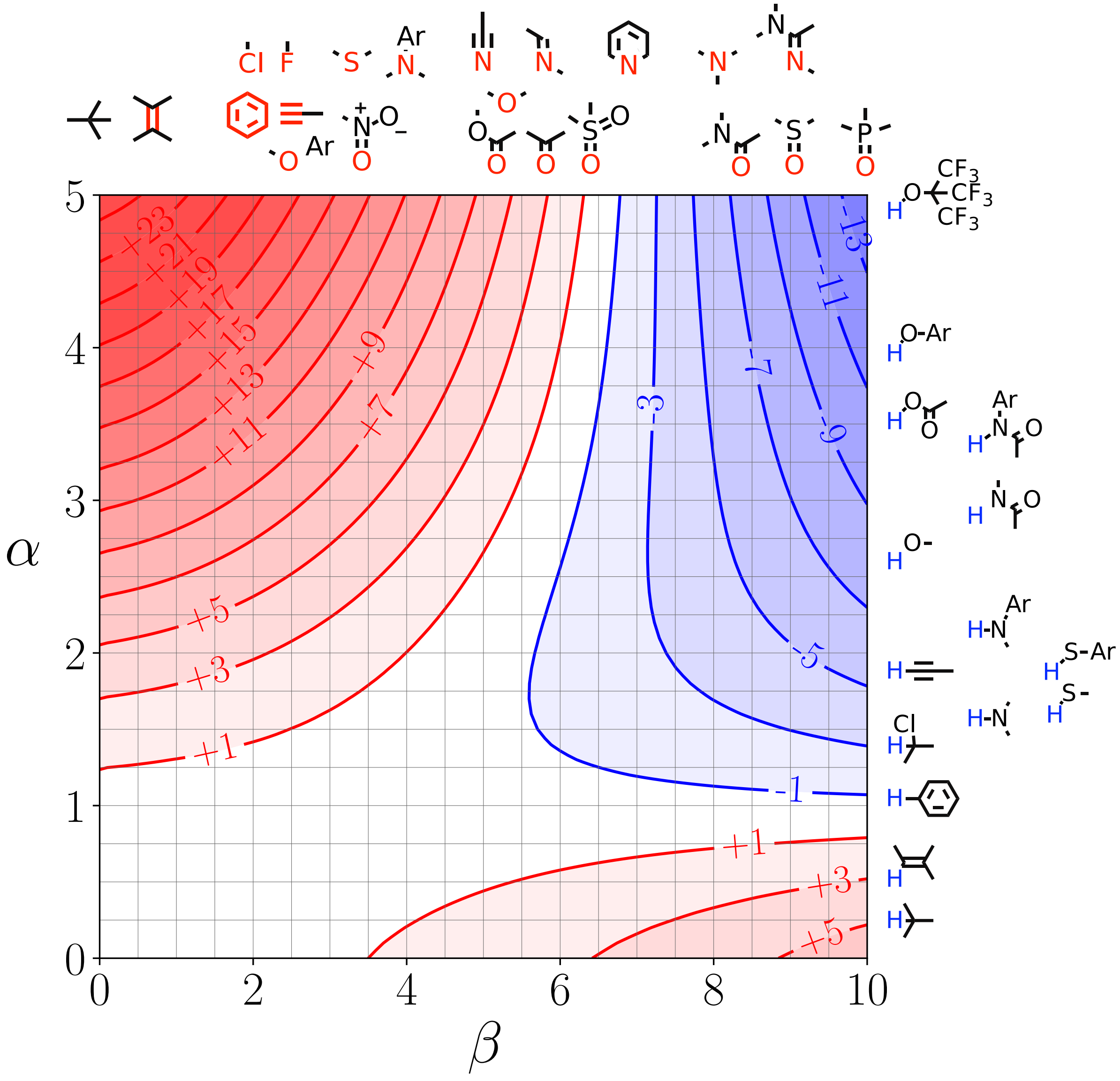

Figure S225: FGIP for N,N-dimethylthioformamide at 298K.

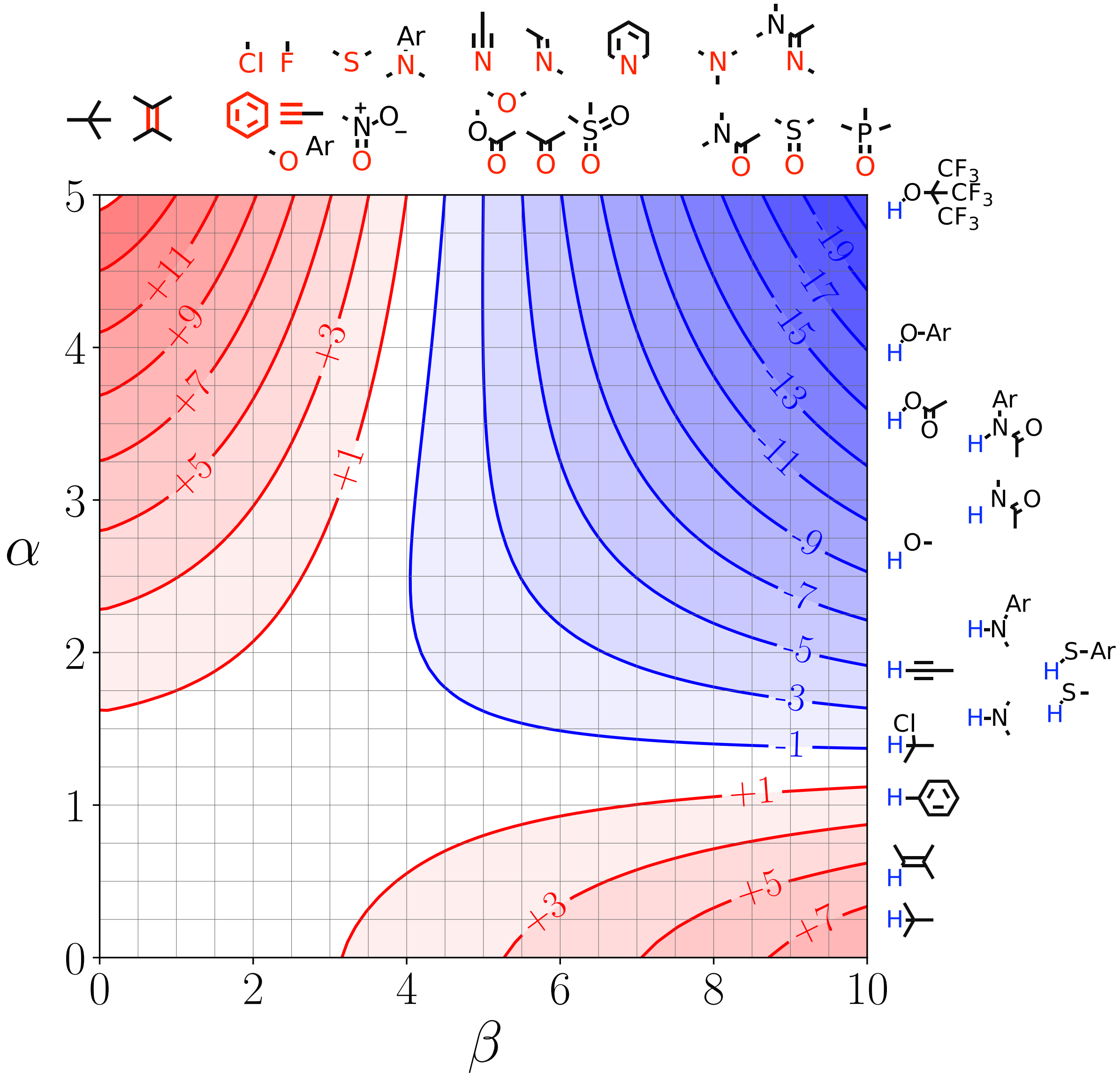

Figure S226: FGIP for N,N-diethylformamide at 298K.

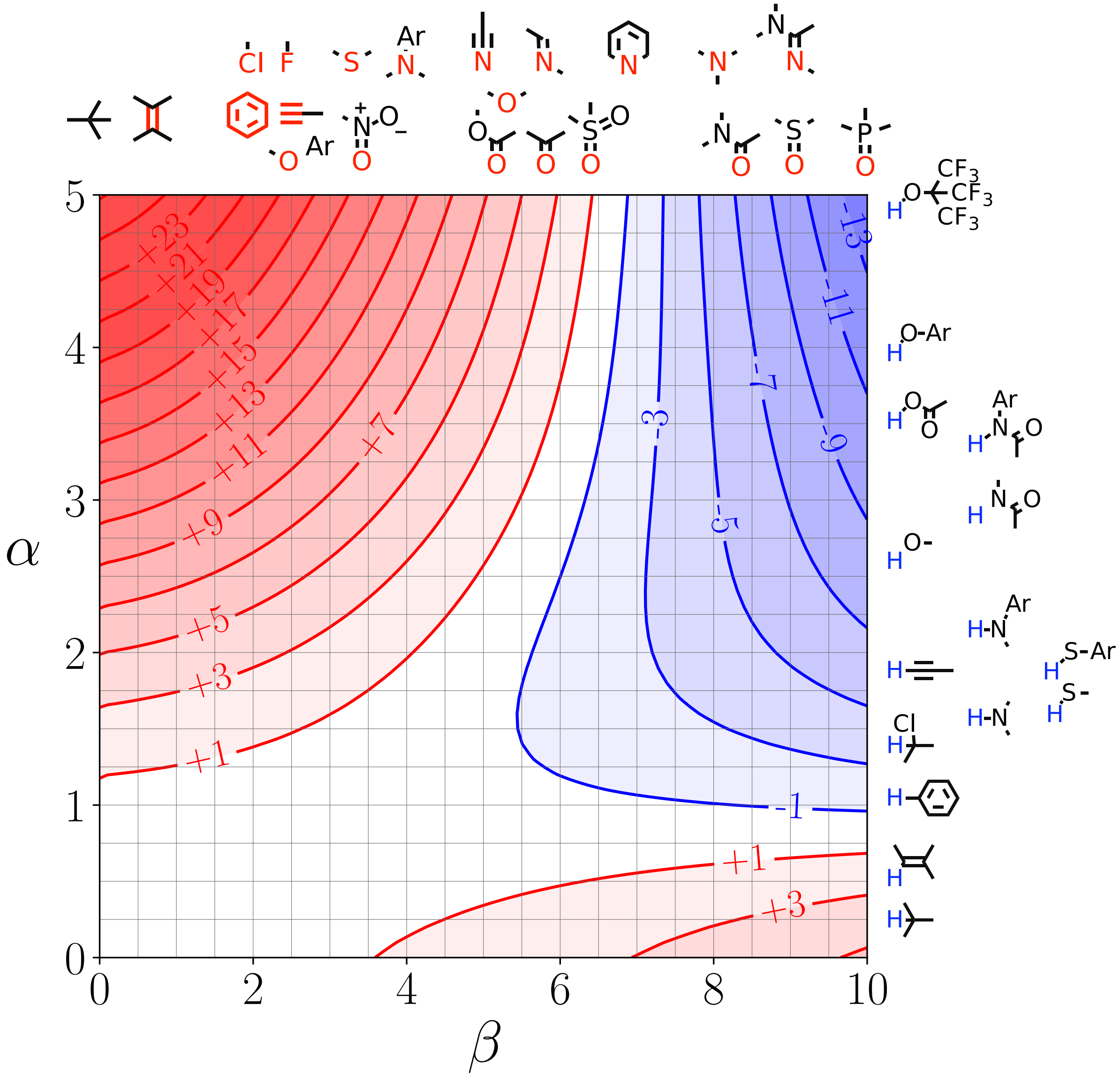

Figure S227: FGIP for N-methylacetamide at 298K.

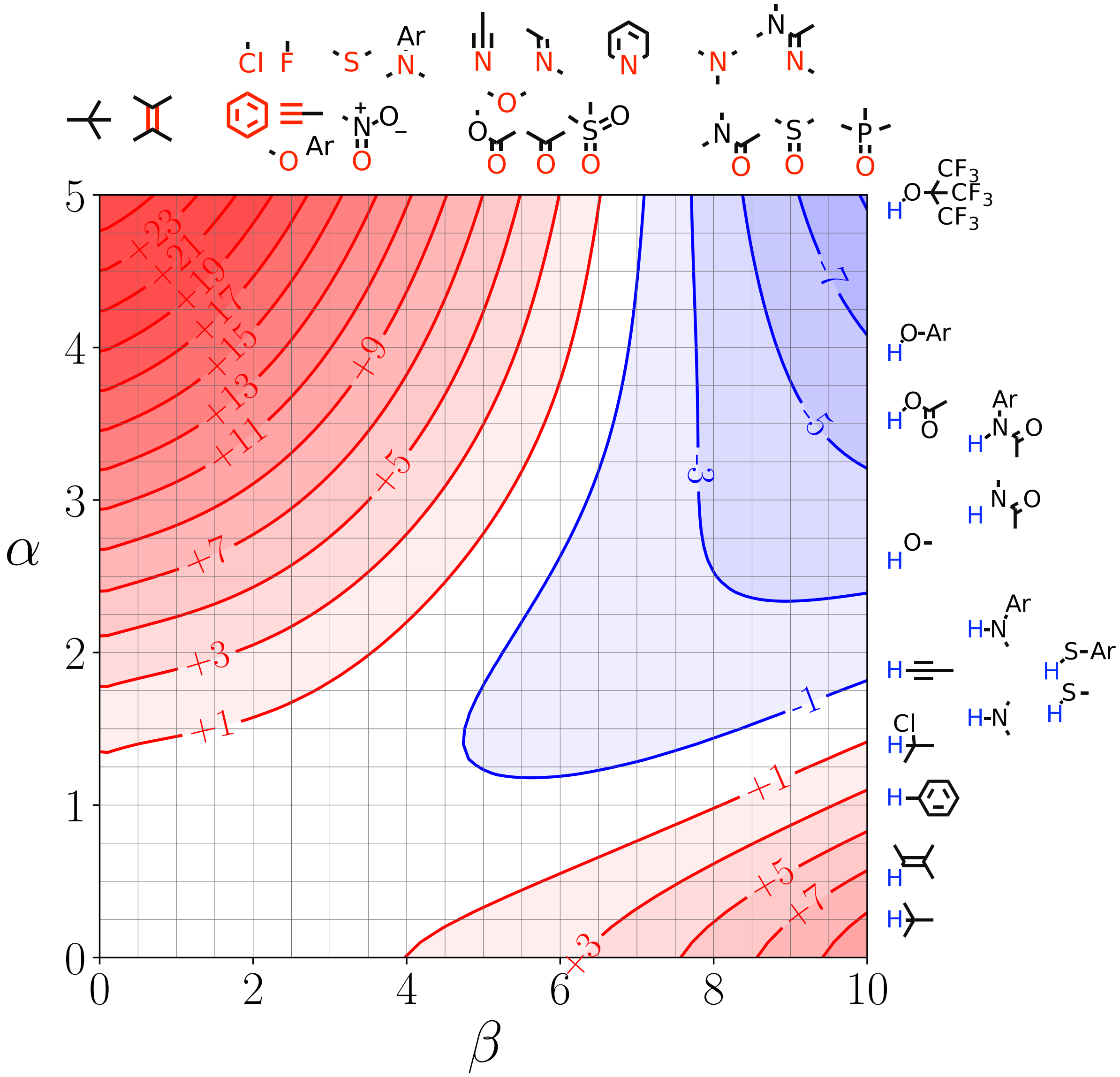

Figure S228: FGIP for N,N-dimethylacetamide at 298K.

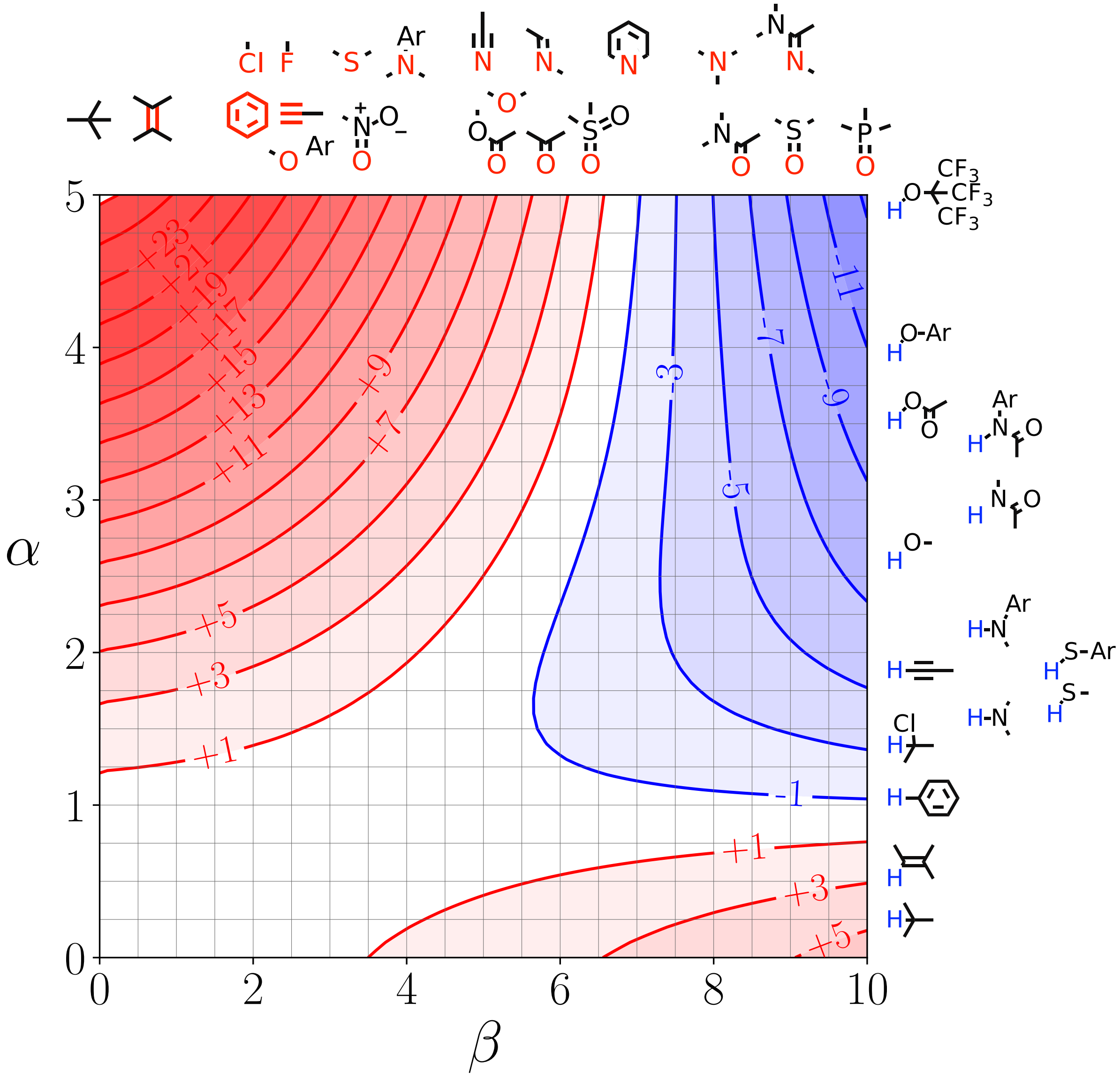

Figure S229: FGIP for N,N-diethylacetamide at 298K.

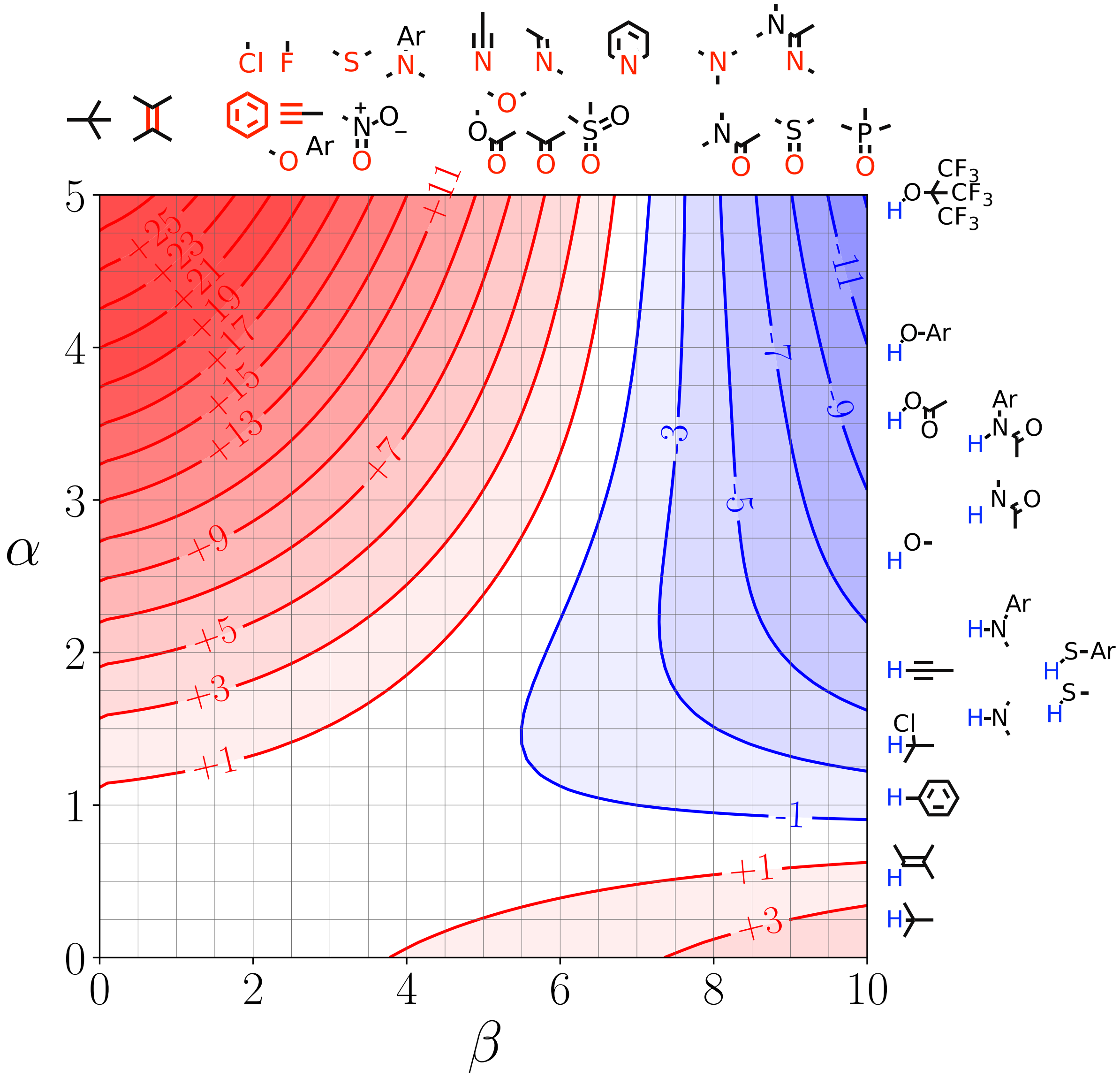

Figure S230: FGIP for 2-pyrrolidinone at 298K.

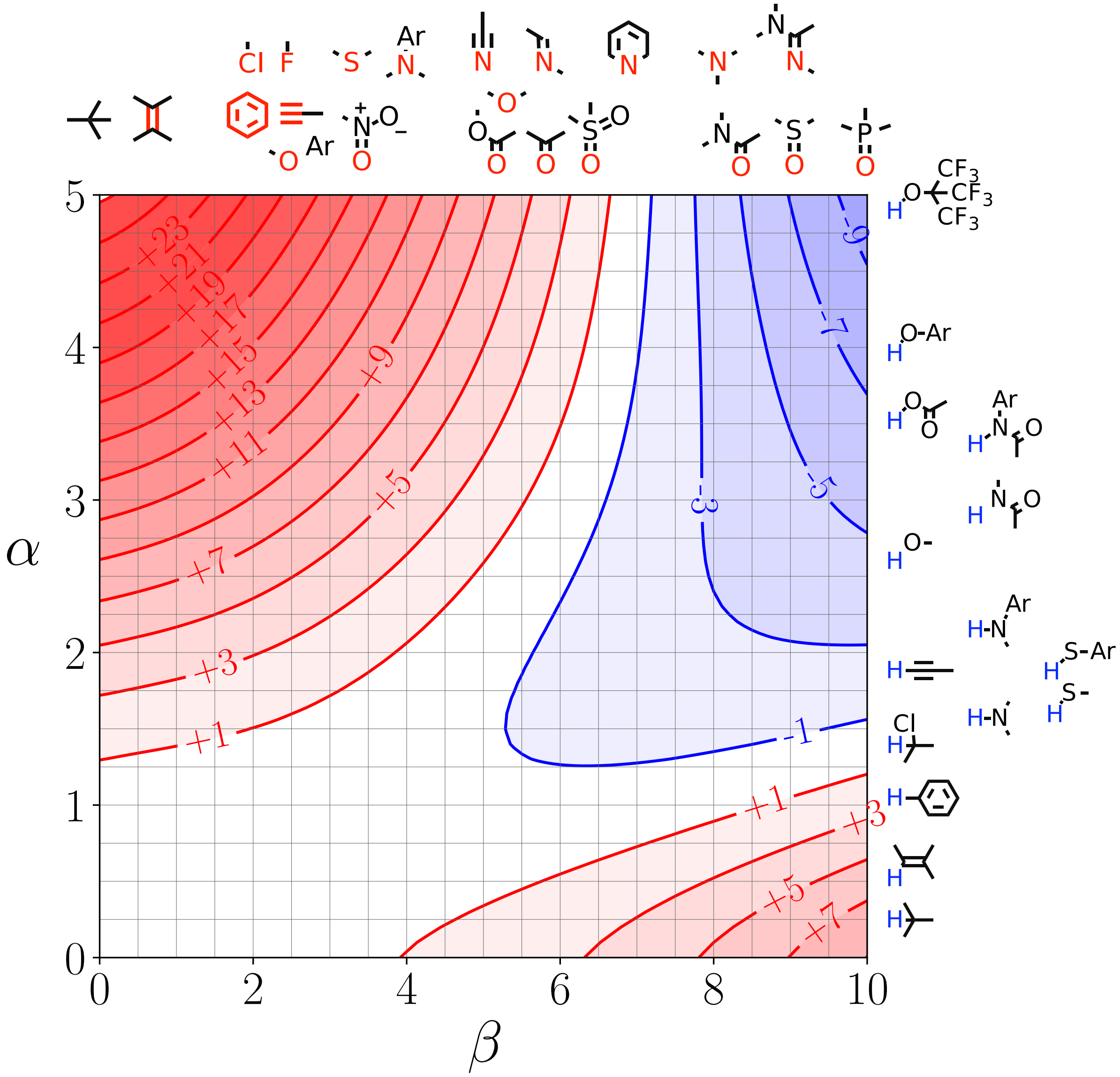

Figure S231: FGIP for N-methyl pyrrolidinone at 298K.

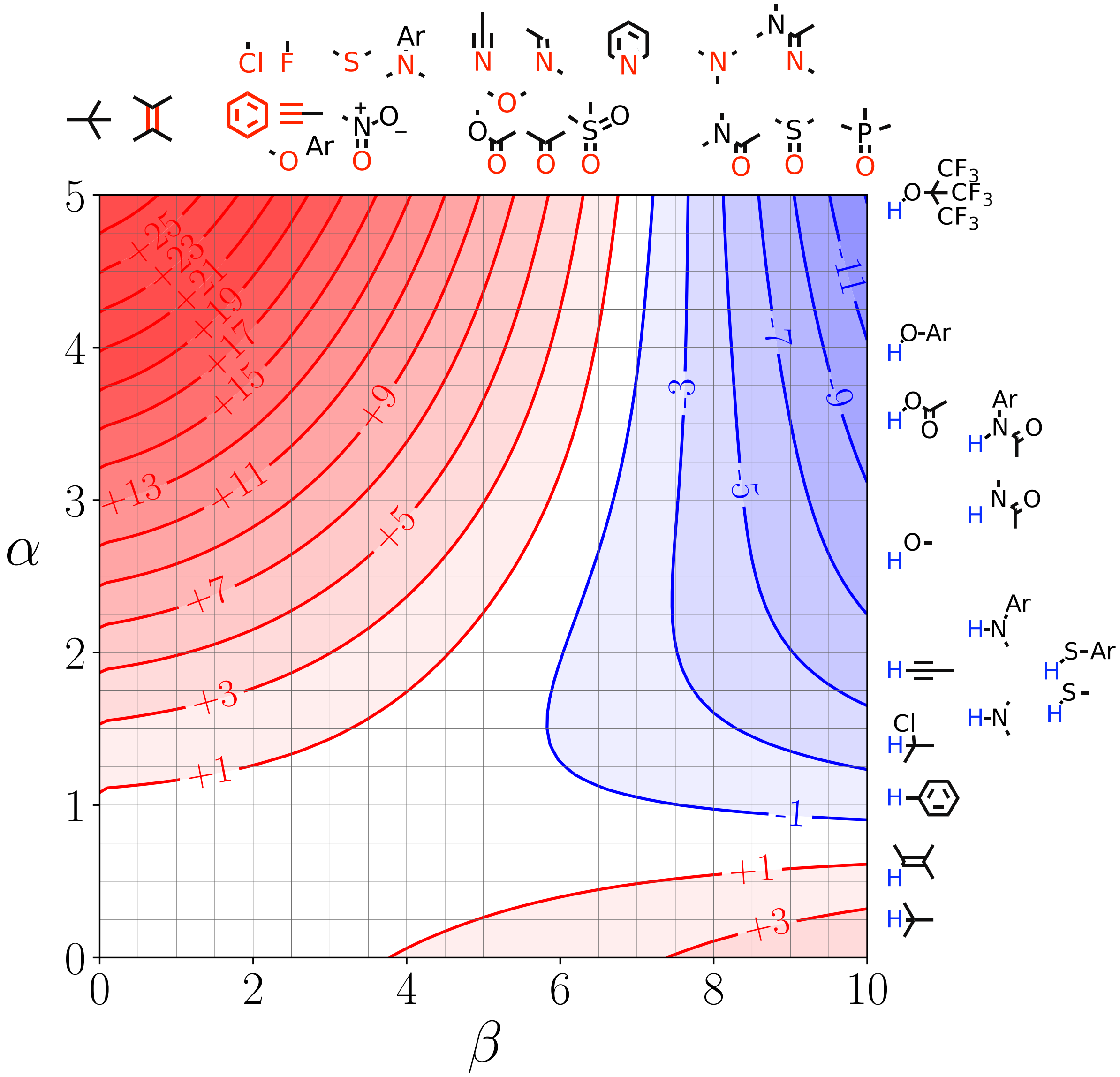

Figure S232: FGIP for 1-methyl-2-pyrrolidinethione at 298K.

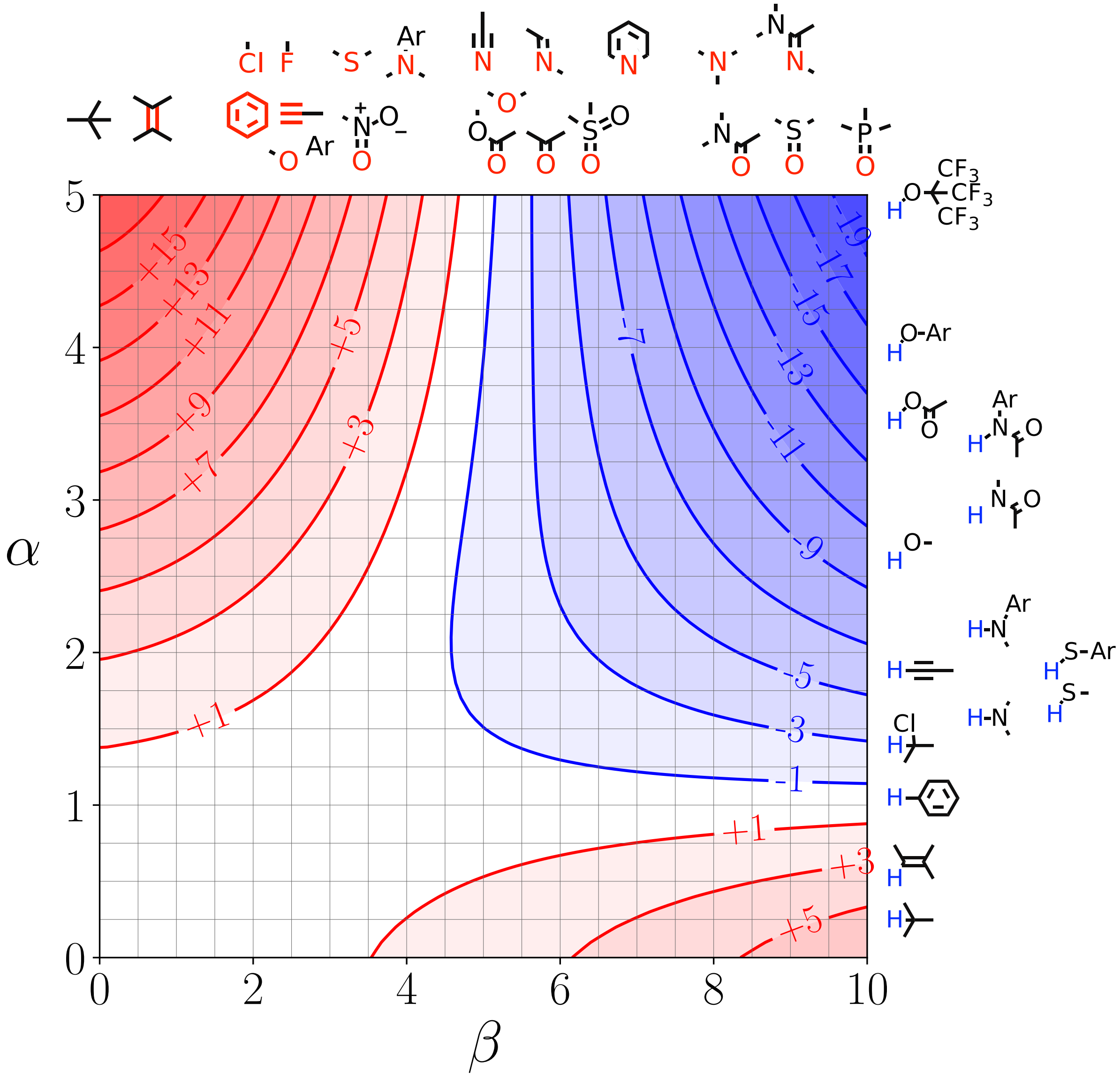

Figure S233: FGIP for tetramethylurea at 298K.

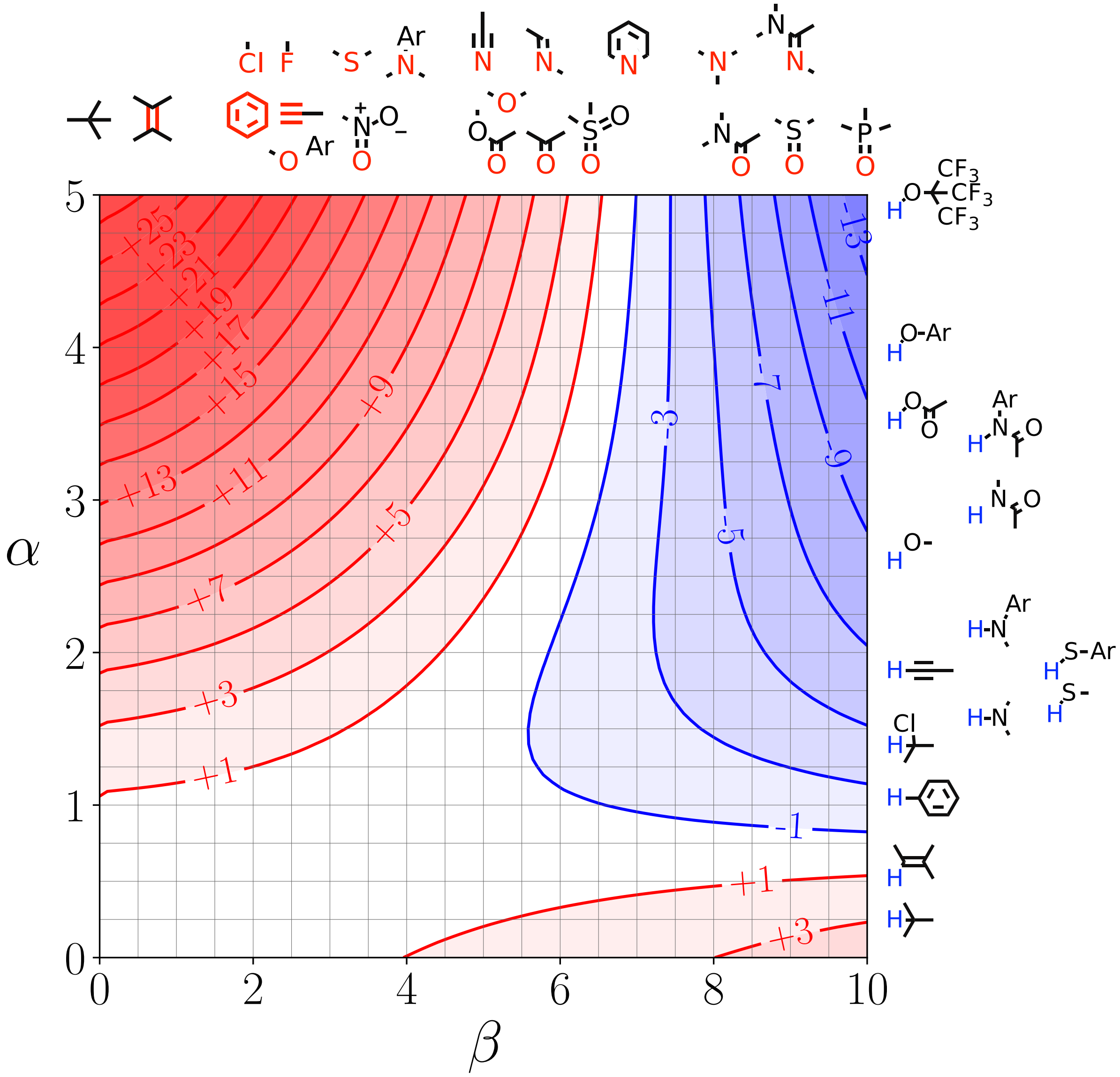

Figure S234: FGIP for tetraethylurea at 298K.

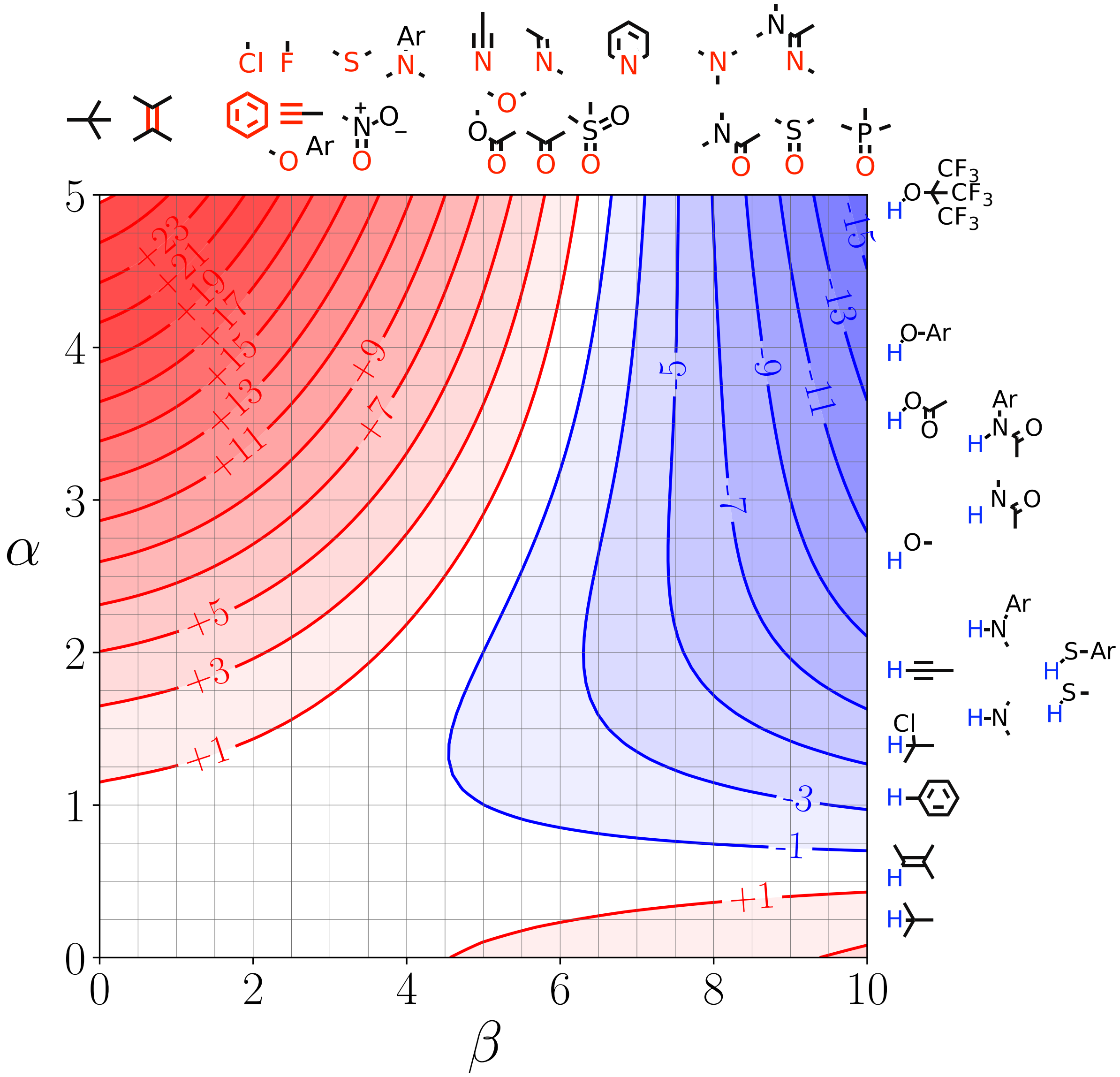

Figure S235: FGIP for dimethylcyanamide at 298K.

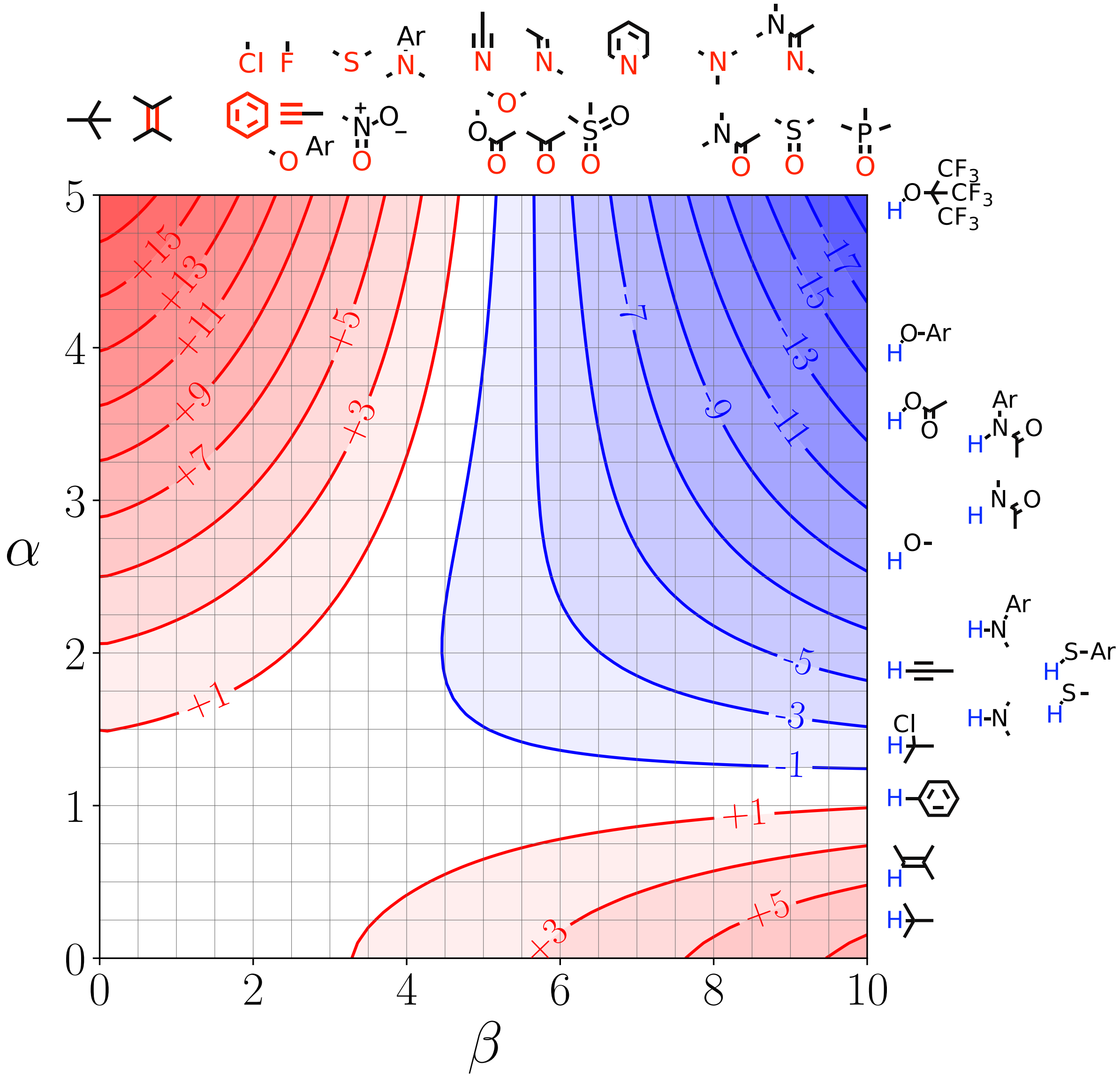

Figure S236: FGIP for carbon disulfide at 298K.

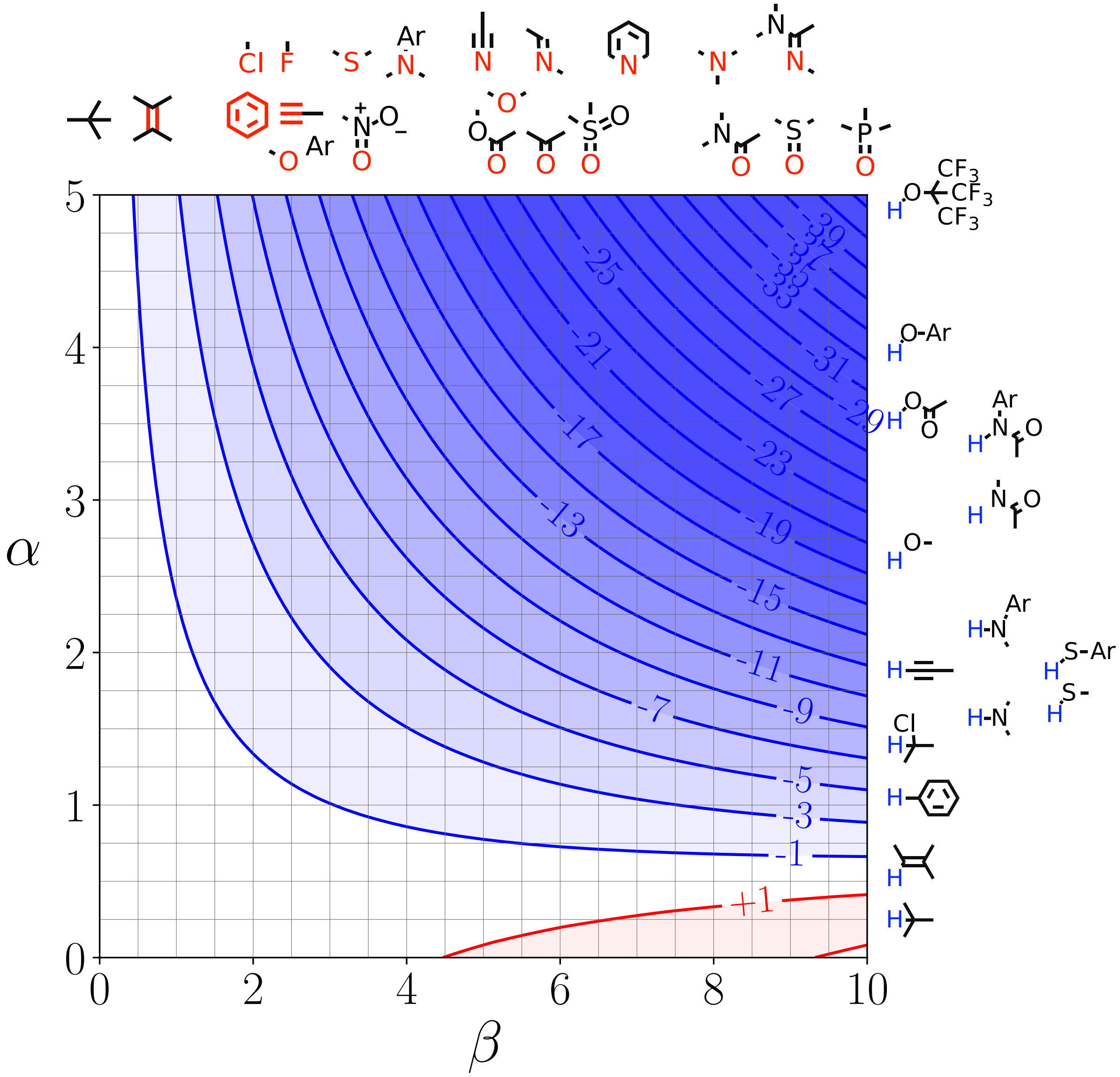

Figure S237: FGIP for dimethyl sulfide at 298K.

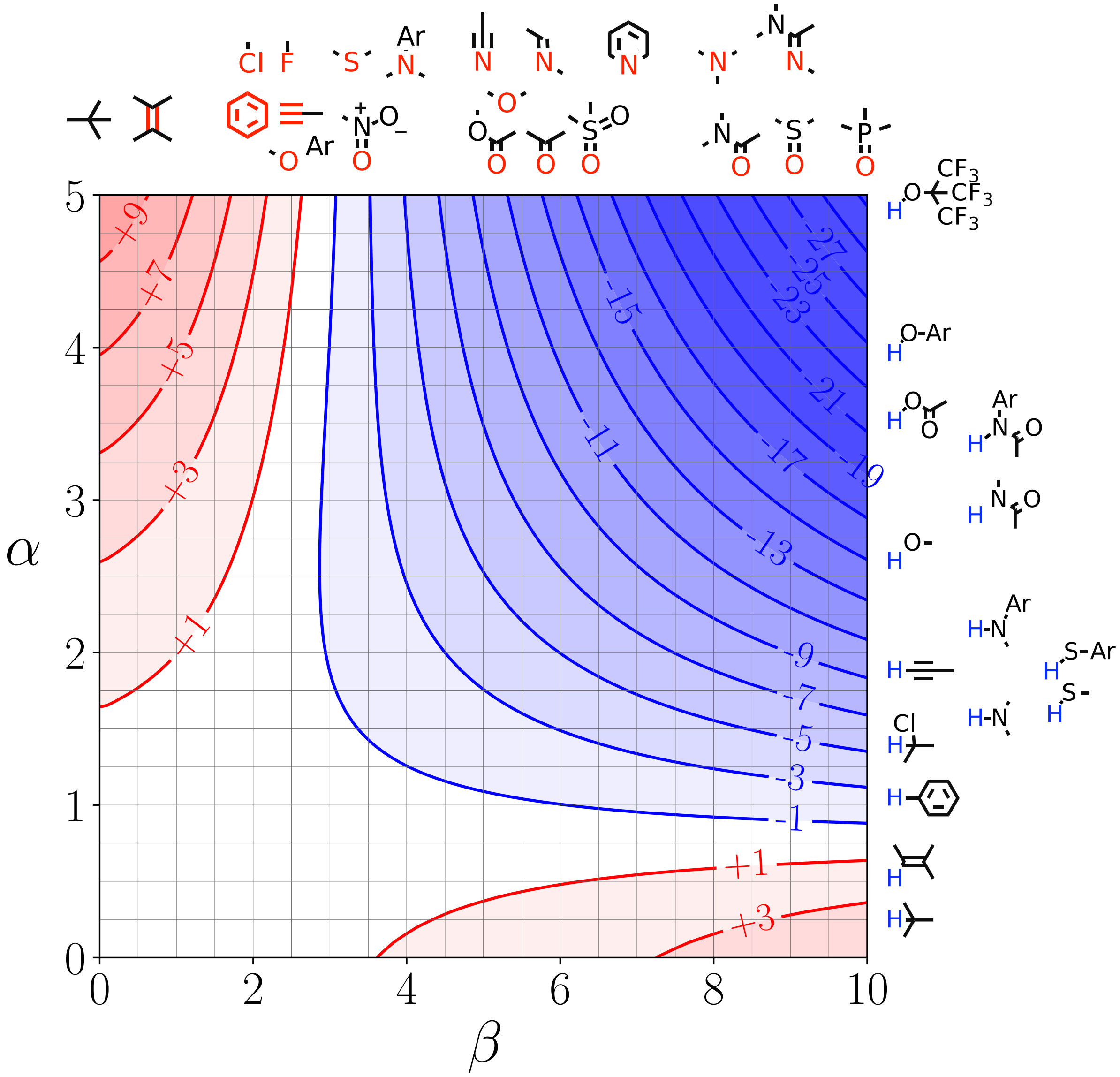

Figure S238: FGIP for diethyl sulfide at 298K.

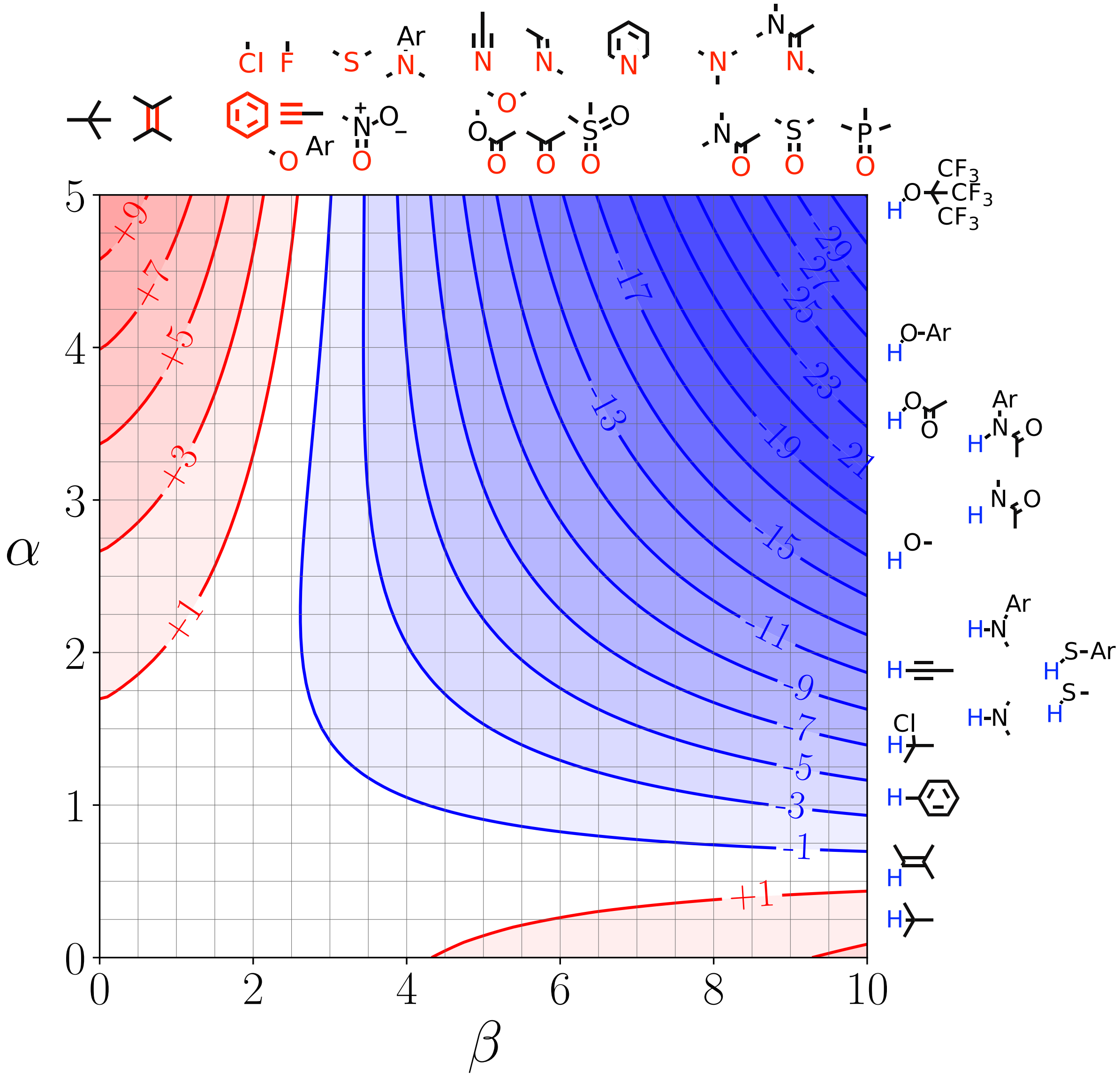

Figure S239: FGIP for diisopropyl sulfide at 298K.

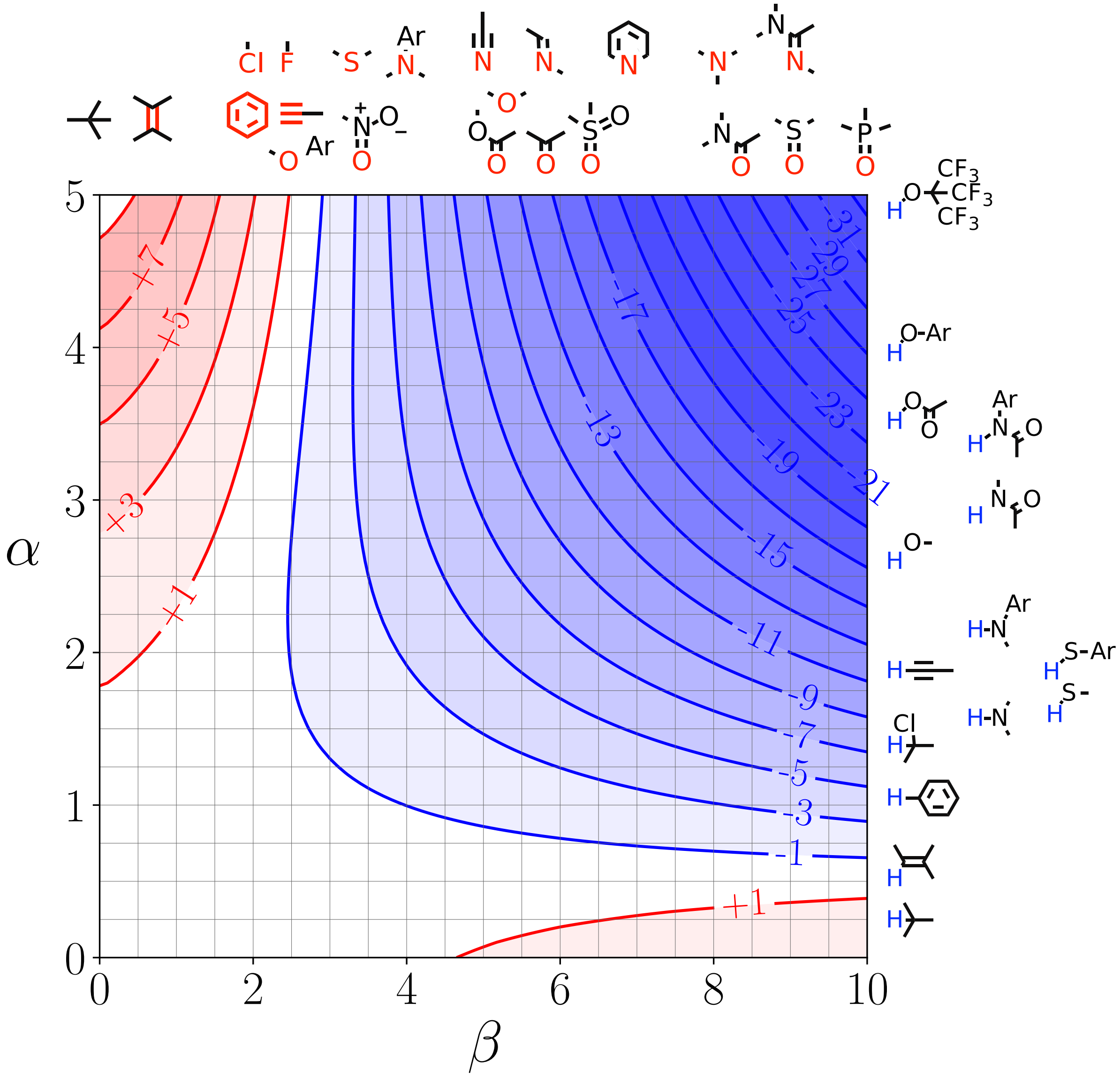

Figure S240: FGIP for dibutyl sulfide at 298K.

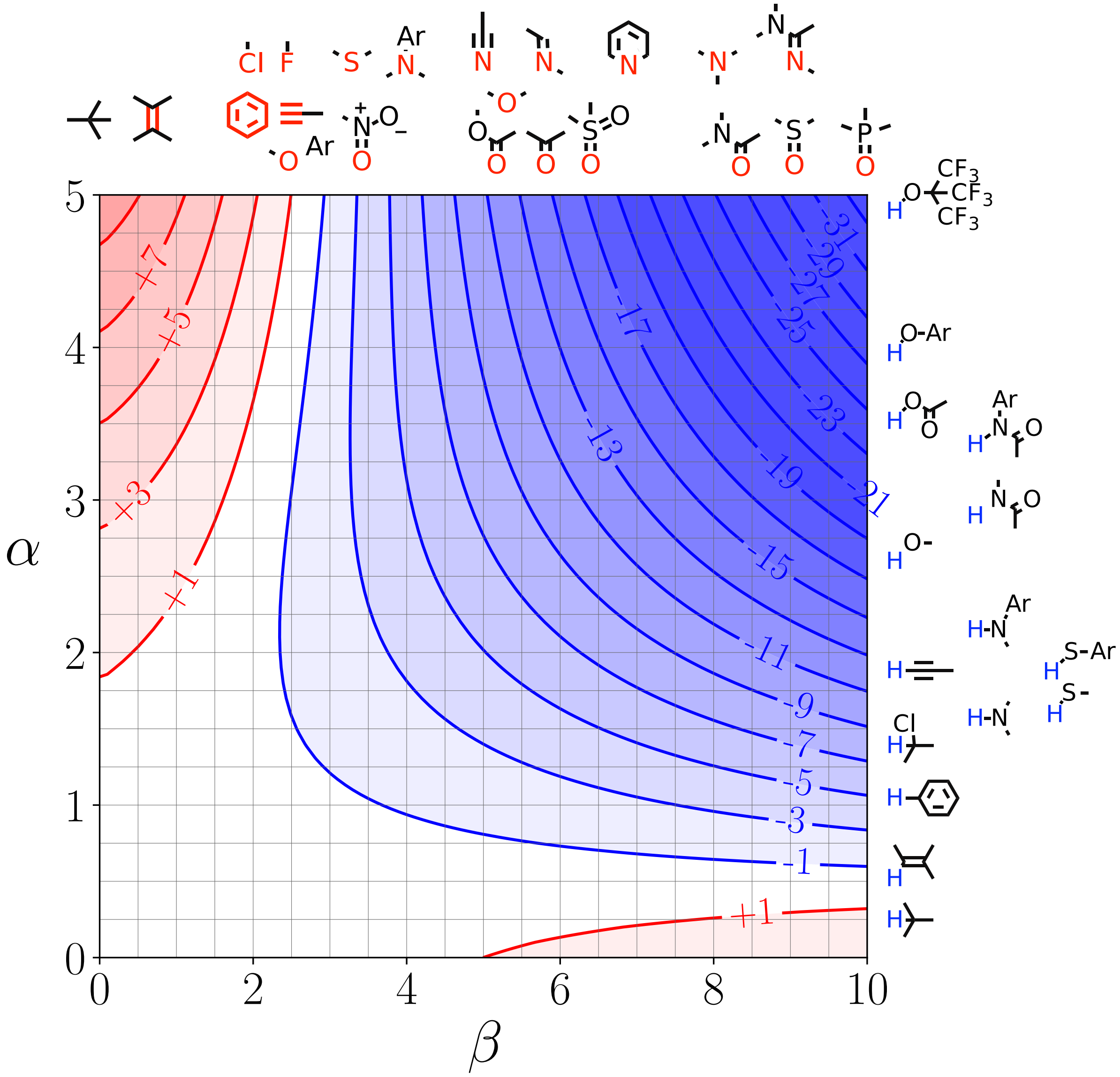

Figure S241: FGIP for tetrahydrothiophene at 298K.

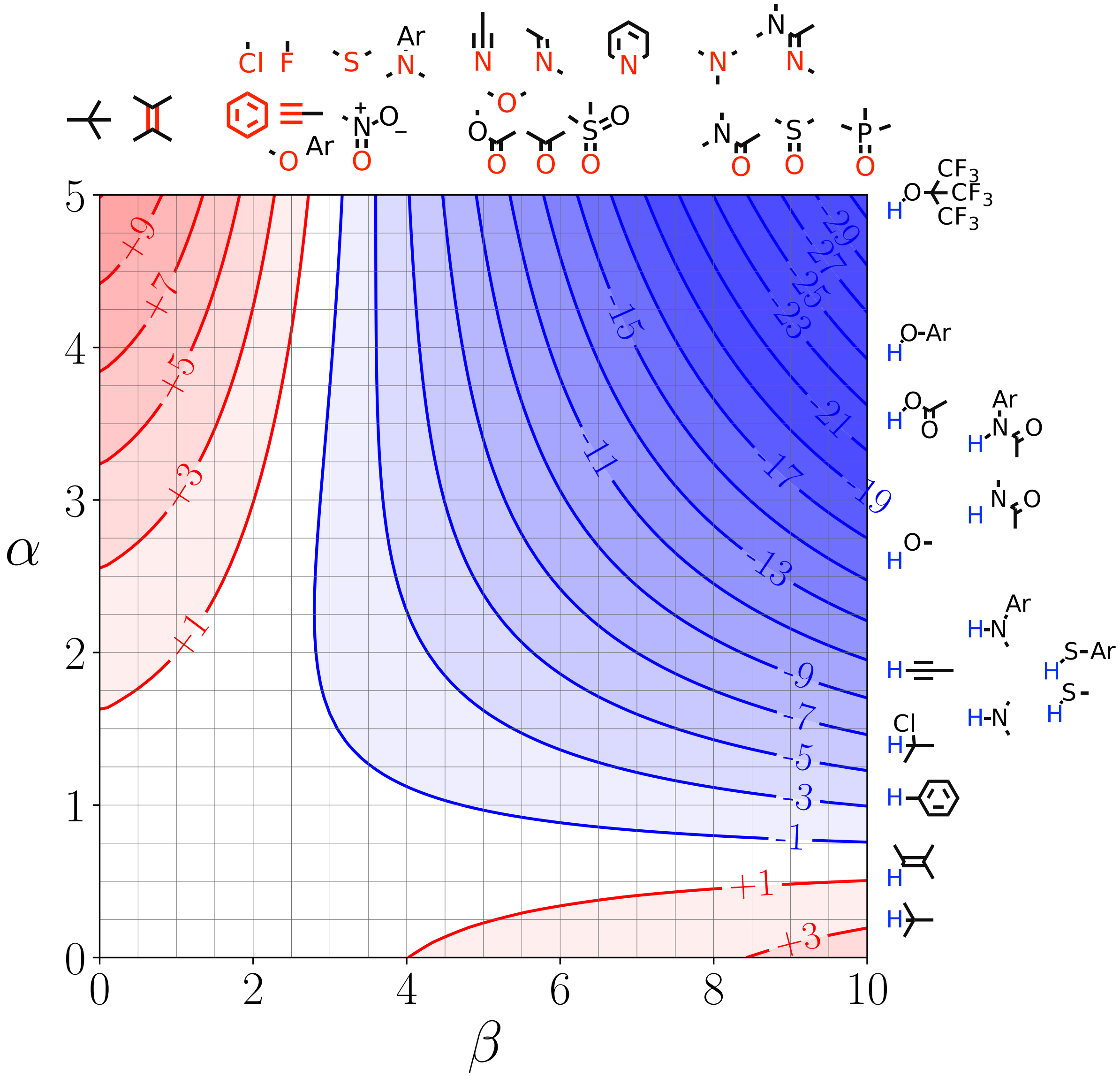

Figure S242: FGIP for thiane at 298K.

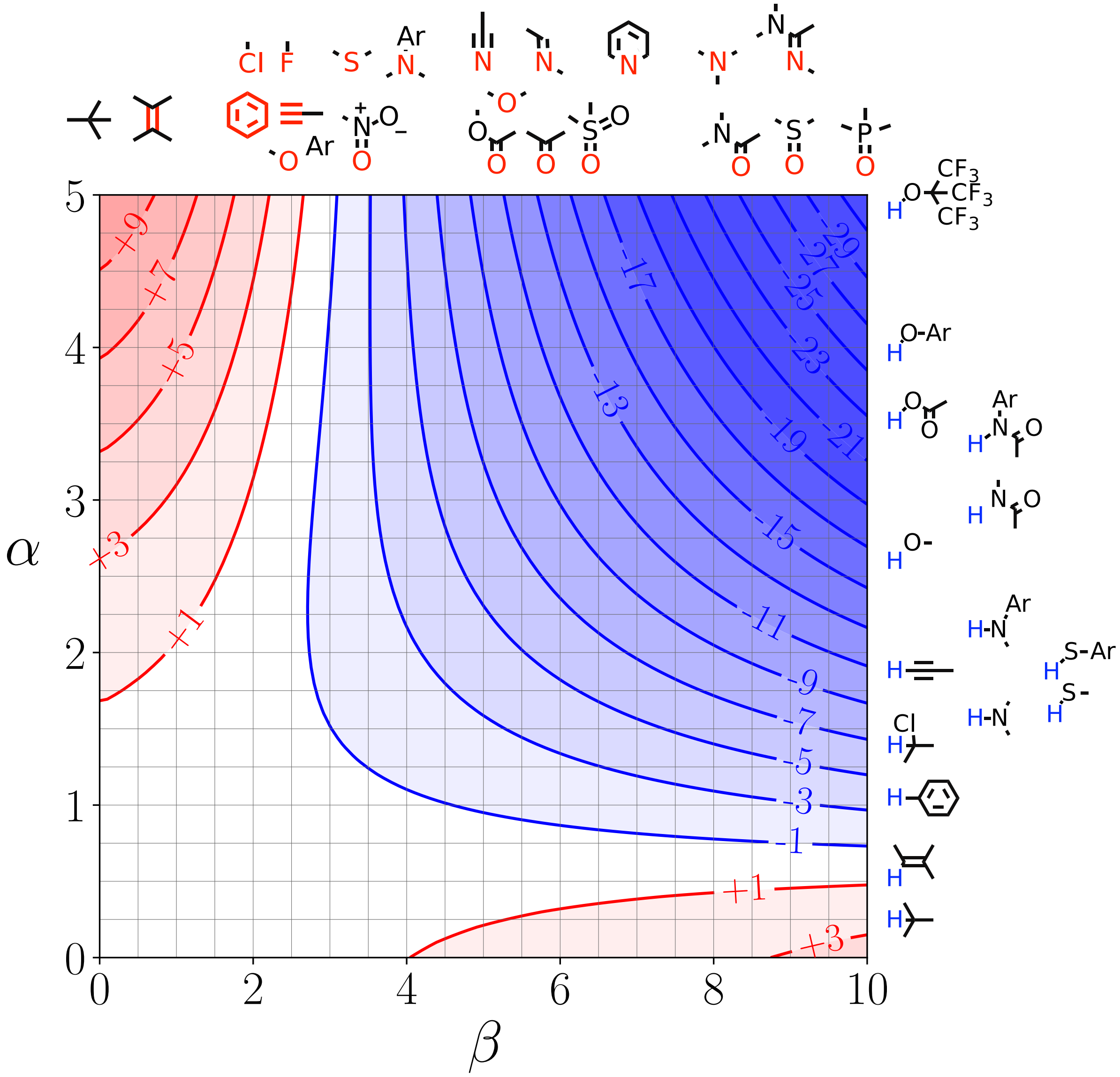

Figure S243: FGIP for dimethylsulfoxide at 298K.

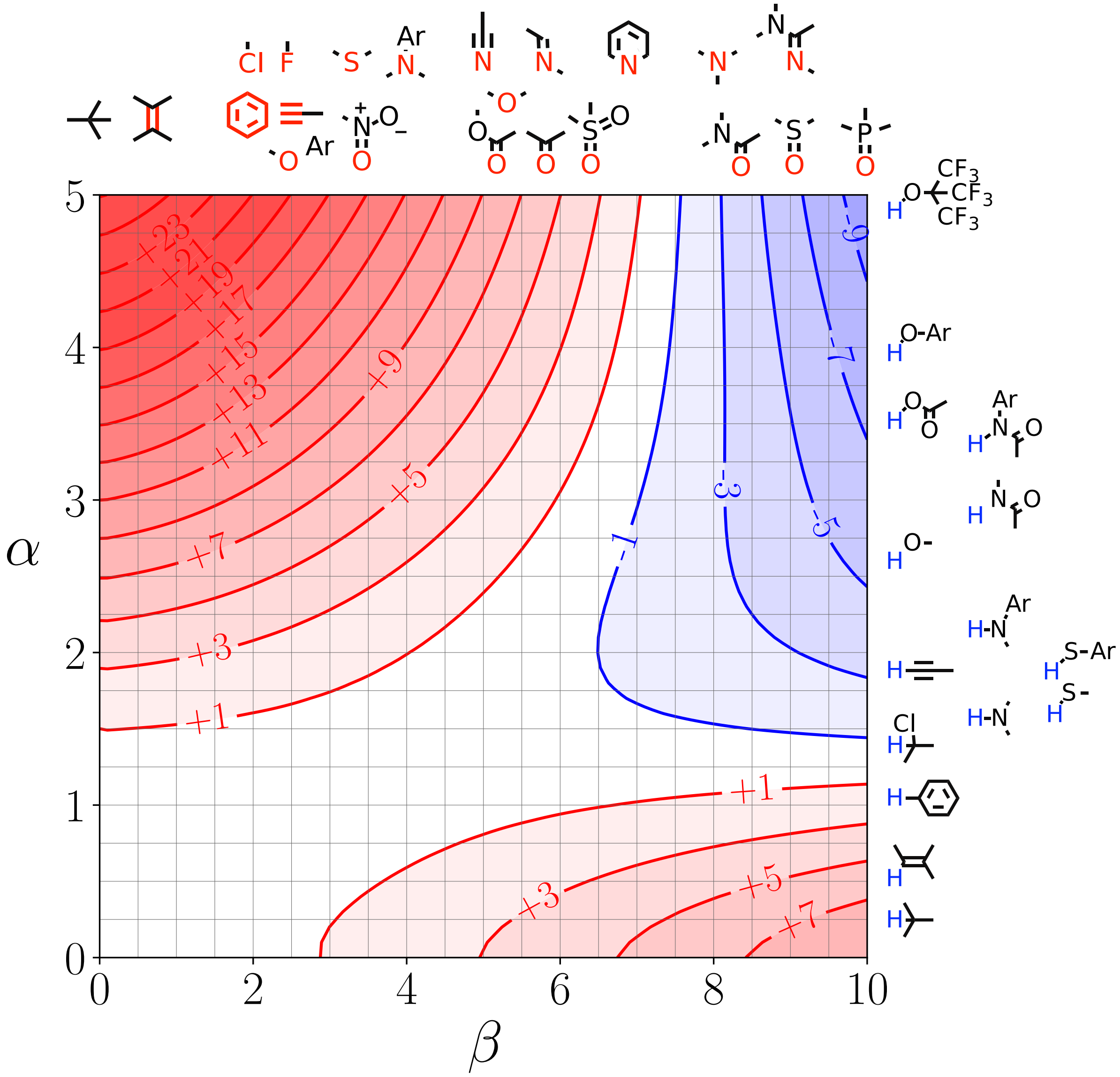

Figure S244: FGIP for dibutyl sulfoxide at 298K.

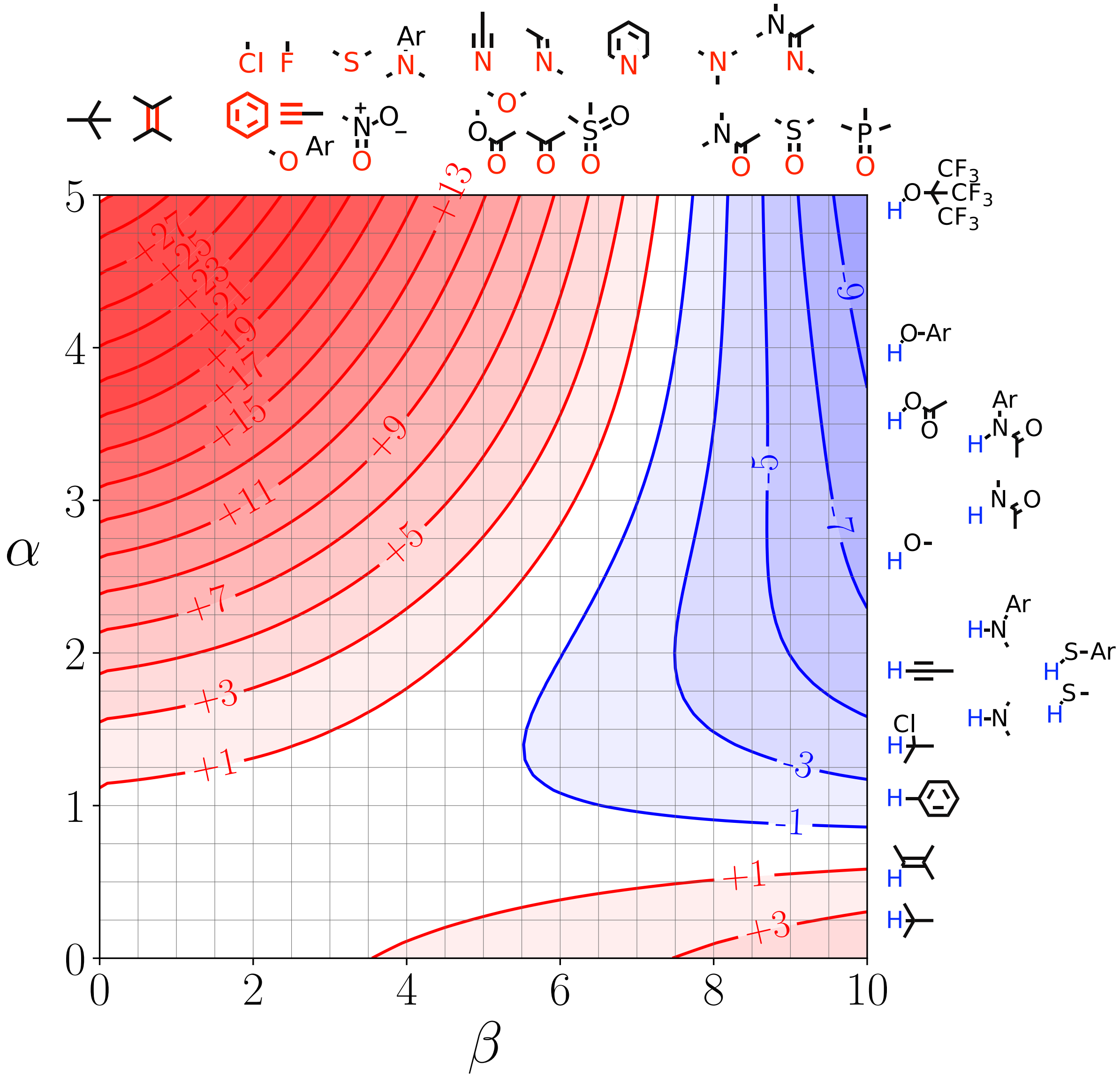

Figure S245: FGIP for sulfolane at 298K.

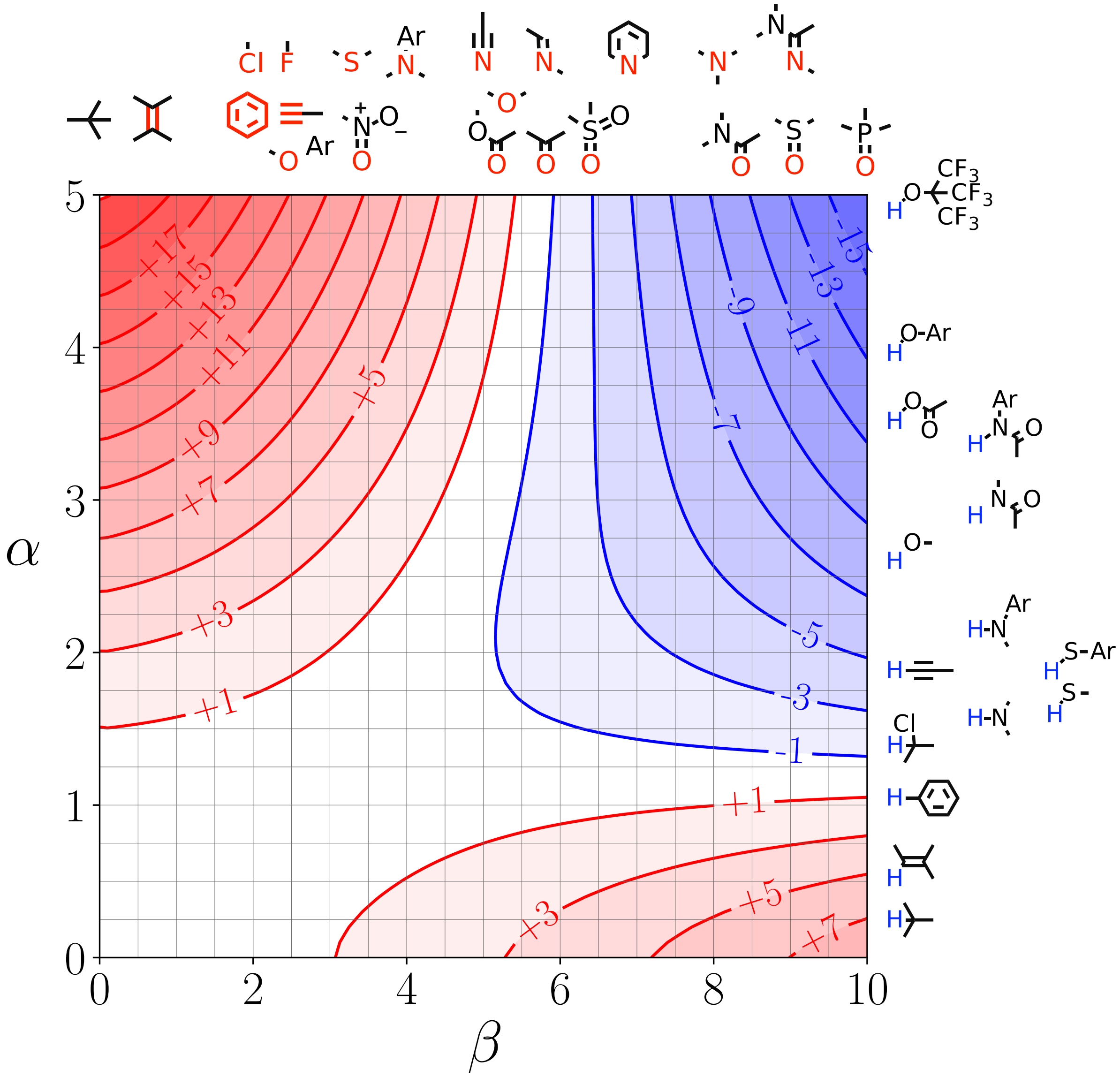

Figure S246: FGIP for thiobis(2-ethanol) at 298K.

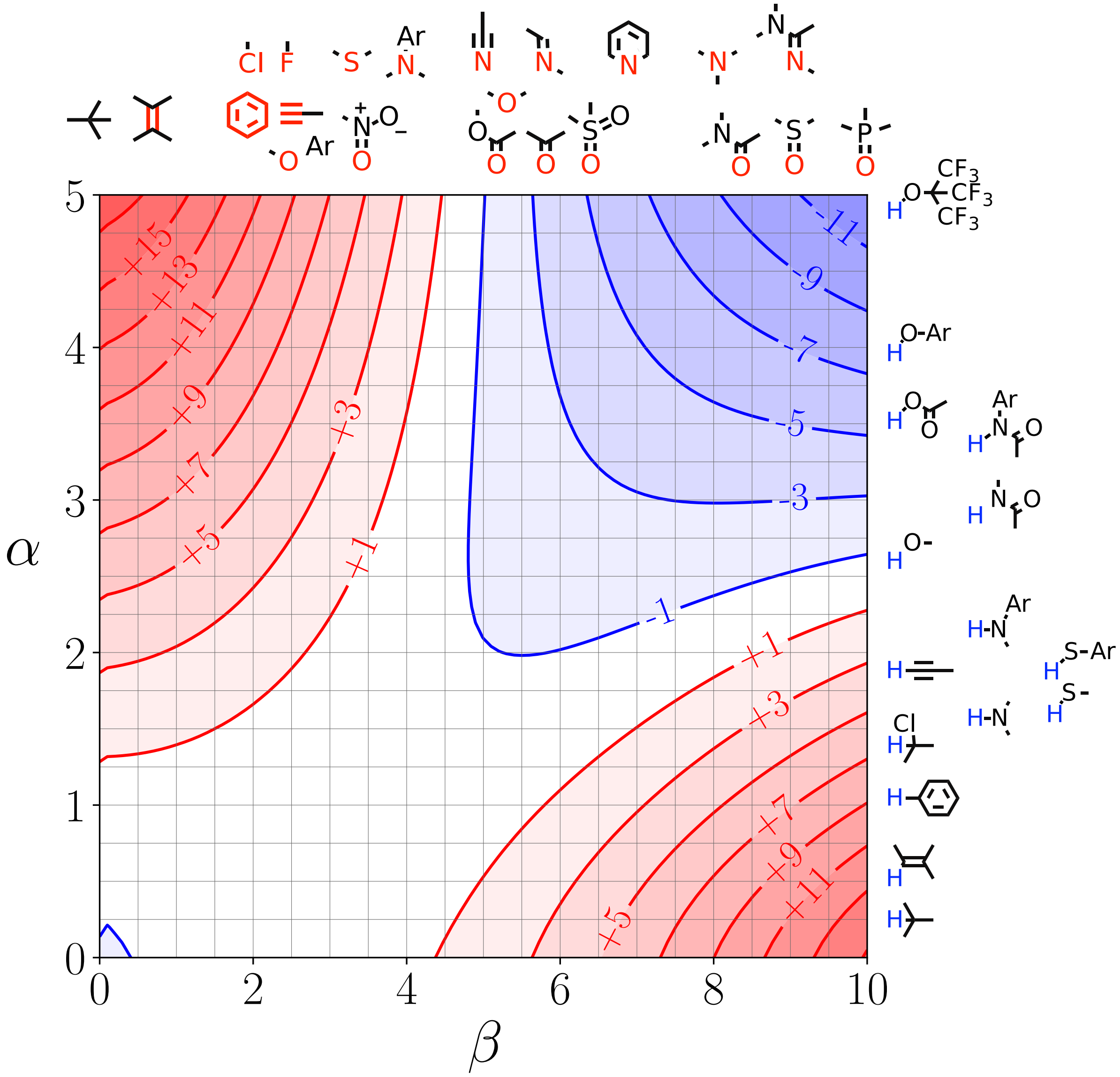

Figure S247: FGIP for diethyl sulfite at 298K.

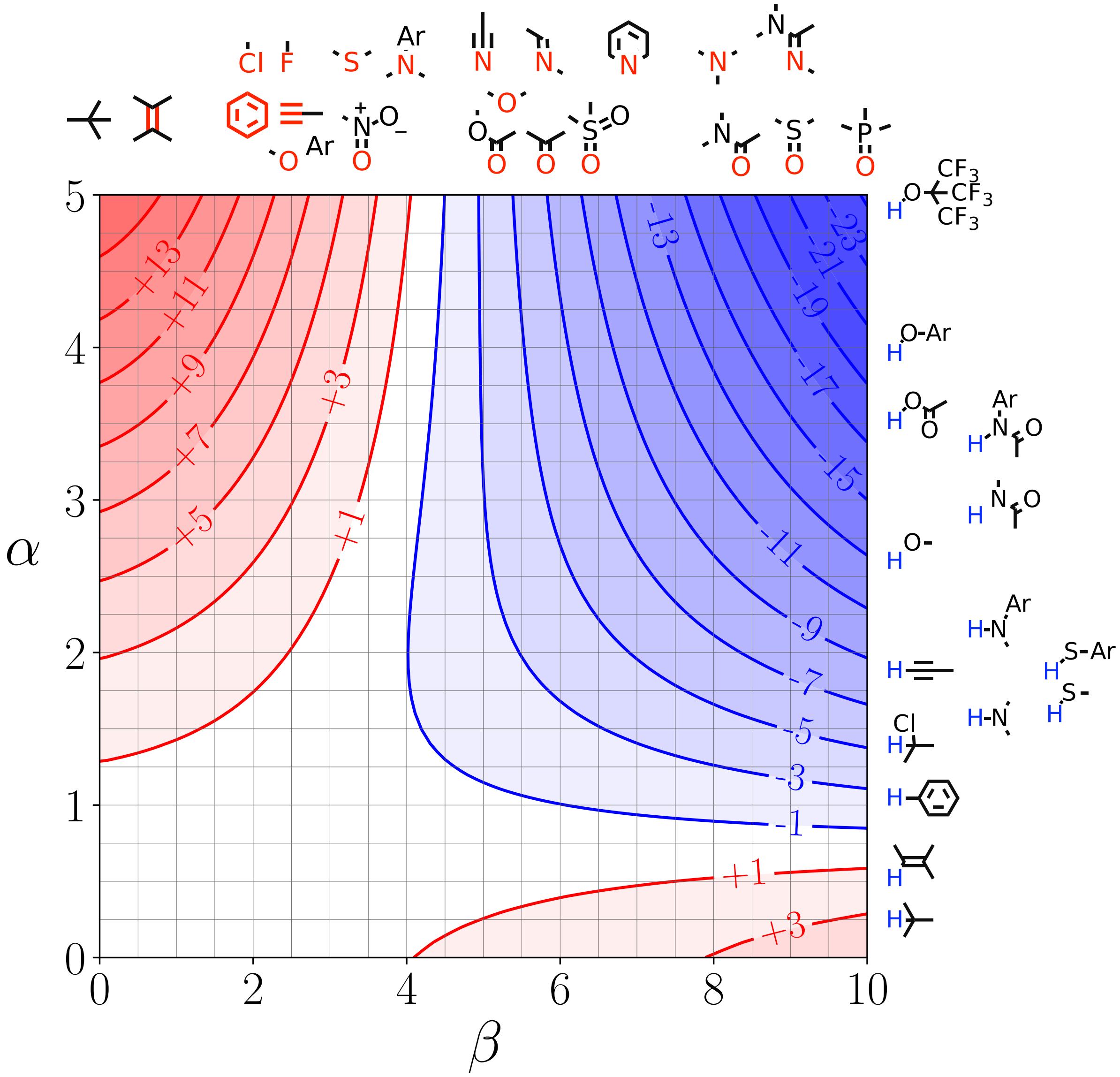

Figure S248: FGIP for dimethyl sulfate at 298K.

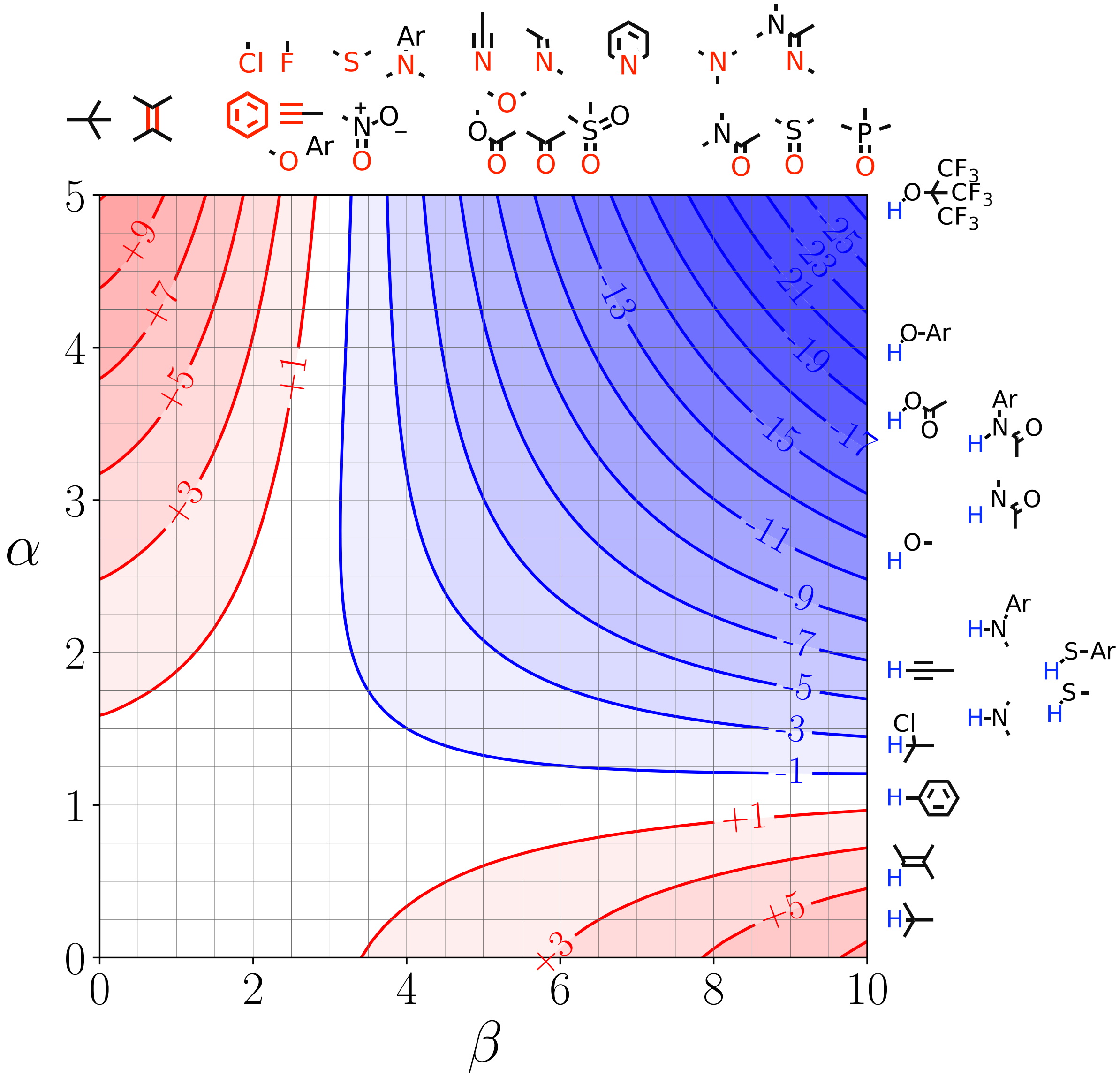

Figure S249: FGIP for diethyl sulfate at 298K.

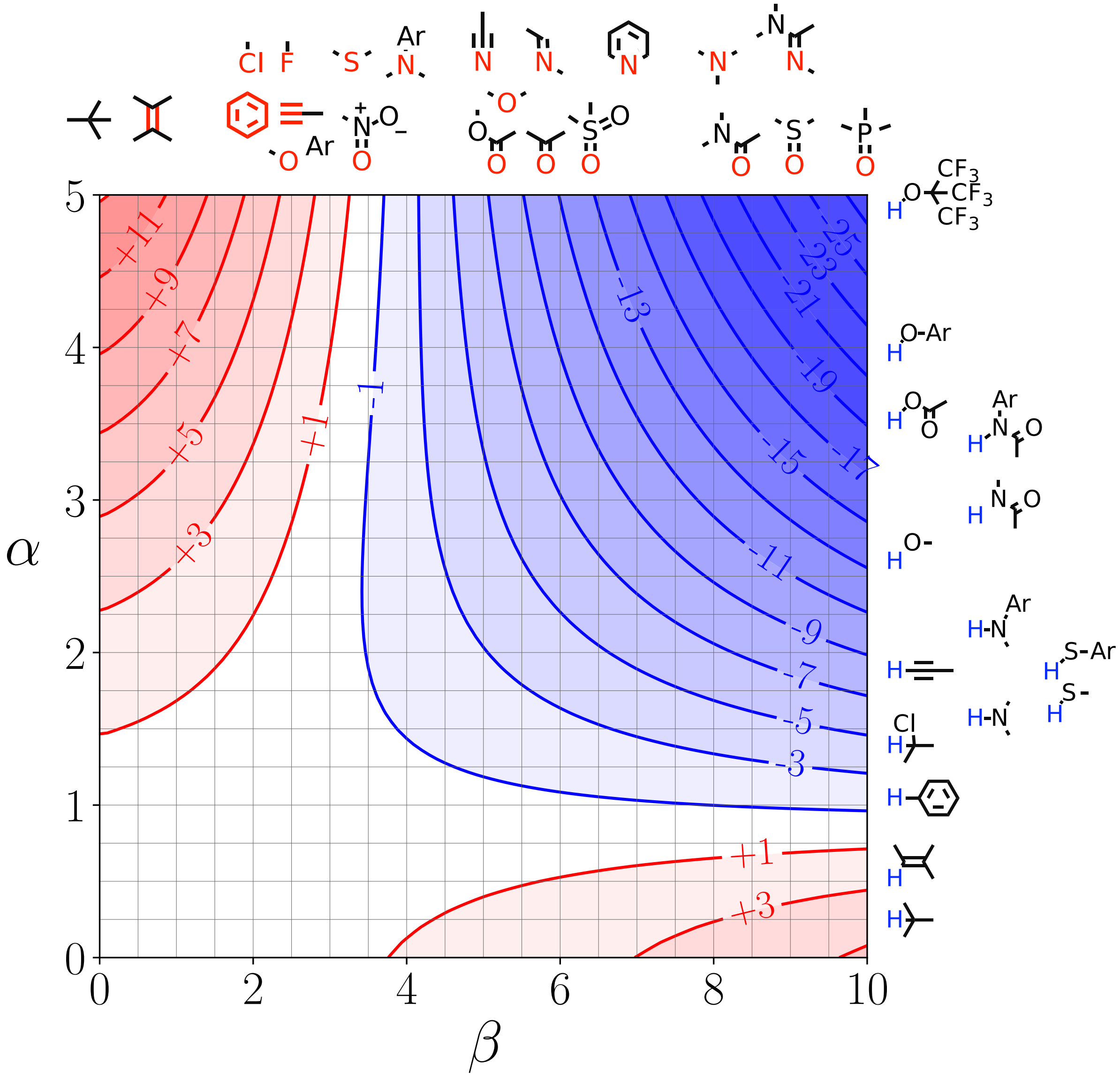

Figure S250: FGIP for methanesulfonic acid at 298K.

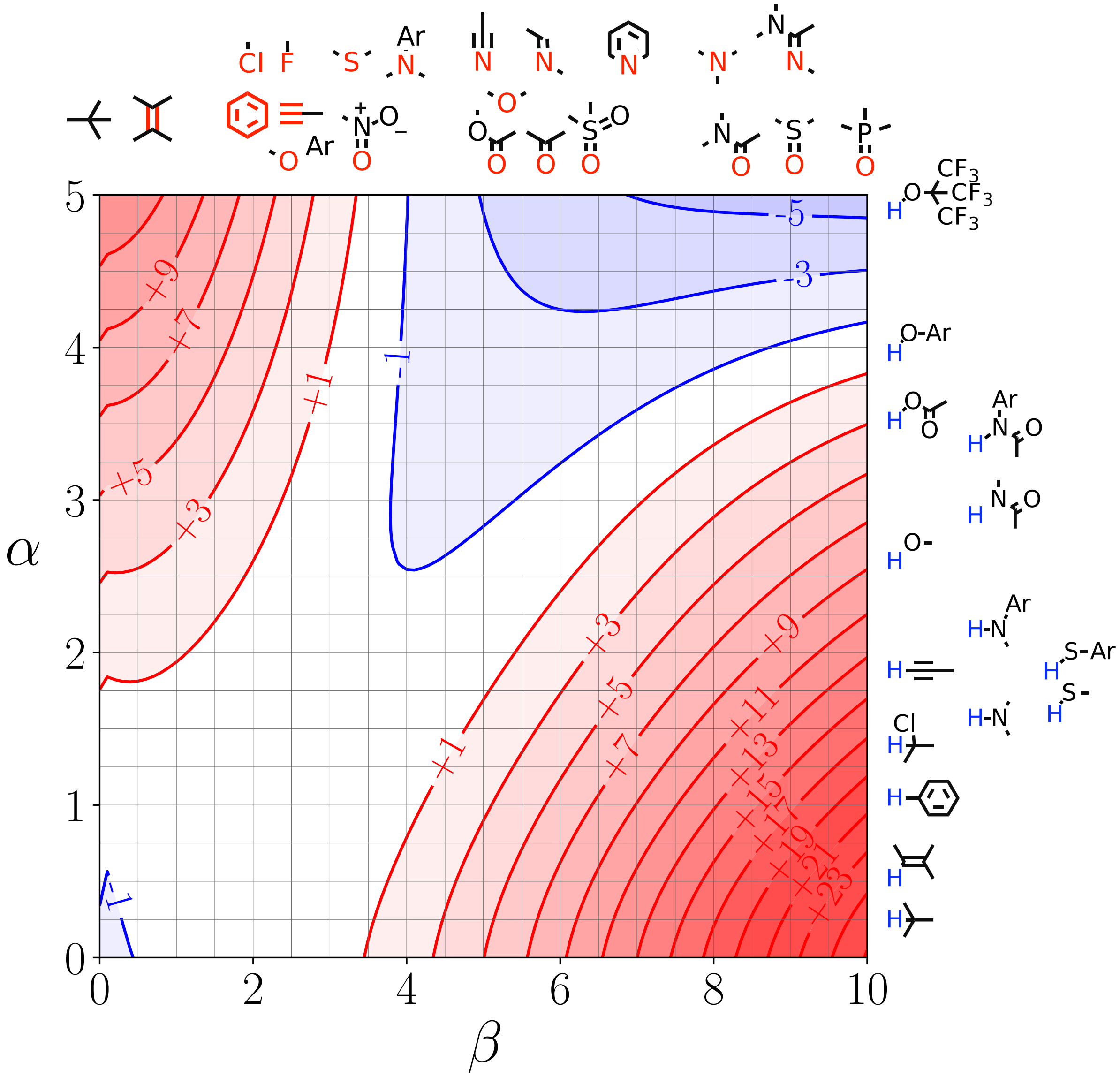

Figure S251: FGIP for trimethylphosphate at 298K.

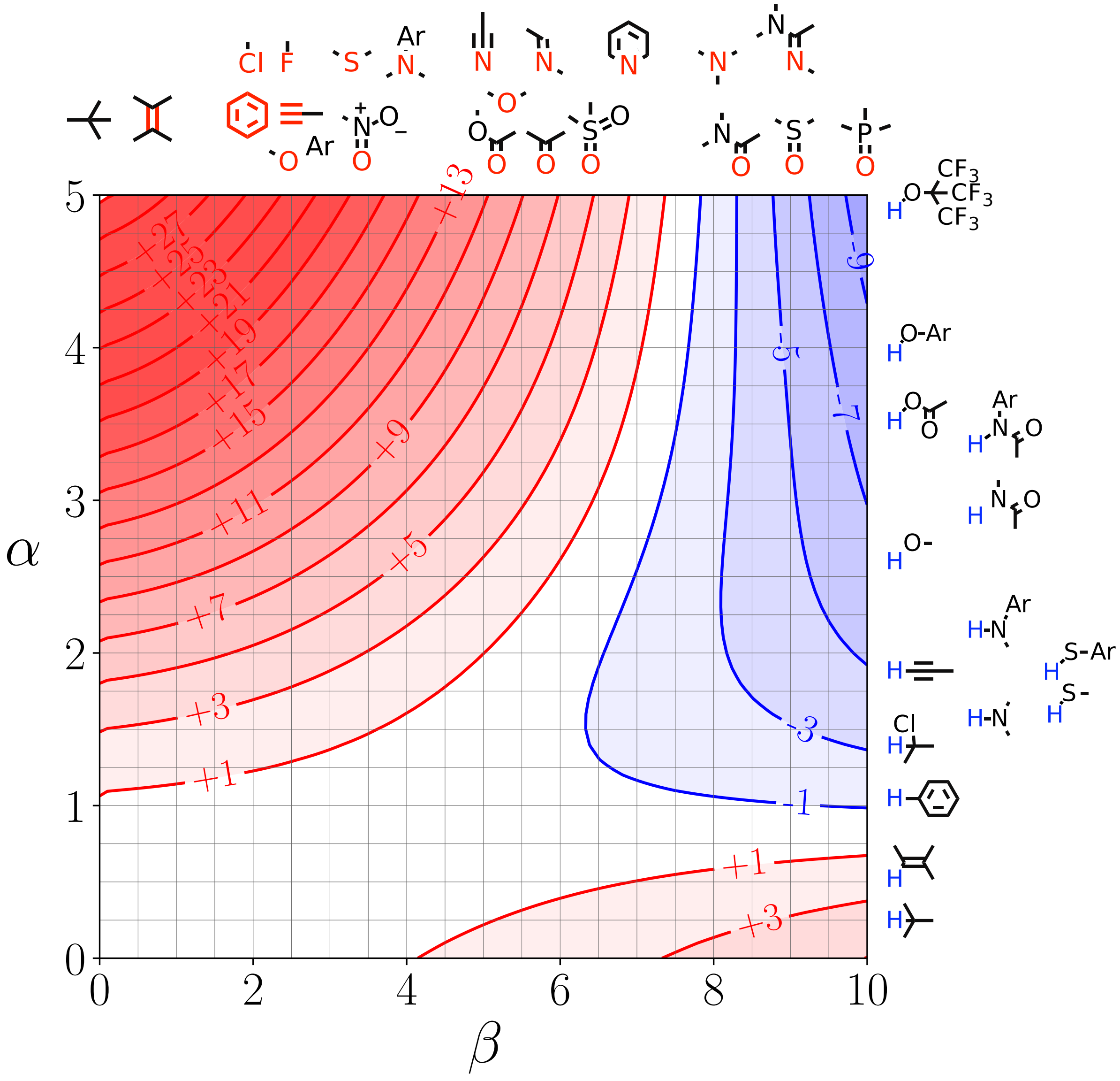

Figure S252: FGIP for triethylphosphate at 298K.

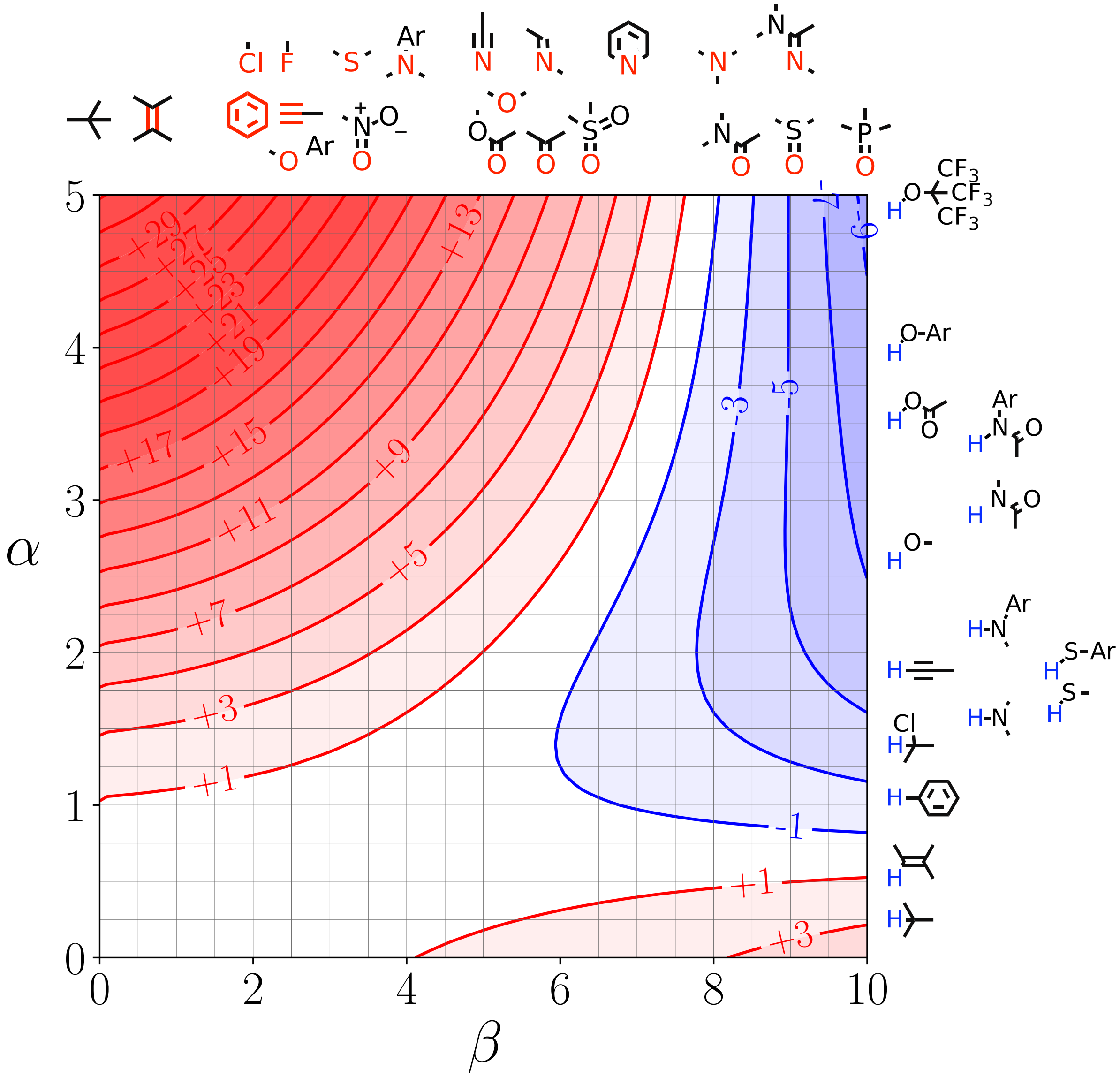

Figure S253: FGIP for tri-n-butylphosphate at 298K.

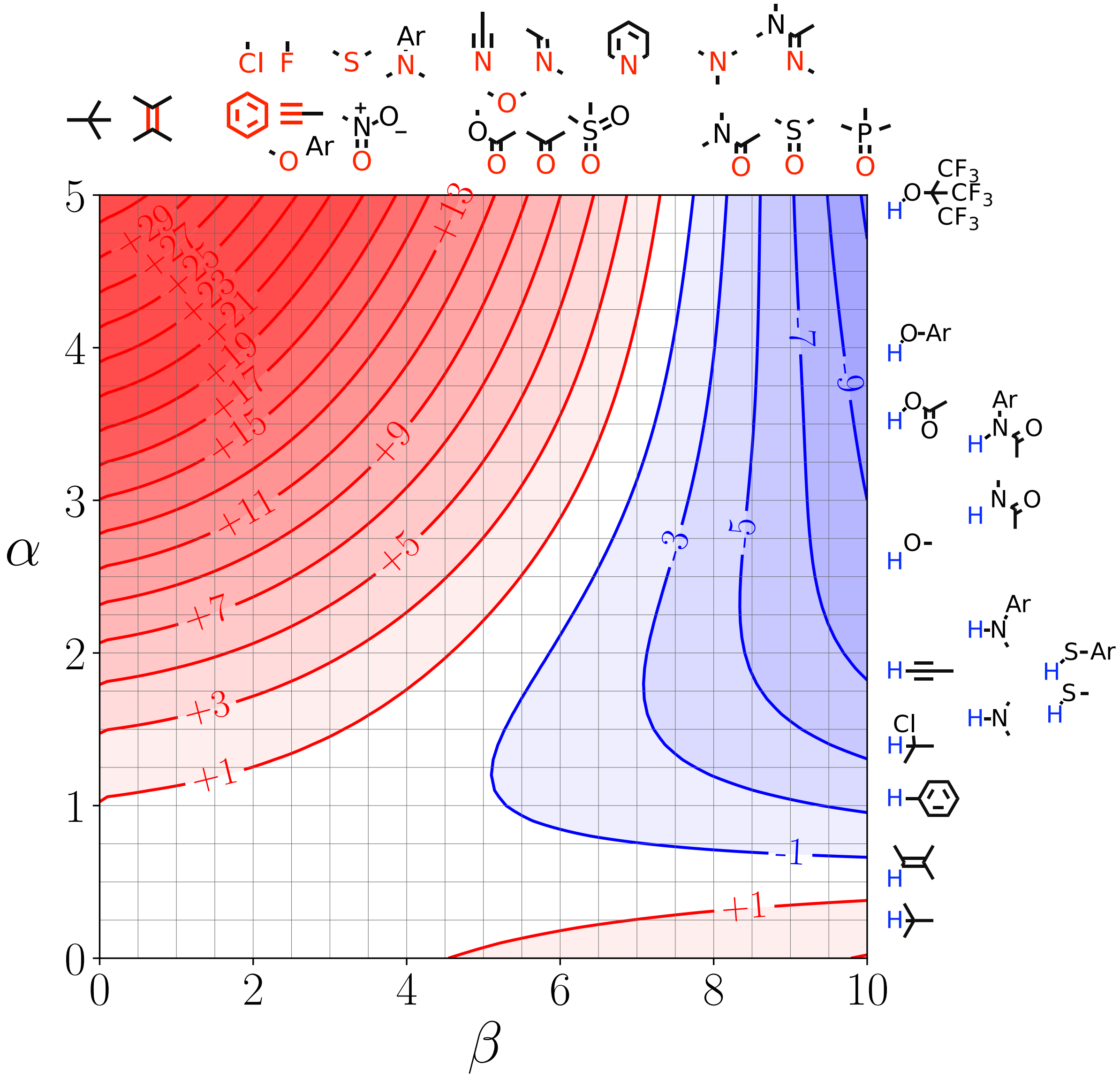

Figure S254: FGIP for hexamethylphosphoric triamide at 298K.

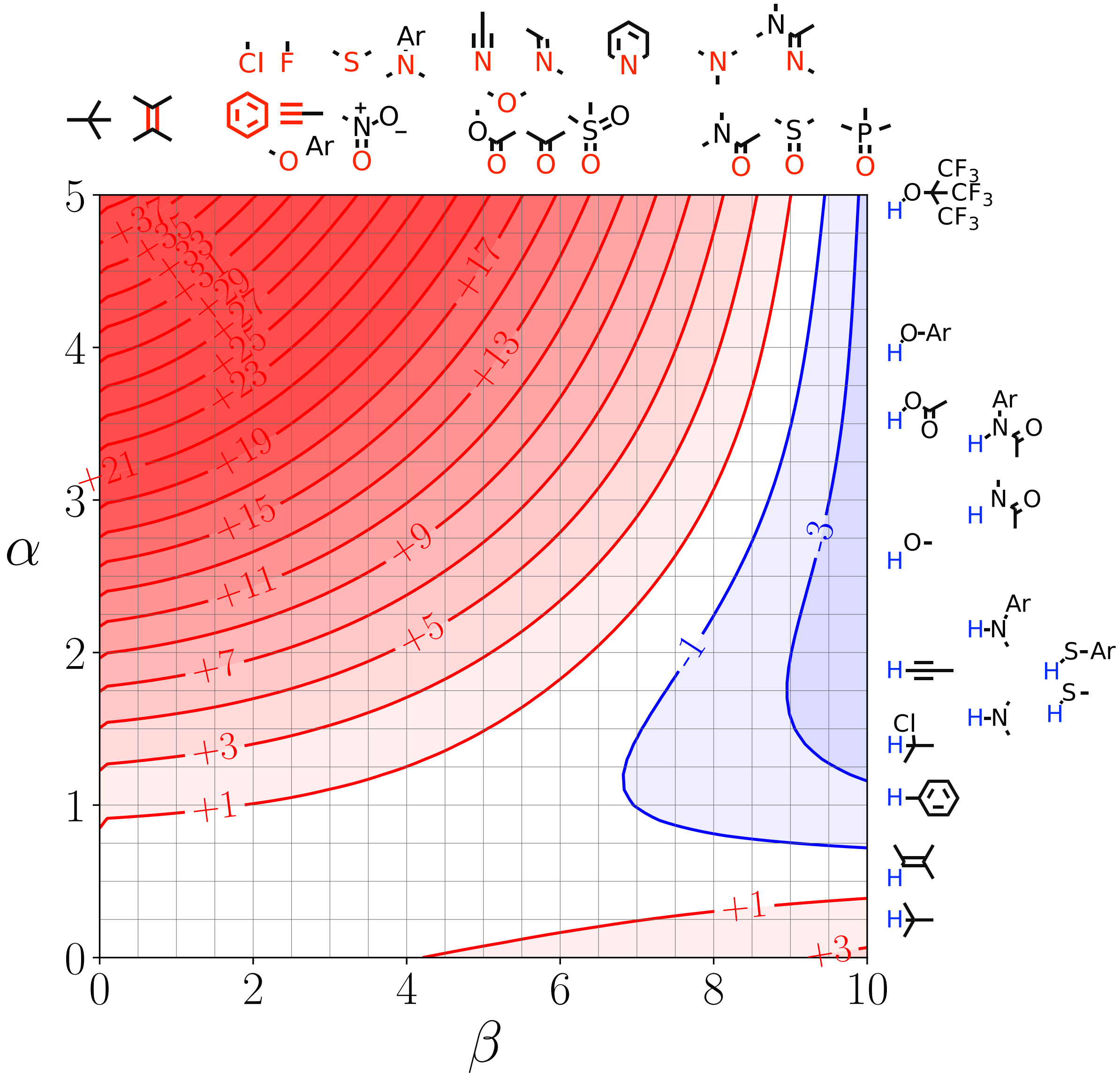

Figure S255: FGIP for hydrogen peroxide at 298K.

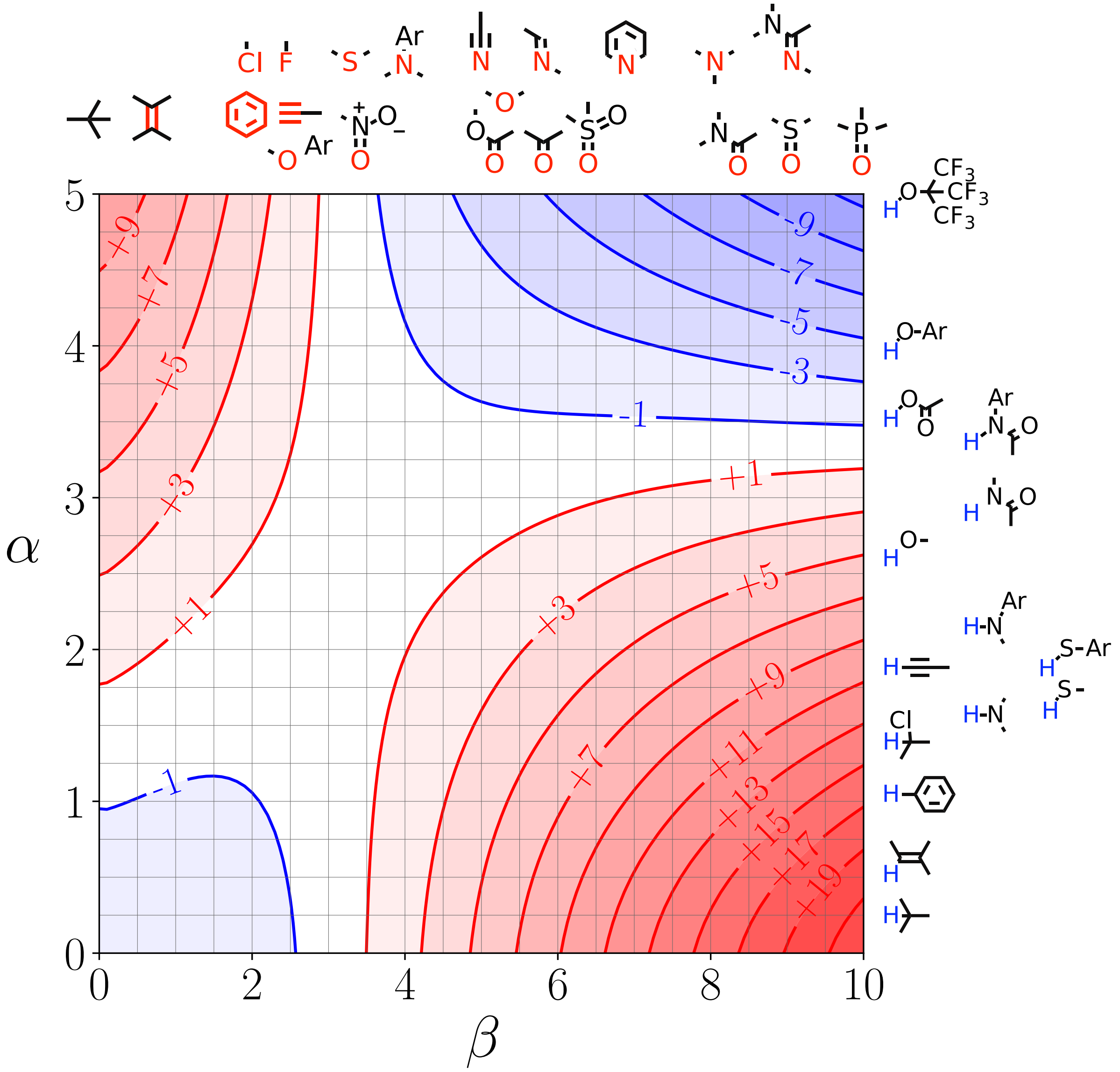

Figure S256: FGIP for hydrogen fluoride at 298K.

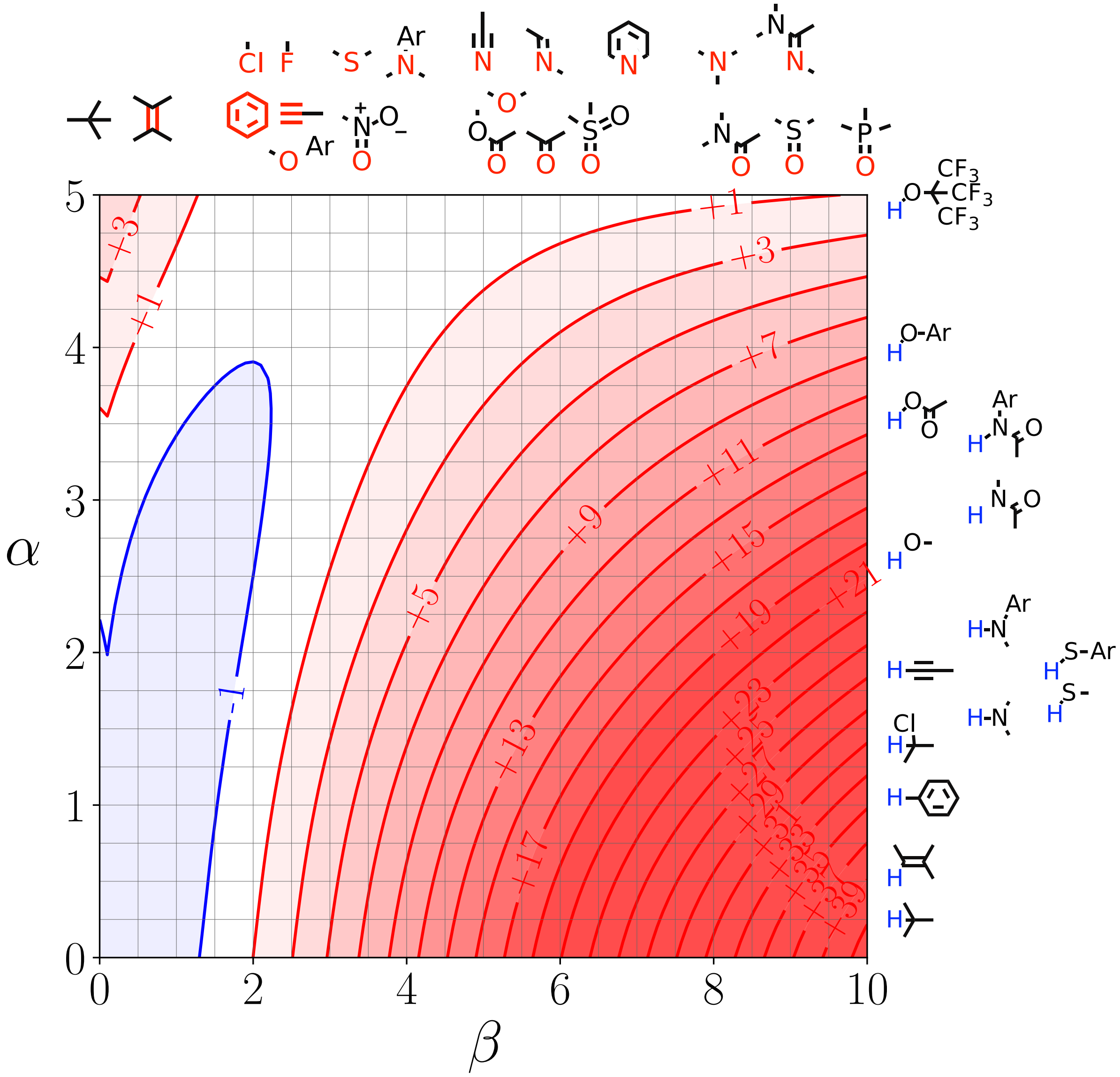

Figure S257: FGIP for sulfuric acid at 298K.

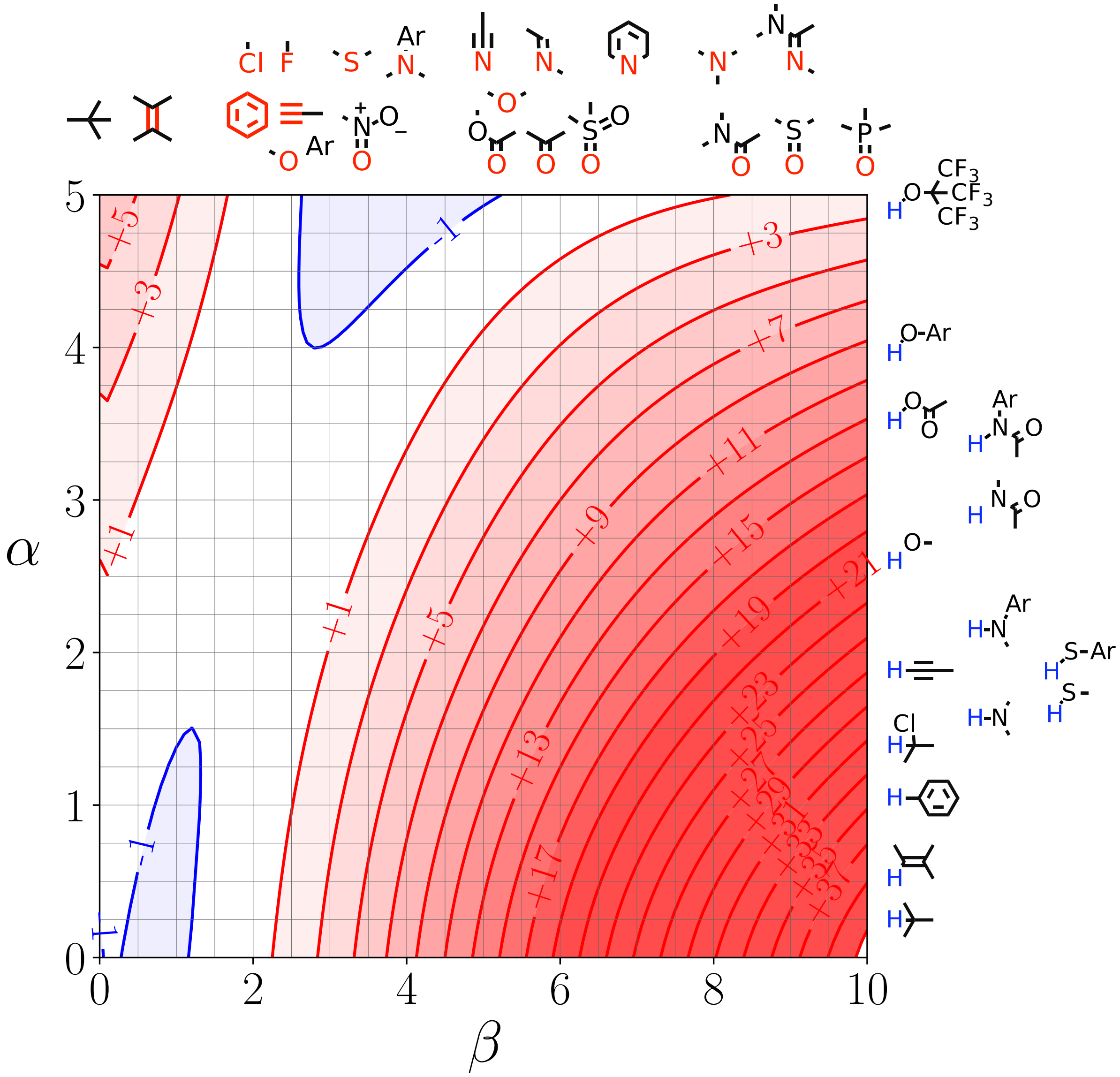

Figure S258: FGIP for ammonia at 298K.

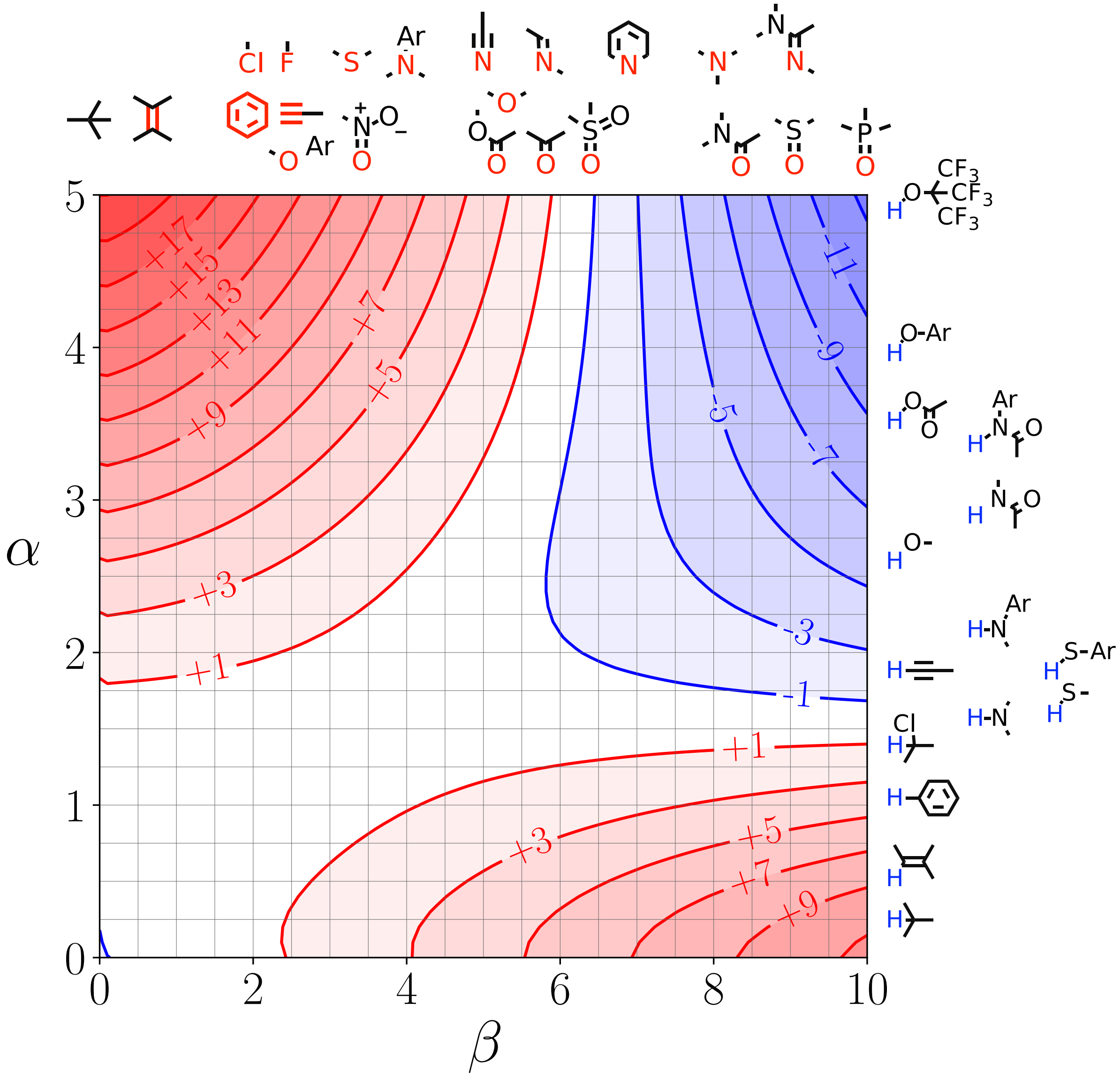

Figure S259: FGIP for hydrazine at 298K.

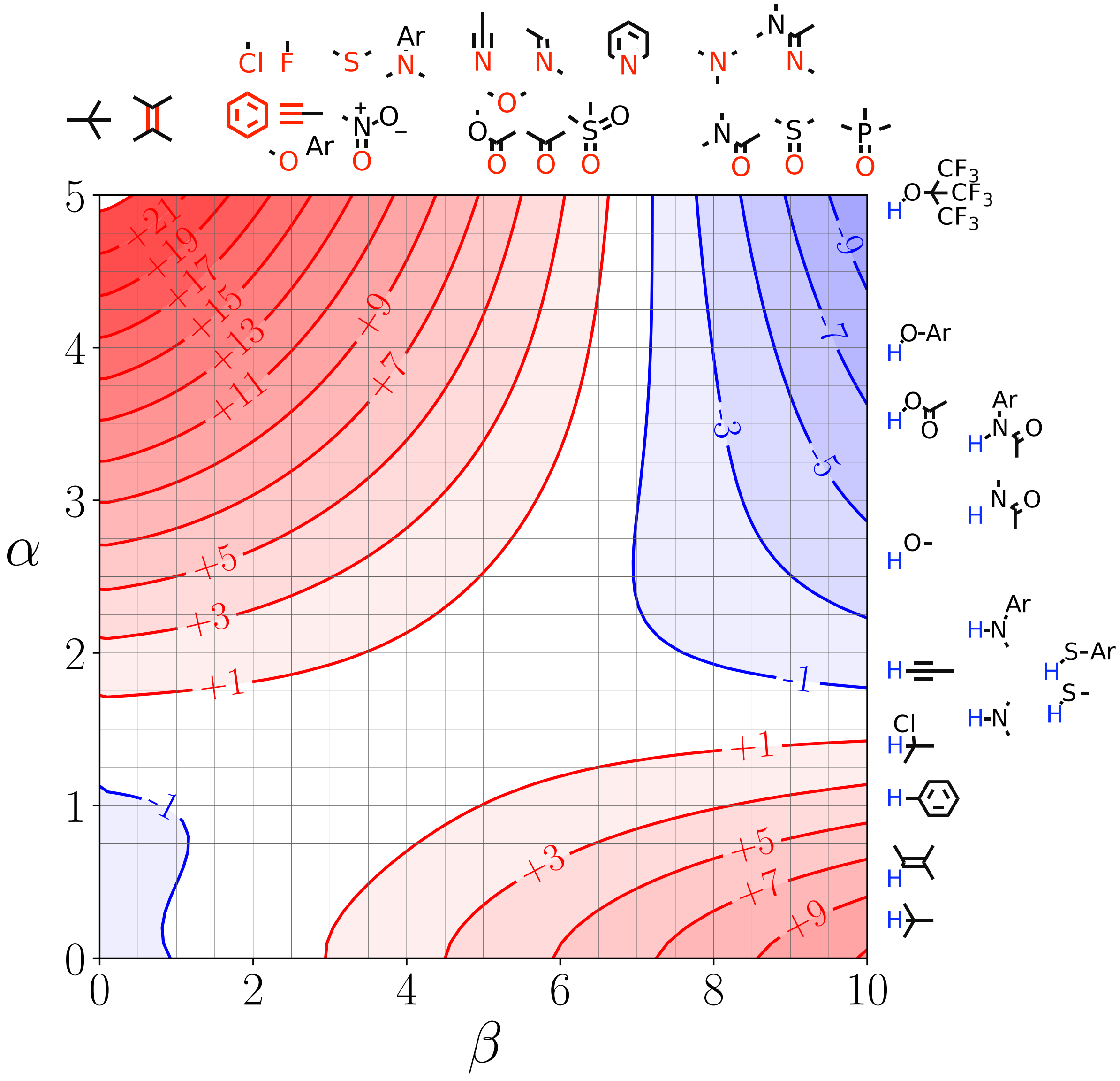

Figure S260: FGIP for sulfur dioxide at 298K.

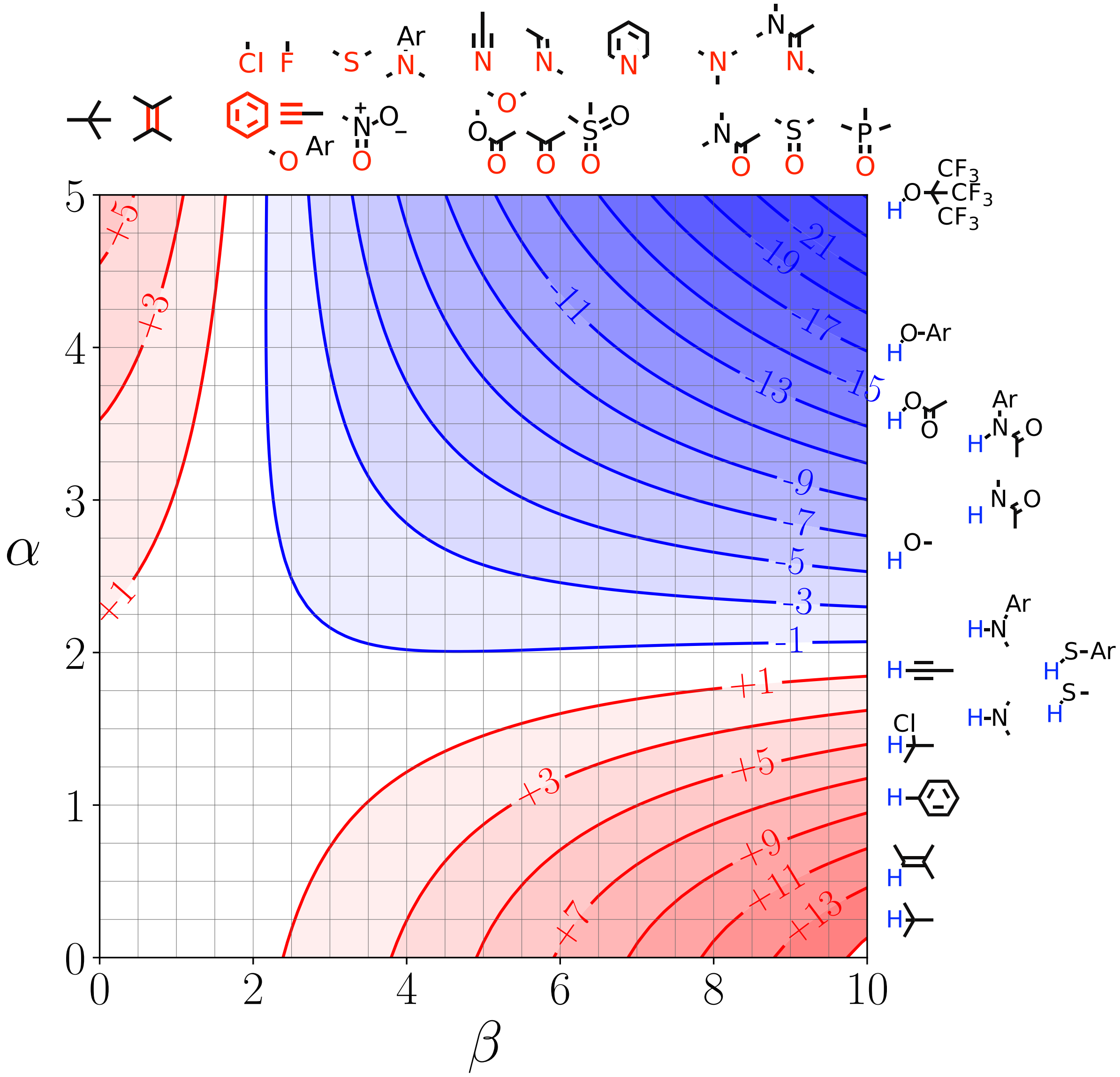

Figure S261: FGIP for thionyl chloride at 298K.

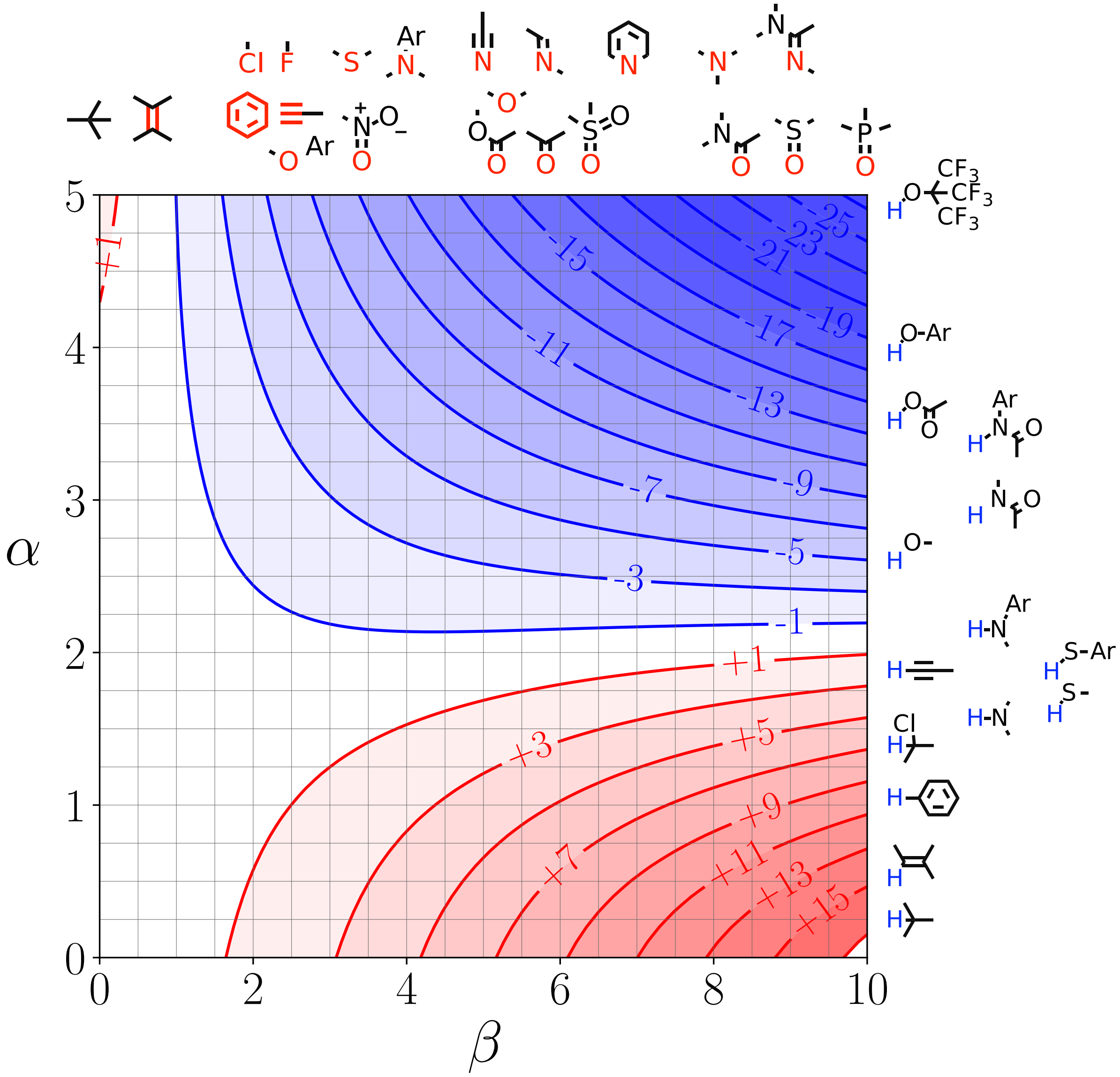

Figure S262: FGIP for phosphorus oxychloride at 298K.

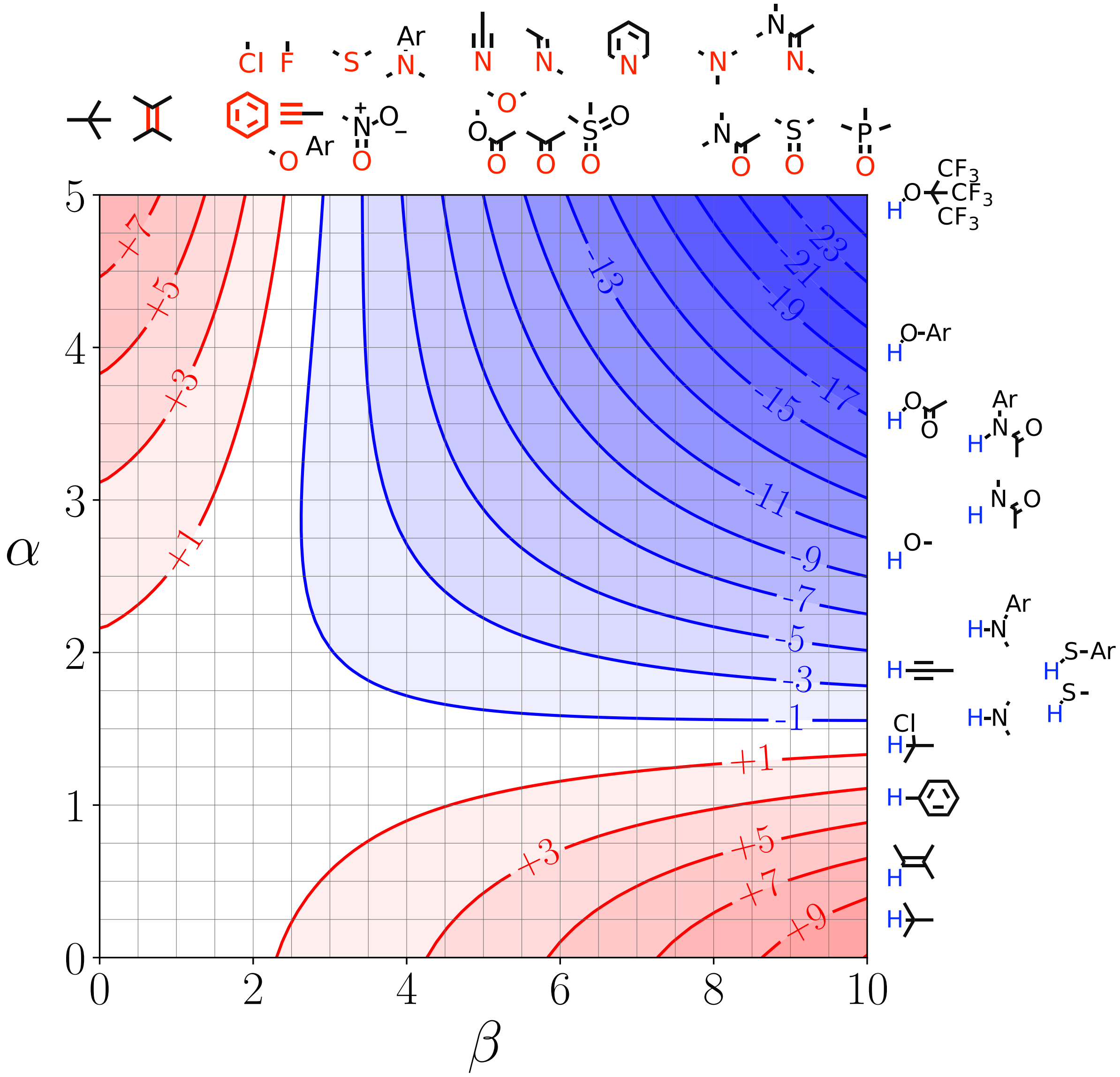

Figure S263: FGIP for dihydrolevoglucosenone at 298K.

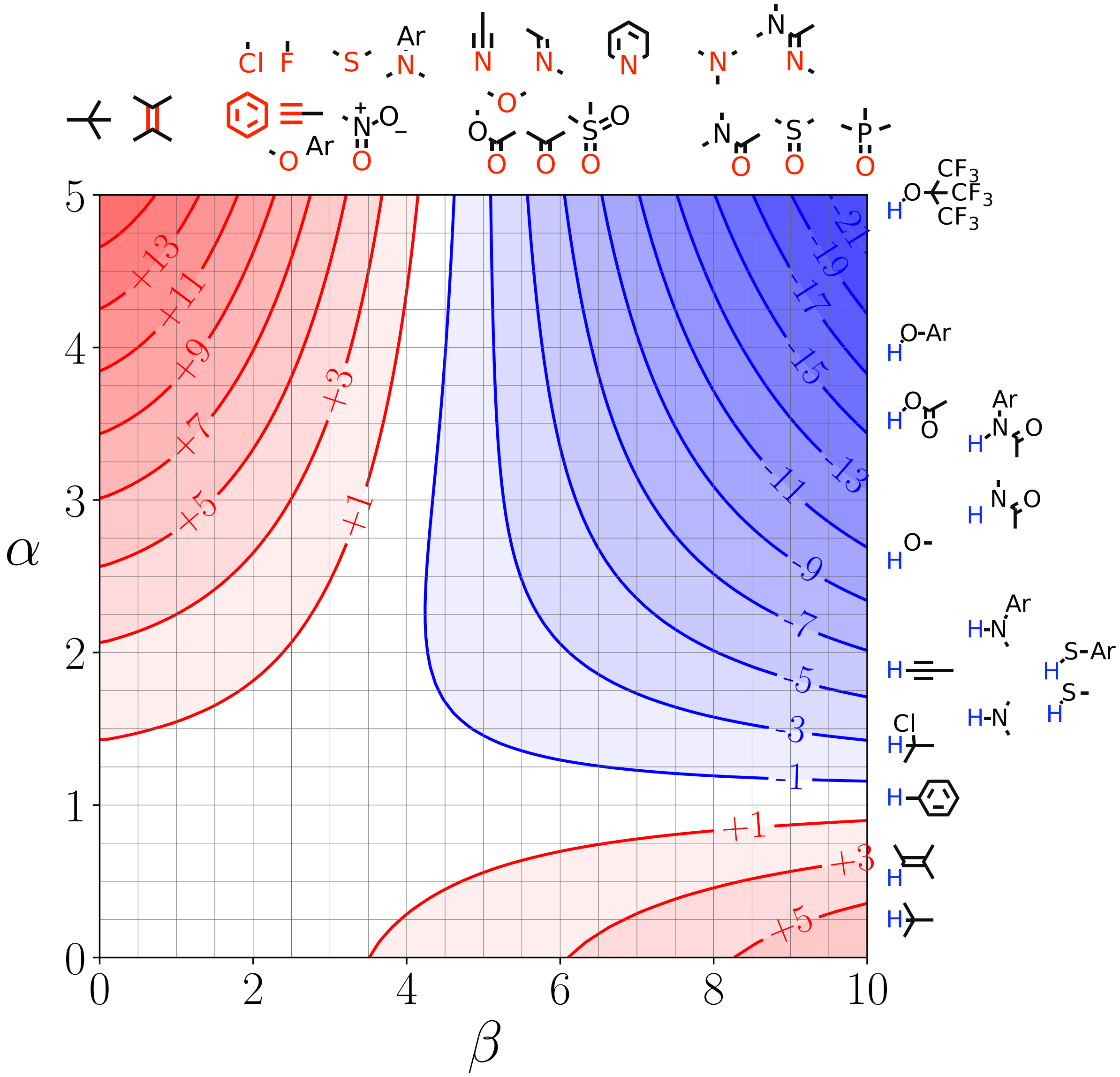

Volume fractions are used to express solvent concentrations for binary mixtures.

Figure S264: FGIP for 0.0% ethanol 100.0% water at 298K.

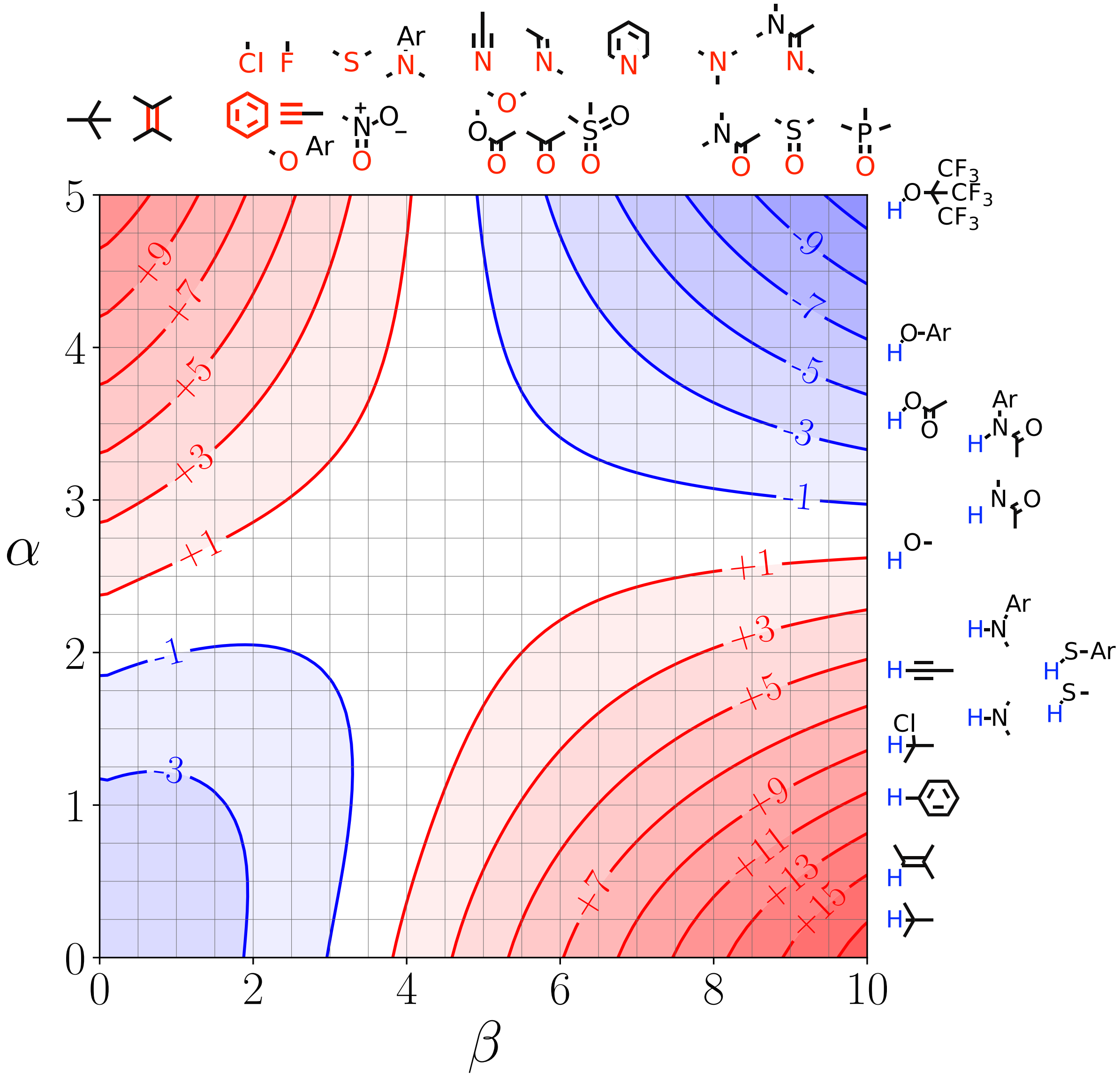

Figure S265: FGIP for 5.0% ethanol 95.0% water at 298K.

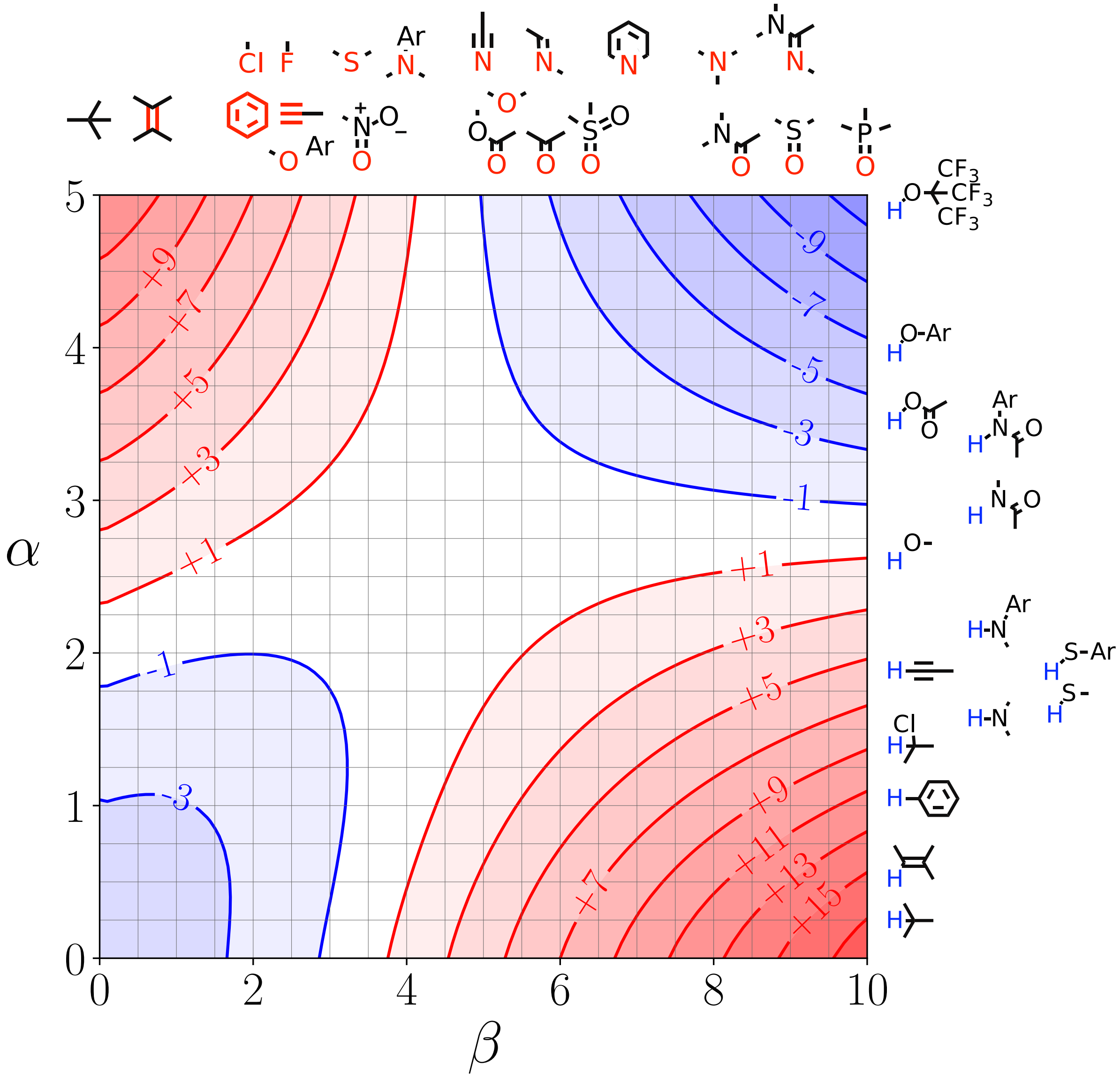

Figure S266: FGIP for 10.0% ethanol 90.0% water at 298K.

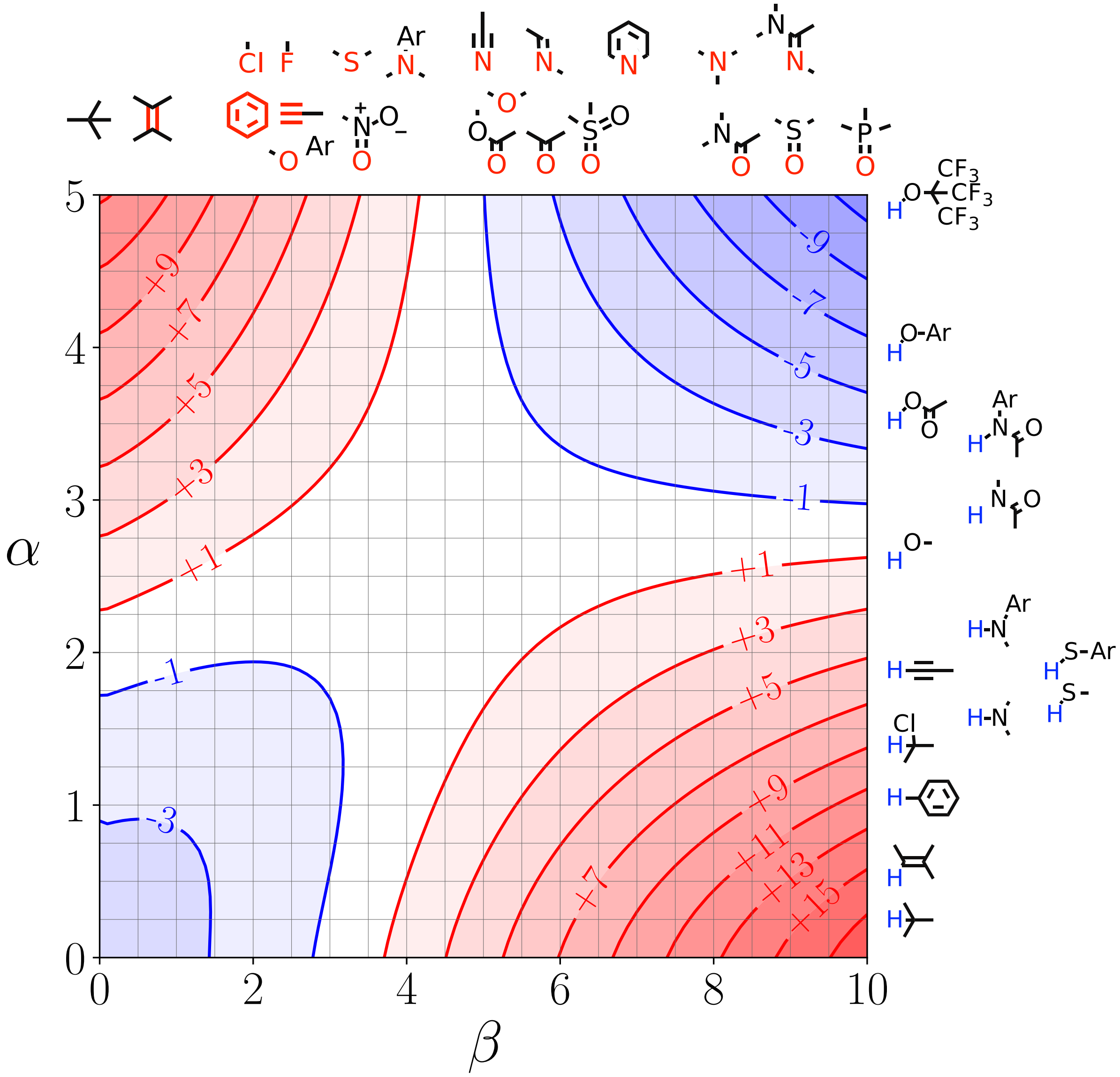

Figure S267: FGIP for 15.0% ethanol 85.0% water at 298K.

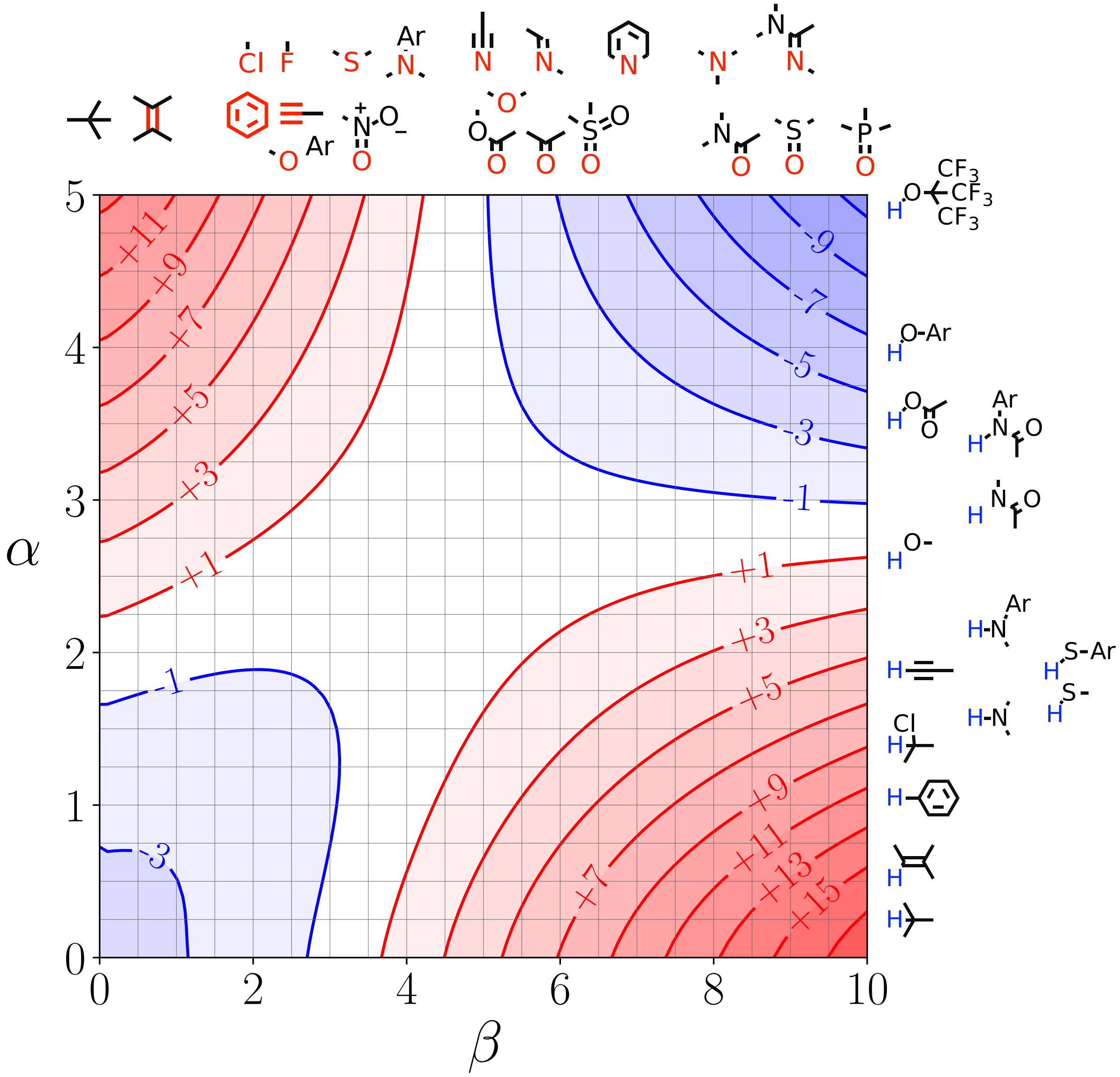

Figure S268: FGIP for 20.0% ethanol 80.0% water at 298K.

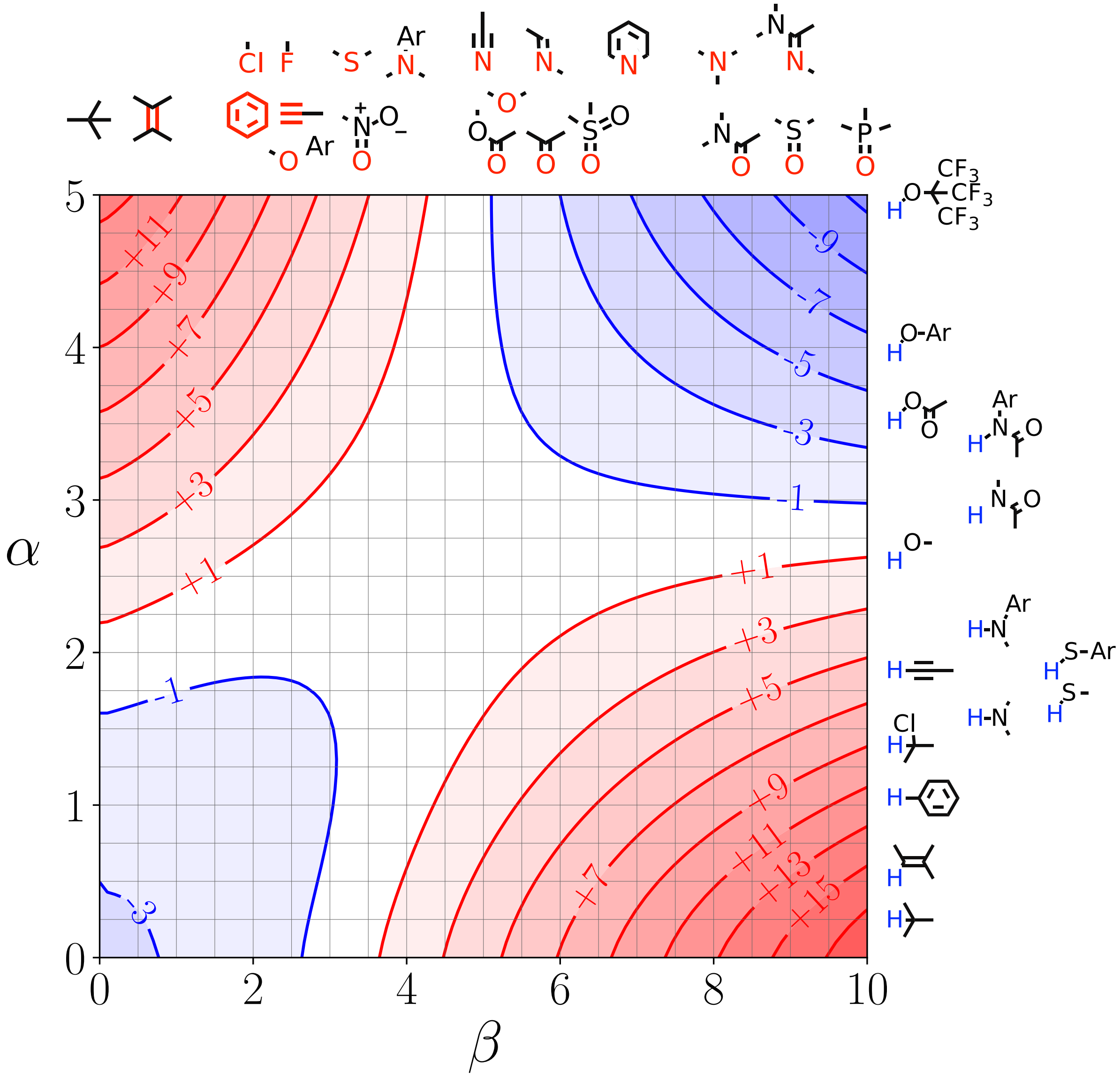

Figure S269: FGIP for 25.0% ethanol 75.0% water at 298K.

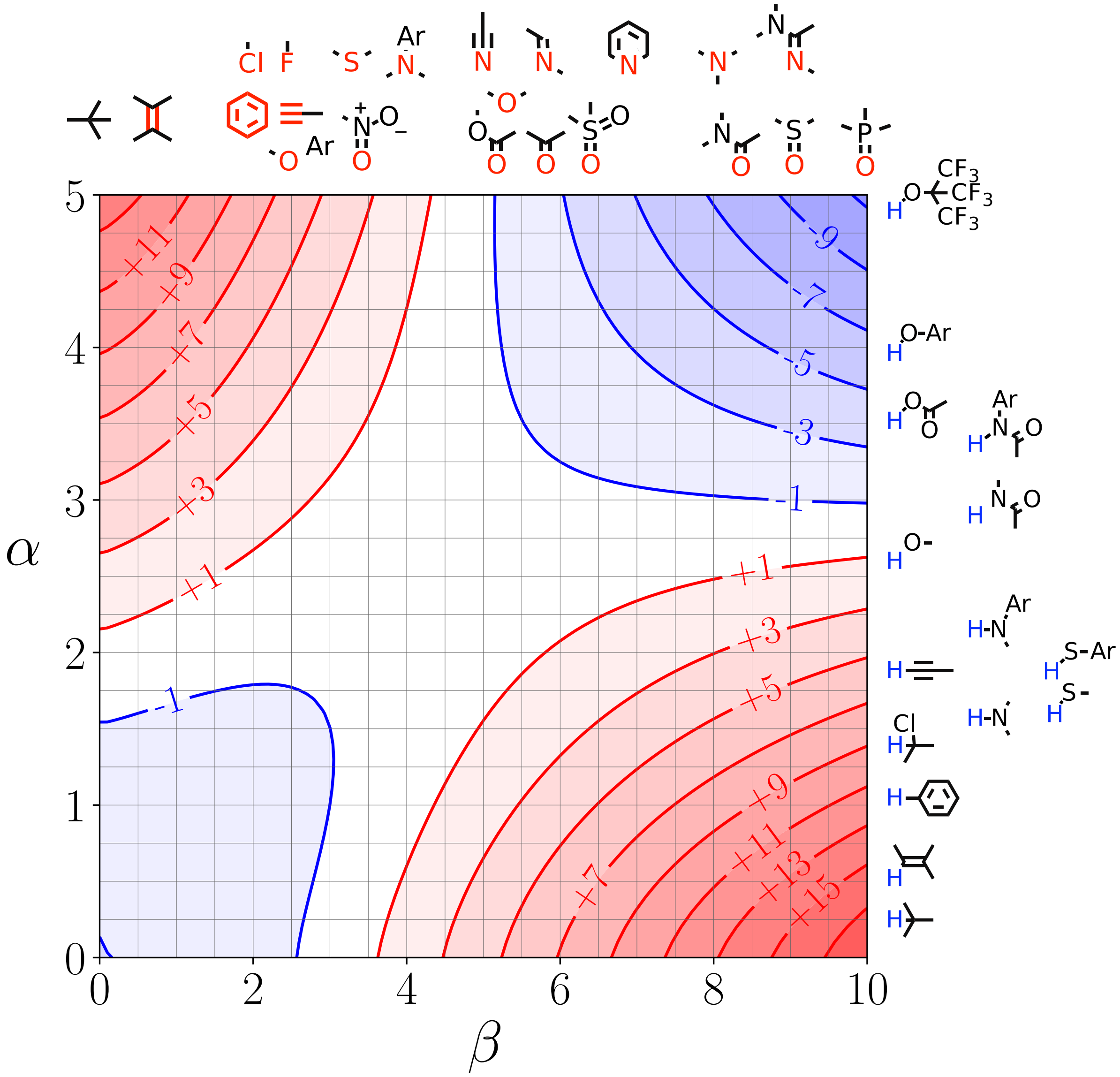

Figure S270: FGIP for 30.0% ethanol 70.0% water at 298K.

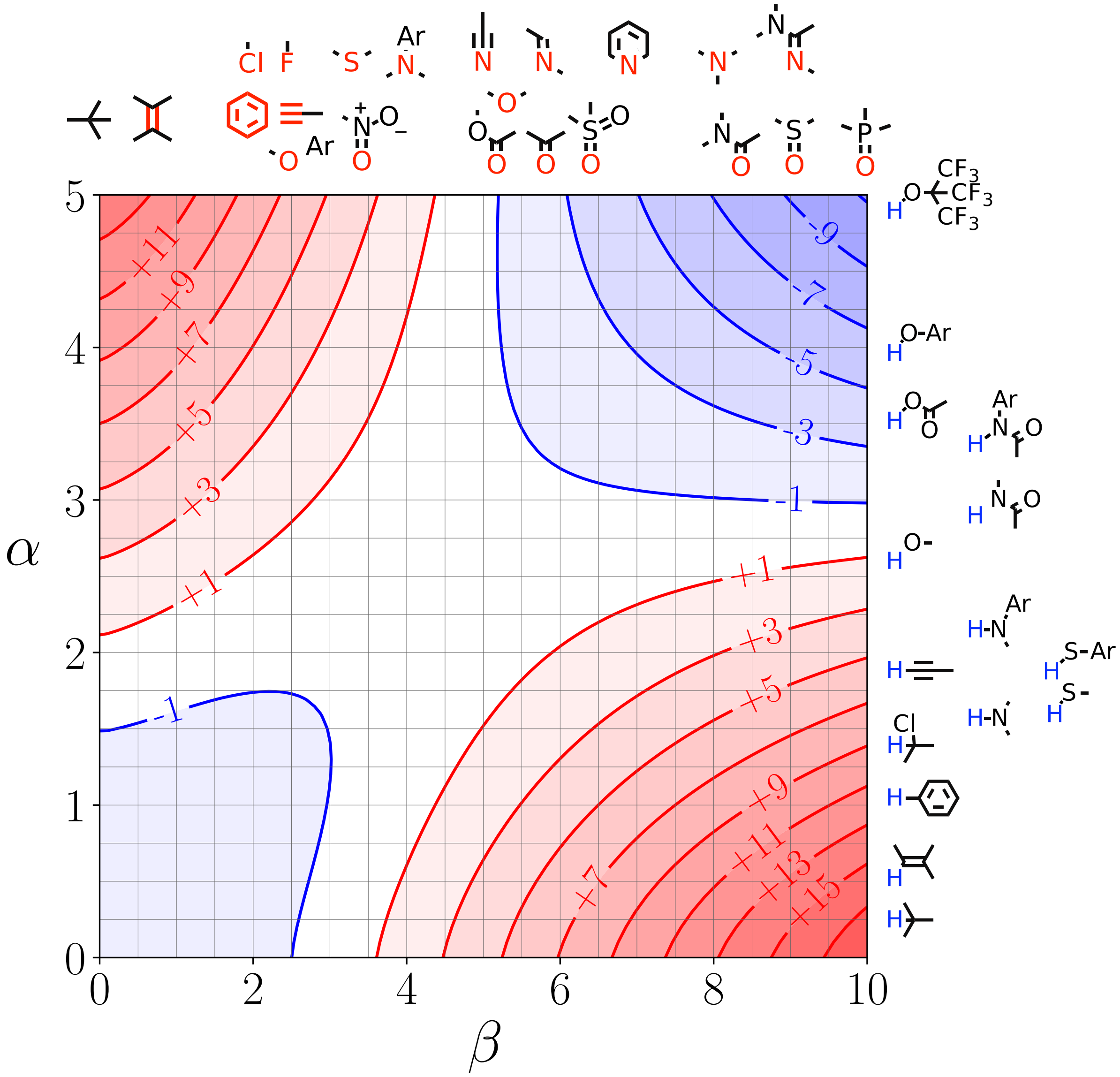

Figure S271: FGIP for 35.0% ethanol 65.0% water at 298K.

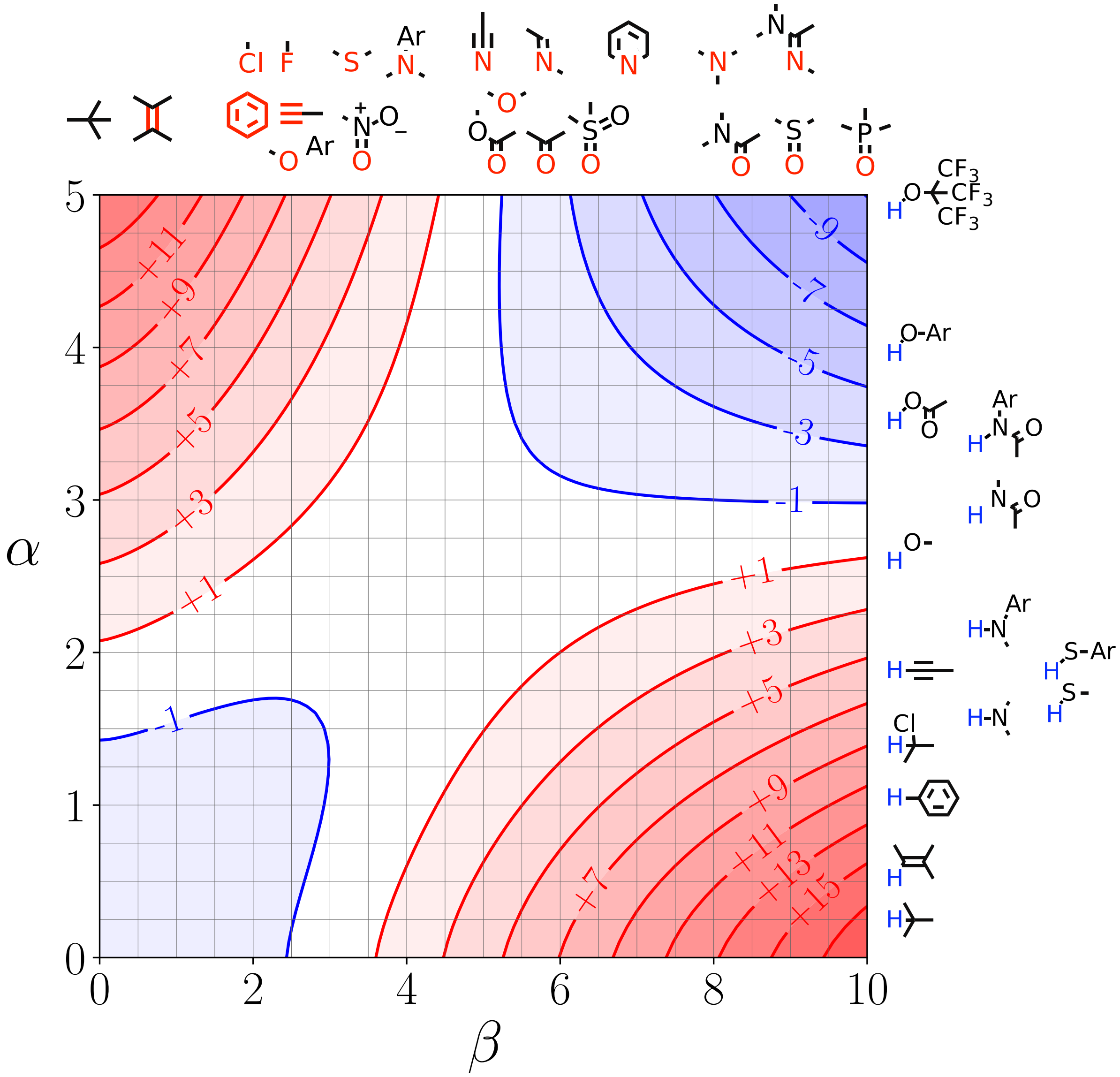

Figure S272: FGIP for 40.0% ethanol 60.0% water at 298K.

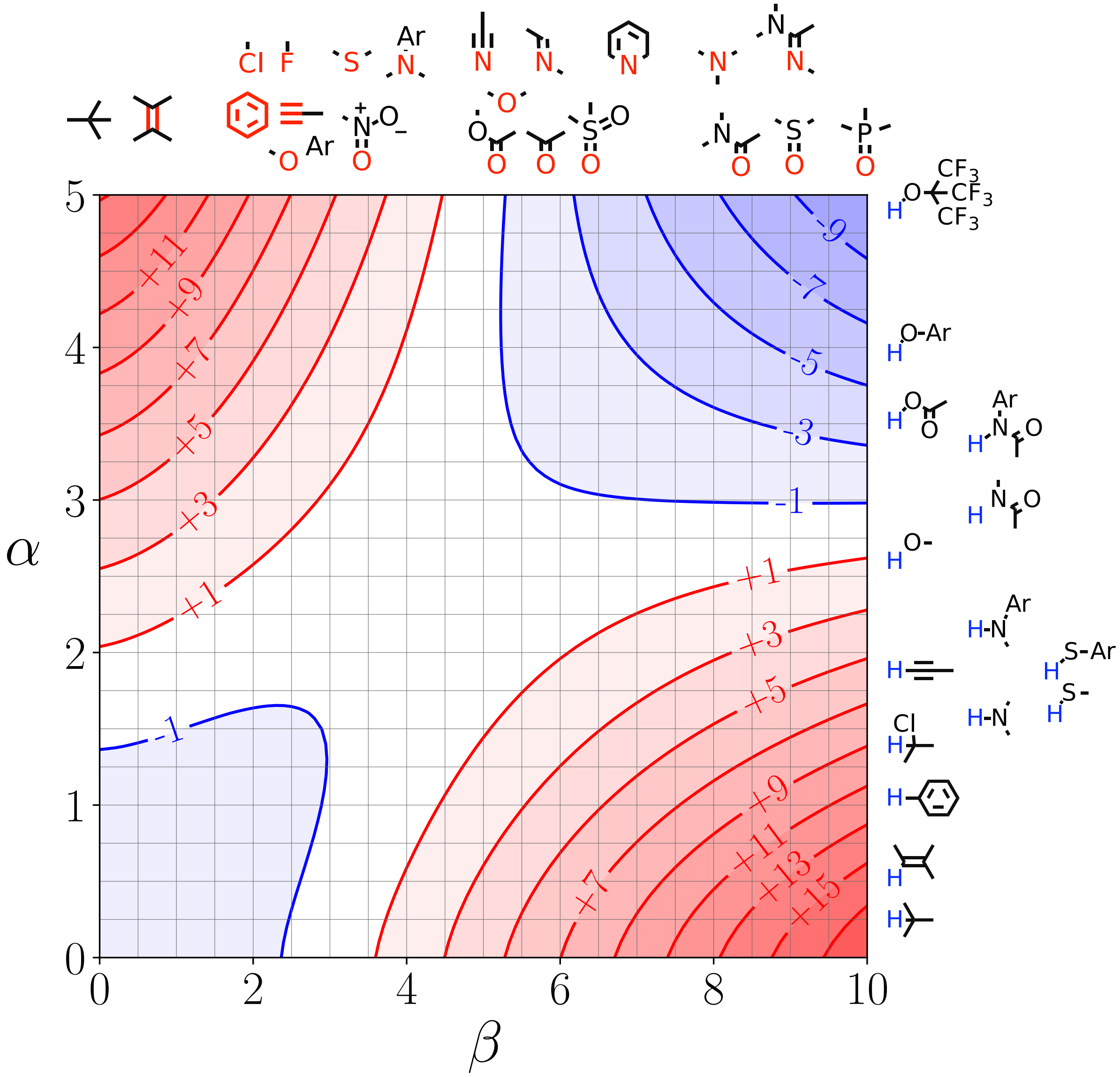

Figure S273: FGIP for 45.0% ethanol 55.0% water at 298K.

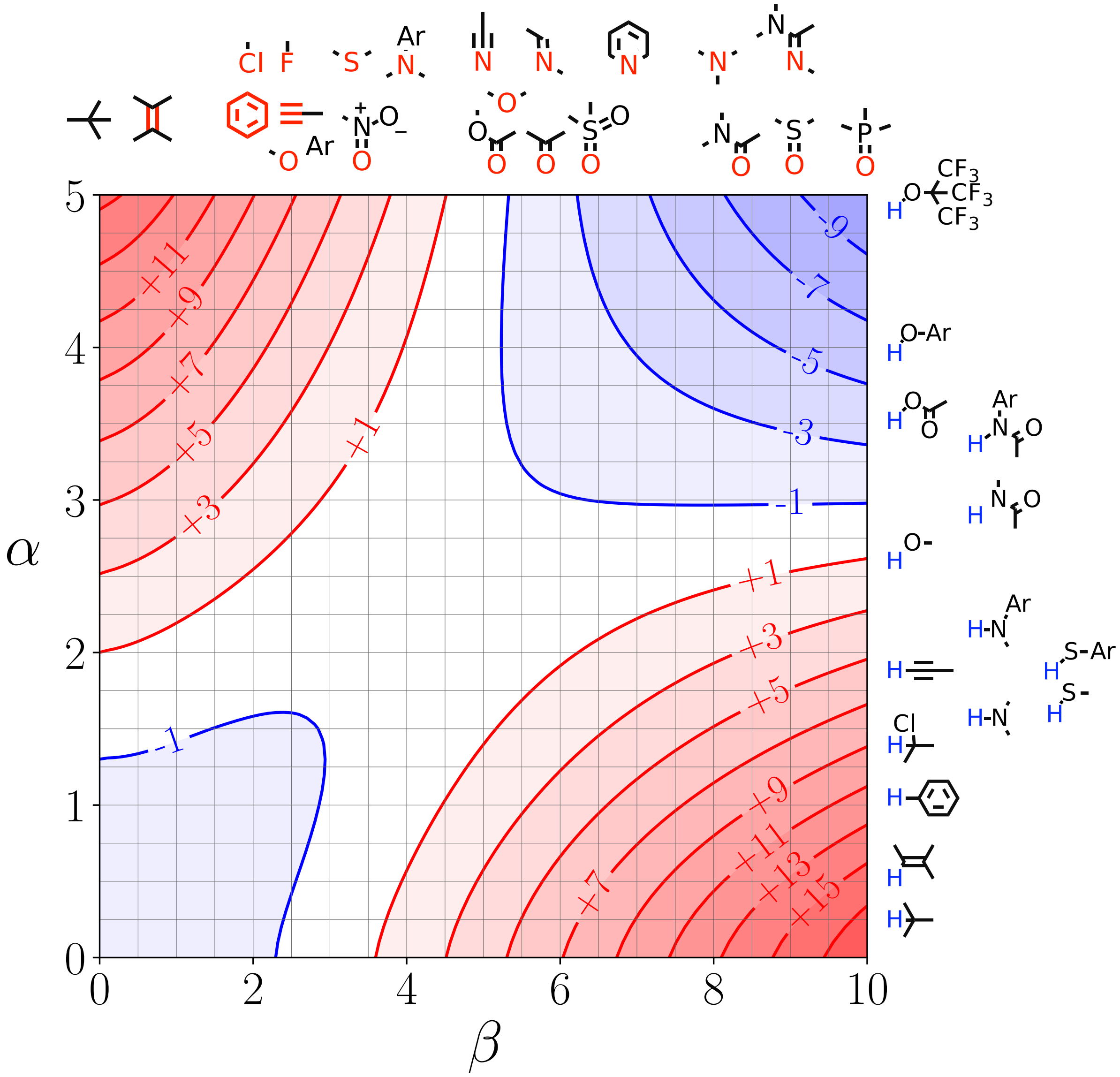

Figure S274: FGIP for 50.0% ethanol 50.0% water at 298K.

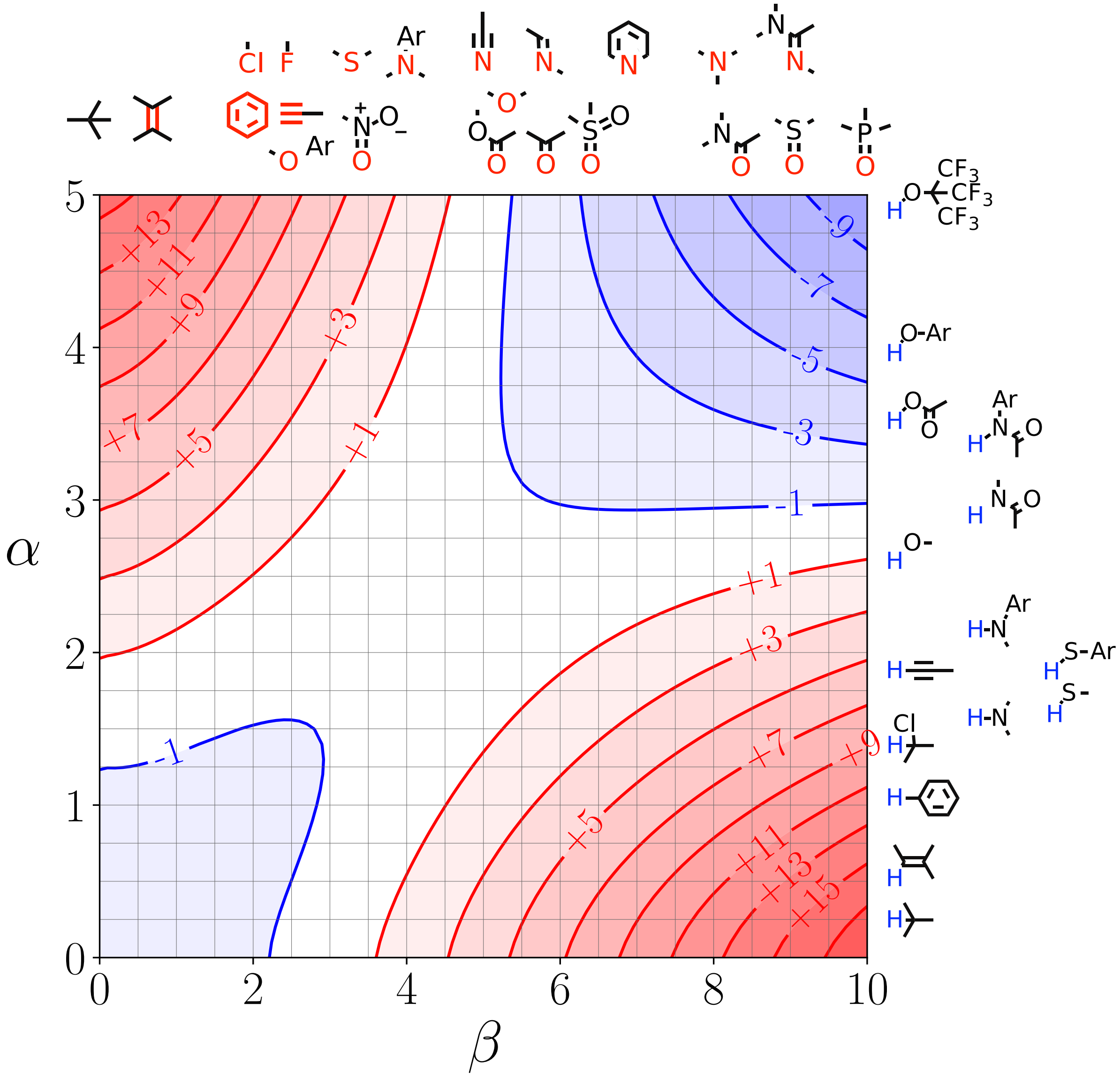

Figure S275: FGIP for 55.0% ethanol 45.0% water at 298K.

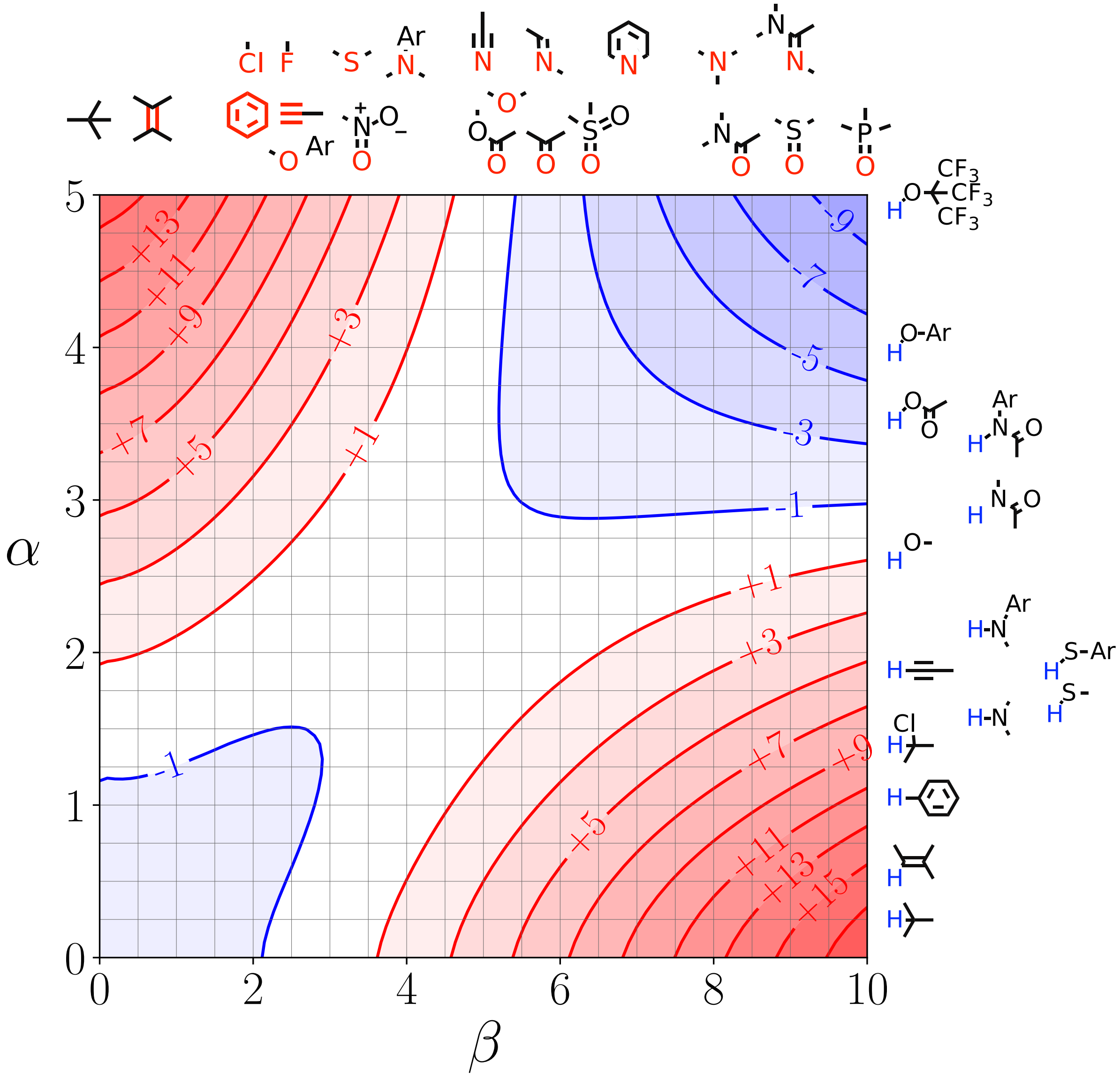

Figure S276: FGIP for 60.0% ethanol 40.0% water at 298K.

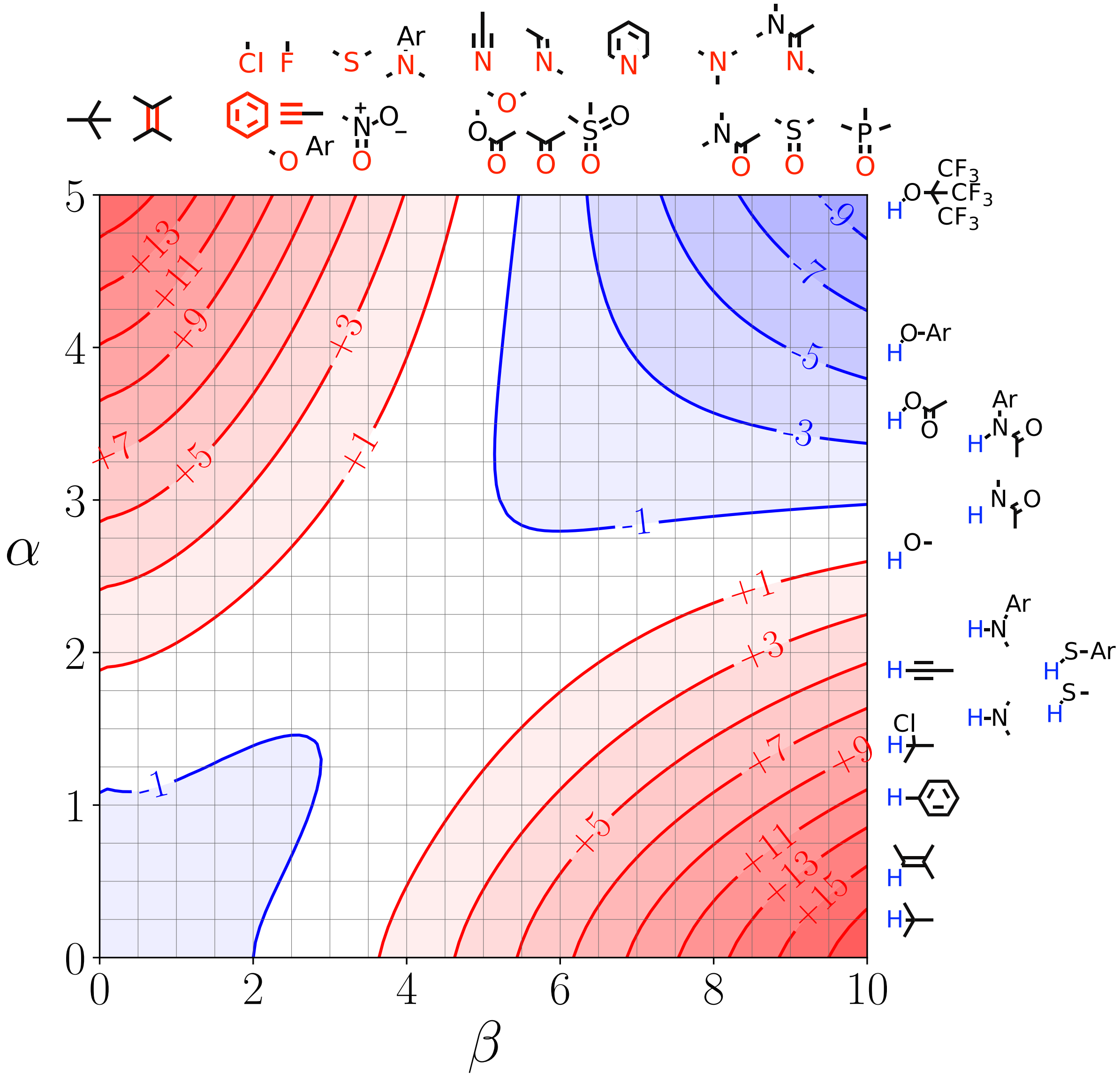

Figure S277: FGIP for 65.0% ethanol 35.0% water at 298K.

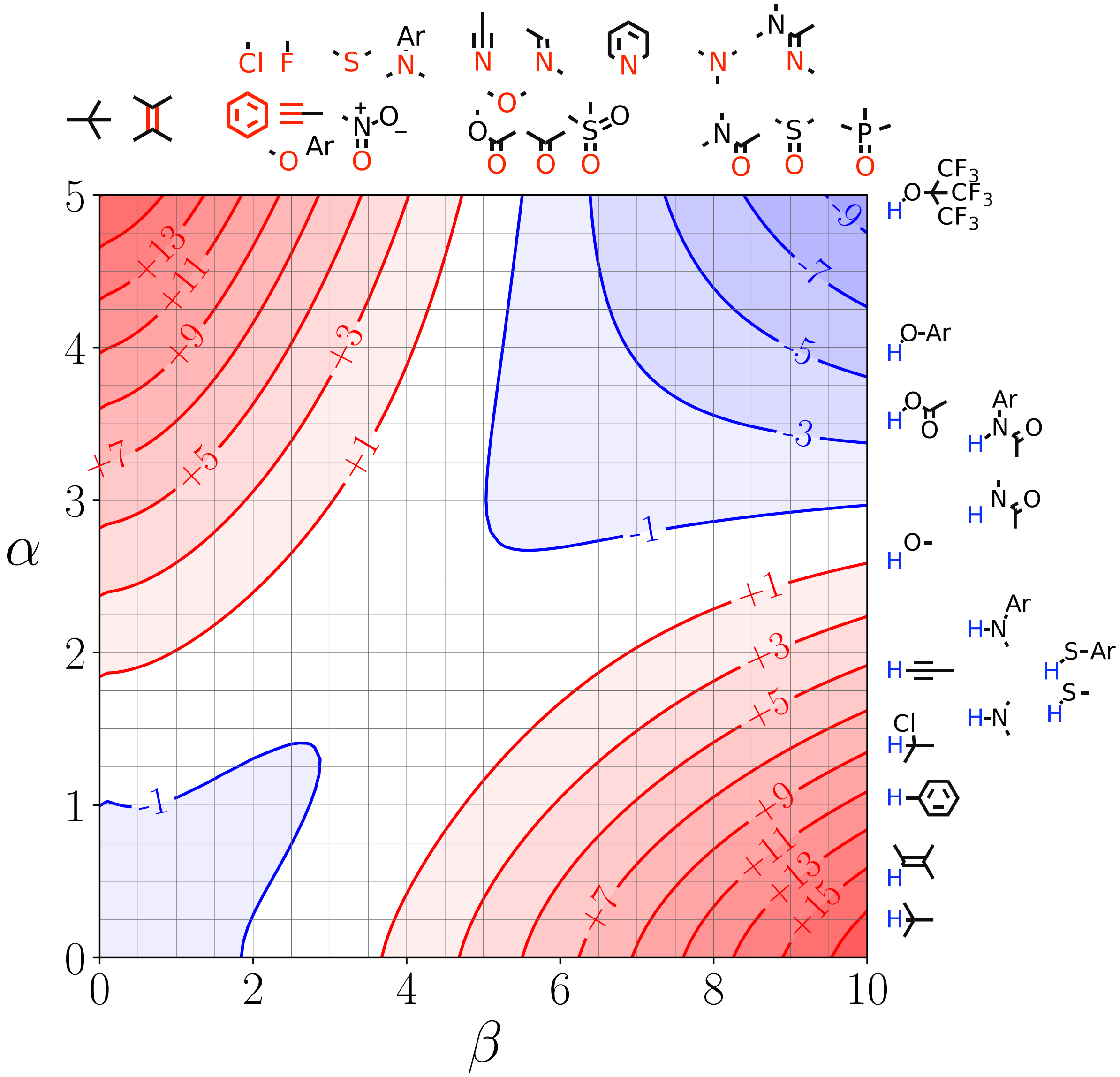

Figure S278: FGIP for 70.0% ethanol 30.0% water at 298K.

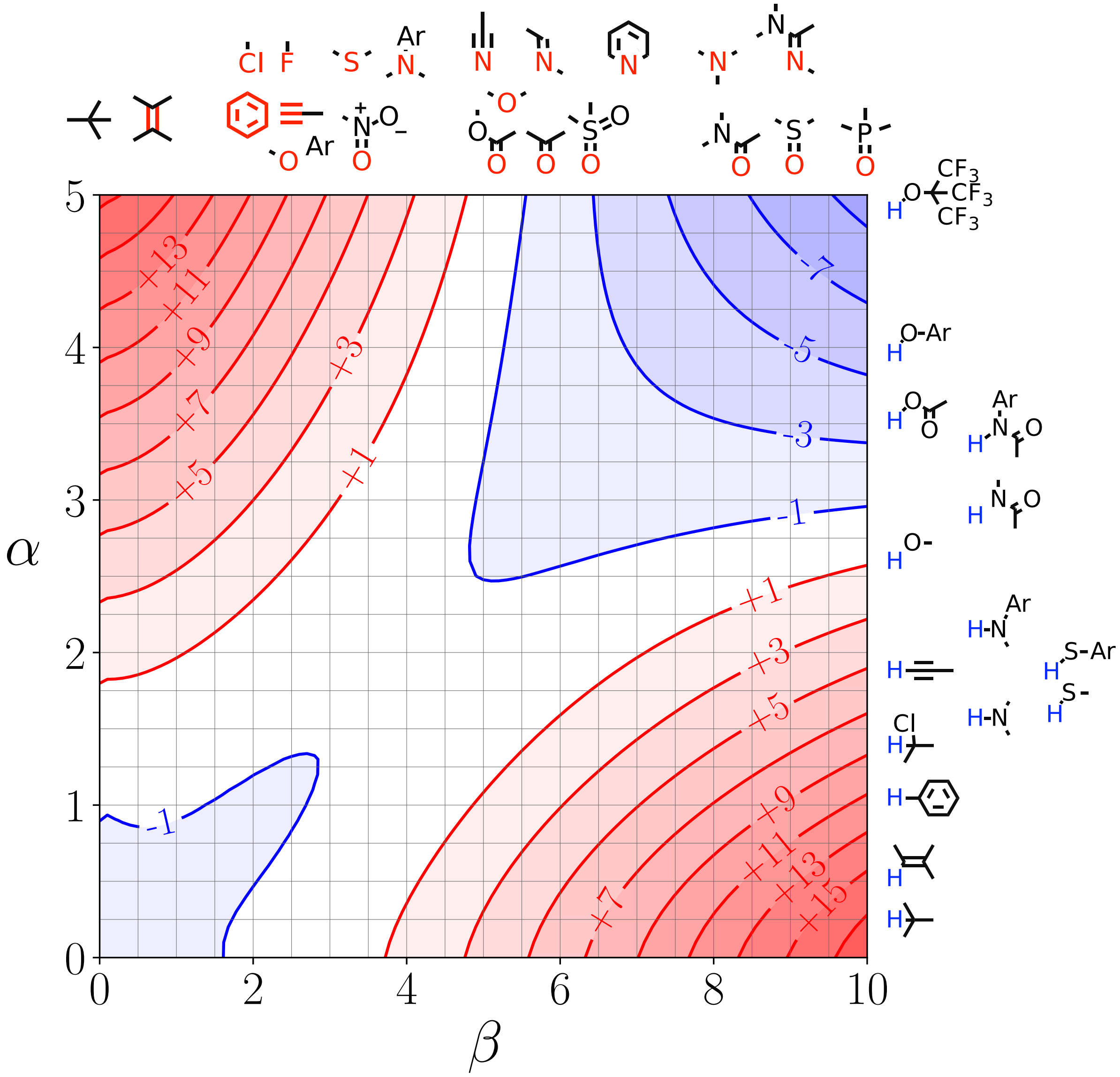

Figure S279: FGIP for 75.0% ethanol 25.0% water at 298K.

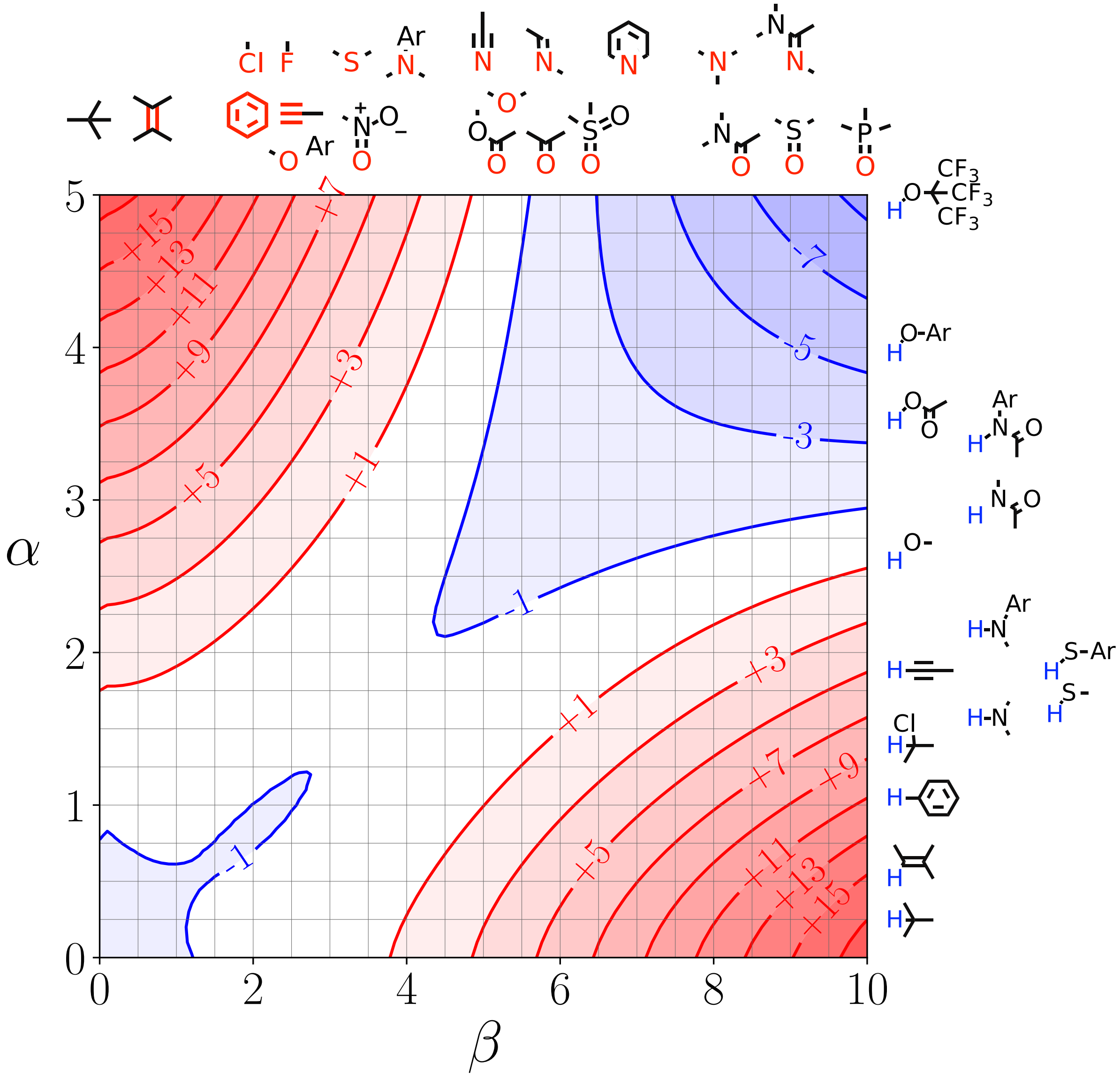

Figure S280: FGIP for 80.0% ethanol 20.0% water at 298K.

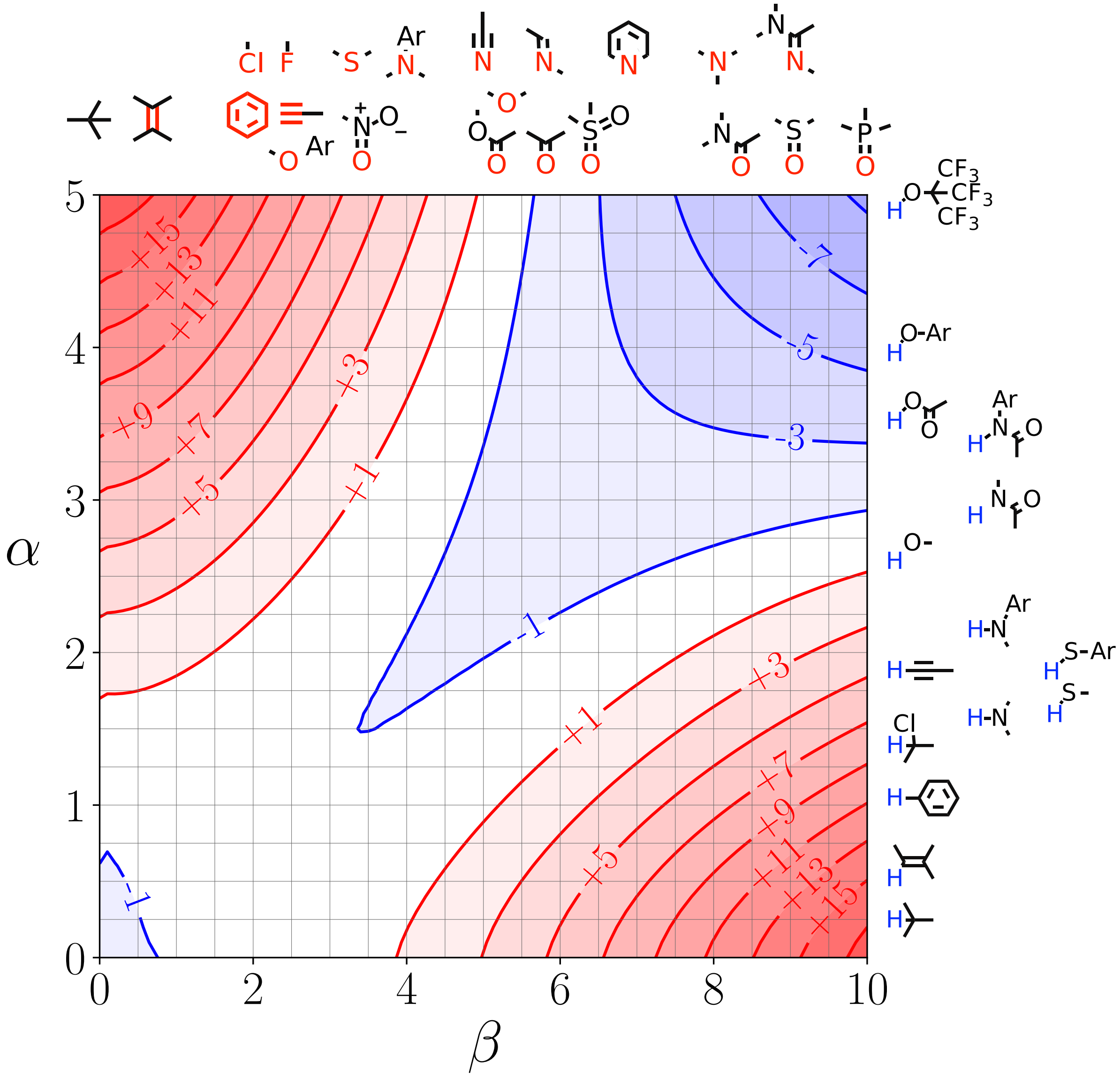

Figure S281: FGIP for 85.0% ethanol 15.0% water at 298K.

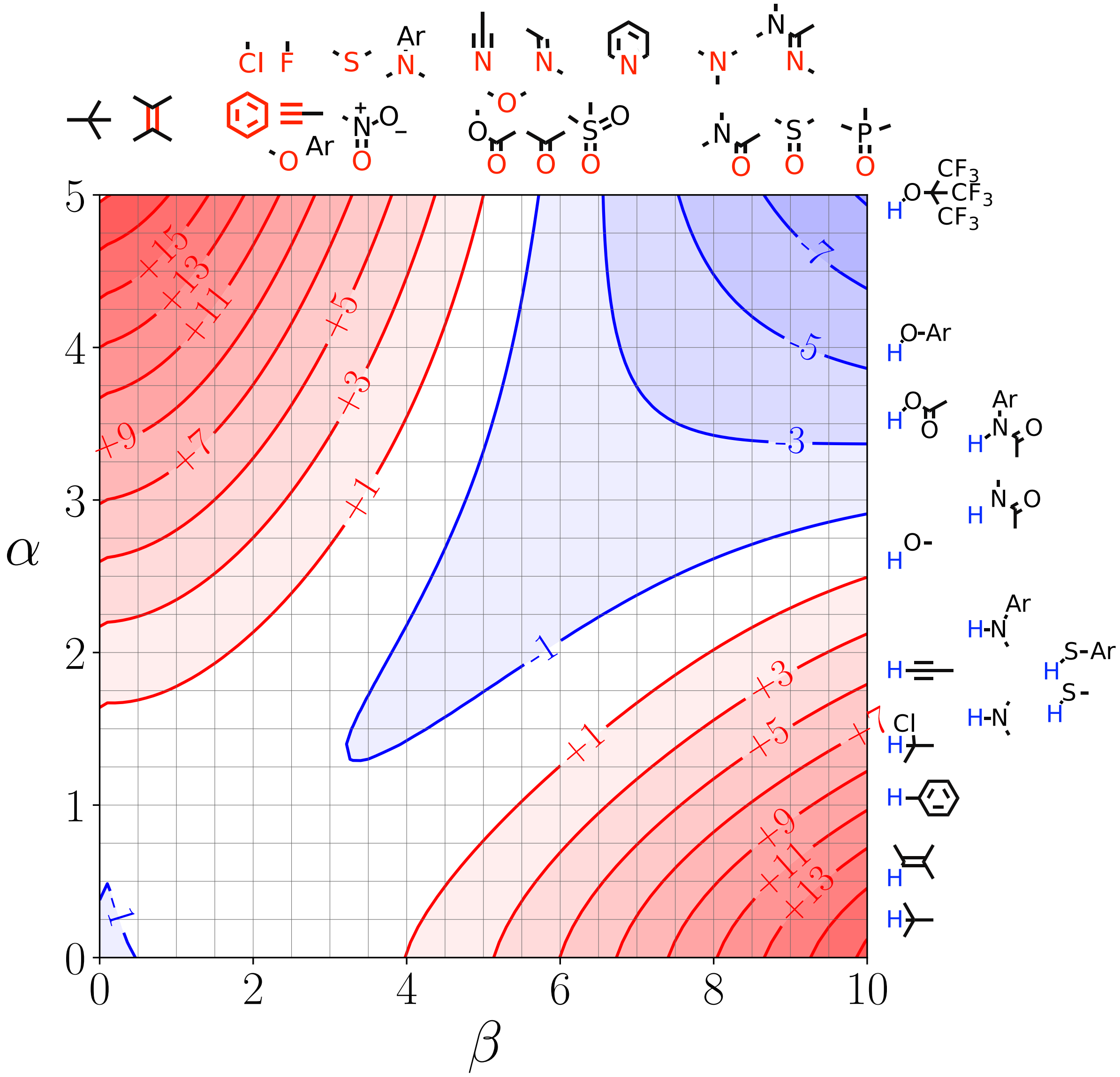

Figure S282: FGIP for 90.0% ethanol 10.0% water at 298K.

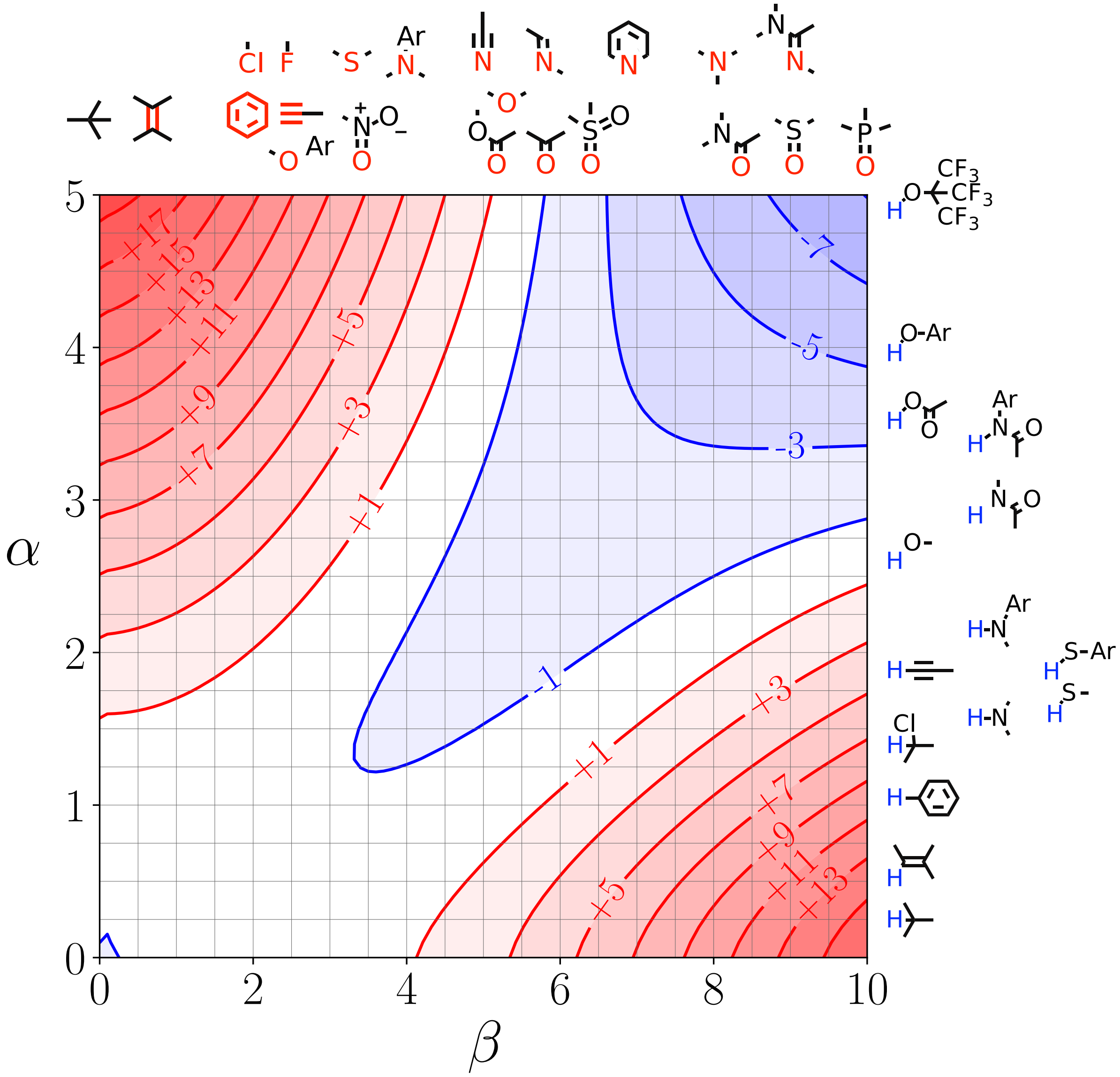

Figure S283: FGIP for 95.0% ethanol 5.0% water at 298K.

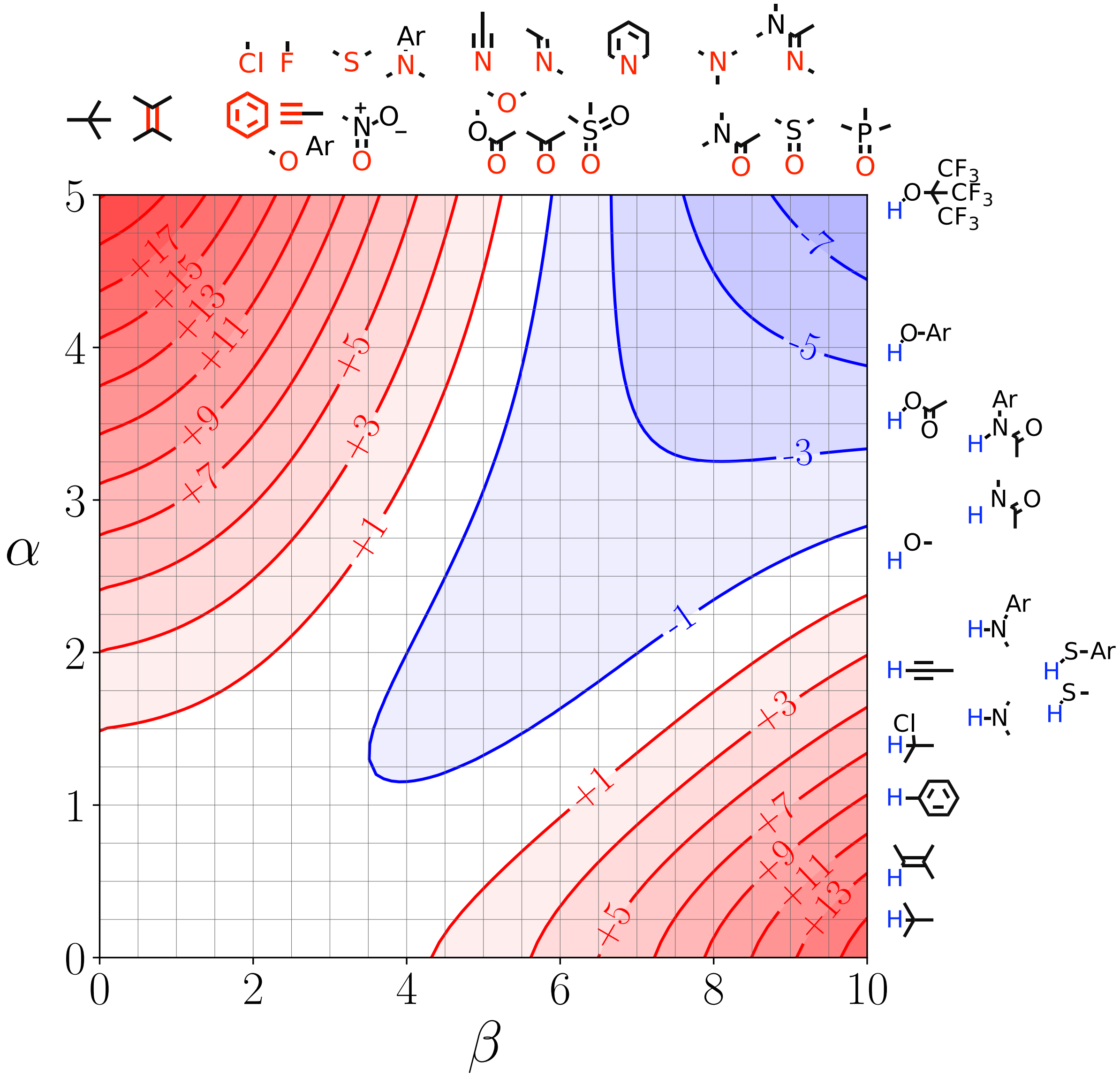

Figure S284: FGIP for 100.0% ethanol 0.0% water at 298K.

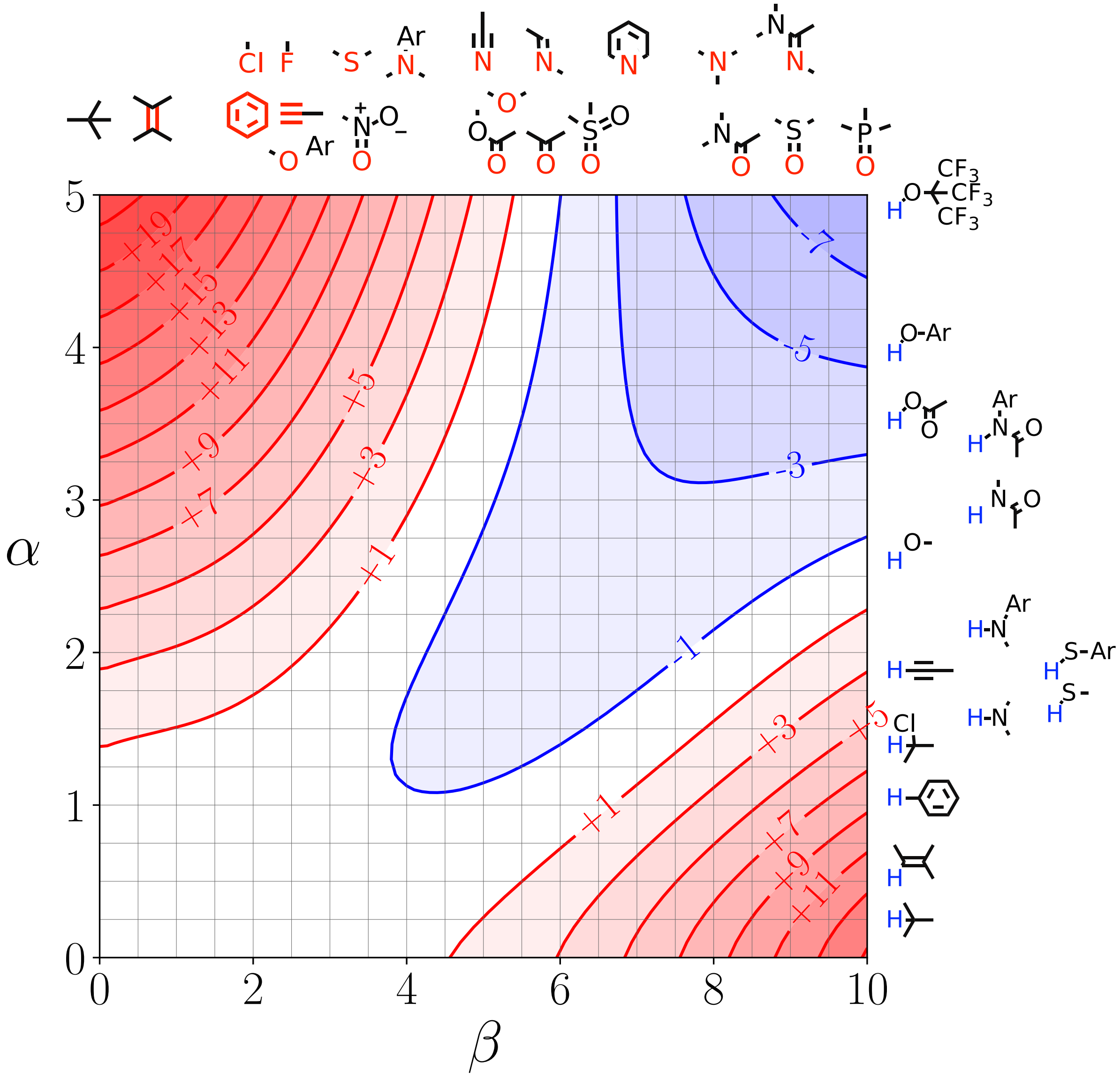

Figure S285: FGIP for 0.0% chloroform 100.0% tetrahydrofuran at 298K.

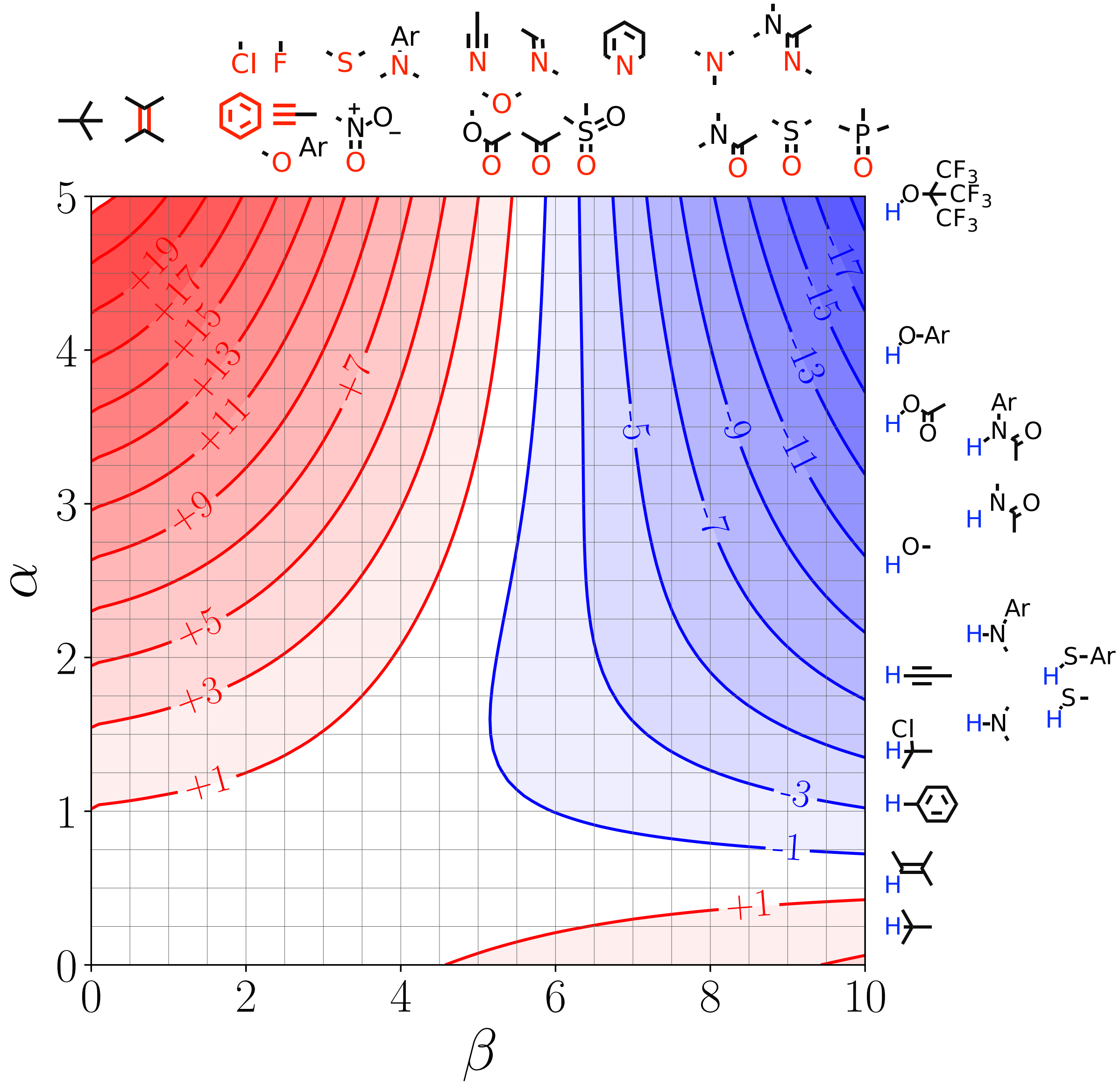

Figure S286: FGIP for 5.0% chloroform 95.0% tetrahydrofuran at 298K.

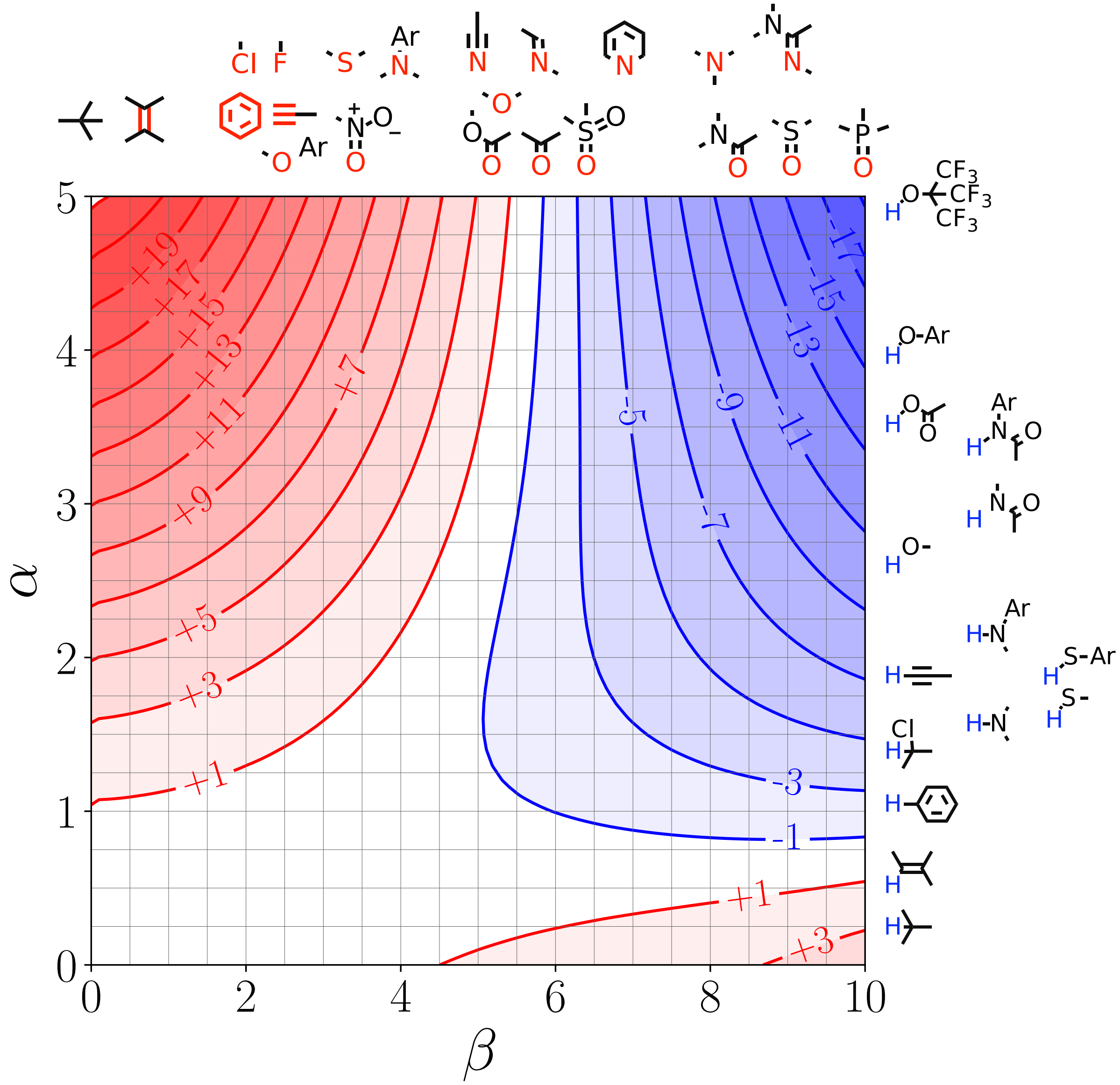

Figure S287: FGIP for 10.0% chloroform 90.0% tetrahydrofuran at 298K.

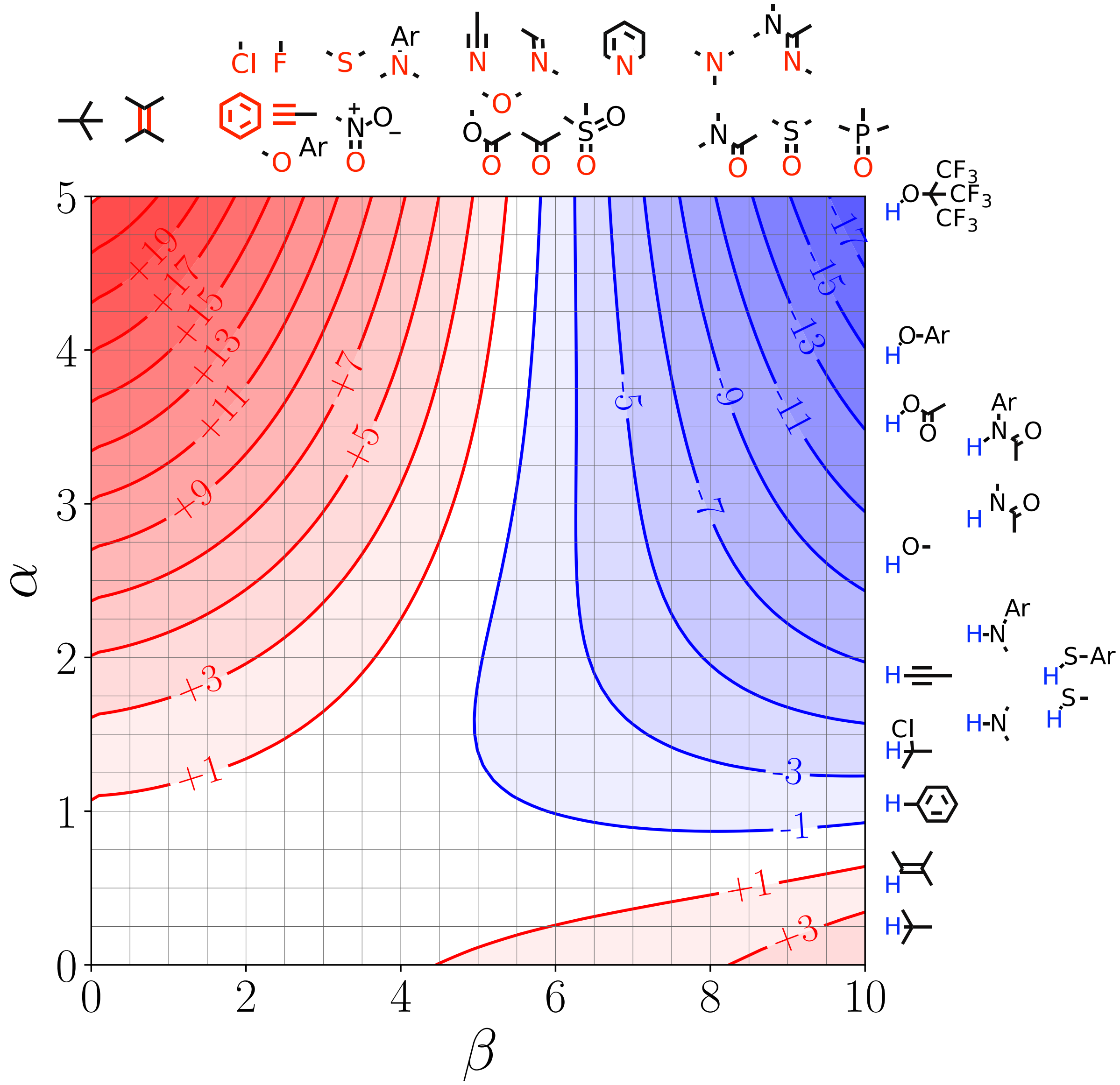

Figure S288: FGIP for 15.0% chloroform 85.0% tetrahydrofuran at 298K.

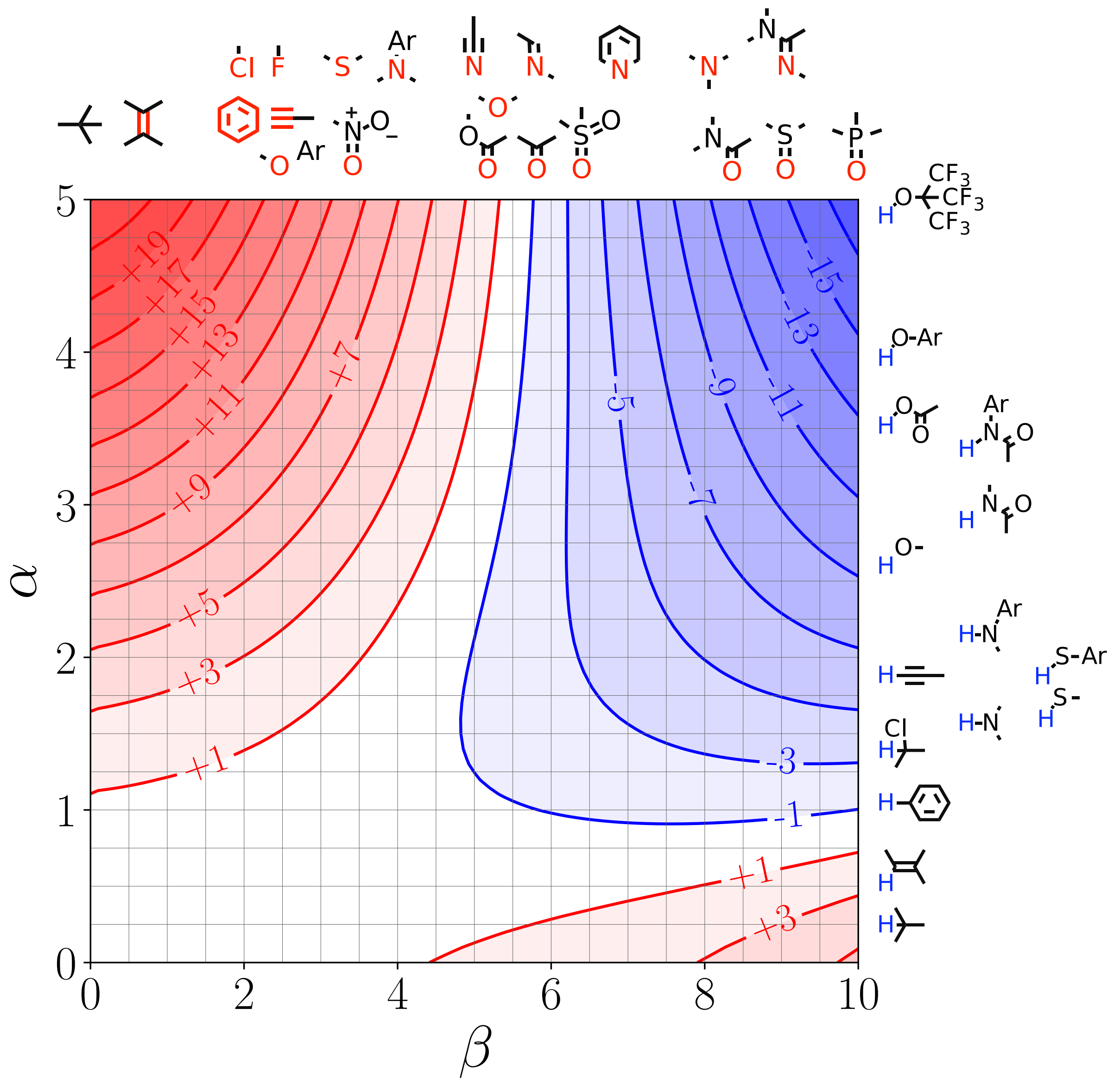

Figure S289: FGIP for 20.0% chloroform 80.0% tetrahydrofuran at 298K.

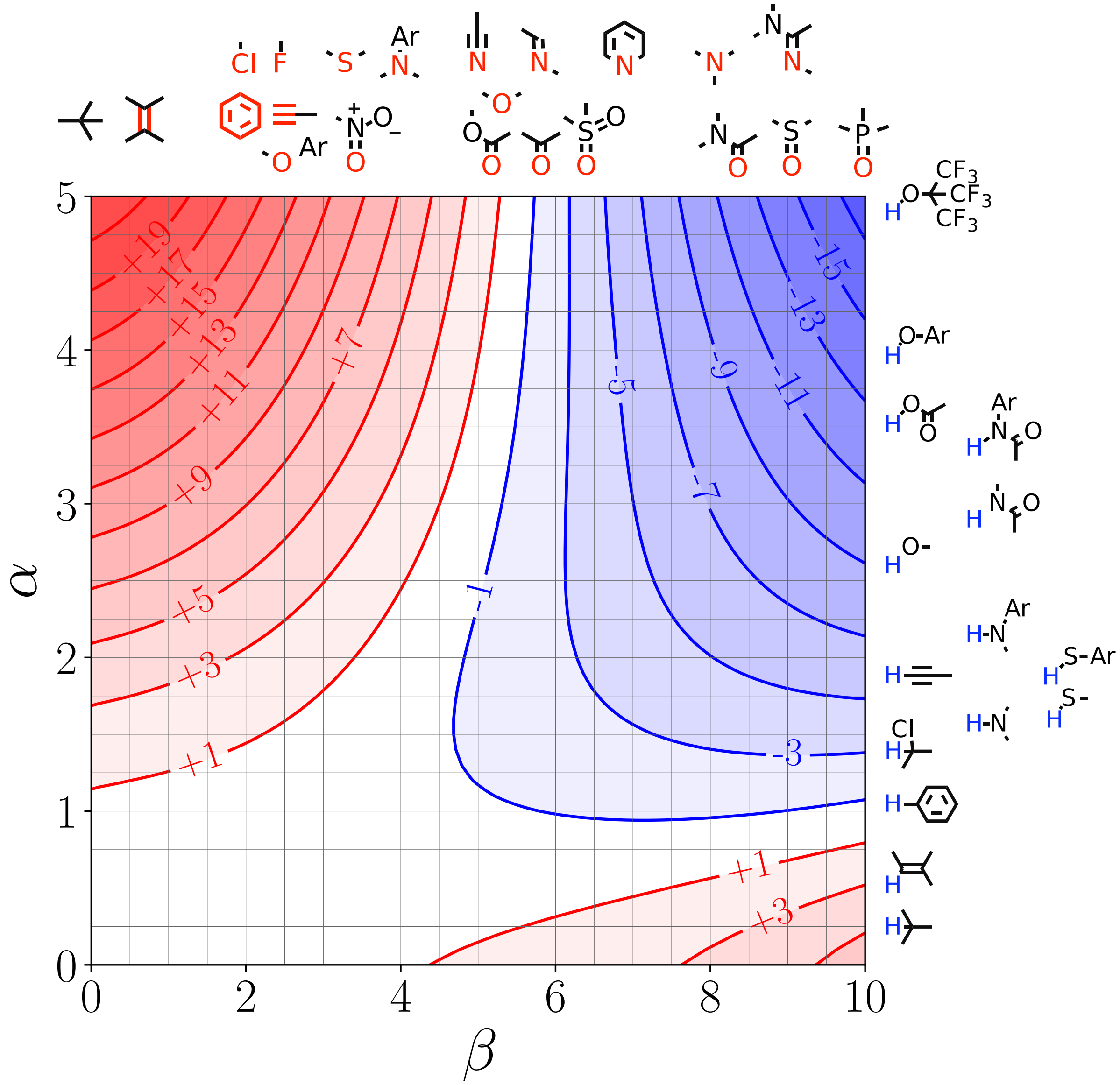

Figure S290: FGIP for 25.0% chloroform 75.0% tetrahydrofuran at 298K.

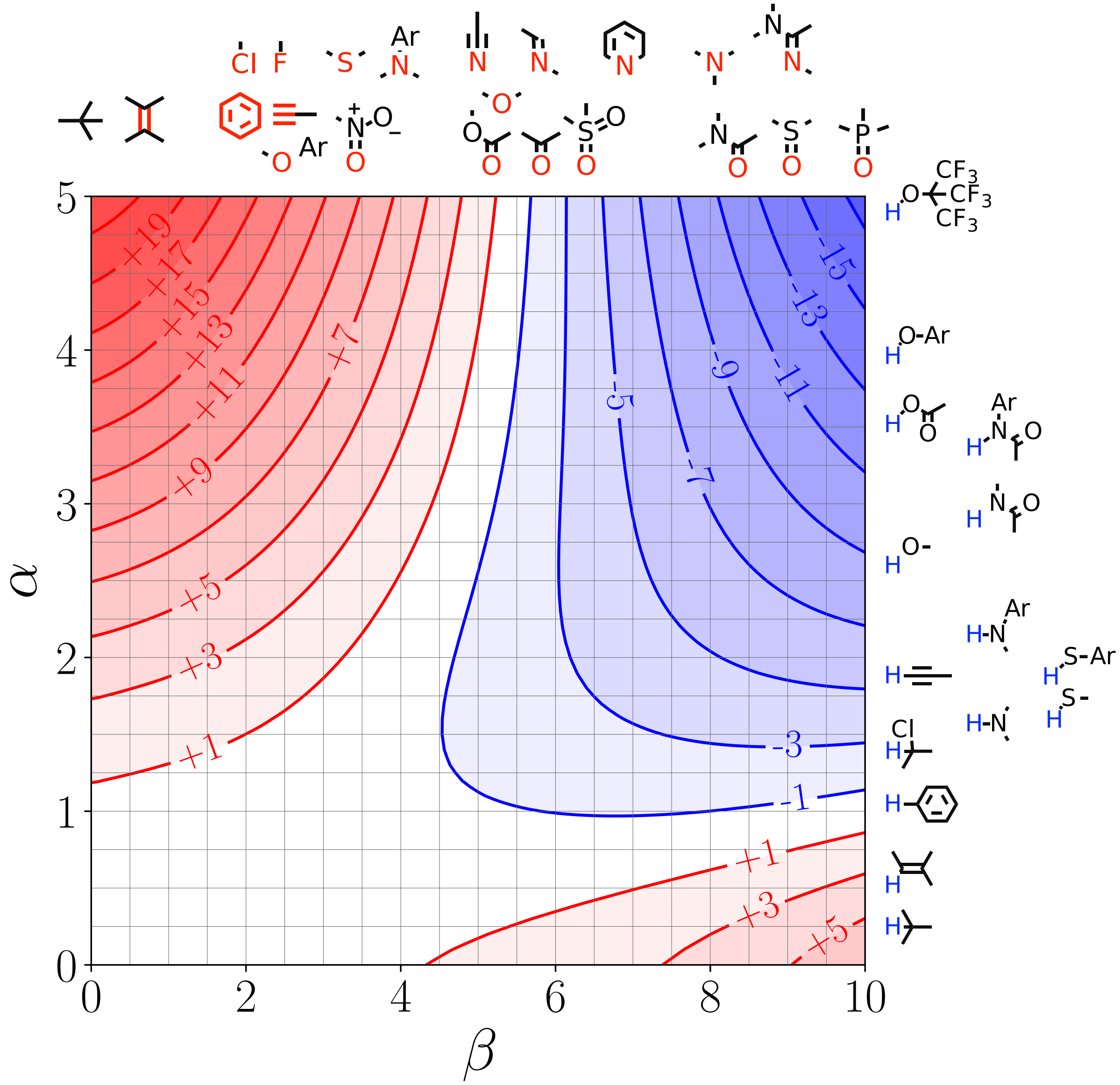

Figure S291: FGIP for 30.0% chloroform 70.0% tetrahydrofuran at 298K.

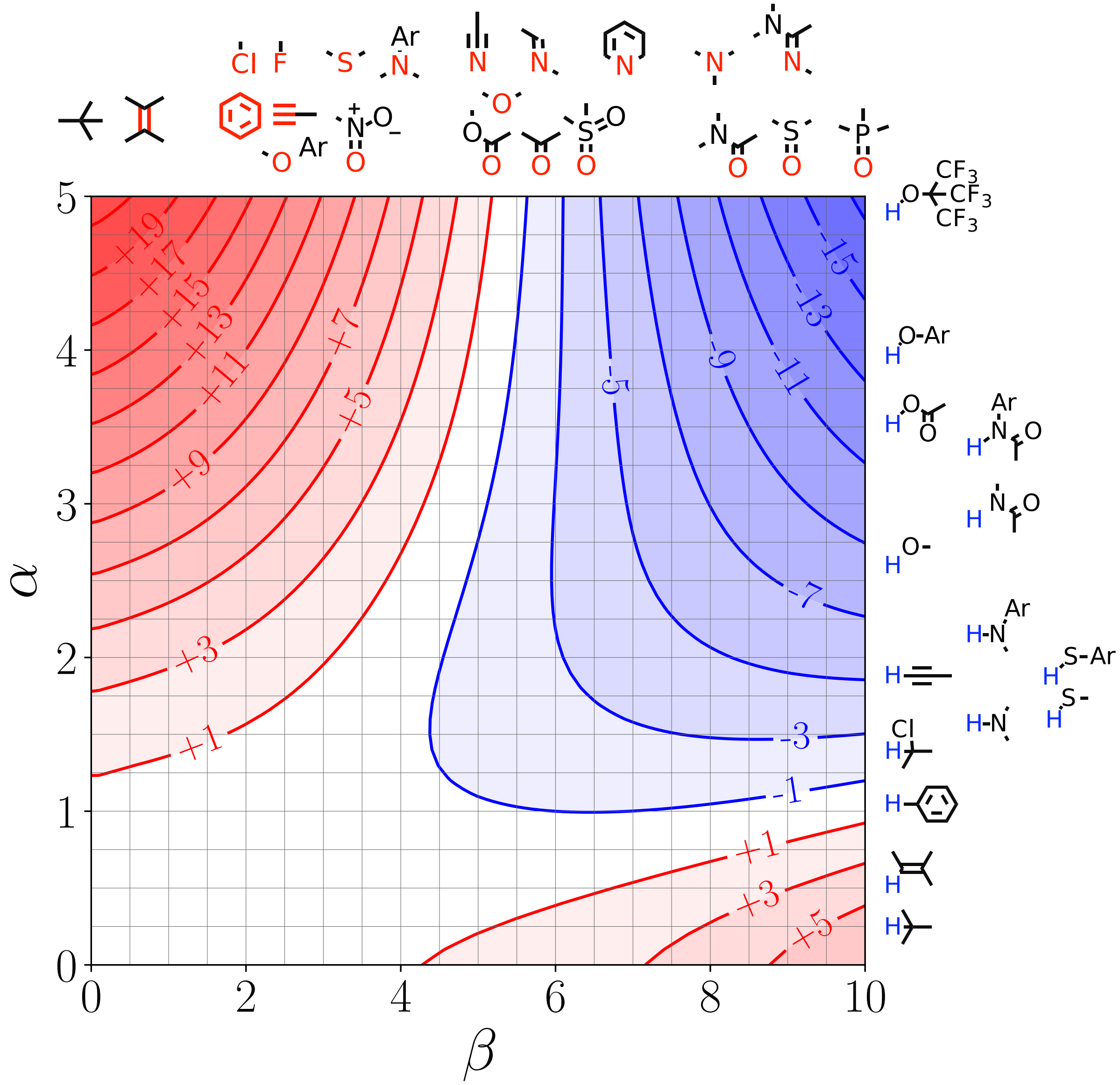

Figure S292: FGIP for 35.0% chloroform 65.0% tetrahydrofuran at 298K.

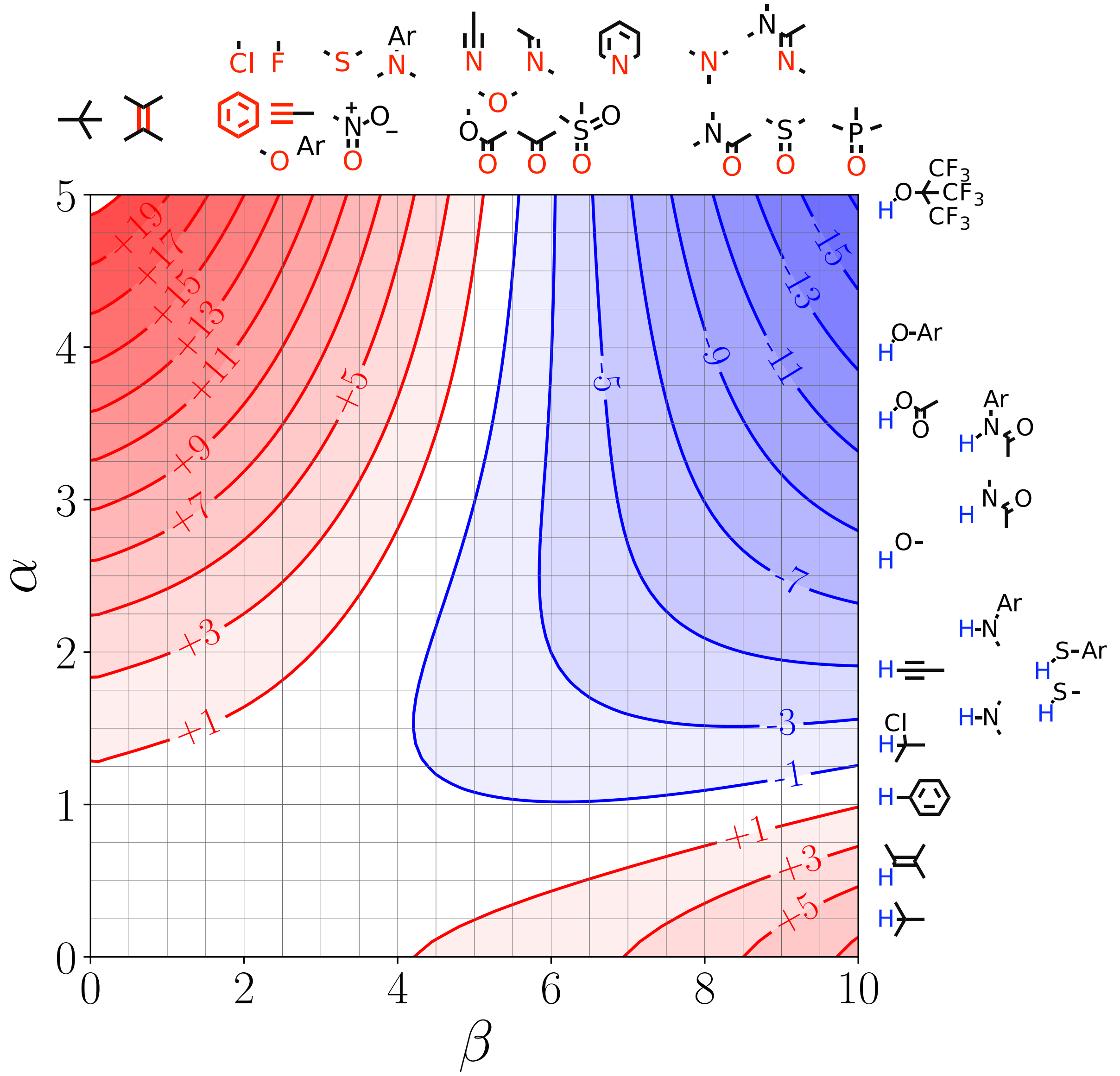

Figure S293: FGIP for 40.0% chloroform 60.0% tetrahydrofuran at 298K.

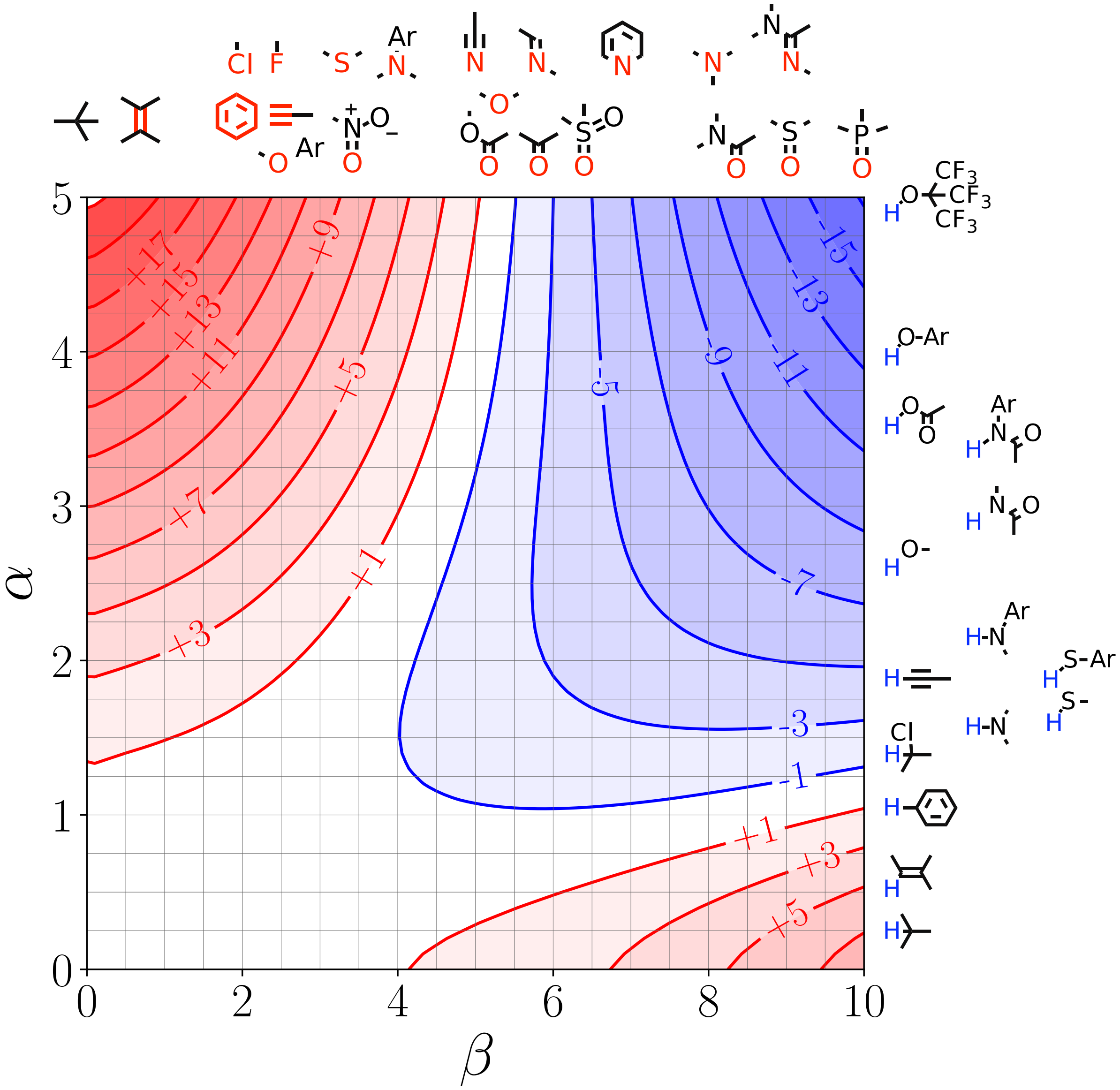

Figure S294: FGIP for 45.0% chloroform 55.0% tetrahydrofuran at 298K.

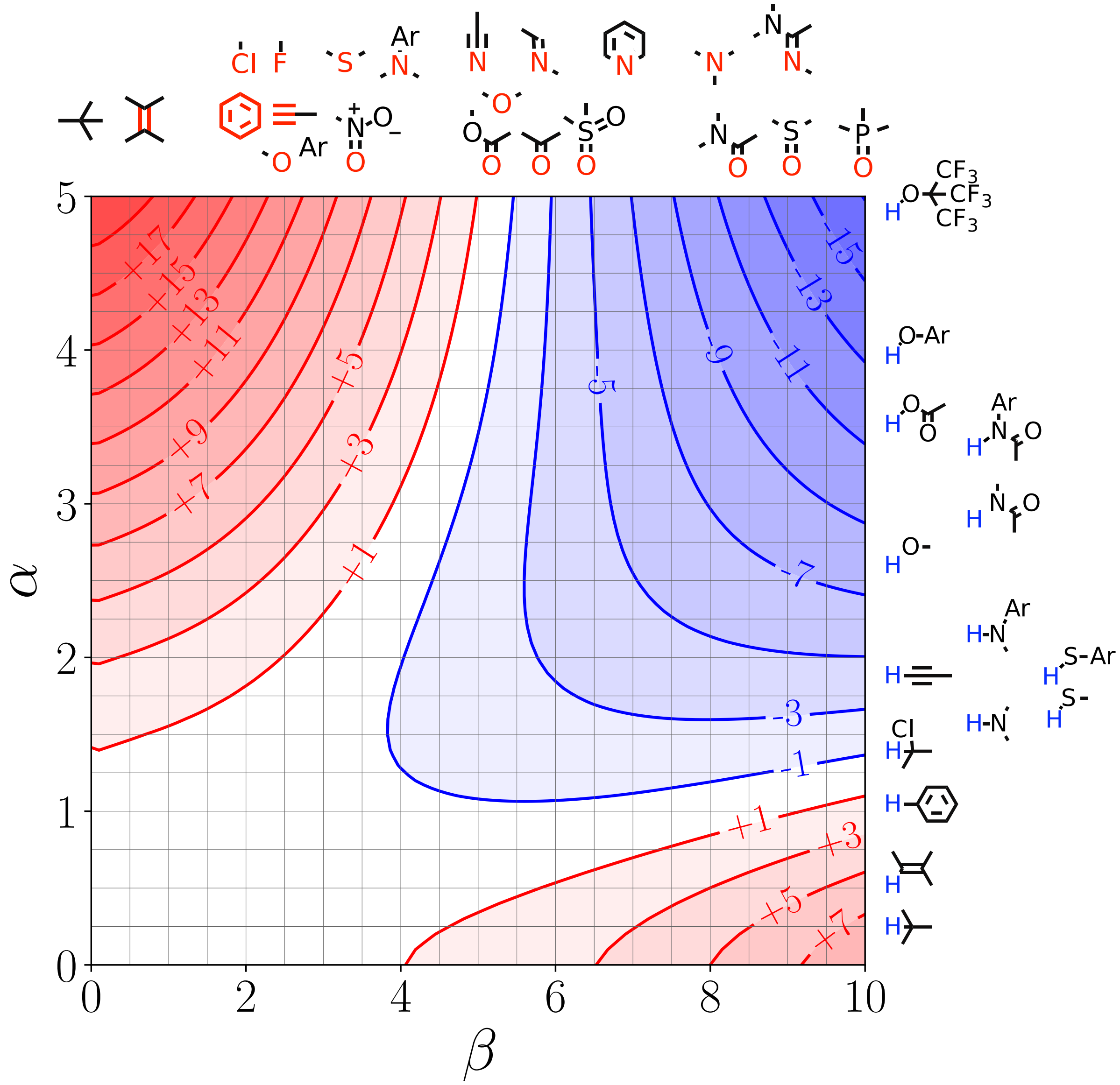

Figure S295: FGIP for 50.0% chloroform 50.0% tetrahydrofuran at 298K.

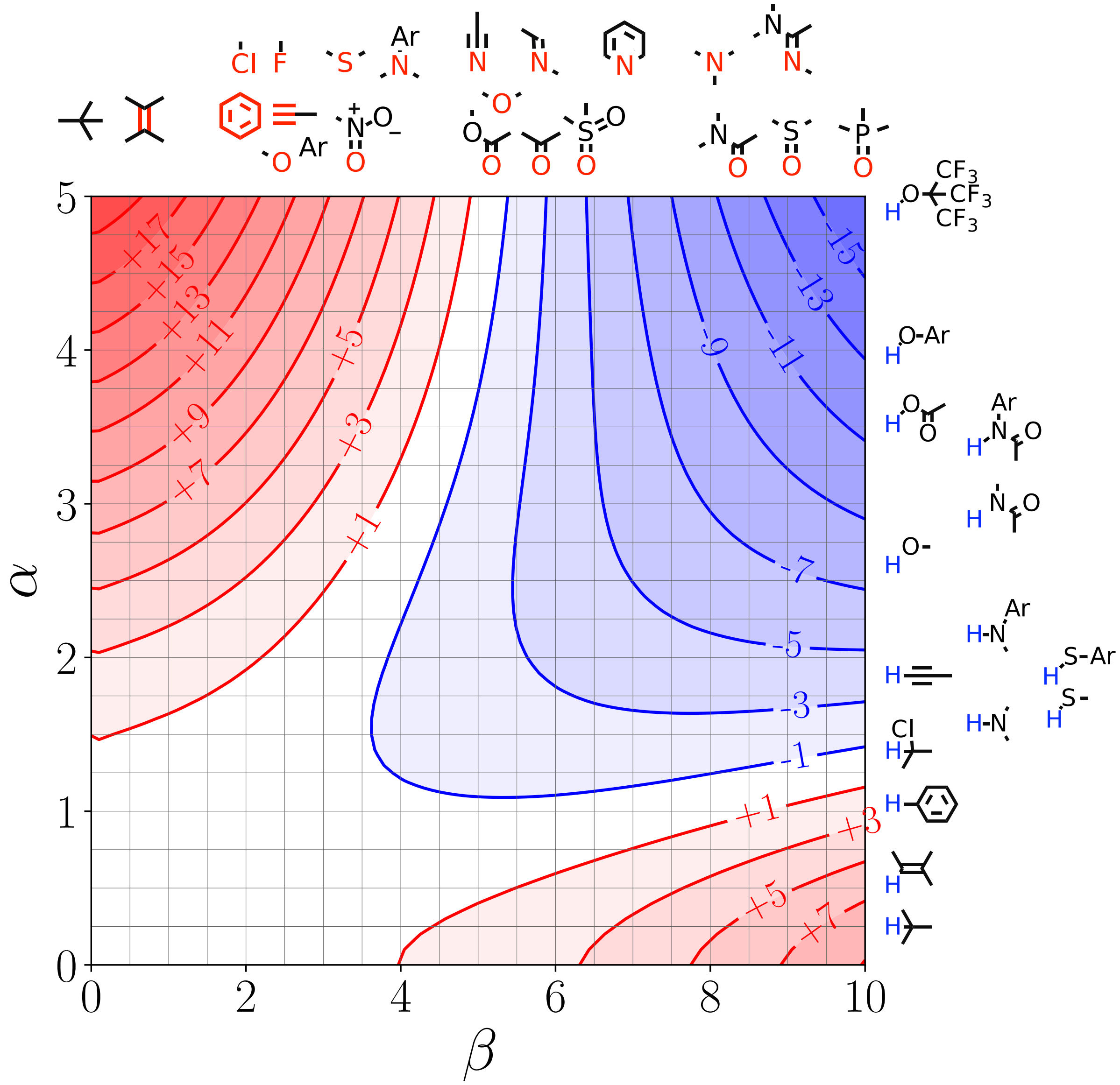

Figure S296: FGIP for 55.0% chloroform 45.0% tetrahydrofuran at 298K.

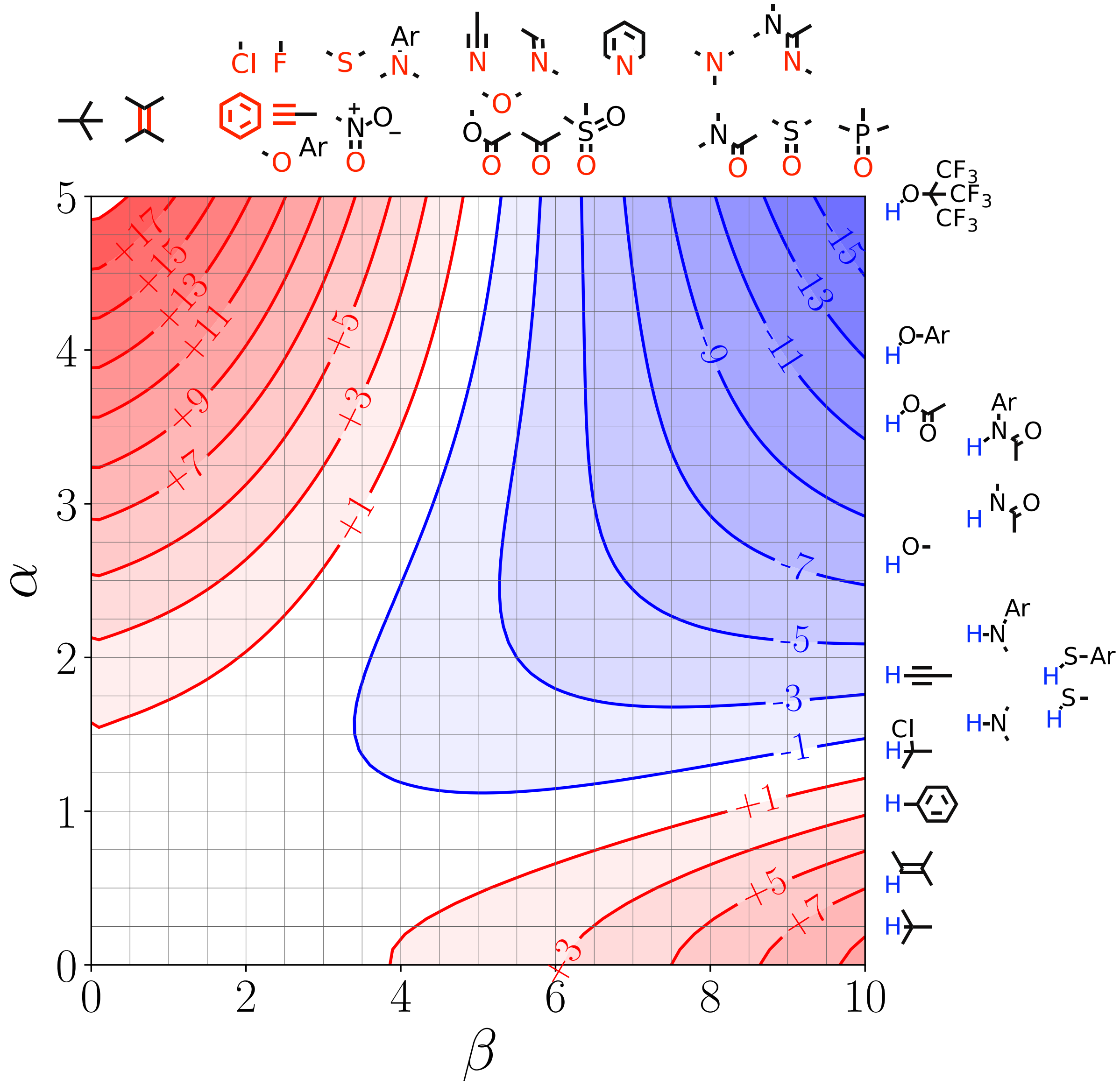

Figure S297: FGIP for 60.0% chloroform 40.0% tetrahydrofuran at 298K.

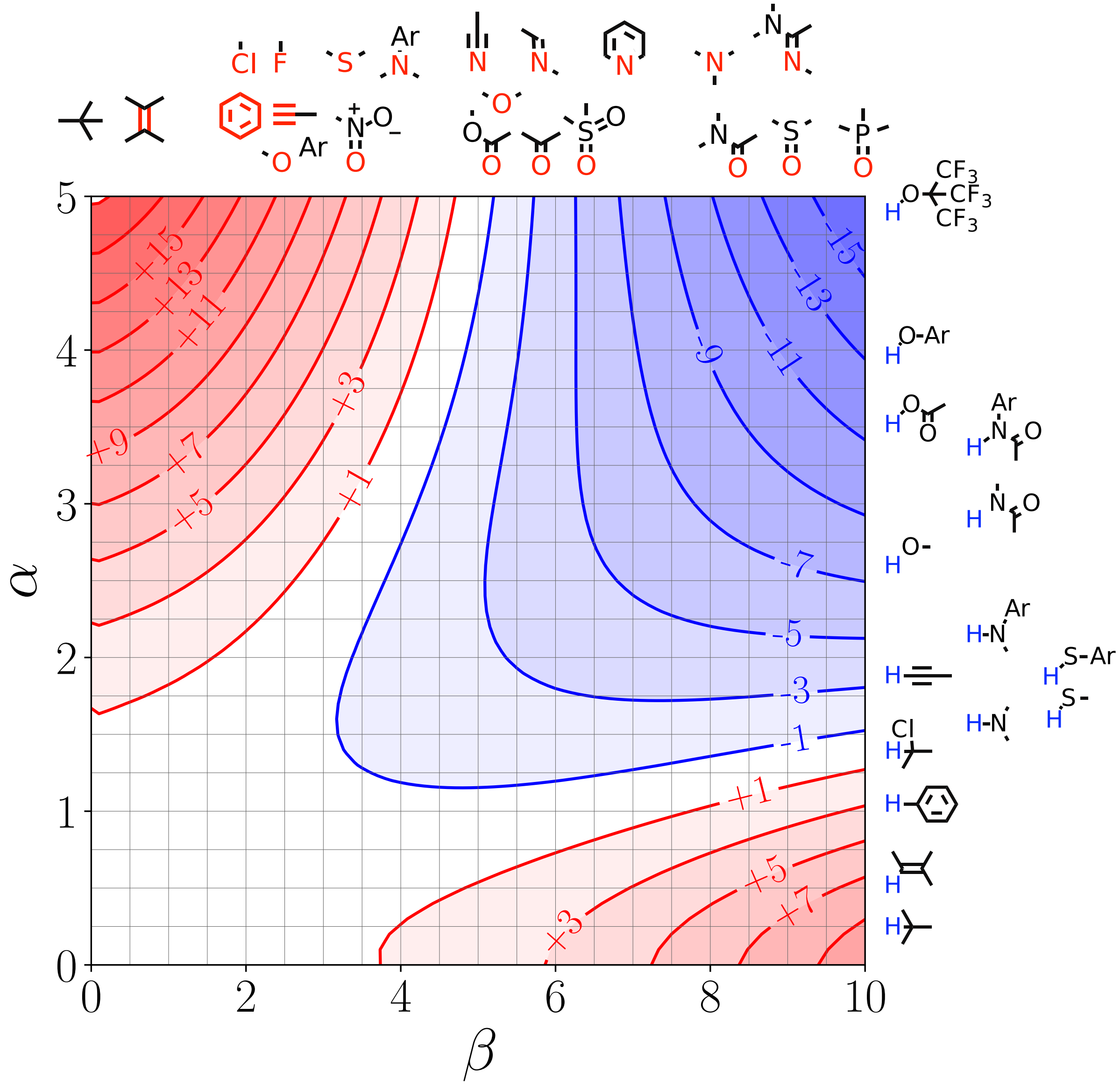

Figure S298: FGIP for 65.0% chloroform 35.0% tetrahydrofuran at 298K.

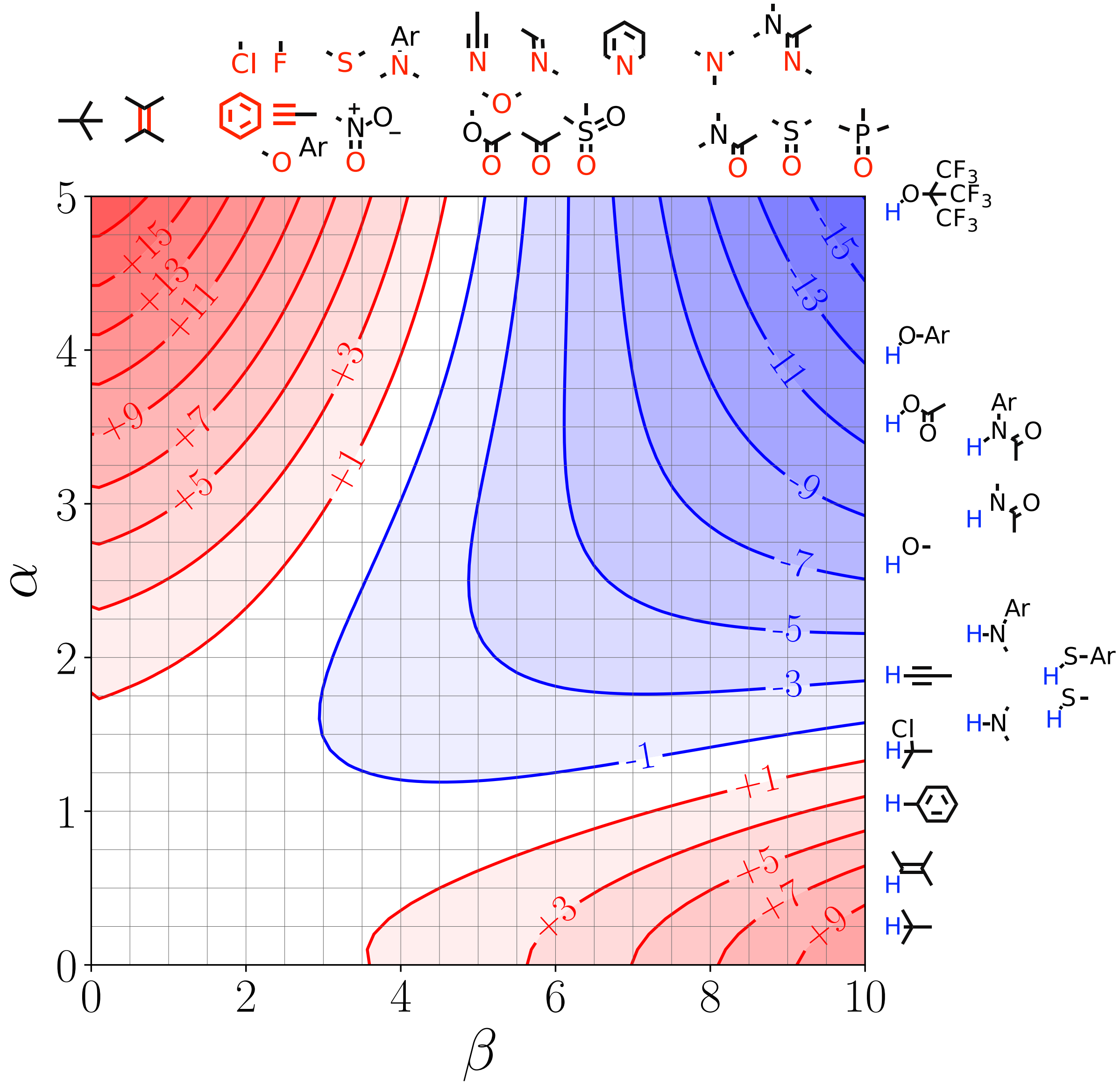

Figure S299: FGIP for 70.0% chloroform 30.0% tetrahydrofuran at 298K.

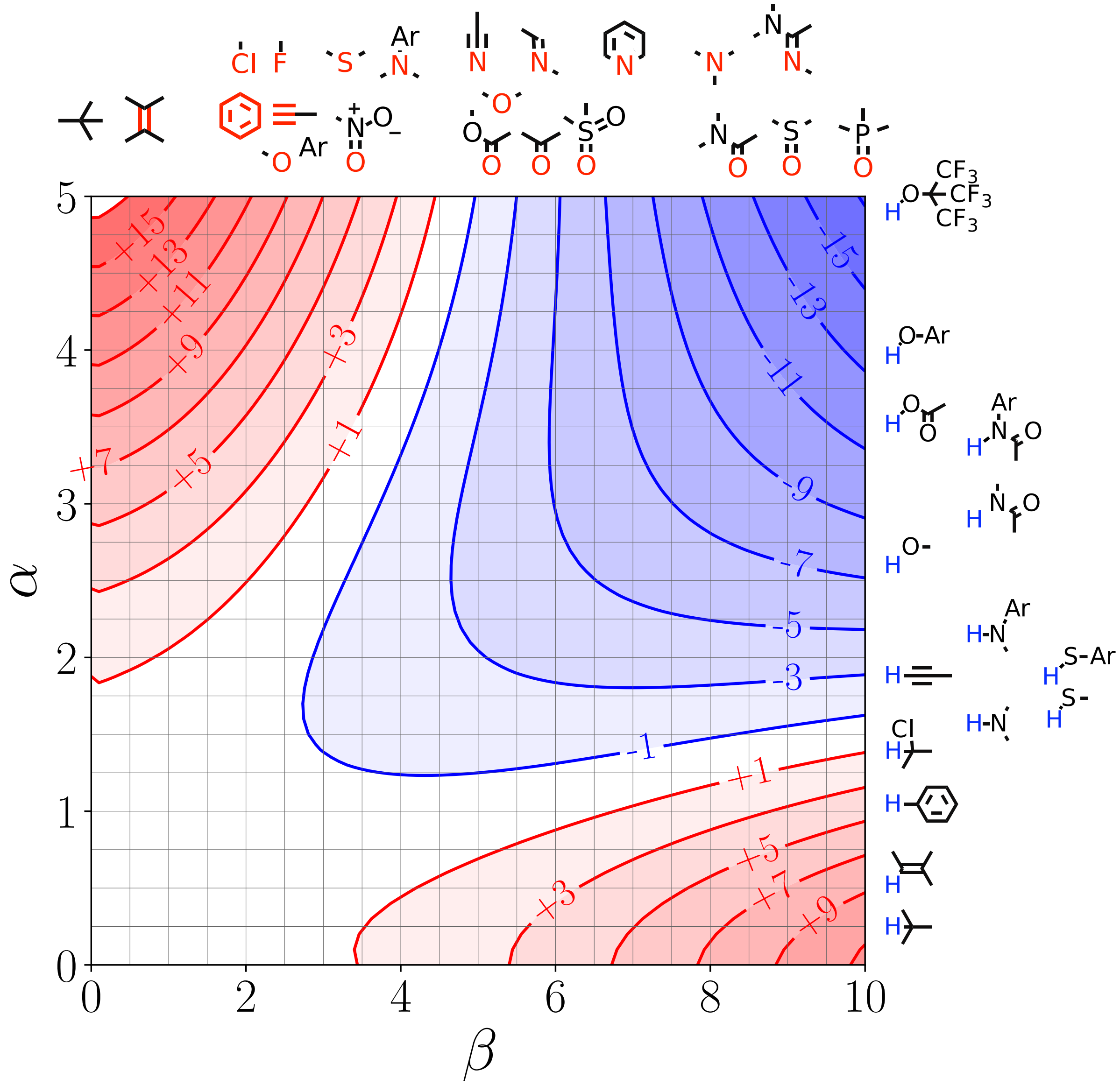

Figure S300: FGIP for 75.0% chloroform 25.0% tetrahydrofuran at 298K.

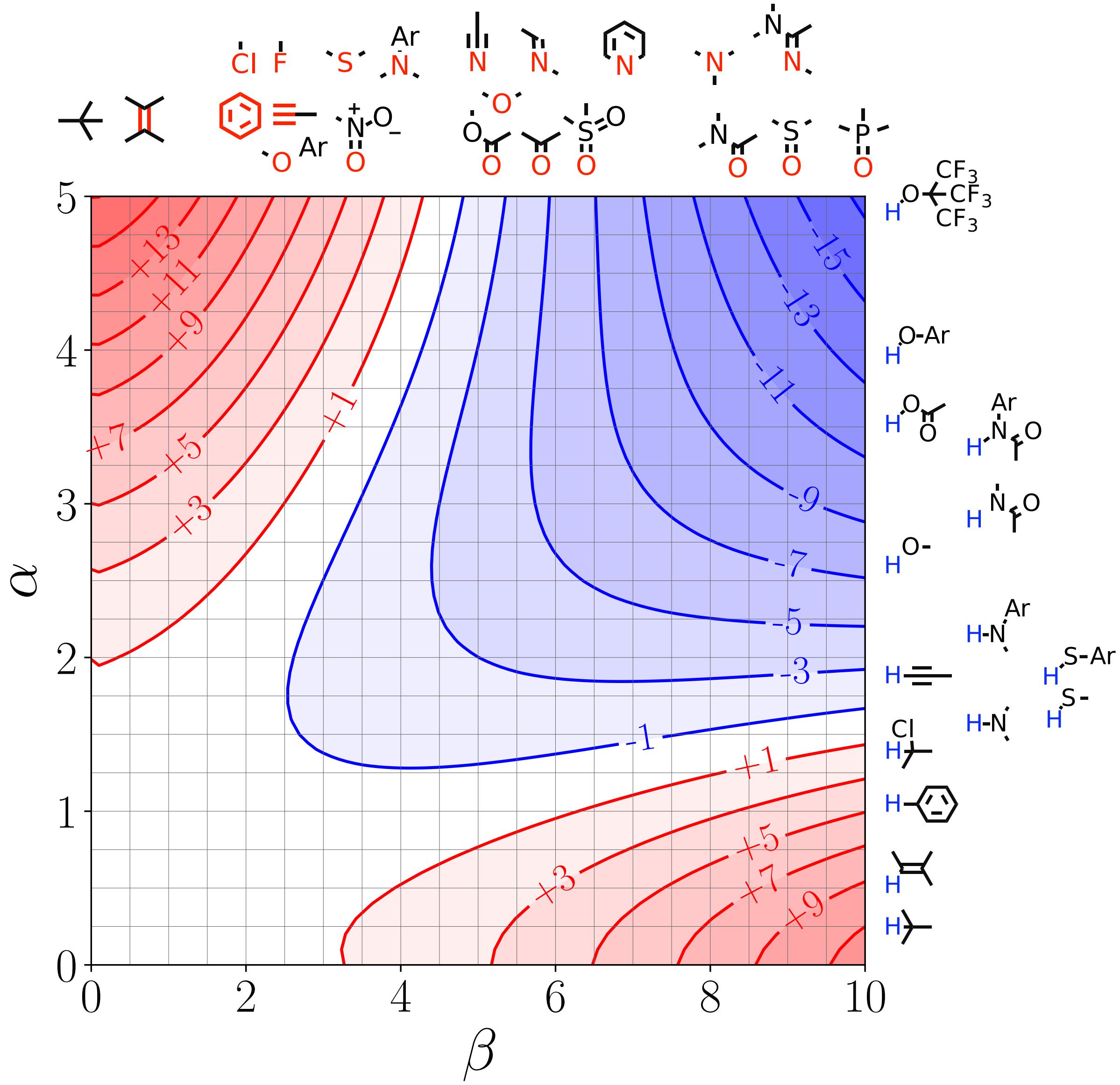

Figure S301: FGIP for 80.0% chloroform 20.0% tetrahydrofuran at 298K.

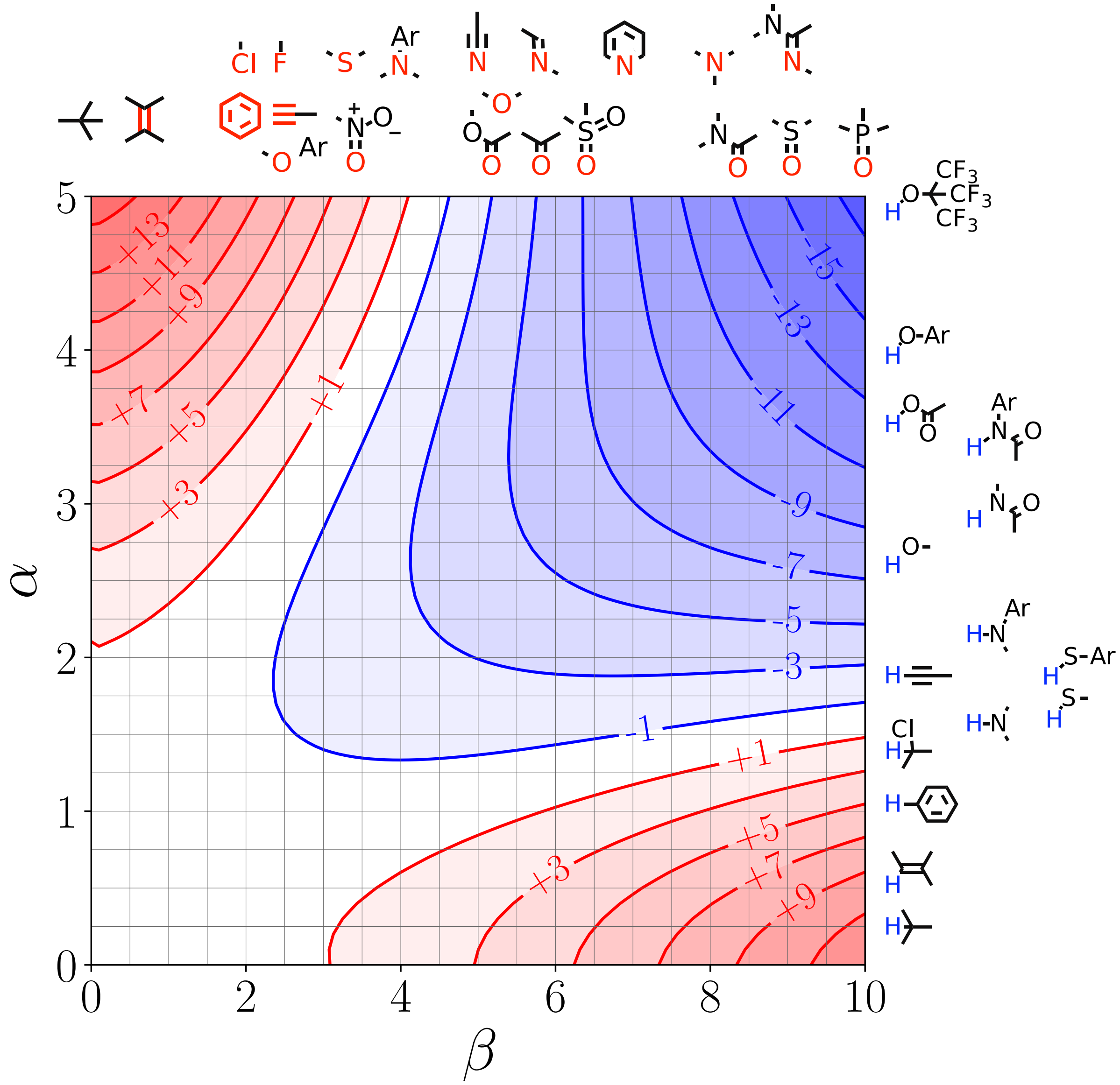

Figure S302: FGIP for 85.0% chloroform 15.0% tetrahydrofuran at 298K.

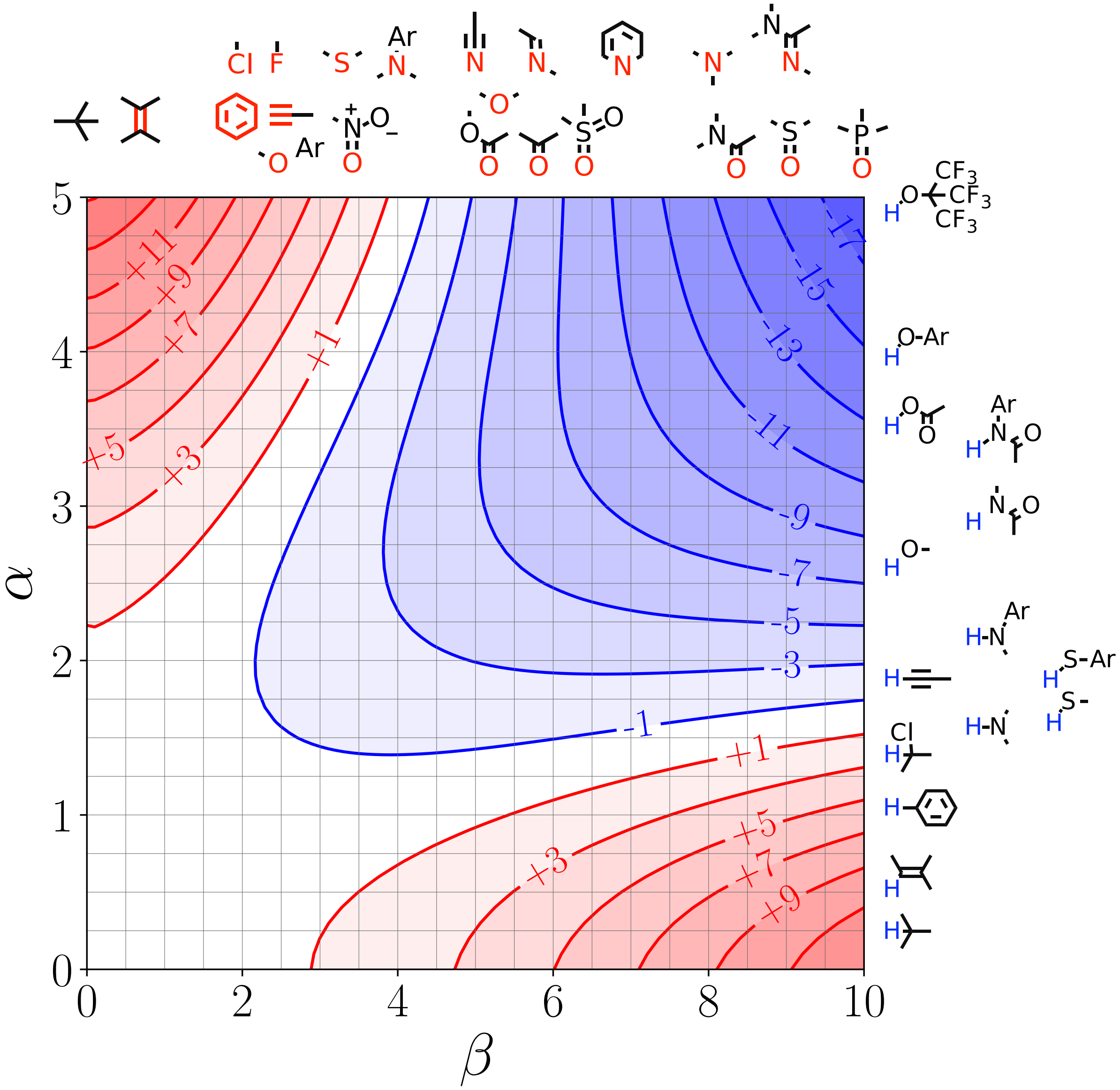

Figure S303: FGIP for 90.0% chloroform 10.0% tetrahydrofuran at 298K.

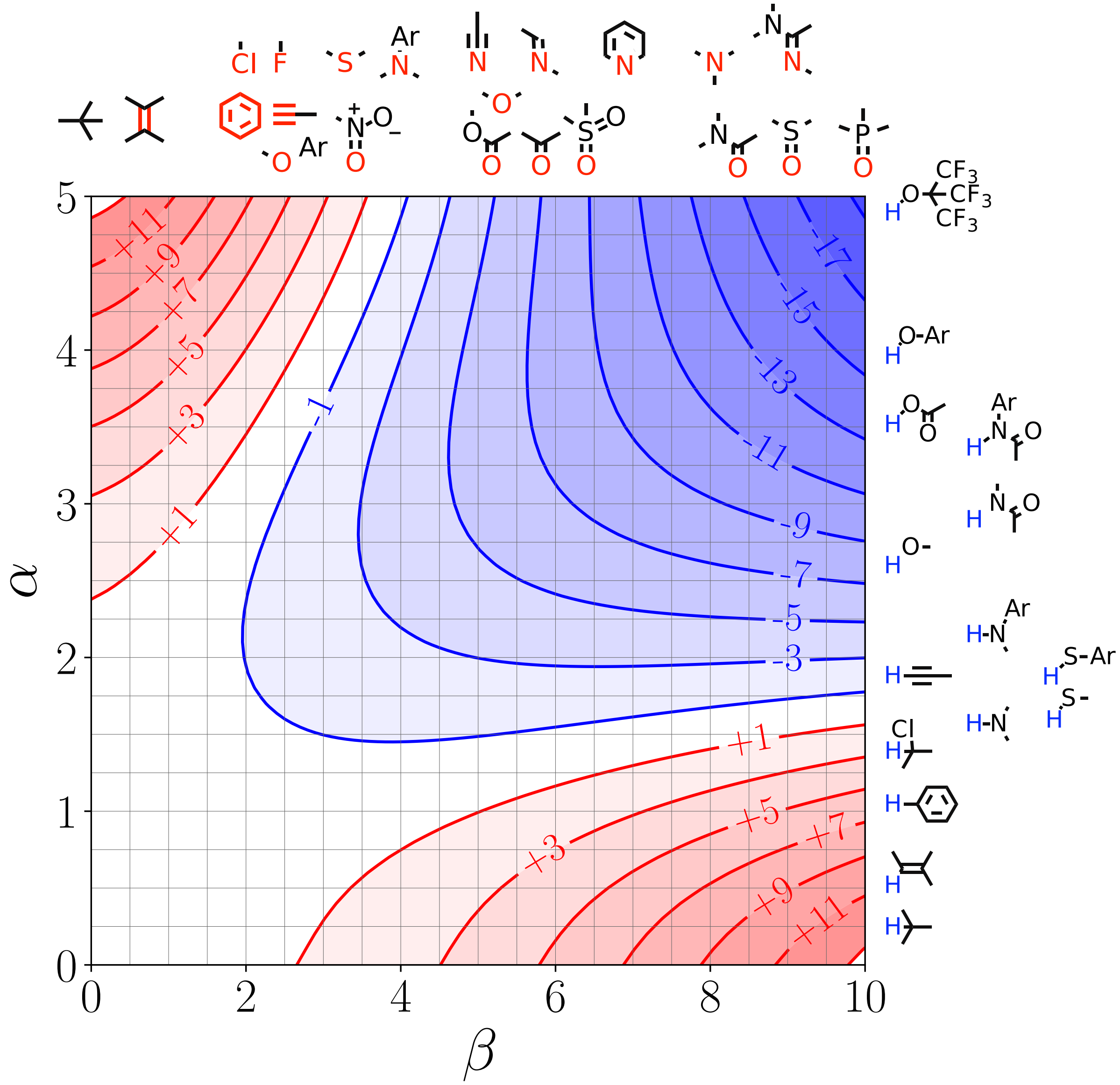

Figure S304: FGIP for 95.0% chloroform 5.0% tetrahydrofuran at 298K.

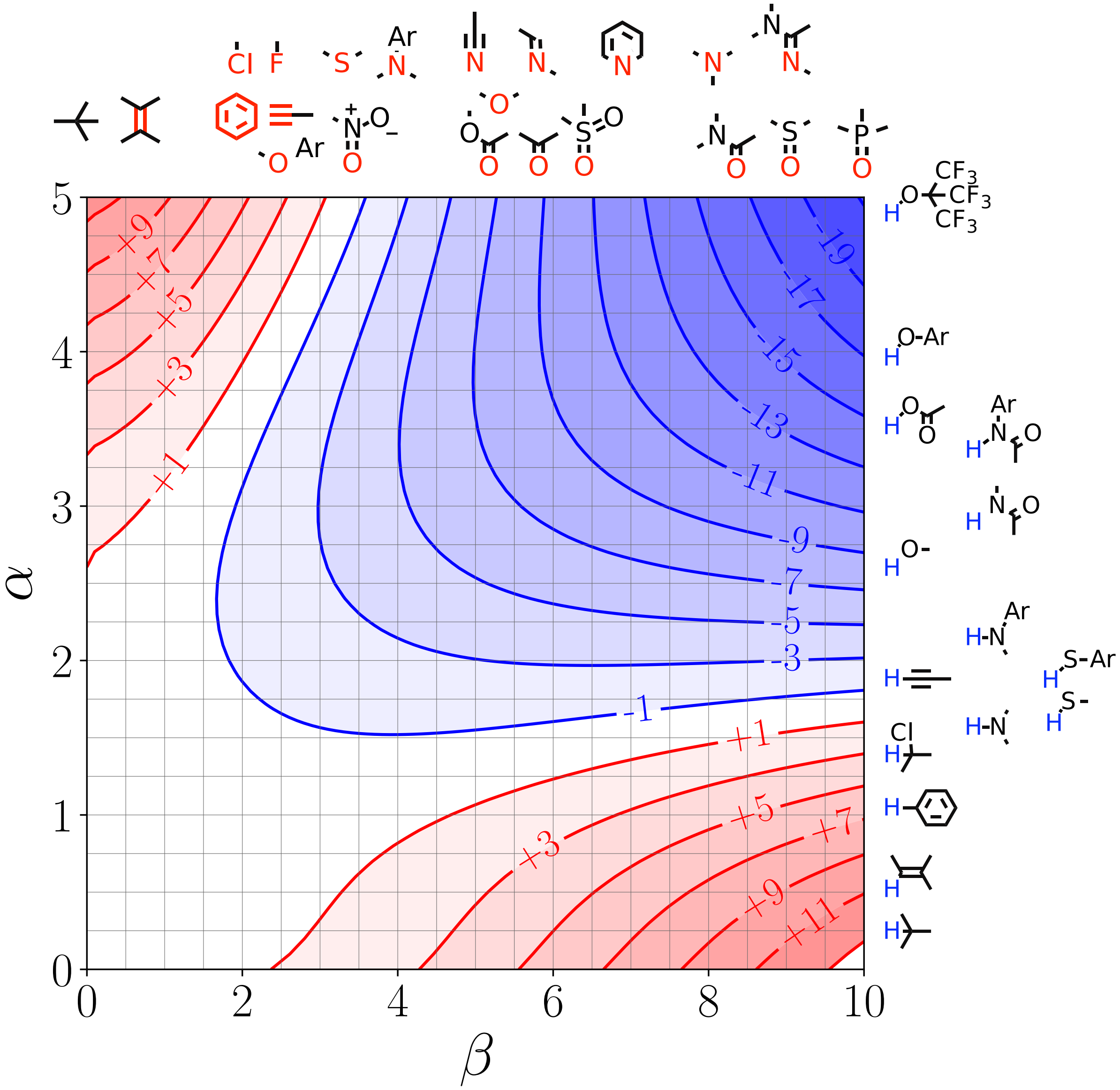

Figure S305: FGIP for 100.0% chloroform 0.0% tetrahydrofuran at 298K.

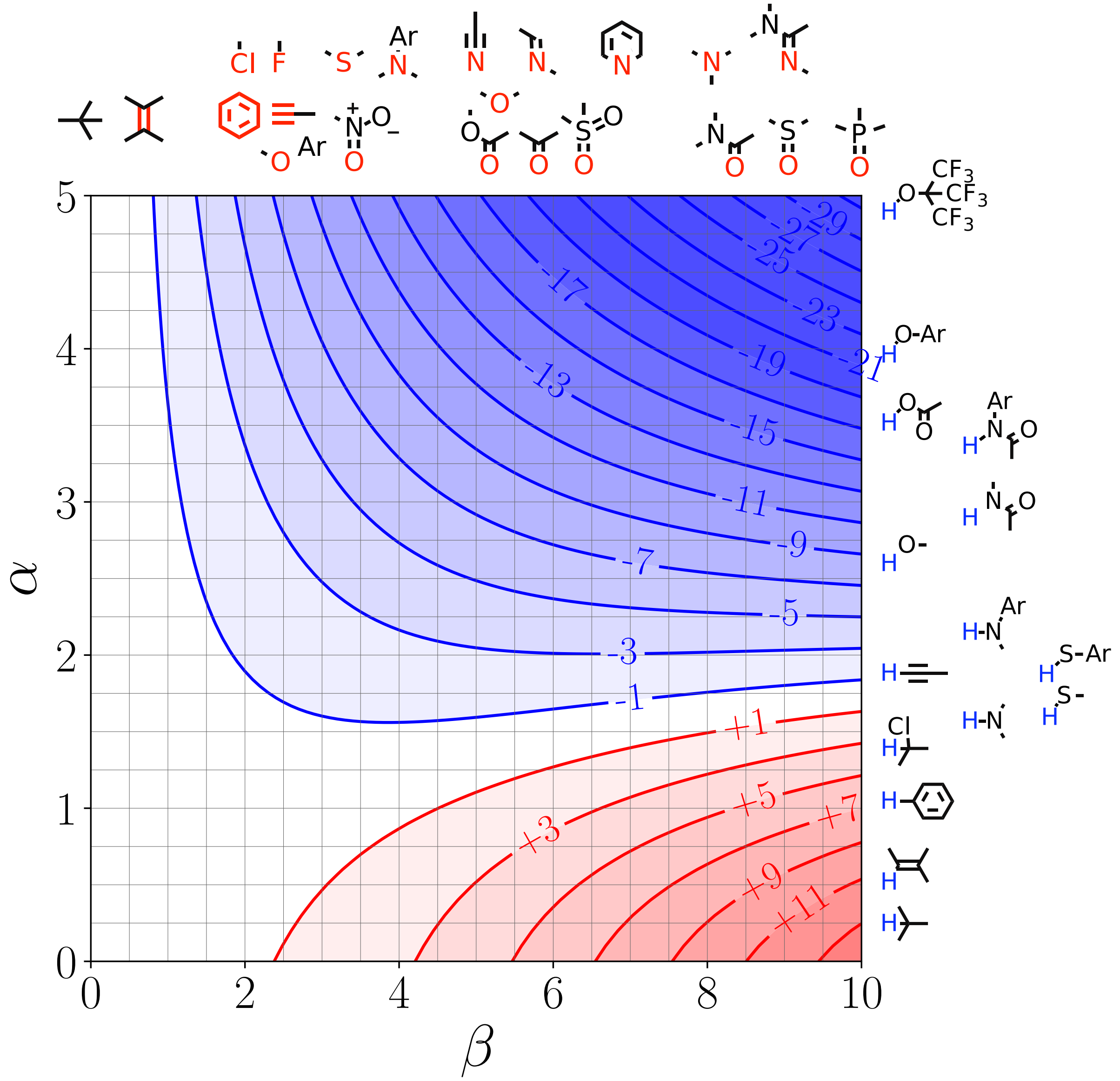

References

(1) Marcus, Y. *The Properties of Solvents*; Wiley, 1998.

(2) Sigma Aldrich, Cyrene product entry. 2019; `https://www.sigmaaldrich.com/catalog/product/sial/807796?lang=en&region=GB`.

(3) Calero, C. S.; Farwer, J.; Gardiner, E. J.; Hunter, C. A.; Mackey, M.; Scuderi, S.; Thompson, S.; Vinter, J. G. Footprinting molecular electrostatic potential surfaces for calculation of solvation energies. *Phys. Chem. Chem. Phys.* **2013**, *15*, 18262–73.

(4) Hunter, C. A. Quantifying intermolecular interactions: Guidelines for the molecular recognition toolbox. *Angew. Chem., Int. Ed.* **2004**, *43*, 5310–5324.

(5) McKenzie, J.; Feeder, N.; Hunter, C. A. H-bond competition experiments in solution and the solid state. *CrystEngComm* **2016**, *18*, 394–397.
